# Rhodium-catalysed *ortho*-alkynylation of nitroarenes†

**DOI:** 10.1039/d1sc04527j

**Published:** 2021-10-11

**Authors:** Eric Tan, Marc Montesinos-Magraner, Cristina García-Morales, Joan Guillem Mayans, Antonio M. Echavarren

**Affiliations:** Institute of Chemical Research of Catalonia (ICIQ), Barcelona Institute of Science and Technology (BIST) Av. Països Catalans 16 43007 Tarragona Spain aechavarren@iciq.es; Departament de Química Analítica i Química Orgànica, Universitat Rovira i Virgili C/ Marcel·lí Domingo s/n 43007 Tarragona Spain

## Abstract

The *ortho*-alkynylation of nitro-(hetero)arenes takes place in the presence of a Rh(iii) catalyst to deliver a wide variety of alkynylated nitroarenes regioselectively. These interesting products could be further derivatized by selective reduction of the nitro group or palladium-catalysed couplings. Experimental and computational mechanistic studies demonstrate that the reaction proceeds *via* a turnover-limiting electrophilic C–H metalation *ortho* to the strongly electron-withdrawing nitro group.

## Abstract 2

Rh(iii)-catalyzed *ortho*-alkynylation of nitro-(hetero)arenes leads to a wide variety of alkynylated nitroarenes *via* a turnover-limiting electrophilic C–H *ortho*-metalation.

## Graphical abstract

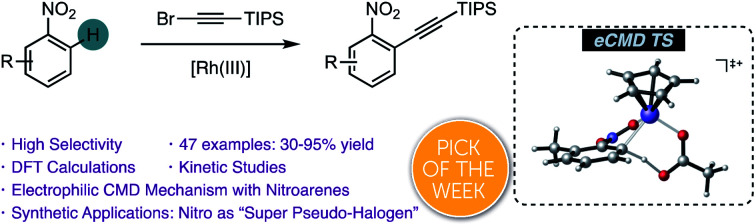

## Introduction

Nitrobenzenes are among the most important bulk chemicals, used in a range of applications such as dyes, organic materials, solvents and perfumes.^1^ With a price comparable to benzene, nitrobenzene serves as precursor to most of the functionalized aromatic building blocks. Therefore, the development of methods for the functionalization of nitrobenzenes is of high interest.

**Scheme 1:**
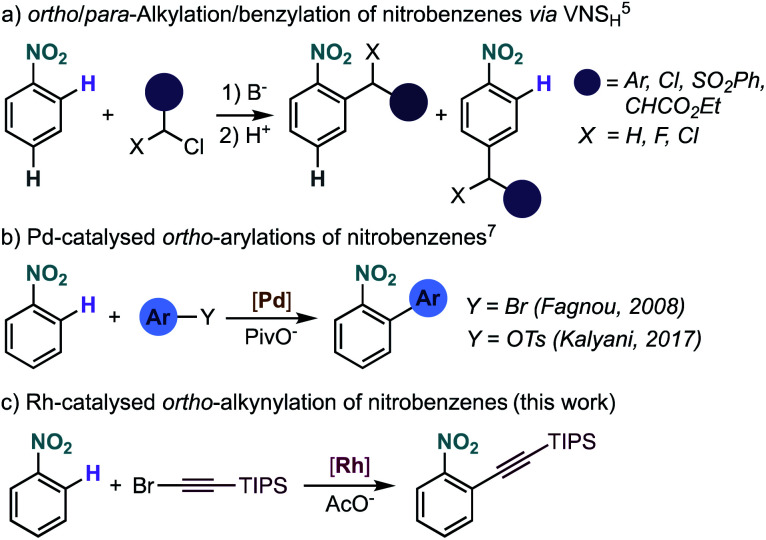
Intermolecular *ortho*-functionalization of nitrobenzenes and present studies. VNSH = vicarious nucleophilic substitution of hydrogen.

Nitrobenzenes can be functionalized at the *ipso*-position by reduction to an aniline and formation of the diazonium salt, allowing access to versatile aryl halides (Sandmeyer reaction).^2^ More recently, the functionalization of nitrobenzenes gained momentum with the discovery that rhodium,^3^ copper,^4^ and palladium^5^ complexes can undergo oxidative addition to nitrobenzenes, opening the door to their use as coupling partners in transition-metal catalysis. Nitrobenzenes are usually functionalized at the *meta*-position *via* electrophilic aromatic substitution, although functionalization at the *ortho*- and *para*-positions is also possible *via* the so-called vicarious nucleophilic substitution (Scheme 1a),^1,6^ where an α-halo-carbanion generated from an active methylene compound adds to the *ortho*- and/or *para*-position. However, this method is mainly limited to alkylation-type functionalization and often requires electronically activated nitrobenzenes to achieve synthetically useful yields and good selectivity. Therefore, the selective *ortho*-functionalization of unbiased nitrobenzene derivatives is an important and yet underdeveloped transformation in organic chemistry.

The development of metal-catalysed directed C–H functionalization reactions has become an efficient method for the synthesis of functionalized arenes. Even though the ability of the ubiquitous nitro group to coordinate to metals is known,^7^ its use as directing group in catalysis is rare and limited to direct arylation.^8^ Our group observed the intramolecular Pd-catalysed *ortho* C–H arylation of nitrobenzene derivatives, although in that reaction the nitro group was not acting as a regiodirecting substituent.^9*a*^ The first genuine intermolecular Pd-catalysed direct arylation of nitrobenzenes was achieved using aryl halides^8*a*^ and tosylates,^8*b*^ although an excess of nitrobenzene was required (Scheme 1b). The *ortho* C–H arylation of nitro-substituted nitrogen heteroarenes has been developed to a greater extent, but, in these examples, the site-selectivity is a consequence of the heteroarene electronics and not of the regiodirecting effect of the nitro group.^10^

Here we report the Rh-catalysed nitro-directed C–H alkynylation of nitrobenzenes, which tolerates a broad range of functional groups (Scheme 1c).^11^ Our experimental and computational mechanistic investigations are consistent with a turnover limiting electrophilic C–H activation step, followed by alkyne insertion and bromide elimination. We also disclose preliminary results on a related C–H iodination reaction, which may pave the way for other types of *ortho*-functionalization *via* sequential Rh/Pd catalysis.

## Results and discussion

As part of our research program on the selective C–H alkynylation of functionalized molecules, we recently found that the combination of a Cp*Rh(iii) catalyst and bromo-alkyne **2a** is a highly active system for the alkynylation of a broad-range of C–H bonds.^12^ Our initial attempts to extend this reactivity to 2-methylnitrobenzene (**1a**) at 50 °C proved unsuccessful (Table 1, entry 1). Interestingly, formation of the corresponding alkynylated product **3a** could be observed at 80 °C as the only product (Table 1, entry 2). A further increase of the temperature to 110 °C led to the formation of **3a** in 95% yield (Table 1, entry 3). Control experiments showed the essential role of all reaction components (Table 1, entries 4–7). Other catalysts frequently used in C–H functionalization, such as MnBr(CO)_5_, Cp*Co(CO)I_2_, Pd(OAc)_2_, [RuCl_2_(*p*-cymene)]_2_ or [Cp*IrCl_2_]_2_ were inactive (Table 1, entry 8). The use of different silver salts (Table 1, entry 9), solvents (Table 1, entry 10), or carboxylate salts (Table 1, entry 11) led to unreactive catalytic systems. Regarding the alkyne counterpart, switching bromine for chlorine (**2b**) did not affect the reaction outcome (Table 1, entry 12). However, using iodo-alkyne **2c** led to the formation of **3a** in low yield (Table 1, entry 13).

**Table 1:** Rh-catalysed *ortho*-alkynylation of **1a**

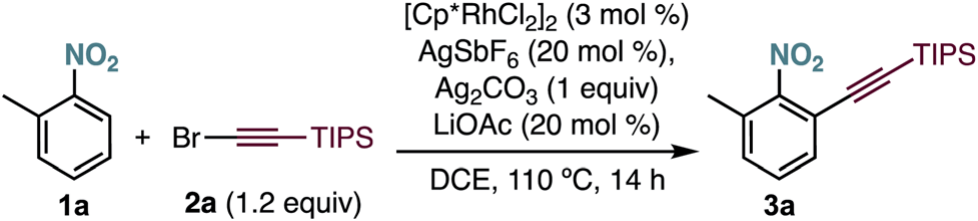		
Entry	Deviation from optimized conditions	Yield **3a**^a^ (%)
1	50 °C	0
2	80 °C	35^b^
3	None	95^b^
4	Without [Cp*RhCl_2_]_2_	0
5	Without Ag_2_CO_3_	0
6	Without LiOAc	0
7	Without AgSbF_6_	0
8	MnBr(CO)_5_, Cp*Co(CO)I_2_, Pd(OAc)_2_, [Cp*IrCl_2_] or [RuCl_2_(*p*-cymene)]_2_ instead of [Cp*RhCl_2_]_2_	0
9	With AgNO_3_ or Ag_2_O instead of Ag_2_CO_3_	0
10	THF or *tert*-amyl alcohol instead of DCE	0
11	NaOPiv instead of LiOAc	0
12	TIPS-Cl-acetylene (**2b**) instead of **2a**	96
13	TIPS-I-acetylene (**2c**) instead of **2a**	10

a Yield determined by ^1^H NMR with an internal standard.

b Isolated yield.

**Scheme 2:**
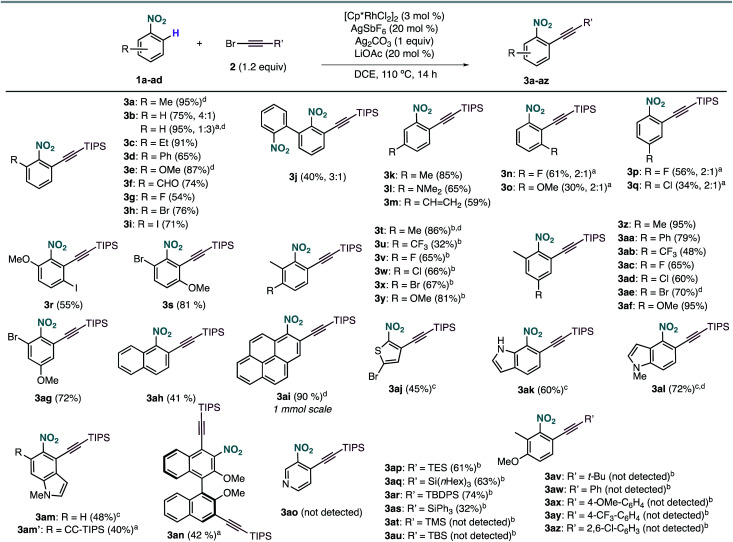
Rh-catalyzed *ortho*-alkynylation of nitrobenzenes. Yields of isolated products in parentheses. In cases in which dialkynylated products were also formed, mono- *vs.* dialkynylation selectivity is shown in parentheses. ^*a*^With 2 equiv. of bromo-alkyne. ^*b*^At 100 °C. ^*c*^With 2 equiv. of nitro-heteroarene. ^*d*^4 h reaction time.

The scope of the Rh-catalysed alkynylation of nitrobenzenes was explored using the optimized conditions for the synthesis of **3a** (Scheme 2). Although the reactions were typically performed overnight for convenience, reaction times can be often reduced to just a few hours. Alkynylation of nitrobenzene was performed under the optimised reaction conditions to obtain a 4 : 1 mixture of mono- and dialkynylated product in 75% yield. This ratio can be reversed by increasing the amount of bromoalkyne **2a**, which results in the obtention of dialkynylated **3b′** in excellent yield. Functionalities such as alkyl (**3a**, **3c**), aryl (**3d**, **3j**), ether (**3e**), aldehyde (**3f**) and halides (**3g–i**), groups at the *ortho* position were well tolerated, leading to **3a–j** in 40–95% yield and complete *ortho*-selectivity. In the case of *meta*-substituted nitrobenzenes, the alkynylation occurred exclusively at the least hindered site leading to methyl, dimethyl amine or vinyl substituted compounds (**3k–m**) groups, whereas fluoro and methoxy substituents led to a 2 : 1 mixture of mono- and dialkynylated products, favoring the formation of 1,2,3-trisubstituted nitrobenzenes (**3n,o**). Regarding *para*-substituted derivatives, mixtures of mono- and dialkynylated products (**3p,q**) with complete *ortho*-selectivity were also obtained. Polysubstituted substrates could also be efficiently alkynylated leading to (**3r–ag**) in 32–95% yield with perfect regioselectivity. In this context, substituents such as alkyl, aryl, ether, halide, and even trifluoromethyl were employed in different substitution patterns. We followed our investigations with polyaromatic nitrobenzenes, which gave the corresponding products (**3ah**, **3ai**, **3an**) in moderate to excellent yields. The formation of dialkynylated compound **3an** might arise from a subsequent electrophilic alkynylation directed by the electron-donating MeO group. Importantly, alkynylated 1-nitropyrene **3ai** was obtained in 90% yield at 1 mmol scale in only 4 hours. Next, we applied our reaction conditions to the alkynylation of nitro-heteroarenes. Expectedly, π-deficient heteroarenes, such as 3-nitropyridine, were unreactive.^13^ On the other hand, when π-excessive nitro-thiophene (**1aj**) and differently substituted nitro-indoles (**1ak–am**) were added in slightly excess (2 equiv.), the corresponding alkynylated products **3ak–am** were obtained in moderate to good yields. Additionally, when 2 equivalents of **2a** were used with 5-nitroindole **1am**, double alkynylated **3am′** was produced selectively. Interestingly, other bromoalkynes bearing different bulky silyl protecting groups could be used as alkynylating reagents affording products **3ap–3as** in moderate to good yields. However, when smaller silyl groups were tried, the desired products could not be observed (**3at**, **3au**). Careful analysis of the crude reaction mixtures revealed that these silyl alkynes react preferentially with AgSbF_6_ yielding the corresponding trialkylsilyl fluorides. In all cases, the alkyne dimer and the chloroalkyne could also be detected. Similarly, carbon-substituted bromo-alkynes could not be used as alkynylating reagents (**3av–3az**).

**Scheme 3:**
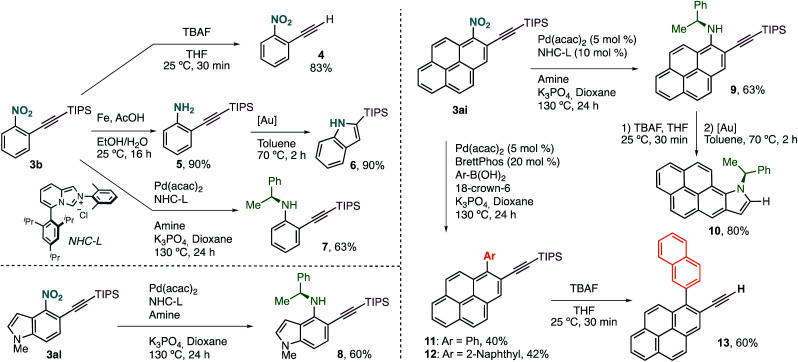
Synthetic transformations. [Au] = [(JohnPhos)Au(MeCN)]SbF_6_. See ESI† for further experimental details.

Terminal alkyne **4** was easily accessed by treatment of **3b** with a solution of TBAF in THF (Scheme 3). Selective reduction of the nitro group with Fe under acidic conditions delivered aniline **5** in excellent yield, with the triple bond untouched. We derivatized this compound further towards the synthesis of 2-(triisopropyl)silylindole (**6**) *via* Au(i)-catalysed cyclisation. This new protocol of alkynylation/reduction/cyclisation allows for the efficient synthesis of important indoles from nitroarene feedstocks. More complex anilines, such as chiral **7**, can also be prepared by denitrative Pd-catalysed C–N coupling, following the procedure reported by Wu and coworkers.^5*g*^

This protocol can also be applied to the synthesis of more complex structures such as chiral aminoalkynylated indole **8**, prepared in just two steps from simple nitroindole **1al**.

Our alkynylation protocol can also be of high interest in the synthesis and modification of π-extended systems, which are attracting attention from the synthetic community due to their interesting properties.^14^ In this context, we performed the alkynylation of 1-nitropyrene, easily accessed by electrophilic nitration, in a 1 mmol scale with an excellent result (Scheme 2). The corresponding alkynylated nitropyrene **3ai** was then subjected to the C–N coupling conditions to form chiral secondary aniline **9**, again in good yield (Scheme 3). Deprotection followed by Au(i)-catalysed cyclisation delivered chiral π-extended heteroarene **10** in excellent overall yield. Alternatively, denitrative Suzuki–Miyaura-type coupling was possible by slightly modifying the reported reaction conditions.^5*a*^ Different aromatic boronic acids were successfully coupled with **3ai** to obtain the desired biaryl systems **11** and **12**. The corresponding terminal alkynes are also accessible by TBAF deprotection. Thus, as an example of functionalized polycyclic aromatic hydrocarbon, 2-ethynyl-1-(naphthalen-2-yl)pyrene (**13**) can be easily obtained in just four steps regioselectively from pyrene by nitration, *o*-alkynylation, cross-coupling, and deprotection.

**Scheme 4:**
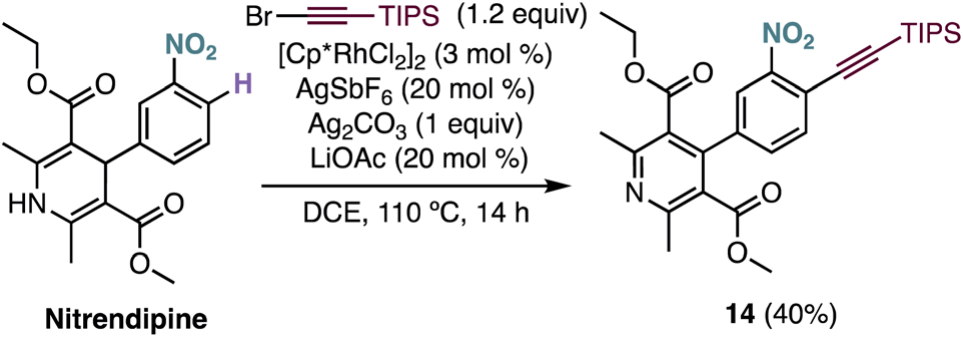
Functionalization of nitrendipine *via* Rh-catalysed C–H alkynylation.

Interestingly, under our optimised reaction conditions, the alkynylation of nitrendipine, a commercialized antihypertensive agent, leads to alkynylated product **14** in 40% yield with concomitant oxidation of the 1,4-dihydropyridine ring (Scheme 4).

**Scheme 5:**
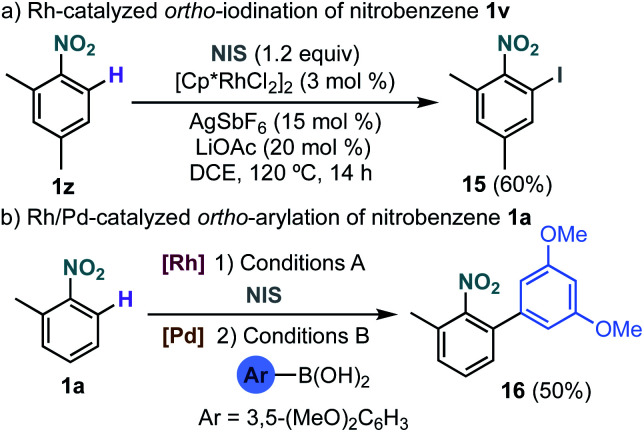
Rh-catalysed *ortho*-iodination of 2-methylnitrobenzene and one-pot *ortho*-arylation *via* sequential Rh/Pd catalysis. Conditions A: [Cp*RhCl_2_]_2_ (3 mol%), AgSbF_6_ (15 mol%), LiOAc (20 mol%), NIS (1.2 equiv.), DCE, 120 °C, 14 h. Conditions B: Pd(PPh_3_)_4_ (5 mol%), DCE, K_2_CO_3_, 60 °C, 14 h.

The site selective Rh-catalysed *ortho*-functionalization could also be extended to the iodination of nitrobenzenes using *N*-iodosuccinimide (Scheme 5a).^15^ Interestingly, an overall regioselective *ortho*-arylation of nitrobenzene could be performed by a sequential Rh-catalysed *ortho*-iodination/Pd-catalysed Suzuki–Miyaura coupling leading to biaryl **16** in 50% yield (Scheme 5b).

To understand the mechanism of this unprecedented nitro-directed Rh-catalysed *ortho*-alkynylation, we performed a series of studies encompassing experimental and computational approaches. The computational calculations were carried out using DFT at ωB97XD/6-31G(d)(H, C, O, N, F, Cl) + LANL2DZ(Rh, Ag, Br)//6-311G++(d,p)(H, C, O, N, F, Cl) + LANL2DZ(Br) + LANL2TZ(Rh, Ag) level of theory, taking into account the solvent effect (SMD = 1,2-dichloroethane).^16^

**Fig. 1:**
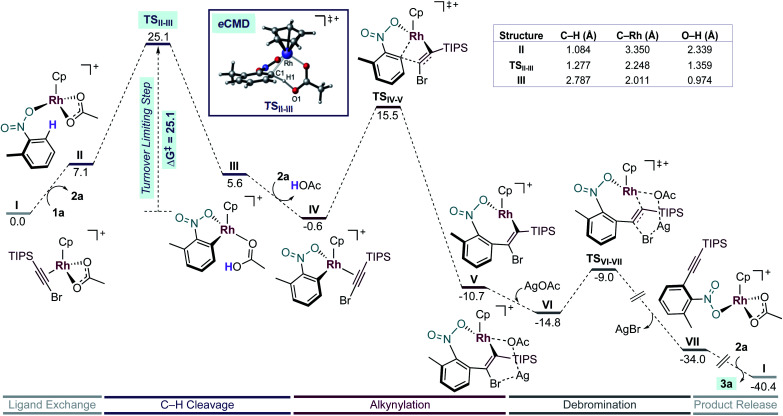
Simplification of the computed free energy profile for the Rh-catalysed alkynylation of 2-methylnitrobenzene (**1a**) and relevant structural information of stationary points **II**, **TSII-III** and **III**. Free energies in kcal mol^−1^ at 25 °C.

First, we studied computationally the complete mechanism of the Rh-catalysed alkynylation of nitrobenzene **1a**.^17^ According to our studies, after several dissociative ligand events, intermediate **II** undergoes a turnover limiting C–H bond cleavage (Δ*G*^‡^ = 25.1 kcal mol^−1^, energy span) which proceeds through a concerted six-membered cyclic transition state with intramolecular acetate-assistance (**TSII-III**) (Fig. 1, violet section).^18^ Dissociative substitution of acetic acid by bromoalkyne **2a** gives (η^2^-alkyne)Rh intermediate **IV**, which undergoes alkyne insertion through a low energy transition state (**TSIV-V**, Δ*G*^‡^ = 16.1 kcal mol^−1^) (Fig. 1, pink section). The final step consists in an almost barrierless AgOAc-assisted β-debromination (Δ*G*^‡^ = 5.8 kcal mol^−1^) to form *o*-alkynylated nitrobenzene **3a** in an overall exergonic reaction (Δ*G* = −40.4 kcal mol^−1^) (Fig. 1, grey sections). The C–H cleavage transition state (**TSII-III**) features a slightly elongated C1–H1 (1.277 Å) bond, whereas C1–Rh (2.248 Å) and H1–O1 (1.359 Å) bond distances are considerably contracted compared to the previous intermediate **II** (Table in Fig. 1). The NBO analysis of **TSII-III** reveals two main electronic interactions not present in intermediate **II** related to the C–H bond cleavage event.^13^

First, a lone pair on O1 delocalized over H1 (η_O1_, 86.3% O1 and 7.2% H1), which highlights the role of the acetate in the abstraction of H1 during **TSII-III** (Fig. S6†). Second, a NLMO associated to σ-C1–H1 bond which is delocalized over Rh (Ω_C1–H1_, 65.4% C, 20.6% H and 8.6% Rh) indicating the formation of the C1–Rh bond during **TSII-III** (Fig. S7†). All in all, the indicated bond distances and the NBO analysis suggest that **TSII-III** is considerably asynchronous with a greater extent of C1–Rh and O1–H1 bonds forming than C1–H1 bond cleavage.

**Scheme 6:**
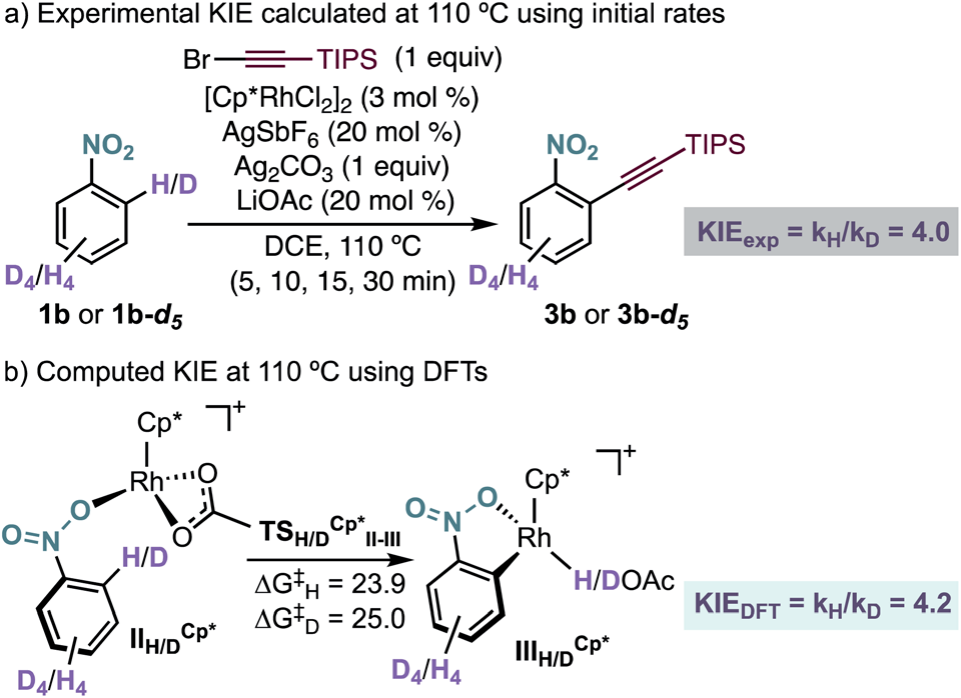
(a) Experimental and (b) computational KIE for the Rh-catalysed *ortho*-alkynylation of nitrobenzenes **1b** and **1b-d5**. Free energies in kcal mol^−1^ at 110 °C.

Experimentally, we found a significant kinetic isotope effect (KIE = 4.0) (Scheme 6a), which supports the computational finding that the C–H bond cleavage corresponds to the turnover determining step of the catalytic cycle. Moreover, the computed KIE at 110 °C using Cp*Rh was 4.2 and accurately reproduces the experimental results (Scheme 6b).^19^ Interestingly, the computed KIE using a model system bearing Cp ligand was significantly smaller (KIE = 2.7).

**Fig. 2:**
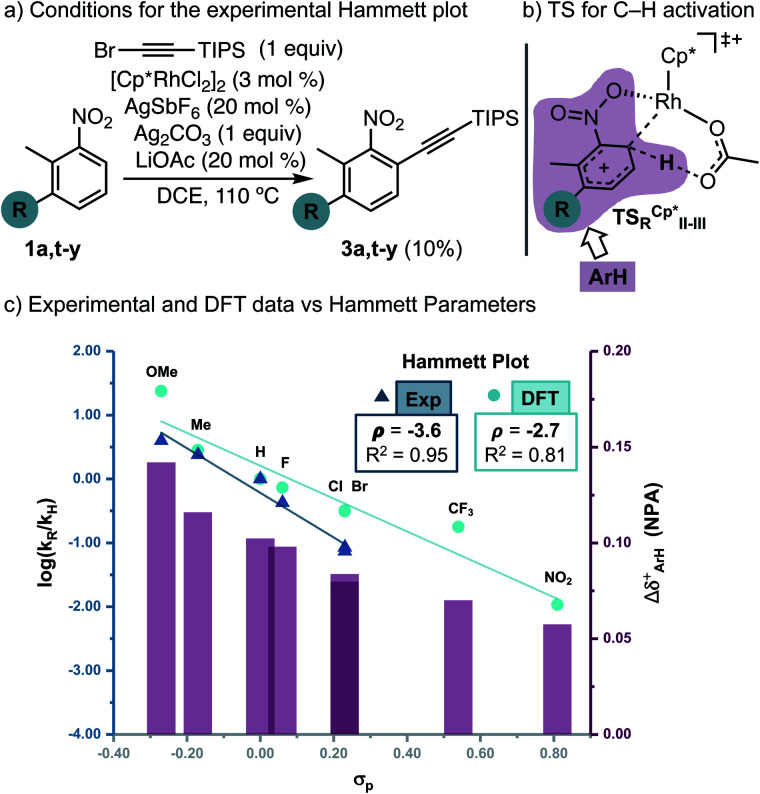
Experimental and computational Hammett correlations. The charge accumulation (Δ*δ*^+^, represented as pink bars) has been calculated as the sum of the NPA on all the atoms of the nitrobenzene fragment on the TS_R_^Cp*^_II-III_ for all the different substituents specified in the graph (pink area in Fig. 2b).

Next, to understand whether or not a positive charge could build-up *ortho* to the nitro group, we performed initial rates measurements of *meta*-substituted 2-methylnitrobenzenes **1a,t-y** (Fig. 2a). A Hammett correlation was found (*R*^2^ = 0.95 using *σ*_p_) with a negative *ρ* value (*ρ* = −3.6), suggesting a decrease of electron density at the aryl ring in the C–H activation step (Fig. 2c, dark blue data, triangles). A computational Hammett correlation was also found for a series of *meta*-substituted nitrobenzenes and the *σ*_p_ parameter (*ρ* = −2.7, *R*^2^ = 0.81) (Fig. 2c, clear blue data, circles).^20,21^ The computed negative *ρ* value is in agreement with the experimental results. To understand the charge distribution in the transition state of the turnover-limiting step, we assessed the charge accumulation (Δ*δ*^+^) over the *meta*-substituted nitrobenzene fragment in the concerted transition states by NBO analysis (Fig. 2b and c, pink data). There is a slight positive charge built up on the substituted nitrobenzenes (Δ*δ*^+^ = 0.06–0.14), which agrees with the trends observed by Hammett analysis. In fact, a straight regression line was obtained by plotting Δ*δ*^+^*vs. σ*_p_ parameters (*R*^2^ = 0.89) (Fig. S13†). Both the Hammett and NBO analysis suggest that the Rh-catalysed C–H activation corresponds to an electrophilic concerted metalation deprotonation process,^12^ in which both an electrophilic metal and a basic ligand cooperate in the cleavage of the C–H bond and subsequent rhodacycle formation.

Despite our previous observation of this mechanism for the alkynylation of esters, ethers and ketones,^12*a*^ the engagement of nitrobenzenes in acetate-assisted internal electrophilic substitution is rather remarkable, considering the strong electron-withdrawing character and reduced coordination ability of the nitro group. An inspection of the structural features in intermediate **II** (Table 2) shows an elongated O–Rh bond for the nitro (2.253 Å) when compared to the other directing groups (2.178–2.209 Å), showcasing the limited coordination offered by the –NO_2_ group (Table 2). Moreover, **TSII-III** exhibits a shorter C1–H1 bond and longer C1–Rh and O–Rh distances for nitrobenzene.

**Table 2:** Representative calculated bond distances

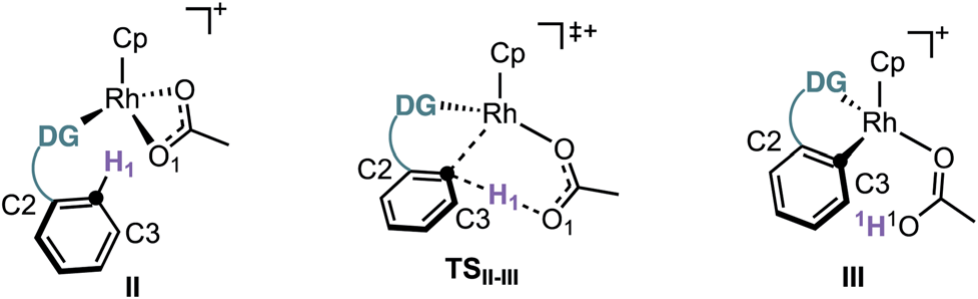				
Structure	Directing group	C1–H1 (Å)	C1–Rh (Å)	O2(DG)–Rh (Å)
**II**	NO_2_	1.085	3.378	2.253
	CO_2_Me	1.084	3.684	2.187
	CH_2_OMe	1.087	3.510	2.209
	C(O)Me	1.086	3.340	2.178
**TSII-III**	NO_2_	1.272	2.251	2.212
	CO_2_Me	1.304	2.228	2.191
	CH_2_OMe	1.306	2.186	2.191
	C(O)Me	1.308	2.214	2.169
**III**	NO_2_	2.923	2.008	2.152
	CO_2_Me	2.273	2.023	2.165
	CH_2_OMe	2.066	1.022	2.170
	C(O)Me	2.798	2.012	2.132

**Scheme 7:**
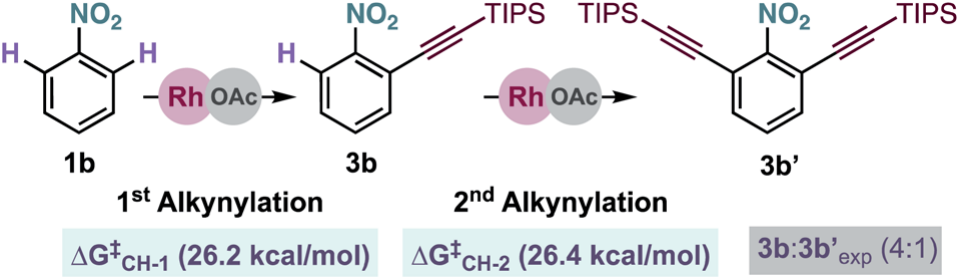
Energy comparison for the Rh-catalysed mono and di-alkynylation of nitrobenzene **1b**. Free energies at 25 °C.

We also examined theoretically the formation of dialkynylated nitrobenzene **3b′** (Scheme 7). Structurally similar transition states were found both for the C–H cleavage of **1b** and the subsequent C–H activation step of mono-alkynylated nitrobenzene **3b**.^13^ The small differences between the activation barriers calculated for the first (Δ*G*^‡^_CH-1_ = 26.2 kcal mol^−1^) and the second (Δ*G*^‡^_CH-2_ = 26.4 kcal mol^−1^) *ortho*-C–H cleavages of nitrobenzene **1j** explains the moderate mono/di-alkynylation selectivity observed experimentally under the optimal conditions 
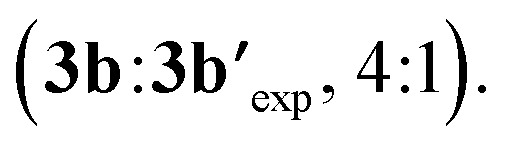


Regarding nitro-heteroarenes, we found that the activation energy for the C–H metalation step is energetically more demanding for thiophene (**1aj**) and 3-nitropyridine (**1ao**) than for nitroarenes which make the overall *ortho*-alkynylation more challenging.^13^

**Fig. 3:**
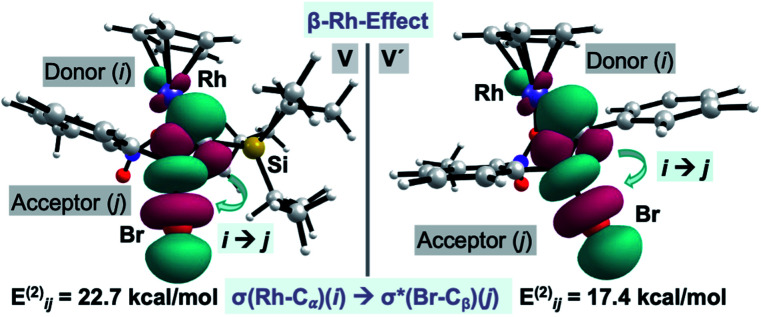
Plot of the donor–acceptor NBO interaction for the σ–σ-conjugation across alkenyl-Rh for **V** and **V′**. Cutoff: 0.05.

Surprisingly, our calculations show that the alkyne insertion/Ag-assisted-β-debromination sequence is energetically feasible for the experimentally unsuccessful alkyne counterparts **2c**, **2d**, and **2f**,^13^ which suggests that alkyne **2a** has the correct steric and electronic balance to prevent scenarios in which the alkyne is unproductively consumed leading to secondary products prior to its insertion into the C–Rh bond. Particularly intrigued by the low activation barrier for the β-debromination for non-Si containing bromo phenylacetylene (**2f**) (Δ*G*^‡^ = 4.3 kcal mol^−1^), we examined the hyperconjugative interactions on the previous intermediates to the bromo elimination **V** and **V′** for alkynes **2a** and **2f**, respectively (Fig. 3).^22^ For **V**, the NBO analysis revealed a strong interaction between σ(Rh-C_α_) and σ*(Br-C_β_) orbitals (*E*_*ij*_^(2)^ = 22.7 kcal mol^−1^) (Fig. 3, left), while no interaction was found between the σ(Si-C_α_) and σ*(Br-C_β_) orbitals. The same scenario was encountered for **V′**, in which the σ(Rh-C_α_) and σ*(Br-C_β_) orbitals also interact significantly (*E*_*ij*_^(2)^ = 17.4 kcal mol^−1^) (Fig. 3, right). These results indicate that the Ag-assisted debromination is facilitated by the σ–σ-conjugation arising from the so-called “β-Rh effect”,^17*a*,*b*^ rather than by β-Si effect.^23^

## Conclusions

We have developed the first general *o*-alkynylation of abundant nitroarenes, which proceeds in a rather straightforward manner under catalytic conditions. Despite the strongly electron-withdrawing nature of the nitro group, our experimental and computational results demonstrate that the rhodium-catalysed *o*-alkynylation of nitroarenes takes place by an electrophilic concerted metalation/deprotonation pathway. The resulting *o*-alkynyl nitroarenes are precursors of indoles by reduction to the corresponding anilines and cyclization^24^ and have also been used as synthons for the preparation of other valuable products.^25^ In this context, the nitro group can be viewed as a “super pseudo-halogen”, that participates in a manifold of cross-coupling reactions,^3–5^ while allowing also the introduction of highly versatile alkynyl groups at the *ortho* position under catalytic conditions.

## Data availability

All experimental and computational data associated with the article are incorporated into the ESI.†

## Author contributions

Conceptualization: A. M. E. and E. T.; formal analysis: C. G.-M.; funding acquisition: A. M. E.; investigation: E. T., M. M.-M., C. G.-M., and J. G. M; methodology: E. T., and C. G.-M; project administration: A. M. E.; supervision: A. M. E.; visualization: E. T., M. M.-M., C. G.-M., and J. G. M.; writing – original draft: E. T., C. G.-M., and A. M. E.; writing – review & editing: E. T., M. M.-M., C. G.-M., J. G. M., and A. M. E.

## Conflicts of interest

There are no conflicts to declare.

## Supplementary Material

SC-012-D1SC04527J-s001
