# Supplementary material for: Rhodium-catalysed ortho-alkynylation of nitroarenes
Source: Chem Sci. 2021 Oct 11;12(44):14731–9. doi: 10.1039/d1sc04527j (PMC8597868; doi:10.1039/d1sc04527j)
Supplement: SC-012-D1SC04527J-s001 [file SC-012-D1SC04527J-s001.pdf]

Electronic Supplementary Material (ESI) for Chemical Science.
This journal is © The Royal Society of Chemistry 2021

Supporting Information

Rhodium-Catalysed *ortho*-Alkynylation of Nitroarenes

Eric Tan, Marc Montesinos-Magraner, Cristina García-Morales, Joan Guillem Mayans and Antonio M. Echavarren*

Institute of Chemical Research of Catalonia (ICIQ), Barcelona Institute of Science and Technology, Av. Països Catalans 16, 43007 Tarragona (Spain).

Departament de Química Orgànica i Analítica, Universitat Rovira i Virgili, C/ Marcel·lí Domingo s/n, 43007 Tarragona (Spain).

Email: aechavarren@iciq.es

Table of Contents

1. GENERAL METHODS 4

2. SCOPE OF THE RH-CATALYZED *ORTHO*-C–H ALKYNYLATION OF NITROBENZENES 5

UNSUCCESSFUL NITRO-HETEROARENES 19

3. SYNTHETIC TRANSFORMATIONS 20

4. ALKYNYLATION OF NITRENDIPINE 24

5. RHODIUM-CATALYZED C–H IODINATION OF NITROBENZENE 1Z 25

6. EXPERIMENTAL MECHANISTIC INVESTIGATIONS 26

KINETIC ISOTOPE EFFECT 26

HAMMETT PLOT 28

7. DFT CALCULATIONS 30

COMPUTATIONAL DETAILS 30

PLAUSIBLE RESTING STATES 31

DFT MECHANISM FOR THE *ORTHO*-ALKYNYLATION OF 1A 32

ALTERNATIVE TRANSITION STATES FOR THE C–H ACTIVATION 33

STRUCTURAL ANALYSIS OF RELEVANT STRUCTURES 34

NBO ANALYSIS – NLMOS 35

FORMATION OF 2,6-DIALKYLYNATED NITROBENZENES 36

KINETIC ISOTOPE EFFECT 37

COMPUTATIONAL CORRELATIONS WITH HAMMETT PARAMETERS 39

DFT STUDIES ON SCOPE LIMITATIONS 45

B-RHODIUM EFFECT 54

CARTESIAN COORDINATES 56

8. NMR SPECTRA 136

9. X-RAY CRYSTALLOGRAPHIC DATA 207

1. General Methods

Reactions were set-up in an argon-filled glovebox, taken out, and stirred at the indicated temperature. Analytical thin layer chromatography was carried out using TLC-aluminum sheets with 0.2 mm of silica gel (Merck GF234) using UV light as the visualizing agent and an acidic solution of vanillin in ethanol as the developing agent. Chromatographic purifications were carried out using automated flash chromatographer CombiFlash Companion. Organic solutions were concentrated under reduced pressure on a Büchi rotary evaporator. All reagents were used as purchased with no further purification, unless otherwise stated. Alkyne **2a**,[1a] **2b**,[1b] **2c**,[1c] **2d**,[1d] **2e**[1e] and **2f**[1d], and indoles **1ag**[1f] and **1ah**[1g] were prepared according to previous reports. Their spectral data are consistent with the previously reported.

NMR spectra were recorded at 298 K (unless otherwise stated) on a Bruker Avance 300, Bruker Avance 400 Ultrashield and Bruker Avance 500 Ultrashield apparatuses. The signals are given as d / ppm (multiplicity, coupling constant (Hertz), number of protons) downfield from tetramethylsilane, with calibration on the residual protio-solvent used (dH = 7.27 ppm and dC = 77.00 ppm for $CDCl_3$, dH = 5.32 ppm and dC = 53.84 ppm for CD_2Cl_2). Mass spectra were recorded on a Waters Micromass LCT Premier (ESI), Waters Micromass GCT (EI, CI) and Bruker Daltonics Autoflex (MALDI) spectrometers. Melting points were determined using a Büchi melting point apparatus.

2. Scope of the Rh-Catalyzed *ortho*-C–H Alkynylation of Nitrobenzenes

General procedure for the Rh-catalyzed *ortho*-C–H alkynylation of nitrobenzenes

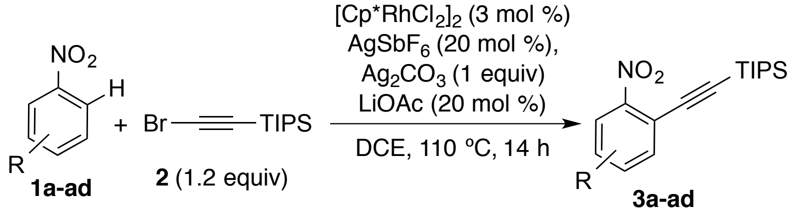

$[Cp^*RhCl_2]_2$ (3 mol%), Ag_2CO_3 (1 equiv), LiOAc (20 mol%), $AgSbF_6$ (20 mol%) were weighted in a vial inside a glovebox and dichloroethane (0.15M) is added. Corresponding nitrobenzene **1a-ah** (0.2 mmol) and 1-bromo-2-(triisopropylsilyl)acetylene (**2a**) (1.2 equiv) are then added and the vial is sealed. The reaction mixture is stirred at 110 °C for 14 h outside the glovebox. After cooling to room temperature, the reaction mixture is filtrated through celite and purified by column chromatography, with a gradient from cyclohexane 100% to 1/1 cyclohexane/ethyl acetate to yield corresponding products **3a-an**.

Triisopropyl((3-methyl-2-nitrophenyl)ethynyl)silane (3a)

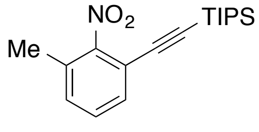

Compound **3a** was synthesized according to the general procedure and it was obtained as a colorless liquid in 95% yield.

^{1}H NMR (300 MHz, $CDCl_3$) δ 7.43 (dt, J = 7.7, 1.0 Hz, 1H), 7.32 (t, J = 7.7 Hz, 1H), 7.24 (ddd, J = 7.7, 1.5, 1.0 Hz, 1H), 2.34 (s, 3H), 1.13 (s, 21H). **^{13}C NMR** (75 MHz, $CDCl_3$) δ 153.3, 131.1, 131.0, 129.7 (2C), 116.5, 99.5, 98.6, 18.5, 17.3, 11.2. **HRMS** (APCI+) *m/z* calc. for $C_{18}H_{28}NO_2Si$ $[M+H]^+$: 318.1884. Found: 318.1884.

Triisopropyl((2-nitrophenyl)ethynyl)silane (3b)

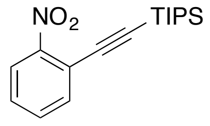

Compound **3b** was synthesized according to the general procedure using 2 equiv of 1-bromo-2-(triisopropylsilyl)acetylene (**2a**) and it was obtained as a white solid in 75% yield.

Mp 55 °C. **^{1}H NMR** (300 MHz, $CDCl_3$) δ 8.03 (dd, J = 8.2, 0.9 Hz, 1H), 7.69 (dd, J = 7.7, 1.3 Hz, 1H), 7.57 (td, J = 7.7, 1.3 Hz, 1H), 7.49 – 7.42 (m, 1H), 1.17 (s, 21H). **^{13}C NMR** (75 MHz, $CDCl_3$) δ 150.0, 135.4, 132.6, 128.6, 124.4, 118.7, 101.1, 100.8, 18.6, 11.2. **HRMS** (ESI+) *m/z* calc. for $C_{17}H_{25}NNaO_2Si$ $[M+Na]^+$: 326.1547. Found: 326.1536.

((2-Nitro-1,3-phenylene)bis(ethyne-2,1-diyl))bis(triisopropylsilane) (3b')

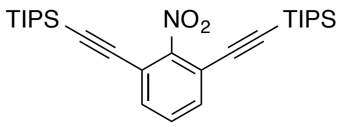

Compound **3b'** was synthesized according to the general procedure using 2 equiv of 1-bromo-2-(triisopropylsilyl)acetylene (**2a**) and it was obtained as a purple solid in 15% yield.

Mp 75 °C. **^{1}H NMR** (300 MHz, $CDCl_3$) δ 7.52 (d, J = 8.2 Hz, 2H), 7.37 (dd, J = 8.2, 7.1 Hz, 1H), 1.12 (s, 42H). **^{13}C NMR** (75 MHz, $CDCl_3$) δ 154.8, 132.9, 129.6, 116.8, 99.9, 98.5, 18.5, 11.1. **HRMS** (ESI+) *m/z* calc. for $C_{28}H_{45}NNaO_2Si_2$ $[M+Na]^+$: 506.2881. Found: 506.2861.

((3-Ethyl-2-nitrophenyl)ethynyl)triisopropylsilane (3c)

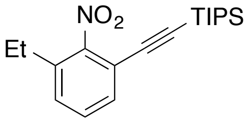

Compound **3c** was synthesized according to the general procedure and it was obtained as a yellow liquid in 91% yield.

^{1}H NMR (300 MHz, $CDCl_3$) δ 7.41 (dd, *J* = 7.6, 1.7 Hz, 1H), 7.34 (t, *J* = 7.6 Hz, 1H), 7.27 (dd, *J* = 7.6, 1.7 Hz, 1H), 2.61 (q, *J* = 7.6 Hz, 2H), 1.23 (t, *J* = 7.6 Hz, 3H), 1.11 (s, 21H). **^{13}C NMR** (75 MHz, $CDCl_3$) δ 153.0, 135.4, 130.9, 129.8, 129.6, 116.3, 99.4, 98.4, 24.4, 18.5, 14.8, 11.1. **HRMS** (APCI+) *m/z* calc. for $C_{19}H_{30}NO_2Si$ $[M+H]^+$: 332.2040. Found: 332.2027.

Triisopropyl((2-nitro-[1,1'-biphenyl]-3-yl)ethynyl)silane (3d)

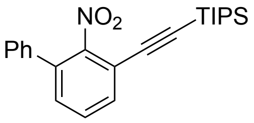

Compound **3d** was synthesized according to the general procedure and it was obtained as a yellow solid in 65% yield.

Mp 99 °C. **^{1}H NMR** (500 MHz, $CDCl_3$) δ 7.60 (dd, *J* = 7.7, 1.4 Hz, 1H), 7.49 (t, *J* = 7.8 Hz, 1H), 7.44 – 7.36 (m, 6H), 1.18 – 1.10 (m, 21H). **^{13}C NMR** (126 MHz, $CDCl_3$) δ 152.5, 136.0, 134.5, 132.3, 130.9, 130.0, 129.0, 128.9, 128.1, 117.0, 99.4, 99.3, 18.7, 11.3. **HRMS** (ESI+): m/z calc. for $C_{23}H_{29}NNaO_2Si$ $[M+Na]^+$: 402.1860. Found: 402.1848.

Triisopropyl((3-methoxy-2-nitrophenyl)ethynyl)silane (3e)

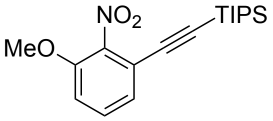

Compound **3e** was synthesized according to the general procedure and it was obtained as a colorless oil in 87% yield.

^{1}H NMR (500 MHz, $CDCl_3$) δ 7.34 (dd, J = 8.5, 7.8 Hz, 1H), 7.12 (dd, J = 7.8, 1.1 Hz, 1H), 6.99 (dd, J = 8.5, 1.0 Hz, 1H), 3.89 (s, 3H), 1.10 (d, J = 3.2 Hz, 21H). **^{13}C NMR** (126 MHz, $CDCl_3$) δ 150.7, 143.6, 130.7, 124.9, 117.7, 112.9, 99.4, 99.0, 56.6, 18.7, 11.3. HRMS (ESI+): m/z calc. for C18H27NNaO3Si [M+Na]+: 356.1652. Found: 356.1653.

2-Nitro-3-((triisopropylsilyl)ethynyl)benzaldehyde (3f)

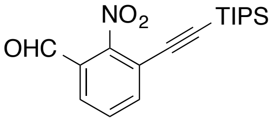

Compound **3f** was synthesized according to the general procedure and it was obtained as an orange solid in 74% yield.

Mp 50 °C. **^{1}H NMR** (300 MHz, $CDCl_3$) δ 10.49 (s, 1H), 7.81 (m, 2H), 7.60 (t, *J* = 8.0 Hz, 1H), 1.13 (m, 21H). **^{13}C NMR** (75 MHz, $CDCl_3$) δ 188.3, 148.1, 137.7, 132.8, 132.1, 125.7, 123.4, 101.7, 100.4, 18.6, 11.2. **HRMS** (ESI+) *m/z* calc. for $C_{18}H_{25}NNaO_3Si$ $[M+Na]^+$: 354.1496. Found: 354.1496.

((3-Fluoro-2-nitrophenyl)ethynyl)triisopropylsilane (3g)

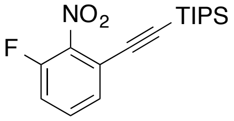

Compound **3g** was synthesized according to the general procedure and it was obtained as a colorless liquid in 54% yield.

^{1}H NMR (300 MHz, $CDCl_3$) δ 7.49 – 7.36 (m, 2H), 7.22 (ddd, *J* = 9.6, 7.9, 1.8 Hz, 1H), 1.13 (s, 21H). **^{13}C NMR** (101 MHz, $CDCl_3$) δ 153.5 (d, *J* = 258.3 Hz), 131.6 (d, *J* = 8.6 Hz), 129.1 (d, *J* = 3.7 Hz), 119.0, 117.1, 116.9, 101.4, 98.1 (d, *J* = 3.7 Hz), 18.48, 11.12. **^{19}F{^{1}H} NMR** (376 MHz, $CDCl_3$) δ -122.44. **HRMS** (APCI+) *m/z* calc. for $C_{17}H_{25}FNO_2Si$ $[M+H]^+$: 322.1633. Found: 322.1632.

((3-Bromo-2-nitrophenyl)ethynyl)triisopropylsilane (3h)

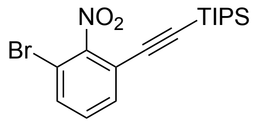

Compound **3h** was synthesized according to the general procedure and it was obtained as a colorless oil in 76% yield.

^{1}H NMR (500 MHz, $CDCl_3$) δ 7.62 (dd, *J* = 8.1, 1.2 Hz, 1H), 7.54 (dd, *J* = 7.8, 1.2 Hz, 1H), 7.31 (t, *J* = 8.0 Hz, 1H), 1.17 – 1.08 (m, 21H). **^{13}C NMR** (126 MHz, $CDCl_3$) δ 153.5, 133.4, 132.3, 130.7, 118.5, 112.8, 101.1, 98.2, 18.6, 11.3. **HRMS** (ESI+): m/z calc. for $C_{17}H_{24}BrNNaO_2Si$ $[M+Na]^+$: 404.0652. Found: 404.0663.

((3-Iodo-2-nitrophenyl)ethynyl)triisopropylsilane (3i)

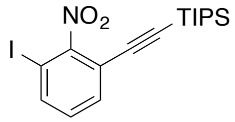

Compound **3i** was synthesized according to the general procedure and it was obtained as a colorless liquid in 71% yield.

^{1}H NMR (300 MHz, $CDCl_3$) δ 7.81 (dd, *J* = 7.9, 1.2 Hz, 1H), 7.53 (dd, *J* = 7.9, 1.2 Hz, 1H), 7.12 (t, *J* = 7.9 Hz, 1H), 1.10 (m, 21H). **^{13}C NMR** (75 MHz, $CDCl_3$) δ 157.2, 139.6, 132.6, 130.5, 117.3, 100.7, 98.3, 84.4, 18.5, 11.1. **HRMS** (APCI+) *m/z* calc. for $C_{17}H_{25}INO_2Si$ $[M+H]^+$: 430.0694. Found: 430.0702.

((2,2'-Dinitro-[1,1'-biphenyl]-3-yl)ethynyl)triisopropylsilane (3j)

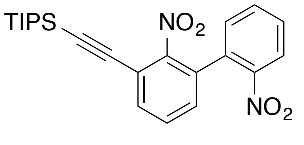

Compound **3j** was synthesized according to the general procedure using 2 equiv of 1-bromo-2-(triisopropylsilyl)acetylene (**2a**) and it was obtained as a red solid in 40% yield.

Mp 100 °C. **^{1}H NMR** (300 MHz, $CDCl_3$) δ 8.14 (dd, *J* = 7.9, 1.7 Hz, 1H), 7.69 – 7.57 (m, 3H), 7.49 (t, *J* = 7.9 Hz, 1H), 7.33 (dd, *J* = 7.2, 1.9 Hz, 1H), 7.26 (dd, *J* = 7.9, 1.4 Hz, 1H), 1.11 (m, 21H). **^{13}C NMR** (75 MHz, $CDCl_3$) δ 150.9, 148.0, 133.4, 133.1, 131.6, 131.5, 130.7, 130.0, 130.0, 129.6, 125.0, 117.3, 100.1, 99.0, 18.5, 11.1. **HRMS** (APCI+) *m/z* calc. for $C_{23}H_{29}N_2O_4Si$ $[M+H]^+$: 425.1891. Found: 425.1881.

2,2'-Dinitro-[1,1'-biphenyl]-3,3'-diyl)bis(ethyne-2,1-diylbis(triisopropylsilane) (3j')

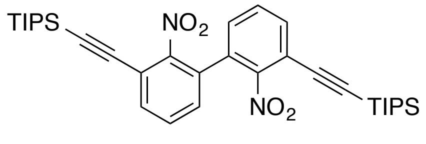

Compound **3j'** was synthesized according to the general procedure using 2 equiv of 1-Bromo-2-(triisopropylsilyl)acetylene (**2a**) and it was obtained as a yellow solid in 15% yield.

Mp 150 °C. **^{1}H NMR** (300 MHz, $CDCl_3$) δ 7.66 (dd, *J* = 7.8, 1.4 Hz, 2H), 7.46 (t, *J* = 7.8 Hz, 2H), 7.30 (dd, *J* = 7.8, 1.4 Hz, 2H), 1.13 (d, *J* = 2.7 Hz, 42H). **^{13}C NMR** (75 MHz, $CDCl_3$) δ 151.9, 134.1, 130.0, 129.9, 128.5, 117.6, 100.6, 98.7, 18.5, 11.1. **HRMS** (APCI+) *m/z* calc. for $C_{34}H_{49}N_2O_4Si_2$ $[M+H]^+$: 605.3225. Found: 605.3244.

Triisopropyl((4-methyl-2-nitrophenyl)ethynyl)silane (3k)

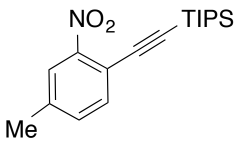

Compound **3k** was synthesized according to the general procedure and it was obtained as a red solid in 85% yield.

Mp 55 °C. **^{1}H NMR** (300 MHz, $CDCl_3$) δ 7.82 (dd, *J* = 1.8, 0.9 Hz, 1H), 7.55 (d, *J* = 7.9 Hz, 1H), 7.35 (ddd, *J* = 7.9, 1.8, 0.9 Hz, 1H), 2.44 (s, 3H), 1.16 (s, 21H). **^{13}C NMR** (75 MHz, $CDCl_3$) δ 149.8, 139.6, 135.1, 133.3, 124.7, 115.7, 101.2, 99.4, 21.2, 18.5, 11.2. **HRMS** (APCI+) *m/z* calc. for $C_{18}H_{28}NO_2Si$ $[M+H]^+$: 318.1884. Found: 318.1882.

***N*,*N*-Dimethyl-3-nitro-4-((triisopropylsilyl)ethynyl)aniline (3l)**

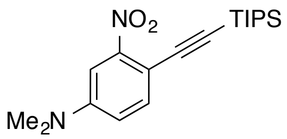

Compound **3l** was synthesized according to the general procedure and it was obtained as a red solid in 65% yield.

Mp 105 °C. **^{1}H NMR** (300 MHz, $CDCl_3$) δ 7.46 (d, *J* = 8.8 Hz, 1H), 7.21 (d, *J* = 2.7 Hz, 1H), 6.78 (dd, *J* = 8.8, 2.7 Hz, 1H), 3.04 (s, 6H), 1.15 (s, 21H). **^{13}C NMR** (75 MHz, $CDCl_3$) δ 151.2, 149.7, 135.9, 115.3, 106.5, 104.8, 102.3, 95.7, 40.1, 18.6, 11.3. **HRMS** (ESI+) *m/z* calc. for $C_{19}H_{31}N_2O_2Si$ $[M+H]^+$: 347.2149. Found: 347.2146.

Triisopropyl((2-nitro-3-vinylphenyl)ethynyl)silane (3m)

Compound **3m** was synthesized according to the general procedure and it was obtained as a colorless oil in 59% yield.

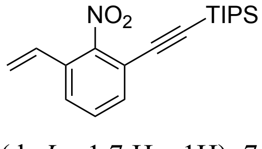

^{1}H NMR (500 MHz, $CDCl_3$) δ 8.03 (d, *J* = 1.7 Hz, 1H), 7.60 (d, *J* = 8.1 Hz, 1H), 7.55 (dd, *J* = 8.1, 1.7 Hz, 1H), 6.72 (dd, *J* = 17.6, 10.9 Hz, 1H), 5.88 (d, *J* = 17.6 Hz, 1H), 5.46 (d, *J* = 10.9 Hz, 1H), 1.18 – 1.11 (m, 21H). **^{13}C NMR** (126 MHz, $CDCl_3$) δ 150.5, 138.6, 135.7, 134.4, 130.0, 122.0, 117.9, 117.6, 101.5, 101.4, 18.8, 11.4. **HRMS** (ESI+): m/z calc. for $C_{19}H_{27}NNaO_2Si$ $[M+Na]^+$: 352.1703. Found: 352.1706.

((2-Fluoro-6-nitrophenyl)ethynyl)triisopropylsilane (3n)

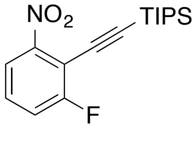

Compound **3n** was synthesized according to the general procedure using 2 equiv of 1-bromo-2-(triisopropylsilyl)acetylene (**2a**) and it was obtained as a red solid in 60% yield.

Mp 70 °C. **^{1}H NMR** (300 MHz, $CDCl_3$) δ 7.83 (dt, *J* = 7.7, 1.4 Hz, 1H), 7.46 – 7.32 (m, 2H), 1.15 (m, 21H). **^{13}C NMR** (101 MHz, $CDCl_3$) δ 163.9 (d, *J* = 255.7 Hz), 150.8, 128.8 (d, *J* = 8.8 Hz), 120.0 (d, *J* = 4.9 Hz), 119.8 (d, *J* = 20.5 Hz), 108.6 (d, *J* = 20.5 Hz), 107.8 (d, *J* = 4.9 Hz),

93.2, 18.5, 11.2. **^{19}F NMR** (376 MHz, $CDCl_3$) δ -104.62 (dd, J = 8.0, 5.5 Hz). **HRMS** (APCI+) m/z calc. for $C_{17}H_{25}FNO_2Si$ $[M+H]^+$: 322.1633. Found: 322.1644.

((4-Fluoro-2-nitro-1,3-phenylene)bis(ethyne-2,1-diyl))bis(triisopropylsilane) (3n')

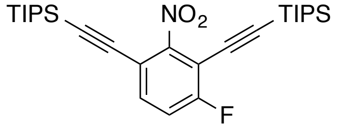

Compound **3n'** was synthesized according to the general procedure using 2 equiv of 1-bromo-2-(triisopropylsilyl)acetylene (**2a**) and it was obtained as a white solid in 30% yield.

Mp 70 °C. **^{1}H NMR** (300 MHz, $CDCl_3$) δ 7.48 (dd, J = 8.5, 5.2 Hz, 1H), 7.16 (t, J = 8.5 Hz, 1H), 1.10 (m, 42H). **^{13}C NMR** (101 MHz, $CDCl_3$) δ 162.1 (d, J = 259.4 Hz), 155.3, 133.7 (d, J = 8.6 Hz), 117.3 (d, J = 22.0 Hz), 112.9 (d, J = 4.4 Hz), 107.0 (d, J = 22.0 Hz), 106.2 (d, J = 3.6 Hz), 99.6 (d, J = 1.8 Hz), 97.6 (d, J = 1.6 Hz), 91.6, 18.5, 18.4, 11.1, 11.1. **^{19}F NMR** (376 MHz, $CDCl_3$) δ -103.51 (dd, J = 8.0, 5.2 Hz). **HRMS** (APCI+) m/z calc. for $C_{28}H_{45}FNO_2Si_2$ $[M+H]^+$: 502.2967. Found: 502.2969.

Triisopropyl((2-methoxy-6-nitrophenyl)ethynyl)silane (3o)

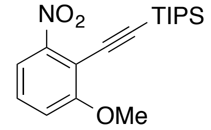

Compound **3o** was synthesized according to the general procedure using 2 equiv of 1-bromo-2-(triisopropylsilyl)acetylene (**2a**) and it was obtained as a brown solid in 30% yield.

Mp 75 °C. **^{1}H NMR** (300 MHz, $CDCl_3$) δ 7.52 (dd, J = 8.3, 1.0 Hz, 1H), 7.35 (t, J = 8.3 Hz, 1H), 7.09 (dd, J = 8.3, 1.0 Hz, 1H), 3.92 (s, 3H), 1.15 (s, 21H). **^{13}C NMR** (75 MHz, $CDCl_3$) δ 162.1, 152.2, 128.7, 115.9, 114.6, 108.4, 105.9, 96.1, 56.6, 18.6, 11.3. **HRMS** (APCI+) m/z calc. for $C_{18}H_{28}NO_3Si$ $[M+H]^+$: 334.1833. Found: 334.1828.

((4-Methoxy-2-nitro-1,3-phenylene)bis(ethyne-2,1-diyl))bis(triisopropylsilane) (3o').

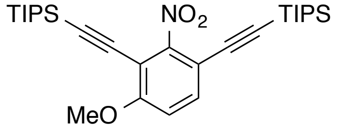

Compound **3o'** was synthesized according to the general procedure using 2 equiv of 1-bromo-2-(triisopropylsilyl)acetylene (**2a**) and it was obtained as a brown solid in 15% yield.

Mp 85 °C. **^{1}H NMR** (300 MHz, $CDCl_3$) δ 7.46 (d, J = 8.8 Hz, 1H), 6.90 (d, J = 8.8 Hz, 1H), 3.93 (s, 3H), 1.12 (s, 21H), 1.11 (s, 21H). **^{13}C NMR** (75 MHz, $CDCl_3$) δ 160.7, 156.0, 133.6, 111.9, 108.4, 106.8, 104.1, 98.6, 97.0, 94.5, 58.5, 18.5, 11.1. The signals at 18.5 and 11.1 ppm correspond to the 18 C for both TIPS groups. **HRMS** (APCI+) m/z calc. for $C_{29}H_{48}NO_3Si_2$ $[M+H]^+$: 514.3167. Found: 514.3169.

((5-Fluoro-2-nitrophenyl)ethynyl)triisopropylsilane (3p)

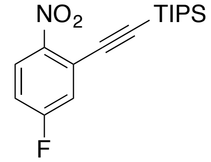

Compound **3p** was synthesized according to the general procedure using 2 equiv of 1-bromo-2-(triisopropylsilyl)acetylene (**2a**) and it was obtained as a red liquid in 56% yield.

^{1}H NMR (300 MHz, $CDCl_3$) δ 8.09 (dd, J = 9.1, 5.1 Hz, 1H), 7.33 (dd, J = 8.5, 2.8 Hz, 1H), 7.13 (ddd, J = 9.1, 7.2, 2.8 Hz, 1H), 1.14 (m, 21H). **^{13}C NMR** (101 MHz, $CDCl_3$) δ 164.2 (d, J = 257.2

Hz), 146.2, 127.2 (d, J = 10.2 Hz), 122.0 (d, J = 24.5 Hz), 121.5 (d, J = 11.0 Hz), 116.0 (d, J = 23.4 Hz), 103.0, 100.1 (d, J = 2.1 Hz), 18.6, 11.2. **^{19}F NMR** (376 MHz, $CDCl_3$) δ -104.60 (td, J = 7.9, 5.1 Hz). **HRMS** (APCI+) *m/z* calc. for $C_{17}H_{25}FNO_2Si$ $[M+H]^+$: 322.1633. Found: 322.1641.

((5-Fluoro-2-nitro-1,3-phenylene)bis(ethyne-2,1-diyl))bis(triisopropylsilane) (3p')

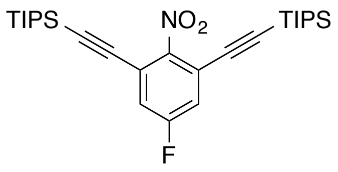

Compound **3p'** was synthesized according to the general procedure using 2 equiv of 1-bromo-2-(triisopropylsilyl)acetylene (**2a**) and it was obtained as a yellow liquid in 30% yield.

^{1}H NMR (400 MHz, $CDCl_3$) δ 7.19 (s, 2H), 1.10 (m, 42H). **^{13}C NMR** (75 MHz, $CDCl_3$) δ 161.5 (d, J = 253.4 Hz), 154.0, 119.9 (d, J = 24.7 Hz), 119.0 (d, J = 11.5 Hz), 101.6, 97.7, 18.5, 11.1. **^{19}F NMR** (376 MHz, $CDCl_3$) δ -108.68 (t, J = 8.1 Hz). **HRMS** (APCI+) *m/z* calc. for $C_{28}H_{45}FNO_2Si_2$ $[M+H]^+$: 502.2967. Found: 502.2965.

((5-Chloro-2-nitrophenyl)ethynyl)triisopropylsilane (3q)

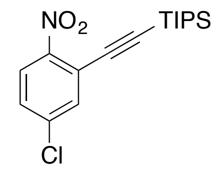

Compound **3q** was synthesized according to the general procedure using 2 equiv of 1-bromo-2-(triisopropylsilyl)acetylene (**2a**) and it was obtained as a white solid in 34% yield.

Mp 60 °C. **^{1}H NMR** (300 MHz, $CDCl_3$) δ 8.00 (d, J = 8.8 Hz, 1H), 7.62 (d, J = 2.3 Hz, 1H), 7.40 (dd, J = 8.8, 2.3 Hz, 1H), 1.14 (s, 21H). **^{13}C NMR** (75 MHz, $CDCl_3$) δ 155.1, 139.2, 135.0, 128.8, 125.9, 120.4, 103.0, 99.9, 18.6, 11.2. **HRMS** (APCI+) *m/z* calc. for $C_{17}H_{25}ClNO_2Si$ $[M+H]^+$: 338.1338. Found: 338.1340.

((5-Chloro-2-nitro-1,3-phenylene)bis(ethyne-2,1-diyl))bis(triisopropylsilane) (3q')

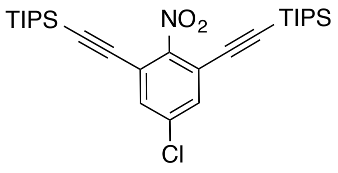

Compound **3q'** was synthesized according to the general procedure using 2 equiv of 1-bromo-2-(triisopropylsilyl)acetylene (**2a**) and it was obtained as a yellow liquid in 18% yield.

^{1}H NMR (300 MHz, $CDCl_3$) δ 7.47 (s, 2H), 1.10 (m, 42H). **^{13}C NMR** (75 MHz, $CDCl_3$) δ 153.1, 135.6, 132.5, 118.3, 101.7, 97.4, 18.5, 11.1. **HRMS** (APCI+) *m/z* calc. for $C_{28}H_{45}ClNO_2Si_2$ $[M+H]^+$: 518.2672. Found: 518.2673.

((6-Iodo-3-methoxy-2-nitrophenyl)ethynyl)triisopropylsilane (3r)

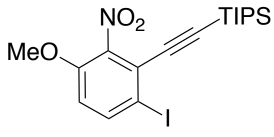

Compound **3r** was synthesized according to the general procedure and it was obtained as a white solid in 55% yield.

Mp 60 °C. **^{1}H NMR** (300 MHz, $CDCl_3$) δ 7.82 (d, J = 8.9 Hz, 1H), 6.75 (d, J = 8.9 Hz, 1H), 3.87 (s, 3H), 1.13 (m, 21H). **^{13}C NMR** (75 MHz, $CDCl_3$) δ 150.4, 143.7, 140.2, 123.4, 114.1, 104.6,

100.5, 89.1, 56.7, 18.6, 11.2. **HRMS** (APCI+) *m/z* calc. for $C_{18}H_{27}INO_3Si$ $[M+H]^+$: 460.0799. Found: 460.0809.

((3-Bromo-6-methoxy-2-nitrophenyl)ethynyl)triisopropylsilane (3s)

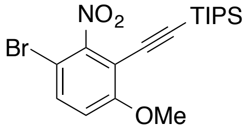

Compound **3s** was synthesized according to the general procedure and it was obtained as a red solid in 81% yield.

Mp 79 °C. **^{1}H NMR** (300 MHz, $CDCl_3$) δ 7.49 (d, *J* = 9.0 Hz, 1H), 6.86 (d, *J* = 9.0 Hz, 1H), 3.91 (s, 3H), 1.12 (s, 21H). **^{13}C NMR** (75 MHz, $CDCl_3$) δ 160.3, 153.9, 133.3, 113.2, 108.3, 105.1, 102.1, 94.3, 56.7, 18.4, 11.1. **HRMS** (APCI+) *m/z* calc. for $C_{18}H_{27}BrNO_3Si$ $[M+H]^+$: 412.0938. Found: 412.0946.

((3,4-Dimethyl-2-nitrophenyl)ethynyl)triisopropylsilane (3t)

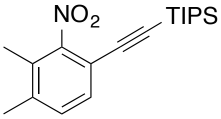

Compound **3t** was synthesized according to the general procedure and it was obtained as a white solid in 86% yield.

Mp 89 °C. **^{1}H NMR** (500 MHz, $CDCl_3$) δ 7.30 (d, *J* = 7.9 Hz, 1H), 7.18 (d, *J* = 7.9 Hz, 1H), 2.32 (s, 3H), 2.18 (s, 3H), 1.10 (s, 21H). **^{13}C NMR** (101 MHz, $CDCl_3$) δ 154.2, 139.3, 131.0, 130.4, 128.0, 113.9, 99.8, 97.6, 20.4, 18.7, 14.4, 11.3. **HRMS** (APCI+) calcd for $[C_{19}H_{30}NO_2Si]^+$ 332.2040 *m/z*; found $[M + H]^+$ 332.2042 *m/z*.

Triisopropyl((3-methyl-2-nitro-4-(trifluoromethyl)phenyl)ethynyl)silane (3u)

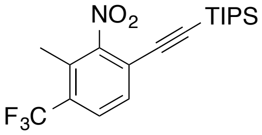

Compound **3u** was synthesized according to the general procedure and it was obtained as a white solid in 32% yield.

Mp 83 °C. **^{1}H NMR** (500 MHz, $CDCl_3$) δ 7.68 (d, *J* = 8.2 Hz, 1H), 7.51 (d, *J* = 8.2 Hz, 1H), 2.39 (d, *J* = 1.6 Hz, 3H), 1.14 – 1.08 (m, 21H). **^{13}C NMR** (126 MHz, $CDCl_3$) δ 155.0, 130.8, 130.1 (q, *J* = 31.3 Hz), 128.8 (q, *J* = 1.4 Hz), 127.1 (q, *J* = 5.7 Hz), 123.2 (q, *J* = 274.13 Hz), 120.1, 102.44, 98.2, 18.6, 13.8 (q, *J* = 2.4 Hz), 11.26. **^{19}F{^{1}H} NMR** (471 MHz, $CDCl_3$) δ -61.39. **HRMS** (APCI+) calcd for $[C_{19}H_{27}F_3NO_2Si]^+$ 386.1758 *m/z*; found $[M + H]^+$ 386.1753 *m/z*.

((4-Fluoro-3-methyl-2-nitrophenyl)ethynyl)triisopropylsilane (3v)

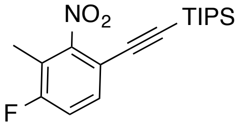

Compound **3v** was synthesized according to the general procedure and it was obtained as a white solid in 65% yield.

Mp 44 °C. **^{1}H NMR** (500 MHz, $CDCl_3$) δ 7.41 (dd, *J* = 8.6, 5.3 Hz, 1H), 7.11 (t, *J* = 8.7 Hz, 1H), 2.23 (d, *J* = 2.1 Hz, 3H), 1.10 (s, 21H). **^{13}C NMR** (126 MHz, $CDCl_3$) δ 160.5 (d, *J* = 252.4 Hz), 154.4, 132.2 (d, *J* = 8.74 Hz), 118.7 (d, *J* = 22.4 Hz), 117.1 (d, *J* = 23.6 Hz), 112.8 (d, *J* = 4.3 Hz), 98.64 (d, *J* = 1.6 Hz), 98.59 (d, *J* = 1.9 Hz), 18.7, 11.3, 10.0 (d, *J* = 4.1 Hz). **^{19}F{^{1}H} NMR**

(471 MHz, $CDCl_3$) δ -110.21. **HRMS** (APCI+) calcd for $[C_{18}H_{27}FNO_2Si]^+$ 336.1790 *m/z*; found $[M + H]^+$ 336.1775 *m/z*.

((4-Chloro-3-methyl-2-nitrophenyl)ethynyl)triisopropylsilane (3w)

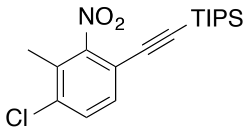

Compound **3w** was synthesized according to the general procedure and it was obtained as a white solid in 65% yield.

Mp 70 °C. **^{1}H NMR** (500 MHz, $CDCl_3$) δ 7.41 (d, J = 8.4 Hz, 1H), 7.35 (d, J = 8.4 Hz, 1H), 2.32 (s, 3H), 1.10 (d, J = 3.5 Hz, 21H). **^{13}C NMR** (126 MHz, $CDCl_3$) δ 154.4, 135.8, 131.4, 130.6, 128.5, 115.1, 100.0, 98.6, 18.6, 15.3, 11.3. **HRMS** (APCI+) calcd for $[C_{18}H_{27}{}^{35}ClNO_2Si]^+$ 352.1494 *m/z*; found $[M + H]^+$ 352.1491 *m/z*.

((4-Bromo-3-methyl-2-nitrophenyl)ethynyl)triisopropylsilane (3x)

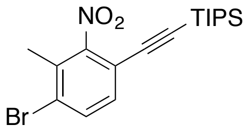

Compound **3x** was synthesized according to the general procedure and it was obtained as a white solid in 67% yield.

Mp 83 °C. **^{1}H NMR** (500 MHz, $CDCl_3$) δ 7.60 (d, J = 8.3 Hz, 1H), 7.27 (d, J = 8.4 Hz, 1H), 2.35 (s, 3H), 1.10 (d, J = 3.6 Hz, 21H). **^{13}C NMR** (126 MHz, $CDCl_3$) δ 154.1, 133.9, 131.5, 130.0, 126.0, 115.7, 100.2, 98.7, 18.6, 18.3, 11.3. **HRMS** (APCI+) calcd for $[C_{18}H_{27}{}^{79}BrNO_2Si]^+$ 396.0989 *m/z*; found $[M + H]^+$ 396.0983 *m/z*.

Triisopropyl((4-methoxy-3-methyl-2-nitrophenyl)ethynyl)silane (3y)

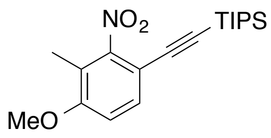

Compound **3y** was synthesized according to the general procedure and it was obtained as a white solid in 81% yield.

Mp 99 °C. **^{1}H NMR** (500 MHz, $CDCl_3$) δ 7.38 (d, J = 8.6 Hz, 1H), 6.84 (d, J = 8.6 Hz, 1H), 3.88 (s, 3H), 2.13 (s, 3H), 1.10 (s, 21H). **^{13}C NMR** (126 MHz, $CDCl_3$) δ 158.2, 154.5, 131.8, 119.2, 111.1, 108.2, 99.7, 96.2, 56.3, 18.68, 11.4, 10.9. **HRMS** (APCI+) calcd for $[C_{19}H_{30}NO_3Si]^+$ 348.1989 *m/z*; found $[M + H]^+$ 348.1993 *m/z*.

((3,5-Dimethyl-2-nitrophenyl)ethynyl)triisopropylsilane (3z)

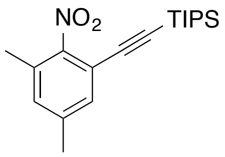

Compound **3z** was synthesized according to the general procedure and it was obtained as a white solid in 95% yield.

Mp 55 °C. **^{1}H NMR** (400 MHz, $CDCl_3$) = 7.23 (s, 1H), 7.04 (s, 1H), 2.34 (s, 3H), 2.30 (s, 3H), 1.13 (s, 21H). **^{13}C NMR** (101 MHz, $CDCl_3$) δ 151.1, 140.2, 131.8, 131.4, 129.8, 116.4, 99.8, 97.9, 20.9, 18.5, 17.4, 11.2. **HRMS** (APCI+) *m/z* calc. for $C_{19}H_{30}NO_2Si$ $[M+H]^+$: 332.2040. Found: 332.2038.

Triisopropyl((5-methyl-4-nitro-[1,1'-biphenyl]-3-yl)ethynyl)silane (3aa)

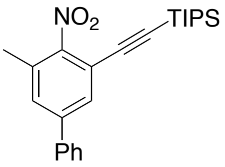

Compound **3aa** was synthesized according to the general procedure and it was obtained as a colorless oil in 79% yield.

^{1}H NMR (500 MHz, $CDCl_3$) δ 7.60 (d, *J* = 0.6 Hz, 1H), 7.57 – 7.54 (m, 2H), 7.49 – 7.44 (m, 2H), 7.44 – 7.38 (m, 2H), 2.40 (s, 3H), 1.17 – 1.10 (m, 21H). **^{13}C NMR** (126 MHz, $CDCl_3$) δ 152.2, 143.2, 138.8, 130.5, 129.9, 129.9, 129.2, 128.6, 127.4, 117.2, 99.8, 98.9, 18.7, 17.9, 11.3. **HRMS** (ESI+): m/z calc. for $C_{24}H_{31}NNaO_2Si$ $[M+Na]^+$: 416.2016. Found: 416.2011.

Triisopropyl((3-methyl-2-nitro-5-(trifluoromethyl)phenyl)ethynyl)silane (3ab)

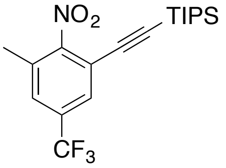

Compound **3ab** was synthesized according to the general procedure and it was obtained as a yellow liquid in 48% yield.

^{1}H NMR (500 MHz, $CDCl_3$) δ 7.68 – 7.64 (m, 1H), 7.50 (dd, *J* = 1.9, 1.0 Hz, 1H), 2.39 (s, 3H), 1.13 – 1.09 (m, 21H). **^{13}C NMR** (126 MHz, $CDCl_3$) δ 154.9, 132.1 (q, *J* = 33.5 Hz), 130.9 (q, *J* = 2.5 Hz), 128.0 (q, *J* = 3.7 Hz), 127.8 (q, *J* = 3.7 Hz), 122.7 (q, *J* = 273.2 Hz), 117.5, 101.4, 97.9 (q, *J* = 2.5 Hz), 18.5, 17.3, 11.1. **^{19}F{^{1}H} NMR** (376 MHz, $CDCl_3$) δ -63.29. **HRMS** (APCI+) *m/z* calc. for $C_{19}H_{27}F_3NO_2Si$ $[M+H]^+$: 386.1758. Found: 386.1763.

((5-Fluoro-3-methyl-2-nitrophenyl)ethynyl)triisopropylsilane (3ac)

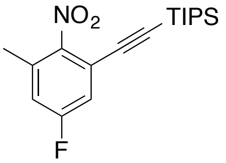

Compound **3ac** was synthesized according to the general procedure and it was obtained as a yellow liquid in 65% yield.

^{1}H NMR (400 MHz, $CDCl_3$) δ 7.10 (ddd, *J* = 8.3, 2.7, 0.6 Hz, 1H), 6.97 – 6.92 (m, 1H), 2.34 (d, *J* = 0.7 Hz, 3H), 1.10 (m, 21H). **^{13}C NMR** (101 MHz, $CDCl_3$) δ 161.8 (d, *J* = 252.3 Hz), 149.7, 133.0 (d, *J* = 9.4 Hz), 118.9 (d, *J* = 10.8 Hz), 118.1 (d, *J* = 23.2 Hz), 117.8 (d, *J* = 24.8 Hz), 100.4, 98.4 (d, *J* = 2.8 Hz), 18.5, 17.8 (d, *J* = 1.4 Hz), 11.1. **^{19}F NMR** (376 MHz, $CDCl_3$) δ -109.29 (t, *J* = 8.4 Hz). **HRMS** (APCI+) *m/z* calc. for $C_{18}H_{27}FNO_2Si$ $[M+H]^+$: 336.1790. Found: 336.1804.

((5-Chloro-3-methyl-2-nitrophenyl)ethynyl)triisopropylsilane (3ad)

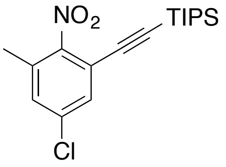

Compound **3ad** was synthesized according to the general procedure and it was obtained as a yellow liquid in 60% yield.

^{1}H NMR (400 MHz, $CDCl_3$) δ 7.39 (dd, *J* = 2.2, 0.7 Hz, 1H), 7.23 (dq, *J* = 2.3, 0.8 Hz, 1H), 2.31 (d, *J* = 0.7 Hz, 3H), 1.10 (m, 21H). **^{13}C NMR** (126 MHz, $CDCl_3$) δ 151.6, 135.6, 131.7, 131.0, 130.7, 118.2, 100.6, 98.2, 18.5, 17.4, 11.1. **HRMS** (APCI+) *m/z* calc. for $C_{18}H_{27}ClNO_2Si$ $[M+H]^+$: 352.1494. Found: 352.1501.

((5-Bromo-3-methyl-2-nitrophenyl)ethynyl)triisopropylsilane (3ae)

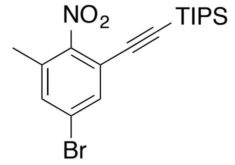

Compound **3ae** was synthesized according to the general procedure and it was obtained as a yellow liquid in 70% yield.

^{1}H NMR (400 MHz, $CDCl_3$) δ 7.55 (dd, *J* = 2.1, 0.7 Hz, 1H), 7.39 (dq, *J* = 1.5, 0.7 Hz, 1H), 2.31 (d, *J* = 0.7 Hz, 3H), 1.12 – 1.09 (m, 21H). **^{13}C NMR** (101 MHz, $CDCl_3$) δ 152.1, 133.9, 133.6, 131.8, 123.5, 118.3, 100.7, 98.1, 18.5, 17.3, 11.1. **HRMS** (APCI+) *m/z* calc. for $C_{18}H_{27}BrNO_2Si$ $[M+H]^+$: 396.0989. Found: 396.0990.

Triisopropyl((5-methoxy-3-methyl-2-nitrophenyl)ethynyl)silane (3af)

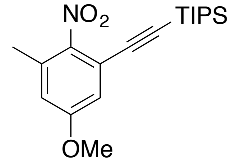

Compound **3af** was synthesized according to the general procedure and it was obtained as a yellow liquid in 95% yield.

^{1}H NMR (500 MHz, $CDCl_3$) δ 6.86 (d, *J* = 2.7 Hz, 1H), 6.71 (dd, *J* = 2.7, 0.8 Hz, 1H), 3.82 (s, 3H), 2.31 (s, 3H), 1.11 (m, 21H). **^{13}C NMR** (126 MHz, $CDCl_3$) δ 159.8, 146.9, 132.4, 118.4, 116.8, 115.6, 99.9, 98.4, 55.7, 18.5, 18.2, 11.1. **HRMS** (APCI+) *m/z* calc. for $C_{19}H_{30}NO_3Si$ $[M+H]^+$: 348.1989. Found: 348.1999.

((3-Bromo-5-methoxy-2-nitrophenyl)ethynyl)triisopropylsilane (3ag)

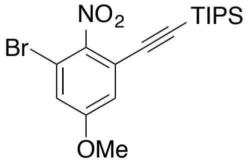

Compound **3ag** was synthesized according to the general procedure and it was obtained as a yellow solid in 72% yield.

Mp 85 °C. **^{1}H NMR** (300 MHz, $CDCl_3$) δ 7.50 (d, *J* = 9.0 Hz, 1H), 6.87 (d, *J* = 9.0 Hz, 1H), 3.92 (s, 3H), 1.12 (s, 21H). **^{13}C NMR** (75 MHz, $CDCl_3$) δ 160.4, 133.3, 113.2, 108.4, 106.3, 105.1, 102.2, 94.3, 56.7, 18.5, 11.1. **HRMS** (ESI+) *m/z* calc. for $C_{18}H_{26}BrNNaO_3Si$ $[M+H]^+$: 434.0758. Found: 434.0756.

triisopropyl((1-nitronaphthalen-2-yl)ethynyl)silane (3ah)

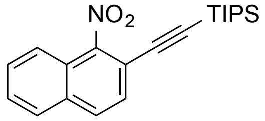

Compound **3ah** was synthesized according to the general procedure and it was obtained as a red solid in 41% yield.

Mp 67 °C. **^{1}H NMR** (300 MHz, $CDCl_3$) δ 7.92 – 7.85 (m, 2H), 7.75 (dt, *J* = 8.6, 1.1 Hz, 1H), 7.67 – 7.53 (m, 3H), 1.14 (m, 21H). **^{13}C NMR** (75 MHz, $CDCl_3$) δ 150.8, 133.1, 130.2, 129.0, 128.3, 128.1, 128.0, 124.1, 121.7, 114.0, 100.8, 99.8, 18.6, 11.2. **HRMS** (ESI+) *m/z* calc. for $C_{18}H_{26}BrNNaO_3Si$ $[M+Na]^+$: 354.1884. Found: 354.1887.

Triisopropyl((1-nitropyren-2-yl)ethynyl)silane (3ai)

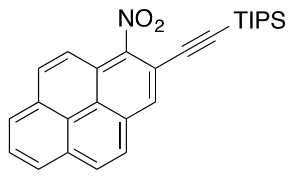

Compound **3ai** was synthesized according to the general procedure at 1 mmol scale and it was obtained as a brown solid in 80% yield.

Mp 120 °C. **^{1}H NMR** (300 MHz, $CDCl_3$) δ 8.30 – 8.07 (m, 6H), 7.98 (m, 2H), 1.22 (s, 21H). **^{13}C NMR** (75 MHz, $CDCl_3$) δ 149.9, 146.5, 131.6, 130.7, 130.1, 129.8, 128.2, 127.3, 127.1, 126.7, 125.9, 123.6, 123.2, 122.4, 119.9, 113.7, 100.4, 99.1, 18.6, 11.3. **HRMS** (APCI+) *m/z* calc. for $C_{27}H_{30}NO_2Si$ $[M+H]^+$: 428.2040. Found: 428.2033.

((5-bromo-2-nitrothiophen-3-yl)ethynyl)triisopropylsilane (3aj)

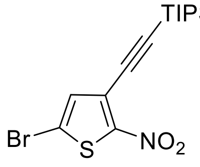

Compound **3aj** was synthesized according to the general procedure using 2 equiv. of heteroarene **1aj** and it was obtained as an orange solid in 45% yield.

^{1}H NMR (500 MHz, $CDCl_3$) δ 6.97 (s, 1H), 1.60 (hept, *J* = 7.5 Hz, 3H), 1.12 (d, *J* = 7.5 Hz, 18H). **^{13}C NMR** (126 MHz, $CDCl_3$) δ 165.3, 161.2, 150.3, 133.25, 126.9, 124.3, 18.8, 12.5. **HRMS** (ESI) m/z calc. for $C_{15}H_{22}BrNNaO_2SSi$ $[M+Na]^+$: 410.0216. Found: 410.0211.

7-nitro-6-((triisopropylsilyl)ethynyl)-1*H*-indole (3ak)

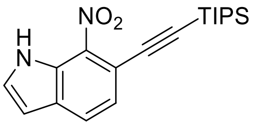

Compound **3ak** was synthesized according to the general procedure using 2 equiv. of heteroarene **1ak** and it was obtained as an orange solid in 60% yield.

^{1}H NMR (500 MHz, $CDCl_3$) δ 10.01 (s, 1H), 7.82 (dd, *J* = 8.1, 0.7 Hz, 1H), 7.49 – 7.35 (m, 2H), 6.67 (d, *J* = 1.0 Hz, 1H), 1.20-1.17 (m, 21H). **^{13}C NMR** (126 MHz, $CDCl_3$) δ 133.9, 131.1, 129.6, 127.65, 127.1, 126.8, 114.7, 104.1, 103.95, 100.45, 18.7, 11.4. **HRMS** (ESI) m/z calc. for $C_{19}H_{26}N_2NaO_2Si$ $[M+Na]^+$: 365.1656. Found: 365.1656.

1-methyl-4-nitro-5-((triisopropylsilyl)ethynyl)-1*H*-indole (3al)

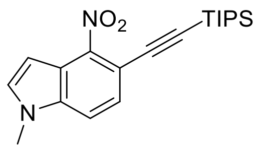

Compound **3al** was synthesized according to the general procedure using 2 equiv. of heteroarene **1al** and it was obtained as an orange solid in 72% yield.

^{1}H NMR (300 MHz, $CDCl_3$) δ 7.49-7.43 (m, 2H), 7.26 (d, *J* = 3.2 Hz, 1H), 6.96 (d, *J* = 3.1 Hz, 1H), 3.87 (s, 1H), 1.22-1.12 (m, 21H). **^{13}C NMR** (126 MHz, $CDCl_3$) δ 142.7, 137.7, 133.0, 127.5, 122.6, 113.6, 111.1, 103.1, 101.5, 97.55, 33.3, 18.7, 11.4. **HRMS** (ESI) m/z calc. for $C_{20}H_{28}N_2NaO_2Si$ $[M+Na]^+$: 379.1812. Found: 379.1815.

1-methyl-5-nitro-4-((triisopropylsilyl)ethynyl)-1*H*-indole (3am)

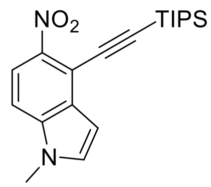

Compound **3am** was synthesized according to the general procedure using 2 equiv. of heteroarene **1am** and it was obtained as an orange solid in 48% yield.

^{1}H NMR (500 MHz, $CDCl_3$) δ 8.02 (d, *J* = 9.0 Hz, 1H), 7.28 (dd, *J* = 9.0, 0.8 Hz, 1H), 7.21 (d, *J* = 3.2 Hz, 1H), 6.83 (dd, *J* = 3.2, 0.8 Hz, 1H), 3.85 (s, 3H), 1.38 – 0.73 (m, 21H). **^{13}C NMR** (126 MHz, $CDCl_3$) δ 143.0, 137.8, 132.1, 131.5, 118.6, 112.45, 109.0, 103.95, 103.5, 100.5, 33.3, 18.7, 11.4. **HRMS** (ESI) m/z calc. for $C_{20}H_{28}N_2N_aO_2Si$ $[M+Na]^+$: 379.1812. Found: 379.1802.

1-methyl-5-nitro-4,6-bis((triisopropylsilyl)ethynyl)-1*H*-indole (3am')

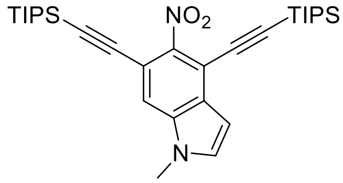

Compound **3am'** was synthesized according to the general procedure using 2 equiv. of 1-bromo-2-(triisopropylsilyl)acetylene (**2a**) and it was obtained as a brown solid in 40% yield.

^{1}H NMR (500 MHz, $CDCl_3$) δ 7.42 (d, *J* = 0.9 Hz, 1H), 7.23 (d, *J* = 3.1 Hz, 1H), 6.67 (dd, *J* = 3.1, 0.9 Hz, 1H), 3.82 (s, 3H), 1.17-1.11 (m, 42H). .**^{13}C NMR** (126 MHz, $CDCl_3$) δ 148.7, 135.3, 133.2, 129.75, 114.1, 109.9, 109.4, 103.1, 101.5, 100.9, 98.1, 95.93, 33.3, 18.62, 18.60, 11.3, 11.2. **HRMS** (ESI) m/z calc. for $C_{31}H_{48}N_2NaO_2Si_2$ $[M+Na]^+$: 559.3147. Found: 559.3126.

(*S*)-((2,2'-Dimethoxy-3'-nitro-[1,1'-binaphthalene]-3,4'-diyl)bis(ethyne-2,1-diyl))bis(triisopropylsilane) (3an)

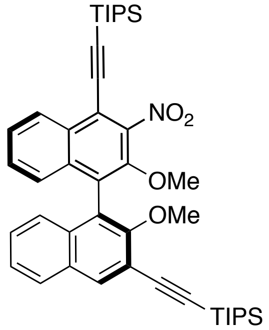

Compound **3an** was synthesized according to the general procedure, using 2 equiv. of 1-bromo-2-(triisopropylsilyl)acetylene (**2a**) and it was obtained as a yellow solid in 42% yield.

^{1}H NMR (400 MHz, $CDCl_3$) δ 8.55 (dd, *J* = 9.3, 0.6 Hz, 1H), 8.02 (d, *J* = 9.0 Hz, 1H), 7.89 (d, *J* = 8.0 Hz, 1H), 7.63 (d, *J* = 9.3 Hz, 1H), 7.46 (d, *J* = 9.1 Hz, 1H), 7.35 (ddd, *J* = 8.1, 6.8, 1.2 Hz, 1H), 7.32 – 7.22 (m, 2H), 7.03 (d, *J* = 8.5 Hz, 1H), 3.79 (s, 3H), 3.77 (s, 3H), 1.21 (q, *J* = 3.8 Hz, 21H), 1.01 (d, *J* = 3.0 Hz, 21H). **^{13}C NMR** (101 MHz, $CDCl_3$) δ 157.3, 155.0, 151.5, 133.85, 133.4, 131.1, 130.4, 129.3, 129.0, 128.2, 127.4, 126.9, 124.9, 123.85, 120.3, 117.6, 116.7, 114.6, 113.8, 105.2, 99.8, 98.3, 97.5, 56.7, 56.7, 18.8, 18.6, 11.4, 11.3. **HRMS** (ESI+): m/z calc. for $C_{44}H_{57}NNaO_4Si_2$ $[M+Na]^+$: 742.3718. Found: 742.3727.

Triethyl((4-methoxy-3-methyl-2-nitrophenyl)ethynyl)silane (3ap)

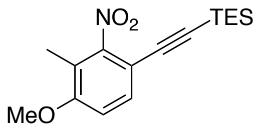

Product **3ap** was synthesized following general procedure starting from 1-methoxy-2-methyl-3-nitrobenzene (30.0 mg, 0.179 mmol). Purification by flash chromatography on silica (pentane:diethyl ether, 98:2) afforded the title compound **3ap** (33.6 mg, 0.110 mmol, 61% yield) as a light yellow oil.

^{1}H NMR (500 MHz, $CDCl_3$) δ 7.38 (dd, *J* = 8.6, 0.4 Hz, 1H), 6.84 (d, *J* = 8.6 Hz, 1H), 3.88 (s, 3H), 2.13 (s, 3H), 1.02 (t, *J* = 7.9 Hz, 9H), 0.64 (q, *J* = 7.9 Hz, 6H). **^{13}C NMR** (126 MHz, $CDCl_3$) δ 158.2, 154.5, 131.8, 119.3, 111.2, 108.1, 99.2, 97.2, 56.3, 10.9, 7.5, 4.4. **HRMS** (ESI+) calcd for $[C_{16}H_{23}NO_3Si]^+$ 328.1339 *m/z*; found $[M + H]^+$ 328.1345 *m/z*.

Tri(*n*-hexyl)((4-methoxy-3-methyl-2-nitrophenyl)ethynyl)silane (3aq)

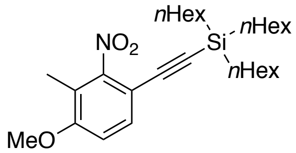

Product **3aq** was synthesized following general procedure starting from 1-methoxy-2-methyl-3-nitrobenzene (30.0 mg, 0.179 mmol). Purification by flash chromatography on silica (pentane:diethyl ether, 98:2) afforded the title compound **3aq** (53.1 mg, 0.112 mmol, 63% yield) a light yellow oil.

^{1}H NMR (500 MHz, $CDCl_3$) δ 7.36 (d, *J* = 8.5 Hz, 1H), 6.84 (d, *J* = 8.6 Hz, 1H), 3.87 (s, 3H), 2.13 (s, 3H), 1.42 – 1.23 (m, 24H), 0.91 – 0.85 (m, 9H), 0.66 – 0.60 (m, 6H). **^{13}C NMR** (126 MHz, $CDCl_3$) δ 158.2, 154.5, 131.7, 119.3, 111.1, 108.2, 99.1, 98.0, 56.3, 33.3, 31.7, 23.9, 22.8, 14.3, 13.3, 10.9. **HRMS** (ESI+) calcd for $[C_{28}H_{48}NO_3Si]^+$ 474.3398 *m/z*; found $[M + H]^+$ 474.3390 *m/z*.

***Tert*-butyl((4-methoxy-3-methyl-2-nitrophenyl)ethynyl)diphenylsilane (3ar)**

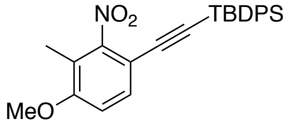

Product **3ar** was synthesized following general procedure starting from 1-methoxy-2-methyl-3-nitrobenzene (30.0 mg, 0.179 mmol). Purification by flash chromatography on silica (pentane:diethyl ether, 95:5) afforded the title compound **3ar** (57.2 mg, 0.133 mmol, 74% yield) as a light yellow solid.

M.p. (pentane) = 108–110 ºC. **^{1}H NMR** (500 MHz, $CDCl_3$) δ 7.84 – 7.79 (m, 4H), 7.50 (d, *J* = 8.6 Hz, 1H), 7.43 – 7.37 (m, 6H), 6.89 (d, *J* = 8.6 Hz, 1H), 3.90 (s, 3H), 2.18 (s, 3H), 1.12 (s, 9H). **^{13}C NMR** (126 MHz, $CDCl_3$) ^{13}C NMR (126 MHz, $CDCl_3$) δ 158.6, 154.4, 135.8, 133.0, 132.1, 129.7, 127.9, 119.5, 111.3, 107.8, 101.8, 94.9, 56.4, 27.1, 18.9, 11.0. **HRMS** (ESI+) calcd for $[C_{26}H_{28}NO_3Si]^+$ 430.1833 *m/z*; found $[M + H]^+$ 430.1827 *m/z*.

Triphenyl((4-methoxy-3-methyl-2-nitrophenyl)ethynyl)silane (3as)

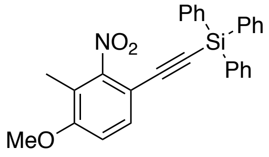

Product **3as** was synthesized following general procedure starting from 1-methoxy-2-methyl-3-nitrobenzene (30.0 mg, 0.179 mmol). Purification by flash chromatography on silica

(pentane:diethyl ether, 95:5) afforded the title compound **3as** (26.1 mg, 0.058 mmol, 32% yield) as a light yellow solid.

M.p. (pentane) = 161–164 ºC. **^{1}H NMR** (500 MHz, $CDCl_3$) δ 7.71 – 7.64 (m, 6H), 7.49 (d, J = 8.6 Hz, 1H), 7.46 – 7.37 (m, 9H), 6.87 (s, 1H), 3.89 (s, 3H), 2.17 (s, 3H). **^{13}C NMR** (126 MHz, $CDCl_3$) δ 158.8, 154.4, 135.8, 133.2, 132.2, 130.2, 128.2, 128.1, 119.7, 111.3, 107.6, 102.4, 94.5, 56.4, 11.0. **HRMS** (ESI+) calcd for $[C_{28}H_{24}NO_3Si]^+$ 450.1520 *m/z*; found $[M + H]^+$ 450.1509 *m/z*.

Unsuccessful Nitro-Heteroarenes

We present herein a list of nitro-heteroarenes that did not engage in the Rh-catalyzed alkynylation. Desired product was not observed, by ^{1}H-NMR or GCMS, in any case for the following substrates:

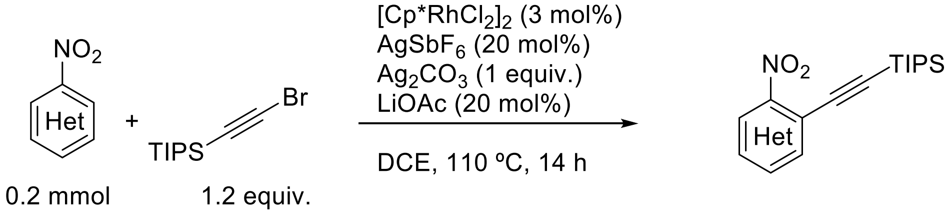

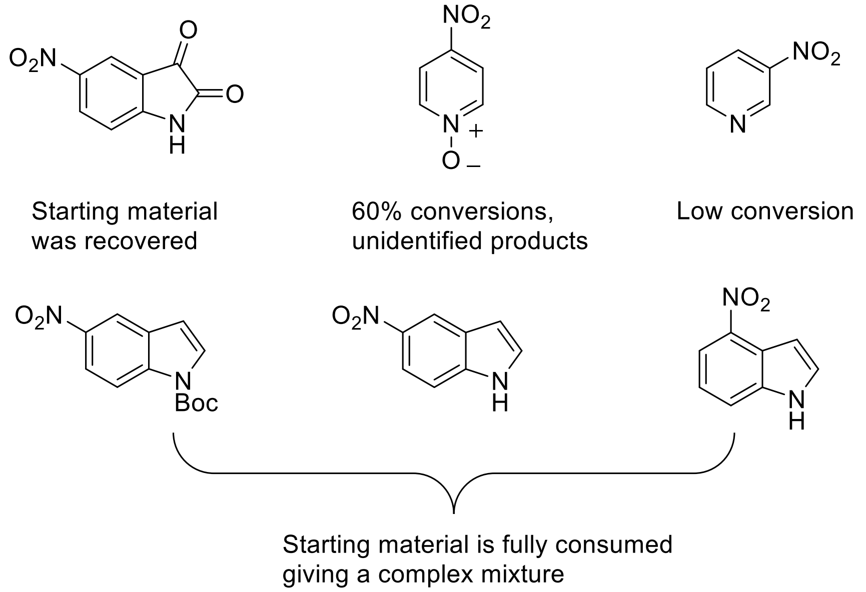

3. Synthetic Transformations

1-Ethynyl-2-nitrobenzene (4)

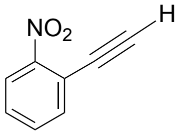

A solution of TBAF (1 M in THF, 1.2 equiv) is added to a solution of **3b** in THF (0.2 M) at 25 °C. The corresponding solution is stirred for 1 hour, and the volatiles were moved by rotatory evaporation. The crude material was purified using a gradient from cyclohexane 100% to 5/1 cyclohexane/ethyl acetate to yield product **4** in 83% yield.

^{1}H NMR (300 MHz, $CDCl_3$) δ 8.04 (dd, *J* = 8.1, 1.4 Hz, 1H), 7.70 (dd, *J* = 7.7, 1.6 Hz, 1H), 7.59 (td, *J* = 7.6, 1.4 Hz, 1H), 7.50 (ddd, *J* = 8.1, 7.4, 1.6 Hz, 1H), 3.51 (s, 1H). **^{13}C NMR** (126 MHz, $CDCl_3$) δ 150.8, 135.7, 132.9, 129.5, 124.7, 117.6, 85.3, 78.7. **MS** (EI) *m/z* calc. for $C_8H_5NO_2$ $[M]^+$: 147.0. Found: 147.0. The spectroscopic data agrees with previously reported values.[2]

2-((Triisopropylsilyl)ethynyl)aniline (5)

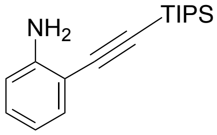

To a solution of **3b** in a mixture of EtOH/H_2O (10:1, 0.05 M) AcOH (30 equiv) is added, followed by Fe (10 equiv). The suspension is stirred at room temperature overnight.

The reaction mixture is filtered through a pad of Celite®, washing with EtOAc. The mixture is then poured into a separating flask, and washed with aq. sat. $NaHCO_3$, H_2O and brine. The organic layer is dried over $MgSO_4$. The crude mater is purified by flash column chromatography using 5% DCM in cyclohexane as eluent, affording compound **5** a yellow oil in 90% yield.

^{1}H NMR (400 MHz, $CDCl_3$) δ 7.36 – 7.31 (m, 1H), 7.13 (ddd, *J* = 8.2, 7.3, 1.6 Hz, 1H), 6.74 – 6.61 (m, 2H), 4.27 (s, 2H), 1.19 – 1.14 (m, 21H). **^{13}C NMR** (101 MHz, $CDCl_3$) δ 148.4, 132.6, 129.8, 117.8, 114.3, 108.4, 103.9, 96.0, 18.9, 11.4. **HRMS** (ESI+) *m/z* calc. for $C_{17}H_{28}NSi$ $[M+H]^+$: 274.1986. Found: 274.1984. The spectroscopic data agrees with previously reported values.[3]

2-(Triisopropylsilyl)-1*H*-indole (6)

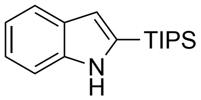

Compound **5** and [(Johnphos)Au($NCCH_3$)]SbF_6 (5 mol%) were dissolved in toluene (0.1 M) and the solution was stirred at 70 ºC for 2 h. Then, the solvent was removed by rotatory evaporation, and the crude mixture was purified by flash column chromatography using 5% DCM in cyclohexane as eluent, affording compound **6** in 90% yield.

^{1}H NMR (400 MHz, $CDCl_3$) δ 8.09 (s, 1H), 7.65 (dd, *J* = 7.8, 1.1 Hz, 1H), 7.42 (dd, *J* = 8.1, 1.0 Hz, 1H), 7.19 (ddd, *J* = 8.1, 7.0, 1.2 Hz, 1H), 7.10 (ddd, *J* = 8.0, 7.0, 1.0 Hz, 1H), 6.78 (dd, *J* = 2.1, 1.0 Hz, 1H), 1.39 (d, *J* = 7.2 Hz, 3H), 1.15 (d, *J* = 7.4 Hz, 18H). **^{13}C NMR** (101 MHz, $CDCl_3$) δ 138.7, 133.8, 128.7, 122.2, 120.6, 119.6, 113.7, 110.8, 18.8, 11.4. **HRMS** (ESI-) *m/z* calc. for $C_{17}H_{26}NSi$ $[M-H]^-$: 272.1840. Found: 272.1835.

(*S*)-*N*-(1-Phenylethyl)-2-((triisopropylsilyl)ethynyl)aniline (7)

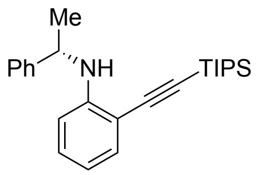

To an oven-dried tube equipped with a magnetic stirring bar were added sequentially **3b** (0.10 mmol), (*S*)-1-phenylethan-1-amine (1.5 Eq, 150 µmol), Pd(acac)$_2$ (0.05 Eq, 5.00 µmol), NHC ligand (0.1 Eq, 10.0 µmol), $K_3PO_4 \cdot 2H_2O$ (3 Eq, 300 µmol), and 1,4-Dioxane (0.2 M) under Ar atmosphere. The reaction mixture was stirred at room temperature for 30 minutes and then heated at 130 °C for 24 hours.

The reaction mixture was then cooled to room temperature, and then it was passed through a short pad of Celite® with CH_2Cl_2. The solution was concentrated *in vacuo* and the residue was purified by silica gel column chromatography (5% EA in Cyclohexane) to give pure **7** as a colorless oil in 63% yield.

[α]^D= +220.4 (c=0.48, $CHCl_3$). **^{1}H NMR** (500 MHz, $CDCl_3$) δ 7.38 – 7.30 (m, 5H), 7.25 – 7.18 (m, 1H), 7.02 (ddd, *J* = 8.3, 7.3, 1.6 Hz, 1H), 6.55 (td, *J* = 7.5, 1.1 Hz, 1H), 6.34 (dd, *J* = 8.3, 1.0 Hz, 1H), 5.18 (s, 1H), 4.55 (p, *J* = 6.3 Hz, 1H), 1.55 (d, *J* = 6.7 Hz, 3H), 1.17 (s, 21H). **^{13}C NMR** (126 MHz, $CDCl_3$) δ 148.6, 145.0, 132.1, 129.9, 128.8, 127.1, 125.9, 116.2, 110.7, 108.0, 104.15, 96.6, 53.3, 25.2, 18.9, 11.5. **HRMS** (ESI+): m/z calc. for $C_{25}H_{36}NSi$ [M+H]$^+$: 378.2612. Found: 378.2610.

(*S*)-1-Methyl-*N*-(1-phenylethyl)-5-((triisopropylsilyl)ethynyl)-1*H*-indol-4-amine (8)

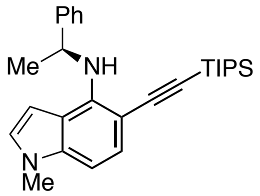

Compound **8** was synthesized from compound **3al** as described for compound **7**, and it was obtained as a yellow oil in 60% yield.

[α]^D= +131.23 (c=0.40, $CHCl_3$). **^{1}H NMR** (400 MHz, $CDCl_3$) δ 7.46 – 7.39 (m, 2H), 7.30 – 7.24 (m, 3H), 7.22 – 7.15 (m, 2H), 6.81 (d, *J* = 3.3 Hz, 1H), 6.66 (d, *J* = 8.6 Hz, 1H), 6.48 (d, *J* = 3.3 Hz, 1H), 5.26 (q, *J* = 6.7 Hz, 1H), 3.65 (s, 3H), 1.64 (d, *J* = 6.7 Hz, 3H), 1.16 (s, 21H). **^{13}C NMR** (126 MHz, $CDCl_3$) δ 146.1, 144.4, 138.7, 128.6, 127.0, 126.8, 126.3, 125.9, 115.9, 106.6, 101.2, 99.9, 98.7, 93.8, 54.9, 33.1, 25.7, 19.0, 11.6. **HRMS** (ESI+): m/z calc. for $C_{28}H_{39}N_2Si$ [M+H]$^+$: 431.2877. Found: 431.2883.

(*S*)-*N*-(1-Phenylethyl)-2-((triisopropylsilyl)ethynyl)pyren-1-amine (9)

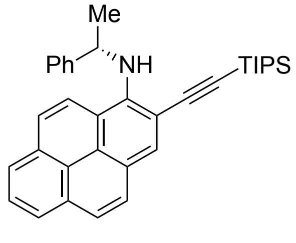

Compound **9** was synthesized from compound **3ai** as described for compound **7**, and it was obtained as a yellow solid in 63% yield.

Mp 142 °C. [α]^D= –116.6 (c=0.12, $CHCl_3$). **^{1}H NMR** (400 MHz, $CDCl_3$) δ 8.22 (d, *J* = 9.3 Hz, 1H), 8.16 (s, 1H), 8.04 (ddd, *J* = 7.8, 3.9, 1.2 Hz, 2H), 7.97 – 7.87 (m, 2H), 7.84 (d, *J* = 0.5 Hz, 2H), 7.49 – 7.42 (m, 2H), 7.32 – 7.27 (m, 2H), 7.24 – 7.17 (m, 1H), 5.10 (s, 2H), 1.62 (d, *J* = 4.9 Hz, 3H), 1.27 – 1.11 (m, 21H). **^{13}C NMR** (126 MHz, $CDCl_3$) δ 145.0, 144.8, 132.2, 131.7, 128.9, 128.6, 127.2, 127.1, 126.5, 126.5, 126.4, 126.3, 125.7, 125.5, 125.3, 124.6, 124.4, 123.3, 121.9,

113.7, 105.1, 96.9, 60.0, 24.3, 19.0, 11.6. **HRMS** (ESI+): m/z calc. for $C_{35}H_{40}NSi$ $[M+H]^+$: 502.2925. Found: 502.2933.

(*S*)-9-(1-Phenylethyl)-9*H*-phenaleno[1,9-*fg*]indole (10)

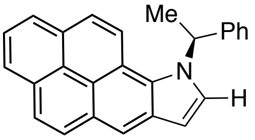

Compound **10** was synthesized from compound **9**, after deprotection/cyclization sequence as described for compounds **4** and **6**, respectively. It was obtained as a yellow oil in 80% yield (two steps).

[α]^D^= –123.0 (c=0.15, $CHCl_3$). **^{1}H NMR** (500 MHz, $CDCl_3$) δ 8.59 (d, *J* = 9.3 Hz, 1H), 8.08 – 8.04 (m, 2H), 8.01 (d, *J* = 9.3 Hz, 1H), 7.90 (t, *J* = 7.6 Hz, 1H), 7.87 (d, *J* = 9.0 Hz, 1H), 7.69 (d, *J* = 3.3 Hz, 1H), 7.33 – 7.28 (m, 1H), 7.27 – 7.22 (m, 1H), 7.19 – 7.15 (m, 1H), 7.06 (d, *J* = 3.3 Hz, 1H), 6.68 (q, *J* = 7.0 Hz, 1H). **^{13}C NMR** (126 MHz, $CDCl_3$) δ 143.4, 131.8, 130.9, 130.3, 129.1, 128.8, 128.2, 127.7, 127.3, 127.2, 126.3, 126.1, 125.8, 125.0, 124.9, 124.0, 123.8, 122.7, 121.6, 118.9, 117.5, 102.6, 58.1, 23.8. **HRMS** (ESI Pos): calculated for $C_{26}H_{20}N$ $[M+H]^+$: 346.1590; found: 346.1587.

Triisopropyl((1-phenylpyren-2-yl)ethynyl)silane (11)

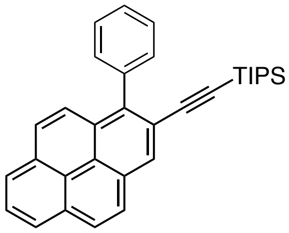

A MW vial was charged with BrettPhos (0.20 Eq, 20 μmol), $Pd(acac)_2$ (0.05 Eq, 5.0 μmol), **3ai** (0.10 mmol), $K_3PO_4{\cdot}2H_2O$ (2 Eq, 0.20 mmol) and phenylboronic acid (2 Eq, 0.20 mmol). In the blovebox, 18-crown-6 ether (0.1 Eq, 10 μmol) was added followed by 1,4-dioxane (0.5 mL). The reaction was sealed and heated out of the glovebox at 130 ºC for 24 hours.

After completion of the reaction, it was allowed to cool to room temperature. The reaction mixture was passed through a short pad of Celite® with CH_2Cl_2 and the solution was concentrated *in vacuo*. The crude residue was purified by column chromatogrpahy on silica gel (cyclohexane) to give compound **11** as a yellow solid in 40% yield.

Mp 118 °C. **^{1}H NMR** (400 MHz, $CDCl_3$) δ 8.40 (s, 1H), 8.20 – 7.91 (m, 6H), 7.82 (d, *J* = 9.3 Hz, 1H), 7.49 (dd, *J* = 24.6, 4.3 Hz, 5H), 1.01 (s, 21H). **^{13}C NMR** (101 MHz, $CDCl_3$) δ 140.2, 139.5, 131.7, 131.1, 131.0, 130.4, 129.5, 128.9, 128.2, 128.1, 127.9, 127.5, 127.0, 126.5, 125.9, 125.5, 125.3, 124.7, 124.5, 121.2, 107.0, 94.7, 18.7, 11.4. **HRMS** (APCI Pos): calculated for $C_{33}H_{35}Si$ $[M+H]^+$: 459.2503; found: 459.2504.

2-Ethynyl-1-(naphthalen-2-yl)pyrene (13)

Compound **12** was synthesized from compound **3ai** as described for compound **11**. This compound was obtained impure by the presence of binaphthyl side-product. The crude material was subjected to deprotection reaction conditions, as described for compound **4**, yielding compound **13** as a white solid in 60% yield.

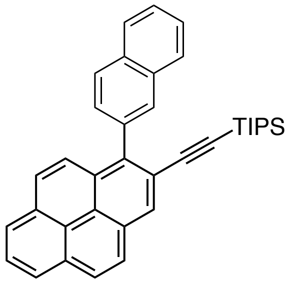

Mp 123 °C. **^{1}H NMR** (500 MHz, $CDCl_3$) δ 8.46 (s, 1H), 8.21 (dd, *J* = 7.6, 0.8 Hz, 1H), 8.18 – 8.14 (m, 1H), 8.12 (d, *J* = 9.0 Hz, 1H), 8.07 (d, *J* = 9.0 Hz, 1H), 8.06 – 7.98 (m, 4H), 7.97 – 7.92 (m, 2H), 7.86 (d, *J* = 9.3 Hz, 1H), 7.68 (dd, *J* = 8.2, 1.8 Hz, 1H), 7.62 – 7.55 (m, 2H), 3.02 (s, 1H). **^{13}C NMR** (126 MHz, $CDCl_3$) δ 139.8, 136.5, 133.2, 132.8, 131.6, 131.0, 130.4, 130.0, 129.6, 129.1, 128.9, 128.3, 128.2, 128.0, 127.9, 127.6, 126.9, 126.6, 126.2, 126.2, 125.6, 125.6, 125.3, 124.7, 124.5, 119.7, 83.6, 80.8. **HRMS** (ESI Pos): calculated for $C_{28}H_{17}$ $[M+H]^+$: 353.1325; found: 353.1322.

4. Alkynylation of Nitrendipine

3-Ethyl 5-methyl 2,6-dimethyl-4-(3-nitro-4-((triisopropylsilyl)ethynyl)phenyl)pyridine-3,5-dicarboxylate (14)

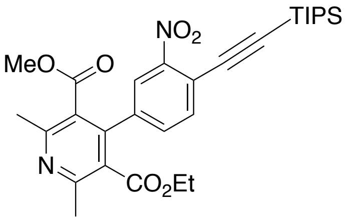

Compound **14** was synthesized according to the general procedure starting from commercial nitrendipine and it was obtained as a purple liquid in 40% yield.

^{1}H NMR (300 MHz, $CDCl_3$) δ 7.97 (d, *J* = 1.8 Hz, 1H), 7.69 (d, *J* = 8.0 Hz, 1H), 7.46 (dd, *J* = 8.0, 1.8 Hz, 1H), 4.14 (q, *J* = 7.1 Hz, 2H), 3.67 (s, 3H), 2.64 (s, 3H), 2.63 (s, 3H), 1.17 (s, 21H), 1.10 (t, *J* = 7.1, 3H). **^{13}C NMR** (75 MHz, $CDCl_3$) δ 179.1, 167.6, 167.1, 156.2, 149.6, 143.2, 137.0, 135.3, 132.2, 126.6, 126.3, 124.2, 118.8, 102.8, 100.5, 61.9, 52.6, 23.0, 18.6, 18.3, 13.8, 11.2. **HRMS** (ESI+) *m/z* calc. for $C_{29}H_{39}N_2O_6Si$ $[M+H]^+$: 539.2572. Found: 539.2572.

5. Rhodium-Catalyzed C–H Iodination of Nitrobenzene 1z

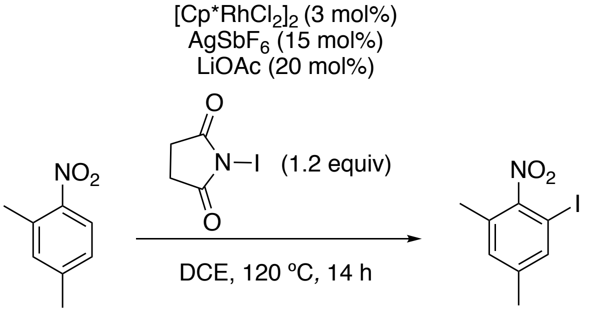

1z **15**

1-Iodo-3,5-dimethyl-2-nitrobenzene (15)

$[Cp^*RhCl_2]_2$ (3 mol%), LiOAc (0.2 equiv), $AgSbF_6$ (0.15 equiv) were weighted in a vial inside a glovebox and dichloroethane (0.2M) is added. 2,4-Dimethyl-1-nitrobenzene (0.2 mmol) and *N*-iodosuccinimide (1.2 equiv) are then added and the vial is sealed. The reaction mixture is stirred at 120 °C for 14 h. After cooling to the room temperature, the reaction mixture is filtrated through celite and purified by column chromatography, with a gradient from cyclohexane 100% to 9/1 cyclohexane/ethyl acetate to yield 1-iodo-3,5-dimethyl-2-nitrobenzene **15** in 60% yield as a yellow liquid.

^{1}H NMR (300 MHz, $CDCl_3$) δ 7.55 (s, 1H), 7.07 (s, 1H), 2.31 (s, 3H), 2.33 (s, 3H).

The ^{1}H NMR data is consistent with the one previously reported.[4]

One-pot *ortho*-arylation of nitrobenzene

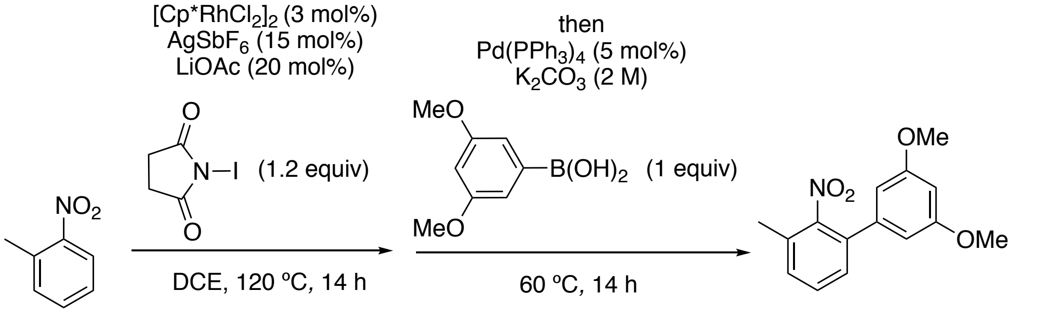

3a **16**

3',5'-Dimethoxy-3-methyl-2-nitro-1,1'-biphenyl (16)

$[Cp^*RhCl_2]_2$ (3 mol %), LiOAc (0.2 equiv), $AgSbF_6$ (0.15 equiv) were weighted in a vial inside a glovebox and dichloroethane (0.2 M) is added. 2-Methyl-1-nitrobenzene (0.2 mmol) and *N*-iodosuccinimide (1.2 equiv) are then added and the vial is sealed. The reaction mixture is stirred at 120 °C for 14 h. After cooling to the room temperature, the vial is opened and $Pd(PPh_3)_4$ (5 mol%), K_2CO_3 (1 mL, 2M in water) and 3',5'-dimethoxy-3-methyl-2-nitro-1,1'-biphenyl (1 equiv) are then added. The reaction is stirred at 60 °C for 14 hours. After cooling to the room temperature, the reaction is diluted with water, extracted with DCM, dried over $MgSO_4$. The crude is then purified by column chromatography, with a gradient from cyclohexane 100% to 1/1 cyclohexane/ethyl acetate to yield **16** in 50% yield as a yellow solid.

Mp 90 °C. **^{1}H NMR** (300 MHz, $CDCl_3$) δ 7.64 – 7.61 (m, 1H), 7.40 (ddt, J = 7.8, 1.4, 0.7 Hz, 1H), 7.32 (d, J = 7.8 Hz, 1H), 6.49 – 6.46 (m, 1H), 6.43 (m, 2H), 3.79 (d, J = 0.6 Hz, 6H), 2.46 (d, J = 0.7 Hz, 3H). **^{13}C NMR** (75 MHz, $CDCl_3$) δ 160.8, 139.3, 138.8, 133.3, 132.9, 131.5, 126.9, 124.2, 106.2, 100.0, 55.4, 20.9. **HRMS** (APCI+) *m/z* calc. for $C_{15}H_{16}NO_4$ $[M+H]^+$: 274.1074. Found: 274.1083.

6. Experimental Mechanistic Investigations

Kinetic Isotope Effect

Parallel experiments (initial rates measurement)

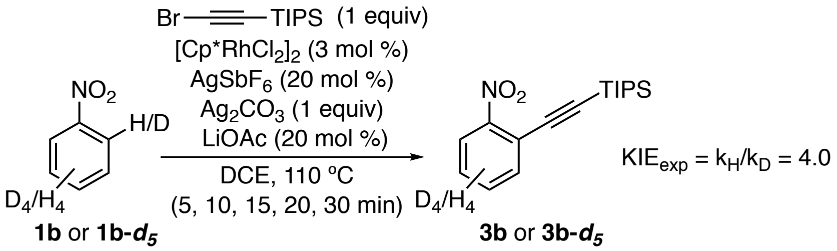

The kinetic isotope effect (KIE) was determined by measuring the initial rates of the reactions with hydrogenated and deuterated substrates. 5 Reactions with hydrogenated substrates and 5 reactions with deuterated substrates were stopped at 5, 10, 15, 20, and 30 minutes, using the following procedure:

$[Cp^*RhCl_2]_2$ (3 mol %), Ag_2CO_3 (1 equiv), LiOAc (0.2 equiv), $AgSbF_6$ (0.2 equiv) were weighted in a vial inside a glovebox and dichloroethane (0.15 M) is added. Nitrobenzene (0.2 mmol) and 1-bromo-2-(triisopropylsilyl)acetylene (**2a**, 1 equiv) are then added and the vial is sealed. The reaction mixture is stirred at 110 °C outside the glovebox for the indicated time. After cooling to the room temperature, the reaction mixture is filtrated through celite and bromomesitylene is added. The yield of the mono-alkynylated product was determined by ^{1}H NMR analysis of the crude using bromomesitylene as internal standard.

Table S1. Time and yields for the Rh-catalyzed ortho-alkynylation of **3b** and **3b-*d*$_5$**.

Time (min)	NMR yield 3b (%)	NMR yield 3b-*d*$_5$ (%)
5	1.4	0.22
10	3.2	0.55
15	5.2	0.7
20	6.6	1.2
30	9.5	2.3

For **3b**: y = 1.95x - 0.7 ; R^2 = 0.99.

For **3b-d$_5$**: y = 0.48x - 0.4. R^2 = 0.89

KIE = $k_{3b}/k_{3b\text{-}d5}$ = 1.95/0.48 = 4

One-pot KIE

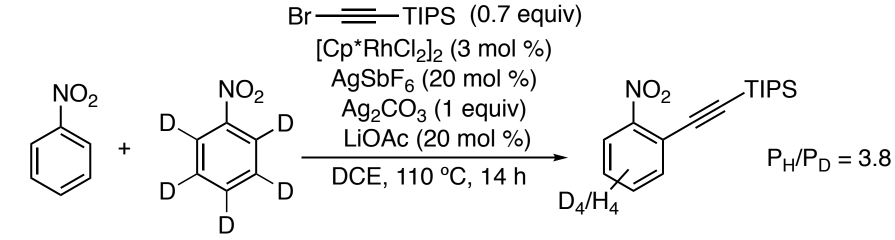

A one-pot competition experiment between hydrogenated and deuterated substrates was also performed, using the following procedure:

$[Cp^*RhCl_2]_2$ (3 mol %), Ag_2CO_3 (1 equiv), LiOAc (0.2 equiv), $AgSbF_6$ (0.2 equiv) were weighted in a vial inside a glovebox and dichloroethane (0.15M) is added. Nitrobenzene-d_5 (0.2 mmol, 1 equiv), nitrobenzene (0.2 mmol, 1 equiv) and 1-bromo-2-(triisopropylsilyl)acetylene (0.7 equiv) are then added and the vial is sealed. The reaction mixture is stirred at 110 °C for 14h. After cooling to the room temperature, the reaction mixture is filtrated through celite and bromomesitylene is added. The yields of the mono-alkynylated products were determined by ^{1}H NMR analysis of the crude using bromomesitylene as internal standard. A ratio of **3b/3b-*d*$_5$** = 3.8 was observed.

Hammett Plot

A Hammett plot study was carried out by measuring the initial rate of the *ortho*-alkynylation of six differently *meta*-substituted nitrobenzenes. Ten identical reactions were run in parallel for each derivative. These reactions were stopped at different times in the first 10-15% of conversion.

Reproducibility issues were prevented by using AgOAc instead of Ag_2CO_3-LiOAc system.

Representing yield *vs* reaction time a straight line was obtained for each derivative. The slope value (k_R) of these straight lines corresponds to the initial reaction rate and can be conveniently used for the Hammett plot analysis.

Table S2. Hammett plot data.

Substituent (R)	k_R	k_R/k_H	Log (k_R/k_H)	σ_p	σ_p^+
Br	0.06	0.0714	-1.13	0.23	0.15
Cl	0.07	0.0833	-1.07	0.23	0.11
F	0.36	0.4286	-0.37	0.06	-0.07
H	0.84 (k_H)	1.0000	0.00	0.00	0.00
Me	2.02	2.4048	0.38	-0.17	-0.31
OMe	4.80	5.7143	0.60	-0.26	-0.78

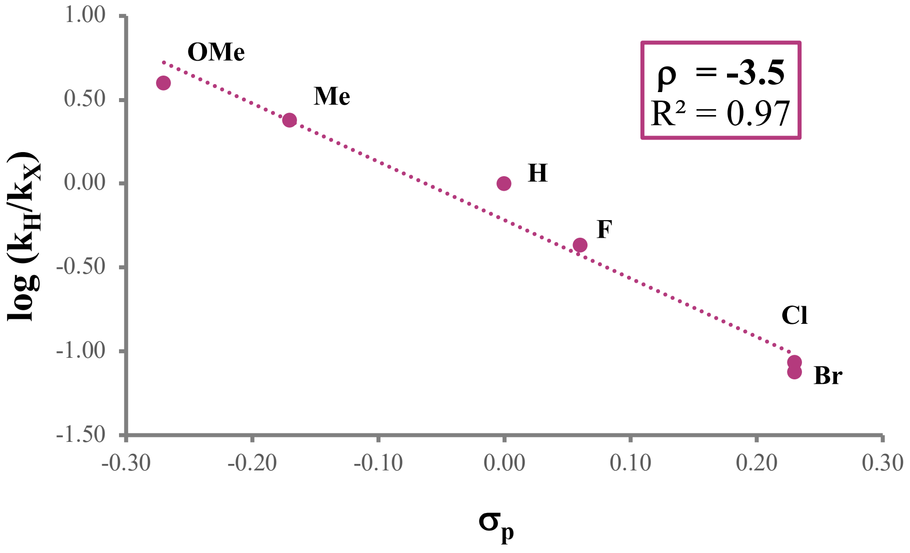

Figure S1. Hammett Plot using σ_p values.

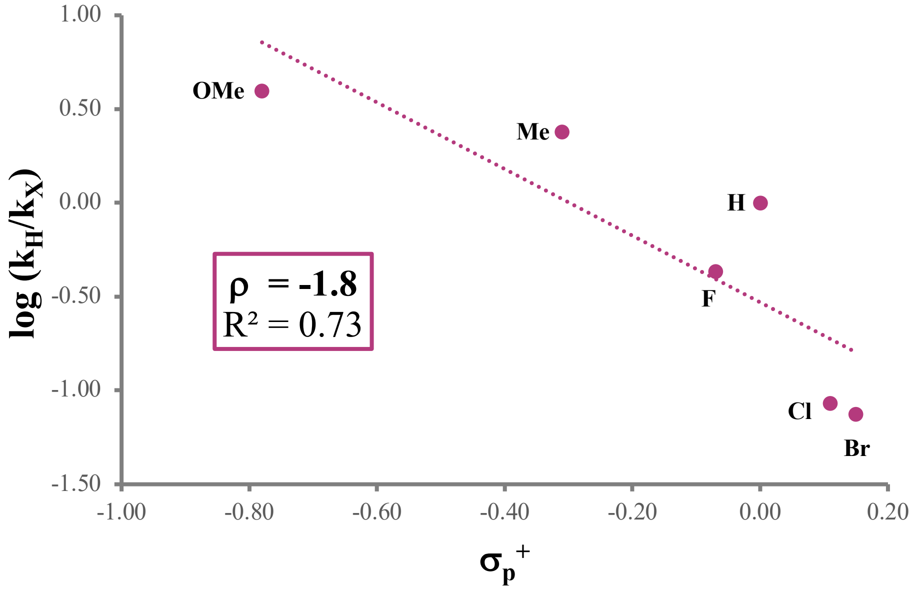

Figure S2. Hammett Plot using σ_P^+ values.

The negative value of the slope illustrates the formation of a partial positive charge in the TS of higher energy. We observe that the correlation with σ_P values fits better the experimental results (R^2 = 0.97), which points in the direction of a relatively low stabilization by resonance of this partial positive charge.

7. DFT Calculations

Computational Details

All density functional calculations were performed with the functional ωB97xD[5] using the Gaussian09 suit.[6] Two different basis set were used. Basis set I: LANL2DZ[7] for Rh, Ag and Br and 6-31G(d)[8] for the remaining atoms. Basis set II: LANL2DZ for Br. The basis set were expanded to LANL2TZ[9] for Rh, Ag and to 6-311++G(d,p)[10] for the rest of the atoms. Using basis set I, all structures were fully optimized and frequency calculations were undertaken. No imaginary frequencies for minima and a single imaginary frequency corresponding to the reaction coordinate in the case of the transition states were found. Additionally, using expanded basis set II, single points of the optimized structures were done to refine the potential energy values. Polarizable Continuum Model (PCM)[11] was used to simulate dichloroethane ($\varepsilon = 10.125$) as solvent throughout all calculations. Unless otherwise stated, all the energies presented are potential (E) and free energies (G) in solution at 298.15 K and 1 atm in kcal/mol. Optimized geometries were visualized using CYLview.[12]

The bonding situation was analyzed using Natural Bond Orbital analysis (NBO 6.0).[13] Charge accumulation in the nitro fragment in the transition states has been calculated using Natural Population Analysis (NPA).[14] The Natural Localized Molecular Orbitals associated to relevant interactions have been determined.[15] The NLMOs isosurface were visualized using ChemCraft, with the isosurface contour set at 0.04.[16]

Plausible Resting States

Unless otherwise stated, the calculations were carried out using 2-methylnitrobenzene **1a** and TIPS-bromoalkyne **2a** as substrates and Cp as ligand at 25ºC and in dichloroethane as solvent.

First, the plausible resting states of the catalytic cycle were computed and their energies were compared (Figure S3). The most stable adduct is **I** in which Rh(III) is coordinated to Cp, AcO^{-} (μ^2) and to the alkyne (η^2), thus, **I** will be considered the resting state of the catalytic cycle for all the computational studies.

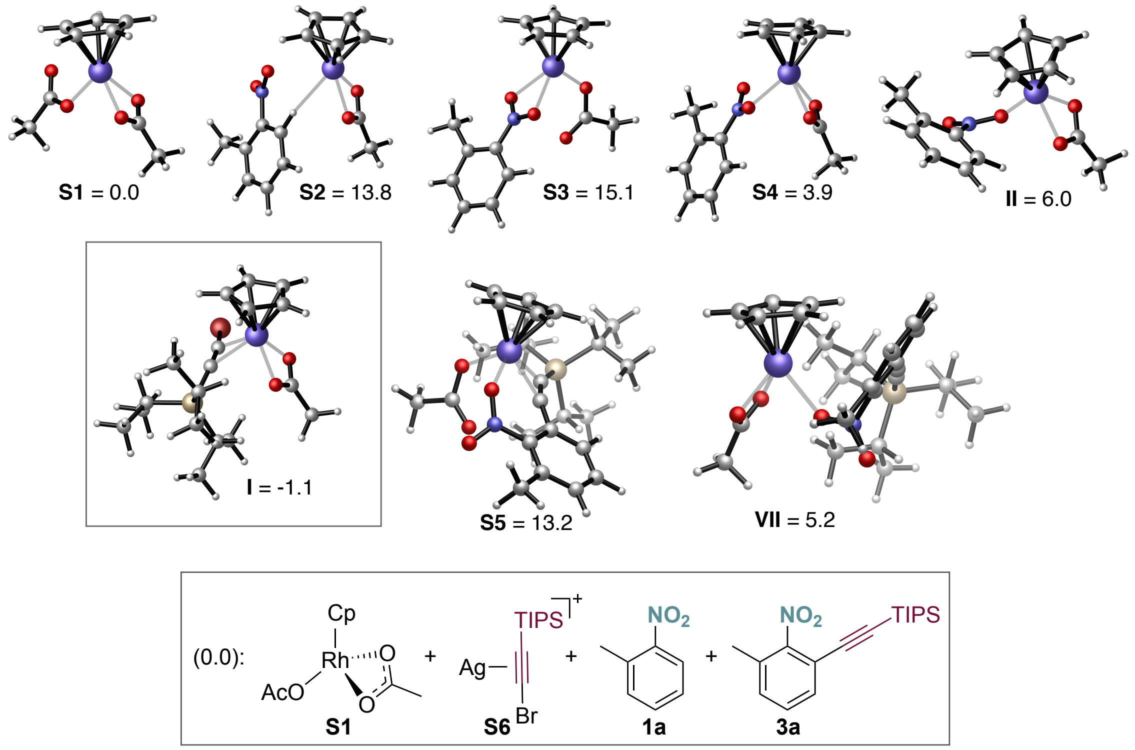

Figure S3. Plausible resting states of the catalytic cycle. Free energies in kcal/mol.

DFT Mechanism for the *ortho*-Alkynylation of 1a

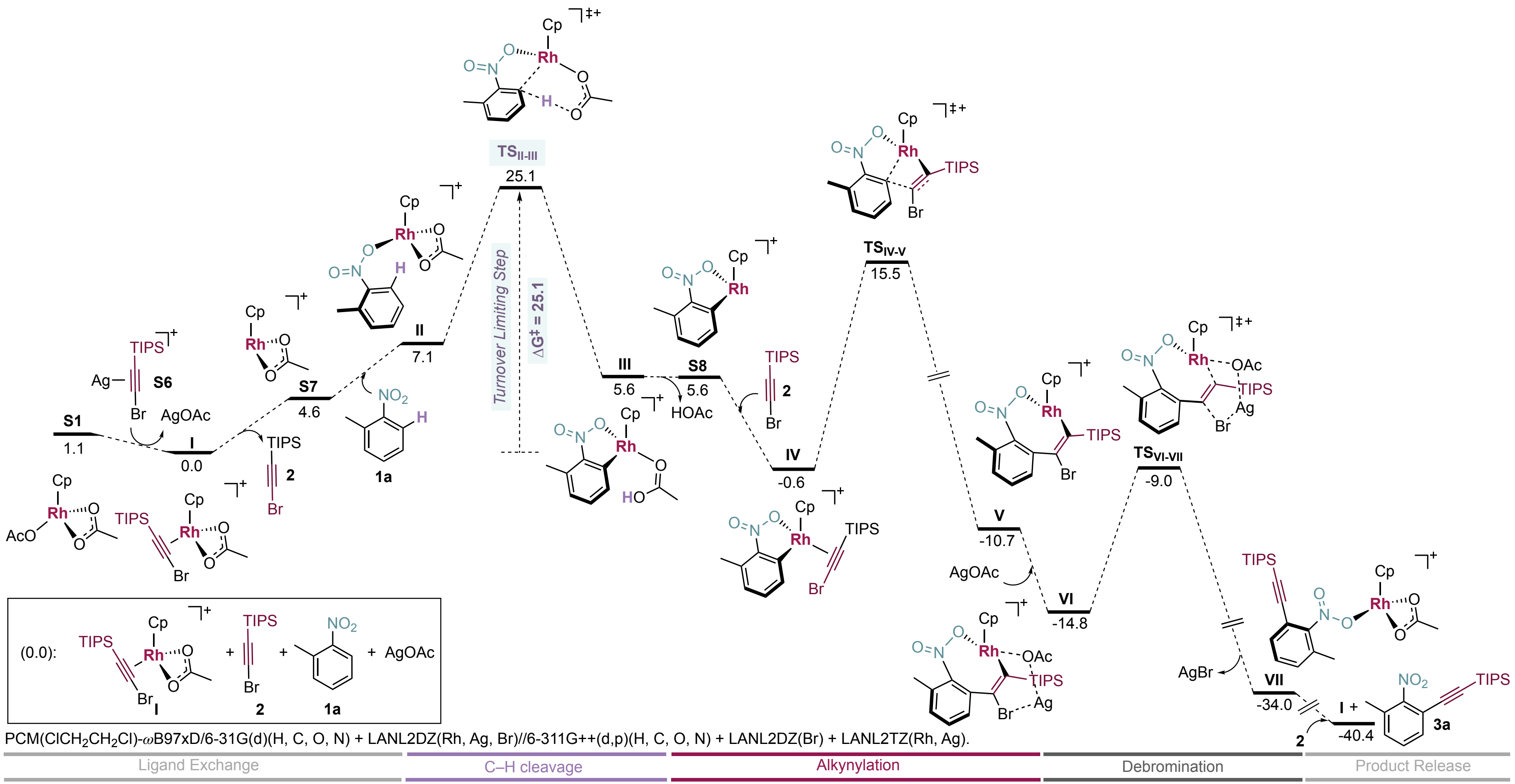

Figure S4. Full free energy profile for the *ortho*-alkynylation of 2-methylnitrobenzene **1a**. Free energies in kcal/mol.

Alternative Transition States for the C–H activation

The lowest activation energy transition state found for the rate limiting C–H activation ($\Delta G^{\ddagger}$ = 25.1 kcal/mol) proceeds through the intramolecular assistance of the acetate ligand in a concerted six-membered cyclic transition state **TS_{II-III}** (Figure S5). The alternative 4-membered cyclic transition state (**TS_{CH-4}**, $\Delta G^{\ddagger}$ = 38.7 kcal/mol) and the intermolecular acetate-assisted transition state (**$TS_{CH-inter}$**, $\Delta G^{\ddagger}$ = 45.4 kcal/mol) were also considered and found to be much higher in energy (Figure S5).

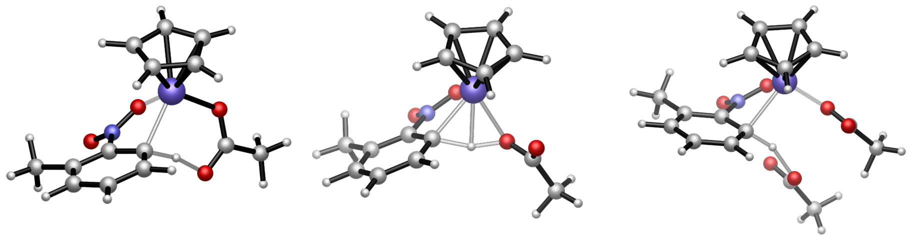

TS_{II-III} ($\Delta G^{\ddagger}$ = 25.1) **TS_{CH-4}** ($\Delta G^{\ddagger}$ = 38.7) **$TS_{CH-Inter}$** ($\Delta G^{\ddagger}$ = 45.4)

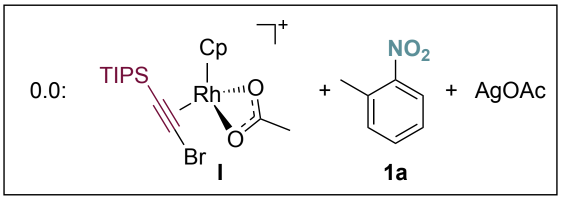

Figure S5. Optimized geometries for the alternative transition states for the C–H activation. Free energies in kcal/mol.

Structural Analysis of Relevant Structures

Table S3. Representative calculated bond distances. C1 represented by a black circle.

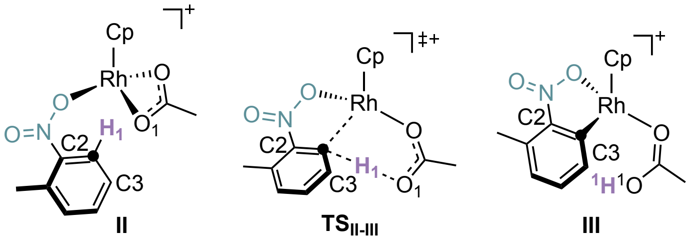

Structure	C1–H1 (Å)	C1–Rh (Å)	O1–H1 (Å)
**II**	1.084	3.350	2.339
**TS$_{II-III}$**	1.277	2.248	1.359
**III**	2.787	2.011	0.974

Table S4. Representative calculated angles.

Structure	C2–C1–C3 (º)	Rh-C1-H1 (º)	C2-C1-H (º)	C3-C1-X (º)
**II**	118.514	-	120.315 (X = H1)	121.169 (X = H1)
**TS$_{II-III}$**	117.366	74.461	116.801 (X = H1)	113.715 (X = H1)
**III**	117.610	-	115.100 (X = Rh)	127.229 (X = Rh)

NBO Analysis – NLMOs

Table S5. Natural localized molecular orbitals (NLMOs) associated to the lone pair over O1 and C1–H1 bond. Contribution of main atoms in percent and NBOs donor-acceptor related to the analyzed NLMO.

Interaction	Analysis	II (X = Rh)	$TS_{II\text{-}III}$ (X = H1)
$\sigma_{O1\text{-}X}$ bond (X = Rh or H1)	NLMO	$2p_y$(O1)	$2p_y$(O1)
	O1	83.9%	86.3%
	H1	0.02%	7.23%
	Rh	7.34%	1.1%
	NBO donor	n_{O1}	n_{O1}
	NBO acceptor	$\Omega^*_{Rh\text{-}C17}$ (47.46 kcal/mol)	$\Omega^*_{C1\text{-}H1}$ (108.32 kcal/mol)
$\sigma_{C1\text{-}H1}$ bond	NLMO	$\Omega_{C1\text{-}H1}$	$\Omega_{C1\text{-}H1}$
	C1	64.2%	65.4%
	H1	34.7%	20.6%
	Rh	0.1%	8.6%
	NBO donor	$\Omega_{C1\text{-}H1}$	$\Omega_{C1\text{-}H1}$
	NBO acceptor	$\Omega^*_{Rh\text{-}C17}$ (0.12 kcal/mol)	$3Cn_{Rh\text{-}C15\text{-}C21}$ (19.65 kcal/mol)

NBO types: n_A = nonbonded lone pair (1 center. valence), n_A^* = unfilled nonbonded (1 center, valence), $\Omega_{A\text{-}B}$ = bond (2 centers, valence), $\Omega^*_{A\text{-}B}$ = antibond (2 centers, valence), 3Cn = 3 filled centers bond.

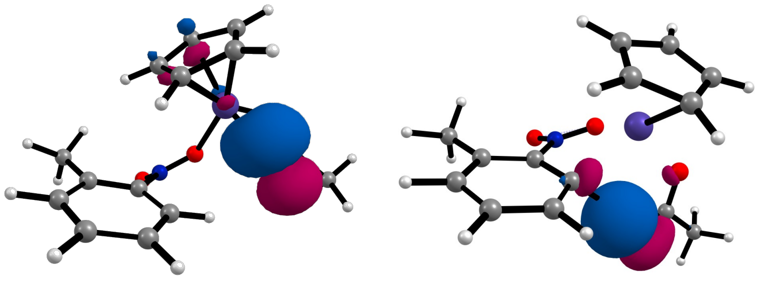

II

$TS_{II\text{-}III}$

Figure S6. NLMOs plot associated to the lone pair over O1 of **II** (left) and **$TS_{II\text{-}III}$** (right).

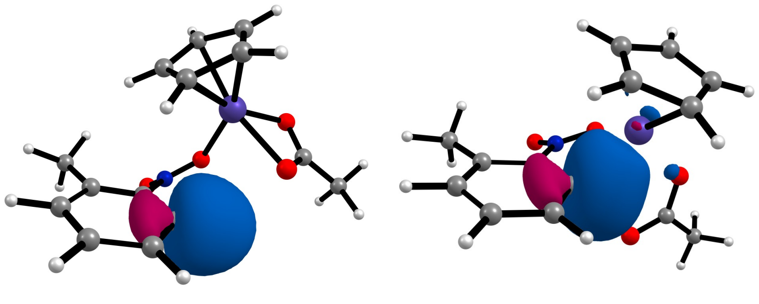

II

$TS_{II\text{-}III}$

Figure S7. NLMOs plots associated to the activated C1–H1 bond of **II** (left) and **$TS_{II\text{-}III}$** (right).

Formation of 2,6-Dialkylynated Nitrobenzenes

For substrates in which one of the ortho positions is not block a second C–H functionalization can take place leading to a 2,6-dialkynylated product. The second C–H activation also occurs *via* intramolecular assistance of the acetate ligand in a concerted six-membered cyclic transition state **TS$_{CH2}$** ($\Delta G^{\ddagger}$ = 26.3 kcal/mol) (Figure S8). As before, the alternative 4-membered cyclic transition state (**TS$_{CH2-4}$**, $\Delta G^{\ddagger}$ = 39.6 kcal/mol) and the intermolecular acetate-assisted transition state (**TS$_{CH2-inter}$**, $\Delta G^{\ddagger}$ = 45.2 kcal/mol) were also considered and found to be much higher in energy (Figure S8).

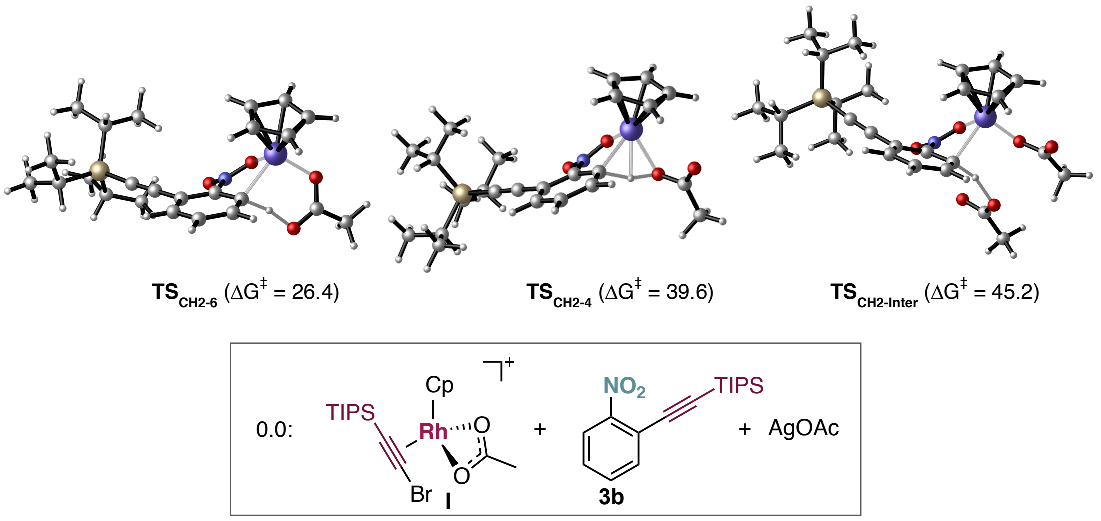

Figure S8. Optimized geometries for the possible transition states for the second C–H activation. Free energies in kcal/mol.

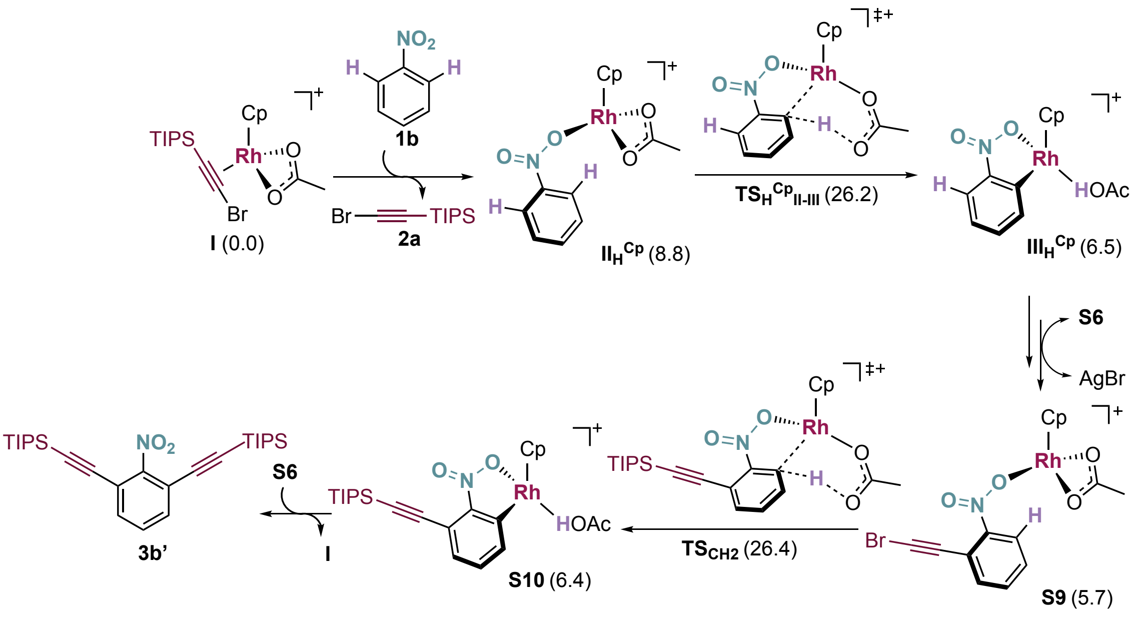

Scheme S1. Simplification of the dialkynylation mechanism of nitrobenzene (**1b**). Free energies in kcal/mol.

Kinetic Isotope Effect

Experimentally, the kinetic isotopic effect between hydrogenated (**1b**) and fully deuterated (**1j-*d*$_5$**) labeled substrates was 4 at 110 ºC (Scheme S2a) indicating that the C–H bond cleavage occurs in the turnover limiting step of the catalytic cycle.

All the frequency calculations needed to compute the KIE were performed at 383.15 K (110 ºC). The KIE was calculated using equation S1:

$$KIE_{DFT} = \frac{k_H}{k_D} = \frac{e^{\frac{-\Delta G_H^\ddagger}{RT}}}{e^{\frac{-\Delta G_D^\ddagger}{RT}}}$$

Equation S1. Equation to calculate KIE from the computed activation energy barriers of the C–H activation steps. Where k_H = rate constant for substrate **1b**, k_D = rate constant for substrate **1b-*d*$_5$**, $\Delta G^\ddagger_H$ = computed free energy change for the C–H activation step (from the zero energy to the TS), $\Delta G^\ddagger_D$ = computed free energy change for the C–D activation step (from the zero energy to the TS), R = ideal gas constant in kcal/K·mol and T = temperature in Kelvin (383.15 K).

The KIE was first computed based on the free energy change between [CpRhAlkyneOAc] **I** (which was found to be the zero energy for all the systems) and $\mathbf{TS_{II\text{-}III}^{X}}$ (X = H or D) at 110 ºC (Scheme S2b).[17] However, the obtained KIE (2.7) was smaller than the experimental value previously obtained (Scheme S2a-b). Gratifyingly, changing Cp by Cp* in the calculations, which is the actual ligand used experimentally, the computed KIE was 4.2 and accurately reproduces the experimental results (Scheme S1c).

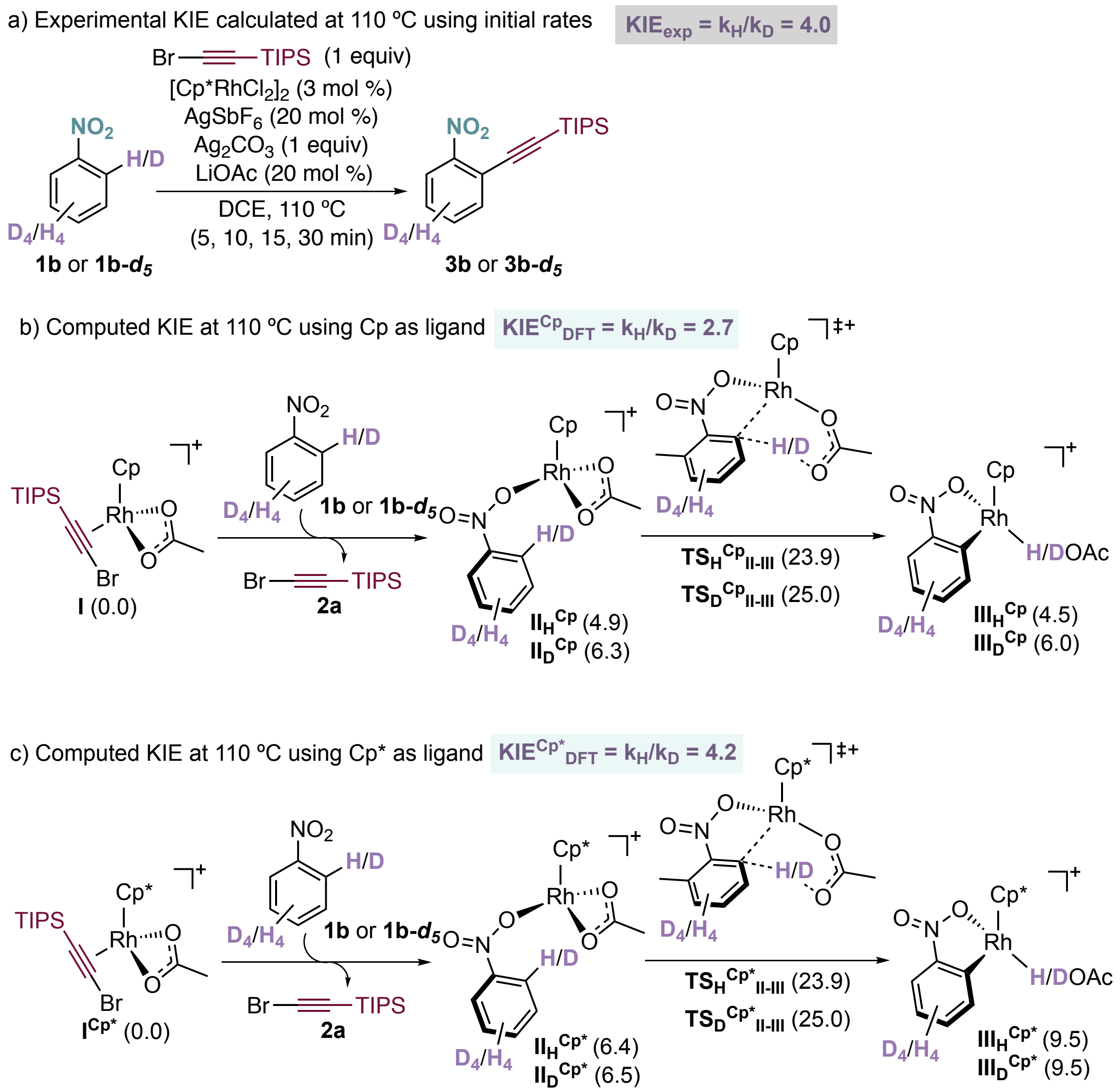

Scheme S2. Experimental and calculated kinetic isotopic effects. Free energies in kcal/mol at 110 ºC.

Computational Correlations with Hammett Parameters

Computational Hammett Plot – Free Energy

A computational Hammett plot was done based on the free energy change between [Cp*RhAlkyneOAc] ($\mathbf{I}^{Cp*}$)[18] and the corresponding $\mathbf{TS_R}^{Cp*}{}_{\mathbf{II\text{-}III}}$ at 25 ºC for a series of *meta*-substituted 2-methyl-nitrobenzene substrates (Table S6). The free energy values were converted to rate constants using the Arrhenius Equation and the corresponding $\log(k_H/k_R)$ were plotted both vs σ_p (Figure S9) and σ_p^+ (Figure S10). For both plots a negative slope is obtained which suggest that a positive charge is being created in the rate determining step. The lineal regression is slightly better using σ_p.

Table S6. Free energy change for the C–H activation step of the following reactions. Free energies in kcal/mol.

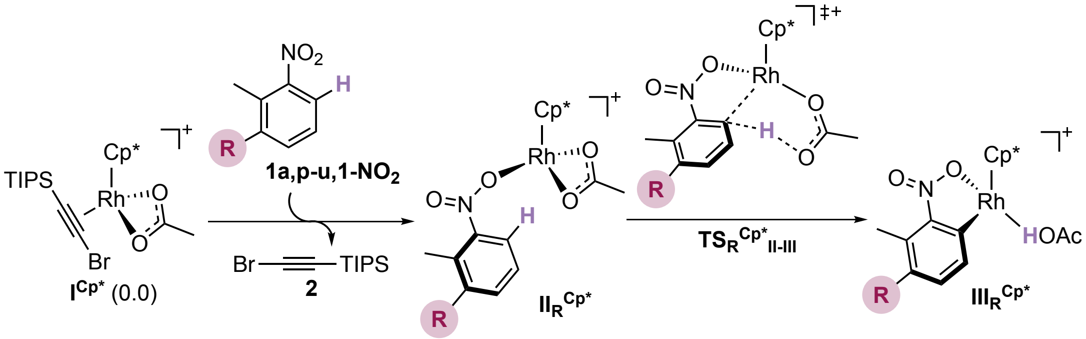

R	σ_p^+	σ_p	$\Delta G^{\ddagger}$($\Delta G(TS_R{}^{Cp*}{}_{II\text{-}III})$ - $\Delta G(I)$)	Log(k_H/k_R)
**NO_2**	0.79	0.81	26.0	-2.5276
**CF_3**	0.61	0.54	23.4	-0.6850
**Br**	0.15	0.23	24.1	-1.1715
**Cl**	0.11	0.23	22.9	-0.3036
**H**	0	0	22.5	0.0000
**F**	-0.07	0.06	22.2	0.2260
**Me**	-0.31	-0.17	22.5	0.0105
**OMe**	-0.78	-0.27	21.0	1.1226

R	$\Delta G(II_R{}^{Cp*})$	$\Delta G(III_R{}^{Cp*})$	$\Delta G(TS_R{}^{Cp*}{}_{II\text{-}III})$
**NO_2**	7.8	9.8	26.0
**CF_3**	5.7	9.9	23.4
**Br**	5.9	10.9	24.1
**Cl**	5.7	9.4	22.9
**H**	4.0	8.9	22.5
**F**	4.3	10.2	22.2
**Me**	4.9	10.9	22.5
**OMe**	4.8	9.9	21.0

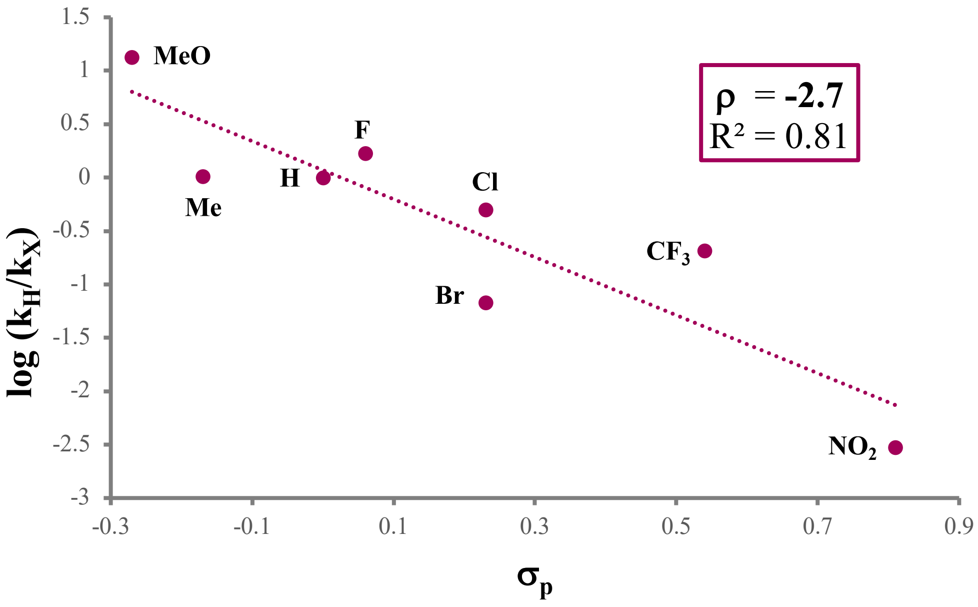

Figure S9. Computed Hammett Plot using σ_P values from free energies.

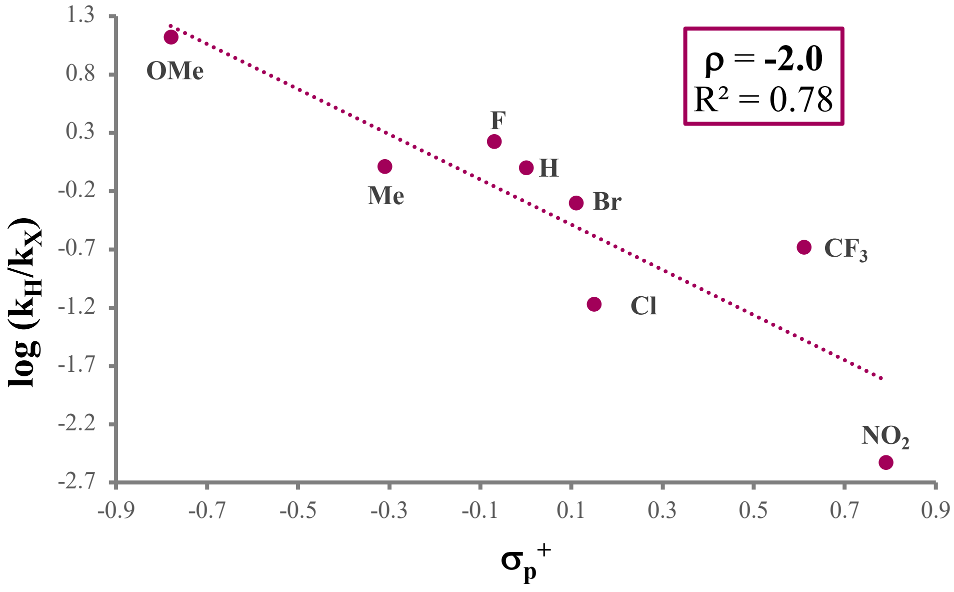

Figure S10. Computed Hammett Plot using σ_P^+ values from free energies.

Computational Hammett Plot – Potential Energy

As the potential energy is less affected by conformational changes than free energy, we also examined the correlation between the Hammett parameters and potential energies.[19] In this case, the computational Hammett plot was done based on the potential energy change between [Cp*RhAlkyneOAc] (**I**^{Cp*})[20] and the corresponding **TS**$_R^{Cp*}$$_{II\text{-}III}$ at 25ºC for a series of *meta*-substituted 2-methyl-nitrobenzene substrates (Table S7). The potential energy values were converted to rate constants using the Arrhenius Equation (assuming than $\Delta G^{\ddagger} = \Delta E^{\ddagger}$) and the corresponding $\log(k_H/k_R)$ were plotted both vs σ_p (Figure S11) and σ_p^+ (Figure S12). For both plots a negative slope is obtained again. which suggest that a positive charge is being created in the rate determining step. The lineal regression are practically the same using σ_p or σ_p^+. Remarkably, the data fits better to a lineal regression than the calculated with free energy changes.

Table S7. Potential energy change for the C–H activation step of the following reactions. Potential energies in kcal/mol.

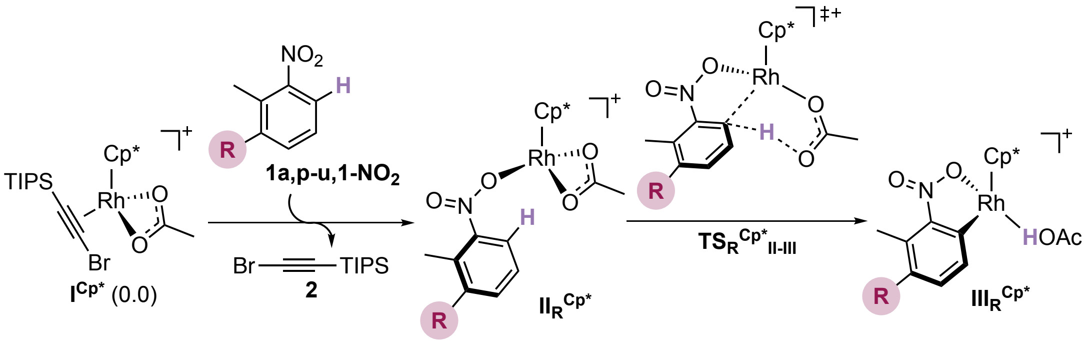

R	σ_p^+	σ_p	$\Delta E^{\ddagger}(\Delta E(TS_R^{Cp*}{}_{II\text{-}III})$ - $\Delta E(I))$	$\log(k_H/k_R)$
**NO₂**	0.79	0.81	28.1	-1.9686
**CF₃**	0.61	0.54	26.4	-0.7512
**Br**	0.15	0.23	26.1	-0.5060
**Cl**	0.11	0.23	26.0	-0.4952
**H**	0	0	25.4	0
**F**	-0.07	0.06	25.6	-0.1370
**Me**	-0.31	-0.17	24.8	0.4509
**OMe**	-0.78	-0.27	23.5	1.3762

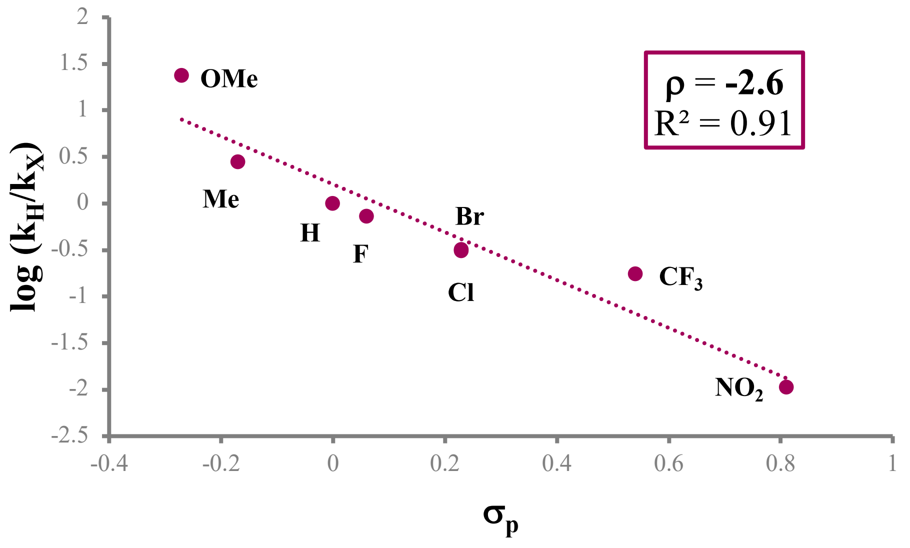

Figure S11. Computed Hammett Plot using σP values from potential energies.

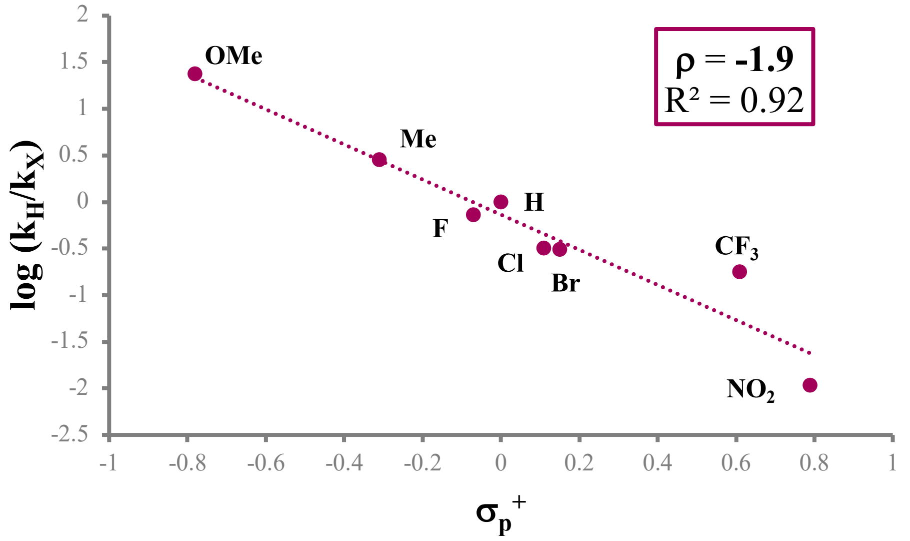

Figure S12. Computed Hammett Plot using σ_P^+ values from potential energies.

Correlation of the Charge Accumulation in the Nitrobenzene versus Hammett Parameters

The charge accumulation on the nitrobenzene fragment ($\Delta\delta^+_{ArH}$) in the concerted transition states (**$TS_R^{Cp*}$$_{II-III}$**) was also assessed by NBO analysis for the representative cases studied for the Hammett plot (Table S8). The sum of the atomic charges, calculated using NPA, on the substituted nitrobenzenes agrees with the trends observed by Hammett analysis suggesting a degree of charge build-up in the substrates. In fact, a straight regression line is obtained by plotting $\Delta\delta^+_{ArH}$ vs σ_p (Figure S13) and σ_p^+ (Figure S14).

Table S8. Charge accumulation on the nitrobenzene fragment ($\Delta\delta^+_{ArH}$) for the corresponding C–H activation transition states. $\Delta\delta^+_{ArH}$ calculated from NPA.

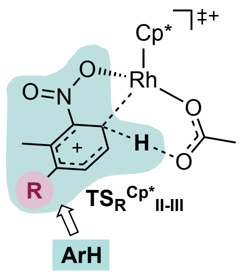

R	σ_p^+	σ_p	$\Delta\delta^+_{ArH}$
**NO_2**	0.79	0.81	0.05744
**CF_3**	0.61	0.54	0.06992
**Br**	0.15	0.23	0.07965
**Cl**	0.11	0.23	0.08369
**H**	0	0	0.10216
**F**	-0.07	0.06	0.09792
**Me**	-0.31	-0.17	0.11585
**OMe**	-0.78	-0.27	0.14196

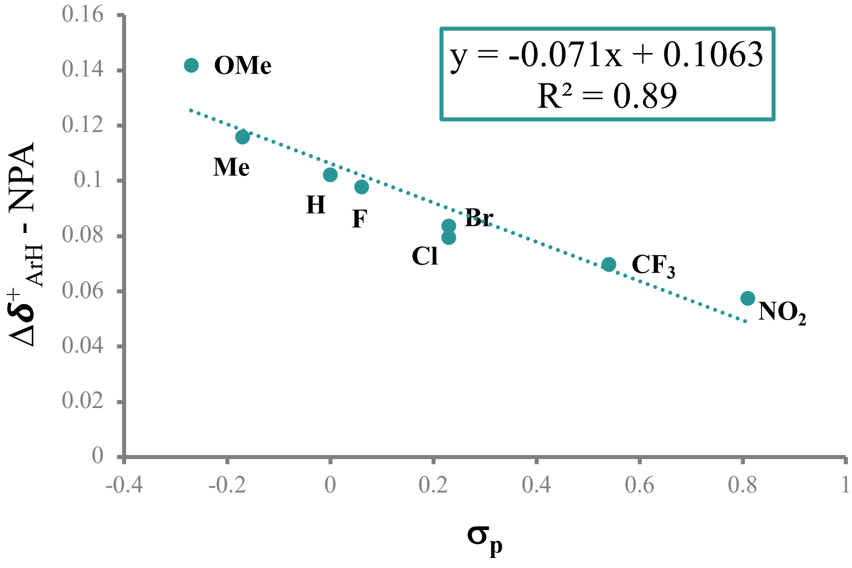

Figure S13. Charge accumulation on the nitrobenzene fragment ($\Delta\delta^+_{ArH}$) vs σ_p values.

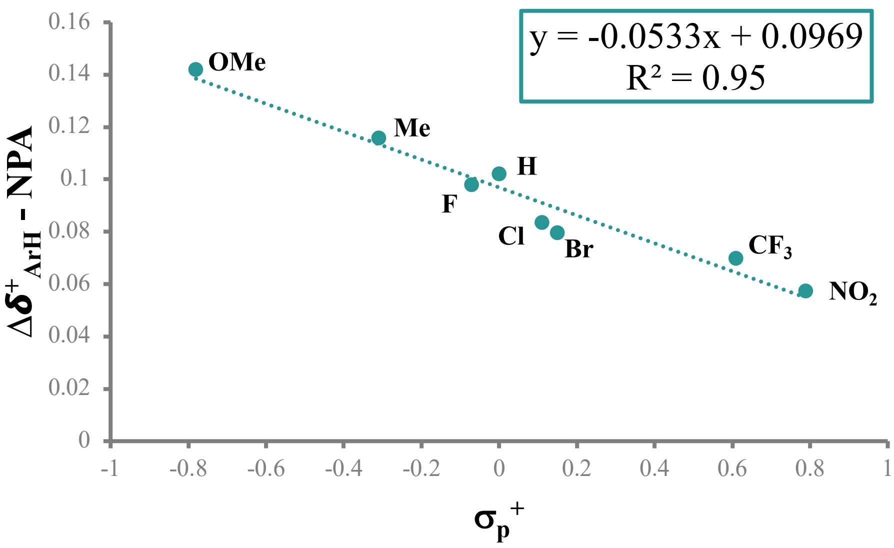

Figure S14. Charge accumulation on the nitrobenzene fragment ($\Delta\delta^{+}_{ArH}$) vs σ_p^{+} values.

DFT Studies on Scope Limitations

Computational Studies on Nitro-Heteroarenes as Substrates

3-Nitropyridene

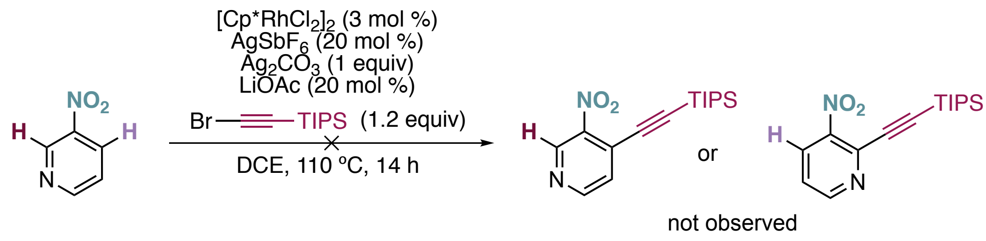

Scheme S3. Unsuccessful Rh-catalyzed *ortho*-alkynylation of 3-nitropyridine.

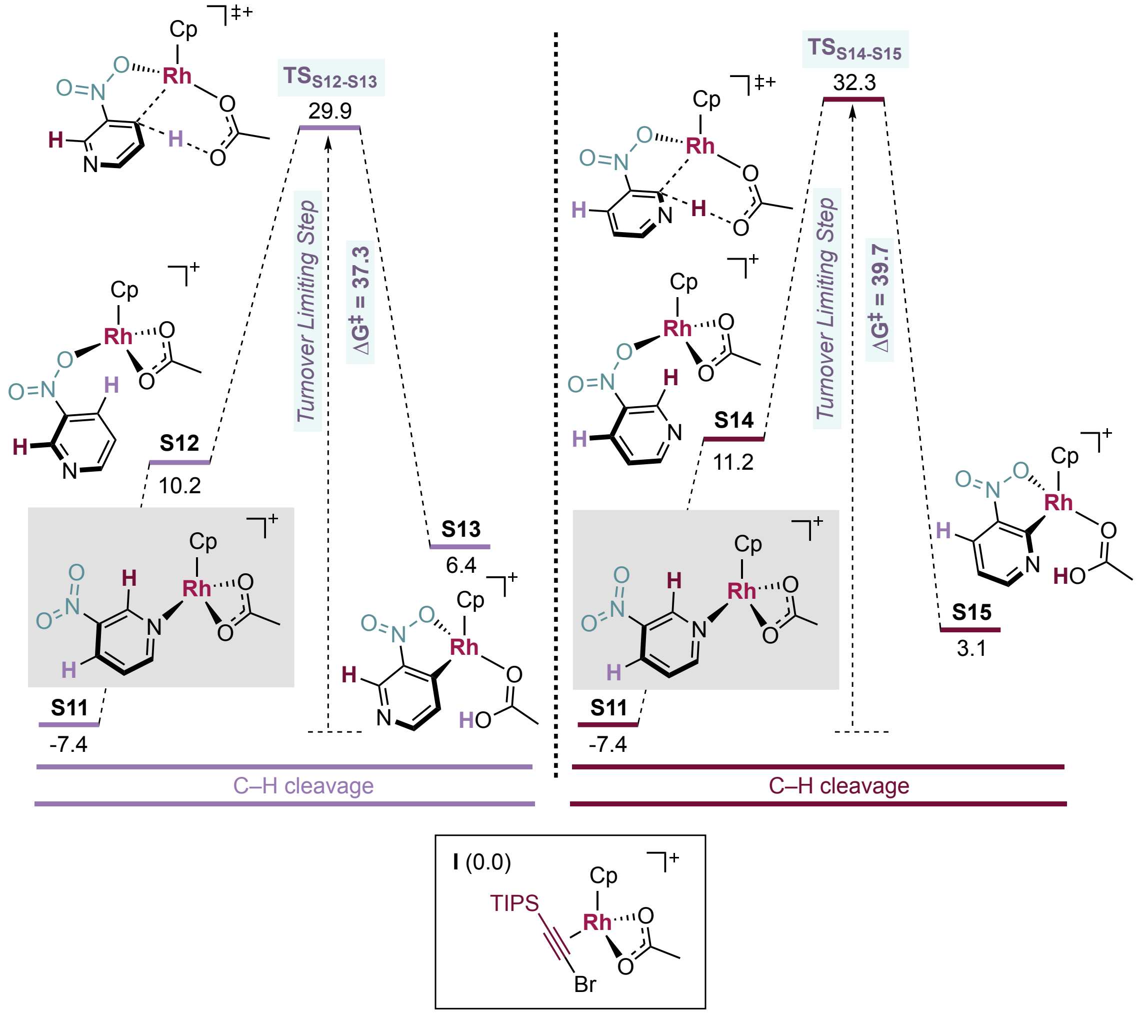

Figure S15. Free energy profile for the plausible Rh-catalyzed *ortho*-C–H metalation of 3-nitropyridene. Energies in kcal/mol at 25 ºC.

Experimentally, the alkynylation of 3-nitropyridene did not provide any alkynylated product while the 3-nitropyridene was recovered untouched (Scheme S3). Computationally, we found that the coordination of the 3-nitropyridene through the N to the Rh center leads to the formation of a highly stable adduct (**S11**, ΔG = -7.4 kcal/mol refered to **I** as ΔG = 0 kcal/mol) (grey square, Figure 1). In addition, the two possible *ortho*-C–H cleavage pathways were computed and the corresponding transition states were found to be pretty high in energy (Figure S15). Therefore, the low-lying energy intermediate **S11** and the high energetic transition states $\mathbf{TS_{S12\text{-}S13}}$ and $\mathbf{TS_{S14\text{-}S15}}$ make the energy span of the two possible ortho-C–H activations of 3-nitropyridene impossible

to overcome at 110 ºC preventing the reaction to occur ($\Delta G^{\ddagger}$ = 37.3 kcal/mol and $\Delta G^{\ddagger}$ = 39.7 kcal/mol).

2-Bromo-5-nitro-thiophene

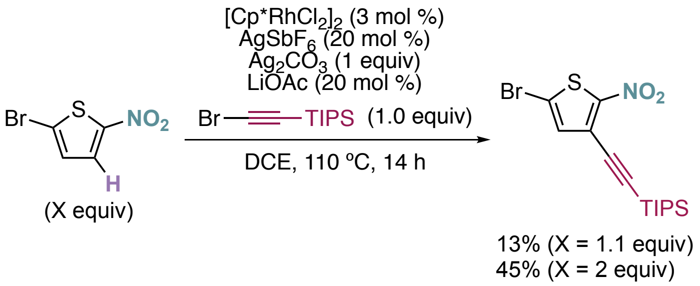

Scheme S4. Rh-catalyzed *ortho*-alkynylation of 2-bromo-5-nitro-thiophene.

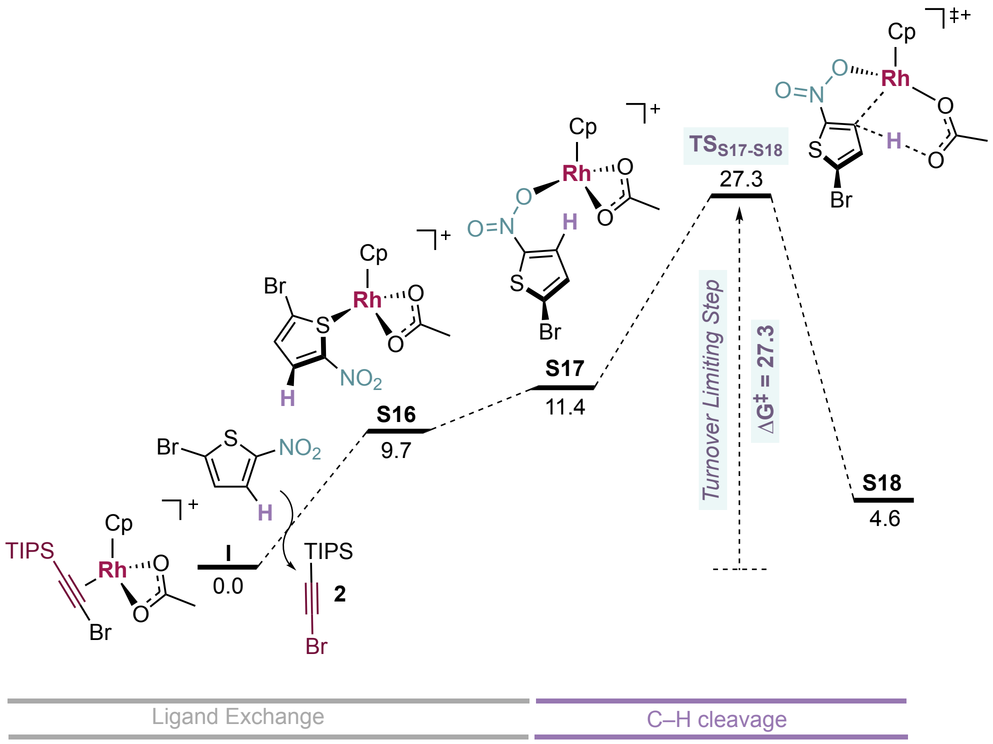

Figure S16. Free energy profile for the Rh-catalyzed *ortho*-C–H metalation of 2-bromo-5-nitro-thiophene. Energies in kcal/mol at 25 ºC.

We also computed the Rh-catalyzed *ortho*-C–H metalation for 2-bromo-5-nitro-thiopene. In this case, the adduct formed by coordination of Rh to the S atom (**S16**, ΔG = 9.7 kcal/mol) is not thermodynamically more stable than the coordination of Rh to alkyne **2** (**I**, ΔG = 0 kcal/mol) (Figure S16). Furthermore, the transition state for the *ortho*-C–H metalation is only 2.2 kcal/mol higher in energy than the one found for the 2-methylnitrobenzene (**1a**). The activation energy for the whole catalytic cycle for 2-bromo-5-nitro-thiopene is 27.3 kcal/mol and, thus, the reaction should be feasible, although less efficient than for nitrobenzene **1a**, at 110 ºC.

Experimentally, when the Rh-catalyzed *ortho*-alkynylation of 2-bromo-5-nitro-thiopene was performed under the same reaction conditions used for nitroarene derivatives (110 ºC, 1 equivalent of nitro-heteroarene) the alkynylated product was obtained only in 13% yield. Gratifyingly, using two equivalents of nitro-heteroarene the yield of the reaction tripled (45%) (Scheme S4).

Computational Studies on Alkyne Counterpart

We next tuned the alkyne counterpart to deeply understand the role of the halogen, the importance of the bulkiness of the Si-protecting group and to reveal the existence or not of a detrimental β-Si-effect.

Halogen Modifications

Experimentally, we found that the reaction worked as efficiently with TIPS-Br-acetylene (**2a**) as with TIPS-Cl-acetylene (**2b**) (Scheme S5). However, TIPS-I-acetylene (**2c**) mainly dimerized under the optimal reaction conditions leading to the formation of **3a** only in a 10% yield (Scheme 3).

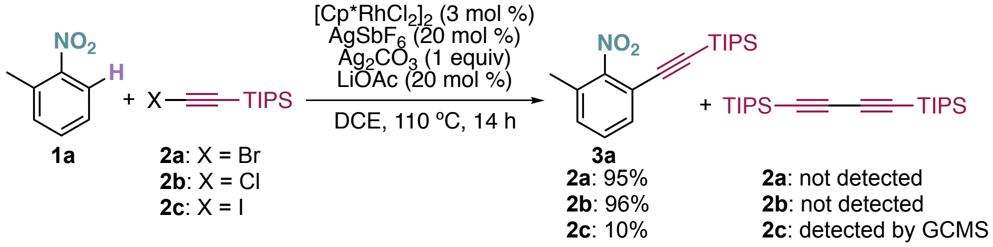

Scheme S5. Rh-catalyzed *ortho*-alkynylation of **1a** using alkynes **2a**, **2b** and **2c**.

To fully understand the experimental results, we computed the free energy profile for the Rh-catalyzed *ortho*-alkynylation of **1a** using alkyne **2b** (Figure S17) and **2c** (Figure S18) and we compared the results with the energy profile already computed for **2a** (Figure S4). We found that the TIPS alkyne migratory insertions are kinetically feasible and thermodynamically favored for the three different alkynes (For **2a**: $\Delta G^{\ddagger}$ = 16.1 kcal/mol; For **2b**: $\Delta G^{\ddagger}$ = 14.5 kcal/mol; For **2c**: $\Delta G^{\ddagger}$ = 16.0 kcal/mol). The Ag-assisted vinyl β-dehalogenation are extremely facile and follow the order of the carbon-halogen bond strengths (**2b** (X = Cl): $\Delta G^{\ddagger}$ = 11.7 kcal/mol > **2b** (X = Br): $\Delta G^{\ddagger}$ = 5.8 kcal/mol > **2c** (X = I): $\Delta G^{\ddagger} \approx$ 0.0 kcal/mol).

Considering the insertion/β-dehalogenation sequence, the alkynylation of **1a** should work with the three TIPS-halogen-acetylene. However, a closer look at the initial adducts formed reveals that the stability of the corresponding $\boldsymbol{\eta}^2$-(alkyne)Rh intermediates (**I**, **S19** and **S23**) alter the final activation energy of the alkynylation process. In particular, for **2c** the energy span is raised to 28.1 kcal/mol and the alkyne dimerization becomes more facile than the C–H metalation of nitrobenzene **1a**. It is also important to consider that Rh-catalyzed alkyne dimerization or other reactions going through oxidative addition of the alkyne may be easier with **2c** than with **2a** and **2b**.

Bulkiness Modification in the Si-Protecting Group

Experimentally, the modification of the Si-protecting group only led to unsuccessful results. Using TES-Br-acetylene **2d** as alkyne counterpart in the Rh-catalyzed alkynylation led to the formation of **3a-TES** only in 10% yield (Scheme S6). Even worse, when less bulky TMS-Br-acetylene **2e** was employed no alkynylated nitrobenzene was detected (Scheme S6). Interestingly, in both cases, the nitroarene **1a** was recovered unreactive and the corresponding alkynes were converted to unknown, undetectable and insoluble products that could not be characterized by NMR or GCMS (they could be trimers or higher polymers).

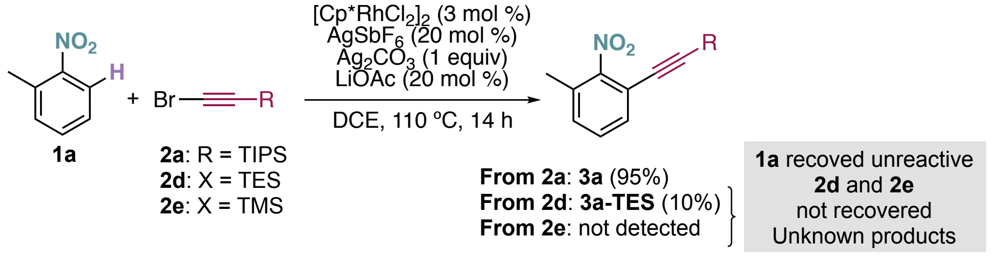

Scheme S6. Rh-catalyzed *ortho*-alkynylation of **1a** using alkynes **2a**, **2d** and **2e**.

Computationally, we focused on the free energy profile of the Rh-catalyzed alkynylation of **1a** using TES-Br-acetylene **2d** since the reaction can occurs experimentally although giving lower yields of **3a-TES** (Figure S19). We found that the activation barrier for the C–H activation ($\Delta G^{\ddagger}$ = 18.0 kcal/mol), as well as for the alkyne migratory insertion ($\Delta G^{\ddagger}$ = 14.0 kcal/mol) and the β-dehalogenation ($\Delta G^{\ddagger} \approx$ 0.0 kcal/mol, barrierless step) are kinetically feasible and thermodynamically favored. In addition, the activation energy of the complete catalytic cycle is even lower than using TIPS-Br-acetlyene **2a** (For **2a**: $\Delta G^{\ddagger}$ = 25.1 kcal/mol; For **2d**: $\Delta G^{\ddagger}$ = 23.5 kcal/mol). This computational data suggests that the bulkiness of the TIPS group is crucial to prevent the consumption of the alkyne before it can engage into the insertion/β-dehalogenation sequence that occurs after the turnover limiting C–H activation step.

TIPS-Cl-Acetylene

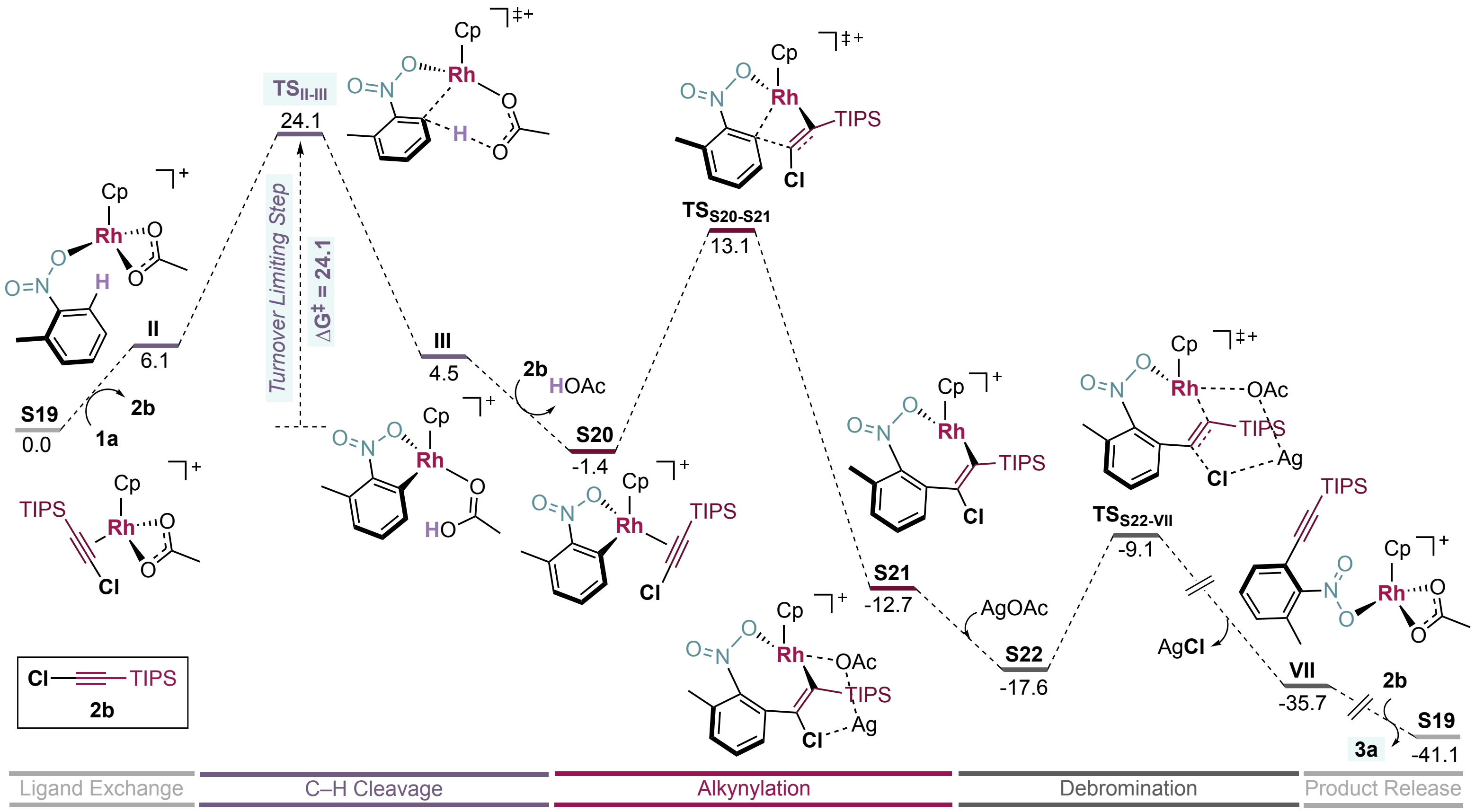

Figure S17. Free energy profile for the Rh-catalyzed *ortho*-alkynylation of 2-methylnitrobenzene (**1a**) using alkyne **2b**. Energies in kcal/mol at 25 ºC.

TIPS-I-Acetylene

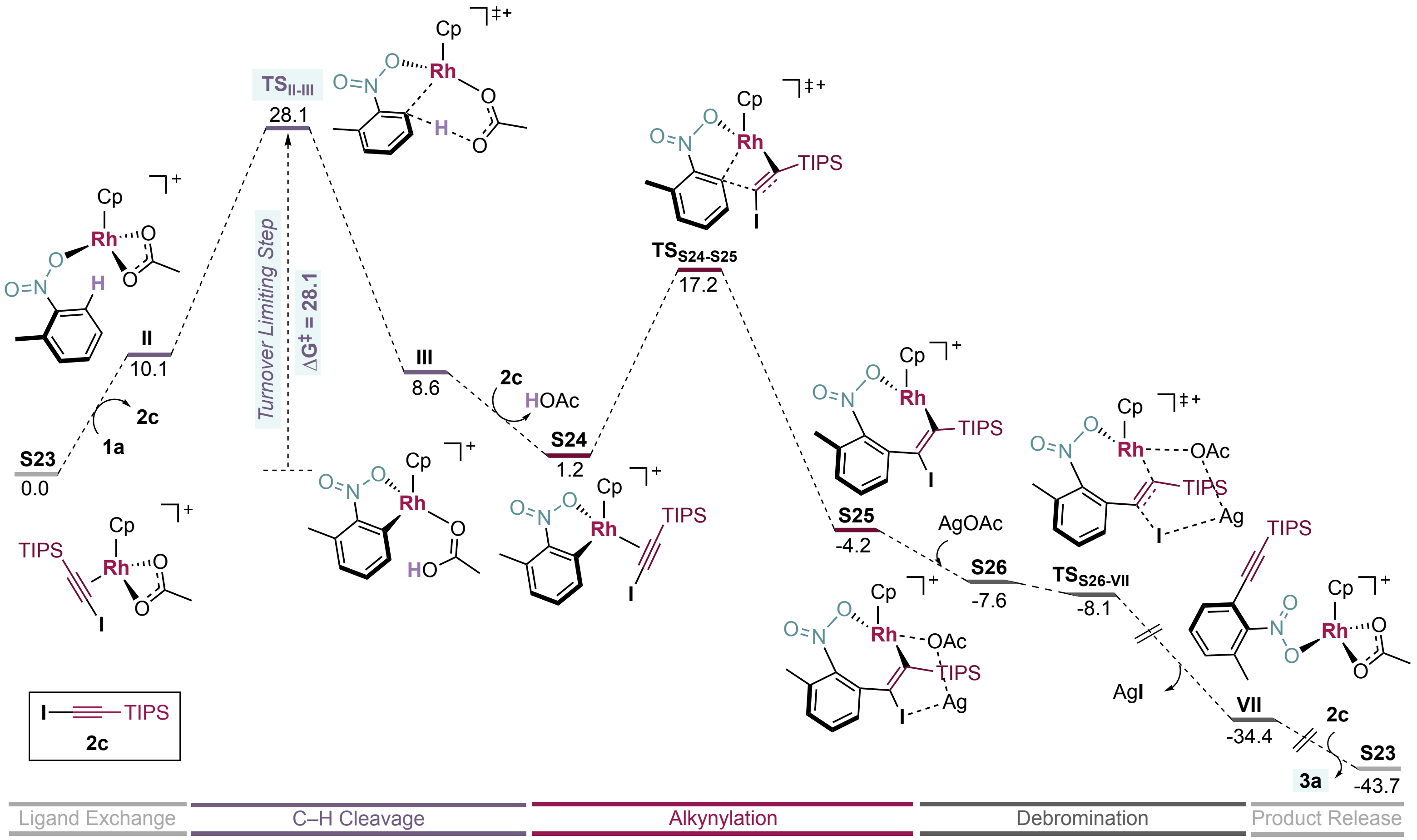

Figure S18. Free energy profile for the Rh-catalyzed *ortho*-alkynylation of 2-methylnitrobenzene (**1a**) using alkyne **2c**. Energies in kcal/mol at 25 ºC.

TES-Br-Acetylene

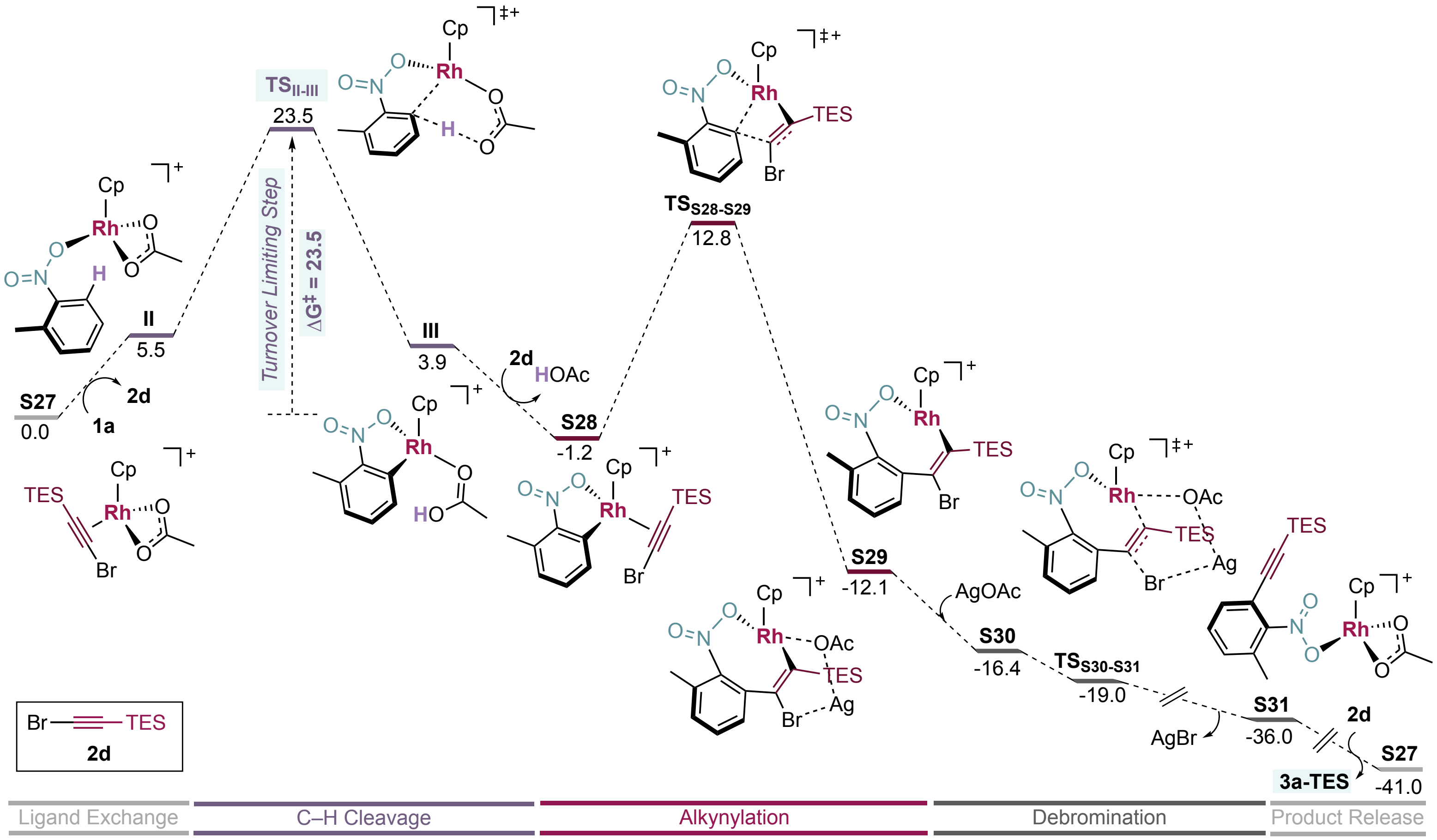

Figure S19. Free energy profile for the Rh-catalyzed *ortho*-alkynylation of 2-methylnitrobenzene (**1a**) using alkyne **2d**. Energies in kcal/mol at 25 ºC.

Ph-Br-Acetylene

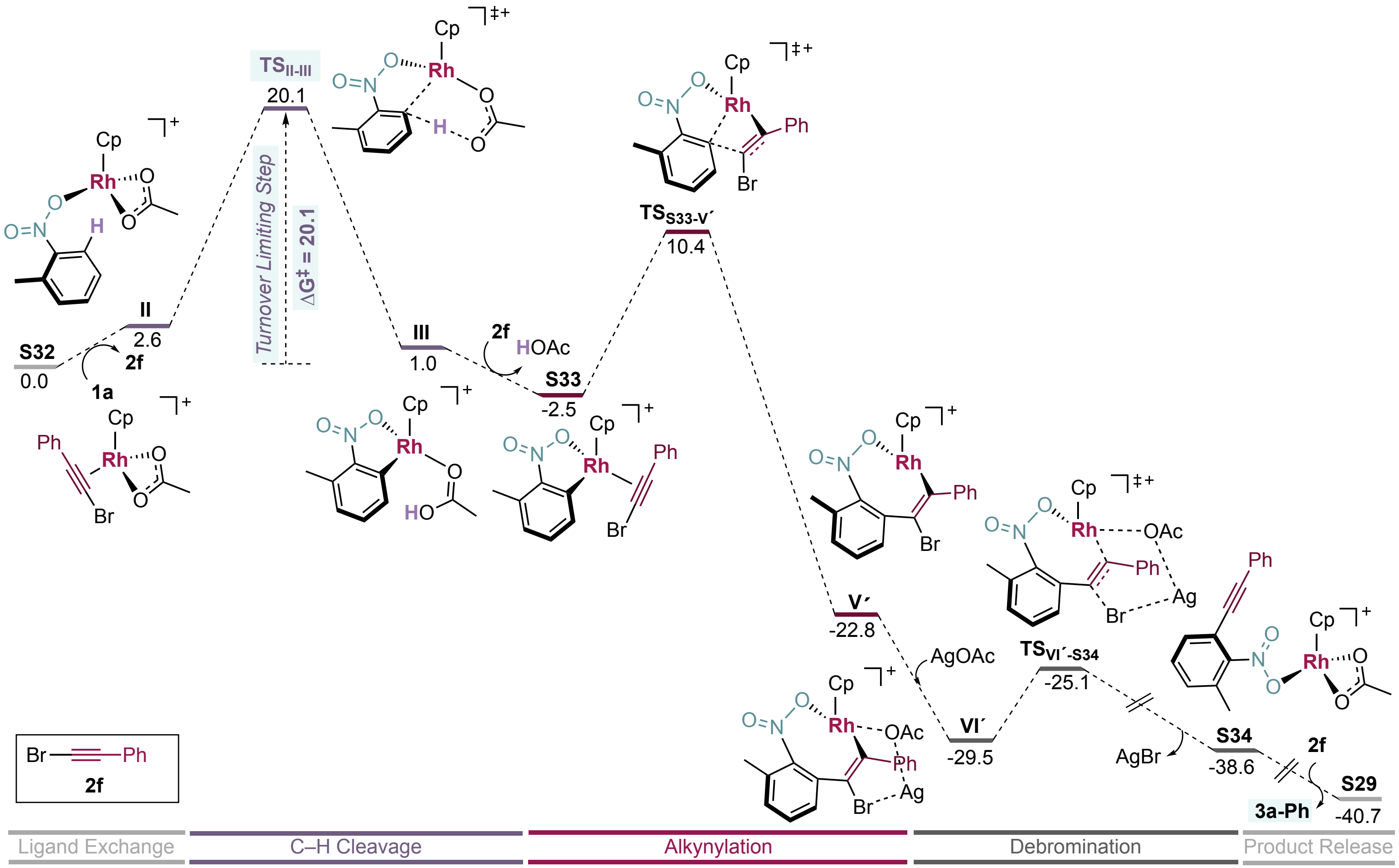

Figure S20. Free energy profile for the Rh-catalyzed *ortho*-alkynylation of 2-methylnitrobenzene (**1a**) using alkyne **2f**. Energies in kcal/mol at 25 ºC.

Non-Si Containing Alkynes

Experimentally the Rh-catalyzed *ortho*-alkynylation of **1a** did not take place with non-Si containing alkyne **2f** (Scheme S7). However, this information is not enough to state that the β-Si effect is essential to facilitate the Ag-assisted debromination.

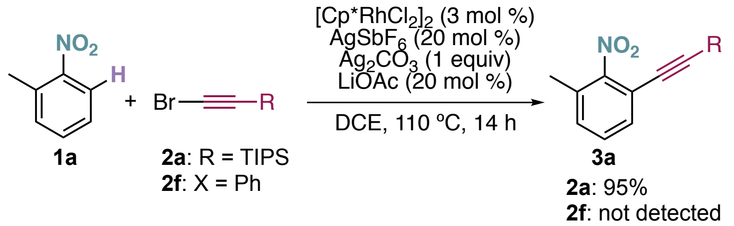

Scheme S7. Rh-catalyzed *ortho*-alkynylation of **1a** using alkynes **2a** and **2f**.

To study the role of the Si atom in detail, we next computed the free energy profile for the Rh-catalyzed *ortho*-alkynylation of **1a** with **2f** (Figure 6). We found that all the elementary steps of the catalytic cycle are energetically accessible under the reaction conditions. In particular, we focused on the Ag-assisted vinyl β-debromination which presents an extremely low activation barrier ($\Delta G^{\ddagger}$ = 4.3 kcal/mol), which suggests that the presence of a Si in β to the Br is not essential for the debromination to happen but, in fact, the bulkiness of the TIPS group is crucial to prevent the consumption of the alkyne counterpart in side reactions.

β-Rhodium effect

Based on the recent work by Musaev and Sarpong on the importance on the β-metal effect on the halogen elimination,[1] we examined the possible hyperconjugative interactions on the previous intermediates to the Ag-assisted debromination for alkyne **2a** (**V** and **VI**) and **2f** (**V´** and **VI´**) (Figure S21). For intermediate **V**, NBO analysis revealed that the σ(Rh-Cα) and σ*(Br-C_β) orbitals are strongly interacting ($E^{(2)}_{ij}$ = 22.7 kcal/mol). A similar interaction was found for intermediate **VI** in which NBO analysis revealed that the σ(Rh-Cα) and σ*(Br-C_β) orbitals are also strongly interacting ($E^{(2)}_{ij}$ = 22.3 kcal/mol). On the other hand, no hyperconjugative interaction was found between σ(Si-Cα) and σ*(Br-C_β). The NBO analysis for the corresponding intermediates for the debromination of non-Si containing alkyne **2f** shows a similar scenario. For intermediate **V´**, a strong interaction between the σ(Rh-Cα) and σ*(Br-C_β) orbitals was also found ($E^{(2)}_{ij}$ = 17.4 kcal/mol) and the same for **VI´** ($E^{(2)}_{ij}$ = 18.4 kcal/mol). Interestingly, no significant interaction was found between Ag filled d orbitals and any orbital associated to the Br atom in any of the two Ag-containing intermediates, **VI** and **VI´** for **2a** and **2f** respectively. All in all, we can conclude that the Ag-assisted debromination is facilitated by this σσ-conjugation arising from the so-called "β-Rhodium effect" rather than by the β-Si effect.

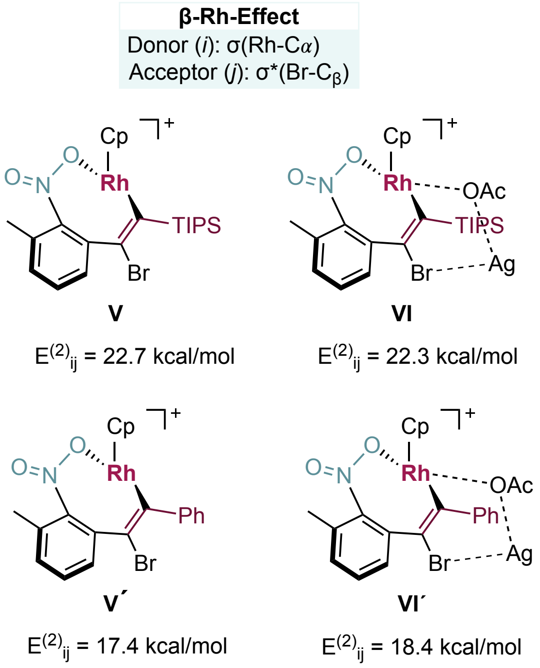

Figure S21. Hyperconjugation across the alkenyl bromide for intermediates **V**, **VI**, **V´** and **VI´**.

It is important to highlight that even if the "β-Rhodium effect" facilitates the debromination, the analysis of the Δd(Br-C_β) in the aforementioned intermediates compared to the corresponding structures in which the CpRh fragment has been changed by a H (Table S9) demonstrated that the main driving force for this process to occur is still the precipitation of AgBr.

Table S9. Analysis of the Br-C_β bond.

[1] a) Usui, K.; Haines, B. E.; Musaev, D. G.; Sarpong, R. *ACS. Catal.* **2018**, *8*, 4516–4527. b) Haines, B. E.; Sarpong, R.; Musaev, D. G. *J. Am. Chem. Soc.* **2018**, *140*, 10612–10618.

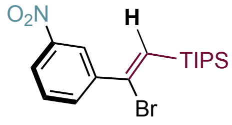

V$_{\mathbf{noRh}}$

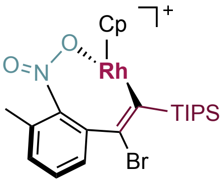

V

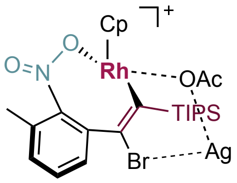

VI

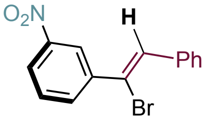

V´$_{\mathbf{noRh}}$

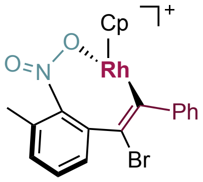

V´

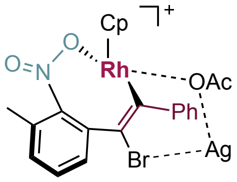

VI´

Structure	d(Br-C$_\beta$) (Å)	Δd(Br-C$_\beta$) (Å) = d(Br-C$_\beta$)$_X$ - d(Br-C$_\beta$)$_{noRh}$
**V**$_{\mathbf{noRh}}$	1.920	0.000
**V**	1.925	0.005
**VI**	1.985	0.065
**V´**$_{\mathbf{noRh}}$	1.933	0.000
**V´**	1.943	0.010
**VI´**	1.999	0.066

Cartesian Coordinates

Cartesian coordinates (Å) and absolute energies (a. u.) for all stationary points from the optimization and frequency calculations using Basis set I. Energies in kcal/mol at 25ºC.

S1

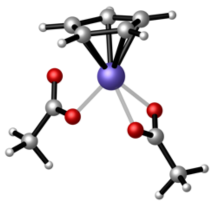

G = -759.704138

E = -759.851301695

O	0.84718000	2.52023500	0.87526800
C	1.55096800	1.95333400	0.04213700
O	1.26554700	0.84214600	-0.56772700
Rh	-0.44633800	-0.17239000	-0.08492800
C	-2.51195900	-0.71446700	-0.48327900
H	-2.78236600	-1.64958100	-0.95497900
C	-2.16411500	0.50468200	-1.17236000
H	-2.17266300	0.65478000	-2.24292300
C	-1.83182500	1.47862800	-0.18863900
H	-1.46977000	2.47869500	-0.37760000
C	-2.35314800	-0.49807000	0.90101700
H	-2.48353700	-1.23698400	1.67979500
C	-1.89707100	0.85761000	1.09012700
H	-1.65244200	1.32372700	2.03335300
C	2.90019000	2.51404000	-0.37381400
H	3.69043200	1.81567500	-0.08234600
H	2.94706100	2.62522900	-1.46069200
H	3.07610300	3.47946600	0.10265700
O	0.65065700	-1.81134200	-0.98790400
C	1.24408300	-2.02242300	0.11266500
O	0.88625400	-1.34554300	1.12646800
C	2.36065600	-3.01348200	0.21290200
H	3.30919100	-2.48539300	0.07169900
H	2.37097600	-3.47548200	1.20158200
H	2.26843600	-3.77566000	-0.56216400

S2

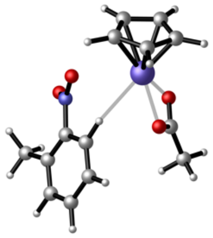

G = -1006.957076

E = -1007.18188286

C	-2.81625900	-1.33866300	1.49066000
H	-2.32697100	-1.67426600	2.39524500
C	-2.74407500	-1.99184900	0.20578300
H	-2.19021600	-2.89469900	-0.01388400
C	-3.45497800	-1.20752500	-0.72753100
H	-3.54159700	-1.39880300	-1.78889100
C	-3.58015700	-0.16156700	1.34903500
H	-3.77560000	0.57121500	2.12009000
C	-3.94663000	-0.03894600	-0.04017100
H	-4.51067300	0.77123800	-0.48291400
C	1.81648000	0.42392200	0.60247200
C	2.26703500	1.61803000	1.13848000
C	3.63971700	1.84403600	1.21877000
C	4.53535700	0.88237900	0.76585800
C	4.11487500	-0.32620100	0.19591800
C	2.72639500	-0.51645000	0.12602200
H	0.75903500	0.20989500	0.53880700
H	1.55271100	2.35652400	1.48546700
H	4.01640700	2.76928300	1.64256100
H	5.60203000	1.06401200	0.85487400
C	5.14932300	-1.31832600	-0.26171300
H	6.12585000	-1.03607900	0.13766400
H	4.91725600	-2.33320300	0.06914700
H	5.21562900	-1.34368900	-1.35232400
N	2.15098500	-1.72433600	-0.46684900
O	1.01915100	-2.05375000	-0.12466300
O	2.80968200	-2.34490300	-1.28688800
C	-0.71914800	1.96702100	-0.93885600
C	0.08041300	3.08710700	-1.50225600
H	1.14092500	2.84946400	-1.36419800
H	-0.11335600	3.19354800	-2.57060300
H	-0.14051400	4.01566000	-0.97454400

Rh	-1.86399900	-0.01474800	0.06633900
O	-1.13594900	1.98376500	0.26604500
O	-0.98262700	0.92440300	-1.62307500

S3

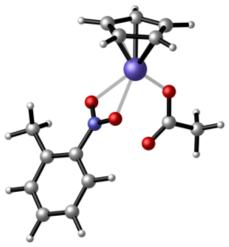

G = -1006.955901

E = -1007.17853786

C	2.40190400	-2.04290100	-0.93390500
H	1.81616400	-2.68783200	-1.57448800
C	2.49375100	-2.12159300	0.46822400
H	1.98495000	-2.83583000	1.10198300
C	3.30409800	-1.01582600	0.93227900
H	3.53220000	-0.78818200	1.96483600
C	3.15003100	-0.88608500	-1.36092300
H	3.23354300	-0.52446600	-2.37738600
C	3.73964100	-0.28483000	-0.20878100
H	4.32493400	0.62436400	-0.19832200
C	-3.03556800	0.24568800	-1.40739400
C	-4.40554200	0.34180100	-1.55258600
C	-5.23381900	-0.14017500	-0.53831100
C	-4.69436800	-0.71193600	0.60817700
C	-3.31562200	-0.82187000	0.80688900
C	-2.51327900	-0.32383600	-0.23947100
H	-2.36287600	0.62558400	-2.16541100
H	-4.82604200	0.79393200	-2.44323000
H	-6.31153600	-0.06815000	-0.64198000
H	-5.35761100	-1.08756800	1.38050400
C	-2.80255100	-1.46174300	2.06555000
H	-3.63208300	-1.93009600	2.59843700
H	-2.04740100	-2.22426300	1.85727000
H	-2.34141900	-0.71958000	2.72281800
N	-1.09065500	-0.36636300	-0.15464200
O	-0.40358900	-0.23346300	-1.17614200
O	-0.51311700	-0.51327600	0.92590000
C	0.55326800	2.56864700	0.33426100
C	0.93599400	3.99234000	0.68098500
H	1.43277400	4.01727000	1.65494800
H	1.64510100	4.37689400	-0.05775200
H	0.04952300	4.62707000	0.70411100

Rh	1.60965000	-0.21768200	-0.06002400
O	-0.60375300	2.24173700	0.11091400
O	1.59452000	1.77187700	0.30524000

S4

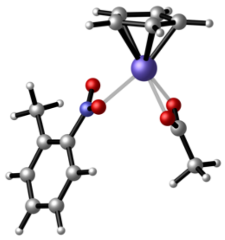

G = -1006.976650

E = -1007.19988665

C	2.60905700	-1.54713800	-1.35924300
H	2.21076500	-1.83530700	-2.32235500
C	2.24469600	-2.11634900	-0.08764000
H	1.51765900	-2.90048600	0.07021300
C	2.95901600	-1.42917500	0.92315200
H	2.85364200	-1.58472200	1.98866900
C	3.55662400	-0.52029900	-1.12122800
H	3.97776400	0.13739600	-1.87030600
C	3.74614800	-0.40239900	0.29589000
H	4.39464500	0.30260200	0.79727100
C	-2.83599300	0.97038700	-0.81251800
C	-4.13391200	1.40250400	-1.01488800
C	-5.19331800	0.60614900	-0.58083900
C	-4.95342400	-0.60807800	0.05183000
C	-3.65944300	-1.07935700	0.29300900
C	-2.61836900	-0.24865900	-0.16182400
H	-1.99154900	1.56666300	-1.13490900
H	-4.31734700	2.35126000	-1.50601200
H	-6.21633000	0.93222900	-0.73798100
H	-5.79111100	-1.21926300	0.37268900
C	-3.47578000	-2.40724400	0.97272000
H	-4.43555200	-2.92423100	1.02357700
H	-2.76498200	-3.04427400	0.43962500
H	-3.09608500	-2.27983100	1.98986900
N	-1.23968400	-0.62349200	0.04513800
O	-0.39996600	-0.08625000	-0.71677500
O	-0.92613400	-1.40174000	0.91708000
C	0.91904700	2.22777600	0.61447600
C	0.30454700	3.52748100	1.00752400
H	-0.77906400	3.45263000	0.86971500
H	0.50029200	3.73239200	2.06139600

H	0.68525600	4.33498200	0.38145200
Rh	1.71014000	-0.02761900	-0.12051900
O	1.42082200	2.04685000	-0.53937700
O	0.92529100	1.23260100	1.40931500

S5

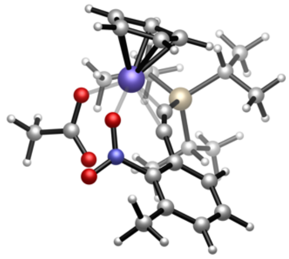

G = -1727.282228

E = -1727.77865837

C	-1.59020500	1.58350700	-0.29171700
C	-3.61771200	3.47115900	-0.59371500
C	-0.46446900	0.70138300	-0.16467200
C	0.72363800	0.35732200	-0.08572500
C	-2.92788300	1.26566000	0.00641100
C	-3.97098700	2.19640500	-0.14013600
C	-1.29128200	2.87787200	-0.72378100
C	-2.30277300	3.81588500	-0.86969500
H	-4.40459800	4.20596600	-0.72793300
H	-2.06349000	4.81888200	-1.20580700
H	-0.25891400	3.13181300	-0.93727000
C	0.97423200	-2.37895800	-1.64655100
C	0.18323600	-1.59454500	-2.57339000
C	0.16281300	-3.46045500	-1.17921000
H	0.51775200	-0.70880400	-3.09663800
H	0.46017500	-4.20057200	-0.44907300
C	-1.11350900	-2.12850300	-2.58483900
H	-1.96447000	-1.72802600	-3.11920600
C	-1.13858700	-3.27422900	-1.69198500
H	-2.00490300	-3.88342000	-1.47092700
H	2.02322000	-2.23427300	-1.42688800
Si	2.58191100	0.65636500	-0.00014200
C	3.51469800	-0.76322800	0.83307200
H	3.70536200	-1.49238100	0.03174700
C	3.08389500	0.83509700	-1.82176500
H	2.71034400	-0.07664300	-2.30968000
C	2.56228200	2.30447800	0.94224000
H	1.79094300	2.89565800	0.42666300
O	-0.47241000	-2.00762900	1.43118500
C	-0.83391900	-1.24274600	2.42603100
O	-1.37703600	-0.14643700	2.33003900
C	-0.52213400	-1.86389700	3.77137800
H	0.55207000	-2.05050000	3.85780600
H	-0.84594400	-1.20056700	4.57405500
H	-1.03220800	-2.82705100	3.86268800
Rh	-0.62771100	-1.50368200	-0.53676600
O	-2.73137400	-1.07290700	-0.04529600
O	-4.16259200	-0.19757300	1.26815400
N	-3.27420800	-0.06929700	0.46288100
C	4.87967200	-0.28889500	1.36402400
C	2.73333500	-1.47513400	1.94444300
C	2.10930700	2.12363400	2.39883100
C	3.87363300	3.09937100	0.86021000
C	4.60390100	0.86606500	-2.04009900
C	2.39495600	2.03453900	-2.48698900
H	5.46654700	-1.14675100	1.71274500
H	5.47671600	0.23327900	0.60990500
H	4.75377100	0.38745100	2.21645100
H	5.08720500	-0.04142100	-1.66418500
H	4.83124100	0.93993900	-3.11045500
H	5.07403400	1.72459400	-1.54886700
H	2.73830700	2.98152400	-2.05532500
H	2.61724200	2.06500800	-3.56023600
H	1.30510600	1.99468700	-2.37655400
H	2.48600500	-0.78600700	2.75964900
H	1.79695900	-1.90638800	1.58339100
H	3.33799300	-2.28351900	2.37394300
H	4.70473100	2.57086400	1.33740400
H	4.16051500	3.31488000	-0.17370000
H	3.76125400	4.06125800	1.37525600
H	1.97716100	3.09888300	2.88251800
H	1.15732500	1.58634100	2.47328900
H	2.85401700	1.56935700	2.98071800
C	-5.42550200	1.90781400	0.12213000
H	-5.73720800	0.93921800	-0.27704500
H	-5.63580900	1.90460400	1.19438400
H	-6.03528100	2.68253700	-0.34619400

S6

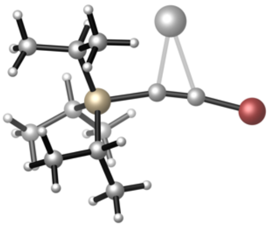

G = -879.732662

E = -879.979124466

Si	-1.44578400	0.16446700	-0.03351500
C	-1.90822000	1.82244300	-0.82511200
C	-1.90820500	-1.34216000	-1.08690600
C	-1.93982000	-0.07565200	1.77972800
H	-1.30898000	1.85875800	-1.74651900
H	-1.31601900	-2.17452000	-0.67125200
H	-1.88699600	-1.16245200	1.94458300
C	-1.49759500	3.01761700	0.04702000
C	-3.38588800	1.91815400	-1.23467600
C	-0.98790400	0.59614900	2.77999500
C	-3.39052000	0.36811200	2.03384700
C	-1.49568500	-1.15589300	-2.55347600
C	-3.38203200	-1.75486600	-0.96072700
H	-1.32085300	0.40702600	3.80738500
H	0.03659400	0.22063900	2.68900900
H	-0.95701900	1.68203000	2.64034500
H	-3.49454100	1.45348800	1.93129900
H	-4.10428000	-0.09926500	1.34805700
H	-3.69408000	0.10646200	3.05433100
H	-3.57816300	2.88631400	-1.71259700
H	-3.66554200	1.14009000	-1.95097700
H	-4.05741900	1.84086300	-0.37342600
H	-1.67522300	3.95861000	-0.48692100
H	-2.07831600	3.05437200	0.97530000
H	-0.43604100	2.98803800	0.31621000
H	-4.06132800	-0.97490800	-1.31916300
H	-3.57572900	-2.65383300	-1.55777900
H	-3.65155800	-1.98540700	0.07481100
H	-0.43246200	-0.90751200	-2.65467400
H	-1.67754600	-2.07273500	-3.12626000
H	-2.06891400	-0.35236200	-3.02944600
C	0.44746100	0.23798800	-0.07173100
C	1.61683100	0.61307500	-0.13761000
Br	3.23407100	1.41189000	-0.25118300
Ag	1.39184400	-1.94591900	0.04058000

S7

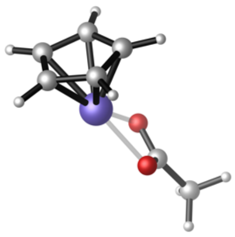

G = -531.150080

E = -531.251341161

C	1.72592900	1.16215300	0.67520400
C	2.24306000	0.72164400	-0.56085500
C	1.35031300	-0.00241300	1.43813300
H	2.54630400	1.35492700	-1.38353200
H	0.90291600	-0.00369300	2.42317600
C	2.24170100	-0.72078300	-0.56349900
H	2.54332200	-1.35156200	-1.38871300
C	1.72425400	-1.16496400	0.67122900
H	1.55036100	-2.19349900	0.95811600
H	1.55303600	2.18988700	0.96557000
O	-1.59434100	1.08532400	0.01876500
C	-2.26286300	0.00026000	0.04622000
O	-1.59298400	-1.08413500	0.01887800
C	-3.74870400	-0.00091000	0.12652400
H	-4.15290900	0.89692600	-0.34279800
H	-4.15147200	-0.89850500	-0.34451000
H	-4.03800300	-0.00221800	1.18249000
Rh	0.20228700	0.00062900	-0.29708200

S8

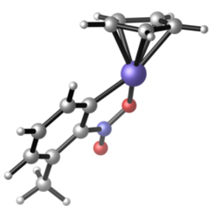

G = -777.980858

E = -778.148951604

C	1.75644200	-0.18112200	-0.00527900
C	0.66945300	0.70262500	-0.10369000
C	3.10572400	0.22036700	0.08142100
C	0.94010200	2.06491200	-0.10988400
C	3.31511000	1.60049200	0.07552700
C	2.26145000	2.50423400	-0.02023300
H	0.13547300	2.78832800	-0.18678400
H	4.33276400	1.97025100	0.14592500
H	2.47348300	3.56910200	-0.02218700
Rh	-1.13228300	-0.15433100	-0.32891800
C	-2.22726700	-0.07202100	1.44877900
H	-1.80420000	-0.37975300	2.39596500
C	-3.10190100	-0.87983400	0.60738800
H	-3.34270700	-1.92033700	0.77663400

C	-3.44216900	-0.12020100	-0.49831200
H	-4.00404300	-0.45675700	-1.35915300
C	-2.17451700	1.25281100	0.89182000
H	-1.65634800	2.10168700	1.31542900
C	-2.81358000	1.19110400	-0.36757700
H	-2.90411800	2.00261500	-1.07713600
O	0.15445800	-1.83765100	-0.12869300
O	2.19326400	-2.46298400	0.05599200
C	4.28485500	-0.70515100	0.16600600
H	4.22841200	-1.34802900	1.04844800
H	5.20474900	-0.11934400	0.21578500
H	4.33830900	-1.36481900	-0.70455500
N	1.38956900	-1.56603700	-0.02303200

S9

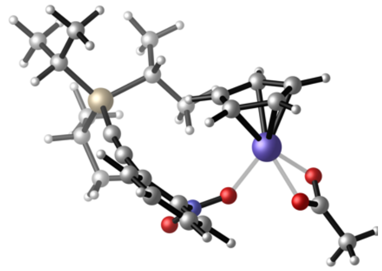

G = -1688.011897

E = -1688.47646000

O	-4.19875500	0.20203700	0.43446700
C	-4.24422800	1.26015700	1.14443500
O	-3.34333600	2.13165600	0.93637100
C	-1.16707100	-2.05902100	0.76924600
C	-2.46396700	-2.53091500	0.64957100
C	-0.08648900	-2.56101500	0.03057700
C	-2.69631800	-3.58713600	-0.22502500
C	-0.35567900	-3.61732600	-0.84736000
C	-1.64485800	-4.12733600	-0.96346200
H	-3.26681100	-2.08033200	1.22197600
H	-3.69930600	-3.98567100	-0.32711000
H	0.45409600	-4.01924100	-1.44563500
H	-1.83207200	-4.94773200	-1.64775900
Rh	-2.36263300	0.82317700	-0.45004300
C	-0.61628900	0.58722500	-1.72128200
H	0.33030500	0.24943000	-1.31798400
C	-1.06282600	1.93101100	-1.77744900
H	-0.50643600	2.80136500	-1.45746300
C	-2.41525000	1.91973000	-2.26769400
H	-3.05461700	2.78292400	-2.39456800
C	-1.67172700	-0.26744700	-2.17169700
H	-1.64028000	-1.34751000	-2.22335200
C	-2.77536400	0.56352100	-2.53217000
H	-3.74542200	0.22127600	-2.86790100
C	-5.28177600	1.45017100	2.19699700
H	-6.07540100	0.71007000	2.09338800
H	-4.80805500	1.33845200	3.17729200
H	-5.69210000	2.46032600	2.13342500
O	-1.50262700	0.13050600	1.50385000
O	-0.16800100	-1.12369800	2.61905500
N	-0.91730100	-0.95468700	1.69111100
C	1.18692600	-1.91648700	0.08088100
C	2.20403200	-1.24901500	0.10606500
Si	3.63490200	-0.05835500	0.11245200
C	4.86678100	-0.72672700	-1.16405300
C	4.37059400	-0.05739400	1.86292800
C	2.83676100	1.57874000	-0.43659100
H	2.31458700	1.31684900	-1.36946000
C	4.31543300	-0.61087600	-2.59212200
H	4.99498900	-1.08241500	-3.31222600
H	3.33860200	-1.09790200	-2.69415100
H	4.19874100	0.43698100	-2.89196200
C	3.84061700	2.68842700	-0.78343000
H	3.31223600	3.57913100	-1.14580900
H	4.42992200	2.99378100	0.08694100
H	4.53881200	2.38160000	-1.56839900
C	1.78446800	2.08327200	0.56220600
H	1.26381200	2.96433300	0.16599600
H	1.02707300	1.32433800	0.78959400
H	2.24593200	2.38064500	1.51038100
C	3.32713000	-0.30293900	2.96311600
H	3.80715800	-0.31129100	3.94933400
H	2.56388600	0.48261700	2.97739300
H	2.81481900	-1.26084700	2.83445300
C	5.17483600	1.21813200	2.16280600
H	4.51659800	2.09154600	2.22926500
H	5.69048000	1.12514400	3.12625300
H	5.93367200	1.42989100	1.40250000
H	5.07186100	-0.90503700	1.87192700
C	6.26735400	-0.10809400	-1.04870900
H	6.70606800	-0.27460400	-0.05952900
H	6.94614100	-0.55276200	-1.78690100
H	6.25429000	0.97218200	-1.22969000
H	4.96059800	-1.79670000	-0.92706000

S10

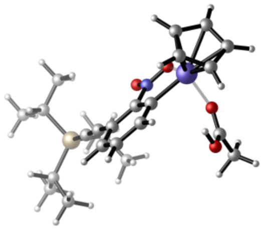

G = -1688.013463

E = -1688.47964934

O	-3.52777100	2.89519700	-0.12847700
C	-2.97896700	2.39748100	-1.21417500
O	-2.92396600	1.18710900	-1.44840200
C	-0.81531900	-0.15350700	0.71917700
C	-2.13331500	0.11021400	1.10986400
C	0.31836600	0.27062900	1.44821900
C	-2.32692800	0.80484800	2.30120600
C	0.07057800	0.96258800	2.63863400
C	-1.23120300	1.21801600	3.05710600
H	-3.85459800	2.17218000	0.43711400
H	-3.33100500	1.03270800	2.64583900
H	0.91437600	1.30101900	3.22887200
H	-1.39214300	1.75330400	3.98741800
Rh	-3.53361900	-0.50002800	-0.20047100
C	-4.55656200	-2.23526800	0.49581500
H	-4.04106800	-3.11372700	0.85989900
C	-5.08278200	-2.05606000	-0.84959200
H	-4.93518700	-2.74052000	-1.67377300
C	-5.69641600	-0.81206200	-0.90808700
H	-6.11791700	-0.34545800	-1.78800400
C	-4.97022000	-1.12044500	1.28289900
H	-4.76779900	-0.97396100	2.33471500
C	-5.57464200	-0.18372500	0.39997500
H	-5.98699200	0.77781600	0.67512300
C	-2.41787000	3.41658700	-2.14229800
H	-3.18915500	4.14925400	-2.39178900
H	-1.60511800	3.94567600	-1.63600900
H	-2.04372700	2.93545600	-3.04377100
O	-1.75361600	-1.27964400	-1.05611700
O	0.38638600	-1.18563900	-0.97827600
N	-0.68303900	-0.90293000	-0.49628200
C	1.67000700	0.07136700	1.04113900
C	2.84755600	-0.02232500	0.74797300
Si	4.59270600	-0.19616500	0.13141700
C	5.09347700	-1.97454500	0.56406500
C	5.66007600	1.06816000	1.06653300
C	4.44270900	0.09248600	-1.73997600
H	3.62358200	-0.57570600	-2.04391200
C	4.30340800	-2.99641700	-0.26562000
H	4.52475400	-4.01949900	0.06204300
H	3.22181700	-2.84598100	-0.17550500
H	4.56015400	-2.93210500	-1.32928900
C	5.68643900	-0.30886200	-2.54658300
H	5.51088200	-0.15387600	-3.61846700
H	6.56305000	0.28705700	-2.27183800
H	5.94220500	-1.36359000	-2.40676400
C	4.01266500	1.52992700	-2.06709500
H	3.81295500	1.64117000	-3.14009700
H	3.10178300	1.81711300	-1.53007900
H	4.79548400	2.25090500	-1.80613000
C	4.91496800	2.36711100	1.40726900
H	5.55505100	3.03296500	1.99933300
H	4.62442500	2.91196200	0.50196200
H	4.00509300	2.17900700	1.98590000
C	6.97647400	1.38142900	0.33779000
H	6.79106800	1.92566500	-0.59487800
H	7.61951800	2.01431300	0.96179800
H	7.54513100	0.47997500	0.08776800
H	5.91136900	0.57071000	2.01538300
C	6.60440100	-2.23508600	0.48306200
H	7.17002200	-1.58282700	1.15652300
H	6.82790400	-3.27121600	0.76633500
H	6.99353400	-2.08650800	-0.53014100
H	4.79713200	-2.09663700	1.61615600

S11

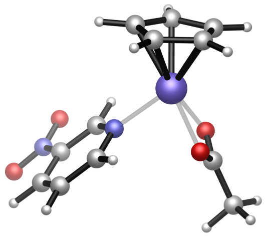

G = -983.750929

E = -983.937227898

O	-1.49402900	1.88913600	0.51913600
C	-1.06792200	2.24143100	-0.62835200
O	-0.85262400	1.32244300	-1.47695300
C	2.96870200	-0.22321900	0.14251600
C	1.64268100	-0.38544900	-0.22123100

C	3.31898400	0.36802100	1.34721500
C	2.28781700	0.78537200	2.17655700
C	0.97551000	0.60327500	1.76720800
H	1.36366300	-0.84347700	-1.16136400
H	2.49217000	1.25393300	3.13103700
H	0.14296800	0.92640900	2.38014100
Rh	-1.35437300	-0.18067700	-0.00762700
C	-1.52891900	-2.30739900	0.38355300
H	-0.66949200	-2.90598900	0.65526200
C	-1.96028800	-2.03053900	-0.93931400
H	-1.51001700	-2.40868400	-1.84690000
C	-3.09621700	-1.14538200	-0.86096200
H	-3.63760800	-0.73179800	-1.70056800
C	-2.35069400	-1.56610000	1.28718700
H	-2.26360500	-1.54937000	2.36446300
C	-3.34048000	-0.87636100	0.50520200
H	-4.08772400	-0.19626500	0.89212800
C	-0.78718500	3.67291100	-0.94048000
H	-1.40581600	4.32499400	-0.32271900
H	0.26605400	3.87504300	-0.71918300
H	-0.96033600	3.86977500	-1.99952800
O	3.64852500	-1.25683000	-1.79579500
O	5.16846200	-0.47769700	-0.46767300
N	4.00806500	-0.68780200	-0.77729600
N	0.66655900	0.02861900	0.59238000
H	4.35953500	0.49439200	1.62055900

S12

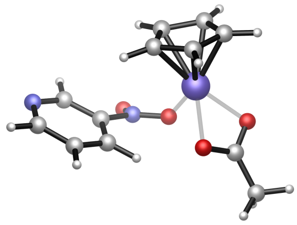

G = -983.724098

E = -983.908090801

O	1.28052400	1.34779300	-1.17271400
C	2.17872400	1.80812800	-0.39348300
O	2.55814800	1.05059300	0.55342700
C	-2.24896100	0.56607900	0.43916600
C	-1.89221200	1.03699500	-0.81971100
C	-3.43928100	-0.12594600	0.64659300
C	-3.94824700	0.07165400	-1.57182500
H	-0.97021700	1.58593300	-0.97884600
H	-4.65821500	-0.14050100	-2.36620600
Rh	1.07696400	-0.40006800	0.04746500

C	0.09482500	-2.26710600	0.54744200
H	-0.74192700	-2.30337200	1.23379300
C	1.46716600	-2.32854000	0.91886500
H	1.85349200	-2.45821200	1.92021700
C	2.24534700	-2.14554400	-0.27309100
H	3.32476700	-2.09989300	-0.32973500
C	0.00739000	-2.03158500	-0.85567000
H	-0.90169700	-1.90373500	-1.42633200
C	1.34396300	-1.96801700	-1.36646000
H	1.62444200	-1.76103700	-2.39076700
C	2.74575900	3.17516400	-0.56423200
H	2.37892200	3.63489100	-1.48160800
H	2.45757300	3.78705300	0.29575600
H	3.83682200	3.11564100	-0.58067900
O	-0.15122600	0.91252700	1.38428200
O	-1.86672000	0.88740800	2.67563300
N	-1.38092300	0.79373200	1.57362800
H	-3.70097800	-0.49558200	1.63379000
N	-4.27563900	-0.38009500	-0.35484100
C	-2.78071000	0.78053600	-1.85167300
H	-2.57882800	1.12896000	-2.85737200

S13

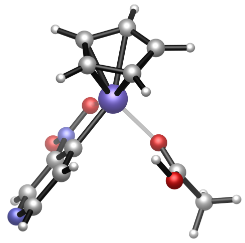

G = -983.731639

E = -983.918836177

O	0.24271500	2.80764400	-1.06012100
C	0.08763500	2.71275400	0.24074300
O	0.33153000	1.68130100	0.87455200
C	-1.95528000	-0.70604000	0.28155400
C	-0.95938800	-0.40047200	-0.64531300
C	-3.30322800	-0.81186500	-0.05633400
C	-2.76865300	-0.33910500	-2.22263200
H	0.57067100	1.96311800	-1.41970500
H	-3.12782600	-0.19945000	-3.23913700
Rh	0.88841300	-0.28249200	0.12813400
C	2.02255600	-2.07066900	-0.14268800
H	1.65119100	-3.03530400	0.17696400
C	2.92580800	-1.21800500	0.61419200
H	3.27767200	-1.41035500	1.61831000
C	3.15647900	-0.07296100	-0.13871900

H	3.71877300	0.79709700	0.17352800
C	1.80120200	-1.47274600	-1.41471500
H	1.20274500	-1.88183500	-2.21680300
C	2.40998500	-0.18413400	-1.38337800
H	2.43440900	0.52862600	-2.19712000
C	-0.40177400	3.95685400	0.89394300
H	-1.33337000	4.27181700	0.41652900
H	-0.55926700	3.78502900	1.95688800
H	0.33470000	4.75163400	0.74534200
O	-0.29673700	-0.83654800	1.86054400
O	-2.32004100	-1.16357100	2.51634200
N	-1.53229200	-0.91724000	1.63475100
H	-4.05008000	-1.04671100	0.69657000
N	-3.70732500	-0.62925900	-1.30680000
C	-1.40817500	-0.21382500	-1.95097300
H	-0.72360200	0.02462800	-2.75768200

S14

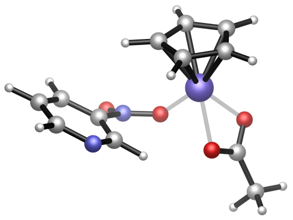

G = -983.723015

E = -983.909383476

O	1.35243800	1.15604300	-1.31610600
C	2.25053100	1.67818300	-0.57672600
O	2.58339900	1.02736500	0.46335500
C	-2.24675600	0.69529600	0.36079200
C	-1.81756500	0.84978900	-0.95402800
C	-3.47099900	0.11164200	0.66187400
C	-4.21674200	-0.35474100	-0.40949000
C	-3.70802500	-0.19781000	-1.69826500
H	-0.88412500	1.35549100	-1.18382500
H	-5.17293100	-0.83924600	-0.25333800
H	-4.26655700	-0.57218200	-2.55154200
Rh	1.05327000	-0.41168300	0.10526100
C	-0.27379800	-2.12963200	0.18877900
H	-1.34467000	-2.04121400	0.31550200
C	0.69262500	-2.20431400	1.25435900
H	0.47422500	-2.19652000	2.31349600
C	1.98139500	-2.26559100	0.66904300
H	2.92162900	-2.27472400	1.20442800
C	0.41990900	-2.11854500	-1.04192600
H	-0.02625500	-2.00536100	-2.02097100
C	1.83101300	-2.16291700	-0.75554000
H	2.63089900	-2.15440100	-1.48360000
C	2.83977500	3.00812500	-0.89258100
H	2.95744000	3.12168600	-1.97155800
H	2.14764500	3.78015800	-0.54051300
H	3.79702100	3.13396500	-0.38622500
O	-0.15722000	1.14378400	1.26838200
O	-1.89689400	1.53412500	2.46630200
N	-1.39314600	1.14830200	1.43781100
N	-2.54487700	0.40367000	-1.97481500
H	-3.80725300	0.00684000	1.68720400

S15

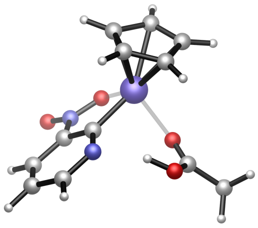

G = -983.739257

E = -983.925435934

O	0.62730300	2.84206100	-0.46753400
C	0.93643200	2.49418500	0.75444300
O	0.83549200	1.34403700	1.20755800
C	-2.15050000	-0.44764500	0.16792700
C	-1.05994700	0.11463100	-0.49524800
C	-3.46366400	-0.12291500	-0.14515400
C	-3.64002700	0.81790700	-1.14870600
C	-2.51399800	1.40246300	-1.72661800
H	0.13132900	2.14068000	-0.96935700
H	-4.63159500	1.12752300	-1.45460000
H	-2.62291800	2.18811400	-2.46800500
Rh	0.73240400	-0.50330900	0.08539700
C	1.29544400	-2.24943400	-1.01004900
H	0.64715900	-3.10735500	-1.12676200
C	2.36474600	-2.12320400	-0.02538400
H	2.56388000	-2.82881700	0.76973400
C	2.99092600	-0.90258800	-0.21804700
H	3.77223200	-0.47483800	0.39501200
C	1.36698300	-1.12616900	-1.88157000
H	0.73327200	-0.93228600	-2.73597200
C	2.32204300	-0.22797900	-1.32468500
H	2.59134400	0.73935200	-1.72775700
C	1.47020100	3.60458800	1.59495000
H	0.88996400	4.51521200	1.43876500
H	1.46711700	3.31650800	2.64512700

H	2.50063900	3.80368100	1.28217500
O	-0.60796600	-1.41718400	1.55436000
O	-2.68534500	-1.88316600	1.89339400
N	-1.82750200	-1.31168500	1.26439600
N	-1.25406600	1.06089500	-1.41124600
H	-4.29733200	-0.57235700	0.38332900

S16

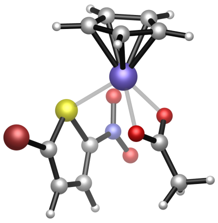

G = -1301.053236

E = -1301.20511591

O	-0.18659600	-0.07255600	1.91314300
C	-0.49611200	1.16156100	1.91673700
O	-1.27897100	1.55495300	0.99059500
Rh	-1.28423600	-0.38529600	0.11271300
C	-2.39312900	-1.13852500	-1.58621100
H	-2.22042000	-0.80477500	-2.60066900
C	-3.29003800	-0.55288400	-0.66127600
H	-3.89137500	0.32950600	-0.83675700
C	-3.15854100	-1.24654900	0.59103400
H	-3.69619300	-1.02700700	1.50349900
C	-1.72714100	-2.23789400	-0.92928300
H	-0.97810100	-2.88347000	-1.36829900
C	-2.21936800	-2.31668000	0.39619300
H	-1.87508000	-3.00174200	1.15977800
C	0.02638100	2.10741500	2.94027400
H	0.93911400	1.71662800	3.39070500
H	0.20424900	3.08274800	2.48369900
H	-0.73074300	2.22975800	3.72167000
O	-0.62301500	2.51443100	-1.97148400
O	0.47396100	4.02303300	-0.85358200
N	0.27626600	2.87833100	-1.22157200
C	1.15870700	1.86070800	-0.74031500
C	2.23787300	2.00872500	0.06260600
C	2.83949900	0.74866000	0.38863800
C	2.18088600	-0.29675700	-0.17022500
S	0.82948900	0.20432300	-1.16899300
H	2.59632600	2.96621100	0.41893200
H	3.71579600	0.64852500	1.01608300
Br	2.58526300	-2.11660300	-0.01669200

S17

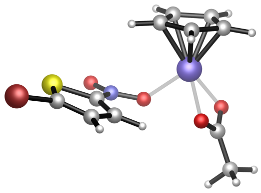

G = -1301.052876

E = -1301.20500020

O	-1.96343600	-1.53326300	-1.12661400
C	-2.93881200	-2.00854000	-0.45369800
O	-3.56203000	-1.19671000	0.29929600
C	1.16172000	-0.42414800	0.96794700
C	1.08609800	-1.03353700	-0.25927200
C	3.27195200	-0.30293700	-0.20894200
C	2.31715900	-0.95886400	-0.94560300
H	0.19198900	-1.51248600	-0.64364500
H	2.48882000	-1.37324400	-1.93031200
Rh	-2.15581200	0.34540800	-0.13650100
C	-2.02859700	2.38413300	0.56015100
H	-1.87210000	2.63411800	1.60110000
C	-3.28048600	2.17753200	-0.06336400
H	-4.24611800	2.22545700	0.42193900
C	-3.03971100	1.75731000	-1.41836500
H	-3.79211800	1.49988600	-2.15089300
C	-0.99501600	2.14197200	-0.41517100
H	0.07093400	2.18842700	-0.23719900
C	-1.61750200	1.78427800	-1.63395400
H	-1.11085000	1.48702200	-2.54268600
C	-3.29411700	-3.45276900	-0.51436900
H	-2.99992500	-3.87777600	-1.47478300
H	-2.74918700	-3.97292900	0.28056500
H	-4.36302900	-3.58772900	-0.34359600
O	-1.03462900	-0.65508600	1.59209200
O	0.39272900	0.08867200	3.03343800
N	0.12497500	-0.31330800	1.91839900
S	2.72249300	0.22953400	1.33554500
Br	5.04117800	0.03574300	-0.73466600

S18

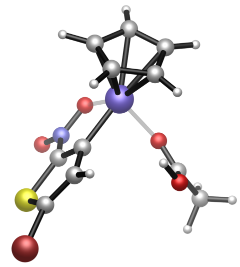

G = -1301.064390

E = -1301.21436717

O	0.57627500	2.44081100	-1.57592400
C	1.15513500	2.67816200	-0.42013700
O	1.67830700	1.79526300	0.26704900
C	-0.74092800	-0.24897400	1.36728600
C	-0.29720300	-0.32881700	0.06930400
C	-2.59676700	-0.27807400	-0.15349400
C	-1.40009000	-0.35183300	-0.82280800
H	0.62420700	1.48922800	-1.78261300
H	-1.32504300	-0.41580100	-1.90095000
Rh	1.70524200	-0.34407700	-0.06434800
C	2.38943600	-2.33196400	-0.42739400
H	2.05088300	-3.18331700	0.14735900
C	3.61777000	-1.58763300	-0.19866600
H	4.29362300	-1.73699900	0.63255100
C	3.70002800	-0.58238400	-1.15621400
H	4.45488900	0.19027500	-1.21148400
C	1.78510200	-1.82850000	-1.61557900
H	0.87960100	-2.19752700	-2.07687100
C	2.52976300	-0.68050200	-2.01353400
H	2.32361400	-0.05848300	-2.87493100
C	1.12646000	4.10457400	0.00142100
H	0.08540400	4.42669200	0.09323300
H	1.64084800	4.22441900	0.95288800
H	1.59938300	4.72037100	-0.76782200
O	1.39533800	-0.29902400	2.09553800
O	-0.17954900	-0.09515100	3.57513000
N	0.17203500	-0.20854800	2.41901800
S	-2.46033400	-0.18798700	1.56997400
Br	-4.30221100	-0.27506500	-0.94411100

S19

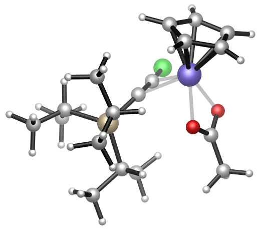

G = -1712.246188

E = -1712.62724020

C	-0.45889600	-0.85412900	1.60408000
C	0.31216200	-0.24104900	0.85985000
C	-2.20139800	2.27414000	-0.42727900
C	-2.12655000	2.03773900	0.96594200
C	-3.25124500	1.44498500	-0.96478000
H	-1.41238900	2.47152300	1.65240200
H	-3.54658200	1.38420700	-2.00331300
C	-3.12019100	1.06005200	1.30771200
H	-3.31313300	0.66488700	2.29515700
C	-3.83753900	0.73283400	0.11850600
H	-4.61949700	-0.01116300	0.03847200
H	-1.54754200	2.91995100	-0.99809500
Si	1.93004100	0.26993500	0.02737900
C	2.44815100	-1.23046700	-1.01556200
H	1.90534100	-1.09902400	-1.96196000
C	1.57122900	1.79501200	-1.03260100
H	0.63349900	1.55270400	-1.54945900
C	3.03810000	0.64696700	1.52064700
H	2.45358100	1.36009900	2.12034200
O	-0.70732600	-0.48658700	-1.86945700
C	-1.15951900	-1.66426900	-1.70751700
O	-1.93969900	-1.84366700	-0.71758500
C	-0.81568300	-2.77294100	-2.64117700
H	0.16025600	-2.59772400	-3.09617500
H	-0.82894000	-3.72830600	-2.11525200
H	-1.56989300	-2.80272600	-3.43428300
Rh	-1.73814400	0.18077900	-0.10606000
C	3.95482200	-1.22112300	-1.32372700
C	2.04132800	-2.58700000	-0.42118700
C	3.29406200	-0.59423600	2.38770900
C	4.36105800	1.33399300	1.14628300
C	2.63765400	2.03875100	-2.11071900
C	1.33702900	3.05377700	-0.18747700
H	4.20029100	-2.01254200	-2.04189600
H	4.29622700	-0.27330400	-1.75130100
H	4.54246000	-1.40985100	-0.41878200
H	2.72558500	1.18870000	-2.79485100

H	2.37582100	2.91758000	-2.71267900
H	3.62573400	2.22589000	-1.67590200
H	2.25340500	3.37802600	0.31723100
H	1.00207100	3.88643200	-0.81773200
H	0.57700000	2.89781700	0.58513500
H	2.54403800	-2.77292400	0.53436900
H	0.96525700	-2.66413600	-0.24034400
H	2.32243300	-3.39936000	-1.10195900
H	4.99524100	0.68845500	0.53034300
H	4.20436700	2.26786200	0.59809500
H	4.92792700	1.57791500	2.05308800
H	3.82770800	-0.31920900	3.30541400
H	2.36539900	-1.09468800	2.68414200
H	3.91306900	-1.32661000	1.85787800
Cl	-1.16200300	-1.80357700	2.75032000

S20

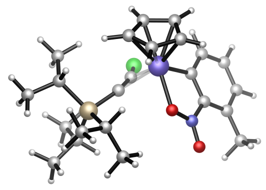

G = -1959.079673

E = -1959.52561603

C	-2.81963300	-1.10096200	-0.20954800
C	-2.53782500	0.19688900	0.24467600
C	-3.97283600	-1.83737100	0.14564000
C	-3.45093900	0.79537100	1.10398800
C	-4.85687100	-1.18264100	0.99821500
C	-4.60333600	0.10456000	1.46850000
H	-3.27111600	1.78759700	1.50202600
H	-5.76316800	-1.69558600	1.30246600
H	-5.31884100	0.57573600	2.13544200
Rh	-0.82846400	1.00610400	-0.46160500
C	-1.51410000	2.35790800	-2.02228300
H	-2.25592000	2.08171200	-2.75883800
C	-0.07695200	2.29067200	-2.19987100
H	0.42748600	1.87903200	-3.06369800
C	0.52988000	2.76904400	-1.03861600
H	1.59069800	2.81664500	-0.84075700
C	-1.76882100	2.96213000	-0.76013800
H	-2.74146600	3.17458900	-0.33979000
C	-0.51664900	3.11648200	-0.09793200
H	-0.36739300	3.52606700	0.89195100
O	-0.87239900	-0.87788100	-1.40484800
O	-1.87108800	-2.76732000	-1.52068100
C	-4.28792100	-3.22756100	-0.32682000
H	-4.34225300	-3.27845100	-1.41762500
H	-5.24816000	-3.54165700	0.08646000
H	-3.52134900	-3.94018500	-0.01197700
N	-1.83261400	-1.64099000	-1.08230000
Si	2.54235700	-0.46771000	0.06428400
C	2.99630700	-1.96034000	1.14198700
C	2.39626000	-0.91181200	-1.77208700
C	3.61532400	1.08131300	0.32951800
H	2.17639900	-2.67203500	0.96562100
H	1.65723700	-0.20557600	-2.17739000
H	3.48972800	1.67823600	-0.58562300
C	3.01663600	-1.62664900	2.64028100
C	4.30445700	-2.65126200	0.72315000
C	3.19093400	1.94651200	1.52464400
C	5.10791100	0.72733800	0.44064900
C	1.86779500	-2.33538500	-2.00189700
C	3.71119500	-0.69771000	-2.53841500
H	3.84931500	2.81892700	1.61465200
H	2.16441100	2.31330200	1.42965600
H	3.25591200	1.38985500	2.46638000
H	5.31591300	0.20728900	1.38168200
H	5.45696500	0.09062400	-0.37817800
H	5.71514800	1.64039100	0.43146900
H	4.42009800	-3.59778400	1.26473900
H	4.33825500	-2.88073400	-0.34631600
H	5.17778800	-2.03499200	0.95710800
H	3.19265100	-2.53238900	3.23284000
H	3.81873300	-0.91936500	2.87912600
H	2.07281300	-1.18879300	2.98292700
H	4.49921800	-1.37091100	-2.18213100
H	3.56527200	-0.90670900	-3.60497000
H	4.08359100	0.32827300	-2.45293000
H	0.93095000	-2.52516700	-1.47299100
H	1.67756100	-2.50350900	-3.06872000
H	2.59717200	-3.08603700	-1.68014100
C	0.83764000	0.01060100	0.71939300
C	-0.07170700	0.30388900	1.51808400
Cl	-0.75059200	0.45337200	3.03078700

S21

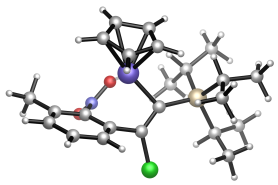

G = -1959.100166

E = -1959.54817671

C	2.54357200	-1.08066400	0.32209500
C	1.69465500	-1.19225200	-0.80066300
C	3.93687600	-0.94293800	0.25228200
C	2.30894800	-1.13794100	-2.06333900
C	4.47928600	-0.89107200	-1.02818500
C	3.68056600	-0.98470400	-2.16956000
H	1.68852000	-1.23565100	-2.94753900
H	5.55221100	-0.76750300	-1.13354800
H	4.14220200	-0.94168700	-3.14976100
Rh	0.93703900	1.02753000	0.01217900
C	1.57705100	3.07234000	0.73049900
H	1.92420000	3.25410200	1.73910300
C	0.17455700	2.99302100	0.33220900
H	-0.67323600	3.21229200	0.96710700
C	0.13532000	2.72290500	-1.06509900
H	-0.74002700	2.66299200	-1.69195100
C	2.36773200	2.76960900	-0.37242600
H	3.44426200	2.67268900	-0.39005100
C	1.47345900	2.46425800	-1.47435100
H	1.78011600	2.16412600	-2.46807500
O	1.13946000	-0.17921000	1.90599900
O	2.20665400	-1.97640500	2.44071600
C	4.79851400	-0.83571000	1.47921900
H	4.39277200	-0.11878200	2.19993200
H	5.80002400	-0.50495700	1.20008700
H	4.88613600	-1.80343900	1.98085600
N	1.92953000	-1.10888800	1.65420800
Si	-2.37667600	-0.12212100	0.10223200
C	-3.36925300	-1.74043800	-0.03660700
C	-2.28127000	0.49469100	1.90334500
C	-3.01840200	1.22547200	-1.07879400
H	-2.74191700	-2.50713900	0.43769000
H	-1.40847600	1.16348800	1.91231900
H	-2.56962700	2.15837900	-0.70775600
C	-3.62825400	-2.16930600	-1.48928400
C	-4.70217500	-1.70153500	0.73229700
C	-2.56531100	1.01354000	-2.53349200
C	-4.54321500	1.41537300	-1.03368300
C	-1.97653700	-0.65084500	2.88090400
C	-3.48355100	1.32491000	2.37192600
H	-2.79245700	1.89595100	-3.14503800
H	-1.49212000	0.81078600	-2.62175300
H	-3.08805300	0.16563700	-2.98698900
H	-5.06320500	0.54453200	-1.44588300
H	-4.92077900	1.57824500	-0.02018800
H	-4.83778100	2.28275700	-1.63770300
H	-5.21740300	-2.66557800	0.63607300
H	-4.56751300	-1.51012400	1.80005000
H	-5.37836600	-0.93467800	0.33998900
H	-4.03441800	-3.18783800	-1.52291200
H	-4.36423200	-1.51187600	-1.96426400
H	-2.72469600	-2.15543900	-2.10528700
H	-4.41791700	0.75392500	2.34390400
H	-3.33859000	1.65818300	3.40742400
H	-3.62268900	2.22119200	1.75824300
H	-1.11226700	-1.24408900	2.56445600
H	-1.75363800	-0.25596700	3.87970400
H	-2.82667900	-1.33292500	2.98448900
C	-0.54435500	-0.38749100	-0.32263200
C	0.22027300	-1.41268000	-0.67338700
Cl	-0.28024700	-3.07381700	-1.04263800

S22

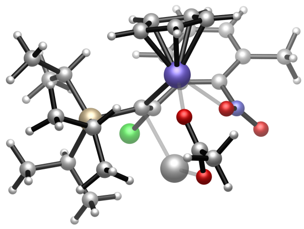

G = -2333.299541

E = -2333.79420684

C	-1.96702400	-1.52354700	-0.62076800
C	-4.61956100	-2.19370700	0.03074800
C	-0.51803400	-1.25560400	-0.90204000
C	0.33684000	-0.54351500	-0.13368100
C	-3.02014800	-0.60838300	-0.72602800
C	-4.35768000	-0.90361500	-0.42645700
C	-2.29552600	-2.80980200	-0.18309300
C	-3.60235100	-3.13406000	0.15306500
H	-5.63885200	-2.45738900	0.29343500
H	-3.82954600	-4.13202100	0.51235000
H	-1.51271800	-3.55577200	-0.09759200
C	0.06180900	-0.08166000	3.13189800
C	-0.93727800	-0.99967600	2.66337000
C	-0.61511400	1.15762000	3.47006400
H	-0.79573900	-2.03093300	2.37429700
H	-0.12175400	2.05414100	3.82082900
C	-2.15439600	-0.28289900	2.57448800
H	-3.08596400	-0.67825700	2.18957700

C	-1.96059000	1.04305400	3.12316000
H	-2.71159500	1.81803300	3.18580000
H	1.10289100	-0.30407400	3.32478200
Si	2.23371000	-0.88820200	0.15523000
C	3.01565900	0.67361500	0.94091900
H	2.21691200	1.13498300	1.53040600
C	2.10409300	-2.43041600	1.30806200
H	1.12960300	-2.32882500	1.79503300
C	3.17983000	-1.41470500	-1.41821700
H	2.75208800	-2.38615200	-1.68723500
O	0.40939400	2.45861100	0.87137300
C	0.54131700	3.33099600	-0.04485400
O	0.45281800	3.14508500	-1.28230700
Ag	0.35415400	1.03842900	-1.98511700
C	0.87432300	4.72295800	0.44762100
H	0.92088500	5.43264800	-0.37850100
H	0.12496900	5.04763700	1.17419500
H	1.84028100	4.69754400	0.96046100
Rh	-0.65031500	0.63862900	1.28074800
O	-1.92093000	1.41772000	-0.42680500
O	-3.29189800	1.24760800	-2.06689900
N	-2.72999300	0.76324300	-1.11268000
C	4.21032000	0.44159300	1.88187900
C	3.43639500	1.71174800	-0.11399100
C	3.07048100	-0.52472000	-2.66231200
C	4.65926800	-1.65590500	-1.06824400
C	3.13886200	-2.57228100	2.43357900
C	2.04998100	-3.73821500	0.49915400
H	4.65453000	1.40779900	2.15239000
H	3.92560700	-0.05150500	2.81205700
H	5.00178700	-0.15515600	1.41676100
H	3.01206300	-1.81174600	3.20873400
H	3.02166800	-3.54827900	2.92111900
H	4.16863700	-2.51159000	2.06692200
H	3.01331300	-3.95738800	0.02558300
H	1.81600900	-4.57884600	1.16365100
H	1.29080200	-3.71906600	-0.28694600
H	4.34955500	1.40057700	-0.63210000
H	2.67668100	1.90903000	-0.87517400
H	3.65250900	2.67052500	0.37290400
H	5.17891000	-0.71823600	-0.84549400
H	4.77978300	-2.31846400	-0.20405100
H	5.17912100	-2.12401700	-1.91305700
H	3.60515300	-0.98108500	-3.50471400
H	2.03053200	-0.41063200	-3.00265700
H	3.49649000	0.46967800	-2.50917500
C	-5.46642400	0.10752000	-0.54478500
H	-5.20561200	1.06140300	-0.07552100
H	-5.70703100	0.30490500	-1.59258000
H	-6.36322100	-0.27442700	-0.05470800
Cl	0.01781100	-2.35752600	-2.23550000

S23

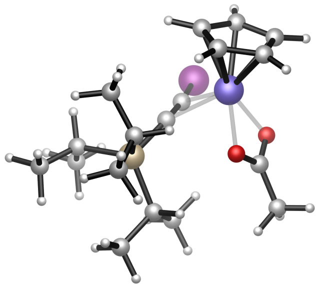

G = -1263.476820

E = -1263.85302210

C	-0.71058700	1.12383200	-0.24574300
C	0.35801600	0.49958800	-0.27330000
C	-1.10128200	-2.67823700	-1.54307500
C	-1.61555000	-1.48953400	-2.12055600
C	-2.02912800	-3.11223000	-0.52881200
H	-1.13121500	-0.88975000	-2.87913700
H	-1.91572100	-3.97915700	0.10769100
C	-2.84450400	-1.16528200	-1.46100800
H	-3.46641600	-0.30418300	-1.66224900
C	-3.11268100	-2.19428400	-0.50718500
H	-3.94368600	-2.21297700	0.18585800
H	-0.16549900	-3.15822700	-1.79478500
Si	2.20732800	0.12419500	-0.23240900
C	2.72328200	0.18527300	1.59258600
H	2.46749300	-0.81193200	1.97509300
C	2.42535400	-1.58961200	-1.00457500
H	1.64884000	-2.20402400	-0.52975300
C	2.91488800	1.51129400	-1.31859800
H	2.33122100	1.45795200	-2.24923700
O	0.13221100	-1.78138100	1.53007100
C	-0.55513000	-1.18026800	2.41734400
O	-1.61189700	-0.59402300	2.02451800
C	-0.15085800	-1.19130400	3.85219000
H	0.92614200	-1.33886700	3.94170700
H	-0.45439800	-0.26377500	4.33954600
H	-0.65949800	-2.02494300	4.34764400
Rh	-1.23271500	-1.17905900	-0.00454700
C	4.23755600	0.39014100	1.76434300
C	1.95584400	1.22217400	2.42676200

C	2.69530000	2.90233000	-0.70730100
C	4.39211200	1.30463300	-1.68928800
C	3.77941900	-2.24023800	-0.68883300
C	2.15808200	-1.56184200	-2.51569900
H	4.52033300	0.25844600	2.81570400
H	4.83343800	-0.31284700	1.17401400
H	4.53307600	1.40419700	1.47346200
H	3.92348200	-2.37664900	0.38766200
H	3.84575000	-3.23004300	-1.15677900
H	4.61903800	-1.64669900	-1.06693500
H	2.95092500	-1.02872900	-3.05157700
H	2.11865200	-2.57854800	-2.92519900
H	1.21072300	-1.06870300	-2.76025000
H	2.20791500	2.24393200	2.12305900
H	0.87063500	1.11818400	2.33679300
H	2.21352200	1.12509800	3.48819800
H	5.04175400	1.33657800	-0.80870500
H	4.56355400	0.34930900	-2.19462000
H	4.72559700	2.09855200	-2.36873600
H	3.01998200	3.68363200	-1.40498000
H	1.64213000	3.08990200	-0.46928800
H	3.27425900	3.02638900	0.21428700
I	-2.12199500	2.53097000	-0.21249800

S24

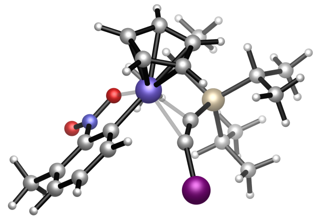

G = -1510.306237

E = -1510.74976511

C	-2.61970700	-1.10720000	0.98090800
C	-2.41751500	-0.41084500	-0.21997000
C	-3.77591700	-0.99104100	1.78645500
C	-3.43247700	0.43099700	-0.65907000
C	-4.75649400	-0.13304600	1.29713800
C	-4.59356400	0.55765600	0.09682500
H	-3.32086000	0.99723000	-1.57700300
H	-5.67057600	-0.00561300	1.86765600
H	-5.38677900	1.21335200	-0.24933700
Rh	-0.65451700	-0.75183600	-1.14037100
C	-1.07452200	-2.10732400	-2.78793400
H	-1.65123600	-3.01459500	-2.66909600
C	0.37219700	-2.01434100	-2.75414500
H	1.04927300	-2.82825300	-2.53337600
C	0.72384100	-0.68297400	-2.97217700
H	1.72319300	-0.27485900	-2.97597900
C	-1.59007900	-0.82392000	-3.11826000
H	-2.63343100	-0.57186400	-3.24416700
C	-0.49625800	0.08834600	-3.12296600
H	-0.55305400	1.15068700	-3.31774500
O	-0.59752800	-2.07830600	0.50058900
O	-1.52141400	-2.62766300	2.35217600
C	-3.99827400	-1.71348200	3.08445400
H	-3.96391200	-2.79798300	2.94951000
H	-4.97450400	-1.44104600	3.48986000
H	-3.23021900	-1.46020500	3.81973300
N	-1.55076200	-1.98085600	1.33003500
Si	2.66474700	-0.18340300	0.45735800
C	3.05140600	0.63048600	2.12657700
C	2.69240200	-2.07430300	0.54227400
C	3.67174700	0.48424600	-1.01275600
H	2.25228700	0.27549300	2.79345300
H	1.99134000	-2.39952200	-0.23916100
H	3.57535600	-0.28640400	-1.79197300
C	2.97363100	2.16238700	2.06832600
C	4.39223600	0.17854800	2.72827800
C	3.14498100	1.81100300	-1.58129200
C	5.16914600	0.61199100	-0.68583400
C	2.17604700	-2.60563700	1.88770400
C	4.06674100	-2.67675600	0.21307700
H	3.73110100	2.11055900	-2.45856000
H	2.09562800	1.74901600	-1.88558200
H	3.22329900	2.61702100	-0.84388000
H	5.34669700	1.41618400	0.03613600
H	5.59513400	-0.30768800	-0.27335600
H	5.73441600	0.85843600	-1.59266000
H	4.51714000	0.60407500	3.73150100
H	4.46724200	-0.90885400	2.82372000
H	5.24094400	0.51798300	2.12663300
H	3.09105300	2.59367900	3.06982600
H	3.77198900	2.57520400	1.44154700
H	2.01766300	2.51687800	1.66730100
H	4.82676100	-2.37036800	0.94063700
H	4.01699000	-3.77219400	0.23826000
H	4.41974400	-2.38683200	-0.78218000
H	1.22050500	-2.16017100	2.17515700
H	2.02591400	-3.69060000	1.83858500
H	2.89456700	-2.41155800	2.69066800
C	0.89271600	0.36710700	0.11706700

C	-0.08697500	1.12986400	0.01477800
I	-1.02644900	2.88936300	0.25166900

S25

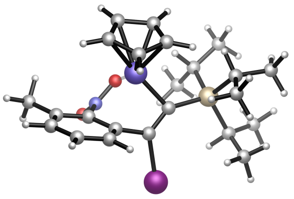

G = -1510.318355

E = -1510.76367060

C	-2.57379000	0.68878900	0.53791900
C	-1.71035000	0.85555800	-0.56684900
C	-3.96635000	0.62179100	0.45303900
C	-2.30992000	0.90676900	-1.83537700
C	-4.49920100	0.69779100	-0.83535200
C	-3.68883300	0.82304700	-1.96196200
H	-1.67463900	1.04505400	-2.70360000
H	-5.57610000	0.63625800	-0.95400600
H	-4.14221900	0.86548000	-2.94599500
Rh	-0.97917200	-1.41775000	-0.14150900
C	-1.84764800	-3.47147700	0.28465000
H	-2.31321200	-3.72622400	1.22741500
C	-0.40995300	-3.46893900	0.03264100
H	0.34858800	-3.81912100	0.71967200
C	-0.20368300	-3.06549400	-1.31606300
H	0.73347900	-3.02934400	-1.84652900
C	-2.48877800	-2.98214500	-0.84512000
H	-3.54669400	-2.78595000	-0.95264300
C	-1.46557600	-2.64553500	-1.82252700
H	-1.64573200	-2.23006700	-2.80515700
O	-1.17486600	-0.47521100	1.93921000
O	-2.18747300	1.24413200	2.76247300
C	-4.84587000	0.43909900	1.65822300
H	-4.50365800	-0.38762600	2.28881200
H	-5.86783900	0.22344900	1.34301000
H	-4.86075900	1.34406800	2.27175300
N	-1.94458700	0.49975700	1.84707000
Si	2.35022300	-0.40480200	0.17492900
C	3.43390600	1.15755200	0.20712200
C	2.14827100	-1.16414700	1.91047400
C	2.96523600	-1.68530300	-1.08929300
H	2.84613900	1.90145200	0.76117700
H	1.23691500	-1.77449300	1.82665700
H	2.46677700	-2.62149000	-0.79978000
C	3.71230400	1.72186300	-1.19460300
C	4.75614200	0.96778000	0.96939900
C	2.56929000	-1.34190300	-2.53524900
C	4.47790500	-1.95005100	-1.01970200
C	1.87585900	-0.08102800	2.96595100
C	3.27815500	-2.10266500	2.35289300
H	2.79359000	-2.17756200	-3.21028700
H	1.50614700	-1.09976000	-2.64261400
H	3.13283100	-0.47657000	-2.89752100
H	5.04754000	-1.08146400	-1.36567200
H	4.82112200	-2.19183900	-0.00967400
H	4.74729600	-2.79189400	-1.66967000
H	5.31333800	1.91280600	0.99477500
H	4.59996400	0.65544600	2.00551500
H	5.40230300	0.22475000	0.49170200
H	4.15558300	2.72296800	-1.12666700
H	4.42222600	1.08855000	-1.73795000
H	2.80792900	1.80398400	-1.80568000
H	4.24011800	-1.58372400	2.42472900
H	3.06306300	-2.52361800	3.34324300
H	3.40325700	-2.94228300	1.66092100
H	1.08893700	0.61345500	2.65350600
H	1.55556900	-0.53282400	3.91258200
H	2.77436000	0.50943700	3.17502400
C	0.54086600	-0.00786200	-0.26083900
C	-0.22966500	1.06059600	-0.41125500
I	0.30536500	3.13585900	-0.42112800

S26

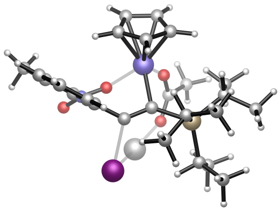

G = -1884.514193

E = -1885.00364195

C	-1.90922000	-0.73885500	1.06141500
C	-4.49648000	-0.62794600	2.13883900
C	-0.50695900	-0.80619800	0.56921000
C	0.44823900	0.10141600	0.49377800
C	-3.03008000	-0.42722300	0.28239100
C	-4.33846200	-0.36418800	0.77719000
C	-2.12744600	-1.01543600	2.41127600

C	-3.40942200	-0.95276500	2.94289900
H	-5.49031900	-0.56740800	2.56976200
H	-3.56167100	-1.15292600	3.99811900
H	-1.28336600	-1.27736100	3.03941200
C	0.13587100	3.35939500	1.53491900
C	-0.67964000	2.42981700	2.26064800
C	-0.73285200	4.11085800	0.64632100
H	-0.35191400	1.74603800	3.03024200
H	-0.40081000	4.86779700	-0.05117700
C	-1.98175600	2.50165200	1.70756400
H	-2.82528700	1.88778100	1.99581200
C	-2.01966600	3.58757000	0.74370400
H	-2.88002600	3.87751900	0.15620000
H	1.18550600	3.55715600	1.69876600
Si	2.32838000	0.00502500	0.94485400
C	3.27187700	1.65952500	0.78244900
H	2.90911700	2.25854200	1.63074000
C	2.13594100	-0.33988900	2.82576700
H	1.32366400	0.34043700	3.12196100
C	3.20930500	-1.39543900	0.00389400
H	2.48534300	-2.21616000	-0.02130600
O	0.28140900	2.30882700	-1.72952900
C	0.71449500	1.81538500	-2.81451800
O	0.76272500	0.60843100	-3.15067700
Ag	0.18078300	-1.26412800	-2.16501200
C	1.24758300	2.83763500	-3.79836600
H	2.14911800	3.29404500	-3.37678100
H	1.48984100	2.37469800	-4.75517500
H	0.51298300	3.63373100	-3.94392300
Rh	-0.58063200	1.90744000	0.17848200
O	-1.92334700	0.77216700	-1.35427400
O	-3.47084500	-0.58383900	-1.97485100
N	-2.80408100	-0.06897500	-1.10415700
C	4.78610700	1.45387500	0.99549800
C	3.08600700	2.48705400	-0.49382100
C	3.53756600	-1.01928100	-1.44944800
C	4.46271100	-1.94440600	0.70623600
C	3.34992500	0.02544000	3.69294700
C	1.67597400	-1.76567400	3.15428600
H	5.27161300	2.42618300	1.14412700
H	5.03762300	0.82989500	1.85536500
H	5.24464000	0.99990200	0.11115100
H	3.63562100	1.07620400	3.59012300
H	3.11839800	-0.14689800	4.75158600
H	4.22366100	-0.58965500	3.45122000
H	2.43335300	-2.50890700	2.88379400
H	1.48901900	-1.87044900	4.23035000
H	0.75473200	-2.03776500	2.63499100
H	3.33521700	1.90545700	-1.38871500
H	2.06832500	2.85222400	-0.61703300
H	3.75879600	3.35446200	-0.47585800
H	5.27468600	-1.21284000	0.72804600
H	4.26470600	-2.25096500	1.73738700
H	4.83467100	-2.82570100	0.16905200
H	3.84600400	-1.90517800	-2.01837600
H	2.69150600	-0.56196200	-1.97621900
H	4.36156600	-0.29942400	-1.49532400
C	-5.52368500	0.01905200	-0.06677500
H	-5.31396800	0.89120200	-0.69340100
H	-5.81854100	-0.80033900	-0.72727100
H	-6.37030800	0.25984400	0.57848100
I	-0.22218400	-3.03793200	-0.00596700

S27

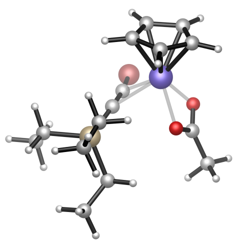

G = -1147.421401

E = -1147.71456436

C	-0.40749600	1.43974900	-0.28103000
C	0.57689300	0.69646100	-0.35291800
C	-1.37280800	-2.57757300	-1.19607900
C	-1.25826000	-1.46580900	-2.06679600
C	-2.54745500	-2.40058800	-0.39011500
H	-0.45246200	-1.28367900	-2.76427000
H	-2.90205200	-3.07352700	0.37817800
C	-2.38757700	-0.60529000	-1.83721300
H	-2.58919500	0.32478100	-2.35055900
C	-3.19063200	-1.19751000	-0.82612900
H	-4.08292600	-0.76380900	-0.39409400
H	-0.64430700	-3.37029600	-1.08757500
Si	2.31251900	-0.04271900	-0.39395000
C	2.98925200	0.17738500	1.34958200
H	2.56476300	-0.61444700	1.97802900
C	2.17676800	-1.83691000	-0.93293300
H	1.46484400	-2.33853500	-0.26783900
C	3.23856000	1.00634500	-1.64888500
H	2.78429300	0.85184300	-2.63566600

O	-0.02122300	-1.32604800	1.64759900
C	-0.67587900	-0.54514900	2.41130300
O	-1.67191000	0.05220600	1.89065200
C	-0.26581800	-0.30441600	3.82085100
H	0.46461000	0.51138800	3.83053900
H	-1.12730300	-0.01030600	4.42202400
H	0.20648500	-1.19658900	4.23436400
Rh	-1.22518700	-0.75354800	-0.01905400
C	4.52281000	0.16706900	1.42997800
C	3.28400100	2.50157500	-1.31188800
C	3.51915600	-2.58224300	-0.91584000
H	4.86021500	0.28522900	2.46494300
H	4.94748400	-0.76864000	1.05208100
H	4.96057200	0.98483400	0.84799500
H	3.92159300	-2.65451900	0.09957200
H	3.40589100	-3.60280400	-1.29577800
H	4.27154100	-2.08483100	-1.53730100
H	3.84809300	3.06091000	-2.06521000
H	2.27832500	2.93413200	-1.26745100
H	3.76138600	2.68156600	-0.34275200
Br	-1.52721800	2.86071300	-0.19524300
H	1.74211800	-1.86598900	-1.93997500
H	4.25797200	0.60462900	-1.72100700
H	2.60478200	1.12357400	1.75064300

S28

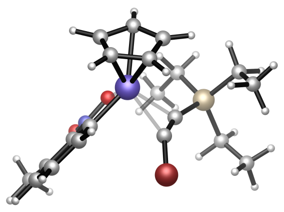

G = -1394.254533

E = -1394.61612484

C	2.47561600	1.00327900	-0.09183400
C	2.06011600	-0.33764700	-0.11587400
C	3.68980700	1.45175100	0.47678200
C	2.90494600	-1.28784500	0.44331100
C	4.49742900	0.45481700	1.01616900
C	4.11734300	-0.88590900	0.99726200
H	2.62743400	-2.33567900	0.46184700
H	5.44479000	0.73686200	1.46346900
H	4.78056200	-1.63070500	1.42655800
Rh	0.27381800	-0.66921600	-0.99901200
C	0.78672700	-1.48044600	-2.94887100
H	1.55391100	-1.05805300	-3.58343200
C	-0.63359600	-1.20033500	-3.03543100
H	-1.08675900	-0.46945700	-3.69165400
C	-1.29229600	-1.96896800	-2.07622900
H	-2.35097800	-1.96273800	-1.86159300
C	0.97007000	-2.50043100	-1.97564200
H	1.91015000	-2.95342500	-1.69517400
C	-0.29083900	-2.72770200	-1.35215700
H	-0.48739300	-3.43344300	-0.55665700
O	0.54033200	1.40607800	-1.25935400
O	1.73103100	3.10657900	-0.73872900
C	4.14124800	2.88312300	0.52887600
H	4.22904400	3.31507700	-0.47168800
H	5.11465600	2.93848600	1.01978300
H	3.43412700	3.50514000	1.08390300
N	1.56626900	1.90891900	-0.70915400
Si	-2.84075500	1.00038700	0.04572100
C	-3.23076400	2.02224900	1.57632800
C	-2.59180900	2.07678900	-1.47278100
C	-4.11721000	-0.34254100	-0.30324300
H	-2.36986600	2.67139800	1.77985700
H	-1.99003200	1.51617000	-2.19874800
H	-4.06507100	-0.58472400	-1.37267100
C	-3.59382800	1.21407700	2.82747400
C	-4.01586100	-1.62229900	0.53519400
C	-1.97553000	3.45698800	-1.21719500
H	-4.78314900	-2.34639600	0.24275600
H	-3.03984100	-2.10532600	0.41433200
H	-4.14422900	-1.41842900	1.60250900
H	-3.75864000	1.87151000	3.68728700
H	-4.51121000	0.63657400	2.67623900
H	-2.80056700	0.51013100	3.10229300
H	-0.99162400	3.38005300	-0.74687800
H	-1.84444300	4.00542900	-2.15587400
H	-2.61357300	4.06508500	-0.56779100
C	-1.25602300	0.08566000	0.49789500
C	-0.40783700	-0.56362100	1.13885200
Br	0.26782600	-1.30299300	2.66548500
H	-4.05869200	2.69487300	1.31375100
H	-3.58249100	2.19364000	-1.93334300

S29

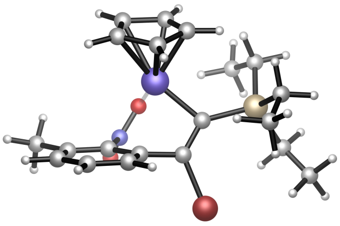

G = -1394.274826

E = -1394.63890940

C	2.14189100	-1.16310400	0.46124700
C	1.13243100	-1.23193000	-0.52206700
C	3.50654000	-1.32002000	0.21126400
C	1.55279500	-1.46065600	-1.84080600
C	3.85730500	-1.55242700	-1.12021900
C	2.90138200	-1.61429500	-2.13067400
H	0.80421100	-1.53615500	-2.62239200
H	4.90797800	-1.67080300	-1.36493700
H	3.21301100	-1.78695400	-3.15490300
Rh	0.77879500	1.20966800	-0.12664300
C	2.00742500	3.13783300	0.00275700
H	2.76930300	3.31817400	0.74860000
C	0.57371300	3.29699700	0.21155700
H	0.11131300	3.69467000	1.10557100
C	-0.09547700	2.96840600	-1.00476600
H	-1.15414500	3.05104600	-1.19985500
C	2.19637100	2.60832200	-1.26363900
H	3.13556100	2.28765400	-1.69381900
C	0.88566500	2.41892900	-1.87577100
H	0.70949300	2.02996300	-2.86972700
O	1.16525400	0.30167700	1.93434200
O	1.95013100	-1.53857700	2.74460900
C	4.54687200	-1.20922400	1.29098400
H	4.37237000	-0.34155400	1.93433200
H	5.53626700	-1.10549500	0.84323300
H	4.54925500	-2.10080300	1.92430600
N	1.72839500	-0.80290200	1.81676600
Si	-2.67452600	0.61424500	0.27787100
C	-3.76901100	-0.71170400	1.07715700
C	-2.63916800	2.09592800	1.44416300
C	-3.33560300	1.12897100	-1.41899600
H	-3.15106600	-1.37332300	1.69549300
H	-2.14706900	2.94751300	0.96073200
H	-3.03468800	2.16296700	-1.62792500
C	-4.65455000	-1.54163700	0.13940300
C	-2.91945300	0.22408900	-2.58487000
C	-1.98988800	1.80776100	2.80269300
H	-3.38418800	0.54373100	-3.52363000
H	-1.83358000	0.24094400	-2.73038000
H	-3.20639600	-0.81846800	-2.41339800
H	-5.29579600	-2.22324700	0.70878800
H	-5.30936500	-0.90495300	-0.46450300
H	-4.06137100	-2.15095000	-0.54847300
H	-0.93852900	1.52211400	2.69126300
H	-2.02758600	2.68056500	3.46342100
H	-2.49461300	0.98456600	3.32072600
C	-0.89481100	0.02773100	0.00861000
C	-0.32968900	-1.15405100	-0.19859600
Br	-1.20620100	-2.88291900	-0.21975500
H	-4.41240200	-0.16739400	1.78227900
H	-3.68323700	2.40471900	1.59019600
H	-4.43041300	1.15916000	-1.34036800

S30

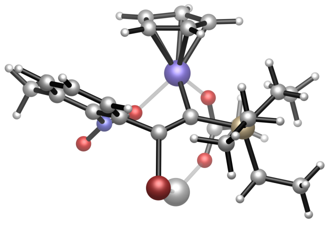

G = -1768.472789

E = -1768.88052868

C	-1.92724800	-1.06745100	0.70793900
C	-4.70569300	-1.06467000	1.08363900
C	-0.44466900	-1.03578500	0.60294800
C	0.41687500	-0.03432600	0.70987100
C	-2.81501500	-0.66067500	-0.29442100
C	-4.20771600	-0.63969900	-0.14829200
C	-2.48123600	-1.50827400	1.91044200
C	-3.85740000	-1.50308000	2.09496700
H	-5.77841300	-1.04699900	1.24660000
H	-4.27290000	-1.83496900	3.04035000
H	-1.82193700	-1.84114900	2.70512100
C	-0.13518200	3.40075800	1.51486100
C	-0.78374300	2.36717600	2.26262200
C	-1.08535100	3.94120900	0.56443200
H	-0.35316400	1.80023600	3.07627500
H	-0.88280700	4.72976400	-0.14646100
C	-2.05817300	2.16166600	1.66966100
H	-2.79747000	1.43608800	1.98025000
C	-2.24612700	3.17666300	0.64837300
H	-3.11497200	3.26069200	0.00862700
H	0.86062200	3.78585500	1.68558200

Si	2.21648200	-0.07009400	1.36450600
Br	0.18791200	-2.97588200	0.37206500
C	2.97819700	1.63341900	1.66693800
H	2.29053100	2.22646600	2.28100600
C	2.13981800	-0.74530500	3.13594600
H	1.53910800	-0.03017700	3.71549100
C	3.38482000	-1.04647700	0.24554000
H	3.01654100	-2.07015900	0.11183700
O	0.91764600	2.19654200	-1.29105300
C	1.56567400	1.66057100	-2.24050200
O	1.62001600	0.44829600	-2.55202500
Ag	0.96236000	-1.56227000	-1.93743200
C	2.39737400	2.61090400	-3.07962000
H	3.45564800	2.41981100	-2.87507900
H	2.22536600	2.41381400	-4.14056000
H	2.17250600	3.65317600	-2.85199800
Rh	-0.50598400	1.75144900	0.22307700
O	-1.37515900	0.66223600	-1.55551400
O	-2.68021700	-0.68806500	-2.59066900
N	-2.26200600	-0.20900500	-1.55987100
C	3.51559800	2.46475900	0.50032500
C	4.82178000	-1.09273600	0.78599500
C	1.66757000	-2.16773900	3.43684600
H	2.22835700	-2.91367900	2.86494300
H	1.79774700	-2.40111200	4.49946800
H	0.60697200	-2.30108300	3.20442600
H	4.15139000	1.86160500	-0.15837100
H	2.70584400	2.87221100	-0.10621800
H	4.12483200	3.29992900	0.86371200
H	5.25099900	-0.09018000	0.88435800
H	4.86484700	-1.56978000	1.77090000
H	5.47440600	-1.66490900	0.11838600
C	-5.14349500	-0.15648500	-1.22311500
H	-4.77990200	0.75589900	-1.70488800
H	-5.27223800	-0.91454900	-2.00027100
H	-6.12122700	0.05521000	-0.78724100
H	3.16432600	-0.62160800	3.51358300
H	3.81293100	1.39634900	2.34259000
H	3.37852200	-0.57368700	-0.74608600

S31

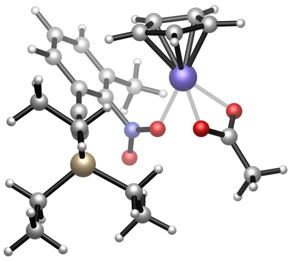

G = -1609.467694

E = -1609.87479717

C	0.61693500	2.35444800	-0.07217000
C	3.20004700	3.43105100	-0.19148200
C	-0.68841100	1.77658300	-0.02311400
C	-1.77849400	1.23805700	0.01379000
C	1.57393800	1.91047500	-0.99734600
C	2.86515300	2.42378500	-1.10141800
C	0.99558100	3.37547200	0.80413000
C	2.28175700	3.90018700	0.74255300
H	4.20038500	3.85025200	-0.22537600
H	2.57185300	4.68595600	1.43157400
H	0.27544700	3.74544400	1.52489000
C	0.79905600	-1.34177800	2.69796900
C	1.01669000	0.01651800	2.35170100
C	1.99139800	-2.07566400	2.39479200
H	0.28861000	0.81226300	2.42424500
H	2.13478600	-3.14042800	2.51982000
C	2.36874700	0.14070100	1.88088200
H	2.82972500	1.04858500	1.51587800
C	2.97287100	-1.13521000	1.91716900
H	3.97199600	-1.37718200	1.58083600
H	-0.13536800	-1.76915600	3.03716200
Si	-3.43368200	0.39470700	0.04865700
C	-3.26830800	-1.23935000	-0.86741700
H	-2.65201300	-1.91059300	-0.25925100
C	-3.86423000	0.11394600	1.86069700
H	-3.12466500	-0.57956300	2.28254800
C	-4.65754200	1.55616400	-0.78819300
H	-4.62250400	2.52909300	-0.28275400
O	-0.28511200	-2.45898900	-0.19294500
C	0.52813300	-3.04941100	-0.97614100
O	1.75890400	-2.76260000	-0.84395900
C	0.04592100	-3.99154700	-2.02326000
H	-0.08032100	-3.43209300	-2.95589300
H	0.78247300	-4.77903700	-2.19141200
H	-0.91560100	-4.41899800	-1.73672800
Rh	1.26323300	-1.25400800	0.60492100

O	0.88614900	-0.27071400	-1.44057200
O	1.15679000	1.06257100	-3.09564100
N	1.18376800	0.83878200	-1.90844400
C	-2.65978300	-1.12826400	-2.27020000
C	-6.09378300	1.01567800	-0.79399100
C	-3.92671400	1.39644700	2.69899300
H	-4.69819800	2.08061600	2.32933000
H	-4.15540800	1.18047600	3.74803400
H	-2.97274600	1.93558200	2.67362300
H	-3.25496600	-0.47764500	-2.92084100
H	-1.64426600	-0.72089500	-2.22620500
H	-2.59985100	-2.10860600	-2.75536000
H	-6.16190400	0.05872900	-1.32292400
H	-6.46891200	0.85736200	0.22322200
H	-6.77888200	1.71161400	-1.28951400
C	3.85922400	1.92207100	-2.11226300
H	3.83363200	0.83263800	-2.20862900
H	3.65691600	2.34974100	-3.09858500
H	4.86924400	2.21318100	-1.81794700
H	-4.82642200	-0.41305500	1.90423500
H	-4.26706400	-1.69337800	-0.92063300
H	-4.31925900	1.73780500	-1.81600900

S32

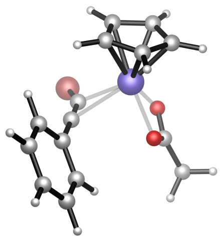

G = -851.948492

E = -852.141380070

C	-0.33441300	1.28858000	-0.52382400
C	0.85772400	0.98322900	-0.40699700
C	-0.03710000	-2.75990100	-0.73598400
C	-0.12917900	-1.83139900	-1.80409200
C	-1.33849800	-2.89759500	-0.14595600
H	0.69249400	-1.48229100	-2.41430100
H	-1.59696200	-3.52417600	0.69634900
C	-1.49640800	-1.39879500	-1.90256200
H	-1.89100600	-0.68916700	-2.61658000
C	-2.23822200	-2.07946000	-0.89865400
H	-3.28752600	-1.92827900	-0.68050700
H	0.87390500	-3.21745700	-0.37360100
O	0.21195900	-0.67283800	1.94696300
C	-0.81133300	-0.07574000	2.41221100

O	-1.79436900	0.06946700	1.61672500
C	-0.86250100	0.41018500	3.81853700
H	0.11373100	0.79538500	4.11836200
H	-1.62835000	1.17867000	3.92802800
H	-1.11140700	-0.43446800	4.46913400
Rh	-0.71537500	-0.84020600	0.03446500
Br	-1.68091300	2.47902200	-0.84737900
C	2.24180800	0.66952700	-0.28232900
C	2.96480800	0.23080300	-1.40392100
C	2.87592800	0.82109500	0.96108800
C	4.31801000	-0.04784800	-1.27601500
H	2.46257800	0.11943900	-2.35901700
C	4.23009400	0.53794500	1.07362100
H	2.30130000	1.15349300	1.81779900
C	4.94854900	0.10542500	-0.04061700
H	4.88374400	-0.38206500	-2.13912400
H	4.72726200	0.65679700	2.03031300
H	6.00774800	-0.11173400	0.05396800

S33

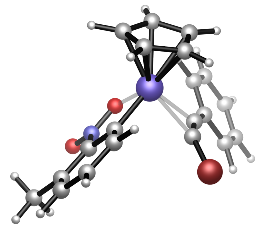

G = -1098.782899

E = -1099.04343646

C	1.96198600	-1.37889600	0.46050300
C	1.80423400	0.00065700	0.25767600
C	3.13617600	-2.09923300	0.14355000
C	2.86955800	0.70492900	-0.28611400
C	4.17274200	-1.34089900	-0.39394800
C	4.04548700	0.03076100	-0.60355800
H	2.79088400	1.77017300	-0.47076400
H	5.10161400	-1.83874100	-0.65169800
H	4.88018700	0.58235800	-1.02489800
Rh	0.01896800	0.75664400	0.81996800
C	0.40787400	1.76170600	2.73011500
H	0.98742300	1.31772900	3.52787500
C	-1.03659700	1.72398400	2.61031200
H	-1.70144100	1.17918800	3.26740300
C	-1.40450400	2.43609300	1.46715200
H	-2.40421000	2.55085700	1.07178600
C	0.90934000	2.57764100	1.68233900
H	1.94683700	2.82346000	1.50508700

C	-0.18918500	2.90980500	0.83574600
H	-0.13929100	3.50826900	-0.06371000
O	-0.15938800	-1.28136200	1.33865200
O	0.76442200	-3.20897200	1.24098100
C	3.32593500	-3.57454400	0.35070600
H	3.19590900	-3.85123400	1.40046200
H	4.33232400	-3.85724500	0.03619200
H	2.60269400	-4.15748500	-0.22548800
N	0.82684900	-2.02076900	1.03301800
C	-1.38663800	0.08756400	-0.79002500
C	-0.38199400	0.57694800	-1.33783600
Br	0.54507100	1.13521900	-2.81190500
C	-2.65288900	-0.56454800	-0.57824300
C	-3.28491900	-0.58640700	0.67000600
C	-3.25870800	-1.18566400	-1.68231200
C	-4.51581500	-1.21565300	0.81057000
H	-2.80985700	-0.11537100	1.52205300
C	-4.49085500	-1.80865500	-1.53012500
H	-2.76348500	-1.17409500	-2.64775300
C	-5.12058500	-1.82539300	-0.28644200
H	-5.00234300	-1.23134100	1.78021100
H	-4.95893200	-2.28454000	-2.38552700
H	-6.08142300	-2.31676600	-0.17174800

S34

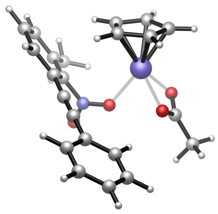

G = -1313.997933

E = -1314.30474815

C	0.85218600	-2.29278900	-0.10466500
C	3.61972000	-2.74031200	-0.04287300
C	-0.55363600	-2.05217300	-0.12296900
C	-1.74332300	-1.82651300	-0.13224500
C	1.68759400	-1.70682000	0.85954600
C	3.06544500	-1.90998500	0.93501400
C	1.45483700	-3.13219700	-1.04786200
C	2.82747900	-3.34448100	-1.01449600
H	4.69105500	-2.91328100	-0.03251600
H	3.28570200	-3.99166400	-1.75462600
H	0.83515900	-3.60993800	-1.79803600
C	0.28461300	1.53113400	-2.59695600
C	0.84180700	0.25472700	-2.32649800
C	1.21457100	2.52788300	-2.15589000
H	0.36208200	-0.69699900	-2.50530900
H	1.07156600	3.59879700	-2.20527400
C	2.14107800	0.45560400	-1.74922400
H	2.80519100	-0.32517600	-1.40178000
C	2.38104800	1.84513700	-1.65783100
H	3.25508500	2.31393900	-1.22669900
H	-0.70800900	1.72026500	-2.98418700
O	-1.23532600	2.09356300	0.29744500
C	-0.63574000	2.84125000	1.13721100
O	0.63138700	2.89419800	1.05504300
C	-1.38049400	3.58285700	2.19192800
H	-1.37658100	2.97921800	3.10546300
H	-0.88578400	4.53186600	2.40502600
H	-2.41376000	3.74910900	1.88520700
Rh	0.63128700	1.40746300	-0.47960100
O	0.48420300	0.19566100	1.46640400
O	1.13823300	-1.12845000	3.01763000
N	1.06973300	-0.82951300	1.84860800
C	3.92484700	-1.26364000	1.98653500
H	3.67936000	-0.20598000	2.12266300
H	3.79892800	-1.76161800	2.95207200
H	4.97617500	-1.33446100	1.70295400
C	-3.14394700	-1.53565400	-0.11399900
C	-3.59015800	-0.30531000	0.39328700
C	-4.07154100	-2.47463900	-0.58895900
C	-4.95115000	-0.02516900	0.42261800
H	-2.86537400	0.42260900	0.74469800
C	-5.43003300	-2.18345000	-0.55205200
H	-3.72093100	-3.42471600	-0.97899300
C	-5.87160300	-0.96141000	-0.04651800
H	-5.29525500	0.92681000	0.81461600
H	-6.14637300	-2.91270300	-0.91655700
H	-6.93385500	-0.73939000	-0.01772800

AcOH

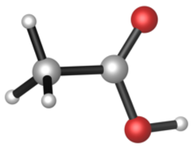

G = -228.989786

E = -229.024496662

C	0.11012800	-0.84171400	-0.65979000
H	0.70573200	-1.65829900	-1.07726400
H	-0.17204000	-0.14771300	-1.45037600

H	-0.78549800	-1.28135800	-0.21251700
C	0.90263200	-0.12178800	0.39075100
O	1.21194500	1.04884300	0.36854700
O	1.24987100	-0.93782900	1.39960100
H	1.75343500	-0.40793300	2.03797100

AgOAc

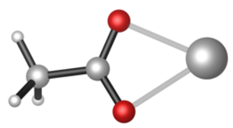

G = -374.186238

E = -374.204717711

C	0.08608200	-0.86459400	-0.68566000
H	0.72845300	-1.62559000	-1.14047600
H	-0.24831900	-0.17320400	-1.46013100
H	-0.77399900	-1.38082200	-0.25181000
C	0.86924900	-0.14842100	0.40005900
O	1.15522400	-0.80491400	1.44245200
O	1.21824900	1.04893200	0.20712700
Ag	2.37787200	1.03377200	2.29778100

AgCl

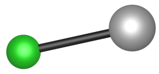

G = -606.027354

E = -606.003493478

Ag	-1.21741900	-1.60099300	-1.34861600
Cl	-2.15113500	-3.47721400	-0.09893900

AgI

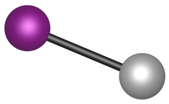

G = -157.259361

E = -157.233292641

Ag	-1.23224900	-1.63079100	-1.32876900
I	-2.26641300	-3.70885400	0.05534800

AgBr

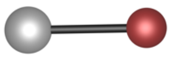

G = -159.034553

E = -159.009407267

Br	-2.20476000	-3.58496800	-0.02716800
Ag	-1.23847100	-1.64329400	-1.32044100

1a

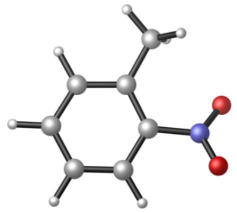

G = -475.821388

E = -475.921462222

C	-0.52384300	-1.46772000	-0.07305900
C	-1.90922700	-1.42792600	-0.09442500
C	-2.55548600	-0.19579600	-0.03188700
C	-1.81810200	0.98000500	0.04882800
C	-0.41836400	0.98436300	0.05263600
C	0.19608100	-0.27581500	-0.01215600
H	0.00975900	-2.40935100	-0.11374800
H	-2.47556700	-2.35062400	-0.15720900
H	-3.63986700	-0.14803200	-0.04128900
H	-2.33594000	1.93211800	0.11468500
C	0.31037000	2.29770400	0.15386900
H	-0.39521800	3.08680300	0.42279300
H	1.10264900	2.26733600	0.90574900
H	0.78087700	2.56031200	-0.79667200
N	1.65417800	-0.40698300	-0.01979400
O	2.13653700	-1.45332300	0.39487900
O	2.32414800	0.52100100	-0.45220200

1j

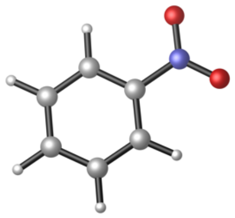

G = -436.542447

E = -436.615062001

C	0.42489100	-1.21917500	-0.00006000
C	1.81473100	-1.20997800	-0.00006700
C	2.50729000	0.00000400	0.00000100
C	1.81473600	1.20997400	0.00006900
C	0.42488500	1.21917300	0.00005300

C	-0.24352600	0.00000400	-0.00000200
H	-0.13378200	-2.14664500	-0.00010300
H	2.35577700	-2.15003600	-0.00012300
H	3.59271200	-0.00000400	0.00000400
H	2.35576800	2.15004100	0.00013000
N	-1.70791700	-0.00000100	0.00000100
O	-2.28370500	-1.07917700	0.00016300
O	-2.28371100	1.07917600	-0.00016000
H	-0.13376700	2.14665500	0.00009900

1p

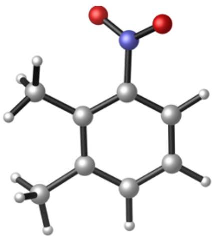

G = -515.103951

E = -515.229749727

C	-0.42675200	1.68685700	-0.06363000
C	0.86843300	2.17303100	-0.10765300
C	1.93412800	1.27833300	-0.07690500
C	1.73570600	-0.10056400	0.01071900
C	0.42510300	-0.62108600	0.05483800
C	-0.62060800	0.30933000	-0.00417700
H	-1.28317200	2.34964000	-0.08998100
H	1.04780300	3.24069000	-0.17227600
H	2.95071000	1.65891900	-0.11842700
C	0.20885000	-2.10584800	0.19192600
H	1.03396300	-2.56056200	0.74285000
H	-0.71777500	-2.34208400	0.71510600
H	0.15947600	-2.58547400	-0.79098600
N	-2.01752100	-0.13061900	-0.02360600
O	-2.84371400	0.57123800	0.54510700
O	-2.29866900	-1.15601800	-0.62941400
C	2.93065100	-1.01860400	0.05378900
H	2.86995900	-1.79554700	-0.71494300
H	3.85355000	-0.45667000	-0.10688300
H	3.01412700	-1.52503900	1.02178800

1q

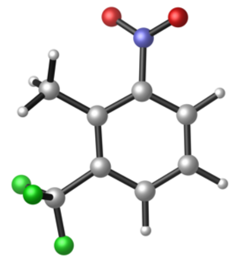

G = -812.766042

E = -812.865797178

C	-1.58464000	1.68237200	-0.07117200
C	-0.42745900	2.43984900	-0.12384400
C	0.80543300	1.79916500	-0.10064300
C	0.88406500	0.41110400	-0.01849800
C	-0.26772400	-0.39718100	0.03052100
C	-1.48335400	0.29593600	-0.01457800
H	-2.56301000	2.14744600	-0.08706300
H	-0.48386800	3.52038300	-0.18575700
H	1.71619300	2.38392200	-0.14289600
C	-0.18843900	-1.89815400	0.15268100
H	0.72225000	-2.20300900	0.66478700
H	-1.03452200	-2.29600700	0.71268500
H	-0.20014600	-2.36438000	-0.83575500
N	-2.75678200	-0.42994400	-0.01274300
O	-3.68569500	0.06192200	0.61212800
O	-2.83399200	-1.46673400	-0.65545500
C	2.25632400	-0.21913100	0.02315100
F	2.40737800	-1.16657600	-0.91998900
F	2.50949600	-0.80646500	1.21032700
F	3.23121900	0.68148300	-0.17432700

1r

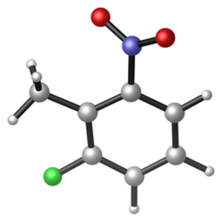

G = -575.034946

E = -575.125231862

C	-0.29398200	1.68458800	-0.07574300
C	1.03043400	2.09670000	-0.10916800
C	2.04803500	1.15078700	-0.06097200
C	1.71387700	-0.19105700	0.01576400
C	0.40468900	-0.67444000	0.04008700
C	-0.57804100	0.32347800	-0.01553800
H	-1.10620600	2.39999600	-0.10606000

H	1.26927400	3.15215100	-0.17234500
H	3.09403900	1.43555200	-0.08054400
C	0.15414500	-2.15297300	0.15110600
H	1.05441900	-2.65338500	0.50808000
H	-0.66331800	-2.36935200	0.84087800
H	-0.11403000	-2.57550600	-0.82042300
N	-1.99722900	-0.03955400	-0.01628400
O	-2.78553000	0.75157700	0.48282900
O	-2.32663900	-1.09876100	-0.53050000
F	2.71875900	-1.08417900	0.07250700

1s

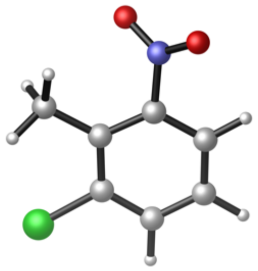

G = -935.407036

E = -935.494507564

C	0.94250600	1.67979400	0.06272300
C	-0.27601500	2.33823500	0.11071500
C	-1.45504900	1.60334100	0.08281300
C	-1.40065600	0.21622600	-0.00262200
C	-0.19831800	-0.50395000	-0.05314100
C	0.95577100	0.29102000	0.00155300
H	1.87847100	2.22417200	0.08893500
H	-0.31118700	3.41963300	0.17626700
H	-2.41604300	2.10332200	0.12458300
C	-0.18440400	-2.00112300	-0.19513400
H	-1.01447200	-2.32505600	-0.82519200
H	0.74287100	-2.36080000	-0.63819500
H	-0.29637100	-2.48159800	0.78185400
N	2.28287300	-0.33194800	0.02327000
O	3.18199000	0.23199000	-0.58381000
O	2.42972000	-1.35888700	0.67006900
Cl	-2.92706300	-0.64600700	-0.03545000

1t

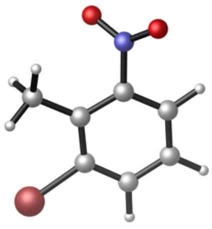

G = -488.408142

E = -488.493904310

C	1.66844300	1.64648300	0.06199300
C	0.52501600	2.42802700	0.11231100
C	-0.72320600	1.81827500	0.08828400
C	-0.81583800	0.43187200	0.00439300
C	0.30661900	-0.40862500	-0.04841700
C	1.53692600	0.26432600	0.00227200
H	2.65557600	2.09163100	0.08517900
H	0.60260400	3.50725700	0.17672900
H	-1.62388000	2.41991500	0.13157400
C	0.17924800	-1.90076600	-0.18825600
H	-0.68490700	-2.14730600	-0.80743400
H	1.06302400	-2.34570100	-0.64256600
H	0.03578700	-2.36979500	0.79019000
N	2.79462800	-0.49003000	0.02030900
O	3.73993300	-0.02651100	-0.60111700
O	2.84320300	-1.51814100	0.67980200
Br	-2.58111300	-0.31560400	-0.02002300

1u

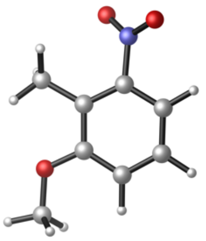

G = -590.282418

E = -590.410677966

C	0.86924400	1.68681100	0.06650800
C	-0.40534000	2.22142600	0.10897100
C	-1.52061800	1.38640300	0.07534600
C	-1.35824200	0.00341300	-0.00704600
C	-0.07415500	-0.58539300	-0.05081800
C	1.00260400	0.29997600	0.00562100
H	1.75140800	2.31366700	0.09534800
H	-0.54172000	3.29527500	0.17505100
H	-2.51077800	1.82364800	0.11321800
C	0.04012400	-2.07953300	-0.18750600
H	-0.82566500	-2.46544100	-0.72693000
H	0.94676700	-2.37211800	-0.71726400
H	0.06270800	-2.56078600	0.79516700
N	2.37703300	-0.20781100	0.02115100
O	3.23695700	0.46319000	-0.53396300

O	2.60571900	-1.25577000	0.60920900
O	-2.39021800	-0.86881400	-0.05040600
C	-3.71230000	-0.35738400	-0.01891600
H	-3.90994700	0.30008300	-0.87316100
H	-4.36961400	-1.22547200	-0.07518500
H	-3.90996400	0.18265700	0.91401500

1-NO_2

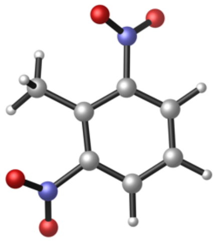

G = -680.247490

E = -680.345805757

C	-1.19609000	1.67800700	-0.16207900
C	-0.00055800	2.37583300	-0.24136000
C	1.19548300	1.67919700	-0.16059700
C	1.17286700	0.29557700	-0.02614700
C	0.00033800	-0.46866800	0.05486100
C	-1.17308200	0.29446800	-0.02645100
H	-2.14838500	2.19197000	-0.21350200
H	-0.00102700	3.45215300	-0.36467100
H	2.14754800	2.19381100	-0.21047100
C	0.00170900	-1.95437100	0.30005400
H	0.89748400	-2.25823000	0.84084400
H	-0.86924800	-2.25240400	0.88342500
H	-0.02236300	-2.49995400	-0.64606300
N	-2.48560500	-0.35837400	0.02239000
O	-3.37968500	0.22096400	0.62124400
O	-2.62290500	-1.42380200	-0.55941800
N	2.48548100	-0.35686900	0.02175500
O	2.62502900	-1.41610900	-0.57089300
O	3.37666900	0.21633300	0.63053300

1ae

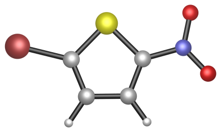

G = -769.910018

E = -769.935997240

C	-0.82680100	0.29643200	-0.00004800
C	-0.28812100	1.55460400	-0.00001100
C	1.12939400	1.51831000	-0.00009000
C	1.59571300	0.23581800	-0.00034300
S	0.35270600	-0.96588900	0.00047900
H	-0.87339800	2.46487700	0.00005600
H	1.77222600	2.38906800	-0.00008100
N	2.96577400	-0.16814900	-0.00015600
Br	-2.65927900	-0.12889800	-0.00006700
O	3.19391600	-1.37409500	-0.00056100
O	3.81997200	0.70631700	0.00040600

1ai

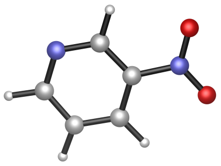

G = -452.579318

E = -452.640022686

C	-0.45161400	1.22078000	-0.00017700
C	-1.83724200	1.16087300	-0.00017800
C	-2.45211000	-0.08885600	0.00000600
C	-0.45348800	-1.19730000	0.00016000
C	0.23213100	0.01281300	-0.00001400
H	0.08519900	2.16154100	-0.00030900
H	-2.43310900	2.06595300	-0.00031400
H	-3.53645600	-0.16210500	0.00004800
N	1.69236900	0.00818100	-0.00000400
O	2.26615800	1.08720900	0.00043700
O	2.26066000	-1.07405500	-0.00043100
H	0.08869900	-2.13693100	0.00032200
N	-1.78450300	-1.24868800	0.00020700

2a

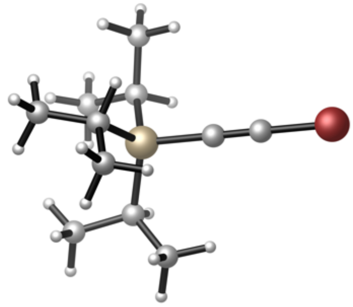

G = -734.093383

E = -734.344017649

Si	-1.00441300	0.08666800	-0.11599200
C	-1.55958800	-0.14190800	1.68375900
C	-1.45171500	1.78937100	-0.82377200
C	-1.52907100	-1.29700000	-1.31028100
H	-0.97898900	0.60782300	2.24122700
H	-0.77808800	1.90554200	-1.68561800
H	-1.45427500	-0.83852900	-2.30794400
C	-1.17316500	-1.52486100	2.22843200
C	-3.04650900	0.15450200	1.92727400
C	-0.59745100	-2.51763100	-1.28972400
C	-2.98692800	-1.73981800	-1.10565800
C	-1.13862000	2.91830200	0.16813700
C	-2.89006000	1.89124500	-1.35100900
H	-0.92229300	-3.25970000	-2.02959700
H	0.43841800	-2.24955800	-1.51931200
H	-0.60227100	-3.00983300	-0.31071800
H	-3.10899500	-2.26106500	-0.14963700
H	-3.69295400	-0.90328200	-1.11916900
H	-3.28935600	-2.43807800	-1.89582500
H	-3.29119800	0.02376900	2.98888800
H	-3.30932600	1.18182400	1.65577400
H	-3.69675800	-0.51817600	1.35856900
H	-1.38244600	-1.59218100	3.30321700
H	-1.74229900	-2.32135300	1.73590200
H	-0.10799700	-1.73777900	2.08549900
H	-3.62933700	1.75971000	-0.55282600
H	-3.06504800	2.87868200	-1.79625800
H	-3.09538100	1.14389600	-2.12447100
H	-0.10575400	2.86984900	0.53128700
H	-1.27836900	3.89972900	-0.30151400
H	-1.80081700	2.87797900	1.04075000
C	0.85399900	0.07310700	-0.05092300
C	2.06949600	0.07654600	-0.02098600
Br	3.87580000	0.07646400	0.00411900

2b

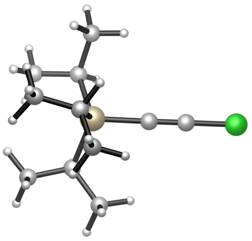

G = -1181.089375

E = -1181.34099213

Si	-0.39315100	0.08876500	-0.12415000
C	-0.91466900	-0.14227600	1.68582900
C	-0.88790400	1.77906400	-0.83003600
C	-0.91129600	-1.30898700	-1.30468100
H	-0.34364900	0.62603000	2.22802800
H	-0.23463300	1.90562200	-1.70599000
H	-0.86468700	-0.85203500	-2.30469900
C	-0.48456200	-1.51115100	2.23298600
C	-2.40397300	0.11904600	1.95304300
C	0.04525100	-2.51035400	-1.29857700
C	-2.35625300	-1.77953400	-1.07277900
C	-0.57744600	2.91730500	0.15189900
C	-2.33796600	1.85090700	-1.32924600
H	-0.27919300	-3.26192600	-2.02895400
H	1.07035400	-2.22169800	-1.55077600
H	0.07170400	-2.99848000	-0.31778400
H	-2.45206900	-2.30006900	-0.11341000
H	-3.07790400	-0.95631500	-1.07647400
H	-2.65905700	-2.48571300	-1.85572500
H	-2.62698100	-0.00953000	3.01970300
H	-2.69744500	1.13719100	1.67847500
H	-3.04688800	-0.57404500	1.40086600
H	-0.67613400	-1.57701200	3.31115200
H	-1.04018600	-2.32524900	1.75397100
H	0.58331600	-1.69827600	2.07532300
H	-3.05934900	1.70950300	-0.51674900
H	-2.54010600	2.83265100	-1.77550600
H	-2.54423900	1.09605800	-2.09515800
H	0.46501500	2.89344200	0.48915800
H	-0.75069800	3.89432500	-0.31574700
H	-1.21597700	2.86367000	1.04131100
C	1.46486000	0.11320800	-0.08143800
C	2.67839300	0.14286100	-0.04461000
Cl	4.32566600	0.17874200	0.00133800

2c

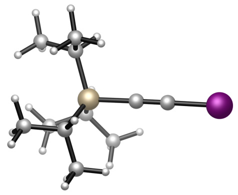

G = -732.312904

E = -732.561618357

Si	-1.52312300	0.08880600	-0.13052100
C	-2.02621900	-0.13406100	1.68670500
C	-2.00041300	1.78515800	-0.83403100
C	-2.08454400	-1.30103300	-1.30192400
H	-1.42968400	0.61774000	2.22429700
H	-1.37161900	1.88960900	-1.73064000
H	-2.04955200	-0.84374400	-2.30223500
C	-1.61933100	-1.51505100	2.22156100
C	-3.50515100	0.15993700	1.97624500
C	-1.14424400	-2.51507600	-1.31389300
C	-3.53151400	-1.75136400	-1.04478600
C	-1.63262300	2.92291200	0.12812300
C	-3.46235400	1.88772700	-1.29148300
H	-1.48835700	-3.26003900	-2.04217500
H	-0.11902200	-2.23859700	-1.57884400
H	-1.11019000	-3.00675000	-0.33508400
H	-3.61966200	-2.26610900	-0.08152600
H	-4.24213900	-0.91857400	-1.04139200
H	-3.85647000	-2.45700200	-1.81942200
H	-3.71889200	0.02057100	3.04348700
H	-3.77503600	1.18961000	1.72111300
H	-4.17191100	-0.50790500	1.42115100
H	-1.80145400	-1.58361000	3.30122400
H	-2.19619700	-2.31477800	1.74347400
H	-0.55707300	-1.72242800	2.05178900
H	-4.16375600	1.76768500	-0.45848700
H	-3.65463600	2.87130900	-1.73820900
H	-3.70768100	1.13359100	-2.04645300
H	-0.57845200	2.88048400	0.42456700
H	-1.80542600	3.90049700	-0.33860400
H	-2.23688900	2.88671200	1.04219400
C	0.33419300	0.07625600	-0.10737700
C	1.55183700	0.07104800	-0.06939500
I	3.54150900	0.04905100	0.00129500

2d

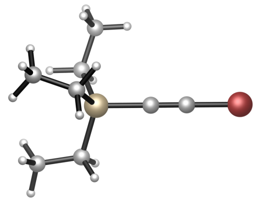

G = -616.264149

E = -616.430494637

Si	-1.32526400	-0.15238000	-0.05743300
C	-1.82424700	1.07740700	-1.39321800
C	-1.82557000	-1.90784300	-0.52534500
C	-1.98905700	0.38004900	1.62721500
H	-1.43342700	0.72170400	-2.35510200
H	-1.38370300	-2.60098300	0.20171600
H	-1.89082300	-0.46399600	2.32183200
C	-1.35402500	2.51280900	-1.12856500
C	-3.44074700	0.88023300	1.60570300
C	-3.34013300	-2.14093400	-0.61268500
H	-3.54967100	1.76309900	0.96662700
H	-4.13272400	0.11904100	1.23108800
H	-3.77630100	1.16043500	2.61005000
H	-1.67003200	3.19306000	-1.92668600
H	-1.75873900	2.90216500	-0.18757400
H	-0.26191100	2.56782600	-1.06186200
H	-3.81794400	-1.44809600	-1.31390700
H	-3.56428700	-3.15736900	-0.95376200
H	-3.82199100	-2.01113100	0.36188600
C	0.52844700	-0.13831400	0.03886900
C	1.74366800	-0.12482400	0.07305500
Br	3.54953900	-0.11203900	0.10722800
H	-1.35554000	-2.14080000	-1.48950000
H	-2.91828000	1.04995500	-1.48395800
H	-1.33480700	1.16825800	2.02004200

2f

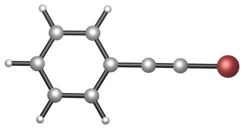

G = -320.795253

E = -320.863424213

C	-0.06679800	0.00037400	-0.00021500
C	-1.27544900	0.00023900	-0.00019600
Br	-3.08017800	-0.00005500	0.00006500
C	1.36700300	0.00023500	-0.00015600
C	2.07156800	-1.21271900	-0.00006600

C	2.07199700	1.21290800	-0.00006300
C	3.46175600	-1.20730100	0.00007100
H	1.52520900	-2.15033700	-0.00013900
C	3.46220000	1.20697100	0.00006900
H	1.52601500	2.15074500	-0.00013800
C	4.15879000	-0.00028500	0.00013700
H	4.00167000	-2.14887800	0.00011900
H	4.00244600	2.14835800	0.00011600
H	5.24447600	-0.00048900	0.00028000

3a

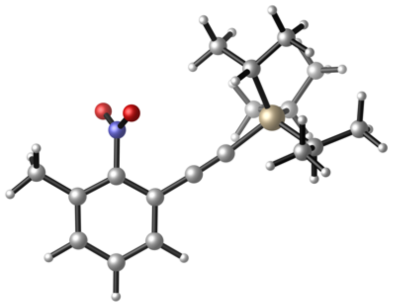

G = -1196.143817

E = -1196.50698265

C	-2.26877500	0.99425800	0.27195900
C	-2.87721400	2.20739000	0.61316400
C	-4.26179200	2.31776300	0.59551300
C	-5.05569900	1.22736100	0.25302500
C	-4.49340000	-0.00901800	-0.07570600
C	-3.10233100	-0.08205300	-0.06233600
H	-2.25384800	3.05153300	0.88584600
H	-4.72830700	3.26362500	0.84965700
H	-6.13638600	1.32929200	0.23784100
C	-5.35250600	-1.19194200	-0.43283100
H	-6.38557600	-0.87460500	-0.58610000
H	-5.00680300	-1.67794400	-1.35038300
H	-5.33671900	-1.94036900	0.36449600
N	-2.46581600	-1.35370600	-0.42654700
O	-1.81738900	-1.38652000	-1.45960600
O	-2.62998400	-2.30258800	0.32469300
Si	2.16437800	0.28182100	0.12032200
C	2.20205400	-1.14272100	-1.13538300
C	2.95257400	1.87737000	-0.54152600
C	2.83632800	-0.14241900	1.84707600
H	1.55949000	-0.78686800	-1.95396000
H	2.54435800	2.66918700	0.10411200
H	3.04953400	0.83335200	2.30894700
C	1.56564400	-2.42405800	-0.57710900
C	3.58847700	-1.43163300	-1.72849800
C	1.82020500	-0.86803200	2.74142900
C	4.15352400	-0.93260400	1.78910300
C	2.50275700	2.17183000	-1.97908700
C	4.48130600	1.92611100	-0.41000300
H	2.23997700	-1.03521700	3.74129800
H	0.89370200	-0.29769300	2.85932000
H	1.55389100	-1.84827000	2.33044200
H	3.99025800	-1.94037400	1.39151500
H	4.91067300	-0.44930600	1.16351500
H	4.58016100	-1.04523300	2.79358900
H	3.52236500	-2.22319500	-2.48569400
H	4.02137100	-0.55189000	-2.21485100
H	4.29631500	-1.77263000	-0.96552800
H	1.48069100	-3.18569600	-1.36219900
H	2.17422700	-2.85325200	0.22679400
H	0.55966900	-2.24920500	-0.18139600
H	4.96906400	1.12915600	-0.98226800
H	4.86985000	2.88029400	-0.78786900
H	4.80340500	1.83354800	0.63251000
H	1.41091300	2.17522900	-2.07273600
H	2.86630100	3.15272400	-2.30978400
H	2.89242800	1.42677000	-2.68237500
C	0.35595000	0.66525500	0.28458800
C	-0.84753600	0.84220000	0.28427000

3a-Ph

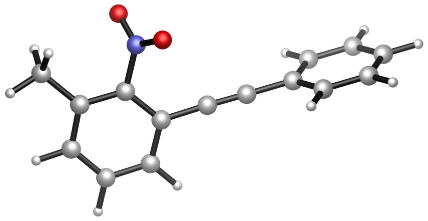

G = -782.845874

E = -783.025470684

C	1.24697800	0.84573400	0.01202800
C	1.72415700	2.15964900	-0.06140000
C	3.09056100	2.40473300	-0.08937900
C	3.99741800	1.35137600	-0.05582200
C	3.57072900	0.02146400	-0.00589800
C	2.19187100	-0.19183900	0.03336400
H	1.01066100	2.97523300	-0.09407600
H	3.45245500	3.42641100	-0.13725000
H	5.06374700	1.55468200	-0.06994900
C	4.57221200	-1.10251700	0.01860500

H	5.55679200	-0.71525000	0.28834100
H	4.30083400	-1.87737300	0.74059700
H	4.64863000	-1.57815300	-0.96299200
N	1.69932200	-1.57032500	0.10360700
O	0.97218200	-1.86841900	1.03745100
O	2.05207700	-2.34255700	-0.77576700
C	-1.36307000	0.44469200	0.00193800
C	-0.16296900	0.60118200	0.01941500
C	-2.78273700	0.24555600	-0.01957700
C	-3.44241800	0.01944200	-1.23657400
C	-3.51955000	0.27322300	1.17372300
C	-4.81907800	-0.17395400	-1.25514300
H	-2.87145800	-0.00238300	-2.15920200
C	-4.89597200	0.07894900	1.14438800
H	-3.00816800	0.44508400	2.11532300
C	-5.54754600	-0.14499800	-0.06716500
H	-5.32396000	-0.34757300	-2.20019500
H	-5.46016600	0.10084900	2.07146100
H	-6.62220600	-0.29759300	-0.08579100

3a-TES

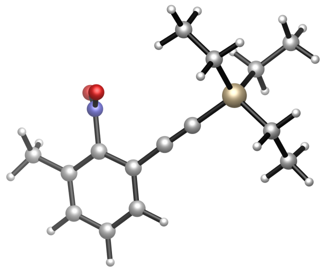

G = -1078.315762

E = -1078.59447431

C	-1.71051200	0.87122000	-0.12208800
C	-4.45064900	1.41990100	-0.01853900
C	-0.30533700	0.60715000	-0.15467000
C	0.89671500	0.42136000	-0.17033900
C	-2.66325000	-0.14488400	0.04756400
C	-4.03669800	0.09100100	0.11071300
C	-2.17264700	2.18882600	-0.22224900
C	-3.53467800	2.45410800	-0.17627400
H	-5.51401800	1.63723900	0.00732200
H	-3.88614400	3.47653700	-0.26660200
H	-1.45147400	2.99012100	-0.33743500
Si	2.72305900	0.11501900	-0.21854100
C	3.04014900	-1.24191500	-1.48511400
H	2.68246000	-0.88833600	-2.46073400
C	3.55643600	1.72507000	-0.72904200
H	3.19149900	1.99500400	-1.72827800
C	3.25722000	-0.42128600	1.50648900
H	2.98905300	0.36636300	2.22142200
O	-1.53311300	-1.98235400	-0.76234000
O	-2.47852200	-2.14226300	1.17687500
N	-2.18580000	-1.52637300	0.16315800
C	2.38577900	-2.58417200	-1.13637800
C	4.75794000	-0.72901800	1.59782400
C	3.33068800	2.88569900	0.24757400
H	3.75740500	2.66926000	1.23290700
H	3.79461700	3.80962500	-0.11406000
H	2.26276400	3.08633200	0.38918800
H	2.78703400	-2.99373800	-0.20276600
H	1.30256800	-2.47979100	-1.01023300
H	2.55659600	-3.32950100	-1.92070900
H	5.04340300	-1.54236500	0.92140600
H	5.36513600	0.14456900	1.33458500
H	5.04243000	-1.03114800	2.61144400
C	-5.04418300	-1.01245700	0.29315000
H	-4.80795700	-1.88713700	-0.31969500
H	-5.07874600	-1.33967500	1.33605700
H	-6.03814100	-0.65869200	0.01219200
H	4.63118200	1.53215600	-0.84445300
H	4.12622100	-1.36577100	-1.58944200
H	2.67313200	-1.30338700	1.79735400

3b

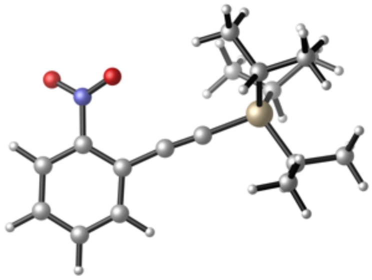

G = -1156.860310

E = -1157.19816694

C	-2.51678400	0.72746700	0.06870800
C	-2.96231600	2.04203500	0.28157100
C	-4.31618500	2.34653400	0.32232600
C	-5.26879900	1.34258600	0.16455800
C	-4.85920300	0.03113800	-0.03227900
C	-3.50195600	-0.26368100	-0.08652600
H	-2.22055500	2.82157100	0.41522900
H	-4.62700800	3.37376300	0.48103300
H	-6.32728000	1.57582300	0.19556400
N	-3.13954700	-1.66256100	-0.31742500

O	-2.04783700	-1.90837700	-0.80584700
O	-3.96384100	-2.51721300	-0.01957800
Si	1.95160600	0.21469500	0.12460200
C	2.35865900	-0.66402500	-1.50974600
C	2.62731500	1.98740700	0.20068300
C	2.41700000	-0.75034000	1.69693600
H	1.81113300	-0.08394600	-2.26722400
H	2.04718100	2.46140700	1.00663300
H	2.46787000	0.01529500	2.48567100
C	1.80831900	-2.09737900	-1.53902400
C	3.84426200	-0.63500600	-1.89528300
C	1.36265900	-1.78401100	2.11865100
C	3.80274300	-1.40772200	1.59564300
C	2.33603500	2.75657800	-1.09530400
C	4.10868500	2.08357500	0.59146300
H	1.66338100	-2.28301400	3.04838800
H	0.38377700	-1.32529500	2.28727200
H	1.23412100	-2.56103100	1.35650000
H	3.80203300	-2.21542600	0.85598100
H	4.58660100	-0.69858400	1.31112800
H	4.09042200	-1.84842600	2.55815800
H	3.99534200	-1.12363400	-2.86621400
H	4.22712400	0.38657600	-1.98276800
H	4.46730700	-1.16381800	-1.16657500
H	1.93834200	-2.54259100	-2.53346700
H	2.33389400	-2.74168600	-0.82505200
H	0.74054200	-2.13234600	-1.29851400
H	4.76105800	1.59709700	-0.14194000
H	4.42095800	3.13346500	0.65722700
H	4.30176100	1.62394300	1.56637900
H	1.27096900	2.72973100	-1.35280600
H	2.62568600	3.81015000	-0.99848400
H	2.89319200	2.34122100	-1.94309000
C	0.10611500	0.40151500	0.11192200
C	-1.10659200	0.49246800	0.06972800
H	-5.57982000	-0.76816800	-0.15225100

3b'

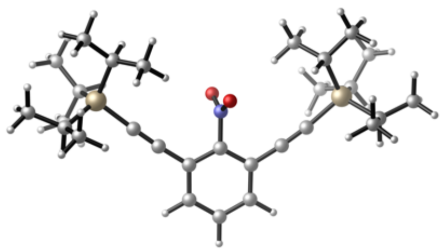

G = -1877.182399

E = -1877.78424617

C	1.25957300	2.36043100	-0.02202600
C	1.19288400	3.75929700	-0.04994200
C	-0.03876300	4.40372000	-0.05101800
C	-1.22380200	3.67805700	-0.01796400
C	-1.19574700	2.27777700	0.02125000
C	0.05408400	1.65501600	0.01281400
H	2.11552100	4.32811100	-0.07049500
H	-0.07601700	5.48726000	-0.07812800
H	-2.18280900	4.18342400	-0.02155500
N	0.10593700	0.18821100	0.04420900
O	0.35727200	-0.38465800	-1.00258300
O	-0.10283900	-0.35695500	1.11437600
Si	4.93087400	-0.22343600	0.03126300
C	4.16963100	-1.84311100	-0.60445500
C	6.21282100	0.50720000	-1.16109600
C	5.60843600	-0.28680200	1.80630700
H	3.64280900	-1.54378200	-1.52230300
H	6.28943200	1.56505000	-0.86858100
H	6.30114500	0.56585000	1.86979000
C	3.12188300	-2.41359100	0.36270000
C	5.19824900	-2.91562800	-0.99210800
C	4.53048400	-0.09067300	2.88263500
C	6.41633500	-1.56500200	2.08455600
C	5.71845700	0.46094000	-2.61378100
C	7.61304900	-0.10741800	-1.02324100
H	4.98020900	-0.10697200	3.88320700
H	4.00708400	0.86335500	2.76798500
H	3.77769800	-0.88616200	2.84975100
H	5.76582200	-2.44663300	2.10258600
H	7.19698700	-1.74360700	1.33789000
H	6.90651000	-1.50194400	3.06387200
H	4.69244700	-3.79785900	-1.40405400
H	5.90175800	-2.55903400	-1.75096400
H	5.78226400	-3.25112300	-0.12850000
H	2.60932000	-3.27215100	-0.08857200
H	3.58654300	-2.76359800	1.29150200
H	2.35483200	-1.67867500	0.62786500
H	7.61422900	-1.18031300	-1.24524300
H	8.31218600	0.36868000	-1.72214000
H	8.01931500	0.02531500	-0.01501700
H	4.73453700	0.93069200	-2.72537700
H	6.41272200	0.98823600	-3.27945700
H	5.63718800	-0.56996000	-2.97735700
C	3.50290500	0.96397000	0.01991100
C	2.49816300	1.64848000	-0.00817900

C	-2.38797700	1.49075500	0.04829900
C	-3.35716000	0.75631200	0.06536300
Si	-4.79839200	-0.41603600	0.08850500
C	-5.31454400	-0.52179100	1.91159100
H	-4.36435000	-0.65865800	2.44847600
C	-4.14989100	-2.09379800	-0.52957000
H	-3.70728500	-2.56147500	0.36276400
C	-6.11232600	0.40490700	-1.00770100
H	-6.18616600	1.42841200	-0.61074400
C	-7.50810100	-0.22668400	-0.90388800
H	-8.22519000	0.33229100	-1.51815500
H	-7.51546900	-1.26237700	-1.25907600
H	-7.88609800	-0.22296000	0.12337800
C	-5.65807900	0.50650000	-2.47099200
H	-6.37182200	1.09576300	-3.06001800
H	-4.67813400	0.98754200	-2.56405600
H	-5.58857500	-0.48232800	-2.93801700
C	-5.27602000	-3.01302900	-1.02969900
H	-5.72254300	-2.62453800	-1.95170200
H	-4.88198200	-4.01176600	-1.25439500
H	-6.08021500	-3.13576400	-0.29705500
C	-3.04489500	-1.97973200	-1.58986400
H	-2.69781300	-2.97661500	-1.88946200
H	-3.40429700	-1.47505200	-2.49383200
H	-2.17545700	-1.42608400	-1.22344100
C	-5.94127100	0.79131000	2.40041600
H	-6.11829700	0.75982500	3.48253200
H	-5.29562500	1.65308600	2.19674800
H	-6.90784500	0.97693900	1.91747300
C	-6.20454500	-1.72691300	2.24646800
H	-6.42534800	-1.75248600	3.32091200
H	-7.16400900	-1.68810700	1.71893700
H	-5.71997900	-2.67477300	1.99074700

I

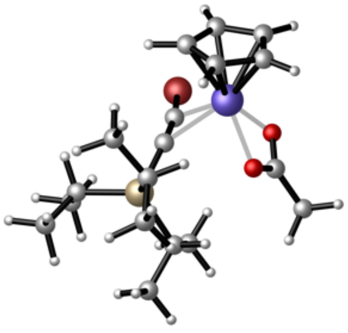

G = -1265.252769

E = -1265.63043123

C	0.58736200	-1.38702800	-0.52532500
C	-0.34720900	-0.58390500	-0.42051100
C	1.74693800	2.45676300	-1.15706300
C	1.89892900	1.32878400	-1.99908700
C	2.79836100	2.41871800	-0.17166000
H	1.24265900	1.04424500	-2.80997700
H	2.94864200	3.13371900	0.62565800
C	3.03125200	0.57613000	-1.54133400
H	3.41039500	-0.33634300	-1.97893500
C	3.60854000	1.28231800	-0.44301300
H	4.45157700	0.94964600	0.14848200
H	0.95400100	3.19041000	-1.21267800
Si	-2.07673000	0.15086600	-0.19488100
C	-2.54285000	-0.18862300	1.61316000
H	-2.07087500	0.63313900	2.16879100
C	-1.92597700	1.99728600	-0.57663500
H	-1.00887400	2.30617900	-0.05788700
C	-3.09047100	-0.79103900	-1.49217600
H	-2.53584400	-0.65169700	-2.43157600
O	0.43298000	1.19623600	1.78749700
C	1.02267100	0.29677100	2.46690300
O	1.91696700	-0.37852100	1.86229500
C	0.69985800	0.06056700	3.90136100
H	-0.34083600	0.32083200	4.10029300
H	0.89355800	-0.97954200	4.16627400
H	1.34273600	0.70258600	4.51255100
Rh	1.56923800	0.66251700	0.04746300
C	-4.06185900	-0.11236400	1.84011000
C	-1.99683900	-1.51265800	2.16738700
C	-3.16448500	-2.29729400	-1.20558900
C	-4.49791700	-0.20753800	-1.69638000
C	-3.07673700	2.83828400	-0.00579000
C	-1.73821300	2.25585300	-2.07778000
H	-4.28575500	-0.15201600	2.91289100
H	-4.50717800	0.80619900	1.44553400
H	-4.57198800	-0.96057200	1.37072400
H	-3.16258300	2.72734900	1.07990800
H	-2.91079200	3.90178800	-0.21589700
H	-4.04093000	2.56592200	-0.44896700
H	-2.65410000	2.03865200	-2.63788100
H	-1.48730000	3.30699800	-2.26417800
H	-0.94161300	1.64068500	-2.50959000
H	-2.45185800	-2.37324400	1.66490900
H	-0.91310600	-1.60664600	2.05082200
H	-2.22478300	-1.60249500	3.23612000
H	-5.11971900	-0.33181400	-0.80462900

H	-4.47666400	0.85871100	-1.94111200
H	-5.00445200	-0.72376400	-2.52094000
H	-3.66396000	-2.82076900	-2.02959300
H	-2.17436800	-2.75027100	-1.08180200
H	-3.73865500	-2.49883700	-0.29464900
Br	1.57124300	-2.89692400	-0.71165700

I^{Cp*}

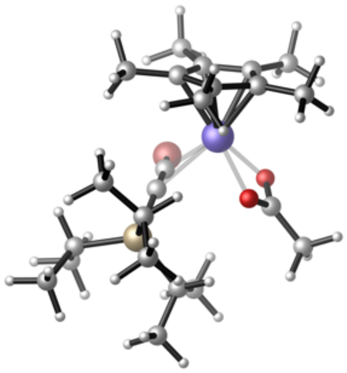

G = -1461.701755

E = -1462.21288893

C	0.06406600	1.60767200	-0.37521300
C	0.84857200	0.65414600	-0.38127100
C	-2.04100300	-1.97327400	-0.95046400
C	-2.00370800	-0.84384800	-1.81794200
C	-2.89671900	-1.64531500	0.17665700
C	-2.83831400	0.18605700	-1.24614700
C	-3.42825600	-0.33172700	-0.04483600
Si	2.47559400	-0.28777300	-0.20187700
C	3.00405800	-0.03351900	1.60517200
H	2.47434700	-0.82582000	2.15167900
C	2.15055400	-2.10435900	-0.61244100
H	1.18692300	-2.32059700	-0.13298500
C	3.57877100	0.56005400	-1.49324300
H	3.01336600	0.47226300	-2.43270300
O	-0.10289900	-1.21553200	1.77079400
C	-0.43724400	-0.28722200	2.57138800
O	-1.19393300	0.63264900	2.12760400
C	0.02208700	-0.29338400	3.99319800
H	1.02289800	-0.72283300	4.06579600
H	0.00817200	0.71564700	4.40624900
H	-0.66056700	-0.92033400	4.57638000
Rh	-1.26853100	-0.28378700	0.17305200
C	4.51411900	-0.24866000	1.79911600
C	2.58502200	1.31731700	2.20315900
C	3.78825500	2.05347000	-1.20729900
C	4.93009700	-0.14299400	-1.70325600
C	3.18686500	-3.06420300	-0.01092500
C	2.00026600	-2.32606900	-2.12292500
H	4.76024100	-0.25995700	2.86783700
H	4.86960600	-1.19082900	1.37018900
H	5.08857200	0.56441200	1.34191200
H	3.24955700	-2.96839000	1.07785600
H	2.91923200	-4.10423500	-0.23459800
H	4.18846600	-2.89306800	-0.42150800
H	2.94967400	-2.17567300	-2.64773900
H	1.67409000	-3.35118500	-2.33654400
H	1.26959600	-1.64230400	-2.56625900
H	3.08064600	2.15095500	1.69314200
H	1.50754700	1.49181700	2.13583700
H	2.86577600	1.36964900	3.26210000
H	5.57250900	-0.05307400	-0.82217000
H	4.82062900	-1.20897000	-1.92532900
H	5.46563200	0.31435000	-2.54404900
H	4.33789300	2.53037100	-2.02772300
H	2.84114300	2.59123000	-1.09049100
H	4.37116900	2.20282800	-0.29185600
Br	-0.59563600	3.29594500	-0.32857500
C	-1.29524700	-0.70932700	-3.12241500
C	-3.18771100	1.47132800	-1.91214500
C	-4.37292800	0.40125700	0.83924000
C	-3.23688800	-2.55756100	1.30559800
C	-1.35295700	-3.27959500	-1.13406000
H	-3.46169900	2.24138300	-1.18951200
H	-2.36889000	1.83786500	-2.53385500
H	-4.05205400	1.30018700	-2.56355200
H	-5.38798900	0.27669400	0.44550500
H	-4.35131800	0.01557500	1.85923100
H	-4.14821300	1.46964000	0.85921000
H	-4.17867900	-3.07276400	1.08881300
H	-2.46033200	-3.31024200	1.45071400
H	-3.35922800	-2.00363500	2.23865600
H	-2.09808100	-4.03613700	-1.40269400
H	-0.60465100	-3.23468500	-1.92476200
H	-0.86925400	-3.60456700	-0.20964300
H	-2.03146300	-0.50778800	-3.90704400
H	-0.58949100	0.12598100	-3.10355600
H	-0.75885100	-1.62098400	-3.38432400

II

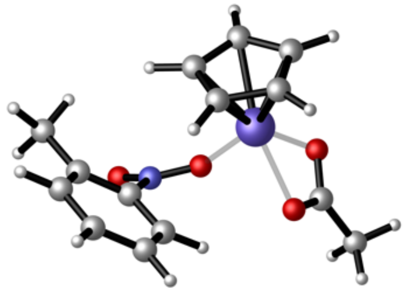

G = -1006.971430

E = -1007.19510544

O	1.69569300	-1.19726900	1.13753100
C	2.58402400	-1.54270500	0.29265900
O	2.76717300	-0.77147100	-0.70184700
C	-1.98197600	-0.83403800	-0.13758400
C	-1.47419400	-1.26863300	1.08539200
C	-3.15818700	-0.08663600	-0.27757900
C	-2.17584200	-0.96064100	2.24004600
C	-3.81799500	0.22560300	0.91460700
C	-3.34608500	-0.20811400	2.14987300
H	-0.55406100	-1.84037500	1.12369900
H	-1.80970000	-1.30541900	3.20042900
H	-4.72497100	0.81957200	0.86326600
H	-3.89625600	0.04523200	3.05029300
Rh	1.13408600	0.46918700	-0.09901600
C	-0.21166800	2.13917100	-0.42916000
H	-1.15923300	2.01863100	-0.93878400
C	1.04288000	2.39562300	-1.05763100
H	1.21024600	2.54513800	-2.11510200
C	2.04415400	2.39748800	-0.03254000
H	3.10809000	2.52313500	-0.18393900
C	0.00115700	1.95466600	0.96392900
H	-0.75355200	1.70169100	1.69571300
C	1.40582600	2.12098900	1.21267000
H	1.90134200	2.00945700	2.16776000
C	3.35063000	-2.81222900	0.43746800
H	3.23167300	-3.22202000	1.44053200
H	2.97304600	-3.53301900	-0.29456700
H	4.40613500	-2.63398300	0.22137700
O	0.02945300	-1.05968600	-1.29963800
O	-1.78471400	-1.60616100	-2.29963900
C	-3.69395000	0.40117300	-1.59561700
H	-2.90405100	0.74646800	-2.26830900
H	-4.38789700	1.22695100	-1.42778400
H	-4.23322800	-0.39754500	-2.11268000
N	-1.21641600	-1.18155900	-1.32237900

II_{Br}^{Cp*}

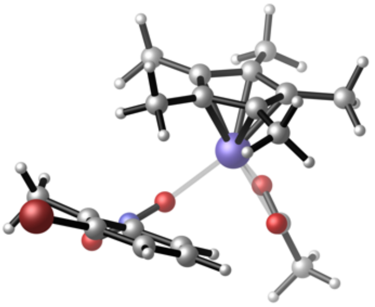

G = -1216.008987

E = -1216.35413676

O	2.00650600	1.73254700	-1.23802600
C	2.87824800	2.24495300	-0.46456100
O	3.24201300	1.57319700	0.54845500
C	-1.57474000	1.20378500	0.32767500
C	-1.10630200	1.45123200	-0.95681200
C	-2.80382400	0.59870400	0.62828800
C	-1.90775000	1.09504800	-2.03032300
C	-3.57162000	0.26557500	-0.49637800
C	-3.14487600	0.50413100	-1.80061100
H	-0.14376100	1.92769700	-1.10825200
H	-1.57650600	1.28635400	-3.04435900
H	-3.77749100	0.22880800	-2.63677200
Rh	1.81062700	0.00806000	0.10067100
C	1.64995500	-1.88194500	1.10253000
C	2.96254900	-1.74209000	0.55068900
C	2.82716100	-1.54327900	-0.87385400
C	0.69987900	-1.85462500	0.01157200
C	1.41965600	-1.65625900	-1.20084800
C	3.42251600	3.61333300	-0.71892700
H	3.53702100	3.78286900	-1.79104200
H	2.70779700	4.34720900	-0.33188100
H	4.37620500	3.74915800	-0.20796500
O	0.50754600	1.40782400	1.36231200
O	-1.20206800	2.23763000	2.35054400
C	-3.24175400	0.26280000	2.02621400
H	-2.40478000	0.21378200	2.72281000
H	-3.74923200	-0.70337100	2.03691700
H	-3.94408800	1.01369500	2.40161700
N	-0.71181300	1.63571200	1.42145700
C	1.30722400	-2.09198400	2.53743300
C	4.23647400	-1.73207400	1.31845500
C	-0.76777500	-2.06164600	0.14754100
C	0.84638300	-1.53542600	-2.56936800
C	3.93550100	-1.35678500	-1.85136600

H	-1.14072600	-1.66302600	1.09267300
H	-1.31831800	-1.59804500	-0.67225700
H	-0.97186100	-3.13799700	0.13287700
H	0.39704500	-1.55332000	2.81046400
H	1.13427400	-3.15854300	2.71672800
H	2.11427700	-1.75938500	3.19162700
H	4.10526600	-1.27527900	2.30125800
H	4.56620100	-2.76643400	1.46562200
H	5.02224500	-1.19633500	0.78446400
H	4.77691400	-0.82863200	-1.39930500
H	4.28764200	-2.33673600	-2.19148100
H	3.59896300	-0.79388300	-2.72377100
H	-0.22437200	-1.32926100	-2.53871900
H	1.34026900	-0.74060300	-3.13281400
H	1.00233000	-2.47741300	-3.10623100
Br	-5.28697900	-0.55297900	-0.27322700

II_{CF3}^{Cp*}

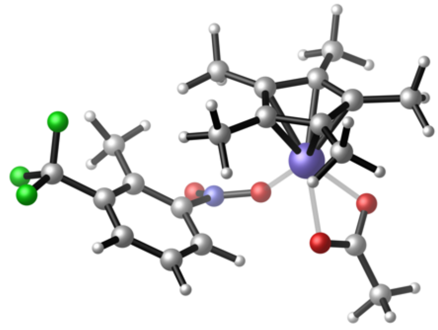

G = -1540.368002

E = -1540.72597331

O	1.89006100	1.85659800	-1.09864200
C	2.82111100	2.27019200	-0.33573700
O	3.23439400	1.49214900	0.57642600
C	-1.56713300	1.19853400	0.60828900
C	-1.14329700	1.82886600	-0.55851600
C	-2.76413800	0.48076000	0.72916100
C	-1.94966100	1.76411100	-1.68028000
C	-3.53326400	0.41307900	-0.44757500
C	-3.14038600	1.05003400	-1.62218600
H	-0.19858400	2.35904100	-0.58084200
H	-1.65117700	2.26246000	-2.59494900
H	-3.76849300	0.98409700	-2.50210900
Rh	1.73433500	0.00422400	0.07344600
C	0.80819700	-1.93470200	0.45603000
C	2.18390800	-1.93307600	0.86370700
C	2.98795200	-1.66368200	-0.30438700
C	0.74224500	-1.61601100	-0.93825200
C	2.09711300	-1.45599600	-1.41489600
C	3.38289000	3.64681000	-0.49376800
H	3.53802500	3.86730200	-1.55195000
H	2.65804200	4.36753900	-0.10163200
H	4.31913300	3.74815100	0.05544600
O	0.53827700	1.30194600	1.59726800
O	-1.17943900	1.60571800	2.84552000
C	-3.19715900	-0.18621500	2.00877300
H	-2.34799200	-0.44623900	2.63904200
H	-3.75431000	-1.09967200	1.80794900
H	-3.84017300	0.48710300	2.58222800
N	-0.69286400	1.36320200	1.76407600
C	-0.34662000	-2.19940500	1.35658700
C	2.68633600	-2.22050200	2.23641500
C	-0.48675700	-1.52747300	-1.77510000
C	2.49765800	-1.15292300	-2.81476100
C	4.47327800	-1.60785300	-0.35005000
H	-1.37067400	-1.32446000	-1.16851400
H	-0.39669700	-0.74211900	-2.52814800
H	-0.64039300	-2.47935800	-2.29464100
H	-1.29266900	-1.95514600	0.87440300
H	-0.35594300	-3.26574700	1.60698100
H	-0.26197300	-1.64297500	2.29386300
H	1.95104600	-1.92956100	2.98916200
H	2.87548700	-3.29431400	2.34063400
H	3.61868600	-1.68921300	2.43713900
H	4.88873500	-1.26876000	0.60018300
H	4.85629400	-2.61491600	-0.54905100
H	4.82283000	-0.94590400	-1.14384000
H	1.71107100	-0.61276300	-3.34360100
H	3.41457200	-0.56194800	-2.84776600
H	2.67944600	-2.09634300	-3.34135500
C	-4.80468200	-0.40242600	-0.47240800
F	-4.54632900	-1.71718000	-0.30736700
F	-5.66165500	-0.04588300	0.49825000
F	-5.45881500	-0.28168800	-1.63566800

II_{Cl}^{Cp*}

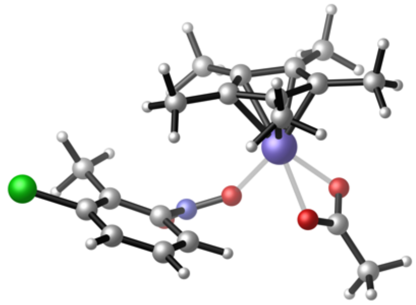

G = -1663.008099

E = -1663.35441771

O	1.49882000	1.92035800	-1.00032100
C	2.33480200	2.38086300	-0.15693900
O	2.74022100	1.60583400	0.76153100
C	-2.04158300	0.99751200	0.50013300
C	-1.63010800	1.67363700	-0.64288800
C	-3.19228400	0.20308300	0.58765400
C	-2.42088200	1.58977000	-1.77821600
C	-3.94467600	0.14988300	-0.59305400
C	-3.58447200	0.82888900	-1.75266000
H	-0.71460900	2.25410300	-0.63593500
H	-2.13765400	2.12074600	-2.67956600
H	-4.21245700	0.75732700	-2.63349700
Rh	1.39949700	0.03298000	0.08374900
C	0.97813600	-1.98430500	0.70997700
C	2.38795600	-1.79207600	0.60653700
C	2.68293700	-1.39802400	-0.75883600
C	0.39058800	-1.75100600	-0.59168000
C	1.44333300	-1.41147700	-1.49661000
C	2.79486900	3.80147600	-0.21994400
H	2.57829000	4.23767900	-1.19530600
H	2.26820600	4.37172500	0.55212100
H	3.86436400	3.85811800	-0.00758600
O	0.02393400	1.14570900	1.56359100
O	-1.74437400	1.41687500	2.74503400
C	-3.57078900	-0.59519300	1.80366900
H	-2.74317800	-0.70536500	2.50325900
H	-3.90513500	-1.59186700	1.50779700
H	-4.39538800	-0.11317600	2.33784400
N	-1.21168900	1.18227000	1.68332600
C	0.21744400	-2.34409500	1.93965900
C	3.39310300	-1.93067100	1.69524500
C	-1.03986600	-1.93892300	-0.96077900
C	1.26878500	-1.08157300	-2.93663900
C	4.03380700	-1.12153700	-1.32076300
H	-1.68452700	-1.92855300	-0.08181700
H	-1.37984500	-1.16676700	-1.65438900
H	-1.15399900	-2.91021100	-1.45412100
H	-0.77502600	-1.88695600	1.93308600
H	0.08697900	-3.43050100	1.98241400
H	0.73999500	-2.02311500	2.84210200
H	2.93050100	-1.85101500	2.67980400
H	3.87152200	-2.91270000	1.61522900
H	4.17119600	-1.16877000	1.61357000
H	4.72035400	-0.76793400	-0.55020500
H	4.44217300	-2.04445800	-1.74658500
H	3.98487300	-0.37282100	-2.11406500
H	0.30863800	-0.59156100	-3.11243400
H	2.06820600	-0.43510000	-3.30130700
H	1.28769600	-2.01186700	-3.51517800
Cl	-5.40424400	-0.81262000	-0.63524100

$II_F{}^{Cp*}$

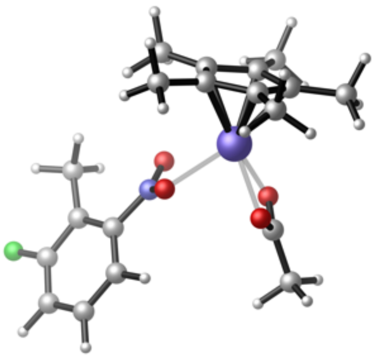

G = -1302.639710

E = -1302.98609836

C	-2.26216200	-1.33326800	1.30934300
C	-1.84524300	-1.91923700	0.05220900
C	-2.56982300	-1.28003700	-0.99727100
C	-3.25806900	-0.34581700	1.02792700
C	-3.41899100	-0.26744200	-0.40397400
C	3.19576500	1.31130500	0.84459900
C	4.49996900	1.69237000	1.11791100
C	5.55542700	0.87443800	0.72778000
C	5.28358400	-0.31197900	0.06621900
C	3.99945900	-0.75302300	-0.25345200
C	2.97904400	0.11550300	0.16112200
H	2.35561200	1.93148700	1.13137400
H	4.69579600	2.62619900	1.63168400
H	6.58666300	1.14118600	0.93005800
C	3.80791000	-2.07369200	-0.94415400
H	4.68985800	-2.69639100	-0.79525700
H	2.93528400	-2.60566100	-0.56029900

H	3.66518300	-1.93137500	-2.01868500
N	1.60010700	-0.20895800	-0.14744000
O	0.74353900	0.19375600	0.66434500
O	1.32909000	-0.82088500	-1.15591300
C	-0.73773300	2.52797600	-0.64105100
C	-0.20913800	3.87799200	-1.01049300
H	0.87157300	3.88792500	-0.83419500
H	-0.38286600	4.07556300	-2.06962100
H	-0.66956400	4.65315500	-0.39677700
Rh	-1.41471100	0.18752600	0.06112800
O	-1.20892600	2.30323600	0.51729200
O	-0.68685900	1.56240200	-1.46498600
C	-4.36897400	0.61666800	-1.13739500
C	-3.95758300	0.50653300	2.02720700
C	-1.76932000	-1.74123400	2.65577100
C	-0.84752500	-3.01702500	-0.09296400
C	-2.44979200	-1.55384500	-2.45551400
H	-2.32857400	-2.61891000	2.99721800
H	-0.71110100	-2.00849000	2.62363500
H	-1.90196400	-0.94400200	3.38912800
H	-1.35279500	-3.97918200	0.04396100
H	-0.38245400	-3.00746700	-1.07887000
H	-0.06314100	-2.93962500	0.66322400
H	-4.86286300	-0.01144700	2.36236700
H	-3.33049000	0.69470600	2.90043100
H	-4.25350500	1.46346200	1.59480700
H	-5.34641000	0.12676800	-1.20159100
H	-4.49760500	1.57134800	-0.62393700
H	-4.02045200	0.81407300	-2.15261000
H	-2.48864600	-0.62895800	-3.03448800
H	-1.51939400	-2.07265900	-2.69005000
H	-3.28695000	-2.18625400	-2.77046900
F	6.32049900	-1.08851300	-0.28539200

II_H^{Cp}

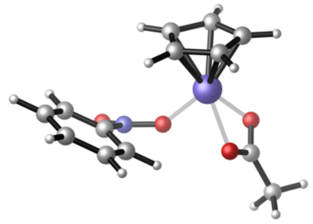

G = -967.689808

E = -967.885749345

O	1.41122800	1.19570800	-1.27993500
C	2.31191300	1.66959700	-0.51247300
O	2.61075900	0.98357100	0.51410900
C	-2.23607900	0.68609300	0.39028700
C	-1.86446900	0.95984000	-0.92362900
C	-3.43531100	0.06820000	0.73127000
C	-2.73858800	0.58453400	-1.93518600
C	-4.28359300	-0.31782500	-0.29807700
C	-3.93668100	-0.05949600	-1.62430300
H	-0.92965900	1.46374500	-1.14351700
H	-2.48519900	0.79791800	-2.96758000
H	-5.21703500	-0.81760800	-0.06469300
H	-4.60946000	-0.35703400	-2.42175500
Rh	1.05314100	-0.41933400	0.07518600
C	0.06696400	-2.19337300	0.83586600
H	-0.69093100	-2.13832100	1.60702800
C	1.46426000	-2.28885400	1.05907000
H	1.95469500	-2.34487200	2.02087200
C	2.11497000	-2.22614800	-0.22207100
H	3.18200800	-2.24291700	-0.39859800
C	-0.16812700	-2.08199000	-0.57184500
H	-1.12934400	-1.96181200	-1.05192600
C	1.10060500	-2.11687300	-1.22323600
H	1.27271900	-1.99955600	-2.28514400
C	2.96088300	2.98341100	-0.78266200
H	4.03522400	2.90783800	-0.60223400
H	2.76793800	3.30593700	-1.80588800
H	2.55317900	3.72290900	-0.08624000
O	-0.11178000	1.10826200	1.25190400
O	-1.80426600	1.36003100	2.54435800
N	-1.34301400	1.06693000	1.46640400
H	-3.68241300	-0.11799800	1.76992200

II_H^{Cp*}

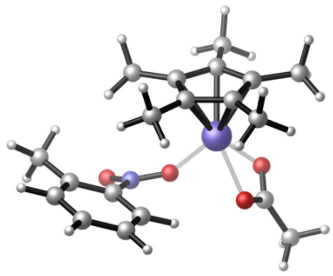

G = -1203.425842

E = -1203.78216141

O	-1.05965400	2.12162400	0.65607100
C	-1.74950900	2.50878900	-0.34139800
O	-2.12369700	1.63738500	-1.18359900
C	2.56709500	0.59292300	-0.34245900
C	2.08742700	1.40801800	0.68372100

C	3.70545300	-0.22125100	-0.21851400
C	2.76085700	1.43068700	1.89362600
C	4.33645200	-0.18663600	1.02966300
C	3.88484800	0.62504400	2.06472400
H	1.20595700	2.01911100	0.52644400
H	2.40915500	2.07113500	2.69465400
H	5.20949000	-0.81283500	1.18315300
H	4.41473800	0.62699700	3.01163600
Rh	-1.01518700	0.08176500	-0.12670200
C	-0.46949200	-2.01137300	-0.17362300
C	-1.81362500	-1.83974100	-0.63089500
C	-2.56894700	-1.23031600	0.44297300
C	-0.37352800	-1.51075800	1.17192700
C	-1.67883400	-1.04157500	1.55724500
C	-2.12120900	3.94761600	-0.51106100
H	-1.53618500	4.58053800	0.15653700
H	-1.96847600	4.24872200	-1.54967700
H	-3.18457100	4.06739900	-0.28125500
O	0.60508600	0.86655200	-1.56491500
O	2.41545700	0.47196200	-2.64016400
C	4.24952800	-1.11640800	-1.29821500
H	3.45977200	-1.65292800	-1.82992900
H	4.93203300	-1.84723400	-0.85950900
H	4.80065900	-0.53517100	-2.04245700
N	1.82874300	0.62975900	-1.59169500
C	0.65714800	-2.57849900	-0.96415500
C	-2.35931100	-2.23360600	-1.95986800
C	0.82936300	-1.55953900	2.04911800
C	-2.03847400	-0.44716900	2.87212700
C	-4.01997400	-0.90333500	0.40562400
H	1.75260100	-1.49973000	1.47085000
H	0.82501900	-0.74690700	2.77706200
H	0.83211800	-2.50810300	2.59712000
H	1.61816000	-2.25855700	-0.55885700
H	0.61409200	-3.67166800	-0.91336300
H	0.59708100	-2.28744200	-2.01520400
H	-1.57630900	-2.26428600	-2.71947800
H	-2.80617100	-3.23075100	-1.88458400
H	-3.13455900	-1.53878000	-2.28937500
H	-4.33543900	-0.61786600	-0.59930100
H	-4.58969700	-1.79093400	0.70256900
H	-4.26442300	-0.09383000	1.09517500
H	-1.17808400	0.03808900	3.33559000
H	-2.84170800	0.28502700	2.77363000
H	-2.38217300	-1.24517000	3.53951800

II_{Me}^{Cp*}

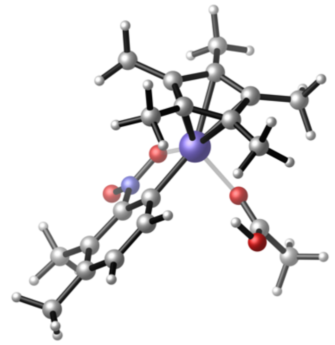

G = -1242.707272

E = -1243.09173867

O	1.28604800	2.01996100	-0.89626200
C	2.07652800	2.45652200	0.00025900
O	2.49809000	1.63987400	0.87442200
C	-2.25566100	0.81480700	0.42260000
C	-1.81896900	1.51342900	-0.70171900
C	-3.40768600	0.01593700	0.45619600
C	-2.58340600	1.44114700	-1.85259700
C	-4.15935100	-0.04698100	-0.73501000
C	-3.74131800	0.66855400	-1.85897400
H	-0.91636000	2.11225900	-0.65966400
H	-2.28272400	1.98972000	-2.73828400
H	-4.34107500	0.61819100	-2.76297200
Rh	1.21693900	0.06060200	0.09446700
C	0.49319900	-1.97219100	0.32641400
C	1.84737300	-1.85836700	0.77940800
C	2.65410100	-1.43801800	-0.34438900
C	0.43714200	-1.58129600	-1.05295800
C	1.78137600	-1.26155900	-1.47198700
C	2.45335000	3.90279500	0.04979300
H	2.53282700	4.31136600	-0.95896600
H	1.66207000	4.44572500	0.57756100
H	3.39059700	4.03792400	0.59082200
O	-0.22526500	1.09511300	1.53408400
O	-2.02372600	1.12850300	2.69543300
C	-3.84505400	-0.77757800	1.65807800
H	-3.03873400	-0.95979200	2.36685300
H	-4.24425000	-1.74488100	1.34488800
H	-4.64174500	-0.25167400	2.19499200
N	-1.46492800	1.00460700	1.62779200
C	-0.66122500	-2.38571800	1.17100200
C	2.35311900	-2.17130000	2.14562300
C	-0.76998100	-1.58661800	-1.92613200
C	2.19193600	-0.82349300	-2.83372600

C	4.12901500	-1.24356100	-0.33621900
H	-1.68596600	-1.45684200	-1.34735400
H	-0.72299200	-0.79296300	-2.67371700
H	-0.83041100	-2.54589500	-2.45142600
H	-1.61018700	-2.15723800	0.68592400
H	-0.60980200	-3.46713300	1.33563400
H	-0.63199200	-1.90075300	2.15023000
H	1.55528200	-2.10975000	2.88767900
H	2.75870600	-3.18861600	2.15973700
H	3.15061600	-1.48441900	2.43709800
H	4.48640500	-0.93605800	0.64781200
H	4.60967700	-2.19468100	-0.59096700
H	4.43761900	-0.49935900	-1.07209000
H	1.36753400	-0.34102200	-3.36080300
H	3.03259600	-0.12870800	-2.79069700
H	2.50121400	-1.70182000	-3.41106800
C	-5.41927300	-0.86996600	-0.79918200
H	-6.11632100	-0.59497500	-0.00124900
H	-5.92458800	-0.72547700	-1.75649000
H	-5.20822900	-1.93932000	-0.68933000

II_{NO2}^{Cp*}

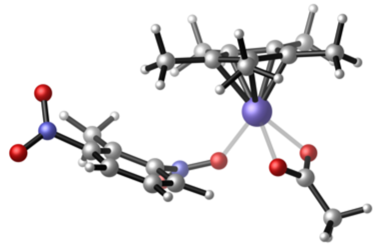

G = -1407.845529

E = -1408.20271049

O	1.67533400	1.88955500	-1.05855500
C	2.55185300	2.32197000	-0.24246500
O	2.93026200	1.54663200	0.68837100
C	-1.86023600	1.10473100	0.59295000
C	-1.44024400	1.75185100	-0.56428100
C	-3.03845100	0.35147300	0.70180000
C	-2.24218600	1.69667800	-1.69400400
C	-3.77405600	0.30108200	-0.48620000
C	-3.42574300	0.97337800	-1.65161100
H	-0.50227700	2.29552100	-0.57540700
H	-1.94723000	2.21371400	-2.59907100
H	-4.06990100	0.90596800	-2.52103900
Rh	1.52003800	0.02248000	0.06119800
C	1.11791100	-1.96707800	0.77773900
C	2.51701300	-1.81229800	0.54291200
C	2.69828400	-1.45285600	-0.85072900
C	0.42471200	-1.77042600	-0.47714100
C	1.39624400	-1.46718600	-1.47652800
C	3.12335200	3.69716400	-0.37141300
H	2.64450600	4.24263500	-1.18450300
H	2.99090800	4.23455300	0.57125700
H	4.19770500	3.61970900	-0.56188100
O	0.23913600	1.21853900	1.58457100
O	-1.48255500	1.42641000	2.84610800
C	-3.46519100	-0.33026700	1.97242700
H	-2.61990600	-0.82209600	2.45959300
H	-4.22722800	-1.08199200	1.78508900
H	-3.86458100	0.40760200	2.67195000
N	-0.99066400	1.24660500	1.75540300
C	0.46681700	-2.27512800	2.08122000
C	3.60837000	-1.93758800	1.54659500
C	-1.03271300	-1.95511200	-0.72114300
C	1.09781500	-1.17658100	-2.90473000
C	4.00202300	-1.20446500	-1.52704100
H	-1.61015500	-1.90079900	0.20157700
H	-1.41442700	-1.21635300	-1.42898500
H	-1.19038000	-2.94753500	-1.15775800
H	-0.52557300	-1.82248400	2.14328900
H	0.34884900	-3.35875200	2.18530900
H	1.06273000	-1.91119100	2.91977200
H	3.22755200	-1.84729100	2.56479700
H	4.07899800	-2.92085000	1.43878600
H	4.37750200	-1.17818700	1.39126200
H	4.74630600	-0.83249900	-0.82087800
H	4.37280700	-2.14165100	-1.95566500
H	3.89526900	-0.47712700	-2.33437100
H	0.15973400	-0.62529400	-3.00396500
H	1.89765300	-0.60312900	-3.37483500
H	0.99084100	-2.12506200	-3.44248000
N	-4.97615400	-0.53724900	-0.58334500
O	-4.86873700	-1.71888000	-0.29084300
O	-5.99433500	-0.00982500	-0.99718800

II_{OMe}^{Cp*}

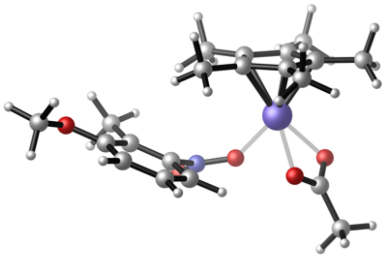

G = -1317.885722

E = -1318.27268236

O	1.56840600	1.78816700	-1.26242300
C	2.41267500	2.32537800	-0.47615200
O	2.81683000	1.65002900	0.51871200
C	-2.01218300	1.04287500	0.44180900
C	-1.62970900	1.46719300	-0.83150000
C	-3.18470800	0.33978900	0.72417000
C	-2.48148700	1.18865700	-1.88495500
C	-4.01520300	0.06840000	-0.38626200
C	-3.66827100	0.49090300	-1.67064400
H	-0.70261200	2.00917700	-0.97762800
H	-2.22672200	1.51776400	-2.88632100
H	-4.31849700	0.28070600	-2.51131300
Rh	1.42819300	0.03896200	0.04782900
C	0.72069100	-1.88370300	0.74174500
C	2.12602400	-1.75689000	0.97358600
C	2.76838600	-1.55070400	-0.30868200
C	0.47510500	-1.75239300	-0.67037300
C	1.74618900	-1.56577700	-1.31998600
C	2.87618900	3.73098900	-0.69027600
H	2.78874100	4.00997300	-1.74100400
H	2.23921900	4.39563400	-0.09698800
H	3.90485000	3.84969200	-0.34615900
O	0.10760900	1.38141700	1.35133600
O	-1.59602000	1.78767000	2.58301900
C	-3.59443000	-0.17080400	2.07776200
H	-2.74957800	-0.28759600	2.75620600
H	-4.09272200	-1.13517300	1.96672300
H	-4.30595000	0.51568900	2.54756700
N	-1.12327700	1.41073200	1.53353800
C	-0.32481600	-2.07306100	1.78577400
C	2.82168100	-1.84167500	2.28761600
C	-0.84161300	-1.89929100	-1.35037200
C	1.95850300	-1.39055000	-2.78205800
C	4.23111500	-1.38894600	-0.53660300
H	-1.66179200	-1.57650700	-0.70785100
H	-0.88078200	-1.32395200	-2.27633000
H	-0.99915200	-2.95518900	-1.59639300
H	-1.28988900	-1.69397300	1.44588200
H	-0.43603800	-3.14276400	1.99276400
H	-0.05638300	-1.57316300	2.71863700
H	2.13420400	-1.64882600	3.11228400
H	3.23841400	-2.84694800	2.41214500
H	3.64400700	-1.12538300	2.34529700
H	4.73127200	-1.02118200	0.36047600
H	4.66348300	-2.36066600	-0.79813800
H	4.43213800	-0.69404700	-1.35421800
H	1.11487000	-0.87783900	-3.24723000
H	2.86961600	-0.82763100	-2.98923800
H	2.05462200	-2.37842100	-3.24625900
O	-5.14309800	-0.61193500	-0.09894100
C	-6.01339300	-0.96448500	-1.16412800
H	-6.83472700	-1.51559000	-0.70650400
H	-6.41177500	-0.07515000	-1.66421800
H	-5.50886400	-1.60526900	-1.89569000

III

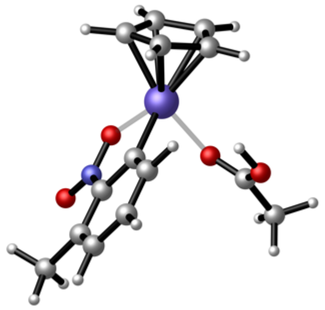

G = -1006.976568

E = -1007.20332418

O	-0.67313900	2.82372400	0.83470300
C	-0.49916100	2.65926400	-0.45827600
O	-0.60392700	1.56566300	-1.02064400
C	1.85163400	-0.50769200	-0.08578900
C	0.75609100	-0.23907900	0.75077000
C	3.20281300	-0.40760100	0.31122500
C	1.02373900	0.13893300	2.06135900
C	3.40716100	-0.01731000	1.63416700
C	2.34433700	0.24926100	2.49316800
H	-0.87059800	1.96711700	1.25483200
H	0.21329800	0.34778700	2.75283500
H	4.42557800	0.07631700	1.99675800
H	2.55006200	0.54424200	3.51758500
Rh	-1.04485700	-0.37264700	-0.13305500
C	-2.03012700	-2.16751000	0.45772600
H	-1.54605500	-3.13449800	0.45193500
C	-2.89006800	-1.64207700	-0.59256500
H	-3.06201500	-2.11417500	-1.55046000
C	-3.34035000	-0.38947300	-0.19768600
H	-3.93646300	0.29380900	-0.78671000
C	-2.06700100	-1.25415900	1.55207000
H	-1.56206100	-1.37464500	2.50037700

C	-2.77694400	-0.10330600	1.11390000
H	-2.97851600	0.78269100	1.70193900
C	-0.16803800	3.90771800	-1.19735300
H	0.72779300	4.35863300	-0.76271600
H	-0.00742100	3.68763900	-2.25101500
H	-0.98911900	4.62115500	-1.08309400
O	0.25757700	-0.99901200	-1.67601600
O	2.30730600	-1.13771200	-2.27645500
C	4.38839600	-0.68451100	-0.56811500
H	4.37234300	-1.70716300	-0.95410800
H	5.30706700	-0.53954500	0.00347600
H	4.40752000	-0.01819500	-1.43473700
N	1.49281700	-0.89972800	-1.41567500

III_{Br}^{Cp*}

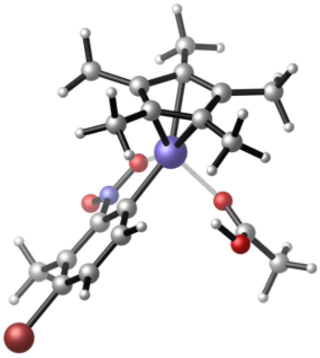

G = -1216.003411

E = -1216.34961256

O	0.72257300	3.14302400	-0.32069000
C	1.01901700	2.86702400	0.93367700
O	1.29047800	1.73019900	1.32195300
C	-1.42440400	-0.49857900	0.79485700
C	-0.58571900	0.00566300	-0.20934200
C	-2.84108800	-0.52970200	0.74915700
C	-1.21105600	0.55757800	-1.32410200
C	-3.38488700	0.03570200	-0.40956800
C	-2.59399500	0.57825700	-1.42264700
H	0.72708500	2.31995600	-0.84105600
H	-0.62843800	0.97824700	-2.13781100
H	-3.06390700	1.01332900	-2.29800000
Rh	1.40751200	-0.12492600	0.13399200
C	2.26515400	-1.87561200	-0.69806800
C	3.34846300	-1.29401000	0.10199300
C	3.60877200	-0.01454100	-0.39694600
C	1.96967500	-0.97808300	-1.77537500
C	2.70458600	0.22580800	-1.52648500
C	0.97320200	4.04314900	1.84607500
H	1.46280600	4.90113700	1.38148000
H	-0.07618800	4.30460400	2.01618900
H	1.44386800	3.79729800	2.79635200
O	0.54161000	-0.94790000	1.92562500
O	-1.28029700	-1.43585100	2.92927700
C	-3.70084600	-1.11959800	1.83075200
H	-3.42729800	-2.15905700	2.02402400
H	-4.75070400	-1.08648100	1.54630300
H	-3.57923600	-0.57480100	2.76974800
N	-0.71602400	-0.99707200	1.95310900
C	1.72141200	-3.25213000	-0.51299500
C	3.96977000	-1.97455300	1.27494200
C	1.08674500	-1.26454000	-2.94117000
C	2.76065100	1.42677500	-2.41298500
C	4.57284900	0.99858600	0.12301800
H	0.25380600	-1.91441000	-2.66631500
H	0.68608200	-0.35113300	-3.38295800
H	1.67803700	-1.77583900	-3.70870500
H	0.75900300	-3.36909200	-1.01457200
H	2.42073600	-3.98367400	-0.93263800
H	1.59121100	-3.48122600	0.54735900
H	3.20558900	-2.41329300	1.92230900
H	4.61806800	-2.78733200	0.93035600
H	4.57032700	-1.28607300	1.87100800
H	4.98055200	0.70885500	1.09249000
H	5.40292500	1.11395600	-0.58198600
H	4.09383700	1.97603000	0.22845700
H	1.81164500	1.59340900	-2.92862100
H	3.01849300	2.32672200	-1.84993000
H	3.53257100	1.28384100	-3.17728900
Br	-5.28004200	0.11220800	-0.69444700

III_{CF3}^{Cp*}

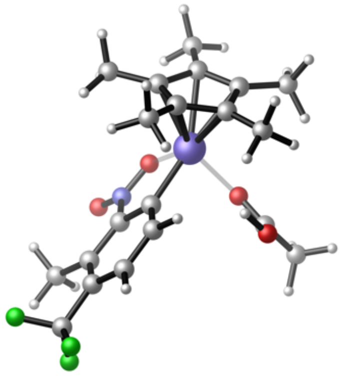

G = -1540.362689

E = -1540.72203024

O	0.69720300	3.14989400	-0.28369600
C	0.95648900	2.85302900	0.97444200
O	1.21957300	1.71043500	1.35107200
C	-1.47030500	-0.52199300	0.76031100
C	-0.62036300	0.00707400	-0.22449300
C	-2.88214000	-0.54622600	0.69178600
C	-1.23033100	0.58540700	-1.33204600
C	-3.42640700	0.05121300	-0.45575000
C	-2.61234100	0.61237000	-1.44056600
H	0.72443000	2.33709600	-0.81911600
H	-0.63480300	1.02301400	-2.12719100
H	-3.06847200	1.07237200	-2.30927700
Rh	1.36664000	-0.12652200	0.13798600
C	2.23709100	-1.86429600	-0.70768200
C	3.31165200	-1.28958300	0.10915100
C	3.57310600	-0.00375400	-0.37243400
C	1.94849700	-0.95424100	-1.77644100
C	2.67922100	0.24779400	-1.50780900
C	0.90408000	4.01916200	1.89930300
H	1.63832700	4.76415100	1.58128300
H	-0.08509700	4.48101600	1.84127700
H	1.11172200	3.69836300	2.91828800
O	0.48449300	-0.98534900	1.90405000
O	-1.34620700	-1.51270700	2.87114200
C	-3.75519300	-1.16205000	1.75266600
H	-3.47102900	-2.20168000	1.92763800
H	-4.80563000	-1.14058700	1.47982300
H	-3.63688900	-0.63564600	2.70250600
N	-0.77311900	-1.04502100	1.91374500
C	1.69402400	-3.24384000	-0.54437900
C	3.92494700	-1.98218500	1.27941600
C	1.07642000	-1.22756100	-2.95339100
C	2.74146800	1.45749100	-2.38210300
C	4.52893000	1.00604300	0.16911000
H	0.24728100	-1.88921700	-2.69591100
H	0.67041800	-0.31013200	-3.38179400
H	1.67735300	-1.72016000	-3.72562000
H	0.73740000	-3.35648300	-1.05787100
H	2.39922200	-3.96924800	-0.96483000
H	1.55233700	-3.48576600	0.51162400
H	3.15765400	-2.44472100	1.90605700
H	4.59116600	-2.77834900	0.93015100
H	4.50581300	-1.29590300	1.89725300
H	4.92852300	0.70535900	1.13857700
H	5.36525000	1.13329500	-0.52635200
H	4.04553100	1.98048100	0.28244200
H	1.79439800	1.63186200	-2.89908500
H	3.00079500	2.35130100	-1.80995600
H	3.51505800	1.31969900	-3.14564300
C	-4.92185500	0.11922100	-0.65952300
F	-5.48348300	-1.10320300	-0.73256300
F	-5.54086600	0.77491500	0.34071300
F	-5.24567200	0.75738500	-1.79457500
C	1.01901700	2.86702400	0.93367700

III_{Cl}^{Cp*}

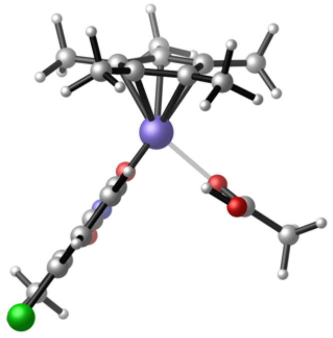

G = -1663.004576

E = -1663.34987970

O	0.21014000	3.04680600	-0.76438200
C	0.59566500	2.95842100	0.49328800
O	0.83348000	1.88472400	1.04705100
C	-1.89810400	-0.34194900	0.68423400
C	-0.98644800	-0.07748300	-0.34776700
C	-3.30824100	-0.36436300	0.55463400
C	-1.52878700	0.21812000	-1.59674400
C	-3.76403900	-0.05767700	-0.73148800
C	-2.90242400	0.23544900	-1.78690700
H	0.12624700	2.15075600	-1.13715700
H	-0.88462500	0.44404400	-2.44070100
H	-3.31602900	0.47064900	-2.76178300
Rh	0.98068700	-0.14174600	0.15668700
C	1.90201800	-1.96944700	-0.39841300
C	2.90032700	-1.28926200	0.43082900
C	3.20983600	-0.07048700	-0.18230100
C	1.71067500	-1.19810400	-1.59216600
C	2.41574900	0.03490700	-1.41117800
C	0.71665400	4.27091600	1.18707000
H	1.35034200	4.93707600	0.59633000
H	-0.27323000	4.73020600	1.26144800
H	1.13625900	4.13032300	2.18154500
O	-0.01939600	-0.64829100	1.99638900
O	-1.91275000	-0.73669500	2.98446200

C	-4.25744800	-0.69458200	1.67186600
H	-3.99070100	-1.64129500	2.14551600
H	-5.27656600	-0.76996600	1.29745100
H	-4.22816900	0.07459600	2.44759900
N	-1.27670900	-0.59327800	1.96521200
C	1.35312900	-3.33088200	-0.13378500
C	3.41639400	-1.82049200	1.72557800
C	0.95615300	-1.63733900	-2.80087700
C	2.52204900	1.14783500	-2.40217600
C	4.11848300	0.99888600	0.32372100
H	0.03746000	-2.16632100	-2.53928600
H	0.70568200	-0.80074600	-3.45482200
H	1.59076500	-2.32426400	-3.37147700
H	0.40442600	-3.48249000	-0.65200800
H	2.06305400	-4.08703200	-0.48681000
H	1.19359500	-3.49078100	0.93482800
H	2.61574800	-2.28448100	2.30703300
H	4.17274500	-2.58898600	1.53299600
H	3.87141400	-1.03387900	2.32945900
H	4.35546500	0.85422100	1.37899600
H	5.05421700	0.99297900	-0.24539100
H	3.66501500	1.98647100	0.20325000
H	1.59908900	1.25722700	-2.97791000
H	2.73643200	2.09870400	-1.90916500
H	3.33675500	0.94105700	-3.10434100
Cl	-5.48005600	-0.01845400	-1.09268700

III_F^{Cp*}

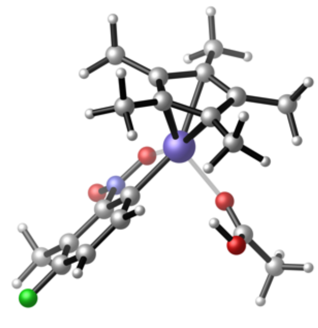

G = -1302.630896

E = -1302.98124593

O	0.10925900	3.12256900	-0.59838400
C	0.26718500	2.92076200	0.69451700
O	0.47955100	1.80856000	1.17735800
C	-2.12052100	-0.42497800	0.45139400
C	-1.14262500	-0.04531900	-0.47882600
C	-3.51381200	-0.41598300	0.22044500
C	-1.59401800	0.38148200	-1.72728400
C	-3.86759700	0.02583400	-1.05018300
C	-2.95256400	0.42282200	-2.01442400
H	0.16489300	2.26991100	-1.06581000
H	-0.88695500	0.68627300	-2.49394400
H	-3.31833600	0.75700500	-2.97966600
Rh	0.77877500	-0.13166400	0.16663800
C	1.81257200	-1.92145600	-0.32687200
C	2.75097900	-1.17688500	0.51549700
C	3.01187200	0.04450800	-0.11500700
C	1.59891200	-1.18009300	-1.53367000
C	2.24410900	0.08871100	-1.36409000
C	0.16531300	4.15660900	1.51917800
H	0.89714200	4.88777000	1.16571700
H	-0.82913400	4.59335100	1.39101600
H	0.34103500	3.92428700	2.56787100
O	-0.35114500	-0.82214000	1.88252100
O	-2.31876000	-1.19034700	2.64025800
C	-4.58548800	-0.82769000	1.18910700
H	-4.45645200	-1.86767000	1.49780600
H	-5.56319000	-0.71830500	0.72170500
H	-4.55696800	-0.21595500	2.09367300
N	-1.60004700	-0.83913400	1.73153100
C	1.32036200	-3.29990100	-0.04317900
C	3.26099900	-1.66640200	1.83000600
C	0.87750900	-1.66849800	-2.74366400
C	2.33974600	1.16917000	-2.39178400
C	3.84797700	1.17246000	0.38869700
H	-0.00717200	-2.25134700	-2.47938800
H	0.57363500	-0.84795900	-3.39509200
H	1.55162800	-2.31555800	-3.31549600
H	0.36349300	-3.48748700	-0.53458900
H	2.04718200	-4.02973600	-0.41710200
H	1.19970300	-3.46381000	1.02954200
H	2.44789200	-2.06762700	2.44116000
H	3.98557100	-2.47210500	1.67117800
H	3.75155000	-0.86989800	2.39173000
H	4.07663100	1.05903900	1.44925000
H	4.79059100	1.21201600	-0.16777700
H	3.34094400	2.13071800	0.24609200
H	1.41282600	1.25460600	-2.96552400
H	2.55668300	2.13744600	-1.93552400
H	3.14875900	0.94140100	-3.09400300
F	-5.17257800	0.07470900	-1.37504800

III_H^{Cp}

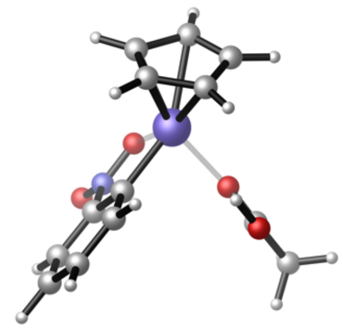

G = -967.695349

E = -967.894375328

O	-0.23751000	2.80144700	1.06981700
C	-0.08251000	2.71113000	-0.23194700
O	-0.32523200	1.68234700	-0.86902200
C	1.95716300	-0.70937800	-0.30762200
C	0.96542800	-0.41586700	0.63039900
C	3.31585700	-0.82305400	-0.01372800
C	1.38732400	-0.23253800	1.94736700
C	3.70134600	-0.63462700	1.30266300
C	2.73845500	-0.34213300	2.27424100
H	-0.55422500	1.95097600	1.42548700
H	0.66682300	-0.00658800	2.72709800
H	4.74682100	-0.71880000	1.57671100
H	3.04766900	-0.19953600	3.30519000
Rh	-0.88824700	-0.28557000	-0.12917500
C	-2.02936000	-2.06546300	0.15340200
H	-1.66620200	-3.03344600	-0.16549200
C	-2.93860900	-1.21292900	-0.59714000
H	-3.30014000	-1.40679200	-1.59750900
C	-3.15825500	-0.06545100	0.15422700
H	-3.72127500	0.80523900	-0.15491200
C	-1.79799500	-1.46588400	1.42363400
H	-1.19458100	-1.87500000	2.22190600
C	-2.40201200	-0.17608900	1.39374700
H	-2.42085800	0.53734400	2.20712700
C	0.40519900	3.95911800	-0.87994600
H	-0.32870500	4.75455200	-0.72269300
H	1.34009800	4.26914300	-0.40570900
H	0.55715300	3.79336300	-1.94468200
O	0.26623400	-0.82689500	-1.86271200
O	2.27636500	-1.13256800	-2.55957000
N	1.50788700	-0.90323200	-1.65566000
H	4.03227600	-1.05281900	-0.79389700

III_H^{Cp*}

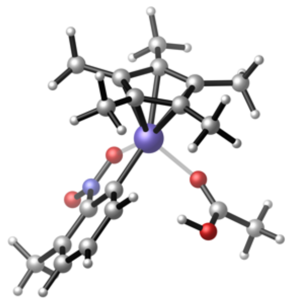

G = -1203.419601

E = -1203.77840921

O	0.66602200	3.01577400	0.70462700
C	0.08542800	2.96621700	-0.47550900
O	-0.30922800	1.91920200	-0.98974800
C	2.31667900	-0.43910300	-0.24888300
C	1.26659000	-0.12902800	0.63224300
C	3.68060700	-0.47807500	0.11113900
C	1.61259400	0.17490700	1.94811100
C	3.95720800	-0.16685300	1.44376100
C	2.94954300	0.16024400	2.34477500
H	0.77472100	2.11139800	1.05409100
H	0.84301400	0.42575400	2.67290600
H	4.98994900	-0.18272500	1.77615600
H	3.20886500	0.39954600	3.37169800
Rh	-0.59525300	-0.12573700	-0.17326200
C	-1.70730900	-1.90949900	0.15086200
C	-2.56738200	-1.10536300	-0.72075000
C	-2.84071500	0.09379500	-0.05625400
C	-1.57530800	-1.23296500	1.40750500
C	-2.15987300	0.06503000	1.24380500
C	-0.03081400	4.28679300	-1.15727800
H	-0.26178200	5.07094600	-0.43449800
H	0.93503600	4.52103100	-1.61684100
H	-0.79231300	4.23847700	-1.93441900
O	0.66403200	-0.68578100	-1.84538300
O	2.68760000	-0.96932000	-2.48339700
C	4.81925800	-0.82262400	-0.80691300
H	4.69308500	-1.81463600	-1.24819900
H	5.75641700	-0.80301600	-0.24691200
H	4.89239000	-0.11402000	-1.63603900
N	1.89859400	-0.71706600	-1.59936900
C	-1.23018000	-3.28834800	-0.15639100
C	-3.00058100	-1.52531400	-2.08572200

C	-0.96760000	-1.80646400	2.64274300
C	-2.26682300	1.13348500	2.28174200
C	-3.61726700	1.26246700	-0.55923600
H	-0.05744600	-2.36799000	2.42237800
H	-0.73389600	-1.03497500	3.37703400
H	-1.68764500	-2.49515200	3.09787000
H	-0.33293600	-3.53313900	0.41582100
H	-2.01069100	-4.01173700	0.10467500
H	-1.00790900	-3.40157500	-1.21954800
H	-2.16509200	-1.94816000	-2.64972500
H	-3.77222400	-2.29901100	-2.00912000
H	-3.41026400	-0.68785300	-2.65259200
H	-3.81253800	1.18432800	-1.62969300
H	-4.57689900	1.31955000	-0.03405400
H	-3.08275000	2.19766200	-0.37300900
H	-1.46491900	1.05066700	3.01942400
H	-2.21588600	2.12683800	1.82935300
H	-3.22379400	1.05060300	2.80829500

III_{Me}^{Cp*}

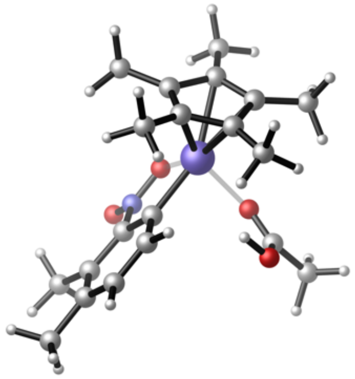

G = -1242.699485

E = -1243.08628152

O	0.05950000	3.06964300	-0.71960800
C	0.24571400	2.93317000	0.57772800
O	0.48681000	1.84870600	1.10806500
C	-2.10500800	-0.40555000	0.49574100
C	-1.13076900	-0.07919000	-0.46125000
C	-3.49926800	-0.43405400	0.25958400
C	-1.59586000	0.27274500	-1.72306000
C	-3.91677000	-0.06514400	-1.03160300
C	-2.96062700	0.28704600	-1.98925000
H	0.11194800	2.19425300	-1.14330800
H	-0.89923700	0.54106000	-2.51311700
H	-3.30068100	0.57397500	-2.98097400
Rh	0.79400700	-0.12796400	0.16752300
C	1.83554000	-1.92443800	-0.28469000
C	2.77468600	-1.15516300	0.53486600
C	3.02758700	0.05048200	-0.12724000
C	1.61817700	-1.21653500	-1.51093000
C	2.25536100	0.05892400	-1.37522900
C	0.13904100	4.20498300	1.34592500
H	0.86665600	4.92389300	0.95943100
H	-0.85780500	4.63077400	1.20201300
H	0.31919400	4.01951300	2.40324600
O	-0.31028700	-0.73114800	1.91724800
O	-2.26339900	-0.92268600	2.76448400
C	-4.51511300	-0.83791400	1.29404400
H	-4.25808300	-1.79430500	1.75409200
H	-5.50441200	-0.92845900	0.84854300
H	-4.56886800	-0.10265600	2.10196000
N	-1.56554100	-0.70775500	1.79775600
C	1.35442700	-3.30004300	0.03173200
C	3.29006900	-1.60896600	1.86027400
C	0.90058800	-1.74291000	-2.70717000
C	2.34299100	1.11342400	-2.43046800
C	3.85995700	1.19537300	0.34407600
H	0.00864100	-2.30736100	-2.42798400
H	0.60892400	-0.94345900	-3.38951600
H	1.57282900	-2.41635100	-3.24998100
H	0.40148300	-3.50800600	-0.45931100
H	2.08876800	-4.03287600	-0.32099300
H	1.22951700	-3.43945700	1.10759100
H	2.47942300	-1.99082400	2.48692800
H	4.01280300	-2.42011100	1.72174400
H	3.78447300	-0.79773100	2.39711700
H	4.09977800	1.10594900	1.40457900
H	4.79717000	1.23118100	-0.22173500
H	3.34424500	2.14684700	0.18776400
H	1.41605700	1.17687100	-3.00739700
H	2.54938100	2.09459000	-1.99713000
H	3.15539700	0.87632300	-3.12569100
C	-5.37616900	-0.03763100	-1.41404800
H	-5.95534700	0.62163100	-0.75986500
H	-5.49410300	0.32314800	-2.43832300
H	-5.82802200	-1.03349300	-1.35821700

III_{NO2}^{Cp*}

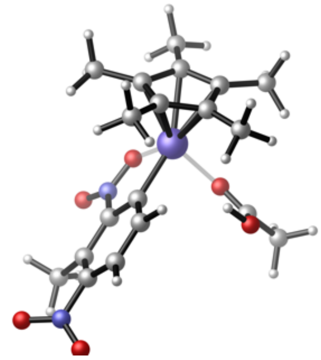

G = -1407.843847

E = -1408.20143269

O	0.39844200	3.14746100	-0.30851200
C	0.67464400	2.85647400	0.94699700
O	0.93054900	1.71355200	1.32632400
C	-1.72105900	-0.50123000	0.70182500
C	-0.84276000	-0.04912200	-0.29411500
C	-3.13211200	-0.45802700	0.63204900
C	-1.41054800	0.46175800	-1.46129600
C	-3.60817300	0.04138600	-0.58388100
C	-2.78579800	0.51051200	-1.60607100
H	0.40502200	2.33087300	-0.83825400
H	-0.77947700	0.81924100	-2.26963800
H	-3.23698000	0.90315900	-2.51032500
Rh	1.13282900	-0.14067500	0.13755900
C	2.11105500	-1.86851300	-0.61889200
C	3.13356600	-1.20169300	0.19184500
C	3.33205200	0.07668900	-0.33947900
C	1.78889900	-1.02065700	-1.72776100
C	2.44545100	0.23266700	-1.49815500
C	0.65074200	4.02976700	1.86376200
H	-0.33321500	4.50387300	1.81837900
H	0.87150800	3.71438200	2.88164400
H	1.38884200	4.76325500	1.52779300
O	0.19680300	-0.97491000	1.90252400
O	-1.66177800	-1.57758200	2.77091200
C	-4.04888500	-0.81799700	1.76922000
H	-4.11853000	-1.90247100	1.88692100
H	-5.05089700	-0.42953600	1.60257800
H	-3.66708300	-0.41071600	2.70521000
N	-1.05721800	-1.04741100	1.86734600
C	1.63974600	-3.26825400	-0.41387900
C	3.77749200	-1.81195500	1.39120600
C	0.95165700	-1.39472100	-2.90392100
C	2.42870900	1.41414900	-2.41270900
C	4.21891200	1.15358300	0.18783000
H	0.09077000	-1.99943400	-2.61200700
H	0.59692200	-0.51696400	-3.44592400
H	1.56402800	-1.98765900	-3.59177100
H	0.69101700	-3.44406700	-0.92443900
H	2.38193700	-3.96756000	-0.81462100
H	1.50989900	-3.48793900	0.64832300
H	3.04024400	-2.31689100	2.02066100
H	4.50942300	-2.56169400	1.07210900
H	4.29393200	-1.06490800	1.99572600
H	4.48080200	0.98222700	1.23295500
H	5.14316700	1.19329500	-0.39867000
H	3.73805000	2.13183500	0.11020900
H	1.44721300	1.54822900	-2.87600700
H	2.69168100	2.33256900	-1.88337200
H	3.15996400	1.27360600	-3.21600500
N	-5.04578000	0.09591200	-0.86178600
O	-5.73157600	-0.86594200	-0.54900300
O	-5.46945400	1.09182900	-1.42949600

III_{OMe}^{Cp*}

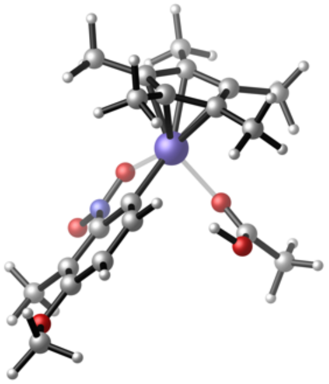

G = -1317.879707

E = -1318.26634164

O	-0.15155400	2.93706400	-0.64262200
C	0.44869100	2.91686600	0.52935800
O	0.83279900	1.87658300	1.06583300
C	-1.84215000	-0.40470000	0.81285300
C	-0.98033700	-0.14989600	-0.26744100
C	-3.24683900	-0.43249400	0.74840300
C	-1.59111400	0.10863600	-1.48809700
C	-3.79914700	-0.15796500	-0.52048300
C	-2.97909000	0.11315200	-1.61669400
H	-0.28436600	2.01996100	-0.94810600
H	-0.99107200	0.31828700	-2.36970600
H	-3.41582000	0.32521200	-2.58551800

Rh	1.00592900	-0.14735100	0.15654500
C	2.05584100	-1.90811600	-0.40938300
C	3.05198300	-1.10425700	0.30176200
C	3.18229000	0.11103000	-0.37972100
C	1.68079200	-1.21131700	-1.60450100
C	2.27165700	0.09093400	-1.53066200
C	0.60730600	4.25713700	1.16071400
H	0.91717800	4.99035700	0.41360200
H	-0.36236600	4.57158200	1.55940800
H	1.33231100	4.20386100	1.97134100
O	0.09604300	-0.69917400	2.04015100
O	-1.75393800	-0.80548900	3.10829900
C	-4.16993100	-0.73283400	1.89696200
H	-3.91676900	-1.68378200	2.37098800
H	-5.19674700	-0.77885200	1.53878100
H	-4.10240300	0.03787300	2.66956700
N	-1.16204200	-0.65158000	2.06346600
C	1.65759300	-3.29761400	-0.04214700
C	3.73311300	-1.53971800	1.55686900
C	0.87052400	-1.77438800	-2.72238000
C	2.15967600	1.17199300	-2.55517400
C	4.01629200	1.29067100	-0.00847100
H	0.01671500	-2.34870000	-2.35767600
H	0.51009200	-0.99454100	-3.39477700
H	1.50743100	-2.44794900	-3.30615100
H	0.69215400	-3.55836300	-0.48008800
H	2.40602800	-4.00580500	-0.41507800
H	1.59194300	-3.41741900	1.04166700
H	3.01754600	-1.97261900	2.26103800
H	4.47813400	-2.30870100	1.32704300
H	4.23820400	-0.70827900	2.05093000
H	4.40518200	1.20986000	1.00740300
H	4.86283400	1.37421700	-0.69843400
H	3.43884800	2.21641300	-0.08453300
H	1.18328400	1.15443600	-3.04548300
H	2.30160500	2.15694200	-2.10481600
H	2.92798800	1.04281000	-3.32545600
O	-5.14925800	-0.17685300	-0.59329200
C	-5.76685900	0.05398500	-1.84887500
H	-6.83901800	-0.03217700	-1.67250700
H	-5.54425600	1.05797900	-2.22772800
H	-5.46163500	-0.69444700	-2.58877900

IV

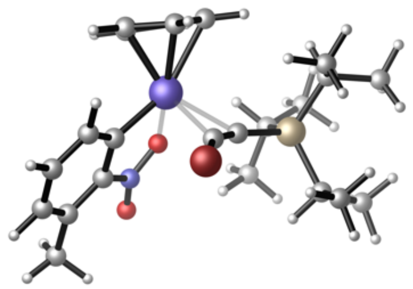

G = -1512.084846

E = -1512.52926001

C	2.73818800	1.25484600	-0.00025000
C	2.48853400	-0.11985200	-0.12705100
C	3.89017900	1.80120900	0.61064700
C	3.43840200	-1.00263400	0.37075400
C	4.80925800	0.86989200	1.08584600
C	4.59242200	-0.50163700	0.96611600
H	3.28539500	-2.07420300	0.30759800
H	5.71590800	1.22993100	1.56067700
H	5.33746400	-1.19266400	1.34845300
Rh	0.76043000	-0.60457400	-1.05363100
C	1.35588800	-1.17921200	-3.07399900
H	2.03300300	-0.59084000	-3.67807700
C	-0.09189100	-1.11633300	-3.11916900
H	-0.67054200	-0.42181200	-3.71300100
C	-0.59782100	-2.05096700	-2.21665300
H	-1.63932200	-2.22777900	-1.99370400
C	1.71944100	-2.22487600	-2.18339600
H	2.72700800	-2.53692500	-1.94902700
C	0.52494000	-2.69334500	-1.56198600
H	0.46047900	-3.47612700	-0.81812300
O	0.76909500	1.50289900	-1.15525800
O	1.74068500	3.29299500	-0.49730200
C	4.16909700	3.26866500	0.76735500
H	4.20908200	3.77428200	-0.20135900
H	5.12775500	3.40415100	1.27175100
H	3.39005100	3.76378800	1.35268800
N	1.72479400	2.08544300	-0.55884000
Si	-2.60038000	0.45940800	0.15590400
C	-3.04758100	1.29966500	1.79679300
C	-2.51497200	1.67947300	-1.28912600
C	-3.62503500	-1.08879200	-0.26302700
H	-2.23843200	2.02745700	1.95503800
H	-1.79425600	1.24071900	-1.99444800
H	-3.47882700	-1.24437000	-1.34206900
C	-3.04661000	0.32758300	2.98471500

C	-4.36868400	2.08468100	1.74217800
C	-3.17198000	-2.36128500	0.46891800
C	-5.12875600	-0.86154500	-0.03369600
C	-1.97808700	3.05488600	-0.86400900
C	-3.85495700	1.82783700	-2.02645100
H	-3.77163200	-3.22053400	0.14528500
H	-2.11994700	-2.60028900	0.28540700
H	-3.29960100	-2.26305400	1.55227600
H	-5.35185000	-0.77946800	1.03532800
H	-5.50242900	0.04200900	-0.52469300
H	-5.70334000	-1.71045600	-0.42260900
H	-4.51419100	2.63824800	2.67754300
H	-4.39281200	2.81079700	0.92428400
H	-5.22916900	1.41985600	1.62179200
H	-3.20808200	0.86836500	3.92483200
H	-3.84778700	-0.41421400	2.89316800
H	-2.09982500	-0.21509500	3.07993600
H	-4.63432700	2.23120700	-1.37005900
H	-3.74862300	2.52406300	-2.86708700
H	-4.21578800	0.87613400	-2.43024500
H	-1.02990700	2.98506300	-0.32507200
H	-1.80358600	3.68318800	-1.74544900
H	-2.69359800	3.58249400	-0.22463700
C	-0.85874900	-0.19448000	0.48360500
C	0.08510200	-0.76668000	1.06373300
Br	0.84508400	-1.54764800	2.52958900

V

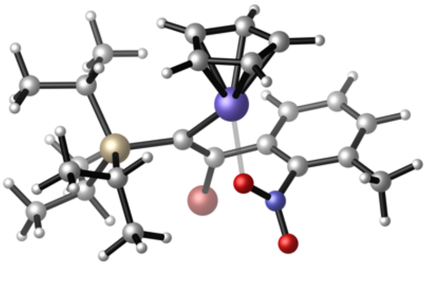

G = -1512.103608

E = -1512.54964304

C	2.54107300	-0.87130600	0.48342100
C	1.70013000	-1.10903200	-0.62448500
C	3.93632600	-0.76659800	0.41162600
C	2.32179100	-1.22371600	-1.87839100
C	4.48863600	-0.88560300	-0.86076900
C	3.69732300	-1.10861900	-1.98903500
H	1.70640200	-1.42100700	-2.74960600
H	5.56423500	-0.79252200	-0.97015900
H	4.16725100	-1.19820500	-2.96220000
Rh	0.97068200	1.22617800	-0.10805500
C	1.72685800	3.30155900	0.37631100
H	2.12786800	3.56563000	1.34581300
C	0.30722400	3.23987700	0.04475000
H	-0.50321900	3.54973200	0.69086500
C	0.19341300	2.84109000	-1.31844100
H	-0.71204300	2.76415700	-1.89808900
C	2.45058800	2.85579300	-0.72180000
H	3.52021600	2.70645100	-0.77305200
C	1.49843600	2.48590700	-1.75574000
H	1.75061300	2.08075300	-2.72699500
O	1.14254300	0.26129000	1.92297900
O	2.14553700	-1.48822600	2.68920400
C	4.78423400	-0.50261800	1.62376900
H	4.40716800	0.35067900	2.19660000
H	5.80935300	-0.28368400	1.32137500
H	4.80193800	-1.37098200	2.28815900
N	1.90810100	-0.71419400	1.79876900
Si	-2.35598100	0.14946400	0.14933900
C	-3.40996100	-1.43455100	0.16877700
C	-2.19160000	0.91104100	1.88870300
C	-2.97659500	1.42089800	-1.12194700
H	-2.81090900	-2.17217600	0.71898700
H	-1.29287400	1.54092200	1.81703800
H	-2.48821700	2.36258300	-0.83199100
C	-3.68290800	-1.99593400	-1.23521500
C	-4.73640200	-1.26959700	0.93041100
C	-2.56495200	1.07740900	-2.56364900
C	-4.49267200	1.66944100	-1.06705900
C	-1.90935600	-0.16264800	2.95118300
C	-3.34696900	1.82611500	2.31456900
H	-2.78795900	1.91060300	-3.24210800
H	-1.49955900	0.84095600	-2.66045000
H	-3.11983200	0.20815900	-2.92986800
H	-5.04873600	0.79292400	-1.41525800
H	-4.84881000	1.91078800	-0.06151900
H	-4.76558600	2.50550500	-1.72297600
H	-5.28103500	-2.22208200	0.94445700
H	-4.58793800	-0.96575800	1.97005000
H	-5.39132900	-0.53068100	0.45813300

H	-4.11536100	-3.00188800	-1.16989800
H	-4.40086200	-1.36861600	-1.77489200
H	-2.77886900	-2.06732500	-1.84743100
H	-4.29989300	1.28888200	2.36898800
H	-3.15668200	2.24901800	3.30919000
H	-3.47651800	2.66485700	1.62240300
H	-1.09562100	-0.83392000	2.65751100
H	-1.62203400	0.30031800	3.90301400
H	-2.79291400	-0.78006700	3.14423200
C	-0.53509200	-0.20632000	-0.27732800
C	0.22065200	-1.27813600	-0.47403500
Br	-0.32736000	-3.14075400	-0.55917700

V′

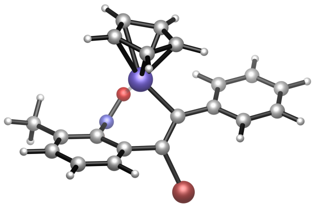

G = -1098.819075

E = -1099.08007759

C	-2.10233300	0.75399600	0.51132000
C	-1.24324100	1.16987400	-0.52537200
C	-3.48680000	0.59403600	0.37666600
C	-1.83342100	1.42785000	-1.76892300
C	-4.01068100	0.85418100	-0.88749500
C	-3.19964100	1.26757300	-1.94350500
H	-1.20480700	1.76320300	-2.58705000
H	-5.07711800	0.72836500	-1.04347900
H	-3.64395000	1.46534400	-2.91287200
Rh	-0.32827300	-1.32837600	-0.12578300
C	-0.74372700	-3.58300100	0.11215800
H	-1.26793500	-4.02120500	0.95041600
C	0.65979400	-3.17174200	0.12142700
H	1.36192100	-3.34297600	0.92664700
C	0.98175100	-2.65883700	-1.17415900
H	1.95044900	-2.31370900	-1.50747300
C	-1.29179800	-3.19689200	-1.09792000
H	-2.33068400	-3.27316000	-1.38858100
C	-0.24461200	-2.54440600	-1.87878800
H	-0.36869500	-2.13211800	-2.87162800
O	-0.74982700	-0.50171400	1.91140200
O	-1.79856400	1.17154500	2.77247600
C	-4.35971800	0.15839100	1.52050500
H	-3.94579500	-0.71422600	2.03563600
H	-5.35166000	-0.10537300	1.15086600
H	-4.47000900	0.96097400	2.25525500
N	-1.51671800	0.47861800	1.82976100
C	0.96138400	0.23682900	-0.11614300
C	0.22484600	1.32648300	-0.31194400
Br	0.88346500	3.13509500	-0.31655300
C	2.40394400	0.09382100	0.10953100
C	2.86338100	-0.56124000	1.26005500
C	3.33227400	0.56769100	-0.82518700
C	4.22675200	-0.71866800	1.48118500
H	2.14520000	-0.93761200	1.98286100
C	4.69608400	0.39671000	-0.60558400
H	2.98358700	1.06292400	-1.72594000
C	5.14628000	-0.24215000	0.54772700
H	4.57284100	-1.21648700	2.38163600
H	5.40836900	0.76518800	-1.33719600
H	6.21038600	-0.37251700	0.71782100

VI

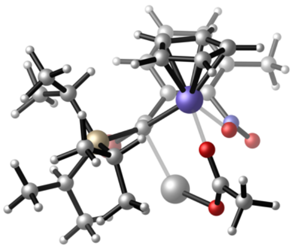

G = -1886.190392

E = -1886.79322670

C	-1.98050000	-1.44750400	0.11483400
C	-4.60874400	-1.81666100	1.04425400
C	-0.54087400	-1.32087700	-0.28928000
C	0.34077300	-0.39208700	0.13993800
C	-3.04960800	-0.65732800	-0.32598400
C	-4.37815400	-0.82276500	0.09508800
C	-2.27858300	-2.44227500	1.05000100
C	-3.57248500	-2.61283800	1.51986600
H	-5.61836900	-1.96168900	1.41378100
H	-3.77534700	-3.37763900	2.26167500
H	-1.48147600	-3.08236000	1.41297700
C	0.08294000	1.37182600	2.95505200
C	-0.95319700	0.38240800	2.91141500
C	-0.54058100	2.66161900	2.72723700
H	-0.85263900	-0.67838400	3.08687200
H	-0.00871700	3.60097700	2.65913600
C	-2.14508500	1.04177500	2.52467300
H	-3.09514600	0.55723300	2.33843700

C	-1.89535100	2.46488600	2.45634600
H	-2.61476300	3.22205700	2.17896900
H	1.11585900	1.20916400	3.23156000
Si	2.22851000	-0.61715500	0.56945000
Br	-0.01659300	-3.01759700	-1.20679100
C	3.01008400	1.12332500	0.72637100
H	2.20256300	1.78017000	1.06292300
C	2.03038700	-1.61033700	2.21371000
H	1.01179500	-1.38430900	2.54522600
C	3.22265500	-1.70127200	-0.64842500
H	2.79540000	-2.70354800	-0.53627700
O	0.44819200	2.75424100	-0.15753000
C	0.65003900	3.15801300	-1.34439500
O	0.55718200	2.48392900	-2.40016500
Ag	0.30184500	0.28900300	-2.19031200
C	1.05441900	4.60963600	-1.48787700
H	1.22598600	5.07551600	-0.51738500
H	1.95602200	4.67949100	-2.10154000
H	0.25669400	5.14660800	-2.01060500
Rh	-0.62709400	1.27524000	0.96388800
O	-1.91911700	1.28788600	-0.90933100
O	-3.40554000	0.54356900	-2.26307800
N	-2.78275100	0.44679500	-1.23186300
C	4.17178600	1.27447600	1.72350000
C	3.46936000	1.66441400	-0.63871100
C	3.18231400	-1.36600400	-2.14403300
C	4.68354100	-1.77659200	-0.16526800
C	2.96245800	-1.24689000	3.37690500
C	2.07878400	-3.13111100	1.98573000
H	4.64023300	2.25797000	1.59110200
H	3.84433800	1.21325200	2.76231000
H	4.95510700	0.52367600	1.57673100
H	2.79634600	-0.23098500	3.74393900
H	2.78221700	-1.92657500	4.21925000
H	4.01970100	-1.33901600	3.10673000
H	3.08208400	-3.46500500	1.69946800
H	1.81565700	-3.65473700	2.91287300
H	1.38342200	-3.46702100	1.21198000
H	4.40275200	1.18896600	-0.95718900
H	2.73690500	1.52019700	-1.43700700
H	3.66067900	2.74216800	-0.56761900
H	5.21192700	-0.83291600	-0.33717500
H	4.76155700	-2.00911100	0.90278400
H	5.22612100	-2.55858700	-0.71007700
H	3.71774400	-2.13203800	-2.71832900
H	2.15879400	-1.35015500	-2.54574100
H	3.64844300	-0.40425900	-2.36974200
C	-5.51095100	0.03767300	-0.39592700
H	-5.27241400	1.10428100	-0.33735300
H	-5.75582800	-0.19058800	-1.43619200
H	-6.39857600	-0.14331900	0.21238800

VI′

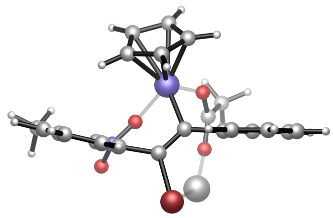

G = -1473.021077

E = -1473.32548338

C	1.70069000	-1.34288400	-0.52437200
C	4.49470500	-1.40947200	-0.80243100
C	0.21643900	-1.26377200	-0.48655000
C	-0.53764700	-0.17139400	-0.67305700
C	2.56101100	-0.79799900	0.43083500
C	3.95753700	-0.81132500	0.33658100
C	2.29489000	-1.95617800	-1.63074300
C	3.67518100	-1.98073600	-1.77066500
H	5.57239700	-1.42485000	-0.92670400
H	4.11690700	-2.44242000	-2.64712500
H	1.66304600	-2.39885600	-2.39367700
C	-0.00971100	2.70831400	-2.26294700
C	0.82622800	1.60061800	-2.61954200
C	0.79966700	3.64584200	-1.49830900
H	0.53717900	0.75086400	-3.22237100
H	0.44436400	4.57835400	-1.08176600
C	2.04930300	1.75848100	-1.92260800
H	2.87857400	1.06262000	-1.93218100
C	2.04240700	3.06548100	-1.27992300
H	2.84227300	3.46824200	-0.67339900
H	-1.01930200	2.88085000	-2.61049500
Br	-0.60341400	-3.06939300	-0.39591800
O	-0.95450700	2.42552900	0.89256600
C	-1.34432300	2.12656600	2.06740300
O	-1.44848000	0.98291800	2.56583800
Ag	-1.43202900	-1.13670500	1.85149200
C	-1.76676600	3.31260300	2.90964900
H	-2.66249200	3.76128000	2.46909400
H	-1.98350100	3.00925300	3.93413900
H	-0.98068800	4.07163100	2.90225200
Rh	0.41677900	1.62015600	-0.50436700

O	1.31156000	0.90383100	1.41535100
O	2.24089100	-0.54180800	2.69732900
N	1.99314500	-0.11871900	1.59137900
C	4.84922500	-0.19422300	1.37988800
H	4.50439600	0.80205600	1.67494400
H	4.88743100	-0.81390700	2.28002500
H	5.86324700	-0.09763700	0.98832600
C	-1.94097800	-0.19010300	-1.15098600
C	-3.00831500	0.41945500	-0.47909400
C	-2.18687200	-0.79711200	-2.39345900
C	-4.29288300	0.38384600	-1.01711300
H	-2.83863100	0.92824200	0.46070300
C	-3.46524200	-0.81124600	-2.93642800
H	-1.36490200	-1.25665000	-2.93413900
C	-4.52592400	-0.22642900	-2.24592300
H	-5.11050500	0.85009400	-0.47635400
H	-3.63377300	-1.28350400	-3.89917600
H	-5.52567300	-0.24150300	-2.66811400

VII

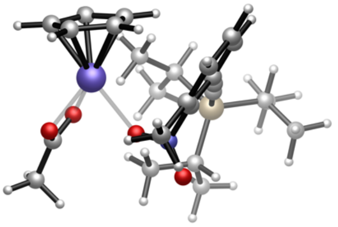

G = -1727.296224

E = -1727.296224

C	-0.66620300	2.49683700	-0.30460600
C	-3.18146800	3.69479300	-0.00152900
C	0.60849400	1.86011700	-0.40361000
C	1.67102000	1.26744000	-0.41297700
C	-1.53452000	2.17175700	0.74600100
C	-2.78801500	2.74313300	0.94349600
C	-1.10206900	3.45998900	-1.21985200
C	-2.35254900	4.04763900	-1.06335300
H	-4.15506400	4.16221900	0.10499500
H	-2.68604700	4.79182400	-1.77834600
H	-0.45201500	3.74073500	-2.04055100
C	-1.68910200	-0.11143900	-2.28641200
C	-2.94722100	0.27143500	-1.70243400
C	-1.71596400	-1.51212300	-2.50020300
H	-3.23007900	1.27178800	-1.40460100
H	-0.89726100	-2.11296900	-2.87423400
C	-3.73586500	-0.88927900	-1.54630500
H	-4.71357500	-0.93746400	-1.08616900
C	-2.96240900	-2.01627600	-2.00096200
H	-3.27627800	-3.05113800	-1.99576900
H	-0.86116800	0.54686200	-2.50930000
Si	3.28336200	0.34400000	-0.28268800
C	3.26387400	-1.02030000	-1.60614500
H	3.64429900	-0.53384700	-2.51650000
C	4.63056500	1.62361800	-0.65941800
H	4.28868200	2.11714900	-1.58118300
C	3.30537900	-0.26183200	1.51665000
H	3.05490700	0.63764300	2.09899900
O	-2.52462900	-2.29910900	1.31976500
C	-1.38053300	-2.85250600	1.35784900
O	-0.58293000	-2.58596900	0.40013100
C	-0.96352100	-3.72414900	2.48996100
H	-0.22774500	-4.45583400	2.15388400
H	-0.49972000	-3.08947000	3.25239800
H	-1.82998500	-4.22017100	2.92937500
Rh	-1.92425900	-1.15274100	-0.39239300
O	-1.02744600	-0.02452400	1.34908400
O	-0.76095500	1.51536500	2.81296700
N	-1.07972600	1.16344900	1.70341700
C	4.21276700	-2.18144000	-1.26856400
C	1.85499100	-1.54846700	-1.91432700
C	2.21286100	-1.30664600	1.78786700
C	4.67401800	-0.75904900	2.00442500
C	6.00220500	1.00027800	-0.95604400
C	4.72787200	2.69276500	0.43779800
H	4.25716700	-2.89352900	-2.10175200
H	5.23537300	-1.84608400	-1.06740200
H	3.86460900	-2.73301500	-0.38875100
H	5.95945700	0.29377200	-1.79155600
H	6.72806900	1.77848100	-1.22221300
H	6.40396700	0.46610300	-0.08829100
H	5.08291300	2.26411500	1.38203200
H	5.43458700	3.48113600	0.15173200
H	3.76050500	3.17005800	0.63068000
H	1.37708800	-1.97976200	-1.02766500
H	1.20170700	-0.75443600	-2.29005500
H	1.89943300	-2.33266200	-2.68106500
H	5.01540700	-1.63412800	1.44137500
H	5.44635200	0.01249000	1.92691600
H	4.61457700	-1.05520600	3.05933700
H	2.14892800	-1.52349500	2.86171500
H	1.22484500	-0.97192400	1.45916100
H	2.42540400	-2.25172500	1.27674600

C	-3.67370000	2.34757900	2.09242500
H	-3.76418300	1.25961000	2.17833700
H	-3.27613400	2.72531900	3.03914200
H	-4.67411100	2.76139700	1.95602700

TS_{II-III}

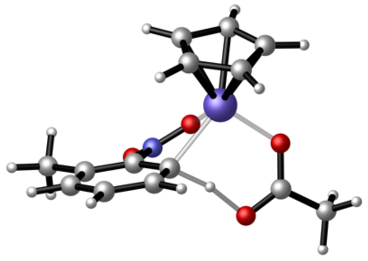

G = -1006.943197

E = -1007.16505848

O	-0.63014400	2.44815900	1.04032300
C	-1.75988700	2.30739200	0.50991300
O	-2.15394200	1.22567800	-0.03838800
C	1.85847200	0.39964700	-0.13183600
C	0.87760000	0.31434100	0.87344200
C	3.20290100	0.05587000	0.03322100
C	1.28212800	-0.14479800	2.12634600
C	3.53852700	-0.42033300	1.31017400
C	2.60868600	-0.51768500	2.33794000
H	0.02218700	1.26093600	0.92499600
H	0.56060200	-0.19145500	2.93545300
H	4.56700600	-0.71693200	1.49047100
H	2.92388000	-0.88341200	3.30963500
Rh	-0.95674700	-0.47459400	-0.15978800
C	-0.56916900	-2.51632700	-0.81145200
H	0.29106200	-2.79630500	-1.40417900
C	-1.84370600	-2.06913700	-1.32346900
H	-2.10019700	-1.94975500	-2.36761900
C	-2.68571700	-1.78332800	-0.22199100
H	-3.68622800	-1.37631900	-0.27881800
C	-0.63608700	-2.48721100	0.59885800
H	0.17189400	-2.72558500	1.27667000
C	-1.92987400	-1.98206300	0.97888600
H	-2.28423500	-1.83159900	1.98918000
C	-2.73218400	3.45322900	0.50640800
H	-2.29948600	4.32940800	0.98794100
H	-2.99991300	3.68985200	-0.52670800
H	-3.64539700	3.15328800	1.02683700
O	0.14592000	0.68611600	-1.66270600
O	2.08501800	1.43010500	-2.20100600
C	4.25699800	0.13941400	-1.03348600

H	3.92468000	-0.31109100	-1.97235600
H	5.15797200	-0.37785600	-0.69918900
H	4.51203600	1.18107400	-1.24498800
N	1.36439800	0.86970700	-1.41148200

TS_{IV-V}

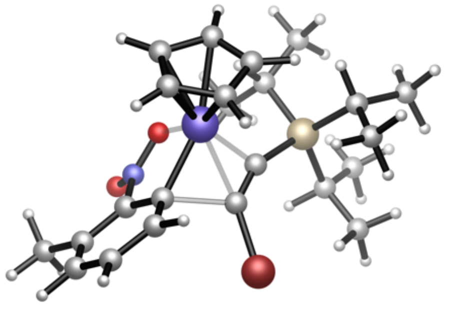

G = -1512.060737

E = -1512.50858986

C	2.61243700	-0.49484300	1.00251400
C	2.17506600	-0.09497400	-0.27358800
C	3.88623500	-1.03212200	1.26556800
C	3.07846800	-0.19441700	-1.33422000
C	4.74526900	-1.10035200	0.17014100
C	4.35825400	-0.67317100	-1.10056000
H	2.77349600	0.09299900	-2.33450800
H	5.74657700	-1.48980700	0.32104000
H	5.06889400	-0.72550700	-1.91899900
Rh	0.65886900	1.34392300	-0.21539200
C	1.18141800	3.47299700	0.06409800
H	1.69643500	3.82225400	0.94923100
C	-0.26176900	3.36630200	-0.08024000
H	-0.99098000	3.64116700	0.66899400
C	-0.52876800	2.86056300	-1.36306300
H	-1.49893300	2.65269800	-1.78920200
C	1.78365800	3.04905200	-1.13286900
H	2.84446400	2.98156000	-1.32895800
C	0.73484500	2.57268100	-1.98959400
H	0.86353700	2.15294100	-2.97847700
O	0.82138700	0.65516300	1.85185300
O	1.70657900	-0.84845200	3.10640700
C	4.36281300	-1.47435200	2.62003600
H	4.20886200	-0.69996200	3.37671200
H	5.42854500	-1.70524700	2.57116100
H	3.82616800	-2.36519700	2.95530400
N	1.68211300	-0.24137200	2.05874400
Si	-2.46596300	-0.29499800	0.32237000
C	-2.95042600	-2.10412000	0.62893300

C	-2.52911900	0.74903800	1.90704800
C	-3.39180300	0.54608200	-1.10849900
H	-2.16652700	-2.49303800	1.29543700
H	-1.81097000	1.56138300	1.72525500
H	-3.25482200	1.62337800	-0.93448000
C	-2.95223200	-2.95354000	-0.64957700
C	-4.29703300	-2.25061600	1.35871600
C	-2.81991300	0.20456500	-2.49241100
C	-4.90319500	0.26652800	-1.07523900
C	-2.05024100	-0.01928600	3.14770300
C	-3.90024900	1.39402500	2.15649900
H	-3.33766400	0.77251500	-3.27508500
H	-1.75078500	0.42739900	-2.57025200
H	-2.94831200	-0.85864500	-2.72235500
H	-5.11368700	-0.78498600	-1.29656300
H	-5.35604500	0.50018600	-0.10705900
H	-5.41819700	0.86853300	-1.83354400
H	-4.48837500	-3.30692200	1.58397300
H	-4.32863900	-1.70378900	2.30522300
H	-5.12906700	-1.89563400	0.74236600
H	-3.12066500	-4.01033200	-0.40979100
H	-3.75309000	-2.64317800	-1.32957300
H	-2.00752000	-2.88770800	-1.19698000
H	-4.68347400	0.64183200	2.30275700
H	-3.87200000	2.01196300	3.06252400
H	-4.20746000	2.04056900	1.32783200
H	-1.07828900	-0.49602400	2.99452100
H	-1.94501200	0.66268600	4.00018500
H	-2.76440700	-0.79563200	3.44052100
C	-0.65188400	-0.30414300	-0.20538900
C	0.39944900	-0.83005500	-0.70743300
Br	0.77017400	-2.29823300	-1.80989900

TS_{VI-VII}

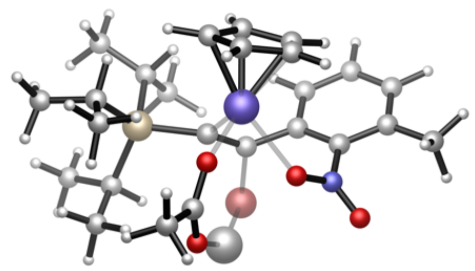

G = -1886.292381

E = -1886.78329591

C	-1.90911700	-0.98064500	0.99656400
C	-4.49749200	-1.01623600	2.06600300
C	-0.50897800	-0.94798500	0.51321700
C	0.44613300	-0.05362500	0.49034300
C	-3.03626000	-0.65461100	0.22988800
C	-4.34625400	-0.66140800	0.72400700
C	-2.11897200	-1.34191600	2.32703800
C	-3.40426100	-1.35410900	2.85479500
H	-5.49404400	-1.01609000	2.49471200
H	-3.55291400	-1.62083900	3.89559900
H	-1.26859200	-1.60701900	2.94512400
C	0.03843200	3.16733700	1.64016100
C	-0.74861000	2.19645000	2.34396200
C	-0.85208300	3.90877000	0.76815300
H	-0.39700900	1.50044900	3.09156700
H	-0.54613000	4.69012300	0.08598600
C	-2.05469900	2.24294900	1.80130400
H	-2.87470300	1.59251600	2.07913100
C	-2.12423300	3.34121600	0.85516700
H	-2.99303800	3.61754700	0.27337000
H	1.08424100	3.38553300	1.80463500
Si	2.32482400	-0.14376600	0.93688900
Br	-0.15091700	-3.04262100	-0.07295200
C	3.23348400	1.53652400	0.88423300
H	2.85605700	2.07514200	1.76606000
C	2.13979000	-0.62333200	2.78798000
H	1.31395800	0.01768500	3.13057100
C	3.21335000	-1.45674200	-0.11617800
H	2.48707300	-2.27029400	-0.22451700
O	0.26075700	2.23455800	-1.61664500
C	0.70115500	1.76714100	-2.71015500
O	0.72745400	0.57150500	-3.08720800
Ag	0.19809300	-1.36773600	-2.20110800
C	1.27219900	2.78918200	-3.67216800
H	2.33337100	2.57289300	-3.82825100
H	0.77310800	2.69710500	-4.64044600
H	1.16061100	3.80431200	-3.29107900
Rh	-0.64105000	1.74199000	0.23943700
O	-1.94155700	0.64339900	-1.34259300
O	-3.49282100	-0.67500300	-2.02828800
N	-2.82049100	-0.21185100	-1.13361500
C	4.75006000	1.33545400	1.08958500
C	3.04362000	2.44453400	-0.33566200
C	3.56529100	-0.94779700	-1.52370500
C	4.45957000	-2.06380000	0.55049700
C	3.34675200	-0.29551300	3.67979900
C	1.71226000	-2.07776700	3.02013800
H	5.22534100	2.30160100	1.29771500

H	5.00696800	0.66223200	1.90948200
H	5.21395300	0.93969500	0.18036300
H	3.61143100	0.76543200	3.65435300
H	3.11739100	-0.54690400	4.72294700
H	4.23189800	-0.87518600	3.39570200
H	2.49606000	-2.78250100	2.72396000
H	1.50610600	-2.24991300	4.08370900
H	0.81289200	-2.34910200	2.46406200
H	3.31858600	1.93247100	-1.26421100
H	2.02036600	2.79584700	-0.45009600
H	3.69564100	3.32303600	-0.24658000
H	5.26130400	-1.32859300	0.66393100
H	4.24802200	-2.48057500	1.53929000
H	4.85261300	-2.87850900	-0.07015800
H	3.89177300	-1.77753500	-2.16288000
H	2.72536600	-0.45242900	-2.02526300
H	4.38492800	-0.22229800	-1.48921200
C	-5.54493500	-0.26206300	-0.09262100
H	-5.35836800	0.64387900	-0.67647100
H	-5.82700200	-1.05422000	-0.79066900
H	-6.39023300	-0.07169100	0.57095300

TS$_{CH-4}$

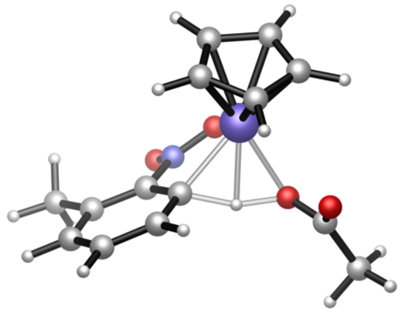

G = -1006.921524

E = -1007.14179954

C	-2.19402900	-1.01724800	1.43976000
H	-2.45955900	-0.30427900	2.20577300
C	-2.91220100	-1.23746000	0.21281600
H	-3.76695100	-0.66590000	-0.12214900
C	-2.22969400	-2.22183600	-0.52860600
H	-2.48305700	-2.56589400	-1.52189200
C	-1.10301400	-1.94395500	1.47976300
H	-0.36355000	-2.02329600	2.26504800
C	-1.08801700	-2.64700400	0.24699200
H	-0.35161600	-3.37738100	-0.05998400
Rh	-0.86486500	-0.55108000	-0.15462100
C	2.01787600	0.14456100	-0.17614400

C	1.07095400	0.90900000	1.89170800
C	3.32646300	0.16305100	0.31323000
C	2.36136300	0.95194000	2.42370400
H	0.21678700	1.20825100	2.48975400
C	3.45107000	0.57793500	1.65072600
H	2.51701700	1.27063400	3.44891300
H	4.44498500	0.60249300	2.08598200
O	-1.02701100	1.52313100	-0.84691100
O	-2.63256600	1.94867300	0.66970800
C	-1.95517300	2.30932200	-0.26901900
C	-2.04977900	3.65908600	-0.92303900
H	-1.09129300	4.17693500	-0.82321700
H	-2.24671300	3.53735100	-1.99147600
H	-2.84088000	4.24816300	-0.45995300
C	0.88273100	0.49714800	0.57671300
H	-0.06610200	1.22689100	-0.07912600
O	0.55002700	-0.58379700	-1.81873900
O	2.59215200	-0.27710200	-2.38747900
N	1.73358800	-0.26285500	-1.54000900
C	4.55429800	-0.23885100	-0.45274800
H	5.40347600	-0.30488800	0.22964400
H	4.42516400	-1.20512100	-0.94691300
H	4.78818300	0.49324100	-1.23017300

TS$_{CH-Inter}$

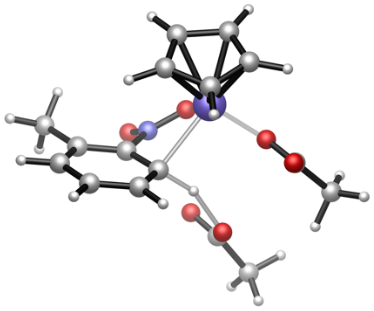

G = -1235.454466

E = -1235.72092182

C	0.23567100	-2.77241300	-1.00804000
H	0.52349300	-2.66933700	-2.04409700
C	1.10695300	-3.08749200	0.07302400
H	2.17755400	-3.20792500	-0.00250800
C	0.34146100	-3.05267800	1.27546400
H	0.71775800	-3.20850100	2.27727500
C	-1.10571200	-2.64730200	-0.47908700
H	-1.98886600	-2.39212300	-1.04891600
C	-1.03615400	-2.80897500	0.91435300
H	-1.85619800	-2.69773900	1.61143500
Rh	0.26879200	-1.13190800	0.33535400

C	-1.75040800	0.88223900	-0.00836900
C	-1.08022700	0.25542400	-2.21276500
C	-3.10885700	0.88136700	-0.32736200
C	-2.42218400	0.22845800	-2.58308100
H	-0.30379200	0.04955700	-2.94275700
C	-3.40981600	0.53573000	-1.65337200
H	-2.70430200	-0.02651600	-3.59962800
H	-4.45386800	0.50395900	-1.95043900
O	2.02533400	-0.16412500	0.60920900
C	2.96566100	-0.29215000	-0.28061300
O	2.93029400	-1.04421100	-1.25236400
C	4.14253100	0.62623400	-0.03145100
H	4.27563400	0.83866800	1.03094100
H	5.05478800	0.19721200	-0.45037400
H	3.92338300	1.56653600	-0.54632300
O	1.41682700	2.12558600	-1.04768300
O	0.37094300	3.11285000	0.67447800
C	1.28338800	3.04152500	-0.16413400
C	2.37929300	4.10517800	-0.15401900
H	2.83765700	4.22145100	-1.13914400
H	1.98211100	5.06316400	0.19097300
H	3.16146600	3.79540100	0.54872700
C	-0.71024800	0.57385100	-0.90173700
H	0.36979800	1.11480100	-0.79583800
O	-0.40729500	0.37935800	1.80104600
O	-1.90780100	1.89037500	2.07916600
N	-1.33893100	1.09762100	1.37295300
C	-4.22025600	1.18305700	0.63960400
H	-5.17575100	0.87933300	0.20719600
H	-4.09086100	0.66060900	1.59137100
H	-4.26610100	2.25299700	0.85922100

TS$_{CH2}$

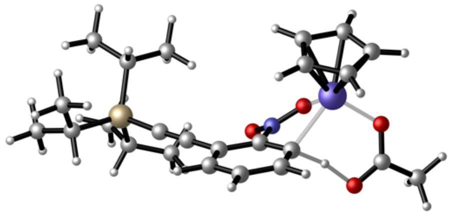

G = -1687.979701

E = -1688.44114878

O	3.68802900	1.84955000	-1.72543000
C	4.41240200	2.10942400	-0.73127000
O	4.45115700	1.39269600	0.32199200
C	0.83540700	0.04262800	-1.00797900
C	2.15225100	-0.24174400	-1.39615600
C	-0.29655200	-0.57376500	-1.55743100
C	2.33214300	-1.18839600	-2.40669800
C	-0.05501600	-1.53259400	-2.55830000
C	1.23401000	-1.82818300	-2.98077800
H	2.98822600	0.72805700	-1.44294100
H	3.33632100	-1.40806800	-2.75405600
H	-0.90559100	-2.04086100	-2.99825900
H	1.38016500	-2.56576800	-3.76257400
Rh	3.28462500	-0.31073400	0.55517100
C	2.59193000	-1.90754200	1.85866300
H	1.57201000	-1.96755700	2.21428300
C	3.66439100	-1.17383200	2.49032700
H	3.59411300	-0.61389900	3.41277600
C	4.82377300	-1.32207400	1.68535300
H	5.77997900	-0.84702100	1.85974100
C	3.08825300	-2.47312900	0.66633200
H	2.51502300	-3.04204900	-0.05285300
C	4.46815900	-2.07508300	0.52366600
H	5.12669700	-2.33555000	-0.29323000
C	5.26709100	3.34403900	-0.75329600
H	5.71483100	3.46948500	-1.74083200
H	4.62195400	4.20761400	-0.56275900
H	6.03814900	3.30069300	0.01534400
O	1.66496200	1.14216000	0.84837700
O	-0.28609900	1.70914600	0.14933800
N	0.70002000	1.02408700	0.05026100
C	-1.63207300	-0.33920700	-1.11724000
C	-2.78327200	-0.18813000	-0.75383800
Si	-4.50265400	0.06076700	-0.08550000
C	-5.61865900	-1.05378900	-1.13995300
C	-4.94667600	1.89327000	-0.31877600
C	-4.36277600	-0.51993300	1.71773800
H	-3.88105600	-1.50583000	1.63805600
C	-5.34250300	-2.54092400	-0.87709900
H	-5.92748400	-3.17198100	-1.55718500
H	-4.28494300	-2.78966500	-1.02207700
H	-5.61487400	-2.82571500	0.14586600
C	-5.71242000	-0.72346800	2.42133300
H	-5.56007500	-1.12880600	3.42948800
H	-6.26023200	0.21829400	2.52888700
H	-6.35636400	-1.42455700	1.88184000
C	-3.43979300	0.38315600	2.54906900
H	-3.26404300	-0.05260200	3.54039900
H	-2.46450700	0.52904600	2.07146800
H	-3.88562500	1.37220900	2.70283000
C	-3.74352900	2.84032100	-0.20359900

H	-4.05500600	3.87797000	-0.37657100
H	-3.29214100	2.79629000	0.79359900
H	-2.96204100	2.60096900	-0.93034300
C	-6.07377000	2.34276100	0.62489900
H	-5.73262200	2.35654500	1.66599300
H	-6.39804400	3.36051600	0.37591600
H	-6.95447400	1.69466600	0.57249500
H	-5.32676800	1.95926600	-1.34920000
C	-7.11508800	-0.73049300	-1.02268500
H	-7.33143600	0.30593400	-1.30165100
H	-7.70059700	-1.37759600	-1.68754700
H	-7.49164400	-0.88746100	-0.00603900
H	-5.31949600	-0.83714500	-2.17629100

TS_{CH2-4}

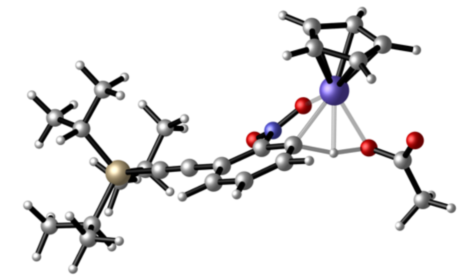

G = -1687.958410

E = -1688.41658715

C	4.95886900	-0.74356400	-1.46788300
H	5.50644200	0.12927800	-1.79086200
C	5.31633300	-1.60078100	-0.37054000
H	6.13971400	-1.43650100	0.31051300
C	4.32703000	-2.59738000	-0.24862300
H	4.27676500	-3.35701600	0.51956400
C	3.77521200	-1.27896600	-2.07370500
H	3.24470500	-0.85455000	-2.91509100
C	3.35673400	-2.39142300	-1.29801700
H	2.46284300	-2.97889300	-1.46007600
Rh	3.37129300	-0.64901800	-0.05247900
C	0.69984900	0.65110800	-0.11016200
C	2.17627200	1.94561400	-1.48356800
C	-0.44365400	1.18502400	-0.71549000
C	1.06167800	2.50678800	-2.11157100
H	3.17766700	2.25247700	-1.76675100
C	-0.21848100	2.12863800	-1.73893300
H	1.19154600	3.24189500	-2.89840200
H	-1.08147600	2.55862200	-2.23483800
O	3.68143100	0.86736600	1.49444600
O	5.68800500	1.40592500	0.63581700
C	4.82531100	1.57937700	1.46958300
C	4.89960000	2.59112000	2.57817200
H	4.08600300	3.31401400	2.46489500
H	4.76453500	2.09563300	3.54317500
H	5.85881900	3.10743000	2.55325800
C	2.00715000	1.00541600	-0.47047500
H	2.88634600	1.15255300	0.56072500
O	1.61535600	-1.01999700	1.20424400
O	-0.42354800	-0.46826300	1.58100600
N	0.59501200	-0.33008800	0.95258900
C	-1.78561700	0.81718500	-0.40901400
C	-2.95852500	0.54772100	-0.22945700
Si	-4.73359700	0.09782000	0.10698400
C	-5.24389500	-1.13538500	-1.24917600
H	-5.52454700	-0.50657800	-2.10778400
C	-5.73697200	1.70785700	-0.00698800
C	-4.71428900	-0.59599300	1.87253000
C	-6.47574200	-1.96755900	-0.85620800
H	-7.32110000	-1.35042300	-0.53771400
H	-6.81277800	-2.57405700	-1.70585700
H	-6.24027800	-2.65773800	-0.03884200
C	-4.10404700	-2.05919000	-1.70223500
H	-4.44506400	-2.72285800	-2.50651700
H	-3.24311800	-1.49748700	-2.07739300
H	-3.75391600	-2.69634500	-0.88220100
C	-7.24039100	1.44792700	-0.19449100
H	-7.79714500	2.39240300	-0.16598200
H	-7.43950000	0.98236800	-1.16671100
H	-7.65905400	0.79577100	0.57843500
C	-3.94101500	-1.91935000	1.96920700
H	-3.81365100	-2.21668200	3.01731000
H	-4.47294400	-2.73228200	1.46241500
H	-2.94274800	-1.84303500	1.52513700
C	-6.10259700	-0.71439700	2.51732500
H	-6.01496900	-1.09357900	3.54320100
H	-6.61224600	0.25296300	2.56975800
H	-6.75058800	-1.40652300	1.96913000
H	-4.15094800	0.15775700	2.44285900
H	-5.59729300	2.21755300	0.95740000
C	-5.22463300	2.63900900	-1.11577200
H	-5.82300800	3.55761200	-1.15600800
H	-4.18058800	2.92755300	-0.96076300
H	-5.29635400	2.16256400	-2.10150400

$TS_{CH2-Inter}$

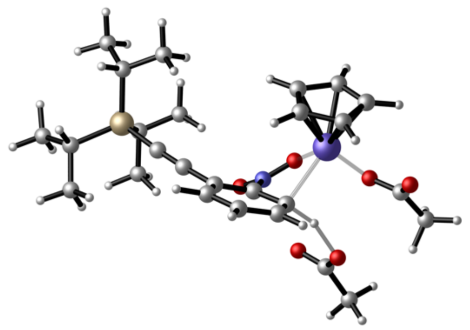

G = -1916.493471

E = -1916.99837589

C	-3.25181600	-2.65438200	0.99183600
H	-3.97400500	-2.48225900	1.77640500
C	-3.55140500	-2.92223900	-0.37412000
H	-4.53973300	-2.92868400	-0.81005400
C	-2.31624900	-3.01166200	-1.08191500
H	-2.19982700	-3.16283600	-2.14655100
C	-1.81295400	-2.68194000	1.14519400
H	-1.27372700	-2.49111100	2.06303200
C	-1.24296800	-2.89205000	-0.12064100
H	-0.18596900	-2.89279700	-0.35188600
Rh	-2.47348700	-1.07791000	-0.20135100
C	-0.47904900	0.65877400	0.85954700
C	-1.98246300	0.33593700	2.67758900
C	0.64342600	0.41419300	1.65992500
C	-0.89597600	0.11587200	3.52039200
H	-2.99212700	0.31761000	3.07595500
C	0.39913000	0.14367000	3.01677100
H	-1.05322600	-0.08856900	4.57417700
H	1.24527100	-0.06032000	3.66363900
O	-3.80884800	0.08698800	-1.17382700
C	-5.05125800	0.10223800	-0.78610600
O	-5.54092200	-0.63591000	0.06601800
C	-5.86456900	1.17883000	-1.47123400
H	-5.51967600	1.35784300	-2.49143700
H	-6.92480000	0.91958700	-1.46708600
H	-5.71872000	2.09822200	-0.89562700
O	-3.59995900	2.37173200	0.61087100
O	-1.85339400	3.11037900	-0.58590400
C	-3.01421700	3.21090900	-0.16077000
C	-3.86404900	4.40387700	-0.58964900
H	-4.62291200	4.64458600	0.15857000
H	-3.23480700	5.27663000	-0.78028500
H	-4.37878700	4.15181100	-1.52406800
C	-1.80108700	0.60133900	1.31348000
H	-2.68893500	1.27130500	0.79868700
O	-1.08172300	0.23379300	-1.32426000
O	0.59902800	1.53649200	-0.99670100
N	-0.29560900	0.85259200	-0.57429700
C	1.97166300	0.32238500	1.14321300
C	3.10097900	0.17146500	0.71705000
Si	4.77001600	-0.08082700	-0.04965200
C	5.01505200	-1.96036800	-0.25229100
H	5.40782500	-2.30353900	0.71582200
C	6.07214900	0.62253000	1.14612200
H	6.24125700	-0.17959300	1.87946700
C	4.73688200	0.82265400	-1.71711300
C	3.71601400	-2.73429300	-0.52139100
H	3.91989800	-3.80872300	-0.61019300
H	2.98788000	-2.59958300	0.28487500
H	3.23911600	-2.41549900	-1.45468600
C	6.07021000	-2.28450400	-1.32164200
H	7.02933400	-1.79420000	-1.12594200
H	6.25850600	-3.36445000	-1.36239400
H	5.73815400	-1.97533300	-2.31924800
C	7.40992600	0.89844100	0.44118900
H	8.16183500	1.23871900	1.16368400
H	7.81381200	0.00975300	-0.05466100
H	7.30887800	1.68378600	-0.31685300
C	5.60255700	1.86016000	1.92466600
H	4.68540100	1.66460800	2.48918100
H	6.37248700	2.17843500	2.63846400
H	5.40423200	2.70788100	1.26006100
C	3.67297700	0.25344300	-2.66757900
H	3.67824500	0.79726900	-3.62025800
H	3.83998600	-0.80437000	-2.89297200
H	2.66743300	0.35335400	-2.24224000
C	4.54775500	2.33749700	-1.55087000
H	5.36721600	2.79871700	-0.99112800
H	4.50237100	2.82920500	-2.53061900
H	3.61175300	2.56769700	-1.02840300
H	5.72488100	0.64975400	-2.16892700

$TS_{Br}{}^{Cp*}{}_{II\text{-}III}$

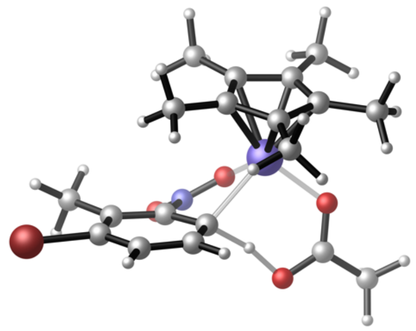

G = -1215.980392

E = -1216.32124713

O	1.05047800	2.86594900	-1.12209800
C	2.27701100	2.66193900	-0.88624400
O	2.73069700	1.56496700	-0.44450400
C	-1.25412700	0.95170600	0.52768400
C	-0.50370000	0.84060100	-0.64931400
C	-2.59928900	0.59370100	0.69068700
C	-1.18640800	0.39739800	-1.78162300
C	-3.20891700	0.15186200	-0.49665200
C	-2.53368000	0.06743000	-1.71252900
H	0.40474300	1.78501200	-0.88041100
H	-0.66536800	0.32130300	-2.73062200
H	-3.05875200	-0.26893300	-2.59958800
Rh	1.47509800	-0.06546000	0.04177500
C	1.38956200	-1.90002600	1.17039900
C	2.76945100	-1.52054700	0.92578700
C	2.97141000	-1.51847100	-0.49252500
C	0.74914600	-2.10989200	-0.08185100
C	1.71758000	-1.83382200	-1.12513400
C	3.25067000	3.78324800	-1.10523000
H	2.86651800	4.49277800	-1.83839800
H	3.38495500	4.30462900	-0.15169300
H	4.21873900	3.39031800	-1.41835500
O	0.72104400	1.28451300	1.69948300
O	-1.05773900	2.20285000	2.47719800
C	-3.32544000	0.62288100	2.00648100
H	-2.64829500	0.47197000	2.84704600
H	-4.08379500	-0.16014200	2.03575300
H	-3.82764900	1.58426500	2.15017000
N	-0.50718300	1.51064800	1.65306200
C	0.74864600	-2.03301000	2.50720700
C	3.80031100	-1.26389800	1.96995300
C	-0.65164600	-2.58802300	-0.26026900
C	1.49666300	-1.95398600	-2.59358300
C	4.24251000	-1.20336500	-1.19842600
H	-1.34179200	-2.05502100	0.39767400
H	-0.99353500	-2.46635100	-1.28730200
H	-0.69764300	-3.65212700	-0.00654800
H	-0.33035900	-1.87821300	2.44415400
H	0.92530900	-3.04458900	2.88888200
H	1.16632100	-1.32425700	3.22491300
H	3.35292400	-0.83982900	2.87098700
H	4.28364400	-2.20873800	2.24115900
H	4.56823900	-0.57749400	1.60898700
H	4.88703200	-0.56493400	-0.59302600
H	4.77473300	-2.14049200	-1.39568000
H	4.05723700	-0.71080000	-2.15435700
H	0.43668600	-1.90094300	-2.84485800
H	2.02154100	-1.16479900	-3.13653400
H	1.88525400	-2.91804700	-2.93931600
Br	-5.05343400	-0.35211400	-0.50154000

$TS_{CF3}{}^{Cp*}{}_{II-III}$

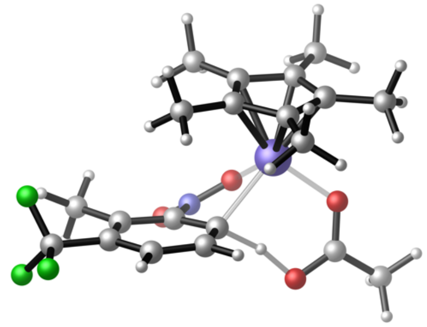

G = -1540.339745

E = -1540.69260690

O	1.05049500	2.88155000	-1.09877800
C	2.27601000	2.65783600	-0.87470800
O	2.71691700	1.54992000	-0.44744600
C	-1.28972600	0.96324900	0.51406600
C	-0.53732800	0.87476300	-0.66490400
C	-2.63156000	0.59540300	0.66160100
C	-1.21543800	0.46090800	-1.80779300
C	-3.25036900	0.17512200	-0.53263400
C	-2.56325400	0.12750900	-1.74313100
H	0.39213500	1.80522400	-0.87299500
H	-0.69220500	0.40814100	-2.75689600
H	-3.08306400	-0.18888200	-2.63947300
Rh	1.43741000	-0.06095600	0.02838900
C	1.27456300	-1.88980200	1.15600900
C	2.66998000	-1.52681000	0.97278200
C	2.94047700	-1.53821300	-0.43354800
C	0.69312900	-2.09990500	-0.12281500

C	1.71197500	-1.83829800	-1.12139900
C	3.25888800	3.77041000	-1.09572100
H	2.96272500	4.37373200	-1.95530500
H	3.24952400	4.41404100	-0.20974100
H	4.26536500	3.37432200	-1.23082700
O	0.68585200	1.27882000	1.69122300
O	-1.09980400	2.15731600	2.49817100
C	-3.35349500	0.59762200	1.98455400
H	-2.67364400	0.37772000	2.80821400
H	-4.14430500	-0.14966800	1.99900700
H	-3.80100800	1.57599700	2.17719200
N	-0.54497600	1.49493900	1.65266000
C	0.57748100	-2.01203800	2.46516100
C	3.64344300	-1.26468900	2.06868400
C	-0.70185600	-2.56159900	-0.37262300
C	1.54362900	-1.97643500	-2.59454100
C	4.24620600	-1.23961000	-1.08128000
H	-1.41560300	-2.04135900	0.27006100
H	-0.99710000	-2.41115500	-1.41037700
H	-0.76509100	-3.63164200	-0.14916300
H	-0.49967900	-1.87507600	2.35379000
H	0.75245600	-3.01481500	2.87024000
H	0.95529100	-1.28719200	3.18915500
H	3.16220400	-0.75777700	2.90776800
H	4.04423600	-2.21717600	2.43239500
H	4.47683900	-0.65045500	1.72385700
H	4.87274900	-0.61174100	-0.44677600
H	4.77302600	-2.18319700	-1.26043000
H	4.10869000	-0.73920200	-2.04115100
H	0.51745100	-1.76307600	-2.89804200
H	2.21490200	-1.30885000	-3.13763800
H	1.77793000	-3.00639100	-2.88565800
C	-4.69728600	-0.26449600	-0.52239900
F	-4.85791300	-1.41664900	0.15949700
F	-5.49973100	0.64831100	0.04830500
F	-5.16712400	-0.47827200	-1.75901000

TS$_{Cl}{}^{Cp*}{}_{II-III}$

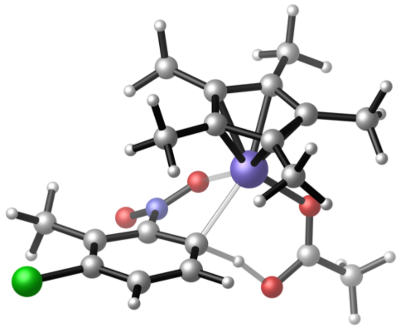

G = -1662.981204

E = -1663.32180154

O	0.61599000	2.88618500	-1.06665200
C	1.82659600	2.73901900	-0.72928600
O	2.29522100	1.66283300	-0.25293600
C	-1.70682300	0.83298600	0.43678100
C	-0.89048500	0.79885900	-0.70103000
C	-3.04184000	0.41546900	0.50904900
C	-1.49243300	0.37281700	-1.88528800
C	-3.56509100	-0.00739400	-0.72485600
C	-2.82505600	-0.01651400	-1.90398100
H	-0.00721700	1.78016100	-0.86515100
H	-0.91791700	0.35768300	-2.80595800
H	-3.29301100	-0.34035100	-2.82700600
Rh	1.08119600	-0.04759600	0.06014900
C	1.01066800	-1.92923300	1.10807300
C	2.38450600	-1.48123800	0.96061100
C	2.66739200	-1.41279600	-0.44179900
C	0.45349500	-2.11808600	-0.18671500
C	1.46518400	-1.75043900	-1.15845200
C	2.76361100	3.90399800	-0.86983200
H	2.37301200	4.63328800	-1.57957500
H	2.86397400	4.38286400	0.10959600
H	3.75107700	3.55755900	-1.17909100
O	0.18955300	1.19891100	1.72382100
O	-1.67006200	1.98909900	2.45283600
C	-3.85192800	0.36811000	1.77359500
H	-3.22630100	0.19586700	2.64933700
H	-4.59241800	-0.43024700	1.71938400
H	-4.38353500	1.31234000	1.92443600
N	-1.04519100	1.36819100	1.62473100
C	0.30111100	-2.13646600	2.40021100
C	3.33547000	-1.21647500	2.07660500
C	-0.90975000	-2.65008700	-0.46952600
C	1.33554400	-1.81689000	-2.64100000
C	3.95985600	-1.01634100	-1.06285700

H	-1.66284800	-2.17276100	0.16210400
H	-1.19076300	-2.50428900	-1.51175100
H	-0.92163100	-3.72392100	-0.25606700
H	-0.77864400	-2.02779800	2.28072000
H	0.50292600	-3.15149100	2.75965400
H	0.64632700	-1.43408100	3.16135800
H	2.82216300	-0.78605700	2.93885400
H	3.79724100	-2.15842200	2.39172000
H	4.12776000	-0.53211300	1.76945500
H	4.53667600	-0.36260500	-0.40757900
H	4.54823400	-1.92108700	-1.25148700
H	3.80468400	-0.50899100	-2.01627500
H	0.29035100	-1.82215500	-2.95133400
H	1.83560000	-0.97193300	-3.11993900
H	1.80718300	-2.73697900	-3.00270600
Cl	-5.22566000	-0.54365000	-0.82181700

$TS_F{}^{Cp*}{}_{II\text{-}III}$

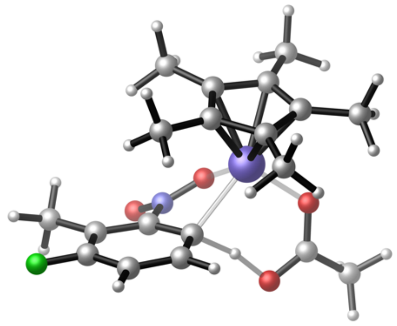

G = -1302.610527

E = -1302.95337695

O	0.24755900	2.86976100	-1.06791500
C	1.43679900	2.83392400	-0.63822900
O	1.97296500	1.79668800	-0.14688800
C	-1.97719500	0.66327800	0.31632700
C	-1.09877400	0.66004500	-0.77781900
C	-3.29544600	0.19816500	0.30860100
C	-1.61225100	0.19154500	-1.98953500
C	-3.71002800	-0.27355600	-0.94172400
C	-2.91779700	-0.27440900	-2.07935100
H	-0.29064900	1.70425300	-0.90676800
H	-0.98561300	0.20228100	-2.87559400
H	-3.33203800	-0.64670400	-3.00976000
Rh	0.89275600	-0.01795800	0.06871200
C	0.88116400	-1.93147700	1.05938600
C	2.22184700	-1.36990000	1.06373300
C	2.63572400	-1.22981800	-0.29764800
C	0.47394900	-2.11779800	-0.28985800
C	1.54319900	-1.64017300	-1.14418200
C	2.25331900	4.09368200	-0.67222900
H	1.89046100	4.76961600	-1.44686100
H	2.14705100	4.58930800	0.29857900
H	3.30846100	3.86342500	-0.82468700
O	-0.17792900	1.15111500	1.69468500
O	-2.12789700	1.75484800	2.36074000
C	-4.23189800	0.14432400	1.48217000
H	-3.71343200	-0.14391800	2.39824200
H	-5.02658000	-0.57595700	1.28805500
H	-4.69078800	1.12175500	1.65402800
N	-1.41638400	1.21917200	1.54253900
C	0.06640700	-2.25608900	2.26253100
C	3.02202600	-1.05632500	2.28108500
C	-0.80843600	-2.74417000	-0.72106900
C	1.55834500	-1.67119800	-2.63329500
C	3.94389200	-0.69918800	-0.76809600
H	-1.65908400	-2.31562800	-0.18478800
H	-0.97772700	-2.61949000	-1.79026500
H	-0.77095000	-3.81578100	-0.49935500
H	-1.00023400	-2.12984500	2.06340500
H	0.23696200	-3.30345300	2.53517300
H	0.34532400	-1.63479400	3.11551100
H	2.38511300	-0.65959900	3.07487500
H	3.49601400	-1.97112800	2.65277800
H	3.80419500	-0.32662700	2.06695400
H	4.34278700	0.04608300	-0.07857100
H	4.65943400	-1.52652500	-0.82938500
H	3.85676500	-0.24870800	-1.75764200
H	0.54582500	-1.68718700	-3.03867400
H	2.08199500	-0.80493900	-3.04338100
H	2.07757300	-2.57455600	-2.97129900
F	-4.95743000	-0.74915800	-1.04779200

$TS_H{}^{Cp}{}_{II\text{-}III}$

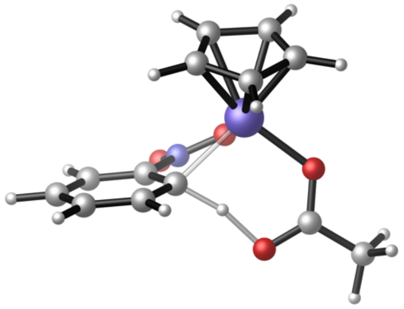

G = -967.661756

E = -967.855493659

O	0.41154000	2.43433600	-1.04121500
C	1.56096600	2.29092100	-0.55931800
O	1.97291900	1.20819200	-0.02230600
C	-2.00528800	0.43314100	0.41940700
C	-1.15268000	0.35289800	-0.69062300
C	-3.36443100	0.17061600	0.38693200
C	-1.74243100	-0.03476000	-1.89795200
C	-3.90995700	-0.22301800	-0.83267200
C	-3.10449000	-0.32666000	-1.96615300
H	-0.26570500	1.25241600	-0.83825600
H	-1.13178400	-0.08043300	-2.79390500
H	-4.96895800	-0.44710800	-0.89569900
H	-3.54484100	-0.62805800	-2.91067900
Rh	0.77492000	-0.48122300	0.11809400
C	0.50459900	-2.52820800	0.80134900
H	-0.22107300	-2.80518800	1.55385000
C	1.85982900	-2.09034300	1.05259600
H	2.32815600	-1.99625100	2.02287400
C	2.46014200	-1.79579800	-0.19697400
H	3.45265500	-1.39069400	-0.34195700
C	0.28044700	-2.48149300	-0.59035700
H	-0.65214400	-2.70291500	-1.09114600
C	1.47469200	-1.97427000	-1.21964900
H	1.61646400	-1.80522900	-2.27808900
C	2.54616100	3.42415300	-0.61396800
H	3.34461200	3.16297900	-1.31450500
H	2.06084000	4.34290600	-0.94122300
H	2.99941400	3.56382000	0.36996600
O	-0.15492100	0.62341500	1.79322200
O	-2.05588000	1.24330500	2.58598700
N	-1.39338600	0.79462300	1.68301800
H	-3.96994500	0.24936400	1.28221600

$TS_H{}^{Cp^*}{}_{II\text{-}III}$

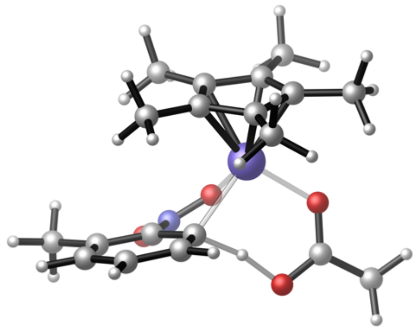

G = -1203.396521

E = -1203.74990515

O	0.02107900	2.95408800	0.77691500
C	-1.15631000	2.92143900	0.31614000
O	-1.71597400	1.86328900	-0.09871800
C	2.21396100	0.47628100	-0.16982700
C	1.25176200	0.66422900	0.83592800
C	3.49266000	-0.05823900	0.03166700
C	1.62575500	0.33490100	2.13834300
C	3.78978900	-0.39865200	1.35863100
C	2.88869900	-0.19201300	2.39715600
H	0.49792600	1.75620000	0.76883100
H	0.92525900	0.50149500	2.95093300
H	4.76198200	-0.83258400	1.57125500
H	3.17369400	-0.45222000	3.41168600
Rh	-0.71096300	0.00105000	-0.10006600
C	-0.72888800	-2.00908800	-0.88167700
C	-2.03026600	-1.38498900	-1.05797200
C	-2.54402700	-1.08280500	0.24201500
C	-0.43978400	-2.06574200	0.50845700
C	-1.54360200	-1.44263300	1.21439600
C	-1.92985300	4.20519800	0.22395100
H	-1.55930400	4.93149800	0.94780700
H	-1.79217900	4.61521100	-0.78206500
H	-2.99419900	4.02020200	0.37439500
O	0.55780500	0.92586400	-1.73203800
O	2.59569500	1.22740000	-2.33385700
C	4.51326400	-0.30729500	-1.04393400
H	4.07299300	-0.77928600	-1.92607800
H	5.29894800	-0.96007700	-0.65809400
H	4.97172800	0.62892100	-1.37305700
N	1.78752800	0.89336900	-1.49724000
C	0.15708800	-2.50312300	-1.97147700
C	-2.70998300	-1.16984400	-2.36656200
C	0.76469200	-2.70775700	1.10763100
C	-1.68305100	-1.31020000	2.69157800
C	-3.84863900	-0.43882900	0.55353100
H	1.68052400	-2.37651100	0.61142700
H	0.85029500	-2.49204600	2.17215300
H	0.68439500	-3.79236100	0.98061400
H	1.20987000	-2.42244200	-1.69146400
H	-0.06185300	-3.56026900	-2.15874700
H	-0.00466800	-1.95490600	-2.90117100
H	-1.99393000	-0.87180800	-3.13536600
H	-3.18609400	-2.10180800	-2.68958700

H	-3.47830300	-0.39849700	-2.29553900
H	-4.26484300	0.06632800	-0.31822200
H	-4.55568900	-1.20984200	0.87797600
H	-3.74690000	0.28861100	1.36134500
H	-0.70888500	-1.24326600	3.17813600
H	-2.26332200	-0.42307900	2.95373000
H	-2.20524400	-2.18844600	3.08651700

$TS_{Me}{}^{Cp*}{}_{II-III}$

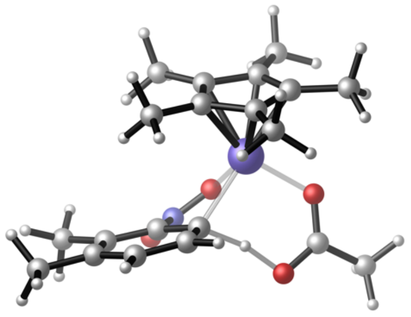

G = -1242.679363

E = -1243.05917303

O	0.26332300	2.85554500	-1.09089400
C	1.45468100	2.82084200	-0.66642100
O	1.99030600	1.78732000	-0.16788100
C	-1.95713600	0.67371300	0.32903900
C	-1.08146800	0.64864400	-0.76908000
C	-3.27906800	0.21795500	0.33085600
C	-1.61581400	0.18240000	-1.96795700
C	-3.76631700	-0.25590800	-0.90902900
C	-2.93653800	-0.24855500	-2.03258100
H	-0.27181600	1.69169700	-0.91799400
H	-0.99965200	0.17422900	-2.86201700
H	-3.33922300	-0.59886700	-2.97857900
Rh	0.90301400	-0.02370500	0.06618400
C	0.89981300	-1.93109100	1.06751200
C	2.24242000	-1.37637700	1.05088000
C	2.63760800	-1.24461700	-0.31679400
C	0.47257000	-2.12210600	-0.27529600
C	1.53229300	-1.65458600	-1.14675000
C	2.27330300	4.07889900	-0.71693200
H	1.91049900	4.74558700	-1.49959500
H	2.16877800	4.58673800	0.24767900
H	3.32790200	3.84525100	-0.86796000
O	-0.13860900	1.15832300	1.68656700
O	-2.06532400	1.84795200	2.33220000
C	-4.15763300	0.18807400	1.55256600
H	-3.58740600	0.02867300	2.46866300
H	-4.89838600	-0.60839900	1.47189000
H	-4.69499400	1.13516600	1.66492500
N	-1.37717900	1.25494600	1.53261800
C	0.09894100	-2.24496900	2.28299000
C	3.06339500	-1.06337000	2.25488900
C	-0.81793600	-2.74807100	-0.68340300
C	1.52755600	-1.69656400	-2.63597500
C	3.94123400	-0.72001900	-0.80751500
H	-1.65938500	-2.31260400	-0.13855900
H	-1.00287400	-2.63035800	-1.75084300
H	-0.78021500	-3.81823800	-0.45457500
H	-0.96952300	-2.11552200	2.09611100
H	0.26900800	-3.29101400	2.56097500
H	0.39027700	-1.61882800	3.12820000
H	2.44188600	-0.66183100	3.05834500
H	3.54054200	-1.97870900	2.62116600
H	3.84459900	-0.33715300	2.02582600
H	4.34853500	0.03177400	-0.12998800
H	4.65580300	-1.54817900	-0.86888100
H	3.84368600	-0.27870400	-1.80028900
H	0.50934300	-1.71433700	-3.02649900
H	2.04482300	-0.83322100	-3.05996800
H	2.04171800	-2.60228900	-2.97528500
C	-5.17915600	-0.75846900	-1.03809600
H	-5.90075900	-0.02524300	-0.66486400
H	-5.41602100	-0.96875100	-2.08302600
H	-5.32659300	-1.68285100	-0.46885800

$TS_{NO2}{}^{Cp*}{}_{II-III}$

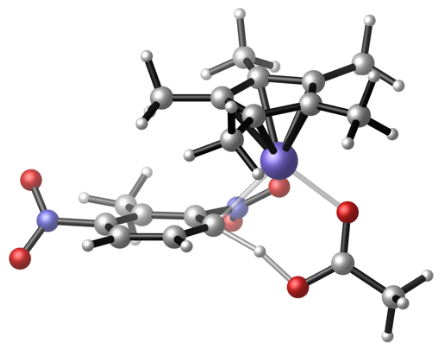

G = -1407.816528

E = -1408.16943772

O	0.84598700	2.87563500	-1.03736500
C	2.05799300	2.68471300	-0.72952500
O	2.49742300	1.59183000	-0.26076200
C	-1.54548600	0.94150700	0.50497200
C	-0.75983000	0.86821200	-0.65159500
C	-2.89404400	0.57128100	0.61178000
C	-1.39565600	0.46524900	-1.82555400
C	-3.42767400	0.12134500	-0.60335700
C	-2.73383800	0.10219600	-1.80764400
H	0.17986800	1.78808000	-0.82613600
H	-0.84177200	0.43677000	-2.75771900
H	-3.23955700	-0.22380300	-2.70970000
Rh	1.21180700	-0.06556800	0.05758900
C	1.14964700	-1.94260700	1.11738600
C	2.51999500	-1.53892300	0.86855300
C	2.70390400	-1.48566700	-0.55291200
C	0.49106800	-2.10622500	-0.13408100
C	1.44632400	-1.79343700	-1.17905700
C	3.04566400	3.79130900	-0.96137700
H	2.54361100	4.75854400	-0.98413500
H	3.81699100	3.77602100	-0.19014800
H	3.52797800	3.62377400	-1.93008600
O	0.39585200	1.20863900	1.74295500
O	-1.40373700	2.06911400	2.53559700
C	-3.69695700	0.67299800	1.87935800
H	-3.14934900	0.25324100	2.72599500
H	-4.64426400	0.14728800	1.79498600
H	-3.89647000	1.72296400	2.10417000
N	-0.83043300	1.43795500	1.67951000
C	0.52910200	-2.11842100	2.45793000
C	3.56662500	-1.30404800	1.90165900
C	-0.91071500	-2.58271500	-0.30914800
C	1.20803300	-1.86121600	-2.64805000
C	3.96721800	-1.11686200	-1.24649700
H	-1.59089000	-2.08866100	0.38838000
H	-1.27215700	-2.41582300	-1.32300300
H	-0.94703200	-3.65803500	-0.10609600
H	-0.55210900	-1.97226200	2.41563100
H	0.72163600	-3.13833700	2.80844500
H	0.95243600	-1.42453400	3.18688800
H	3.13605000	-0.88426900	2.81300000
H	4.04268700	-2.25710400	2.15666000
H	4.33749000	-0.62407400	1.53464800
H	4.44119000	-0.26030200	-0.76241700
H	4.66114500	-1.96276300	-1.19869200
H	3.79017600	-0.87549800	-2.29516500
H	0.14383200	-1.84252900	-2.88407500
H	1.69541600	-1.03145200	-3.16513200
H	1.63033400	-2.79456200	-3.03580800
N	-4.80358200	-0.38978900	-0.67086300
O	-5.11939300	-1.27863500	0.10530700
O	-5.52971400	0.07699200	-1.53192900

$TS_{OMe}{}^{Cp*}{}_{II\text{-}III}$

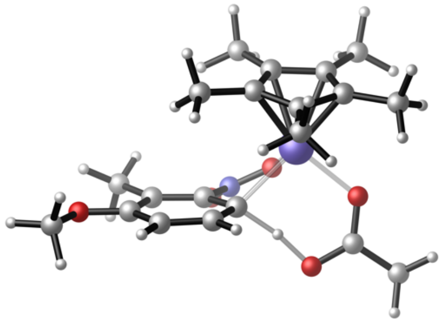

G = -1317.860542

E = -1318.24211288

O	0.53457200	2.89076600	-1.12212200
C	1.76715800	2.73134500	-0.87887600
O	2.25959700	1.65539000	-0.43203300
C	-1.66177400	0.84655600	0.55272000
C	-0.89226300	0.77649400	-0.62476400
C	-2.99012500	0.46173500	0.69011800
C	-1.57050200	0.33531800	-1.76111400
C	-3.61121600	0.02080000	-0.50749300
C	-2.91254900	-0.02531400	-1.71687900
H	-0.06938200	1.79953200	-0.87014000
H	-1.04328800	0.28889400	-2.70945400
H	-3.40873000	-0.34857100	-2.62386500
Rh	1.08213200	-0.04601500	0.04617100
C	1.04326900	-1.90998500	1.12245800
C	2.41749900	-1.47541100	0.93907900
C	2.66737500	-1.42229900	-0.46601500
C	0.45768200	-2.11713500	-0.15709100
C	1.44543400	-1.75754800	-1.15516400
C	2.69852700	3.88879900	-1.09840800
H	2.28308600	4.59026300	-1.82227200
H	2.82611700	4.40720000	-0.14236900
H	3.67650900	3.53227200	-1.42489900
O	0.29831300	1.22558000	1.73878500
O	-1.52375100	2.00319600	2.56605600
C	-3.77649400	0.44693800	1.97094700
H	-3.14214000	0.25534000	2.83715700
H	-4.54557100	-0.32403600	1.91901900

H	-4.27559600	1.40751800	2.13115100
N	-0.94200300	1.38672700	1.70332700
C	0.36129600	-2.10209000	2.43254800
C	3.39769700	-1.20796900	2.03004100
C	-0.91206900	-2.65279000	-0.39900300
C	1.28288000	-1.85170200	-2.63378300
C	3.94611600	-1.03845900	-1.12322700
H	-1.65106300	-2.15697500	0.23536800
H	-1.21507300	-2.53075500	-1.43818000
H	-0.92245900	-3.72116300	-0.15929400
H	-0.72035800	-1.98985000	2.33434600
H	0.56615200	-3.11346000	2.80032300
H	0.72363400	-1.39200900	3.17860200
H	2.90344700	-0.79763400	2.91294400
H	3.88751000	-2.14414900	2.31894100
H	4.16709800	-0.50412800	1.70801700
H	4.55809000	-0.40962700	-0.47561100
H	4.51022900	-1.94957800	-1.35165400
H	3.76854000	-0.50803200	-2.06033500
H	0.23148000	-1.81473600	-2.92212800
H	1.80816700	-1.03959800	-3.14136100
H	1.70355200	-2.79942000	-2.98700400
O	-4.89895000	-0.33223800	-0.37858900
C	-5.60355100	-0.80350000	-1.52103500
H	-6.60990500	-1.03324300	-1.17232200
H	-5.65509900	-0.03426900	-2.29828900
H	-5.14143500	-1.71044500	-1.92480800

TS$_{S12\text{-}S13}$

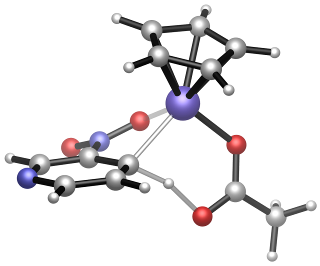

G = -983.692086

E = -983.875003644

O	0.44085100	2.41043800	-1.05295600
C	1.57711100	2.28586300	-0.53723300
O	1.98584100	1.21125500	0.02251700
C	-2.01406200	0.42564300	0.40322800
C	-1.17706400	0.37164500	-0.71392500
C	-3.37067100	0.13699100	0.34121000
C	-3.16726900	-0.27944600	-1.89982700
H	-0.24013300	1.22064100	-0.85242800

H	-3.66576500	-0.56412100	-2.82225600
Rh	0.78916400	-0.47665800	0.11393200
C	0.50248200	-2.52696300	0.77789900
H	-0.22476100	-2.80244000	1.52951200
C	1.85664100	-2.08662300	1.03131100
H	2.32540000	-1.99686500	2.00194900
C	2.46137000	-1.79243600	-0.22079200
H	3.45517700	-1.38955300	-0.36362300
C	0.27887300	-2.46927000	-0.61202800
H	-0.65542900	-2.68156700	-1.11407100
C	1.47720200	-1.96611500	-1.24104200
H	1.61568900	-1.78678500	-2.29836500
C	2.54718700	3.43246400	-0.55679500
H	2.11200300	4.29980700	-1.05161600
H	2.81715100	3.68615300	0.47178400
H	3.45974500	3.12432900	-1.07341700
O	-0.18078100	0.59289000	1.79493400
O	-2.09571500	1.18371100	2.58100100
N	-1.41825700	0.76057300	1.67730400
H	-3.99481300	0.17506200	1.22910000
N	-3.93894900	-0.22137300	-0.80854900
C	-1.80114200	0.00735100	-1.90469700
H	-1.24171300	-0.03041700	-2.83262000

TS$_{S14\text{-}S15}$

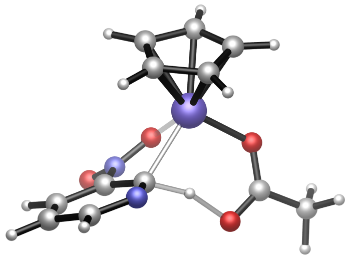

G = -983.691792

E = -983.875122813

O	0.30813500	2.36084200	-1.11639800
C	1.44628700	2.32304600	-0.59073800
O	1.91095900	1.29645600	0.01261500
C	-2.04475900	0.36326000	0.40794000
C	-1.19635900	0.24786700	-0.69791100
C	-3.40208000	0.08465900	0.34539000
C	-3.87640200	-0.35631700	-0.87926500
C	-2.98018700	-0.48756600	-1.94403900
H	-0.28675400	1.13534000	-0.86679500

H	-4.92246700	-0.60339100	-1.01407900
H	-3.33475000	-0.84990500	-2.90473300
Rh	0.80381100	-0.45035000	0.13631000
C	0.68145900	-2.50538200	0.86385800
H	0.01031900	-2.80458800	1.65780000
C	2.01620700	-1.97913400	1.04031800
H	2.52617400	-1.84336700	1.98428000
C	2.53696700	-1.67776500	-0.24546500
H	3.49560000	-1.21940700	-0.44867800
C	0.38095600	-2.48912300	-0.51249900
H	-0.56049600	-2.77041000	-0.96408800
C	1.51422500	-1.93071400	-1.21085800
H	1.58686800	-1.76248000	-2.27655000
C	2.35572800	3.51511200	-0.67299500
H	1.79850900	4.40659100	-0.95925800
H	2.85658500	3.66673900	0.28491600
H	3.12346900	3.31340700	-1.42632100
O	-0.21468900	0.64817700	1.78430600
O	-2.13876400	1.26341600	2.52763500
N	-1.45326900	0.78882700	1.65564000
N	-1.68087600	-0.18753400	-1.87016100
H	-4.04290700	0.18938700	1.21354200

TS$_{S17\text{-}S18}$

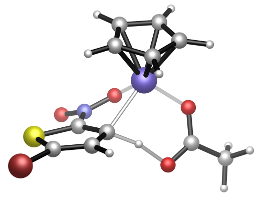

G = -1301.026498

E = -1301.17178878

O	1.28496300	2.25085600	-1.40112100
C	2.49479000	1.96508500	-1.23506800
O	2.90342500	0.89995700	-0.65638800
C	-0.75221800	0.80713300	1.05225900
C	-0.39140100	0.58738700	-0.25880400
C	-2.67980700	0.20680500	-0.25897700
C	-1.53945700	0.22924600	-1.01818200
H	0.51641700	1.26267100	-0.82195000
H	-1.51972600	0.02467900	-2.08133700

Rh	1.59654200	-0.55019900	0.03776400
C	1.51973100	-2.44252800	1.11264700
H	1.29898000	-2.49118000	2.17115200
C	2.82638400	-2.22568400	0.53930500
H	3.75509000	-2.13592100	1.08595700
C	2.67287000	-2.17014500	-0.87587200
H	3.45835400	-1.97551100	-1.59363000
C	0.57309600	-2.47371100	0.06820900
H	-0.49776500	-2.57361500	0.18064100
C	1.28165700	-2.27283600	-1.17315100
H	0.83710800	-2.21356800	-2.15732800
C	3.55969800	2.89073300	-1.75006700
H	3.12052600	3.82019200	-2.11048100
H	4.28388300	3.09503600	-0.95828100
H	4.09051100	2.39472700	-2.56802400
O	1.39235600	0.85704200	1.80861300
O	-0.17020700	1.59905200	3.11363800
N	0.19000000	1.11411500	2.06427300
S	-2.42662700	0.62730500	1.40099800
Br	-4.41051700	-0.22138300	-0.85267700

TS$_{S20\text{-}S21}$

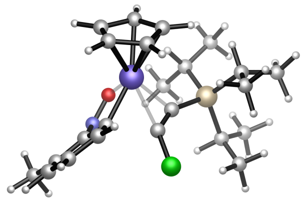

G = -1959.058105

E = -1959.50583823

C	-2.64799500	-1.06055900	-0.38678900
C	-2.21604400	-0.09007900	0.53619800
C	-3.89507700	-1.70954600	-0.32189100
C	-3.09759300	0.28142200	1.55276600
C	-4.73451700	-1.29182100	0.70983200
C	-4.35241500	-0.30561200	1.61928900
H	-2.79651600	1.01964800	2.28744800
H	-5.71627900	-1.74549200	0.79592600
H	-5.04702700	0.00245900	2.39382800
Rh	-0.75524000	1.19977200	-0.23153400
C	-1.45866000	2.95565700	-1.39026400
H	-2.12292200	2.85714000	-2.23821200
C	-0.00530900	2.88831300	-1.44382900
H	0.58153900	2.74439600	-2.34086500
C	0.48746900	3.06307700	-0.13978900
H	1.52334700	3.06066200	0.16586500

C	-1.84036900	3.16348900	-0.05593700
H	-2.85200900	3.22124000	0.32058100
C	-0.64598500	3.13808200	0.74388300
H	-0.59978400	3.25255700	1.81862100
O	-0.93940500	-0.36451000	-1.74305800
O	-1.74816900	-2.32032900	-2.11396900
C	-4.36479100	-2.75305100	-1.29546600
H	-4.24429000	-2.42554700	-2.33169300
H	-5.42065800	-2.96529400	-1.11853400
H	-3.79854500	-3.68089500	-1.18140400
N	-1.74834200	-1.29246200	-1.47376500
Si	2.42507900	-0.45141800	-0.04381500
C	2.87828200	-2.23611000	0.41084800
C	2.50680700	-0.13547000	-1.91217000
C	3.35649800	0.89921400	0.91975100
H	2.10217800	-2.84368900	-0.07759900
H	1.80143900	0.69224600	-2.07368000
H	3.28967000	1.79349600	0.28294000
C	2.81156800	-2.51005000	1.91946100
C	4.23856400	-2.68163100	-0.15154500
C	2.72399400	1.23326300	2.27765700
C	4.84993100	0.57481500	1.08565300
C	2.02100300	-1.33198900	-2.74335800
C	3.88834100	0.33773500	-2.38858900
H	3.28233000	2.03604700	2.77471500
H	1.68520100	1.56423200	2.17830300
H	2.73154100	0.36711300	2.94931400
H	4.99897800	-0.28301000	1.74988300
H	5.33996300	0.35060300	0.13303700
H	5.37464800	1.42761000	1.53305400
H	4.38691600	-3.75363800	0.02621000
H	4.32766300	-2.51298400	-1.22852400
H	5.06582700	-2.15611000	0.33595200
H	3.00081500	-3.57074000	2.12485500
H	3.56754400	-1.93325000	2.46355800
H	1.83527600	-2.26368800	2.34609300
H	4.65685300	-0.42796500	-2.23297900
H	3.86544700	0.55839900	-3.46289300
H	4.21329300	1.24746700	-1.87315100
H	1.04171500	-1.69485800	-2.41890400
H	1.93017900	-1.05335700	-3.80035500
H	2.72692100	-2.16760400	-2.68992400
C	0.61619600	-0.24726800	0.45417400
C	-0.39034800	-0.45832300	1.21253900
Cl	-0.66675800	-1.07073300	2.78347000

TS$_{S22\text{-}VII}$

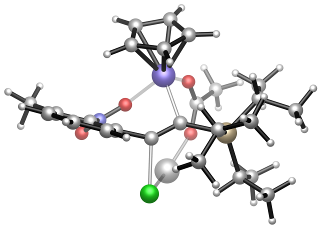

G = -2333.284493

E = -2333.77770912

C	1.89878700	1.51292200	0.11892800
C	4.47476000	2.28983000	0.88817100
C	0.51591400	1.13164100	-0.21036700
C	-0.45659300	0.41784500	0.26075200
C	3.04039700	0.81309900	-0.29570300
C	4.34603100	1.17218600	0.06082100
C	2.08492800	2.63017400	0.93286700
C	3.36534700	3.00970200	1.31489800
H	5.46877800	2.58896200	1.20404600
H	3.49750200	3.87284400	1.95825700
H	1.22105500	3.19598400	1.26190400
C	-0.09404900	-1.54867100	3.08819600
C	0.65201300	-0.32150300	3.10667200
C	0.83818600	-2.62675700	2.83624900
H	0.25444800	0.66635900	3.29204900
H	0.57410900	-3.67042100	2.73322400
C	1.98242800	-0.62907600	2.73471600
H	2.78313900	0.08593000	2.59498900
C	2.09972900	-2.06912800	2.60549500
H	2.99313600	-2.60510300	2.31466400
H	-1.14401000	-1.66302300	3.31782300
Si	-2.32834100	0.78917800	0.54363700
C	-3.26991000	-0.60436100	1.44345900
H	-2.90124900	-0.55355900	2.47865100
C	-2.12458800	2.23878800	1.78437300
H	-1.29913500	1.89547500	2.42512200
C	-3.16084900	1.27823300	-1.09363700
H	-2.40584700	1.86479700	-1.62888500
O	-0.22439100	-2.69013200	-0.07571700
C	-0.65313200	-2.91650700	-1.25250600
O	-0.67093100	-2.13777300	-2.23038000
Ag	-0.15288700	-0.02659700	-2.64031100
C	-1.22252400	-4.30707400	-1.44539200
H	-1.42037400	-4.50546300	-2.49907700
H	-0.53434400	-5.05548500	-1.04527100
H	-2.15876300	-4.38174200	-0.88222400
Rh	0.63440100	-1.19564000	1.14238900

O	2.00518200	-1.21950200	-0.71962600
O	3.52726100	-0.55758100	-2.07956200
N	2.85312800	-0.38757100	-1.08797700
C	-4.78119800	-0.29754400	1.48465300
C	-3.08747400	-2.04398300	0.95137000
C	-3.52460700	0.06272100	-1.96232200
C	-4.39198000	2.18342700	-0.91623600
C	-3.32087300	2.49495400	2.71223700
C	-1.68001700	3.55836500	1.13915900
H	-5.27419600	-0.96358600	2.20289100
H	-5.01891900	0.72840500	1.77332200
H	-5.24141500	-0.48088700	0.50835100
H	-3.59520000	1.61102900	3.29582900
H	-3.07497400	3.29268100	3.42413100
H	-4.20546100	2.82163700	2.15442600
H	-2.49961800	4.02613000	0.58382500
H	-1.36521100	4.27380100	1.90880100
H	-0.85125100	3.43388400	0.43933400
H	-3.36202600	-2.14639600	-0.10439400
H	-2.06425500	-2.39994600	1.05847400
H	-3.74169100	-2.71569100	1.52216800
H	-5.21100700	1.66671700	-0.40726700
H	-4.16798600	3.09143700	-0.34964900
H	-4.76864100	2.49772100	-1.89761800
H	-3.80859800	0.38315400	-2.97241600
H	-2.70675000	-0.66065400	-2.06061500
H	-4.37893800	-0.47848400	-1.54192900
C	5.56727500	0.40153500	-0.36244200
H	5.43148400	-0.67831600	-0.25378600
H	5.81014000	0.60034300	-1.40926500
H	6.41907400	0.70197100	0.25022900
Cl	0.13673700	2.48428700	-1.86248700

$TS_{S24\text{-}25}$

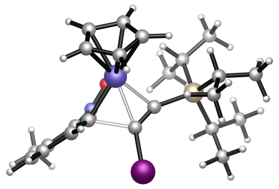

G = -1510.282840

E = -1510.72717282

C	-2.51225000	-0.33671700	1.25511600
C	-2.12703500	-0.09006300	-0.07811000
C	-3.78661500	-0.04141200	1.77745700
C	-3.10329200	0.40417700	-0.95073500
C	-4.71166600	0.44819500	0.85934300
C	-4.38502100	0.64294100	-0.48468400
H	-2.84680000	0.60994300	-1.98404600
H	-5.71734300	0.66794500	1.20226600
H	-5.14486900	1.00088500	-1.17168900
Rh	-0.56738700	-1.33071500	-0.73416800
C	-1.07345300	-3.33468900	-1.53684600
H	-1.64277800	-4.05562500	-0.96571100
C	0.37481300	-3.19029300	-1.50588600
H	1.05225200	-3.79460800	-0.91845200
C	0.72717700	-2.15376500	-2.38366500
H	1.72338000	-1.79230700	-2.58926600
C	-1.59334500	-2.38077600	-2.42461200
H	-2.63826600	-2.20790500	-2.64075800
C	-0.49273500	-1.57536700	-2.88107700
H	-0.55993100	-0.73935200	-3.56465100
O	-0.67014000	-1.67945000	1.42085400
O	-1.50106400	-0.92984000	3.25614600
C	-4.19230200	-0.26962000	3.20632000
H	-3.99123000	-1.29659100	3.52412900
H	-5.26053600	-0.07457900	3.31804900
H	-3.64322200	0.39013400	3.88232700
N	-1.52825400	-1.00002800	2.04606600
Si	2.48603000	0.04987500	0.46594600
C	2.92246600	1.47501900	1.64219600
C	2.69821100	-1.64196500	1.30402600
C	3.36585400	0.10363700	-1.21993400
H	2.17644700	1.40487000	2.44756100
H	2.00923800	-2.30338800	0.76076100
H	3.32601400	-0.93210100	-1.58521100
C	2.79376200	2.86147500	0.99837800
C	4.31390000	1.32104600	2.28063500
C	2.67173900	1.00412700	-2.25158000
C	4.85274400	0.47600400	-1.10245500
C	2.26374800	-1.63320300	2.77696700
C	4.10974600	-2.22806700	1.14902900
H	3.19391900	0.95682000	-3.21495400
H	1.62976700	0.71514800	-2.42420600
H	2.67326500	2.05175700	-1.93005200
H	4.98003200	1.52159500	-0.80308500
H	5.38866000	-0.14652600	-0.37952500
H	5.34980900	0.35421800	-2.07252400
H	4.47072900	2.10540000	3.03121700
H	4.44998700	0.35896700	2.78191700
H	5.10942500	1.42206100	1.53573300
H	2.96767600	3.64781300	1.74326000

H	3.53255000	3.00014800	0.20115100
H	1.80332200	3.02952900	0.56777300
H	4.86966000	-1.59985100	1.62655200
H	4.16559800	-3.21667900	1.62089500
H	4.39073000	-2.35061700	0.09779600
H	1.25593000	-1.23083400	2.90941900
H	2.26194300	-2.65272200	3.18148000
H	2.94508900	-1.04079800	3.39627200
C	0.64527200	0.19908400	0.05557700
C	-0.44290400	0.83363400	-0.19053500
I	-0.85656500	2.79047700	-0.72789000

TS$_{S26\text{-}VII}$

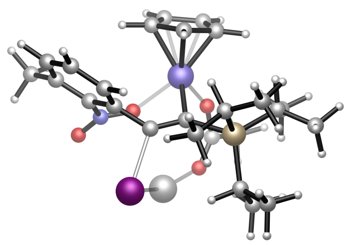

G = -1884.514675

E = -1885.00369202

C	1.92362300	-0.66360000	-1.10377900
C	4.51109100	-0.51413500	-2.16794100
C	0.52682100	-0.74169300	-0.61040500
C	-0.45700000	0.12126500	-0.51314700
C	3.04020700	-0.37304600	-0.31018800
C	4.34957000	-0.29513800	-0.79828500
C	2.14345400	-0.89467900	-2.46142100
C	3.42744500	-0.81272500	-2.98613200
H	5.50660700	-0.44119900	-2.59323300
H	3.58369400	-0.97948000	-4.04651700
H	1.30180200	-1.13707500	-3.10014600
C	-0.20949000	3.40863200	-1.44875000
C	0.64502200	2.53010700	-2.19403900
C	0.62269500	4.15414800	-0.52285600
H	0.34860100	1.86311300	-2.99023900
H	0.25799700	4.87401600	0.19731200
C	1.93962800	2.63247400	-1.62851200
H	2.80436500	2.05227000	-1.92568500
C	1.92945300	3.67942900	-0.62403400
H	2.77270900	3.97785900	-0.01640700
H	-1.26250300	3.57854700	-1.62029800
Si	-2.33329400	-0.00765500	-0.96092500
C	-3.30310000	1.62842700	-0.77558200
H	-2.94841500	2.24631900	-1.61380100
C	-2.13619300	-0.33544300	-2.84453400
H	-1.33432400	0.35762500	-3.13935200
C	-3.17944200	-1.43446500	-0.02989000
H	-2.43697700	-2.23921700	-0.01540100
O	-0.33559900	2.25155600	1.78350600
C	-0.75099800	1.71826700	2.85656800
O	-0.75801000	0.50351600	3.16507400
Ag	-0.16646900	-1.34410700	2.14169200
C	-1.31139600	2.70198700	3.86407700
H	-2.20677800	3.16897100	3.44142800
H	-1.56797800	2.20570800	4.80019900
H	-0.58353700	3.49579500	4.05118200
Rh	0.54053600	1.94135200	-0.13166500
O	1.91993600	0.77149300	1.35782800
O	3.47454900	-0.59190400	1.94219700
N	2.80627500	-0.05600500	1.08522900
C	-4.81366100	1.40263200	-0.99383800
C	-3.13155400	2.43918500	0.51368600
C	-3.51207400	-1.07836200	1.42728900
C	-4.42443900	-2.00091100	-0.73352200
C	-3.35795300	0.01568900	-3.70644900
C	-1.65274300	-1.75171300	-3.18115800
H	-5.31336700	2.36939700	-1.13091400
H	-5.05420800	0.78615700	-1.86216900
H	-5.26645600	0.93042300	-0.11609100
H	-3.65876400	1.06169800	-3.59752000
H	-3.12765300	-0.14844100	-4.76660600
H	-4.22178700	-0.61313100	-3.46432500
H	-2.39764000	-2.50931500	-2.91654200
H	-1.46275300	-1.84613300	-4.25762100
H	-0.72750600	-2.01110300	-2.66203200
H	-3.38101300	1.84242700	1.39834500
H	-2.11809600	2.81157400	0.64966600
H	-3.81218400	3.30053700	0.50385600
H	-5.25036500	-1.28462700	-0.74361700
H	-4.22588600	-2.29209900	-1.76893900
H	-4.77697100	-2.89468300	-0.20404600
H	-3.80754700	-1.97460600	1.98684400
H	-2.67235300	-0.61512700	1.95843900
H	-4.34664500	-0.37117800	1.48059400
C	5.53437900	0.05138700	0.06174600
H	5.32703200	0.89668400	0.72469800
H	5.82477700	-0.79638900	0.68779800
H	6.38287300	0.31528500	-0.57195400
I	0.29898700	-3.03156200	-0.04070600

TS$_{S28-S29}$

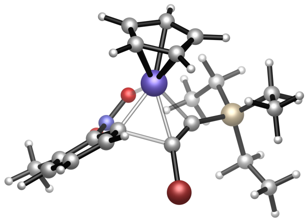

G = -1394.233609

E = -1394.59593581

C	2.35490700	-0.42212700	0.80390300
C	1.76539000	-0.02569300	-0.41048700
C	3.65274200	-0.95820600	0.90823700
C	2.53229600	-0.11411900	-1.57320000
C	4.37438000	-1.01318500	-0.28304700
C	3.83546900	-0.58193000	-1.49593800
H	2.10349100	0.17189200	-2.52764300
H	5.38855200	-1.39774400	-0.25658000
H	4.44195700	-0.62621700	-2.39469100
Rh	0.20948200	1.35401800	-0.17618800
C	0.81208600	3.48668100	-0.09073600
H	1.56479000	3.84775400	0.59685800
C	-0.60977800	3.37945400	0.19826000
H	-1.08089100	3.65158600	1.13318200
C	-1.25496400	2.87509900	-0.94274400
H	-2.31095000	2.67484500	-1.05132300
C	1.02582800	3.04045400	-1.40338400
H	1.97947800	2.95912400	-1.90573400
C	-0.23592900	2.56936700	-1.91151600
H	-0.40361700	2.14502900	-2.89249200
O	0.65442800	0.69612400	1.85620000
O	1.71694500	-0.77528400	3.00568400
C	4.28536700	-1.41936600	2.19049900
H	4.23048600	-0.65343100	2.96889000
H	5.33525600	-1.65774400	2.01127700
H	3.78479000	-2.31045000	2.57733700
N	1.55412500	-0.18035600	1.96349500
Si	-2.76286900	-0.42939600	0.74456300
C	-3.12856700	-2.25392900	1.04389400
C	-2.79692300	0.53336100	2.35825000
C	-3.90339500	0.33147600	-0.55037600
H	-2.25819300	-2.70633500	1.53548500
H	-2.32210500	1.50848400	2.18987700
H	-4.00302300	1.40498800	-0.34749300
C	-3.51755700	-3.06478900	-0.19775100
C	-3.48779700	0.10122100	-2.00895600
C	-2.14080900	-0.18146200	3.54586100
H	-4.21483400	0.53559100	-2.70312200
H	-2.51296200	0.55298500	-2.22271700
H	-3.40592400	-0.96489300	-2.24225500
H	-3.71214100	-4.11167000	0.05798000
H	-4.42375200	-2.66780700	-0.66686700
H	-2.72498600	-3.05612000	-0.95292800
H	-1.08300400	-0.38641400	3.35576300
H	-2.19756100	0.42655100	4.45501300
H	-2.63422000	-1.13524100	3.75966100
C	-1.02663400	-0.33291400	0.03119400
C	-0.04662400	-0.82901700	-0.61724000
Br	0.21772000	-2.29449200	-1.74774300
H	-3.94193700	-2.29519500	1.78144200
H	-3.85088700	0.74479400	2.58463900
H	-4.90130300	-0.09287600	-0.37584100

TS$_{S30-S31}$

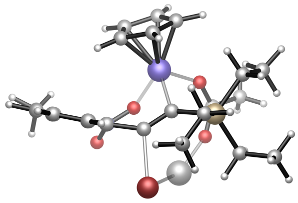

G = -1768.475827

E = -1768.87865481

C	-1.94470300	-0.91182000	0.82204200
C	-4.70531600	-0.88949800	1.26182600
C	-0.48485300	-0.85217400	0.67113800
C	0.48020600	0.00276400	0.76987700
C	-2.85205100	-0.55787300	-0.18550100
C	-4.23965300	-0.52994800	-0.00485300
C	-2.46249100	-1.28441900	2.06221300
C	-3.83458500	-1.27139700	2.27603100
H	-5.77384600	-0.86221300	1.44913600
H	-4.22853900	-1.54956700	3.24754600
H	-1.78095900	-1.57490000	2.85415500
C	0.08241700	3.55383600	1.29073600
C	-0.59895800	2.61934400	2.13644800
C	-0.84692800	4.02209300	0.29095500
H	-0.17430600	2.10629600	2.98835200
H	-0.62230400	4.72285900	-0.50060800
C	-1.89594800	2.41861100	1.58975000

H	-2.65453700	1.74910300	1.97219500
C	-2.04644800	3.31934400	0.46723000
H	-2.91452500	3.37250400	-0.17702600
H	1.10189200	3.89441500	1.40938400
Si	2.27108400	-0.09415300	1.41527400
Br	0.03151900	-3.06591500	0.27769300
C	3.09786900	1.58779800	1.63670300
H	2.41947600	2.24878700	2.18950100
C	2.11745300	-0.72904600	3.19051900
H	1.53902500	0.02116600	3.74764400
C	3.35829700	-1.18088700	0.32500400
H	2.90689100	-2.17360200	0.21875000
O	1.05507100	2.06833400	-1.36227400
C	1.59561000	1.46552000	-2.34212200
O	1.47571900	0.26472200	-2.67250900
Ag	0.82253100	-1.65914200	-1.84168300
C	2.51686000	2.31926300	-3.19037100
H	3.55230800	2.06803400	-2.93707800
H	2.36839500	2.09089700	-4.24799600
H	2.35784000	3.38242700	-3.00846500
Rh	-0.40068400	1.82906400	0.15577400
O	-1.41286600	0.68090600	-1.53782100
O	-2.78970500	-0.66295700	-2.48159700
N	-2.32402400	-0.16321800	-1.48182300
C	3.68587200	2.30636500	0.41978000
C	4.78282700	-1.31949900	0.88283100
C	1.53898400	-2.12062000	3.44472200
H	2.09869400	-2.89698500	2.91365100
H	1.56346300	-2.36535900	4.51238800
H	0.49754000	-2.18863300	3.11631000
H	4.32243900	1.63775400	-0.17135300
H	2.90071700	2.67881200	-0.24021600
H	4.30616600	3.15570500	0.72736600
H	5.27609300	-0.34693100	0.98414400
H	4.77991500	-1.79323800	1.87021000
H	5.40493000	-1.93685200	0.22653700
C	-5.20710200	-0.09966700	-1.07386200
H	-4.86220400	0.79206200	-1.60519200
H	-5.35242200	-0.89191500	-1.81282200
H	-6.17352000	0.12695500	-0.62075700
H	3.13357100	-0.67153900	3.60485200
H	3.90866700	1.36708700	2.34617700
H	3.39093000	-0.74018800	-0.68081100

TS$_{S33-V}$

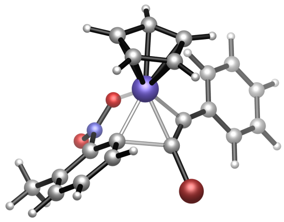

G = -1098.764149

E = -1099.02451641

C	2.07310900	-0.37721800	1.08642400
C	1.62542700	0.02724000	-0.18550200
C	3.38109500	-0.83092600	1.34762700
C	2.54342000	0.03185900	-1.23452600
C	4.25483600	-0.80280600	0.26226200
C	3.85248700	-0.36157900	-0.99802900
H	2.23068800	0.33252700	-2.22860000
H	5.27995500	-1.12394500	0.41394800
H	4.57370000	-0.33614300	-1.80847300
Rh	-0.06623400	1.24921100	-0.12902200
C	0.32487800	3.46307300	-0.03360200
H	0.98042900	3.91368600	0.69890700
C	-1.09369900	3.20056600	0.15123800
H	-1.65940700	3.43208500	1.04403100
C	-1.59796700	2.62441300	-1.02782300
H	-2.61394000	2.29914700	-1.20207000
C	0.68477000	3.01638800	-1.31110900
H	1.67649300	3.02818800	-1.74130300
C	-0.48082400	2.40911400	-1.90635600
H	-0.52749000	1.95939000	-2.88915100
O	0.15697700	0.56763200	1.92110000
O	1.20394900	-0.81020300	3.19168000
C	3.87344200	-1.29111100	2.69040100
H	3.68717300	-0.54425300	3.46720100
H	4.94732100	-1.47894800	2.63737500
H	3.37175100	-2.21089200	3.00064400
N	1.11733100	-0.22434700	2.13679500
C	-1.17572500	-0.48975700	-0.12631400
C	-0.09987400	-0.86905500	-0.70695500
Br	0.36665900	-2.17327500	-1.95901900
C	-2.52568100	-0.77587900	0.29620300
C	-3.29431800	0.18827700	0.96091100
C	-3.07229700	-2.04372700	0.03905700
C	-4.59682800	-0.10357000	1.34604000
H	-2.86103000	1.15928100	1.17481200
C	-4.37486600	-2.32731900	0.42869300
H	-2.47951300	-2.79872400	-0.46718900

C	-5.13921000	-1.35910900	1.07924000
H	-5.18740700	0.64793300	1.85982100
H	-4.79576400	-3.30634700	0.22465300
H	-6.15725600	-1.58511800	1.37995000
C	-4.70531600	-0.88949800	1.26182600

TS$_{VI-S34}$

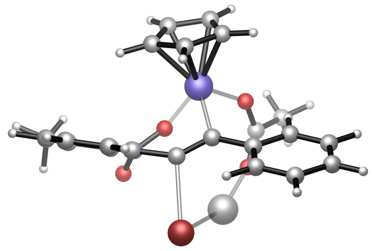

G = -1098.764149

E = -1473.31366354

C	1.80091300	-0.91813400	-0.90235500
C	4.56514000	-1.19301400	-1.15283200
C	0.36056100	-0.77042000	-0.80908700
C	-0.62616100	0.04434200	-0.93815100
C	2.66658700	-0.68851500	0.17786300
C	4.05515000	-0.81504600	0.09396700
C	2.35742700	-1.30642800	-2.12086800
C	3.73526400	-1.44084700	-2.23949100
H	5.64007700	-1.28967200	-1.26448300
H	4.16633500	-1.73525000	-3.18997300
H	1.70200500	-1.50619300	-2.96108700
C	-0.23441800	3.28341600	-1.69636300
C	0.75363200	2.41822300	-2.27836900
C	0.40910200	4.03047800	-0.63738100
H	0.59624500	1.73371500	-3.10053800
H	-0.08102000	4.74485500	0.01012500
C	1.93154400	2.53313800	-1.49998900
H	2.84340500	1.96886400	-1.64361800
C	1.72479800	3.56933600	-0.50863600
H	2.44011000	3.87739300	0.24153200
H	-1.24931200	3.41849000	-2.04463100
Br	-0.32805200	-3.12193800	-0.41721900
O	-1.11884800	2.13999800	1.27996800
C	-1.56191300	1.54762500	2.32177100
O	-1.55691000	0.32451600	2.56826900
Ag	-1.22082500	-1.55916700	1.49974300
C	-2.18691000	2.48043200	3.33752200
H	-3.05396900	2.97234500	2.88662400
H	-2.50109500	1.93499800	4.22749000
H	-1.47116400	3.25958300	3.61173500
Rh	0.29247300	1.83961600	-0.26170500
O	1.29462800	0.70741800	1.44499300
O	2.41331300	-0.81295800	2.45979000
N	2.08999100	-0.24814100	1.43945700
C	4.98534500	-0.53698100	1.24244700
H	4.71932300	0.38186400	1.77296000
H	4.96733600	-1.35550600	1.96650300
H	6.00541600	-0.43089700	0.86980600
C	-2.03257900	-0.07330600	-1.32103300
C	-3.03109200	0.73921900	-0.76759300
C	-2.37861100	-1.00785000	-2.31081900
C	-4.35453800	0.58779900	-1.16687600
H	-2.76290900	1.48441300	-0.02919200
C	-3.70128300	-1.14432400	-2.71256700
H	-1.60715500	-1.62121100	-2.76253600
C	-4.69389500	-0.35355800	-2.13606200
H	-5.12160700	1.21489700	-0.72382900
H	-3.95701300	-1.86984800	-3.47796700
H	-5.72745600	-0.46519200	-2.44840300

Cartesian coordinates (Å) and absolute energies (a. u.) for all stationary points from the optimization and frequency calculations using Basis set I. Energies in kcal/mol at 110ºC

1b

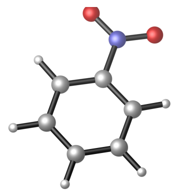

G = -436.554344

E = -436.615062001

C	-0.42489100	1.21917500	-0.00006000
C	-1.81473100	1.20997800	-0.00006700
C	-2.50729000	-0.00000400	0.00000100
C	-1.81473600	-1.20997400	0.00006900
C	-0.42488500	-1.21917300	0.00005300
C	0.24352600	-0.00000400	-0.00000200
H	0.13378200	2.14664500	-0.00010300
H	-2.35577700	2.15003600	-0.00012300
H	-3.59271200	0.00000400	0.00000400
H	-2.35576800	-2.15004100	0.00013000
N	1.70791700	0.00000100	0.00000100
O	2.28370500	1.07917700	0.00016300
O	2.28371100	-1.07917600	-0.00016000
H	0.13376700	-2.14665500	0.00009900

1b-*d*$_5$

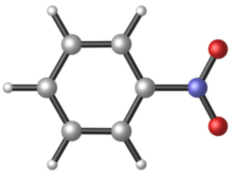

G = -436.572110

E = -436.615062001

C	-0.42489100	1.21917500	-0.00006000
C	-1.81473100	1.20997800	-0.00006700
C	-2.50729000	-0.00000400	0.00000100
C	-1.81473600	-1.20997400	0.00006900
C	-0.42488500	-1.21917300	0.00005300
C	0.24352600	-0.00000400	-0.00000200
H(Iso=2)	0.13378200	2.14664500	-0.00010300
H(Iso=2)	-2.35577700	2.15003600	-0.00012300
H(Iso=2)	-3.59271200	0.00000400	0.00000400
H(Iso=2)	-2.35576800	-2.15004100	0.00013000
N	1.70791700	0.00000100	0.00000100
O	2.28370500	1.07917700	0.00016300
O	2.28371100	-1.07917600	-0.00016000
H(Iso=2)	0.13376700	-2.14665500	0.00009900

2a

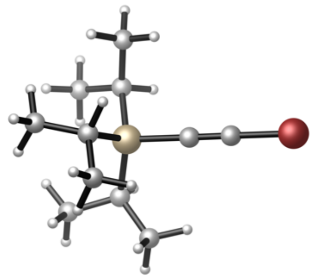

G = -734.113256

E = -734.344017649

Si	-1.00441300	0.08666800	-0.11599200
C	-1.55958800	-0.14190800	1.68375900
C	-1.45171500	1.78937100	-0.82377200
C	-1.52907100	-1.29700000	-1.31028100
H	-0.97898900	0.60782300	2.24122700
H	-0.77808800	1.90554200	-1.68561800
H	-1.45427500	-0.83852900	-2.30794400
C	-1.17316500	-1.52486100	2.22843200
C	-3.04650900	0.15450200	1.92727400
C	-0.59745100	-2.51763100	-1.28972400
C	-2.98692800	-1.73981800	-1.10565800
C	-1.13862000	2.91830200	0.16813700
C	-2.89006000	1.89124500	-1.35100900
H	-0.92229300	-3.25970000	-2.02959700
H	0.43841800	-2.24955800	-1.51931200
H	-0.60227100	-3.00983300	-0.31071800
H	-3.10899500	-2.26106500	-0.14963700
H	-3.69295400	-0.90328200	-1.11916900
H	-3.28935600	-2.43807800	-1.89582500
H	-3.29119800	0.02376900	2.98888800
H	-3.30932600	1.18182400	1.65577400
H	-3.69675800	-0.51817600	1.35856900
H	-1.38244600	-1.59218100	3.30321700
H	-1.74229900	-2.32135300	1.73590200
H	-0.10799700	-1.73777900	2.08549900
H	-3.62933700	1.75971000	-0.55282600
H	-3.06504800	2.87868200	-1.79625800
H	-3.09538100	1.14389600	-2.12447100

H	-0.10575400	2.86984900	0.53128700
H	-1.27837000	3.89972900	-0.30151400
H	-1.80081700	2.87797900	1.04075000
C	0.85399900	0.07310700	-0.05092300
C	2.06949600	0.07654600	-0.02098600
Br	3.87580000	0.07646400	0.00411900

I

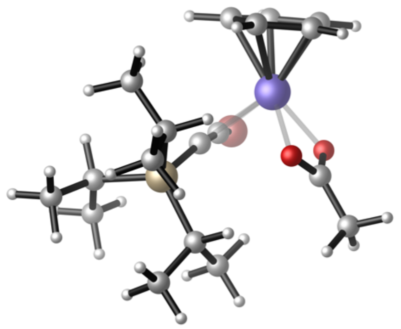

G = -1265.280255

E = -1265.63043712

C	-0.58716600	1.38677500	-0.52641100
C	0.34742900	0.58374600	-0.42125100
C	-1.74699000	-2.45853800	-1.15378400
C	-1.89861800	-1.33227000	-1.99813800
C	-2.79864000	-2.41849400	-0.16873000
H	-1.24193400	-1.04960000	-2.80933200
H	-2.94924300	-3.13196100	0.62989800
C	-3.03101500	-0.57858000	-1.54214700
H	-3.40987600	0.33308600	-1.98167500
C	-3.60864900	-1.28256500	-0.44254700
H	-4.45193700	-0.94870800	0.14792000
H	-0.95408300	-3.19237900	-1.20745200
Si	2.07657100	-0.15167800	-0.19469900
C	2.54248900	0.18984700	1.61307600
H	2.07049900	-0.63121100	2.16972000
C	1.92531600	-1.99842300	-0.57445900
H	1.00827200	-2.30670300	-0.05526500
C	3.09119100	0.78889700	-1.49221300
H	2.53664200	0.65027000	-2.43175300
O	-0.43395800	-1.19400100	1.78836500
C	-1.02386200	-0.29380300	2.46668000
O	-1.91750300	0.38136800	1.86107100
C	-0.70236800	-0.05710300	3.90136700
H	0.33862600	-0.31557000	4.10113600
H	-0.89829900	0.98253000	4.16643100
H	-1.34425800	-0.70076000	4.51190100
Rh	-1.56959300	-0.66198200	0.04729600
C	4.06147400	0.11402000	1.84039600
C	1.99629100	1.51446800	2.16587200
C	3.16666800	2.29506100	-1.20536600
C	4.49821800	0.20424600	-1.69624800
C	3.07617200	-2.83878700	-0.00273000
C	1.73753200	-2.25896600	-2.07527400
H	4.28520100	0.15541100	2.91313700
H	4.50683700	-0.80518300	1.44733200
H	4.57161300	0.96150000	1.36966800
H	3.16203100	-2.72680200	1.08285000
H	2.91030300	-3.90248600	-0.21188900
H	4.04031100	-2.56677500	-0.44626300
H	2.65346900	-2.04273200	-2.63564700
H	1.48650400	-3.31033500	-2.26024000
H	0.94102800	-1.64433500	-2.50802800
H	2.45112700	2.37467000	1.66257600
H	0.91254100	1.60807300	2.04926100
H	2.22418000	1.60539700	3.23452100
H	5.11988300	0.32786300	-0.80430700
H	4.47615400	-0.86191900	-1.94115700
H	5.00534600	0.72029400	-2.52054700
H	3.66625400	2.81829400	-2.02944900
H	2.17703000	2.74892000	-1.08096400
H	3.74146700	2.49572600	-0.29463000
Br	-1.56963300	2.89748500	-0.71412500

I^{Cp*}

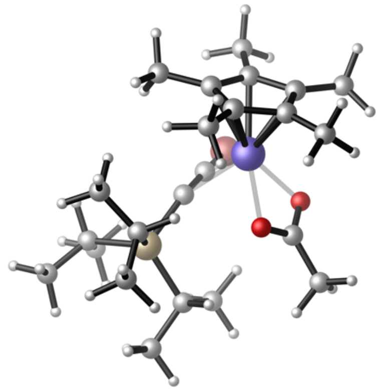

G = -1461.734137

E = -1462.21289103

C	0.06350100	1.60784900	-0.37331000
C	0.84827200	0.65450300	-0.38048700
C	-2.04085500	-1.97195900	-0.95224100
C	-2.00406500	-0.84160100	-1.81867800
C	-2.89636600	-1.64536500	0.17539800
C	-2.83881700	0.18747600	-1.24554100

C	-3.42811200	-0.33161000	-0.04450900
Si	2.47548500	-0.28733600	-0.20227500
C	3.00315800	-0.03749400	1.60557900
H	2.47310100	-0.83106400	2.14990100
C	2.15100400	-2.10292400	-0.61762800
H	1.18746900	-2.32070700	-0.13862600
C	3.57885100	0.56434800	-1.49108400
H	3.01366900	0.47912500	-2.43091000
O	-0.10268500	-1.21836600	1.76849200
C	-0.43698300	-0.29173400	2.57105800
O	-1.19329000	0.62934700	2.12918400
C	0.02192300	-0.30132500	3.99301600
H	1.02173700	-0.73313700	4.06524400
H	0.01017100	0.70704500	4.40774500
H	-0.66245600	-0.92763800	4.57485000
Rh	-1.26828500	-0.28383300	0.17271600
C	4.51312900	-0.25339400	1.79948600
C	2.58420400	1.31192800	2.20683600
C	3.78785700	2.05705000	-1.20104200
C	4.93031200	-0.13790600	-1.70252100
C	3.18754100	-3.06421600	-0.01874400
C	2.00062400	-2.32067300	-2.12866700
H	4.75896000	-0.26712300	2.86824600
H	4.86854800	-1.19467800	1.36857100
H	5.08792900	0.56051000	1.34422000
H	3.25018200	-2.97153700	1.07030800
H	2.92000000	-4.10366600	-0.24525900
H	4.18913500	-2.89183700	-0.42880000
H	2.94991600	-2.16856400	-2.65320300
H	1.67487200	-3.34538500	-2.34495500
H	1.26963200	-1.63601800	-2.57010200
H	3.08103600	2.14659400	1.69971300
H	1.50694400	1.48735800	2.13856800
H	2.86363100	1.36100700	3.26628400
H	5.57218100	-0.05088400	-0.82070700
H	4.82097800	-1.20318300	-1.92797400
H	5.46645400	0.32206000	-2.54150200
H	4.33766500	2.53621000	-2.02004600
H	2.84054200	2.59424900	-1.08331900
H	4.37033500	2.20422200	-0.28499800
Br	-0.59561200	3.29638300	-0.32569800
C	-1.29648000	-0.70566700	-3.12344900
C	-3.18844100	1.47344000	-1.91014500
C	-4.37250000	0.40042700	0.84066300
C	-3.23577900	-2.55873000	1.30371100
C	-1.35247400	-3.27787500	-1.13755700
H	-3.46222800	2.24280100	-1.18668900
H	-2.36971500	1.84056400	-2.53162600
H	-4.05290100	1.30292600	-2.56155600
H	-5.38763500	0.27654800	0.44690000
H	-4.35075500	0.01346400	1.86016700
H	-4.14748100	1.46872200	0.86190400
H	-4.17713900	-3.07454800	1.08652900
H	-2.45858900	-3.31085300	1.44824000
H	-3.35847600	-2.00552700	2.23714100
H	-2.09718400	-4.03373300	-1.40924300
H	-0.60281100	-3.23109500	-1.92687100
H	-0.87030300	-3.60491800	-0.21308400
H	-2.03328900	-0.50360100	-3.90737600
H	-0.59093500	0.12982200	-3.10429600
H	-0.75993500	-1.61690900	-3.38660100

II_D^{Cp}

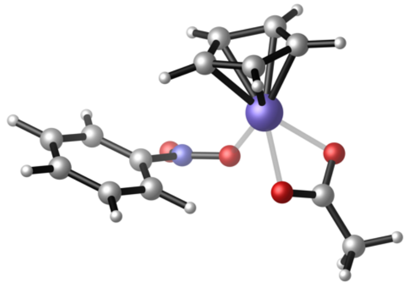

G = -967.728581

E = -967.885749345

O	1.41122800	1.19570800	-1.27993500
C	2.31191300	1.66959700	-0.51247300
O	2.61075900	0.98357100	0.51410900
C	-2.23607900	0.68609300	0.39028700
C	-1.86446900	0.95984000	-0.92362900
C	-3.43531100	0.06820000	0.73127000
C	-2.73858800	0.58453400	-1.93518600
C	-4.28359300	-0.31782500	-0.29807700
C	-3.93668100	-0.05949600	-1.62430300
H(Iso=2)	-0.92965900	1.46374500	-1.14351700
H(Iso=2)	-2.48519900	0.79791800	-2.96758000
H(Iso=2)	-5.21703500	-0.81760800	-0.06469300
H(Iso=2)	-4.60946000	-0.35703400	-2.42175500
Rh	1.05314100	-0.41933400	0.07518600
C	0.06696400	-2.19337300	0.83586600
H	-0.69093100	-2.13832100	1.60702800
C	1.46426000	-2.28885400	1.05907000
H	1.95469500	-2.34487200	2.02087200
C	2.11497000	-2.22614800	-0.22207100

H	3.18200800	-2.24291700	-0.39859800
C	-0.16812700	-2.08199000	-0.57184500
H	-1.12934400	-1.96181200	-1.05192600
C	1.10060500	-2.11687300	-1.22323600
H	1.27271900	-1.99955600	-2.28514400
C	2.96088300	2.98341100	-0.78266200
H	4.03522400	2.90783800	-0.60223400
H	2.76793800	3.30593700	-1.80588800
H	2.55317900	3.72290900	-0.08624000
O	-0.11178000	1.10826200	1.25190400
O	-1.80426600	1.36003100	2.54435800
N	-1.34301400	1.06693000	1.46640400
H(Iso=2)	-3.68241300	-0.11799800	1.76992200

II_D^{Cp*}

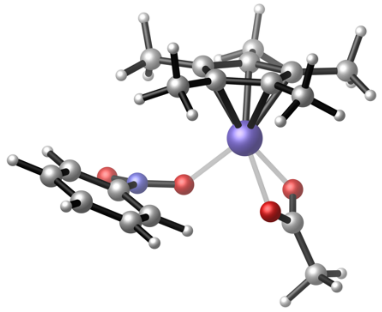

G = -1164.185030

E = -1164.47328485

O	-0.61792400	2.14083900	0.73277400
C	-1.35914000	2.62401700	-0.18292500
O	-1.90769200	1.81940700	-0.99451900
C	2.67787800	0.31822000	-0.59625200
C	2.38930700	1.20572500	0.43601300
C	3.74677500	-0.57360300	-0.55375100
C	3.20509400	1.18322100	1.56054800
C	4.53644800	-0.59277200	0.58864600
C	4.26704800	0.28325100	1.64060100
H(Iso=2)	1.55383800	1.89317400	0.36556500
H(Iso=2)	3.00774900	1.87136800	2.37499400
H(Iso=2)	5.36218500	-1.29230700	0.65589300
H(Iso=2)	4.89124700	0.26555500	2.52806200
Rh	-0.86267000	0.12681200	-0.10088400
C	-0.46936700	-2.00117700	-0.21259000
C	-1.81433200	-1.71410100	-0.61057500
C	-2.48290200	-1.09290900	0.51122600
C	-0.27857300	-1.53548200	1.13279700
C	-1.53221600	-0.98450700	1.58581000
C	-1.53678100	4.10276900	-0.31292100
H	-0.63226000	4.52248700	-0.76508900
H	-2.39190200	4.33727300	-0.94729200
H	-1.65932200	4.55442200	0.67364900
O	0.67062900	0.75095600	-1.69777500
O	2.28723900	-0.10423300	-2.82261200
N	1.83486300	0.31189700	-1.77868000
C	0.55654200	-2.64633900	-1.07711800
C	-2.43101000	-2.02884100	-1.92986100
C	0.95647300	-1.67842100	1.95406500
C	-1.79137200	-0.40007500	2.92888900
C	-3.90578000	-0.65923700	0.54152700
H	1.85409500	-1.70256000	1.33365800
H	1.05244500	-0.86256900	2.67247100
H	0.90382200	-2.61906800	2.51318000
H	1.56621900	-2.48480700	-0.69673900
H	0.37063000	-3.72566600	-1.09374000
H	0.49633800	-2.28725600	-2.10741000
H	-1.67504300	-2.09151200	-2.71432400
H	-2.94681200	-2.99300700	-1.87017300
H	-3.16232000	-1.26932300	-2.21383800
H	-4.52751600	-1.50753300	0.84863400
H	-4.06026300	0.15342700	1.25287000
H	-4.24059100	-0.33288000	-0.44453100
H	-0.88369500	0.02797100	3.35712800
H	-2.55763300	0.37569800	2.88470100
H	-2.14477300	-1.19238700	3.59803500
H(Iso=2)	3.93711700	-1.24450800	-1.38286500

II_H^{Cp}

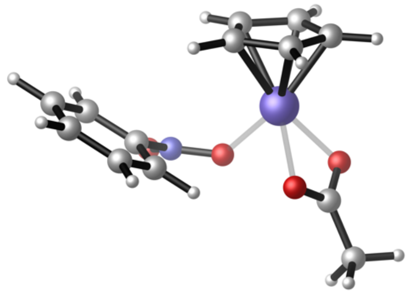

G = -967.710360

E = -967.885749345

O	1.41122800	1.19570800	-1.27993500
C	2.31191300	1.66959700	-0.51247300
O	2.61075900	0.98357100	0.51410900
C	-2.23607900	0.68609300	0.39028700

C	-1.86446900	0.95984000	-0.92362900
C	-3.43531100	0.06820000	0.73127000
C	-2.73858800	0.58453400	-1.93518600
C	-4.28359300	-0.31782500	-0.29807700
C	-3.93668100	-0.05949600	-1.62430300
H	-0.92965900	1.46374500	-1.14351700
H	-2.48519900	0.79791800	-2.96758000
H	-5.21703500	-0.81760800	-0.06469300
H	-4.60946000	-0.35703400	-2.42175500
Rh	1.05314100	-0.41933400	0.07518600
C	0.06696400	-2.19337300	0.83586600
H	-0.69093100	-2.13832100	1.60702800
C	1.46426000	-2.28885400	1.05907000
H	1.95469500	-2.34487200	2.02087200
C	2.11497000	-2.22614800	-0.22207100
H	3.18200800	-2.24291700	-0.39859800
C	-0.16812700	-2.08199000	-0.57184500
H	-1.12934400	-1.96181200	-1.05192600
C	1.10060500	-2.11687300	-1.22323600
H	1.27271900	-1.99955600	-2.28514400
C	2.96088300	2.98341100	-0.78266200
H	4.03522400	2.90783800	-0.60223400
H	2.76793800	3.30593700	-1.80588800
H	2.55317900	3.72290900	-0.08624000
O	-0.11178000	1.10826200	1.25190400
O	-1.80426600	1.36003100	2.54435800
N	-1.34301400	1.06693000	1.46640400
H	-3.68241300	-0.11799800	1.76992200

II_H^{Cp*}

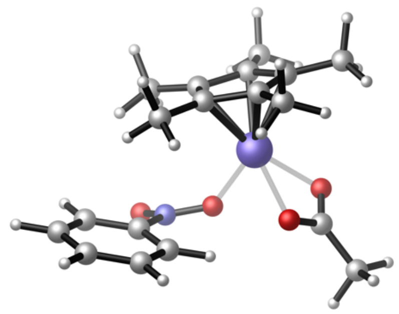

G = -1164.167488

E = -1164.47328421

O	-0.61770700	2.14082000	0.73281400
C	-1.35891300	2.62410500	-0.18283100
O	-1.90745800	1.81958100	-0.99452100
C	2.67808300	0.31802200	-0.59620800
C	2.38968200	1.20559400	0.43603500
C	3.74696200	-0.57383100	-0.55379100
C	3.20563900	1.18316000	1.56044600
C	4.53682300	-0.59291500	0.58847800
C	4.26759400	0.28318500	1.64041100
H	1.55426500	1.89309500	0.36566400
H	3.00842200	1.87137600	2.37486500
H	5.36258200	-1.29243400	0.65563200
H	4.89193600	0.26556400	2.52777200
Rh	-0.86270200	0.12685000	-0.10087700
C	-0.46976800	-2.00123300	-0.21257100
C	-1.81465600	-1.71391500	-0.61062400
C	-2.48316700	-1.09261900	0.51114200
C	-0.27895700	-1.53558800	1.13283300
C	-1.53251600	-0.98436600	1.58577100
C	-1.53638200	4.10288000	-0.31277900
H	-0.63176300	4.52254900	-0.76479300
H	-2.39140900	4.33750700	-0.94722800
H	-1.65900400	4.55447700	0.67380900
O	0.67079700	0.75087200	-1.69769300
O	2.28730200	-0.10459400	-2.82248600
N	1.83500200	0.31172300	-1.77859500
C	0.55605700	-2.64663600	-1.07702700
C	-2.43134100	-2.02855700	-1.92993300
C	0.95602400	-1.67880900	1.95415200
C	-1.79165000	-0.39987300	2.92882900
C	-3.90596700	-0.65869800	0.54138300
H	1.85365000	-1.70322400	1.33376900
H	1.05219700	-0.86295400	2.67252700
H	0.90310600	-2.61941900	2.51330200
H	1.56575000	-2.48532700	-0.69660000
H	0.36990400	-3.72592100	-1.09362500
H	0.49597800	-2.28758600	-2.10733700
H	-1.67536200	-2.09137800	-2.71437100
H	-2.94733800	-2.99261800	-1.87025500
H	-3.16249500	-1.26890300	-2.21394800
H	-4.52785100	-1.50686000	0.84856200
H	-4.06031100	0.15405200	1.25266100
H	-4.24070700	-0.33236600	-0.44470700
H	-0.88391800	0.02799900	3.35712600
H	-2.55774400	0.37606200	2.88456500
H	-2.14526500	-1.19209800	3.59796400
H	3.93720500	-1.24475900	-1.38291000

III_D^{Cp}

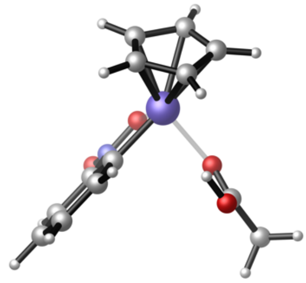

G = -967.733425

E = -967.894374582

O	-0.23634000	2.80100100	1.06977000
C	-0.08200000	2.71097400	-0.23207200
O	-0.32499400	1.68229300	-0.86918800
C	1.95699600	-0.70969000	-0.30759200
C	0.96532100	-0.41600400	0.63043000
C	3.31568700	-0.82355800	-0.01373600
C	1.38732800	-0.23241000	1.94731600
C	3.70125900	-0.63501300	1.30261500
C	2.73844100	-0.34220300	2.27417800
H(Iso=2)	-0.55327900	1.95057800	1.42540600
H(Iso=2)	0.66690600	-0.00605900	2.72699700
H(Iso=2)	4.74673300	-0.71929400	1.57662900
H(Iso=2)	3.04769800	-0.19942900	3.30509100
Rh	-0.88837100	-0.28543600	-0.12914600
C	-2.02985400	-2.06504000	0.15335400
H	-1.66693600	-3.03308200	-0.16563200
C	-2.93892800	-1.21221400	-0.59704000
H	-3.30052100	-1.40587700	-1.59742500
C	-3.15822000	-0.06471200	0.15444600
H	-3.72098500	0.80617300	-0.15460900
C	-1.79834400	-1.46566300	1.42363800
H	-1.19506300	-1.87508800	2.22185300
C	-2.40192000	-0.17569800	1.39391900
H	-2.42047100	0.53760700	2.20738300
C	0.40591000	3.95888800	-0.88014600
H	-0.32861800	4.75400800	-0.72416200
H	1.34015100	4.26976500	-0.40518500
H	0.55898100	3.79263200	-1.94464900
O	0.26602300	-0.82727100	-1.86260500
O	2.27611100	-1.13286900	-2.55957200
N	1.50765300	-0.90357900	-1.65561700
H(Iso=2)	4.03204800	-1.05345300	-0.79392000

III_D^{Cp*}

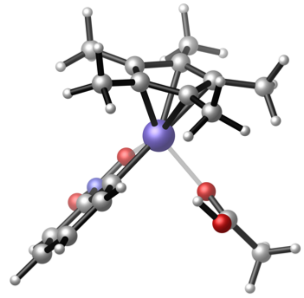

G = -1164.181038

E = -1164.47117523

O	0.41876300	3.01961100	0.84183700
C	0.27965700	2.93716900	-0.46601900
O	0.03213100	1.87995100	-1.04621800
C	2.43902100	-0.59622700	-0.44359700
C	1.49203800	-0.21227200	0.51031400
C	3.80424200	-0.74582600	-0.19819900
C	1.99228900	0.04318700	1.79043700
C	4.25783200	-0.49021600	1.08469200
C	3.35056400	-0.09238700	2.07181600
H(Iso=2)	0.33466100	2.13039500	1.23045900
H(Iso=2)	1.31716200	0.34704800	2.58669700
H(Iso=2)	5.31097600	-0.59869200	1.31821700
H(Iso=2)	3.70997300	0.10917100	3.07647900
Rh	-0.41594700	-0.10294400	-0.17608400
C	-1.57721600	-1.85603900	0.13303000
C	-2.45633700	-0.97282500	-0.63761700
C	-2.63796900	0.19823000	0.10685900
C	-1.32924500	-1.25311100	1.40739000
C	-1.88157200	0.06977400	1.35775000
C	0.44723400	4.23386700	-1.18001800
H	0.28342400	4.09548000	-2.24711100
H	-0.26246100	4.96273900	-0.77945900
H	1.45557700	4.61707700	-1.00108500
O	0.70823200	-0.72758300	-1.95965100
O	2.68831800	-1.15826400	-2.67567900
N	1.94382000	-0.84714800	-1.77232100
C	-1.16644500	-3.22663200	-0.28687600
C	-2.99460600	-1.29990600	-1.99069000
C	-0.65480600	-1.89719800	2.57056300
C	-1.91441900	1.04612800	2.48768900
C	-3.38765300	1.42636600	-0.28588100
H	0.16618300	-2.54352400	2.25538600
H	-0.26722000	-1.15819900	3.27352000
H	-1.38708000	-2.51392900	3.10296700
H	-0.25017200	-3.53801900	0.21864100

H	-1.95861000	-3.93874600	-0.03059600
H	-1.00439000	-3.27683800	-1.36561300
H	-2.20881700	-1.68690400	-2.64513500
H	-3.76488100	-2.07407300	-1.90643100
H	-3.43934700	-0.42579900	-2.46861000
H	-4.33686300	1.47041600	0.25903900
H	-2.82159600	2.32753300	-0.03401900
H	-3.60102700	1.44470800	-1.35553800
H	-0.98636500	1.01925400	3.06586100
H	-2.07182800	2.06569800	2.12900700
H	-2.73799900	0.79886900	3.16611100
H(Iso=2)	4.47638200	-1.05334900	-0.99095100

III$_{H}^{Cp}$

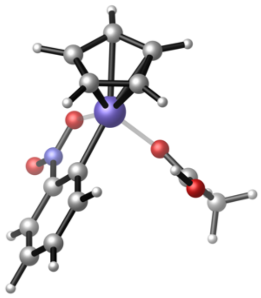

G = -967.715143

E = -967.894374323

O	-0.23619400	2.80143500	1.07112100
C	-0.08177600	2.71129300	-0.23072900
O	-0.32507400	1.68284600	-0.86814600
C	1.95702800	-0.70933200	-0.30795000
C	0.96531900	-0.41642100	0.63027500
C	3.31572200	-0.82331800	-0.01415700
C	1.38724100	-0.23418300	1.94738200
C	3.70124600	-0.63585400	1.30235900
C	2.73836400	-0.34410100	2.27417300
H	-0.55326900	1.95115400	1.42694000
H	0.66677000	-0.00879100	2.72729800
H	4.74672200	-0.72027800	1.57632300
H	3.04757100	-0.20231000	3.30523500
Rh	-0.88836400	-0.28541300	-0.12923500
C	-2.02972000	-2.06530600	0.15230900
H	-1.66670700	-3.03315800	-0.16714500
C	-2.93881500	-1.21215900	-0.59771700
H	-3.30035600	-1.40535200	-1.59821100
C	-3.15829700	-0.06510000	0.15437300
H	-3.72117300	0.80587200	-0.15422700
C	-1.79823100	-1.46652300	1.42287700
H	-1.19488700	-1.87625800	2.22088500

C	-2.40207500	-0.17667900	1.39384300
H	-2.42083100	0.53614300	2.20772900
C	0.40554800	3.95942600	-0.87877600
H	-0.32855200	4.75468700	-0.72152100
H	1.34042400	4.26973200	-0.40469000
H	0.55739800	3.79368200	-1.94353300
O	0.26607100	-0.82603500	-1.86302700
O	2.27617500	-1.13109600	-2.56018800
N	1.50771600	-0.90238000	-1.65609400
H	4.03211800	-1.05252100	-0.79450800

III$_{H}^{Cp*}$

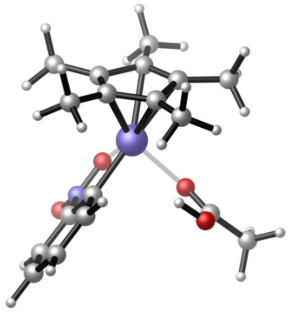

G = -1164.163249

E = -1164.47117523

O	0.41877200	3.01921200	0.84330200
C	0.27985100	2.93731500	-0.46454200
O	0.03228500	1.88036300	-1.04523600
C	2.43900600	-0.59605000	-0.44396500
C	1.49203700	-0.21273100	0.51021300
C	3.80420200	-0.74602300	-0.19866200
C	1.99232700	0.04175400	1.79051100
C	4.25782000	-0.49139100	1.08441700
C	3.35058900	-0.09412400	2.07179800
H	0.33463300	2.12985300	1.23162000
H	1.31720500	0.34501500	2.58699100
H	5.31094000	-0.60022300	1.31788900
H	3.71000100	0.10671200	3.07660300
Rh	-0.41590900	-0.10291300	-0.17603600
C	-1.57726700	-1.85600800	0.13205100
C	-2.45652400	-0.97243400	-0.63804600
C	-2.63804600	0.19822000	0.10706800
C	-1.32932600	-1.25383800	1.40677800
C	-1.88161800	0.06908100	1.35787800
C	0.44770100	4.23421300	-1.17809400
H	0.28409700	4.09615100	-2.24525000
H	-0.26198300	4.96304900	-0.77740800
H	1.45606000	4.61725200	-0.99884000
O	0.70820900	-0.72580300	-1.96015100

O	2.68829500	-1.15569000	-2.67663500
N	1.94376500	-0.84578700	-1.77288500
C	-1.16637800	-3.22629800	-0.28873400
C	-2.99504700	-1.29864300	-1.99122600
C	-0.65486700	-1.89864600	2.56955300
C	-1.91452100	1.04478500	2.48841800
C	-3.38765900	1.42661200	-0.28497300
H	0.16607200	-2.54482400	2.25393900
H	-0.26720200	-1.16010400	3.27294000
H	-1.38716100	-2.51567100	3.10159300
H	-0.25006500	-3.53793200	0.21654400
H	-1.95847500	-3.93868000	-0.03297900
H	-1.00431000	-3.27573100	-1.36750800
H	-2.20946400	-1.68531300	-2.64611500
H	-3.76536600	-2.07280600	-1.90732500
H	-3.43976800	-0.42419100	-2.46852900
H	-4.33691100	1.47035300	0.25990100
H	-2.82160600	2.32760700	-0.03250200
H	-3.60100000	1.44563700	-1.35461500
H	-0.98641900	1.01766700	3.06649000
H	-2.07213200	2.06453100	2.13035900
H	-2.73799100	0.79694400	3.16675800
H	4.47625600	-1.05314400	-0.99164800

TS$_D^{Cp}_{II-III}$

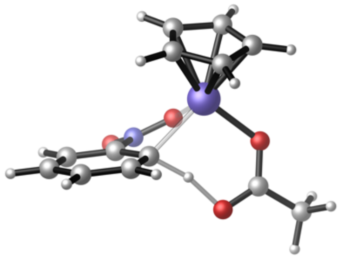

G = -967.697845

E = -967.855494056

O	0.41126700	2.43419800	-1.04138900
C	1.56061900	2.29105200	-0.55923600
O	1.97270700	1.20840700	-0.02215400
C	-2.00528200	0.43301200	0.41939500
C	-1.15271300	0.35268300	-0.69065900
C	-3.36441500	0.17042700	0.38700100
C	-1.74248600	-0.03517500	-1.89791200
C	-3.90996300	-0.22340100	-0.83253100
C	-3.10453000	-0.32716100	-1.96602500
H(Iso=2)	-0.26579800	1.25224600	-0.83841500
H(Iso=2)	-1.13187100	-0.08094300	-2.79388100
H(Iso=2)	-4.96895400	-0.44755900	-0.89548700

H(Iso=2)	-3.54489300	-0.62873900	-2.91048800
Rh	0.77494400	-0.48119800	0.11809200
C	0.50488200	-2.52827800	0.80120300
H	-0.22080400	-2.80542600	1.55362800
C	1.86002800	-2.09022900	1.05258100
H	2.32826200	-1.99613300	2.02290300
C	2.46038400	-1.79549700	-0.19692000
H	3.45284400	-1.39022600	-0.34179500
C	0.28082800	-2.48151200	-0.59051800
H	-0.65168700	-2.70305000	-1.09139900
C	1.47503900	-1.97404800	-1.21968100
H	1.61686700	-1.80491200	-2.27809800
C	2.54555300	3.42452300	-0.61375400
H	3.34416900	3.16349000	-1.31415000
H	2.06002300	4.34310800	-0.94116400
H	2.99865400	3.56440600	0.37022000
O	-0.15488400	0.62343500	1.79316300
O	-2.05579100	1.24364300	2.58580100
N	-1.39334900	0.79464800	1.68294900
H(Iso=2)	-3.96990000	0.24926100	1.28229700

TS$_D^{Cp*}_{II-III}$

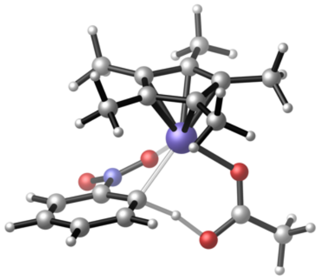

G = -1164.155084

E = -1164.44085252

O	-0.33225500	2.99475600	-0.54985200
C	0.88767700	2.96972100	-0.22226500
O	1.50902200	1.90797700	0.08576100
C	-2.34439200	0.35189500	0.47297400
C	-1.51927000	0.68117300	-0.60955100
C	-3.61892000	-0.18482700	0.36108700
C	-2.06351600	0.46362200	-1.87973400
C	-4.10959400	-0.40727400	-0.92205100
C	-3.33983200	-0.07365200	-2.03690900
H(Iso=2)	-0.78374900	1.77806300	-0.55061400
H(Iso=2)	-1.48133500	0.73363900	-2.75592600
H(Iso=2)	-5.09769500	-0.83556000	-1.04950500
H(Iso=2)	-3.74055500	-0.23550000	-3.03235100
Rh	0.55604800	0.01805200	0.08139400
C	0.55935800	-2.06303600	0.63969100

C	1.85412300	-1.46069000	0.92663000
C	2.41201600	-1.01442900	-0.30924800
C	0.33107300	-1.98246000	-0.75859200
C	1.44621900	-1.26895700	-1.35048900
C	1.66713200	4.25355500	-0.20684000
H	2.15866200	4.36876600	0.76216300
H	2.44716300	4.20249800	-0.97197300
H	1.01839300	5.10665800	-0.40254200
O	-0.57621800	0.75553800	1.91622000
O	-2.55362500	0.65718100	2.75795300
N	-1.81107400	0.59692200	1.80495800
C	-0.35170500	-2.67577400	1.64712200
C	2.48307000	-1.38348600	2.27424000
C	-0.83418400	-2.55936500	-1.48445900
C	1.62928000	-0.97918600	-2.79999100
C	3.72573800	-0.34454500	-0.50511600
H	-1.76732700	-2.40831000	-0.93689000
H	-0.94163500	-2.12802600	-2.47954000
H	-0.67397700	-3.63743700	-1.59355200
H	-1.38001300	-2.70863900	1.28225300
H	-0.02887500	-3.70265600	1.84990100
H	-0.32953000	-2.12572800	2.59051700
H	1.73293900	-1.22535400	3.05165800
H	2.99502200	-2.32875200	2.48561600
H	3.21542700	-0.57668900	2.32830400
H	4.45185500	-1.08793800	-0.85157700
H	3.65797800	0.43972300	-1.26147000
H	4.09770500	0.09443300	0.42102300
H	0.66943300	-0.82916400	-3.29797800
H	2.24192700	-0.08777500	-2.94740800
H	2.13411400	-1.82528700	-3.27890200
H(Iso=2)	-4.20159100	-0.42908900	1.24168500

$TS_H{}^{Cp}{}_{II\text{-}III}$

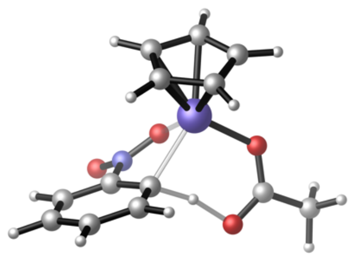

G = -967.681290

E = -967.855493662

O	0.41191000	2.43400200	-1.04218500
C	1.56107100	2.29082400	-0.55959000
O	1.97284300	1.20825200	-0.02211700
C	-2.00536200	0.43312000	0.41951300
C	-1.15274300	0.35288900	-0.69051100
C	-3.36450900	0.17060700	0.38701800
C	-1.74248900	-0.03475400	-1.89784800
C	-3.91002500	-0.22301400	-0.83259500
C	-3.10454600	-0.32665500	-1.96606700
H	-0.26580200	1.25245100	-0.83811500
H	-1.13183800	-0.08040500	-2.79379900
H	-4.96902700	-0.44708900	-0.89563800
H	-3.54488800	-0.62804000	-2.91060100
Rh	0.77490600	-0.48122600	0.11816400
C	0.50468600	-2.52828100	0.80125600
H	-0.22098200	-2.80535900	1.55372400
C	1.85988800	-2.09036700	1.05255500
H	2.32819200	-1.99632700	2.02284900
C	2.46019700	-1.79569100	-0.19698300
H	3.45269000	-1.39052100	-0.34191600
C	0.28055200	-2.48148500	-0.59045200
H	-0.65201700	-2.70292700	-1.09127400
C	1.47477000	-1.97412800	-1.21968700
H	1.61654800	-1.80499500	-2.27811100
C	2.54614100	3.42418500	-0.61392200
H	3.34501600	3.16293400	-1.31393800
H	2.06084400	4.34275900	-0.94170600
H	2.99884900	3.56421600	0.37021400
O	-0.15501000	0.62345800	1.79334000
O	-2.05596400	1.24343400	2.58604100
N	-1.39347900	0.79463200	1.68312600
H	-3.97003700	0.24936900	1.28229100

$TS_H{}^{Cp*}{}_{II\text{-}III}$

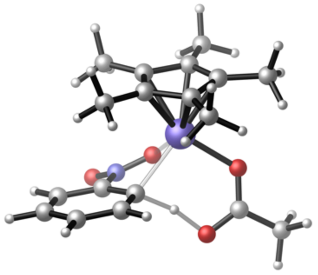

G = -1164.139065

E = -1164.44085252

O	-0.33225500	2.99475600	-0.54985200
C	0.88767700	2.96972100	-0.22226500
O	1.50902200	1.90797700	0.08576100
C	-2.34439200	0.35189500	0.47297400
C	-1.51927000	0.68117300	-0.60955100
C	-3.61892000	-0.18482700	0.36108700

C	-2.06351600	0.46362200	-1.87973400
C	-4.10959400	-0.40727400	-0.92205100
C	-3.33983200	-0.07365200	-2.03690900
H	-0.78374900	1.77806300	-0.55061400
H	-1.48133500	0.73363900	-2.75592600
H	-5.09769500	-0.83556000	-1.04950500
H	-3.74055500	-0.23550000	-3.03235100
Rh	0.55604800	0.01805200	0.08139400
C	0.55935800	-2.06303600	0.63969100
C	1.85412300	-1.46069000	0.92663000
C	2.41201600	-1.01442900	-0.30924800
C	0.33107300	-1.98246000	-0.75859200
C	1.44621900	-1.26895700	-1.35048900
C	1.66713200	4.25355500	-0.20684000
H	2.15866200	4.36876600	0.76216300
H	2.44716300	4.20249800	-0.97197300
H	1.01839300	5.10665800	-0.40254200
O	-0.57621800	0.75553800	1.91622000
O	-2.55362500	0.65718100	2.75795300
N	-1.81107400	0.59692200	1.80495800
C	-0.35170500	-2.67577400	1.64712200
C	2.48307000	-1.38348600	2.27424000
C	-0.83418400	-2.55936500	-1.48445900
C	1.62928000	-0.97918600	-2.79999100
C	3.72573800	-0.34454500	-0.50511600
H	-1.76732700	-2.40831000	-0.93689000
H	-0.94163500	-2.12802600	-2.47954000
H	-0.67397700	-3.63743700	-1.59355200
H	-1.38001300	-2.70863900	1.28225300
H	-0.02887500	-3.70265600	1.84990100
H	-0.32953000	-2.12572800	2.59051700
H	1.73293900	-1.22535400	3.05165800
H	2.99502200	-2.32875200	2.48561600
H	3.21542700	-0.57668900	2.32830400
H	4.45185500	-1.08793800	-0.85157700
H	3.65797800	0.43972300	-1.26147000
H	4.09770500	0.09443300	0.42102300
H	0.66943300	-0.82916400	-3.29797800
H	2.24192700	-0.08777500	-2.94740800
H	2.13411400	-1.82528700	-3.27890200
H	-4.20159100	-0.42908900	1.241685

8. NMR Spectra

^{1}H NMR: 300 MHz, $CDCl_3$, compound **(3a)**

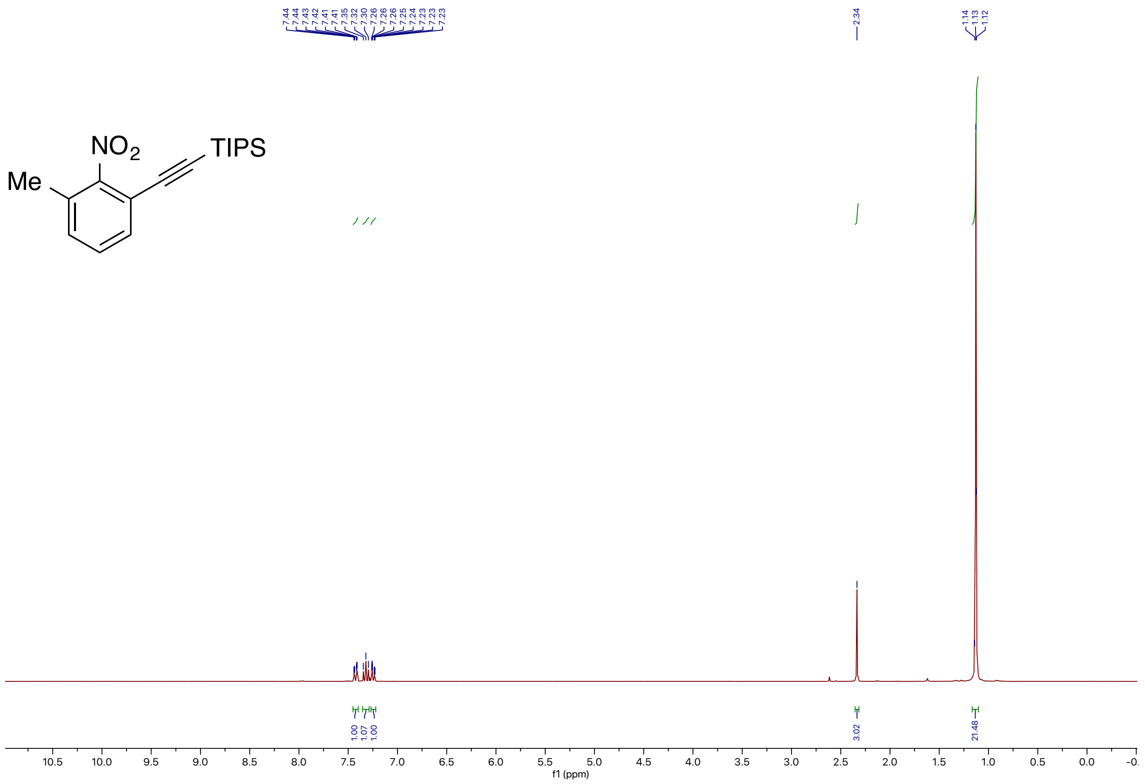

^{13}C NMR: 75 MHz, $CDCl_3$, compound **(3a)**

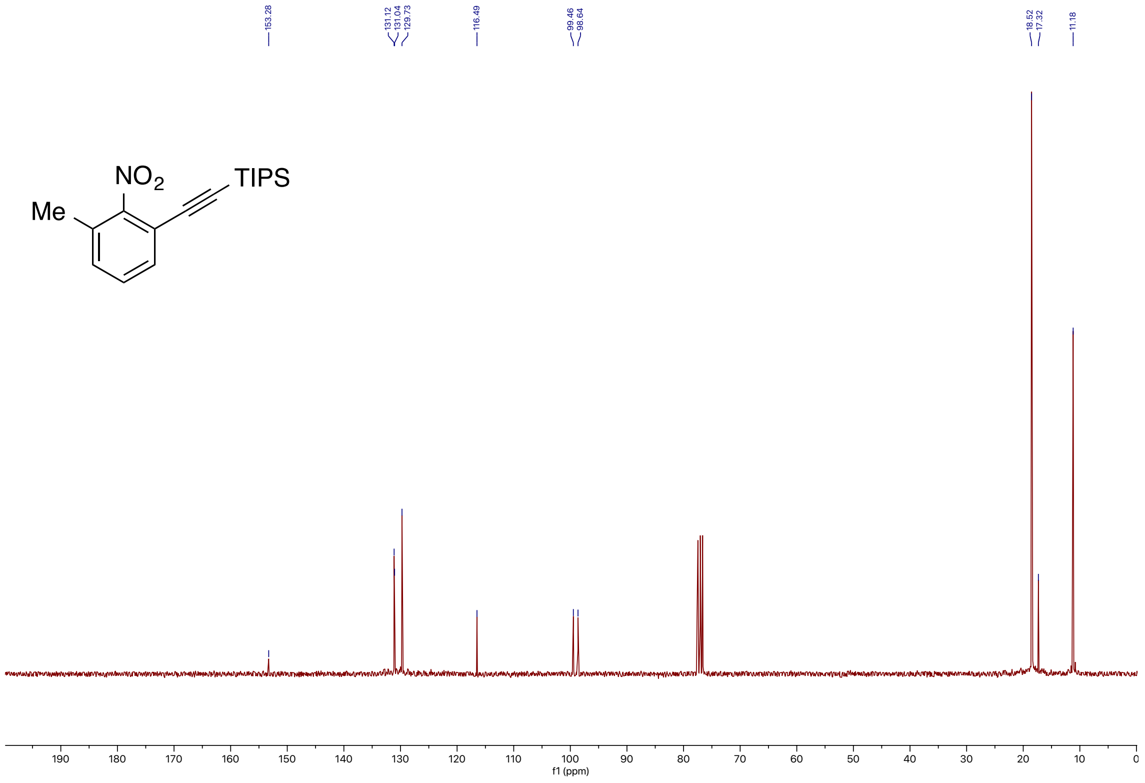

^{1}H NMR: 300 MHz, $CDCl_3$, compound **(3b)**

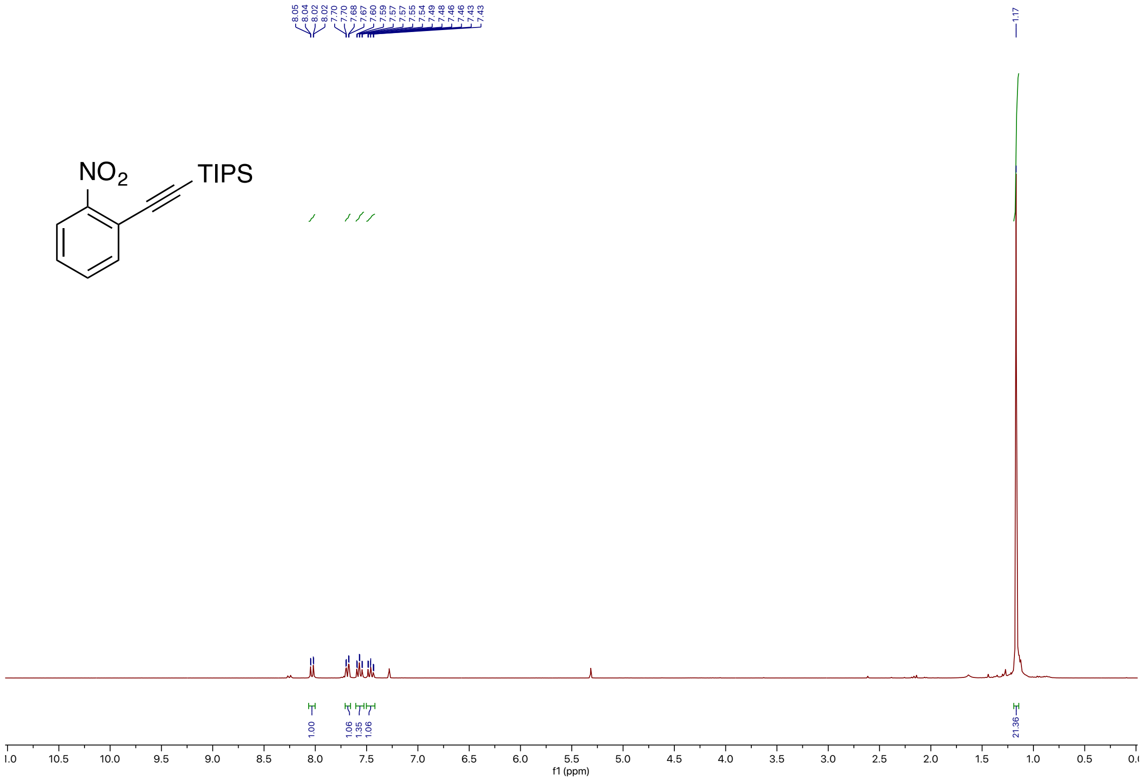

^{13}C NMR: 75 MHz, $CDCl_3$, compound **(3b)**

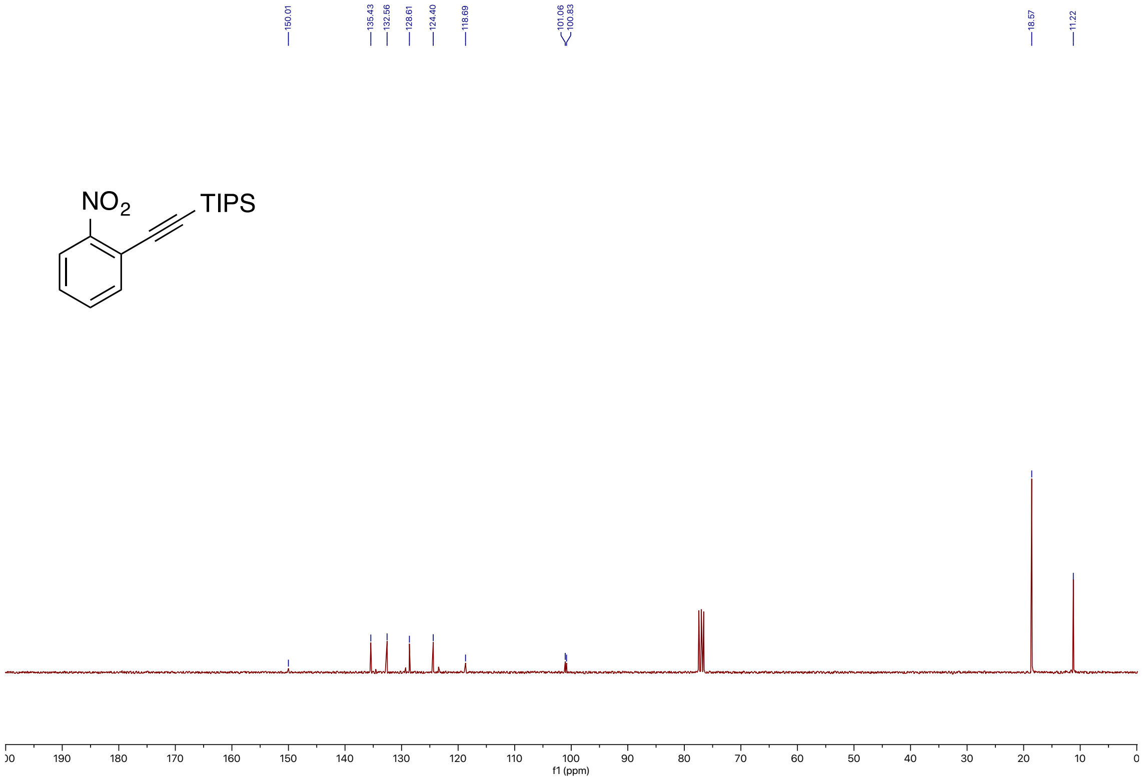

^{1}H NMR: 300 MHz, $CDCl_3$, compound **(3b')**

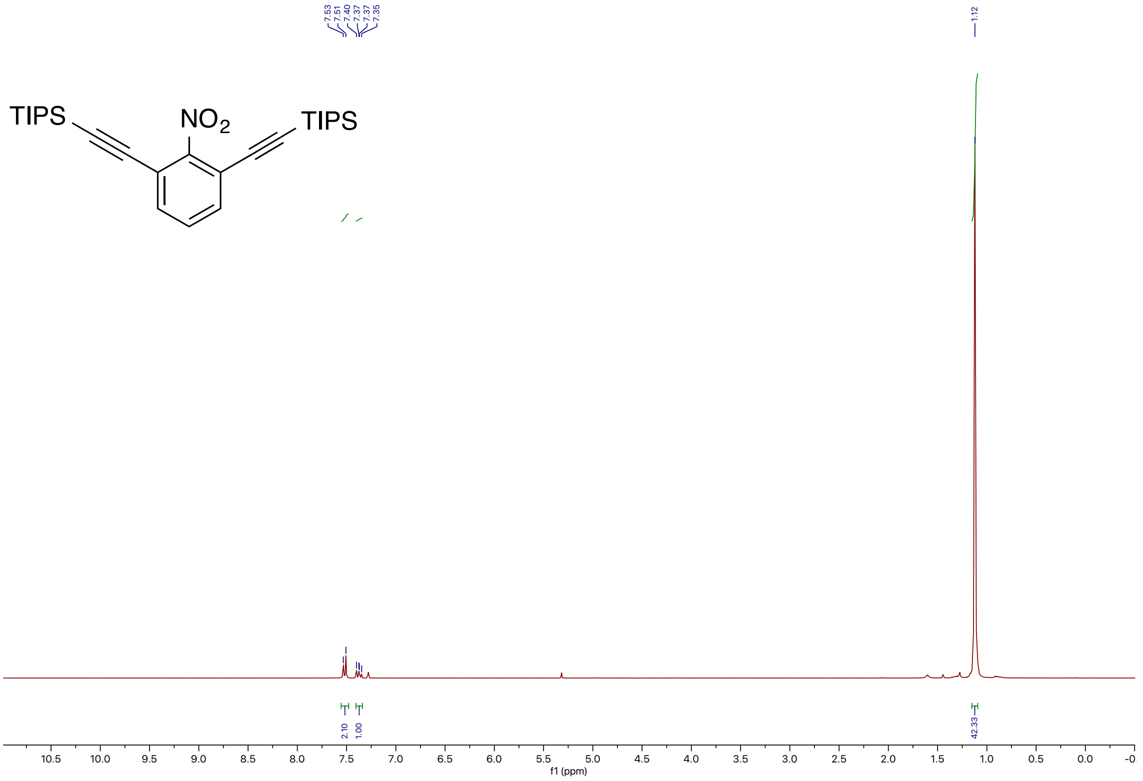

^{13}C NMR: 75 MHz, $CDCl_3$, compound **(3b')**

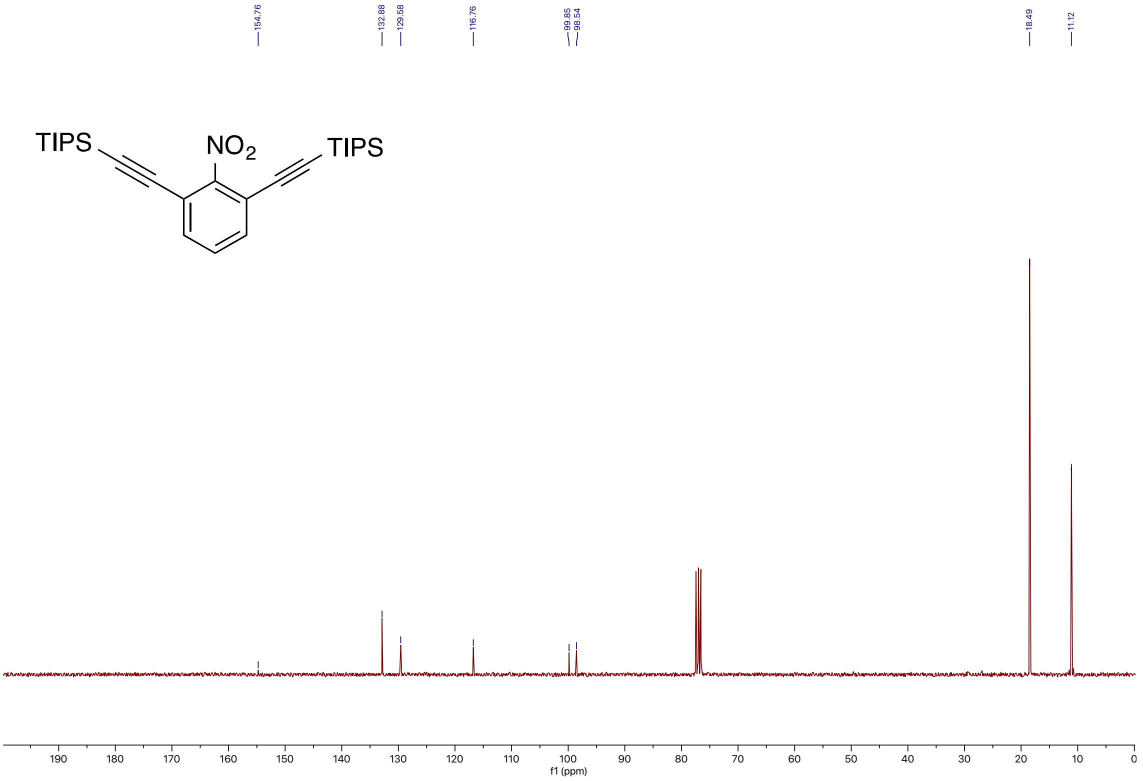

^{1}H NMR: 300 MHz, $CDCl_3$, compound **(3c)**

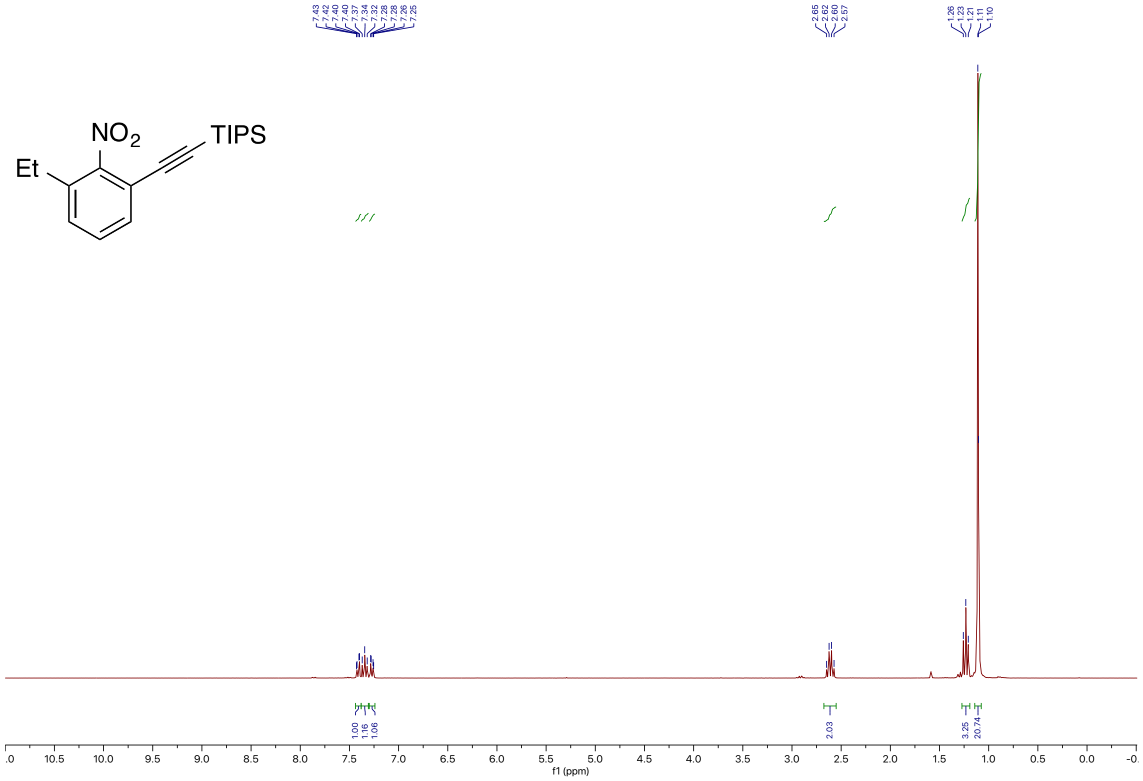

^{13}C NMR: 75 MHz, $CDCl_3$, compound **(3c)**

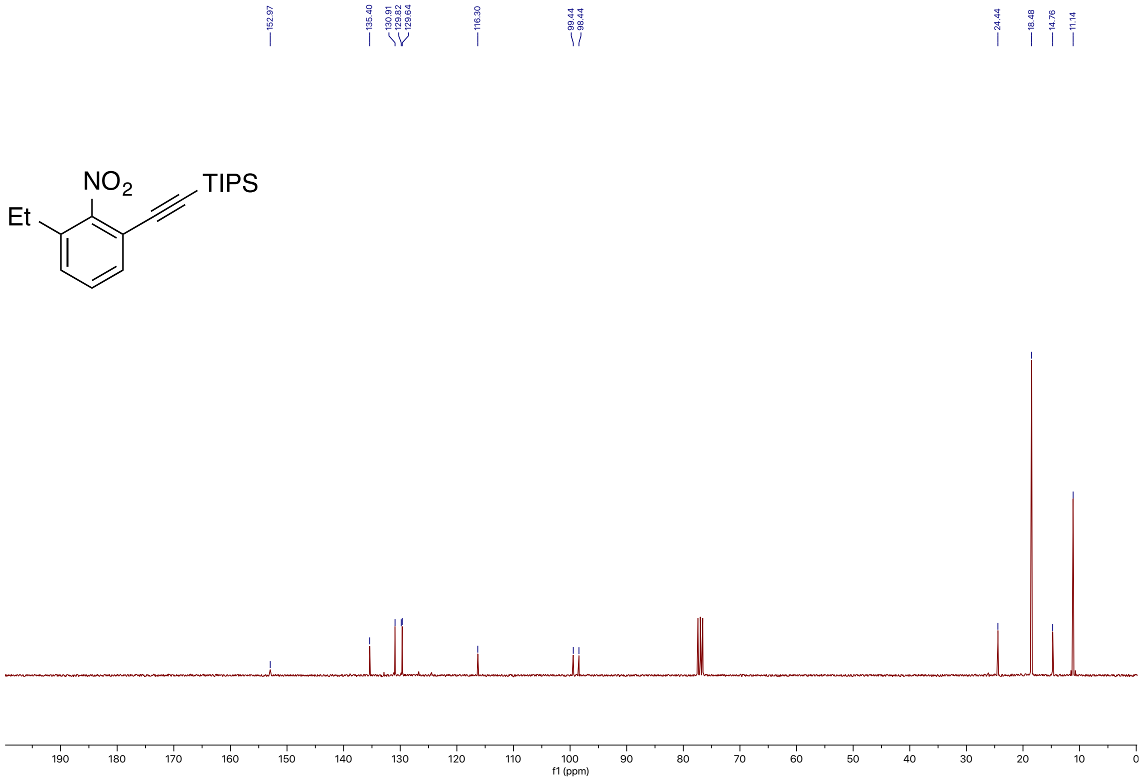

^{1}H NMR: 500 MHz, $CDCl_3$, compound **(3d)**

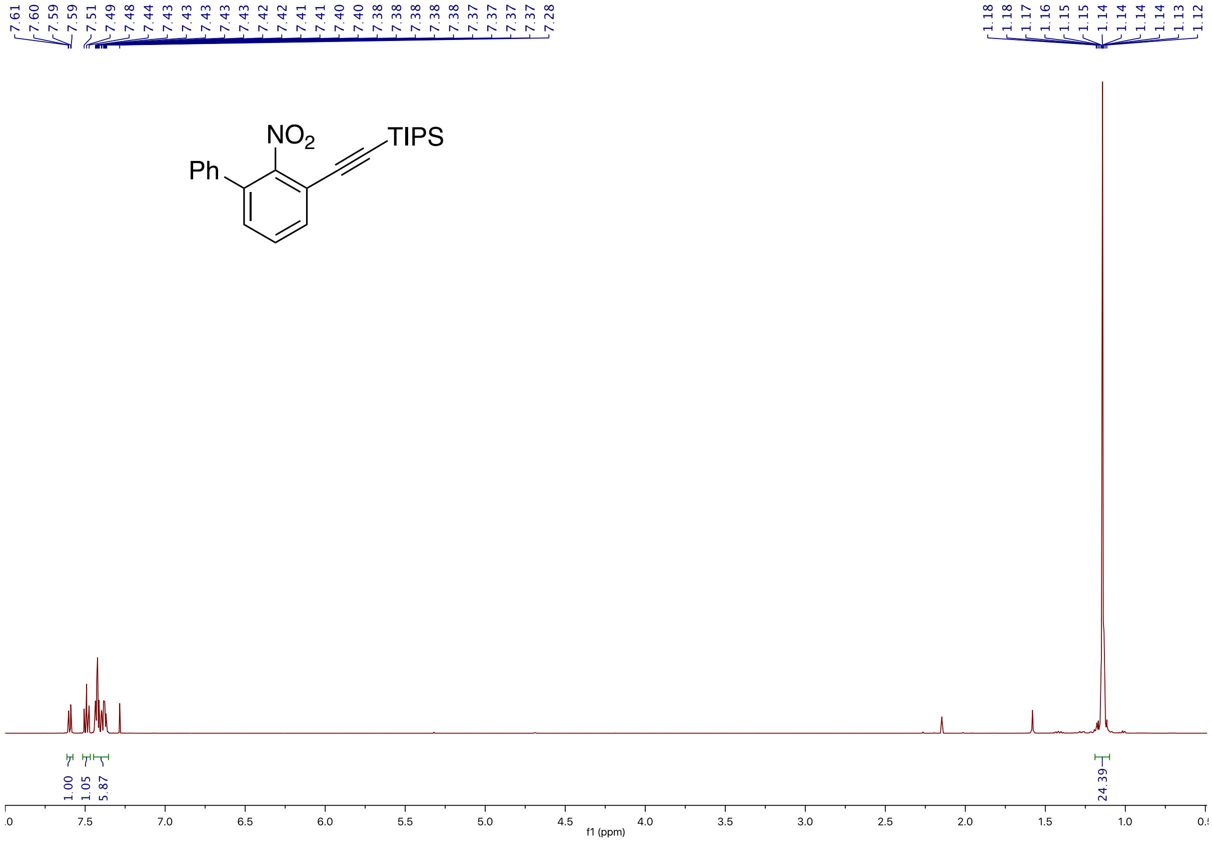

^{13}C NMR: 126 MHz, $CDCl_3$, compound **(3d)**

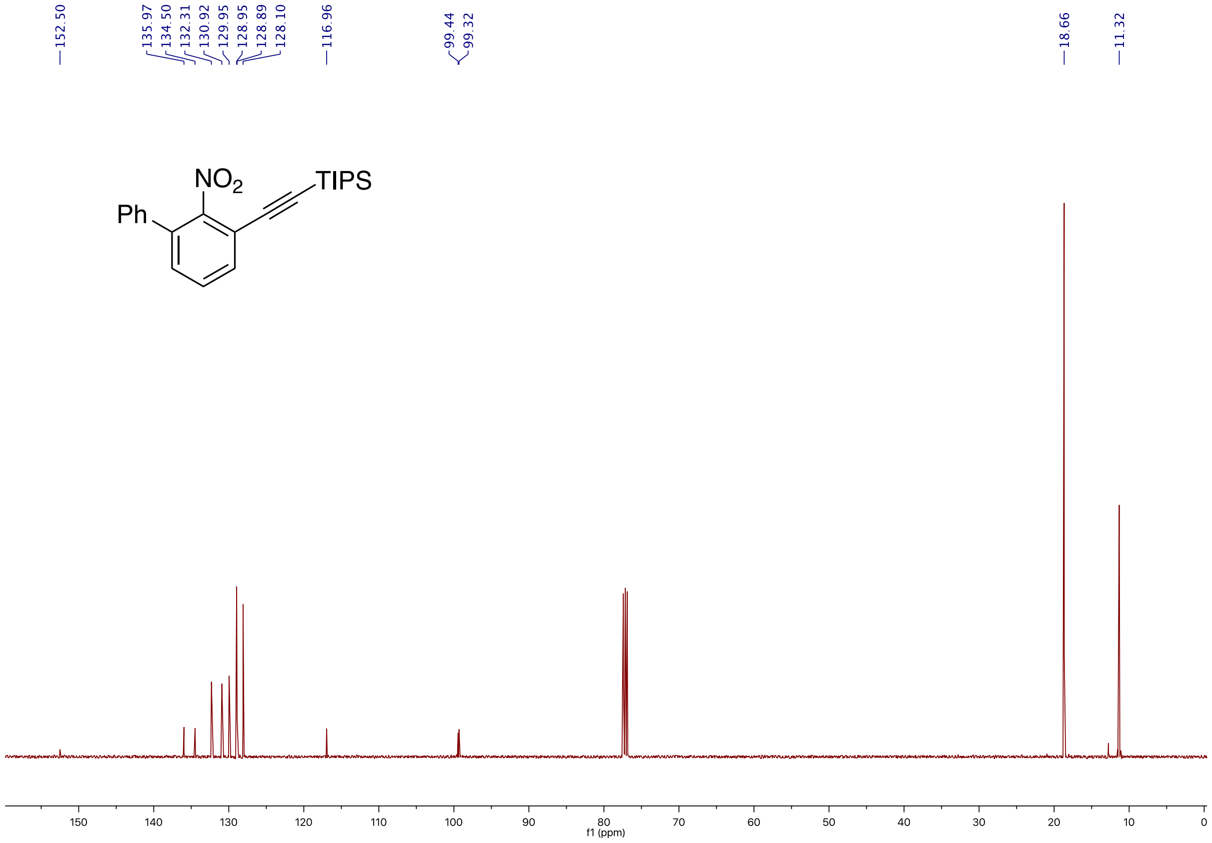

^{1}H NMR: 500 MHz, $CDCl_3$, compound **(3e)**

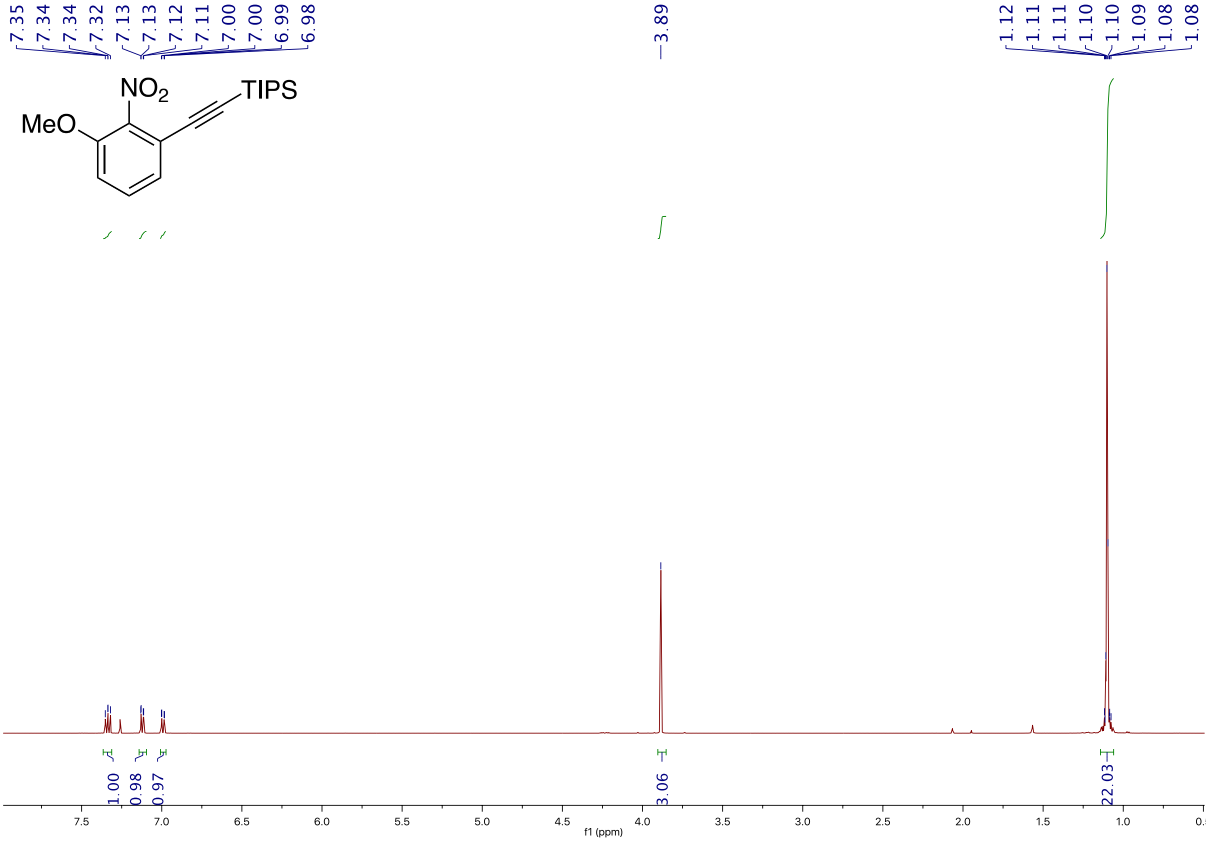

^{13}C NMR: 126 MHz, $CDCl_3$, compound **(3e)**

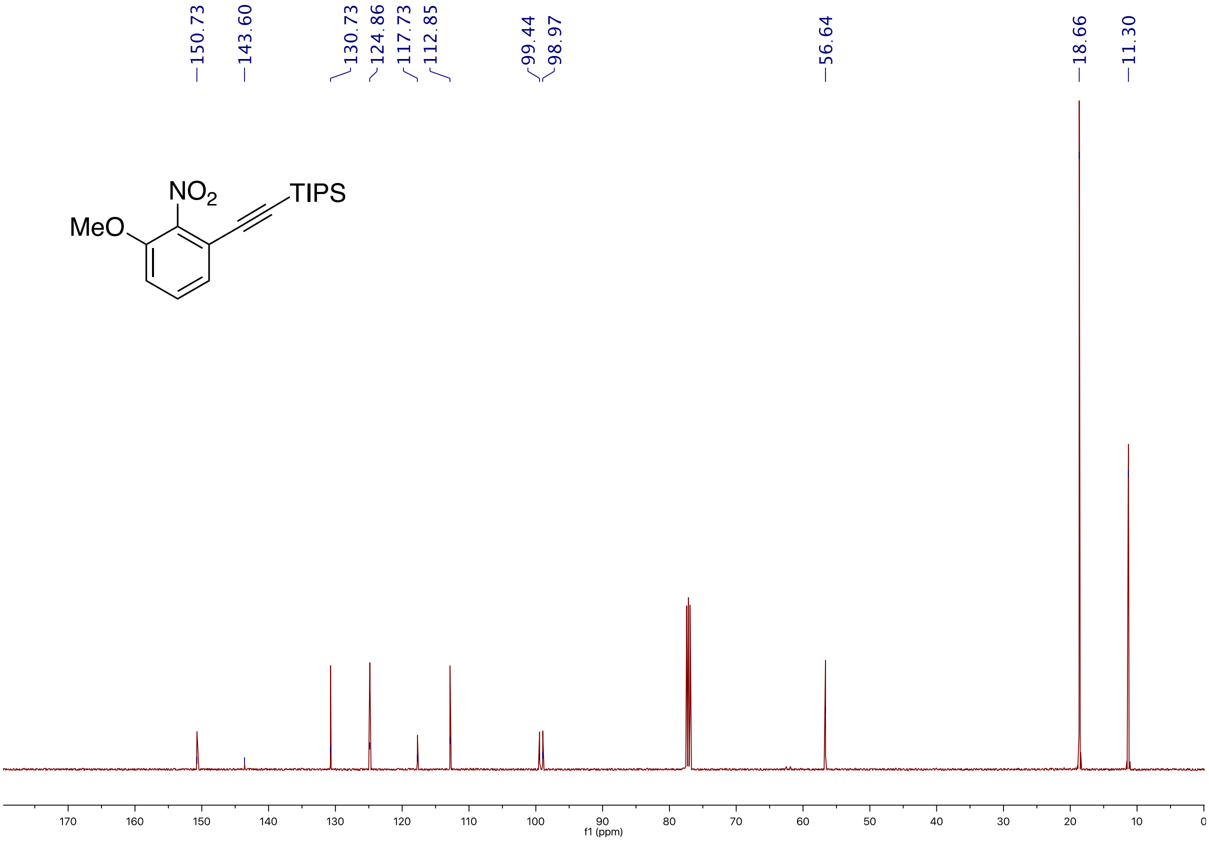

^{1}H NMR: 300 MHz, $CDCl_3$, compound **(3f)**

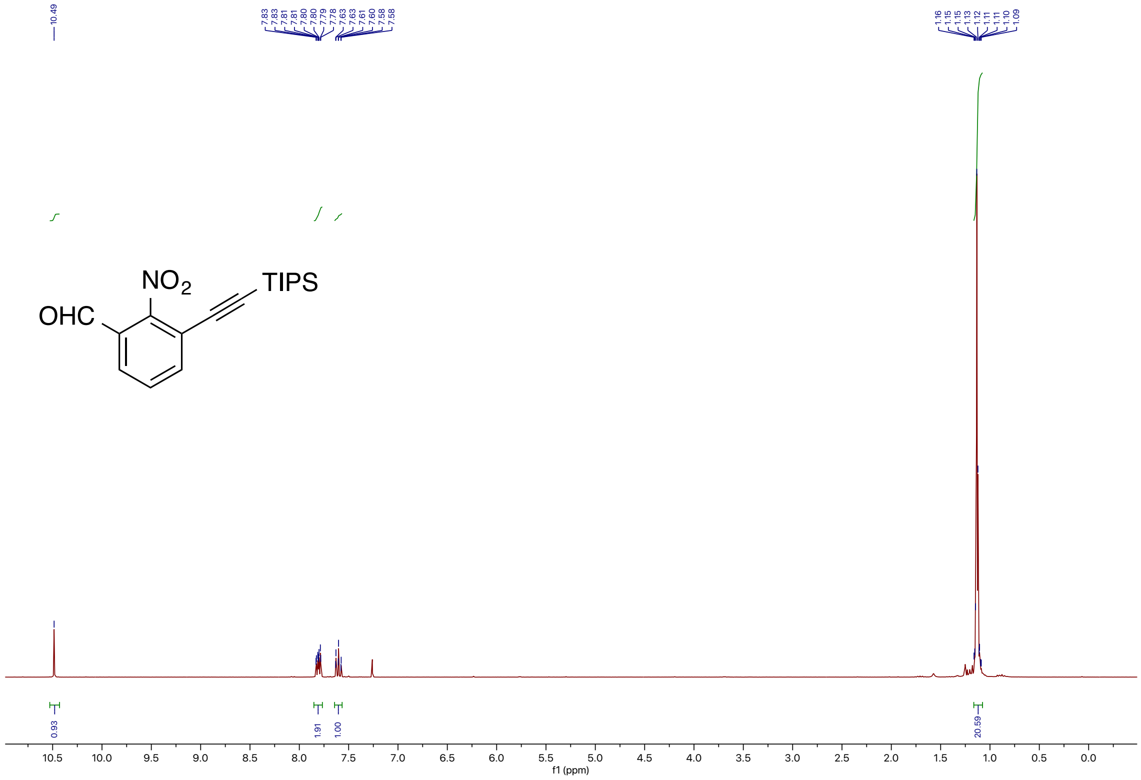

^{13}C NMR: 75 MHz, $CDCl_3$, compound **(3f)**

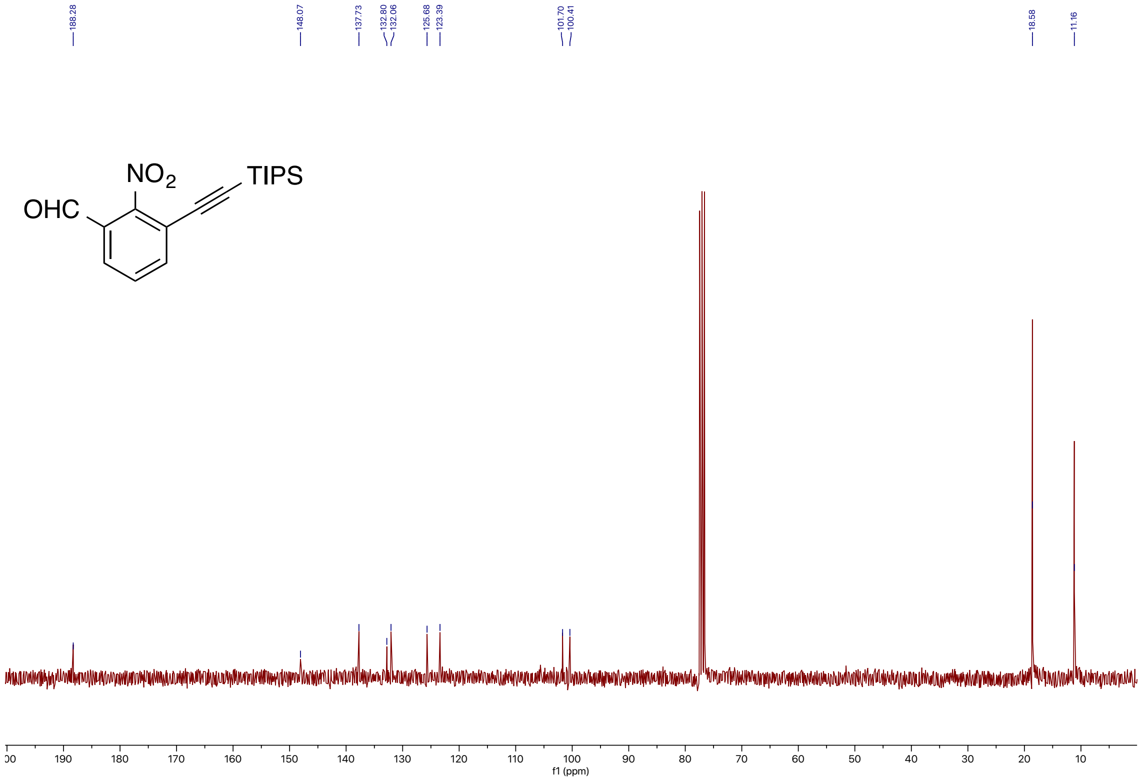

^{1}H NMR: 300 MHz, $CDCl_3$, compound **(3g)**

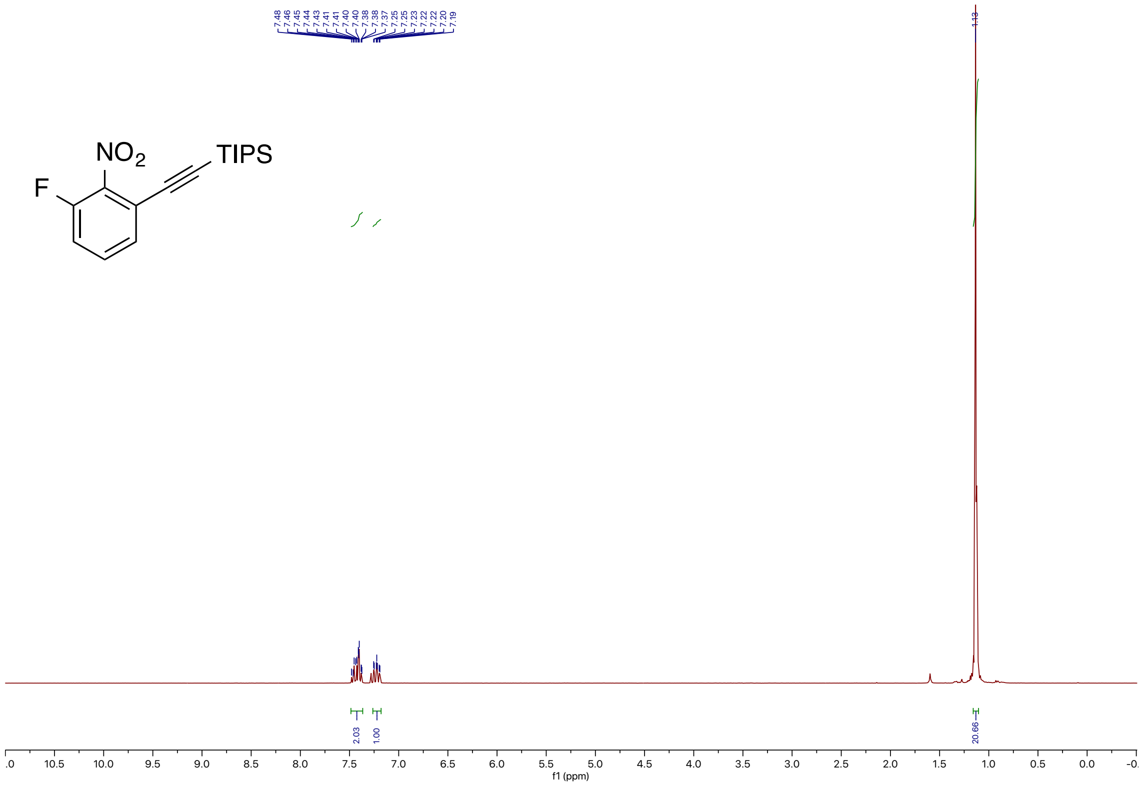

^{13}C NMR: 75 MHz, $CDCl_3$, compound **(3g)**

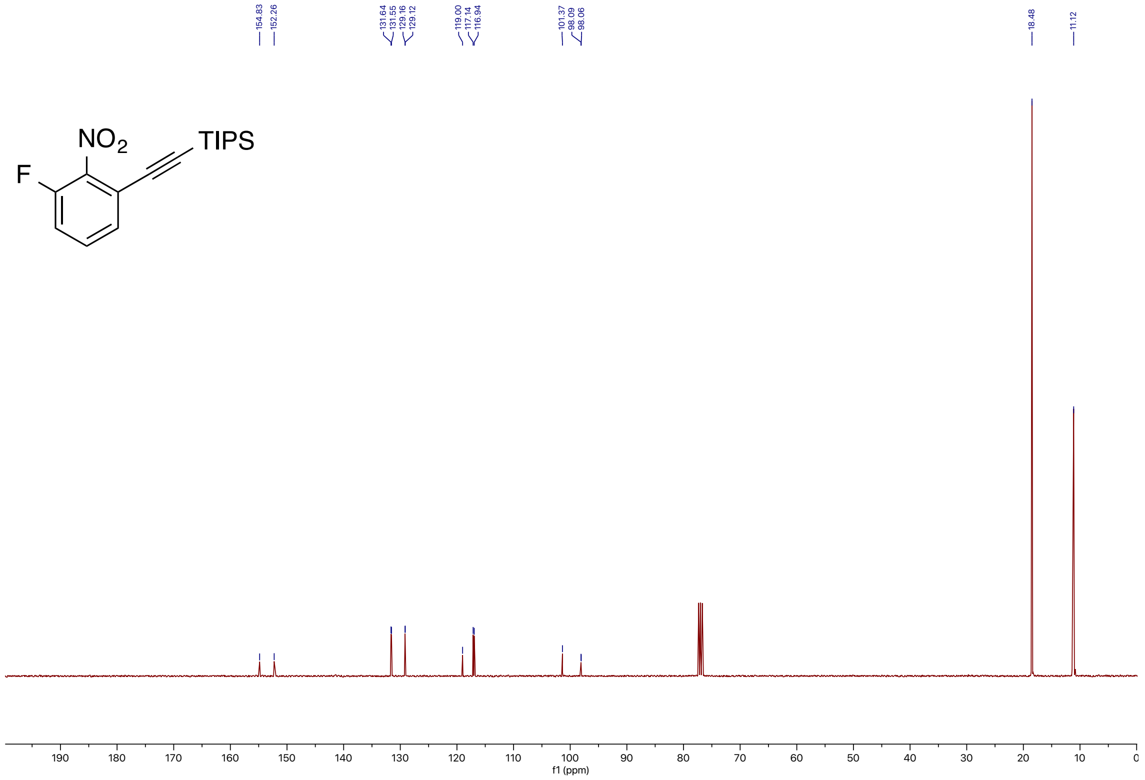

$^{19}F\{^1H\}$ NMR: 376 MHz, $CDCl_3$, compound **(3g)**

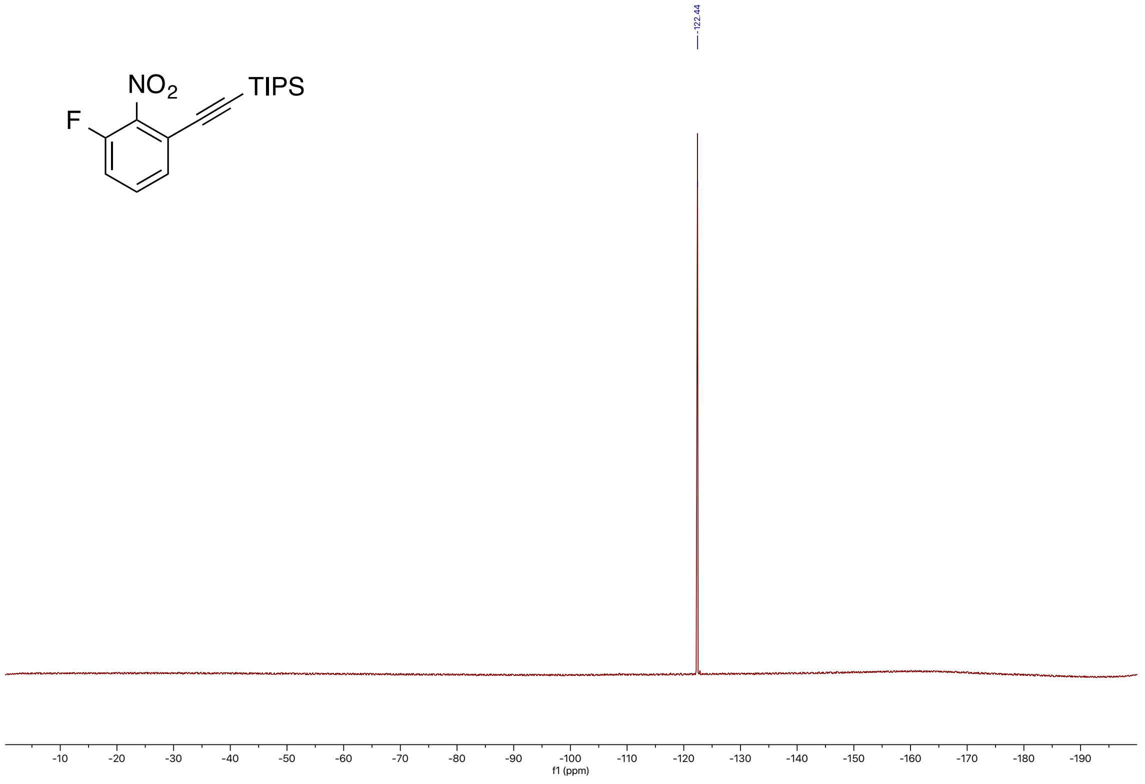

^{1}H NMR: 500 MHz, $CDCl_3$, compound (**3h**)

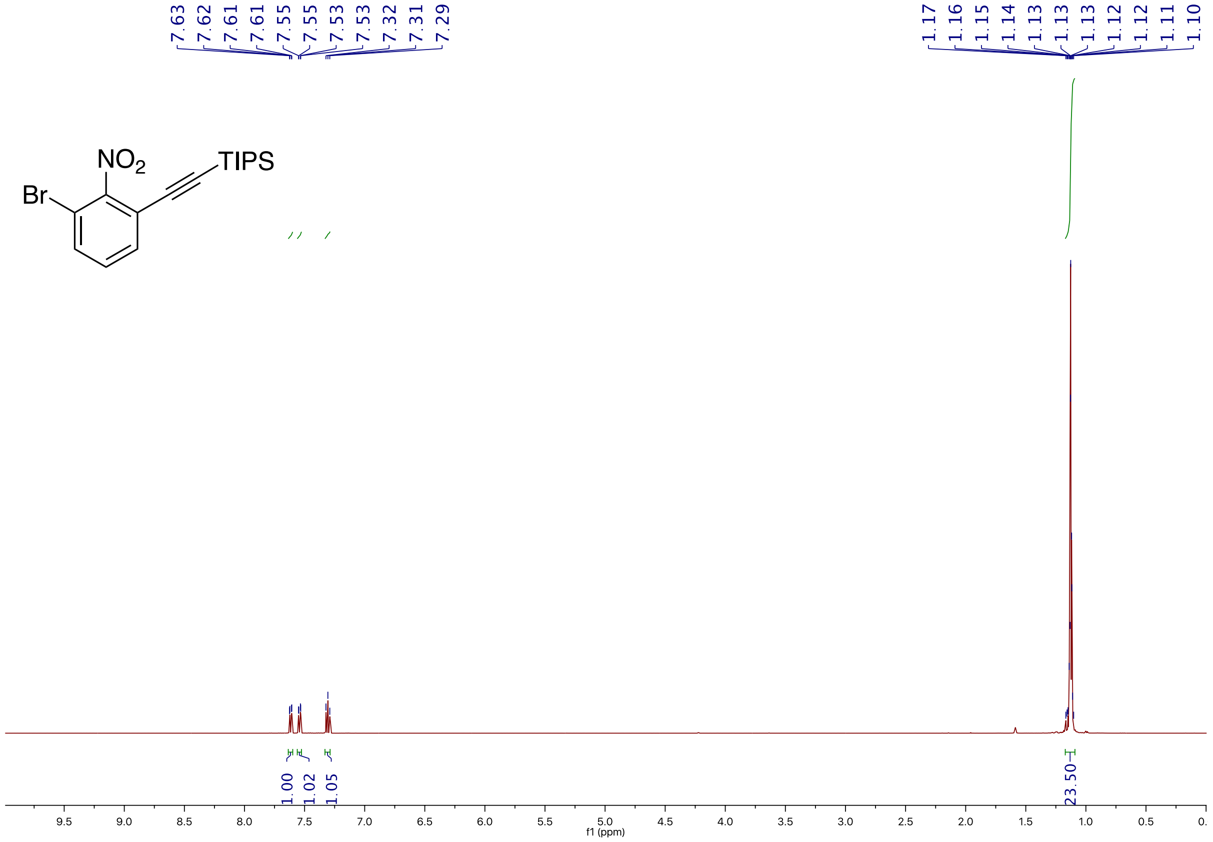

^{13}C NMR: 126 MHz, $CDCl_3$, compound (**3h**)

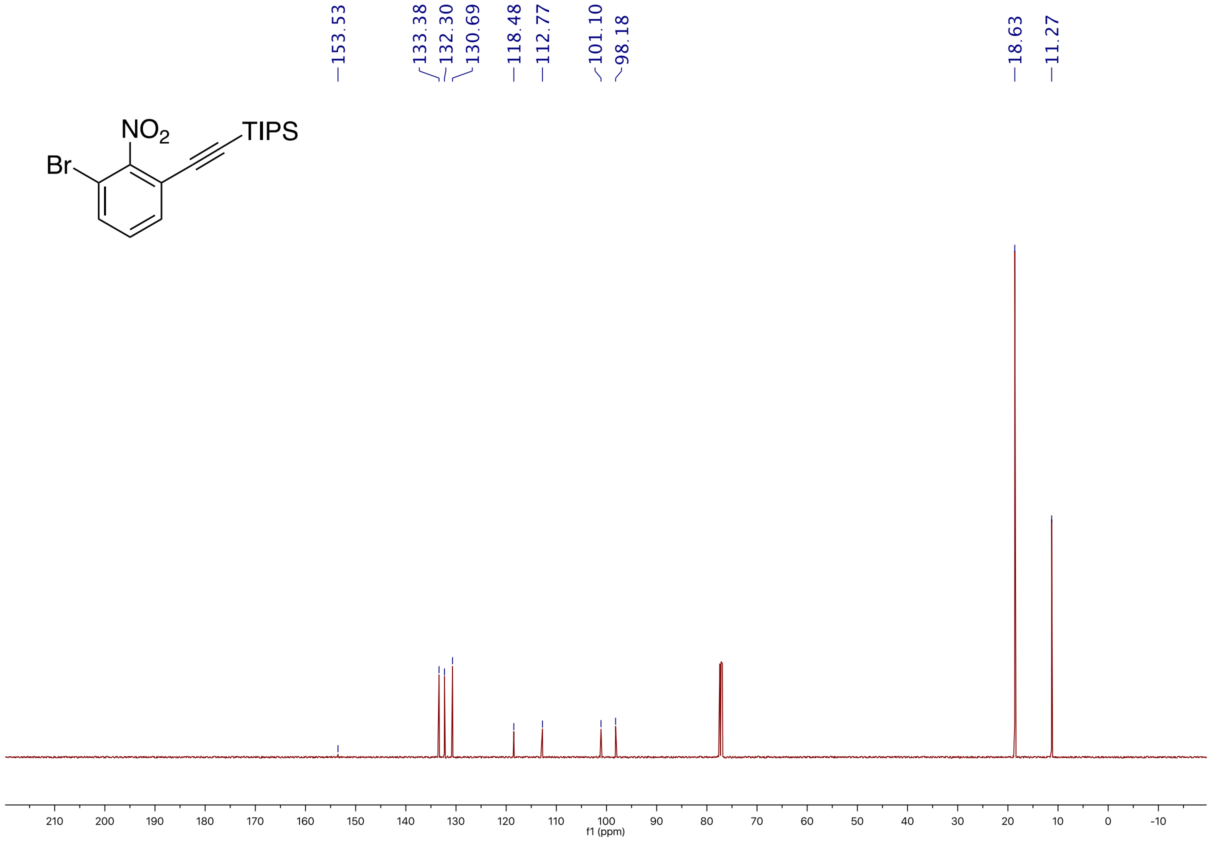

^{1}H NMR: 300 MHz, $CDCl_3$, compound **(3i)**

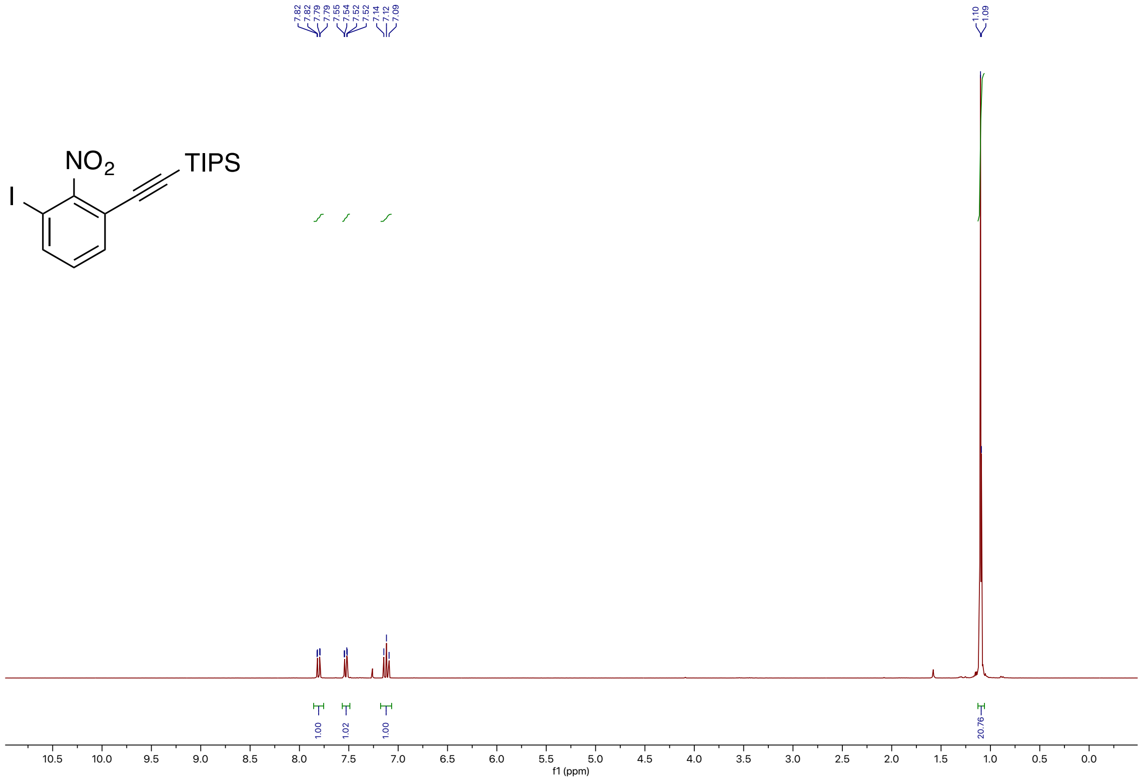

^{13}C NMR: 75 MHz, $CDCl_3$, compound **(3i)**

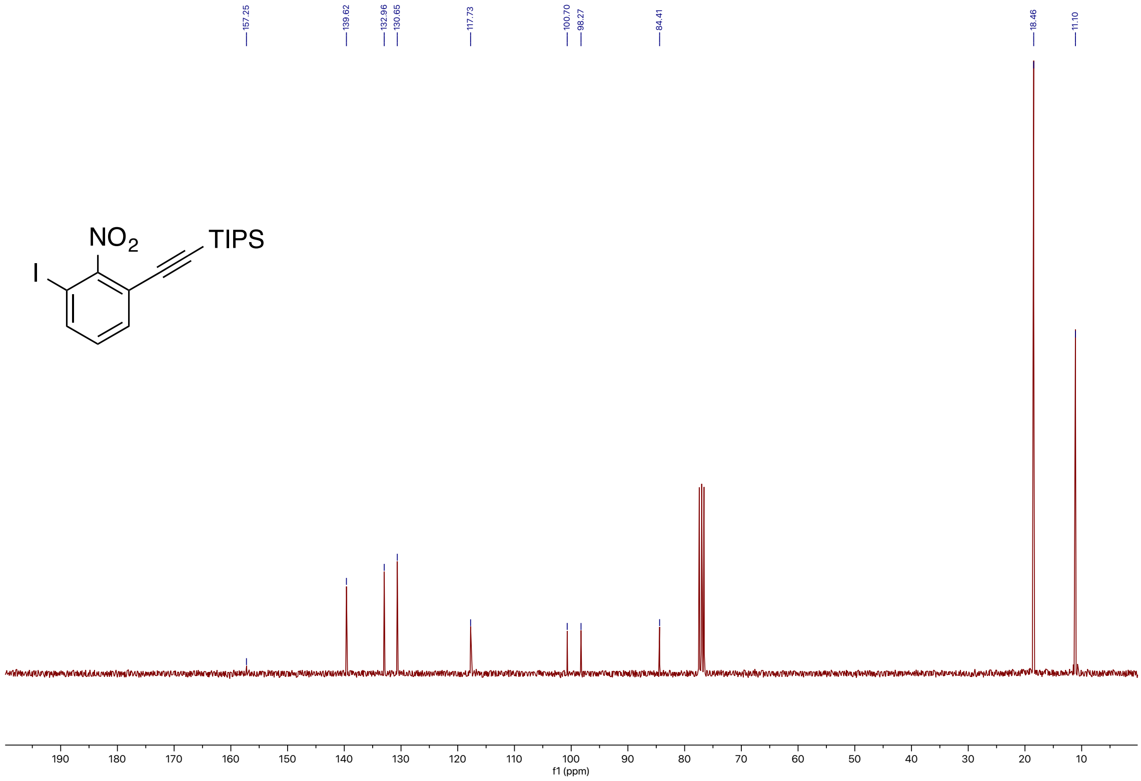

^{1}H NMR: 300 MHz, $CDCl_3$, compound **(3j)**

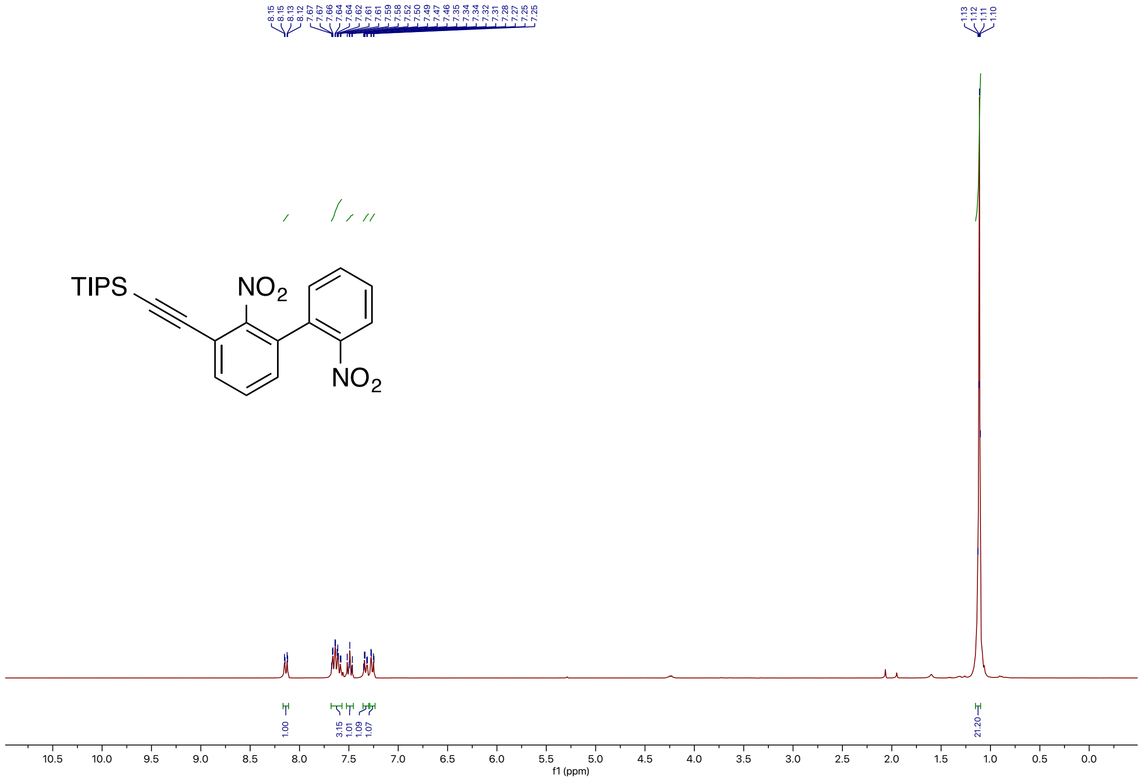

^{13}C NMR: 75 MHz, $CDCl_3$, compound **(3j)**

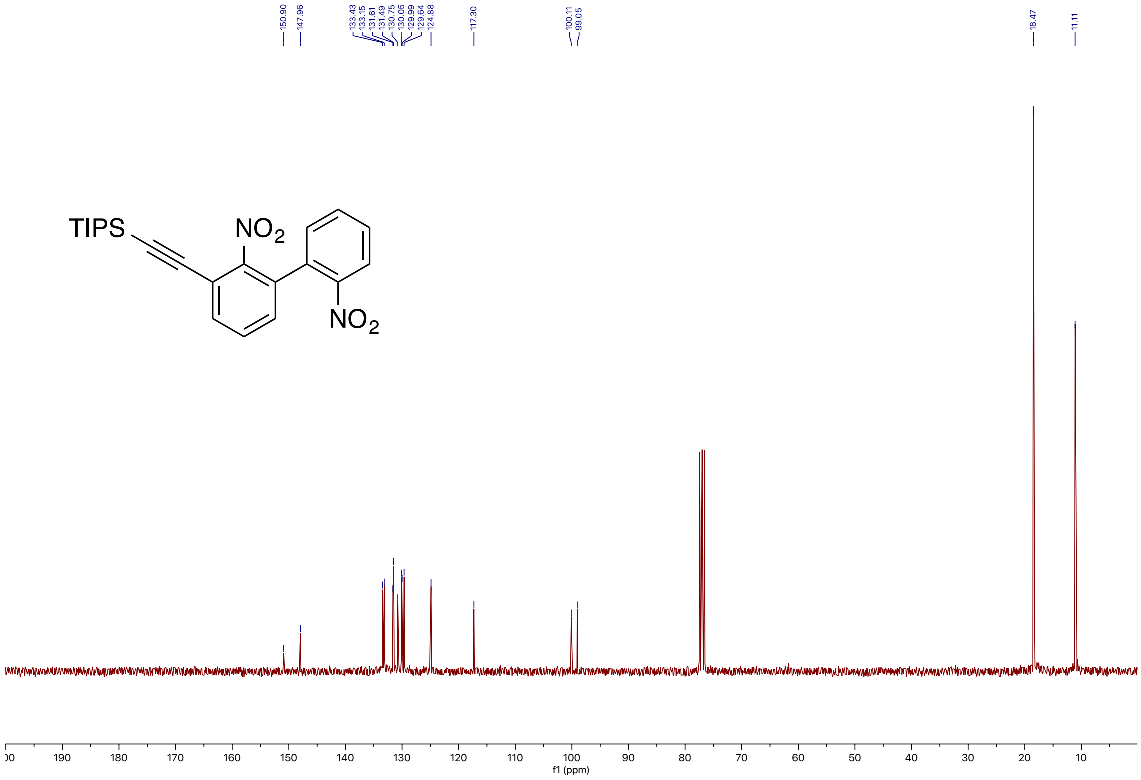

^{1}H NMR: 300 MHz, $CDCl_3$, compound **(3j')**

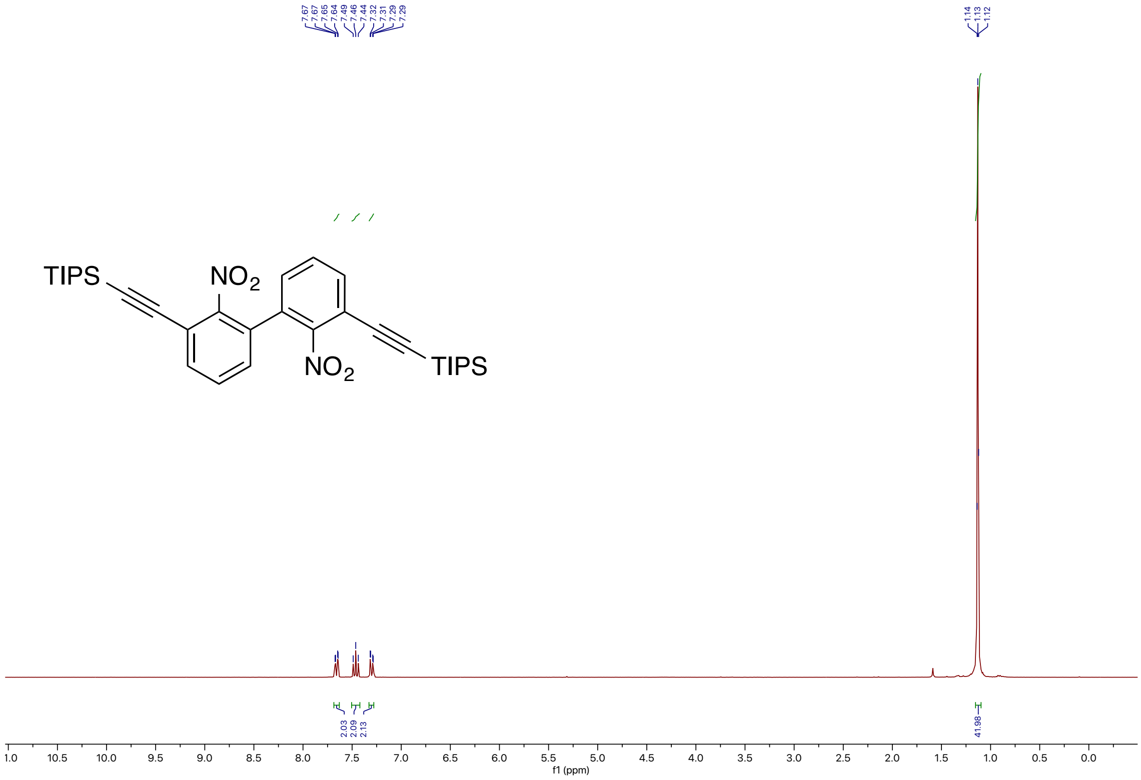

^{13}C NMR: 75 MHz, $CDCl_3$, compound **(3j')**

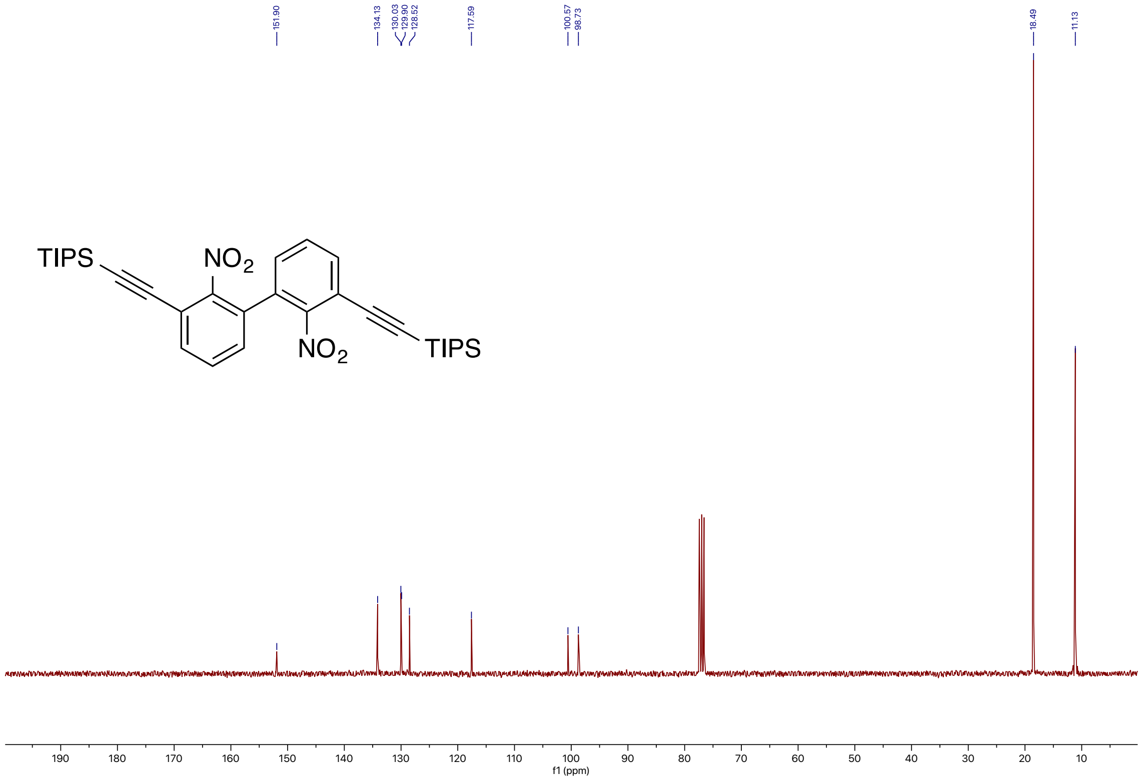

^{1}H NMR: 300 MHz, $CDCl_3$, compound **(3k)**

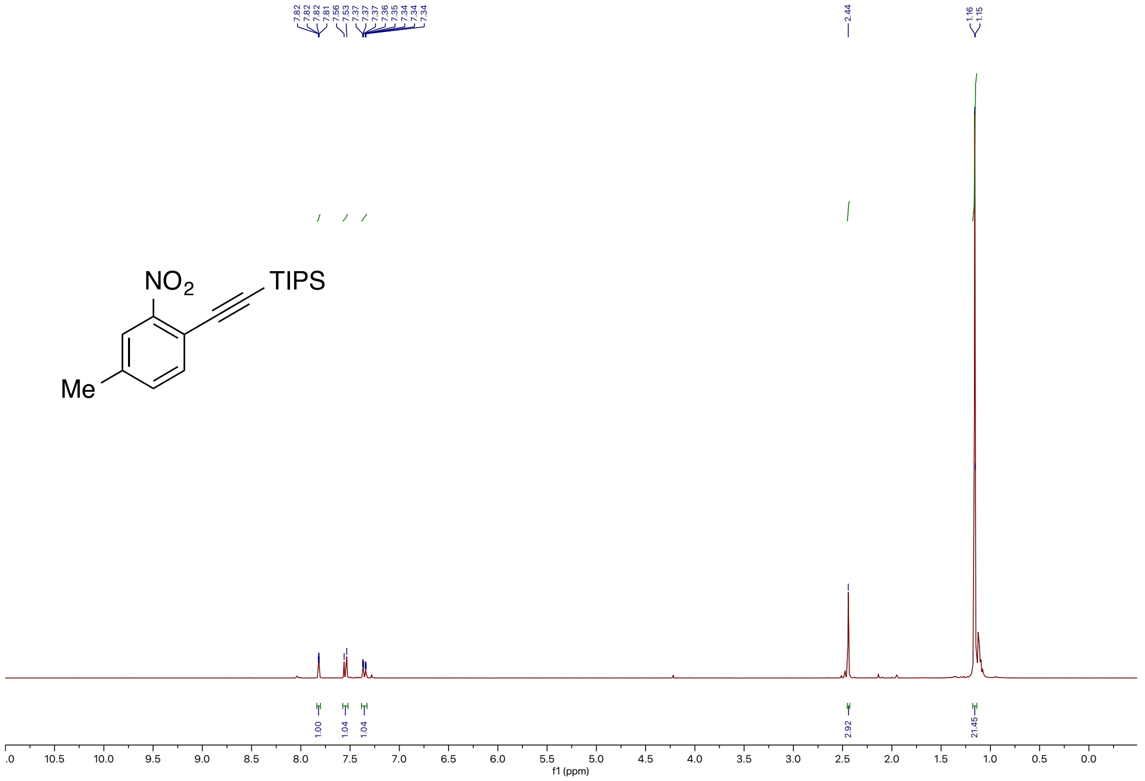

^{13}C NMR: 75 MHz, $CDCl_3$, compound **(3k)**

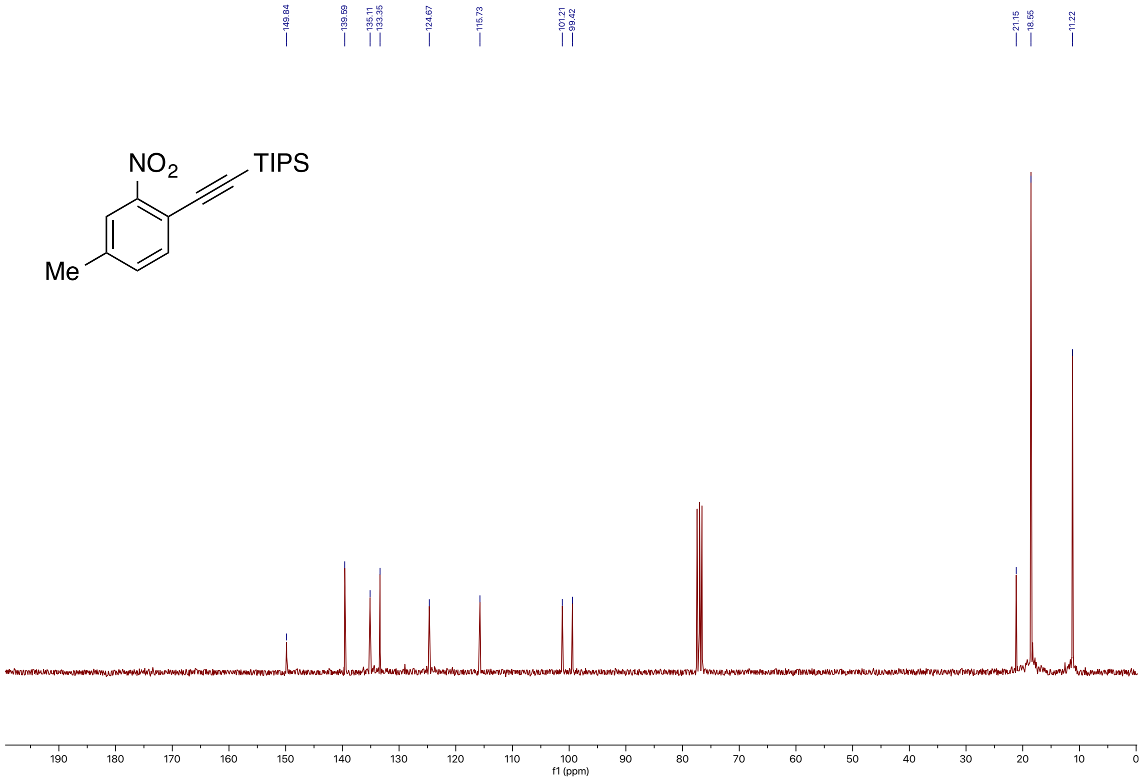

^{1}H NMR: 300 MHz, $CDCl_3$, compound **(3l)**

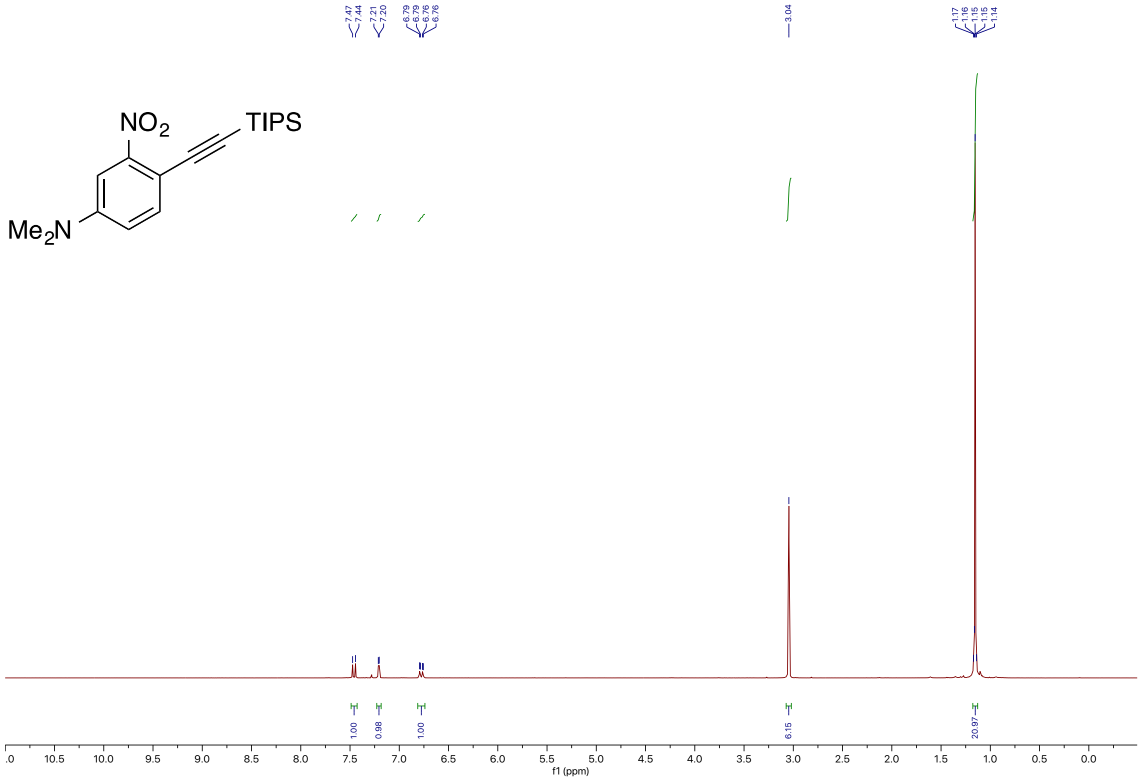

^{13}C NMR: 75 MHz, $CDCl_3$, compound **(3l)**

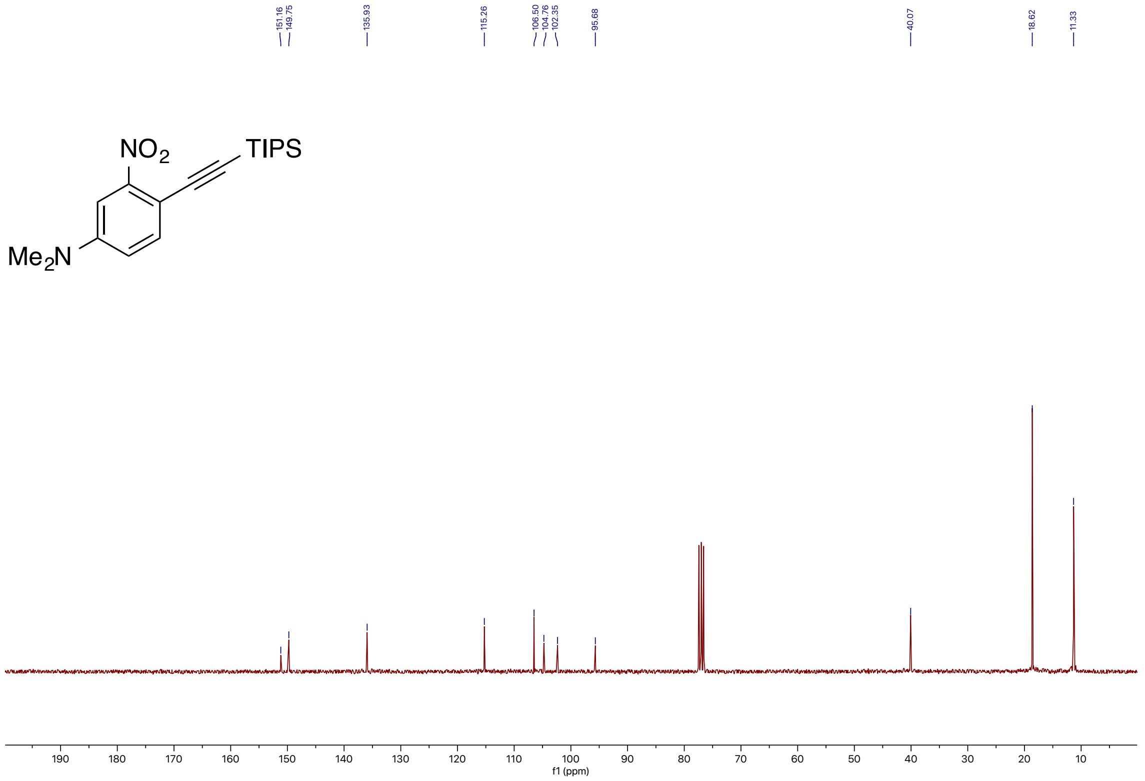

^{1}H NMR: 500 MHz, $CDCl_3$, compound (**3m**)

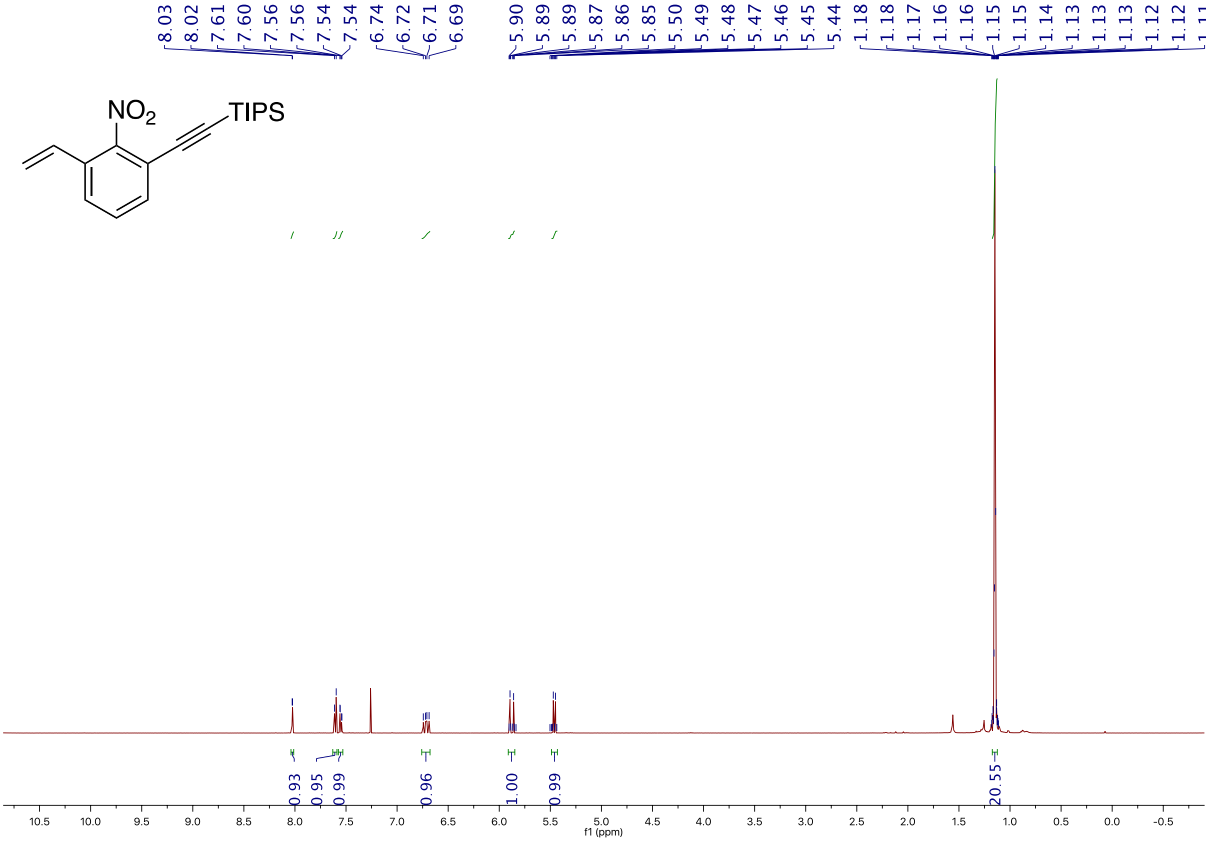

^{13}C NMR: 126 MHz, $CDCl_3$, compound (**3m**)

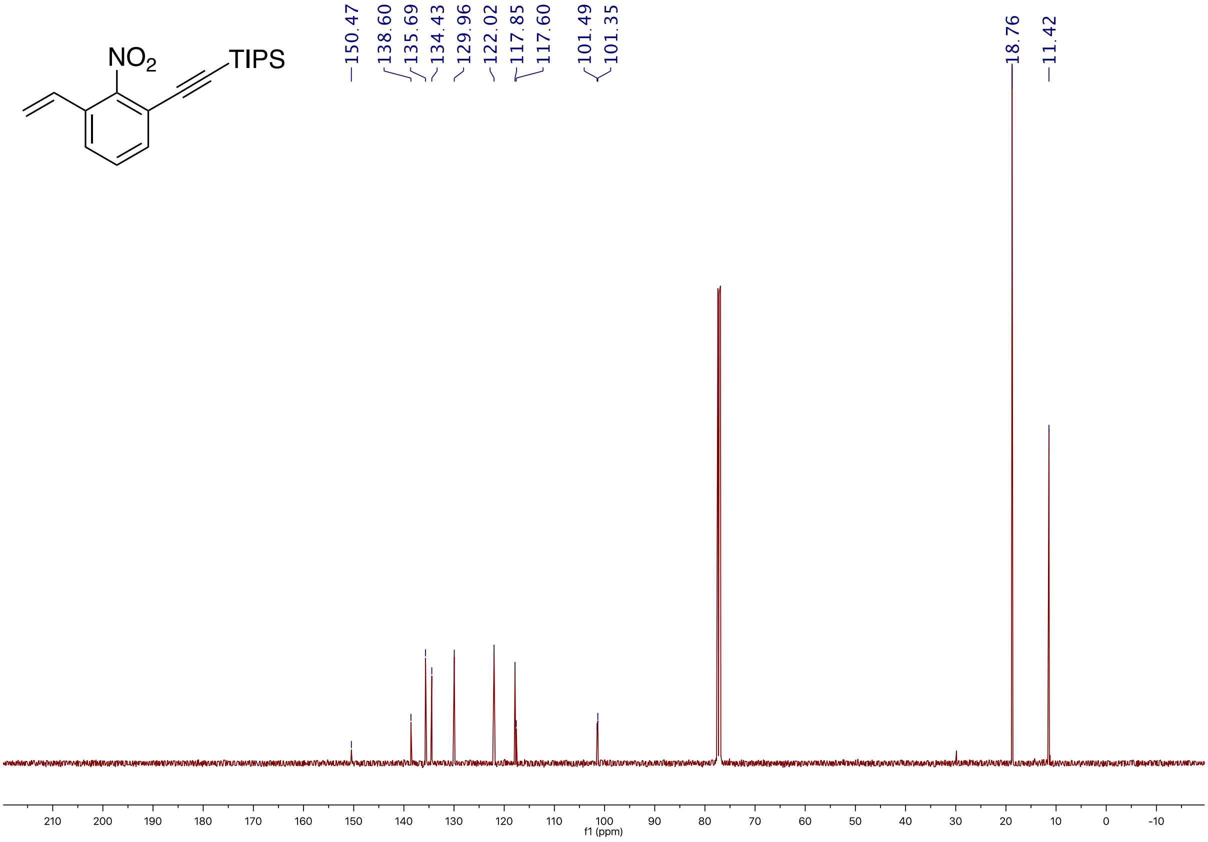

^{1}H NMR: 300 MHz, $CDCl_3$, compound **(3n)**

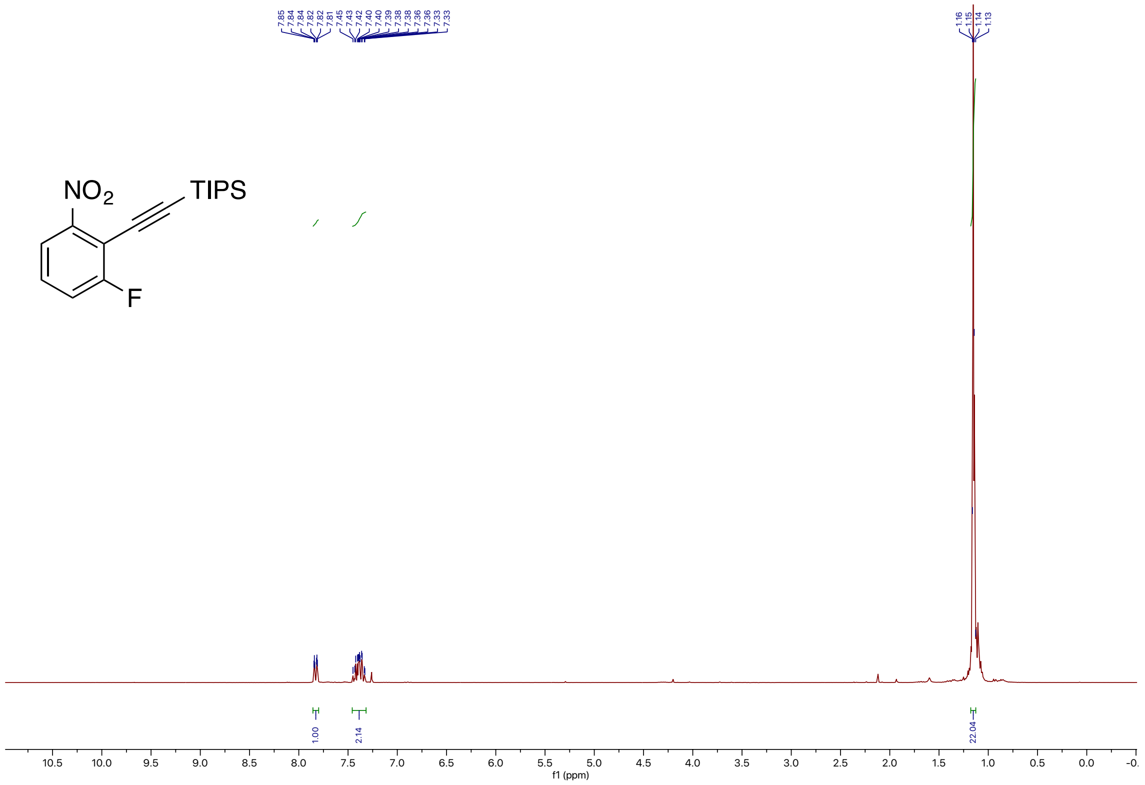

^{13}C NMR: 75 MHz, $CDCl_3$, compound **(3n)**

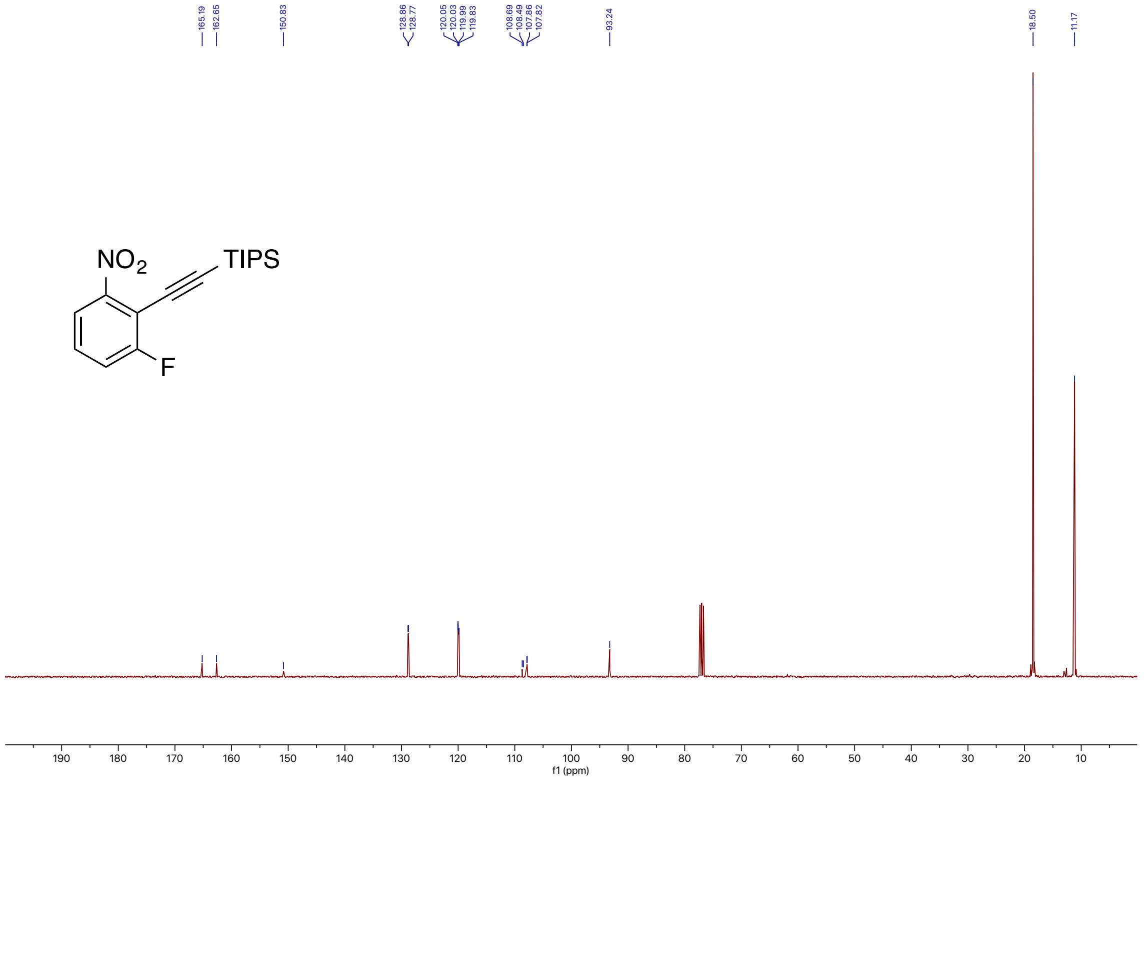

^{19}F NMR: 376 MHz, $CDCl_3$, compound **(3n)**

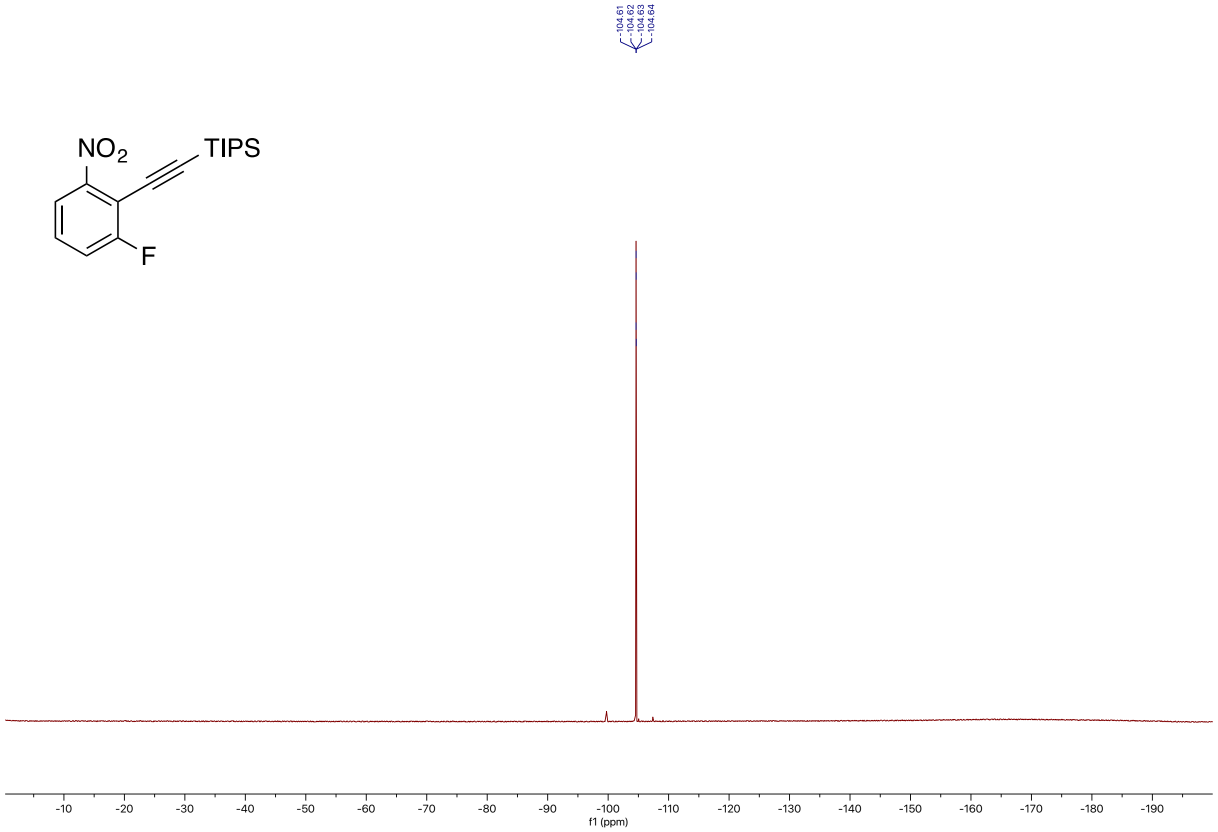

^{1}H NMR: 300 MHz, $CDCl_3$, compound **(3n')**

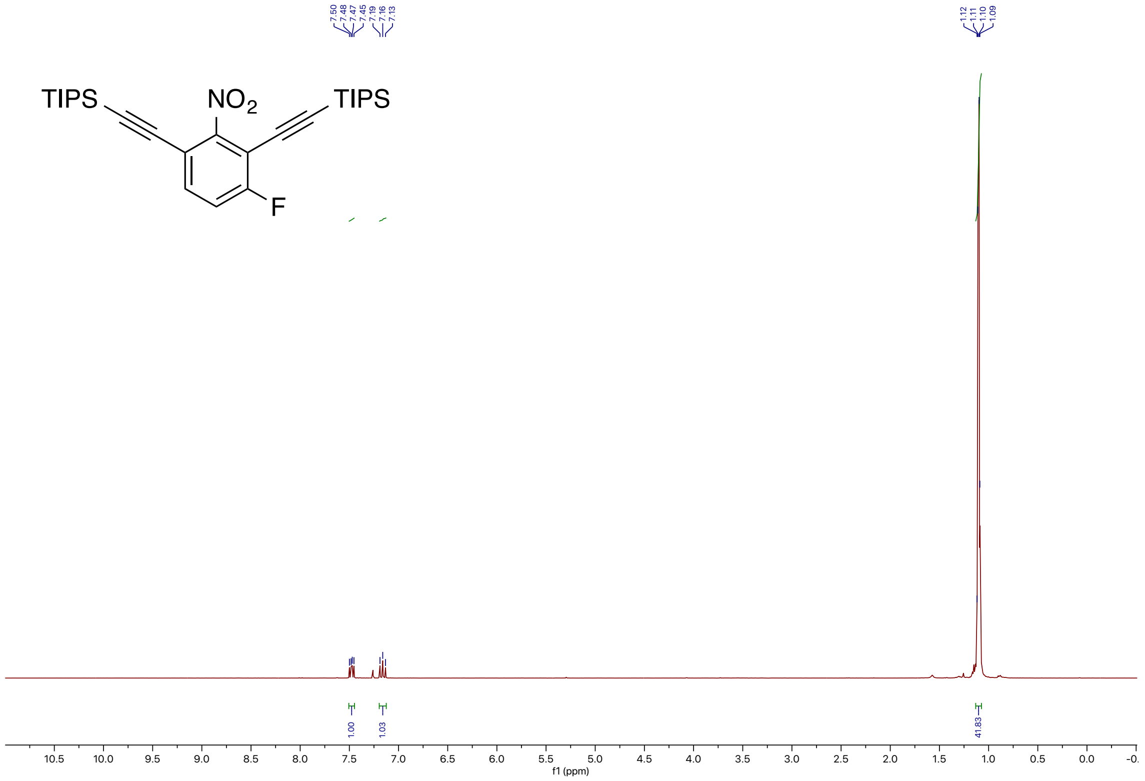

^{13}C NMR: 75 MHz, $CDCl_3$, compound **(3n')**

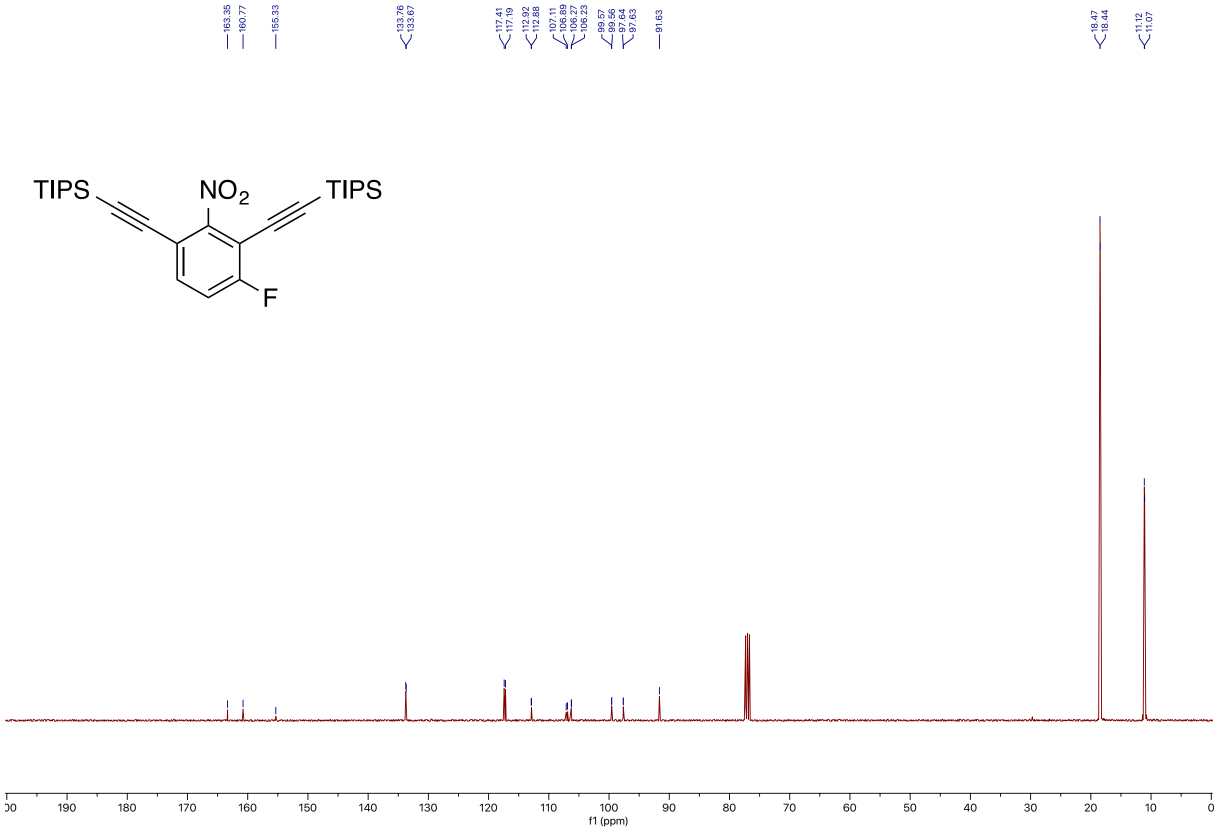

^{19}F NMR: 376 MHz, $CDCl_3$, compound **(3n')**

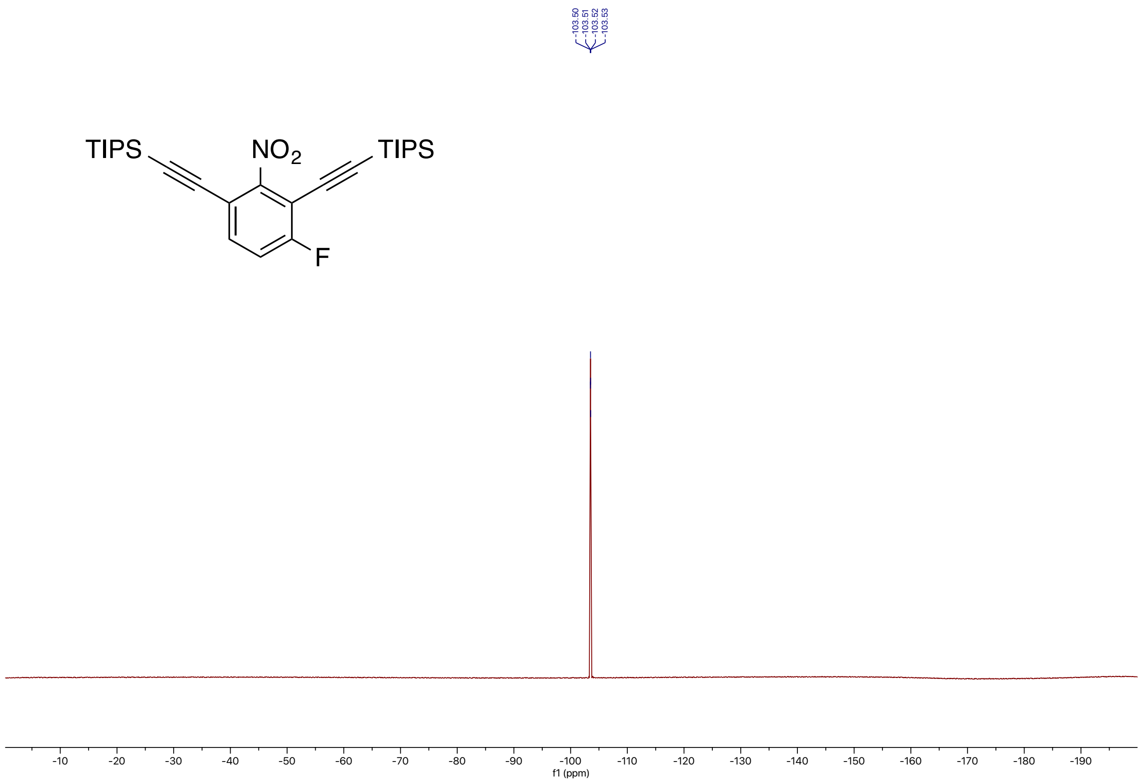
-103.50
-103.51
-103.52
-103.53
TIPS
NO2
TIPS
F
-10
-20
-30
-40
-50
-60
-70
-80
-90
-100
-110
-120
-130
-140
-150
-160
-170
-180
-190
f1 (ppm)

^{1}H NMR: 300 MHz, $CDCl_3$, compound **(3o)**

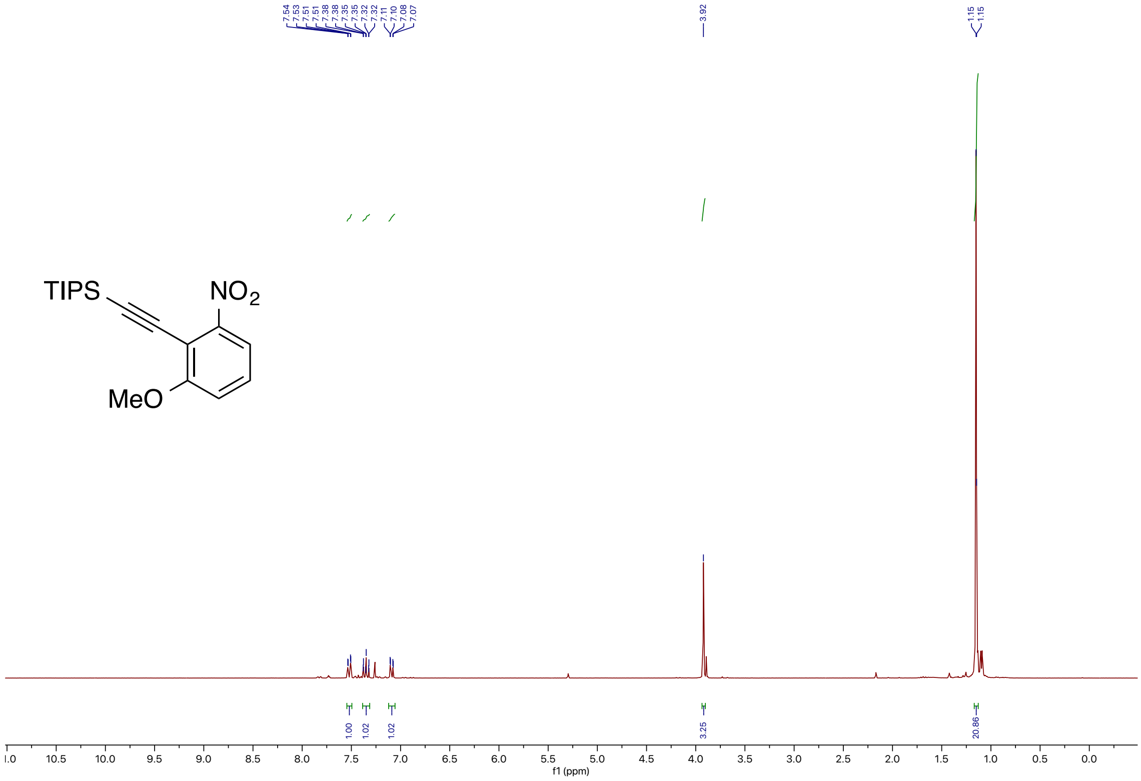

^{13}C NMR: 75 MHz, $CDCl_3$, compound **(3o)**

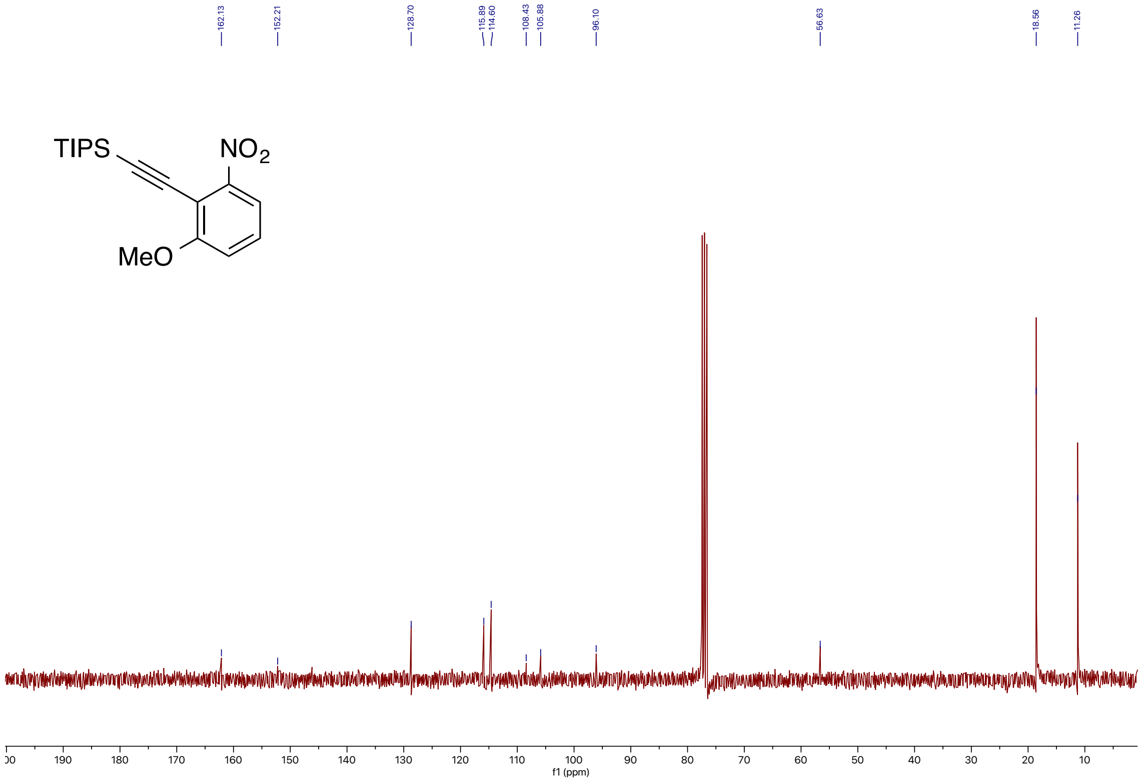

^{1}H NMR: 300 MHz, $CDCl_3$, compound **(3o')**

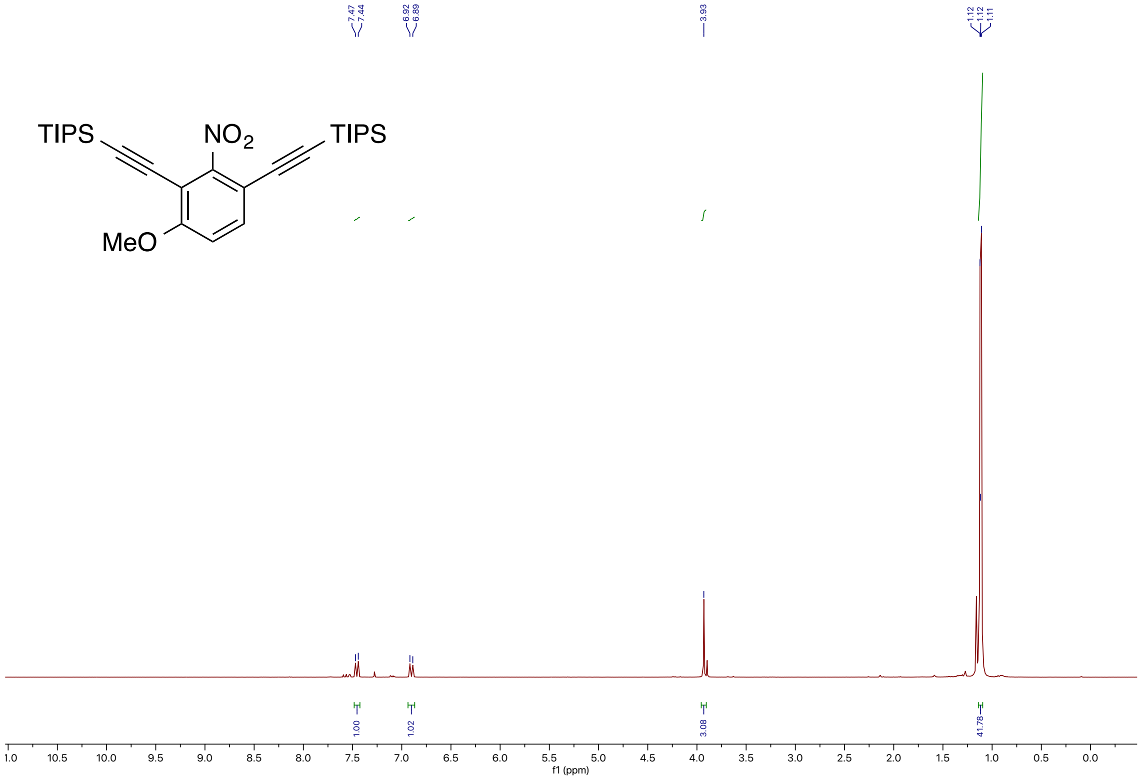

^{13}C NMR: 75 MHz, $CDCl_3$, compound **(3o')**

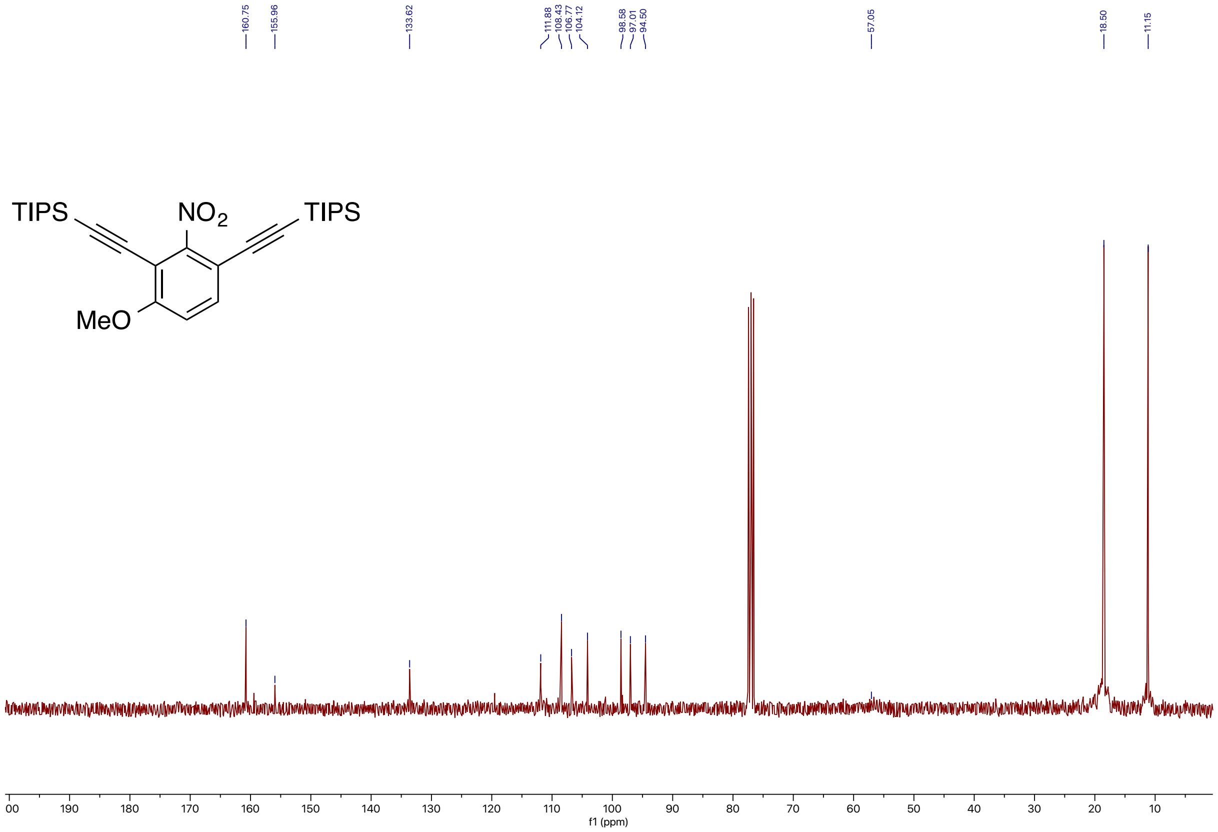

HSQC correlation: 300 MHz, $CDCl_3$, compound (**3o’**)

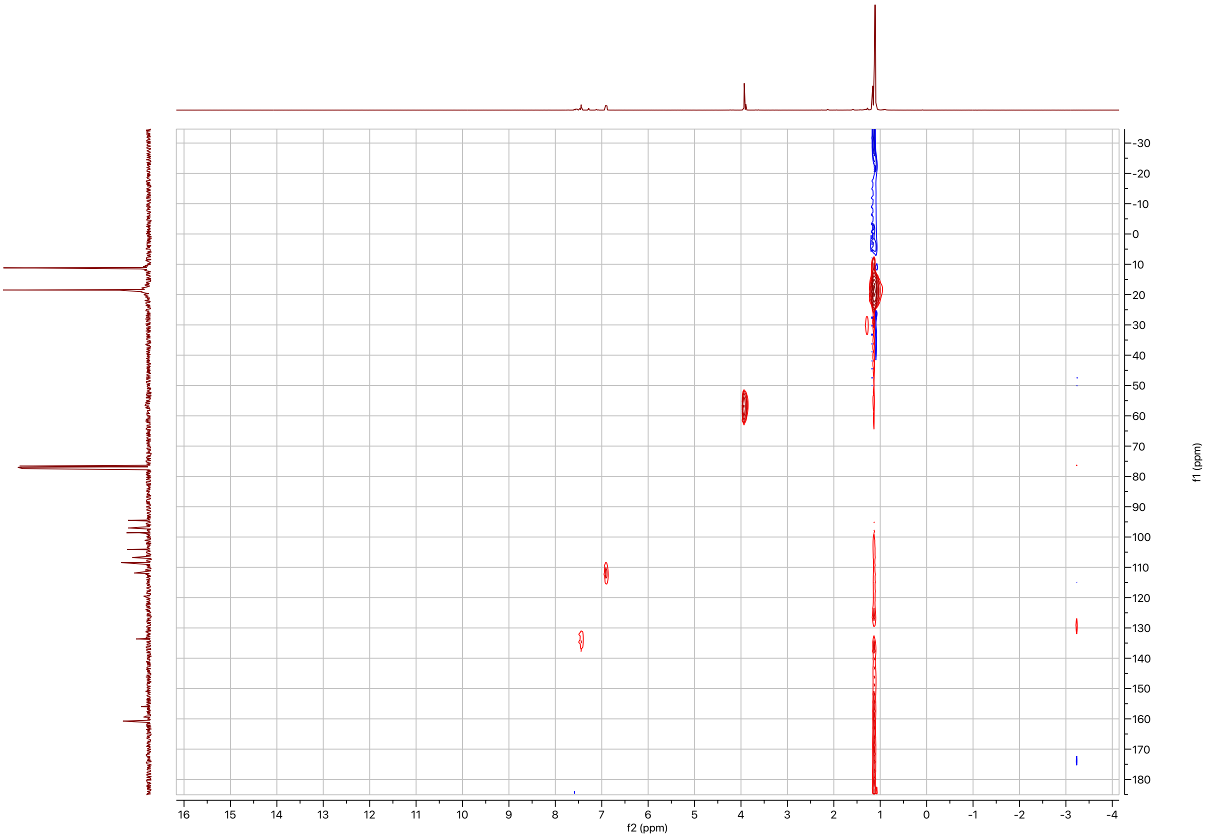

^{1}H NMR: 300 MHz, $CDCl_3$, compound **(3p)**

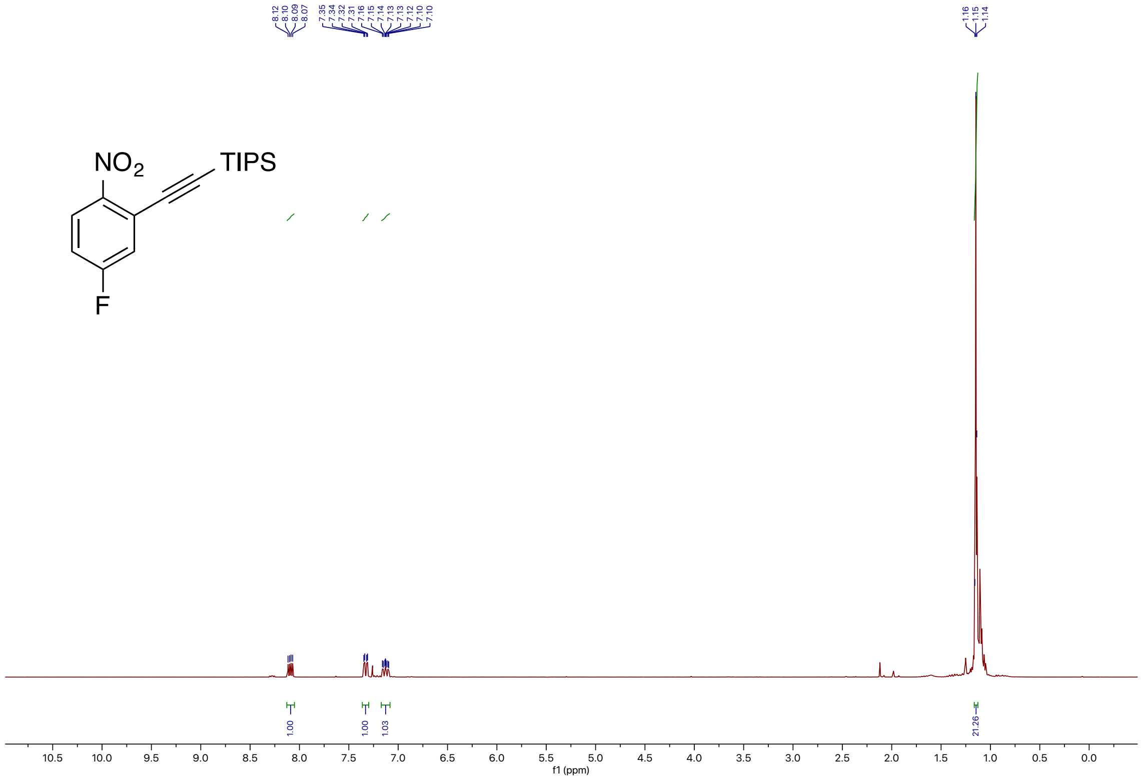

^{13}C NMR: 101 MHz, $CDCl_3$, compound (**3p**)

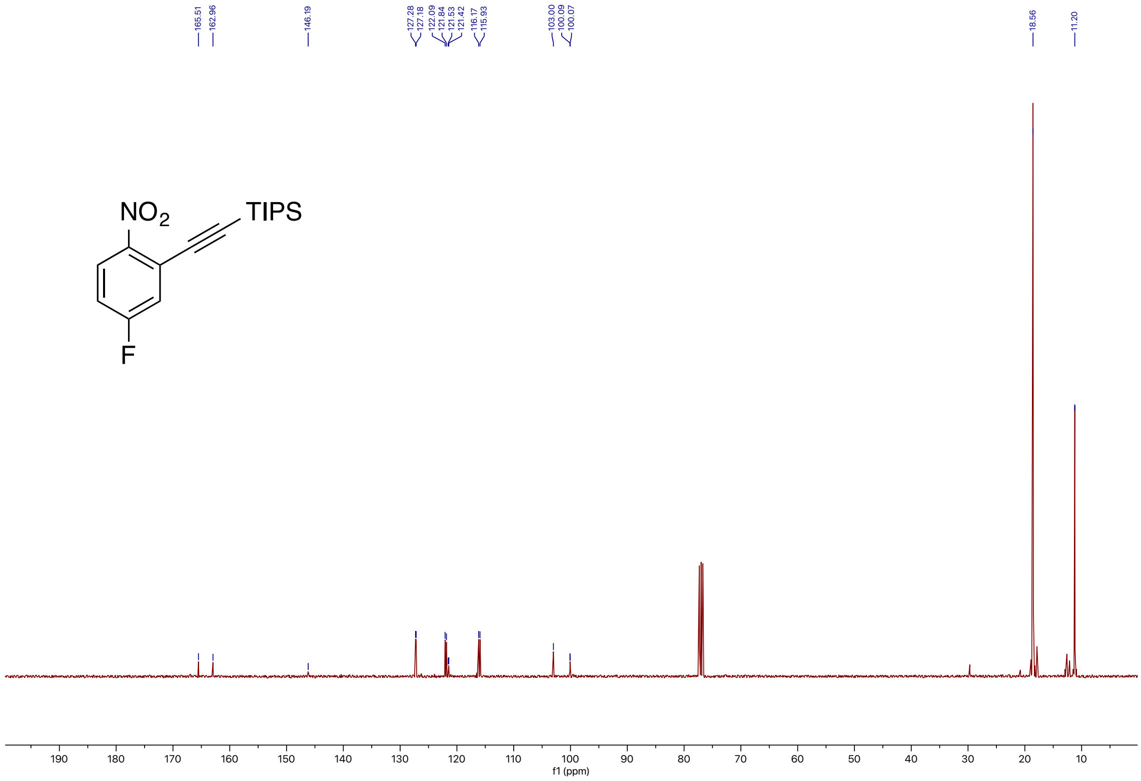

^{19}F NMR: 376 MHz, $CDCl_3$, compound **(3p)**

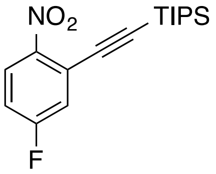

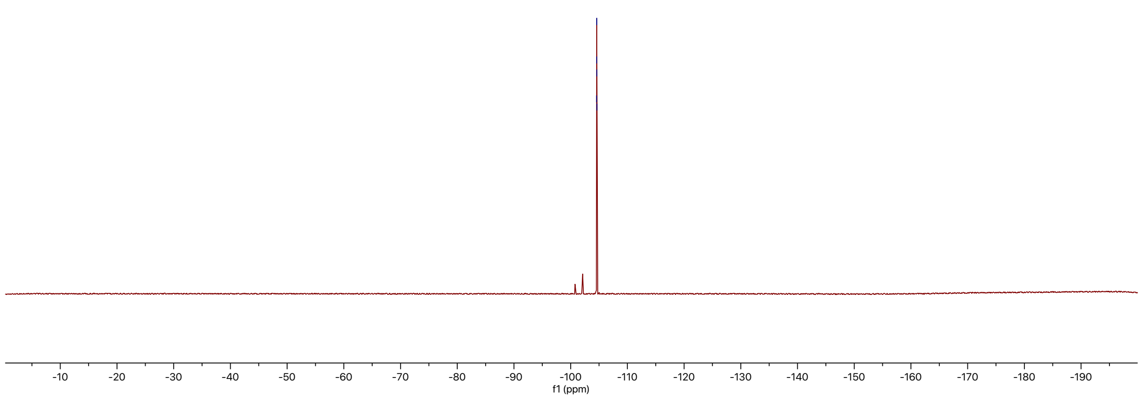

^{1}H NMR: 400 MHz, $CDCl_3$, compound **(3p')**

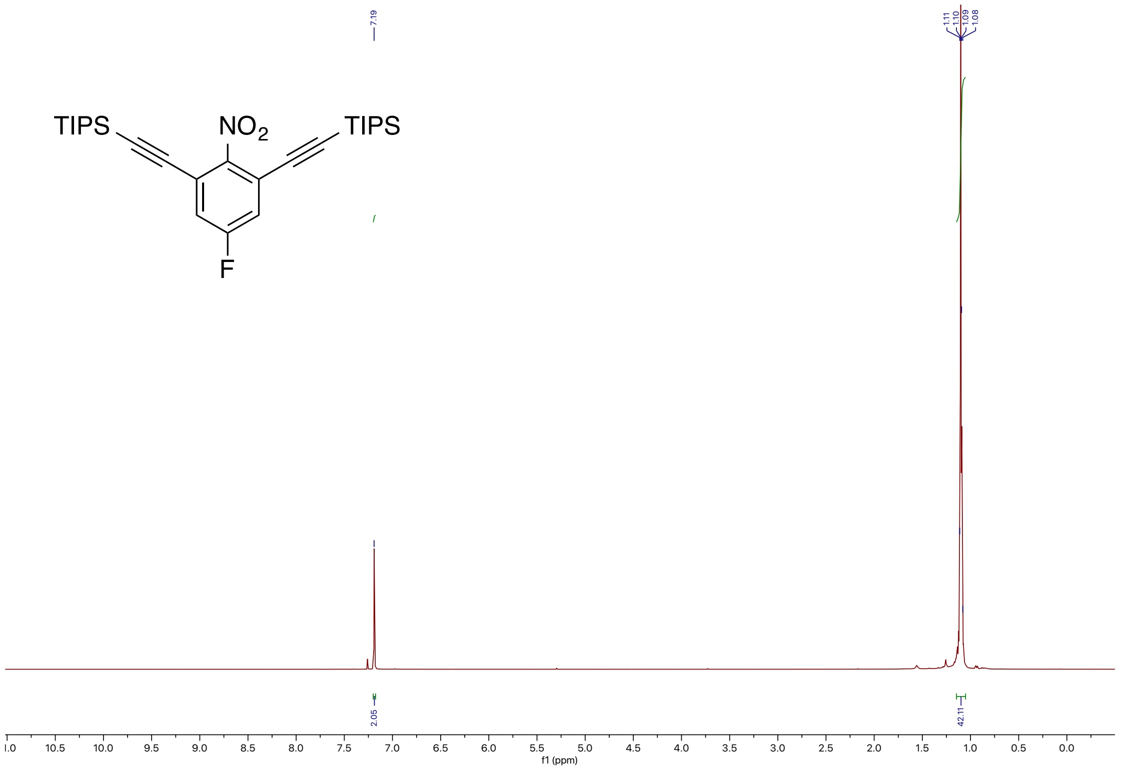

^{13}C NMR: 75 MHz, $CDCl_3$, compound **(3p')**

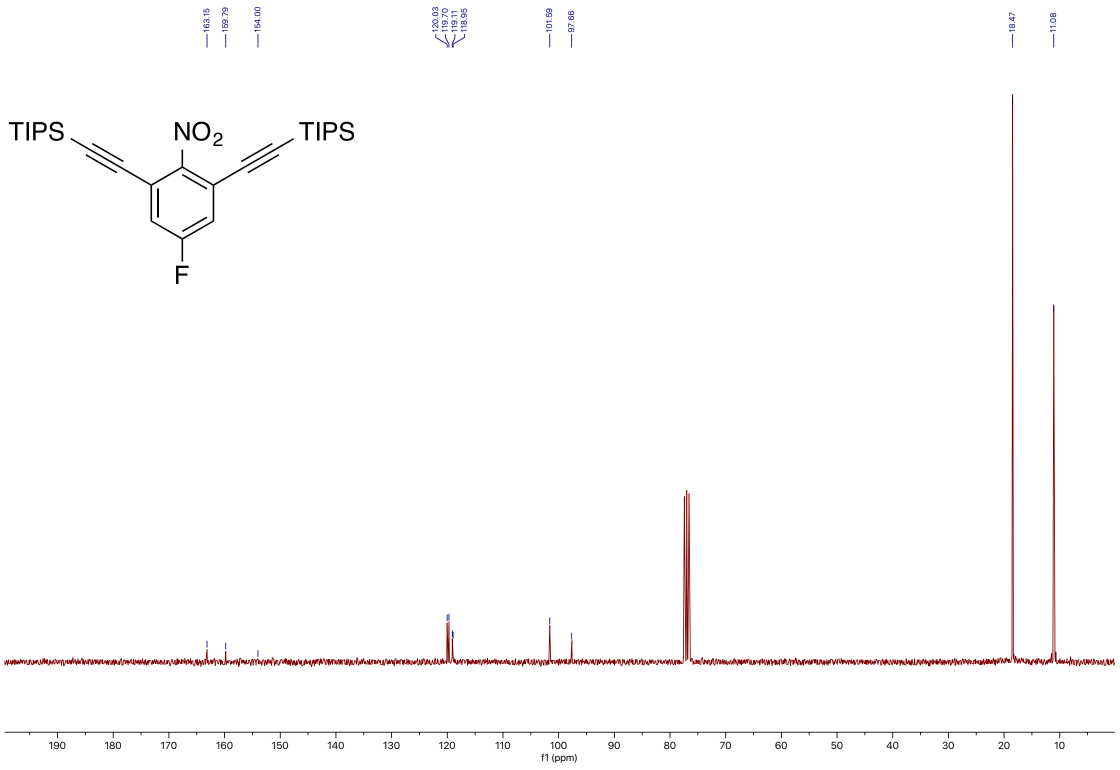

^{19}F NMR: 376 MHz, $CDCl_3$, compound **(3p')**

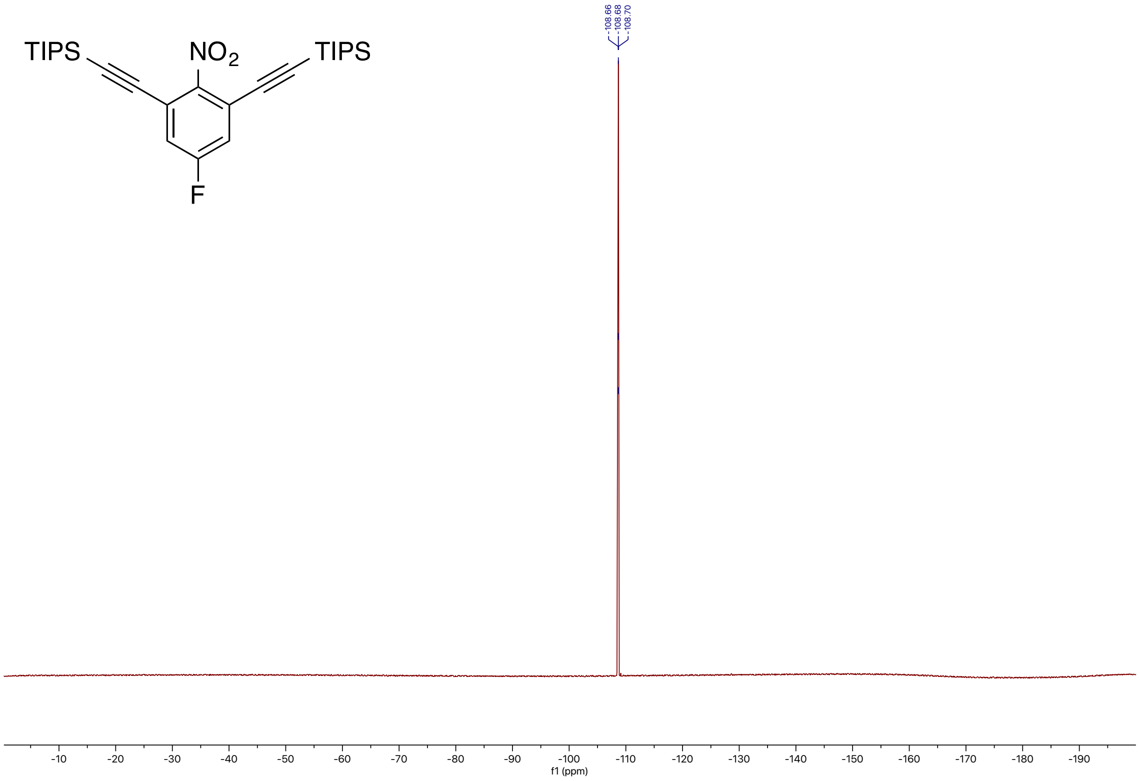

^{1}H NMR: 300 MHz, $CDCl_3$, compound **(3q)**

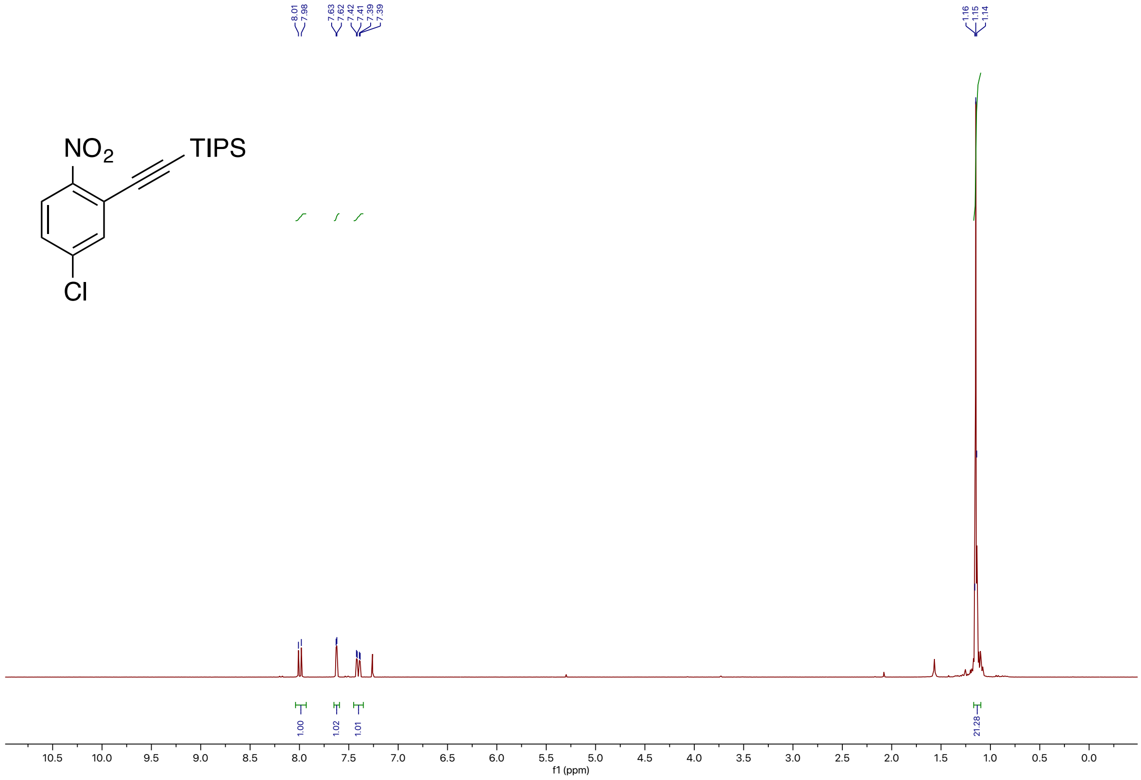

^{13}C NMR: 75 MHz, $CDCl_3$, compound **(3q)**

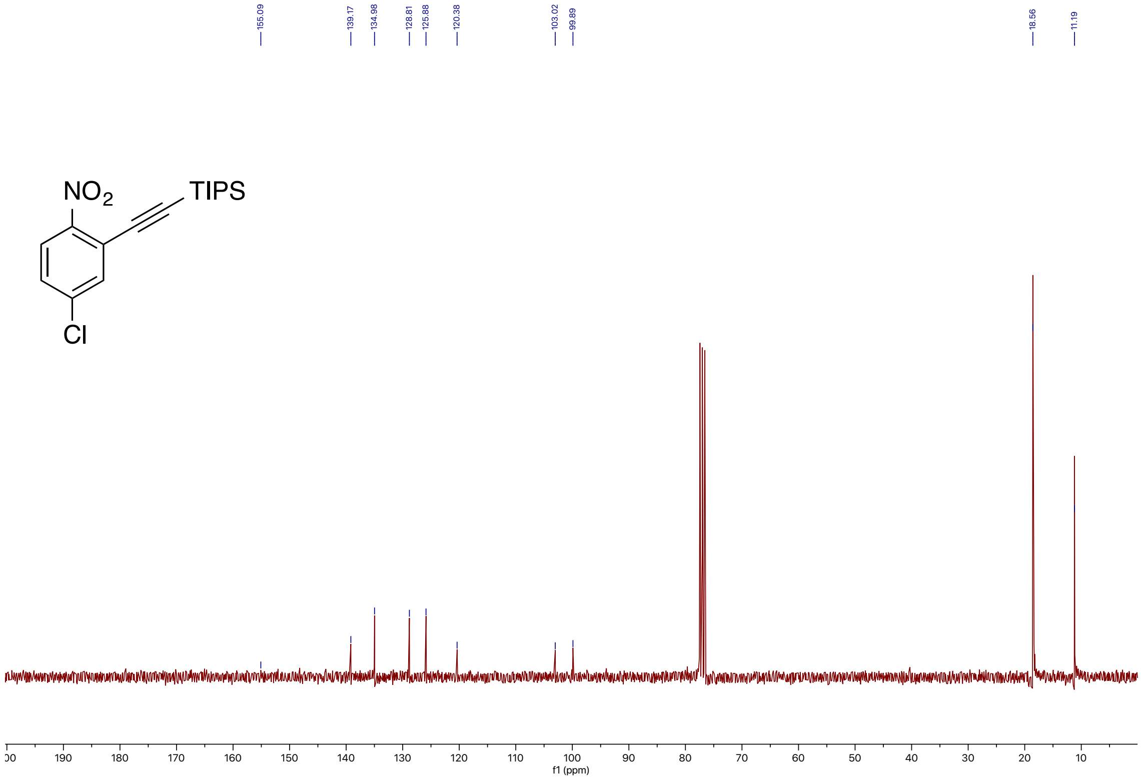

^{1}H NMR: 300 MHz, CDCl$_3$, compound **(3q')**

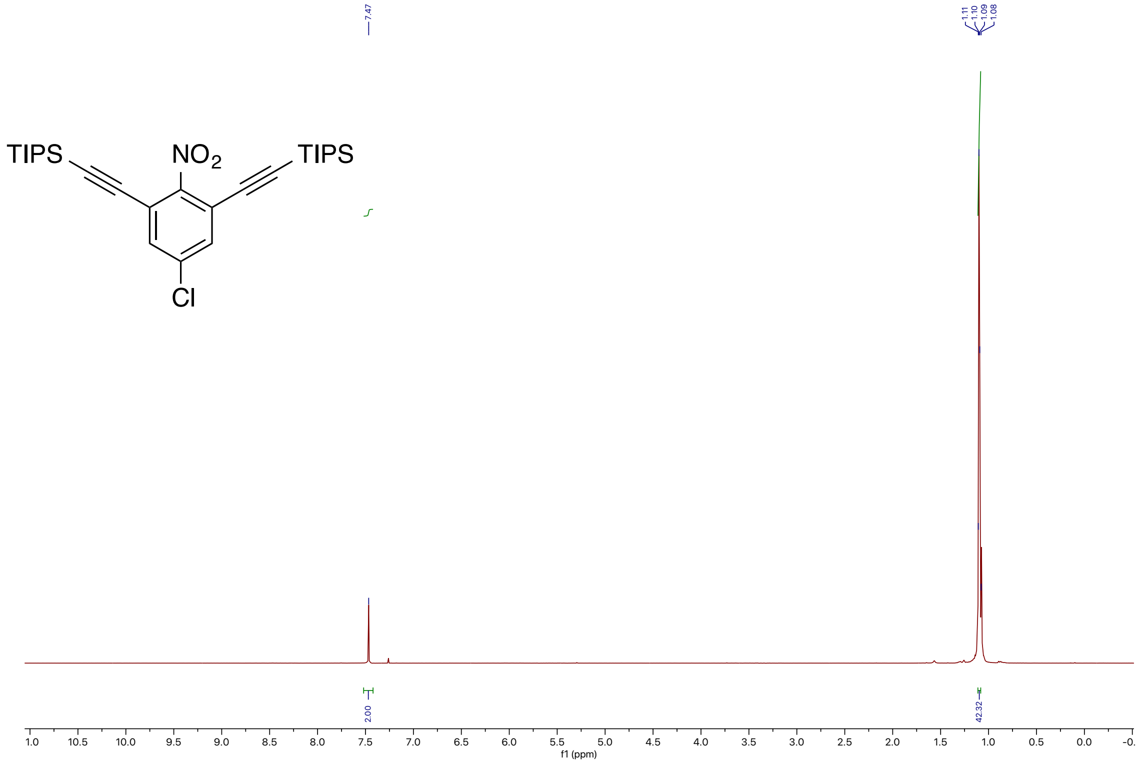

^{13}C NMR: 75 MHz, CDCl$_3$, compound **(3q')**

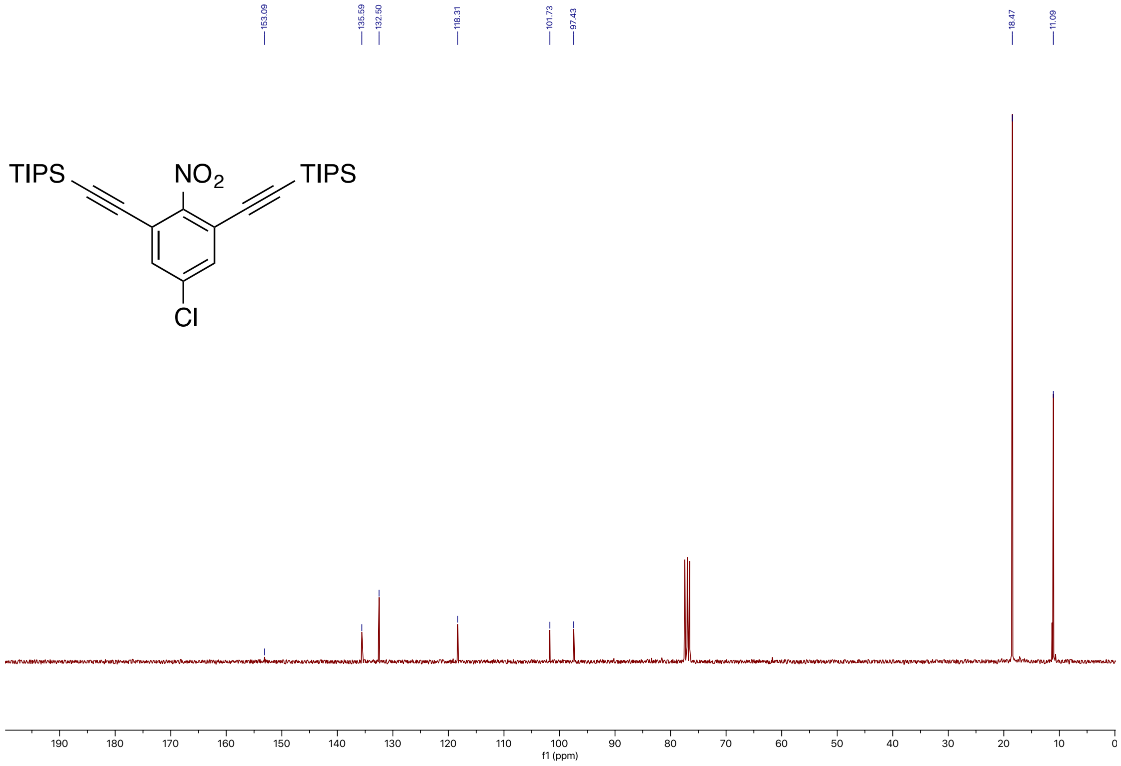

^{1}H NMR: 300 MHz, $CDCl_3$, compound **(3r)**

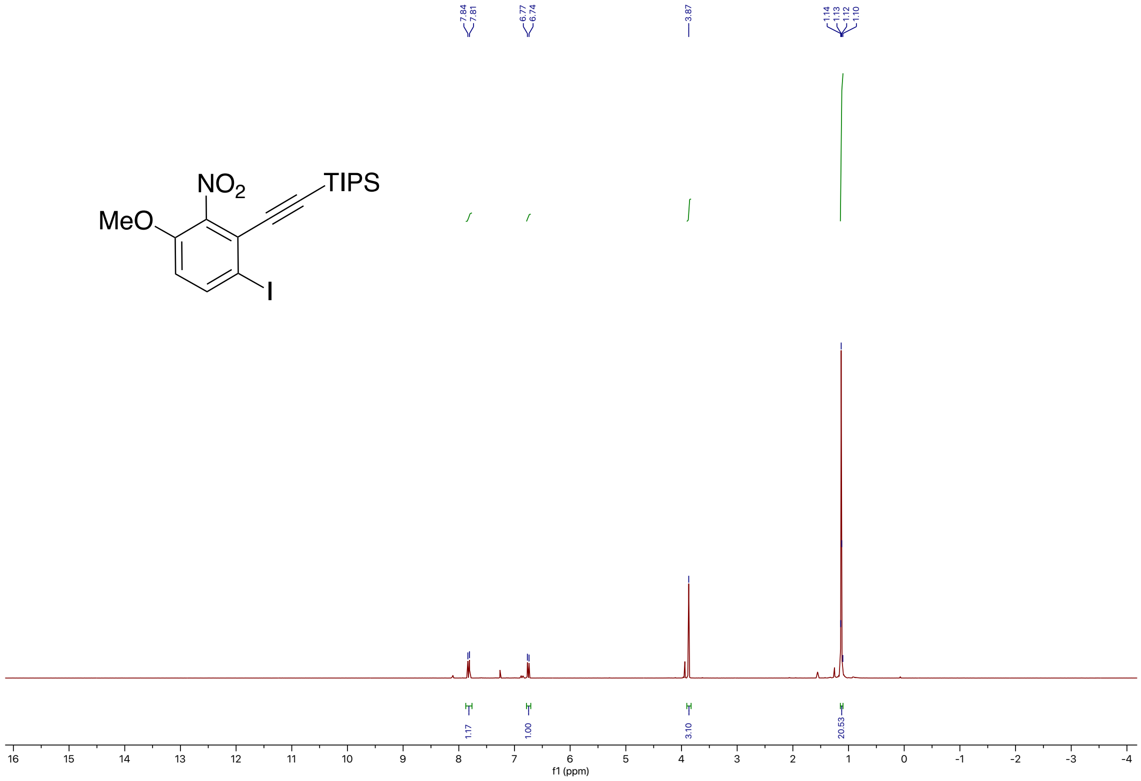

^{13}C NMR: 75 MHz, $CDCl_3$, compound **(3r)**

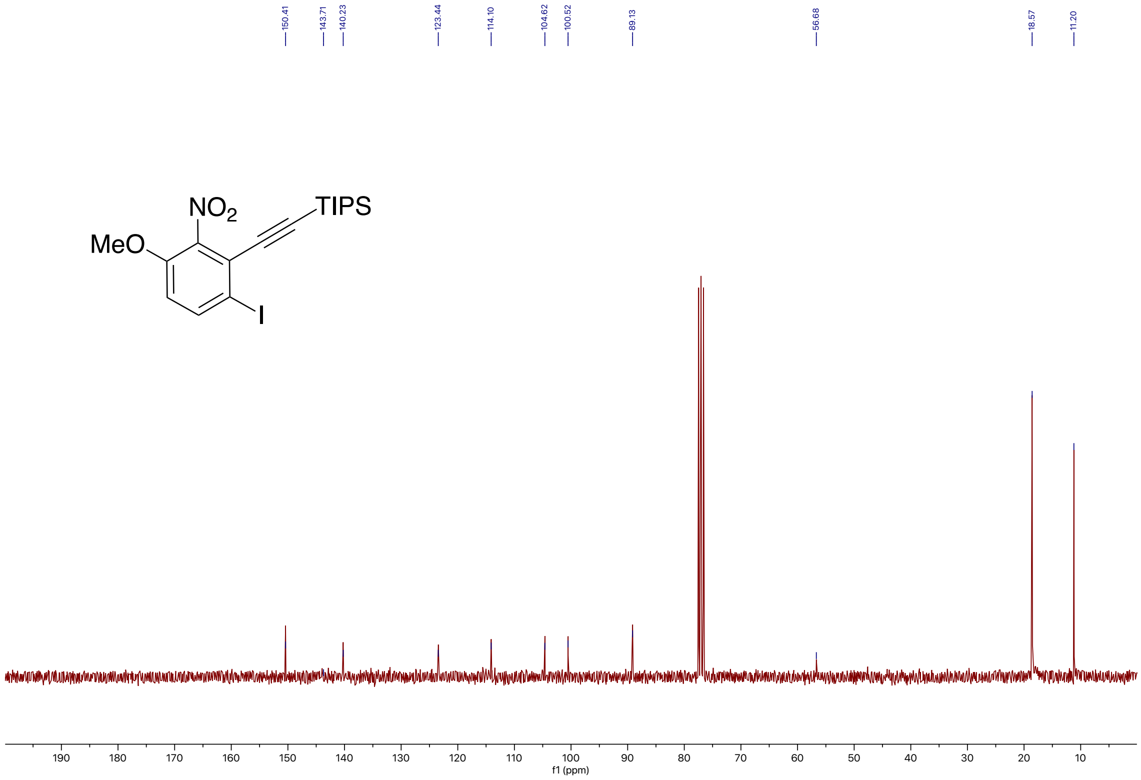

^{1}H NMR: 300 MHz, $CDCl_3$, compound **(3s)**

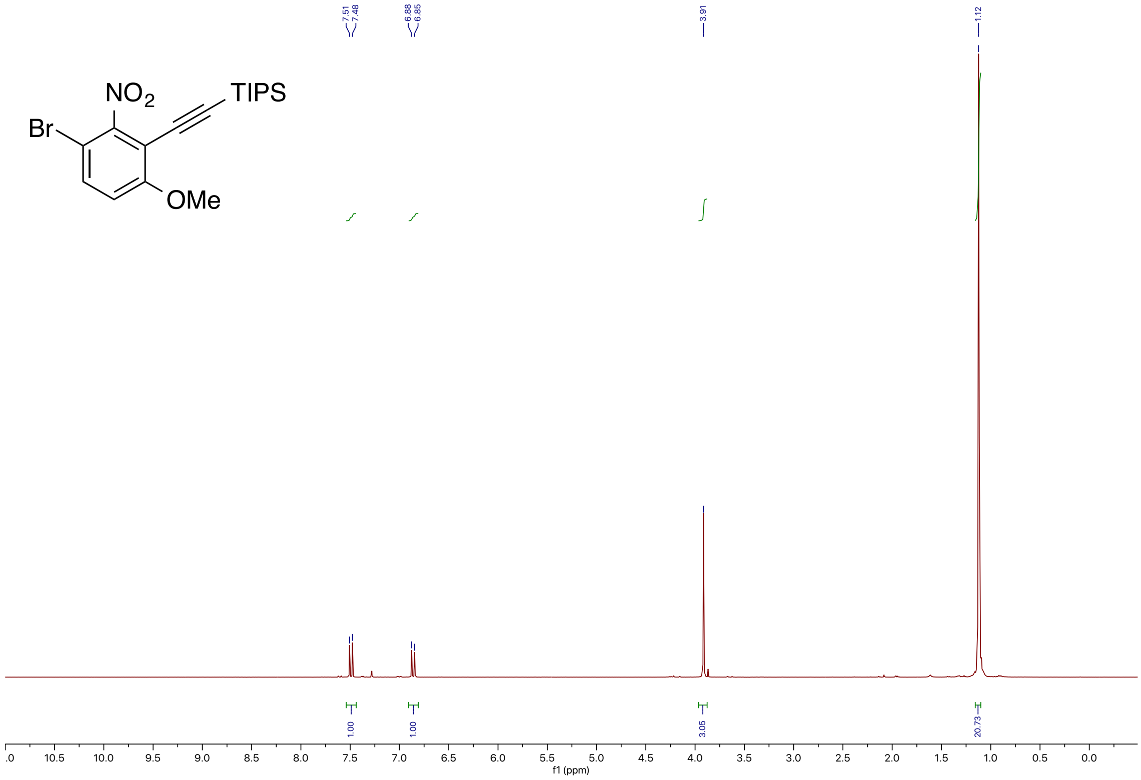

^{13}C NMR: 75 MHz, $CDCl_3$, compound **(3s)**

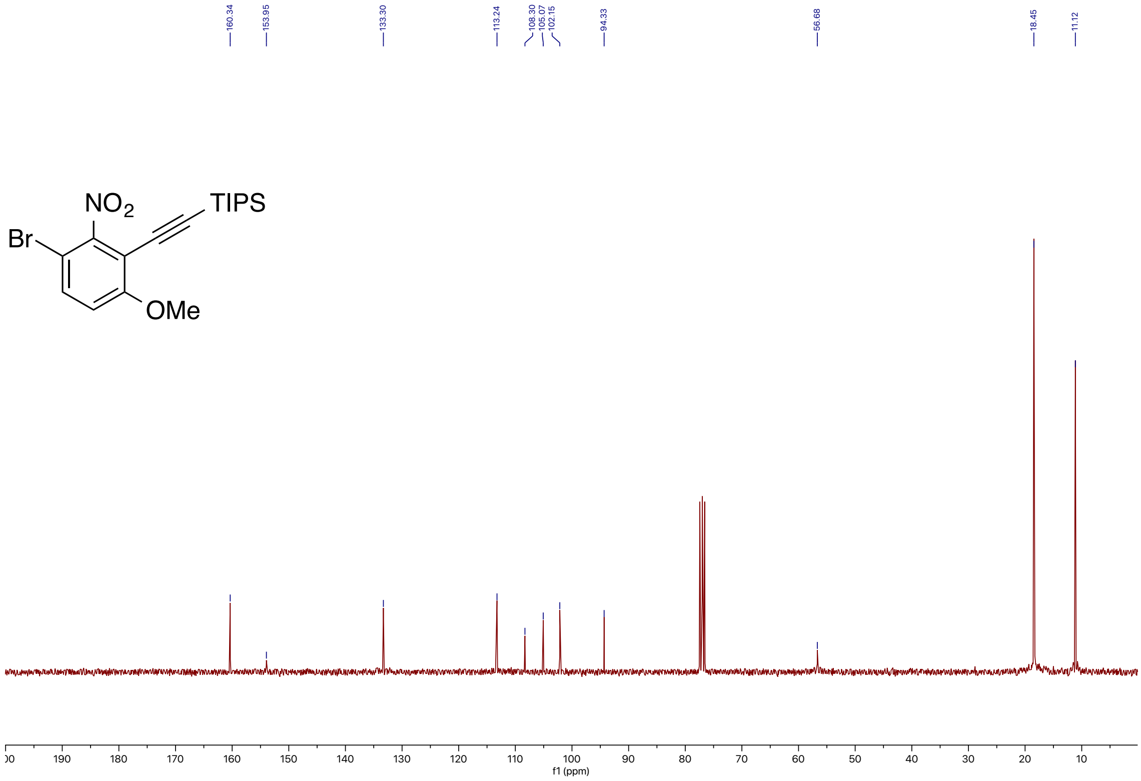

^{1}H NMR: 500 MHz, $CDCl_3$, compound **(3t)**

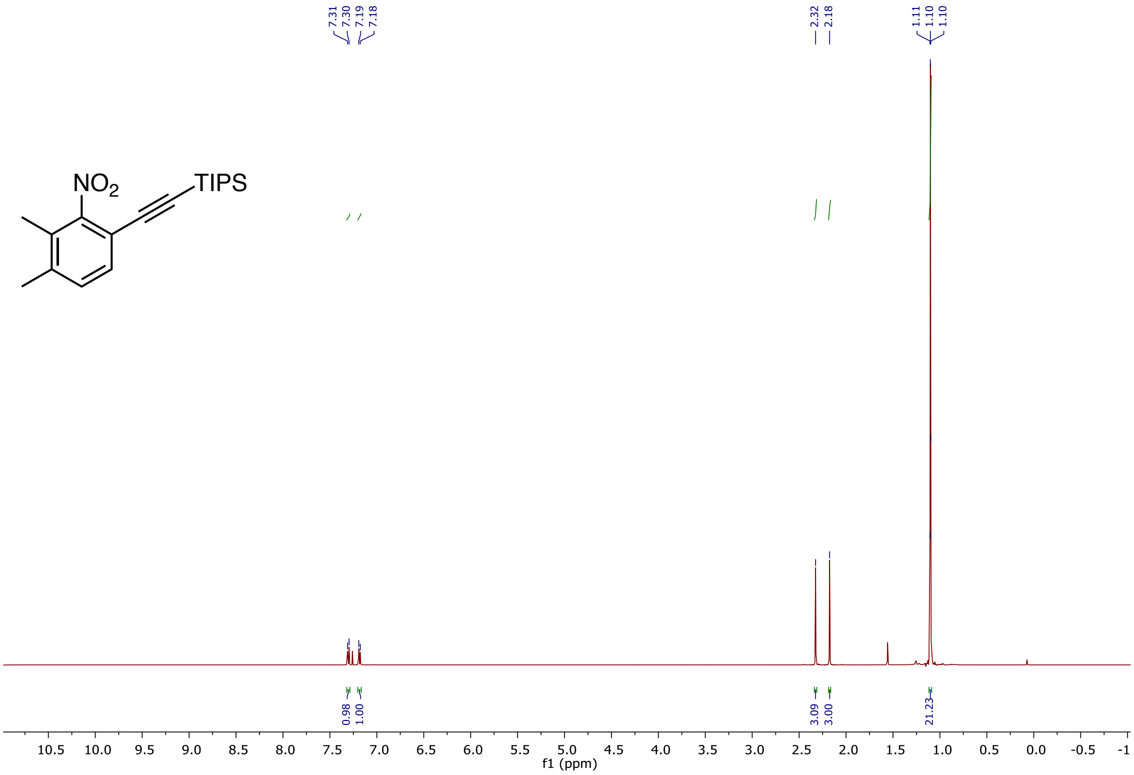

^{13}C NMR: 101 MHz, $CDCl_3$, compound **(3t)**

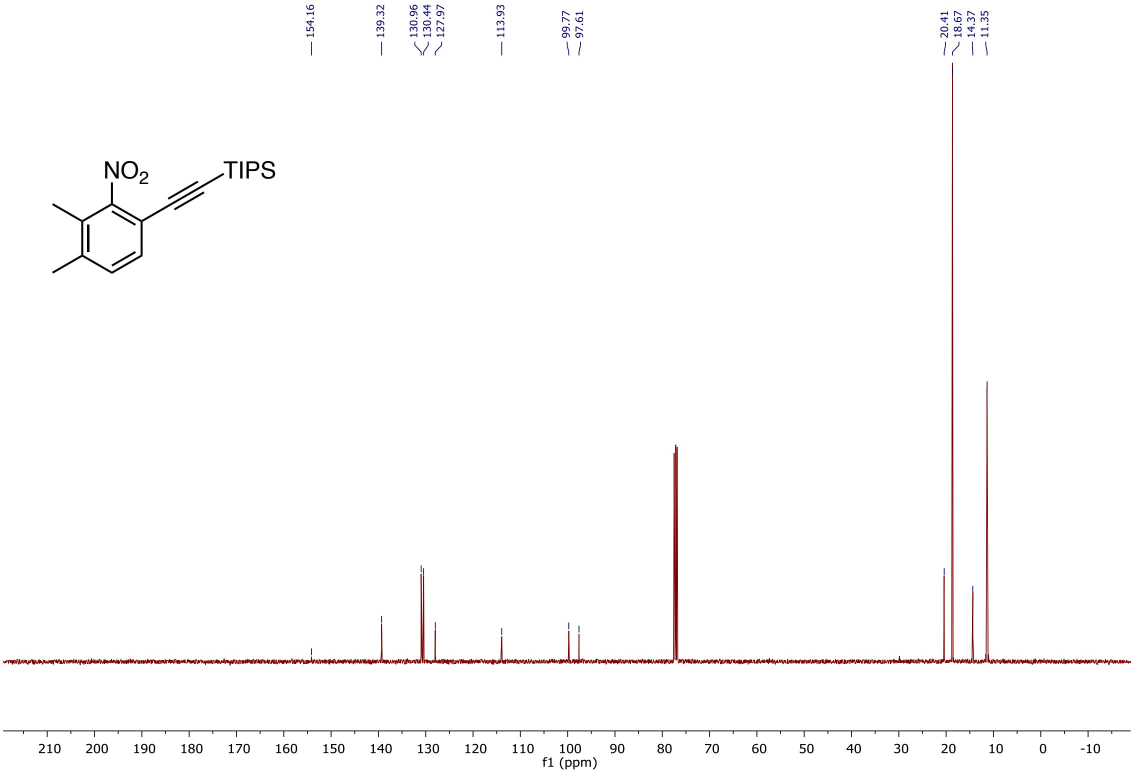

^{1}H NMR: 500 MHz, $CDCl_3$, compound **(3u)**

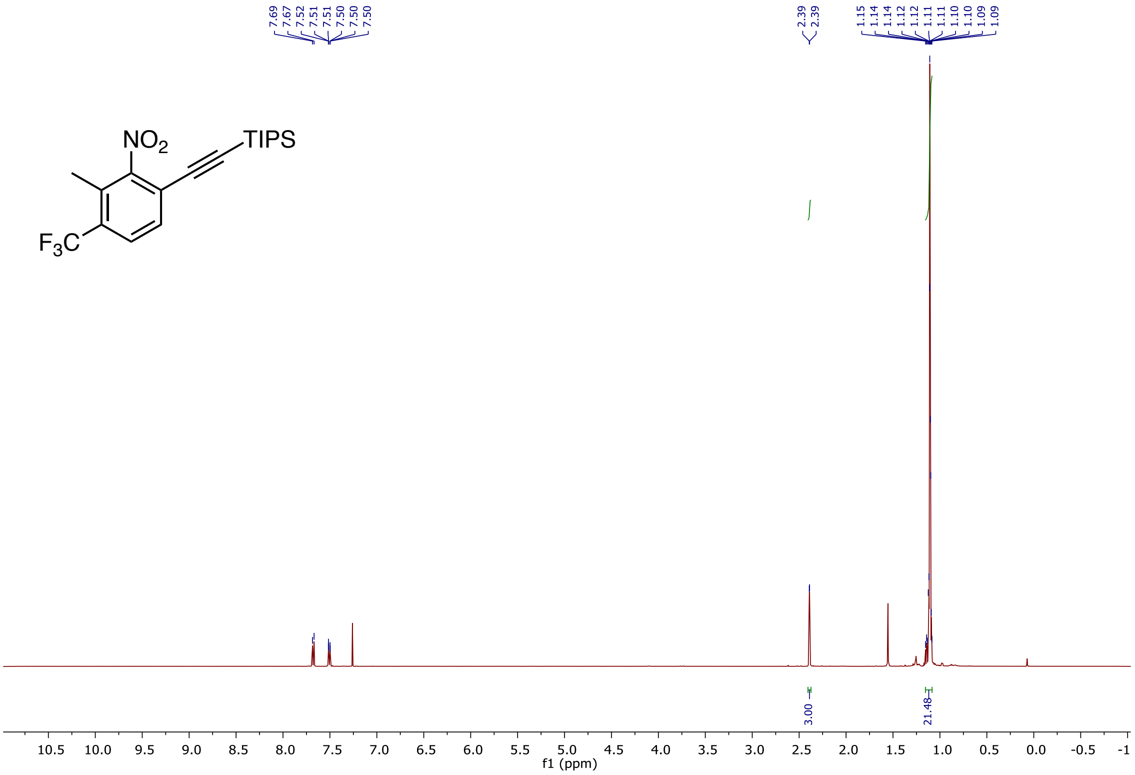

^{13}C NMR: 126 MHz, $CDCl_3$, compound **(3u)**

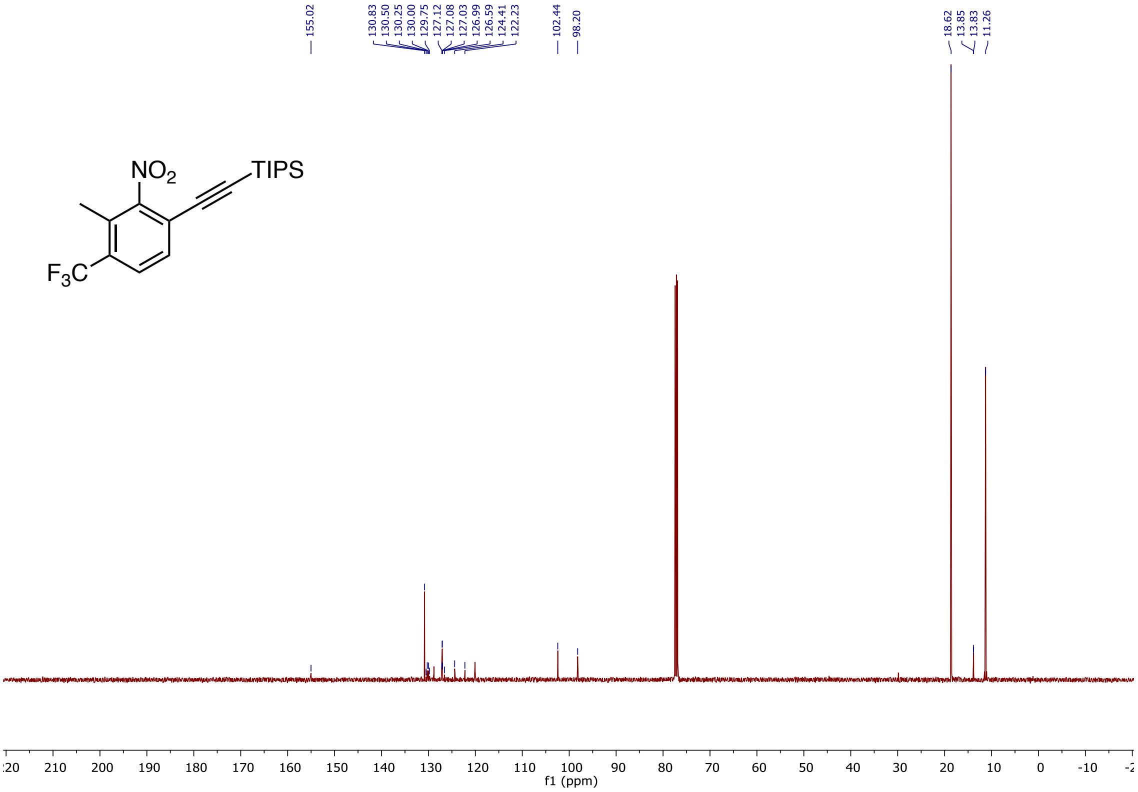

$^{19}F\{^{1}H\}$ NMR: 471 MHz, $CDCl_3$, compound **(3u)**

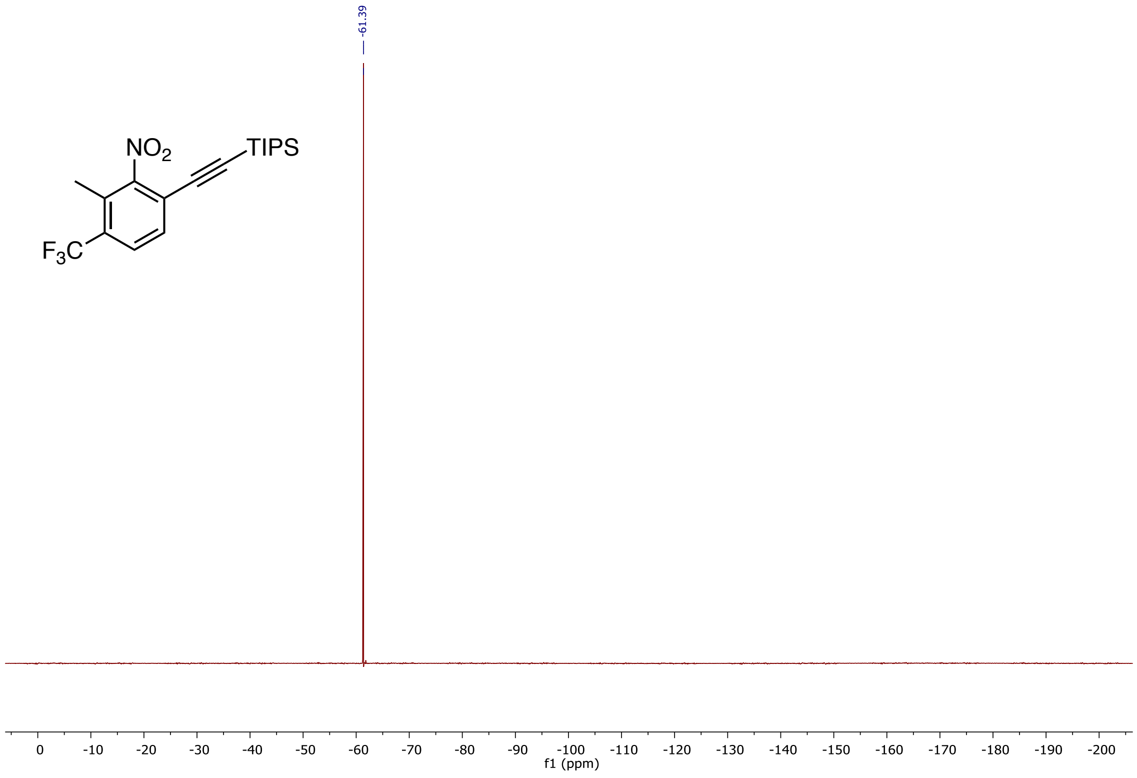

^{1}H NMR: 500 MHz, $CDCl_3$, compound **(3v)**

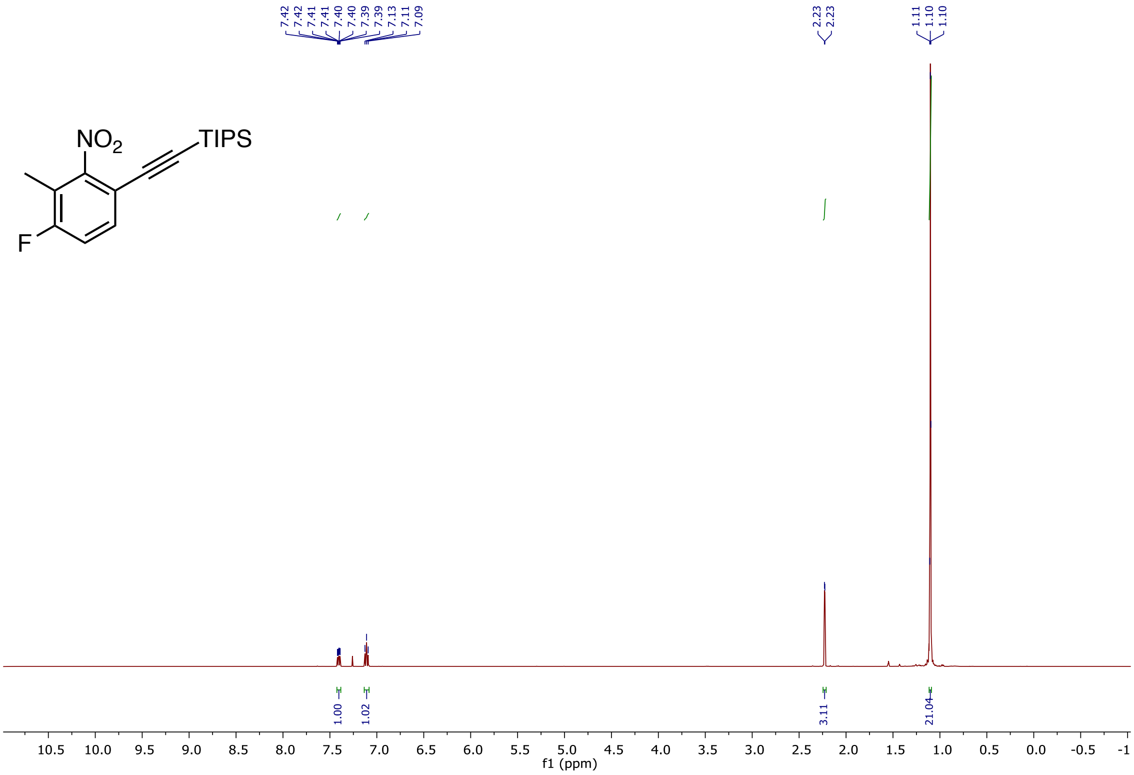

^{13}C NMR: 126 MHz, $CDCl_3$, compound **(3v)**

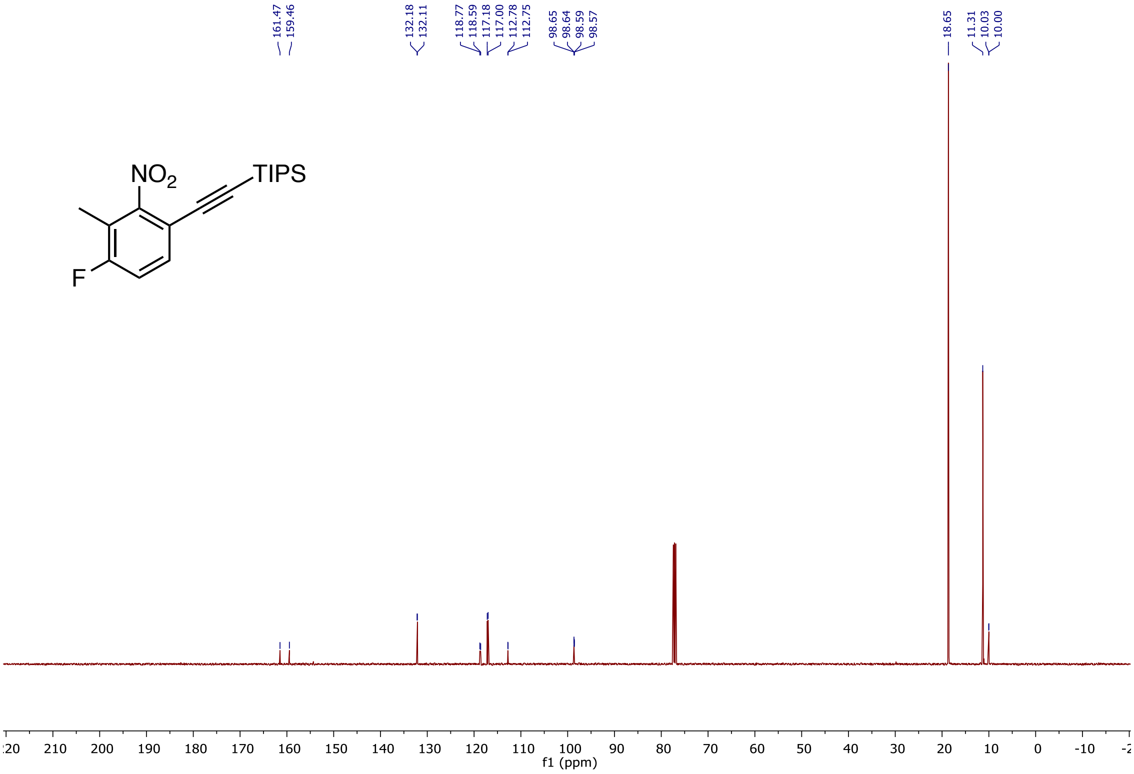

$^{19}F\{^{1}H\}$ NMR: 471 MHz, $CDCl_3$, compound **(3v)**

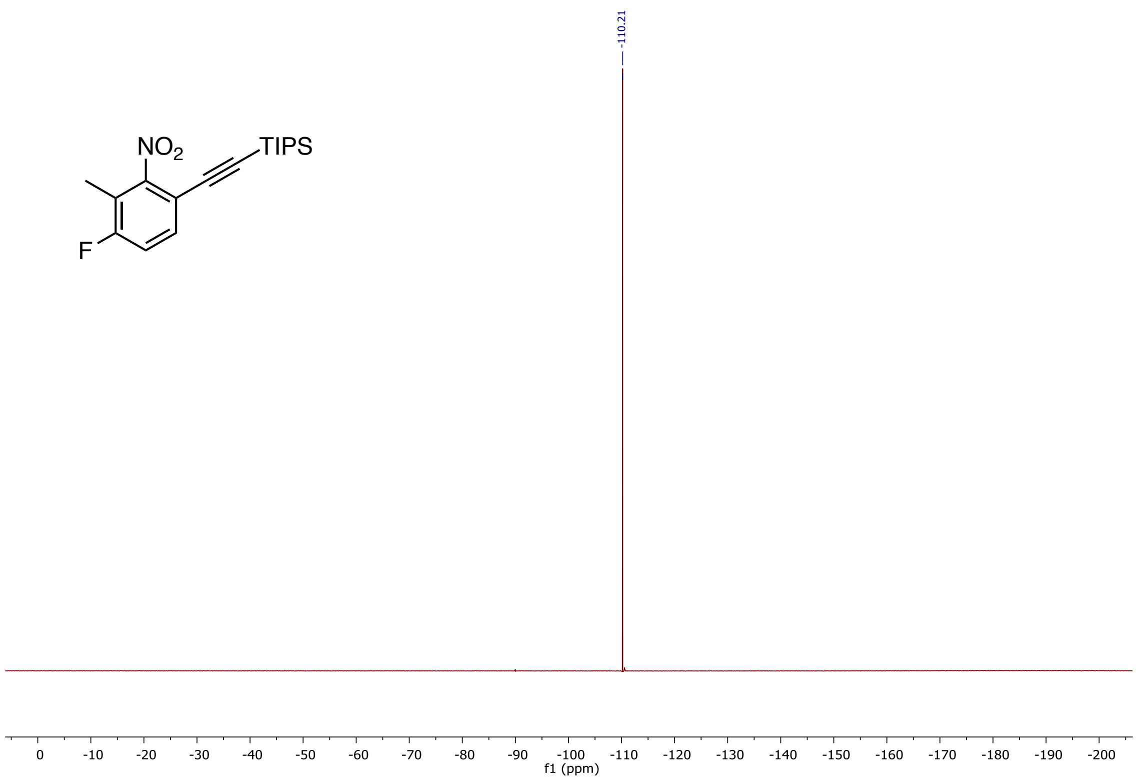

^{1}H NMR: 500 MHz, $CDCl_3$, compound **(3w)**

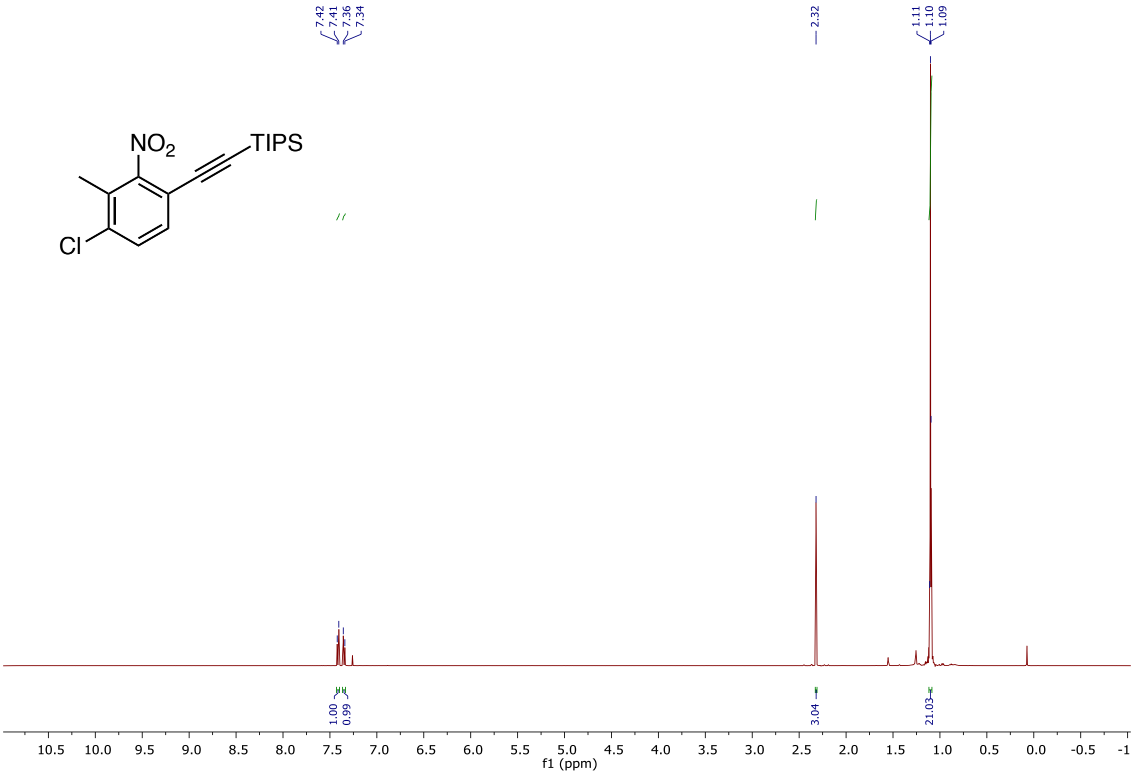

^{13}C NMR: 126 MHz, $CDCl_3$, compound **(3w)**

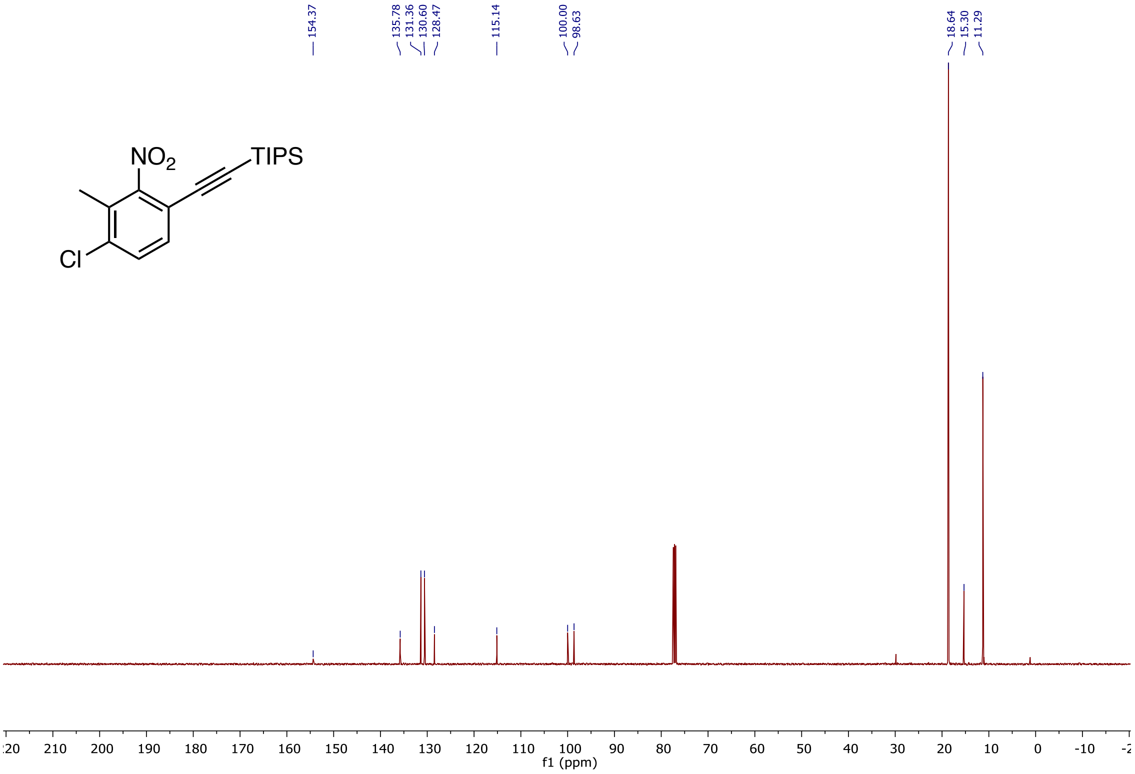

^{1}H NMR: 500 MHz, $CDCl_3$, compound **(3x)**

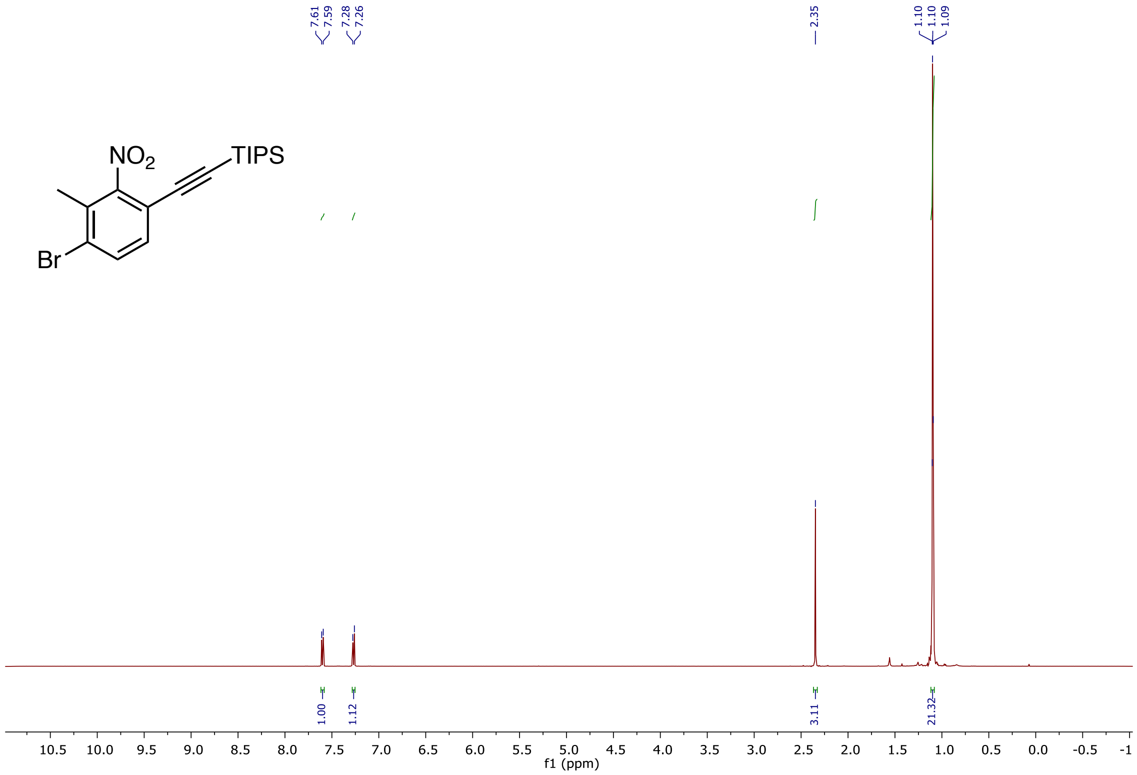

^{13}C NMR: 126 MHz, $CDCl_3$, compound **(3x)**

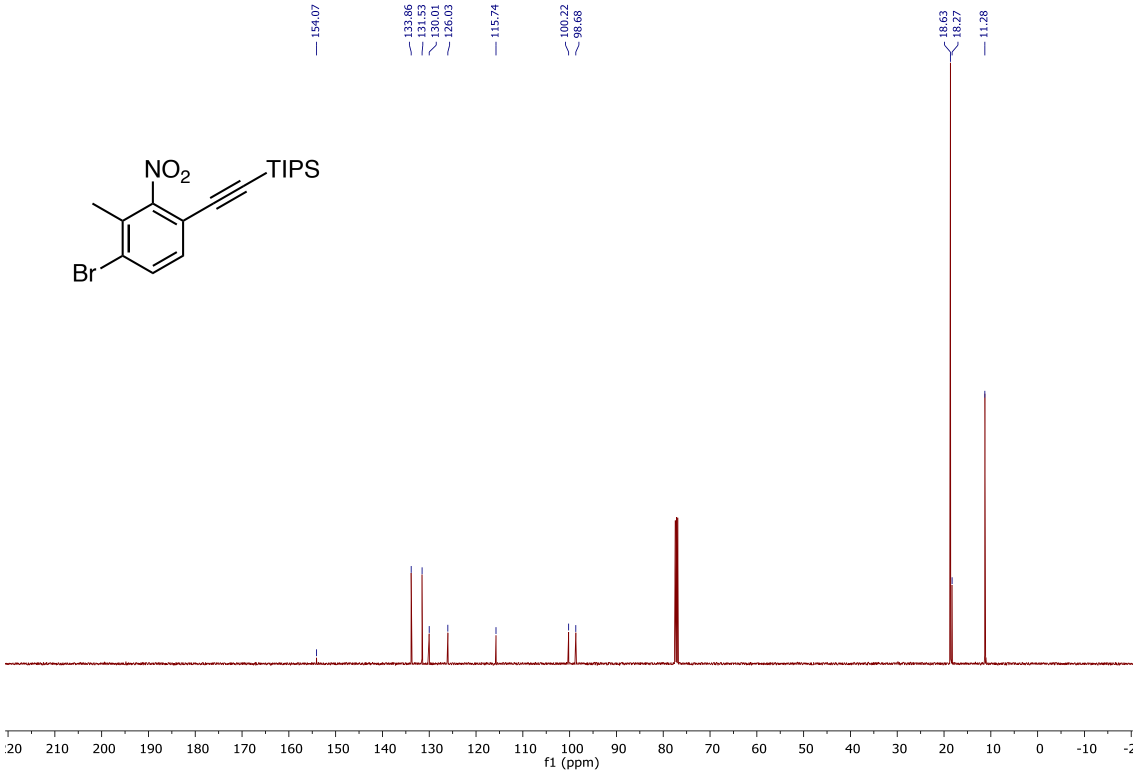

^{1}H NMR: 500 MHz, $CDCl_3$, compound **(3y)**

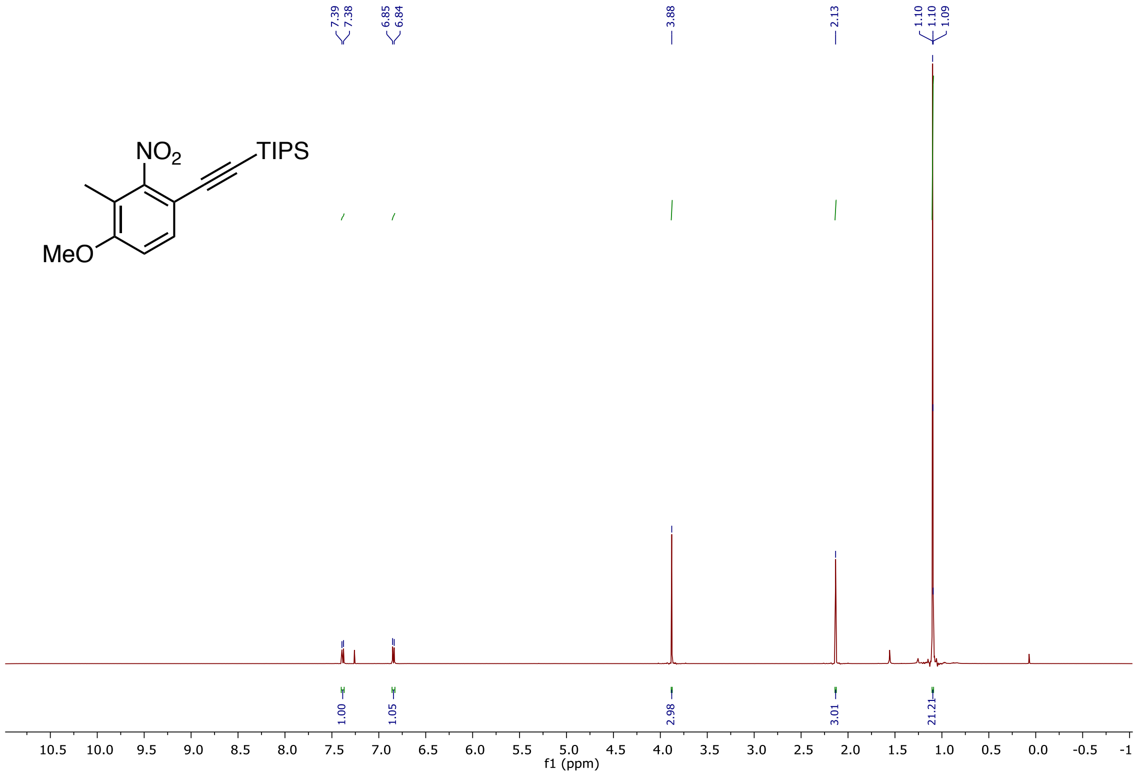

^{13}C NMR: 126 MHz, $CDCl_3$, compound **(3y)**

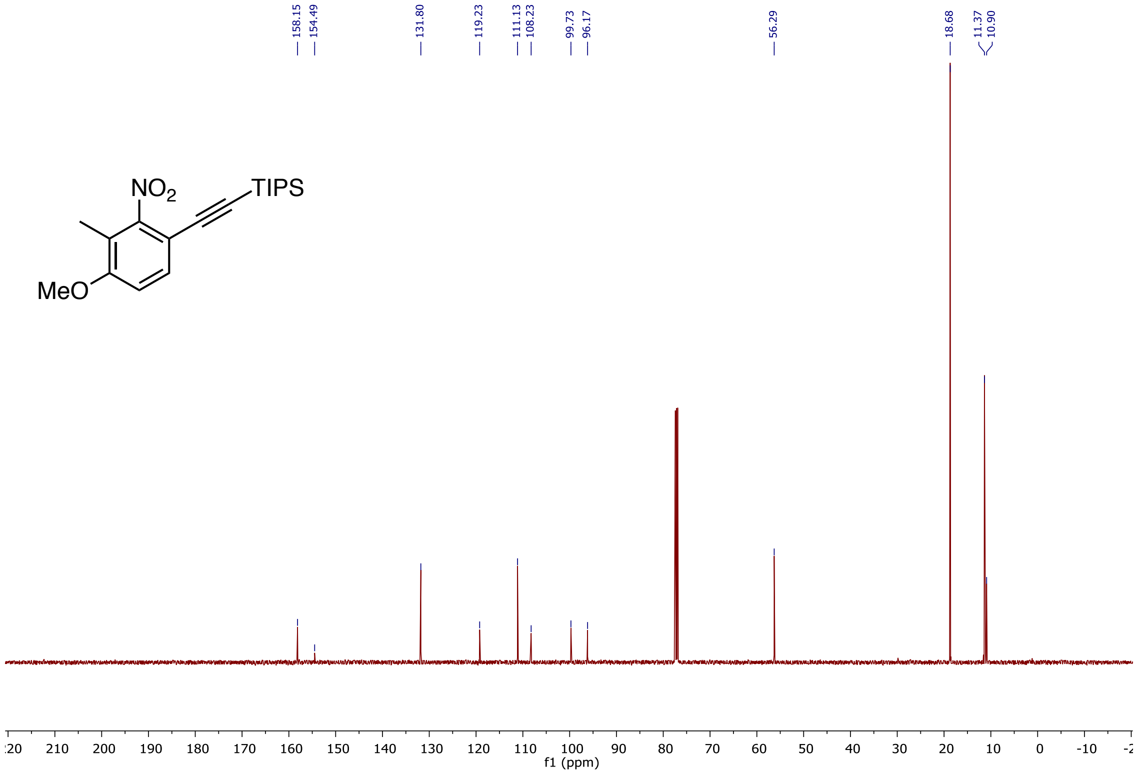

^{1}H NMR: 400 MHz, $CDCl_3$, compound **(3z)**

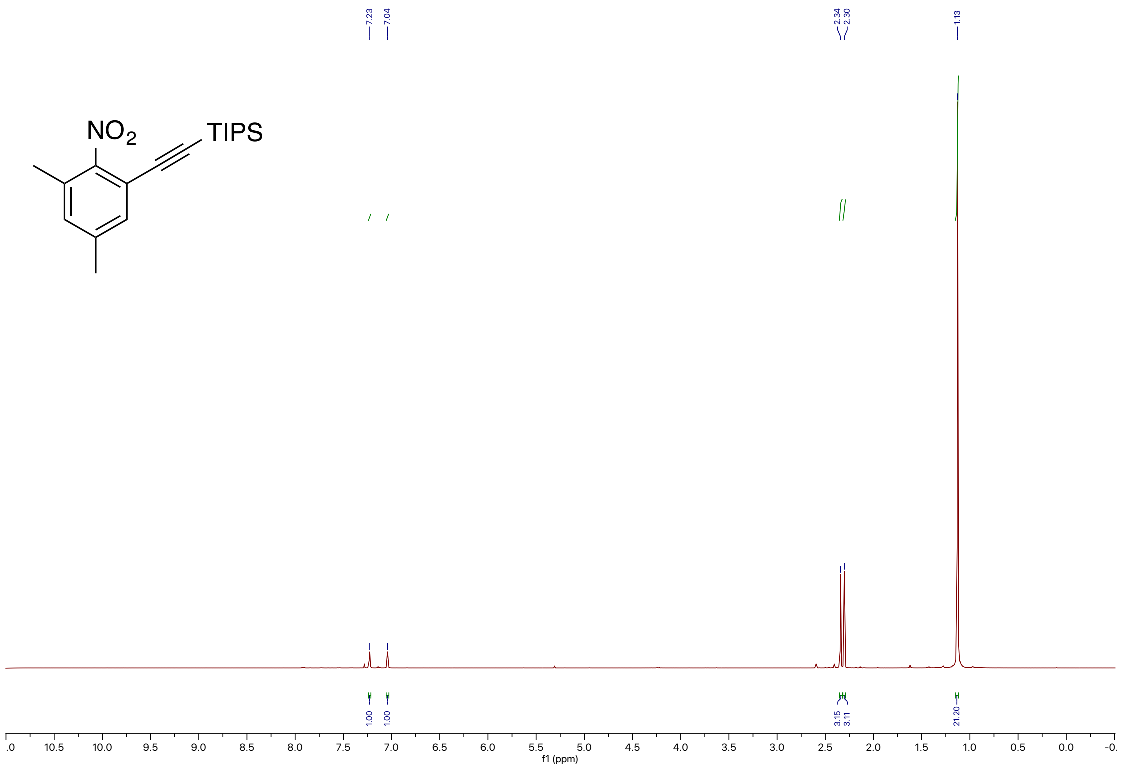

^{13}C NMR: 101 MHz, $CDCl_3$, compound (**3z**)

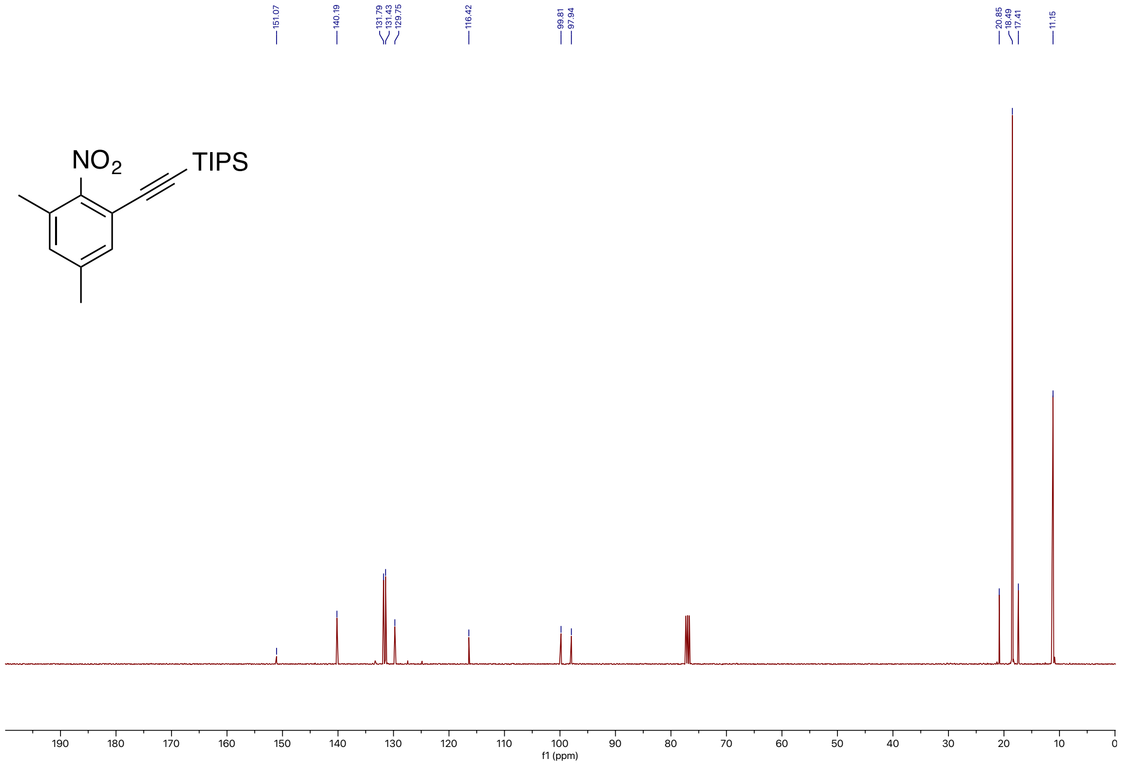

^{1}H NMR: 500 MHz, $CDCl_3$, compound (**3aa**)

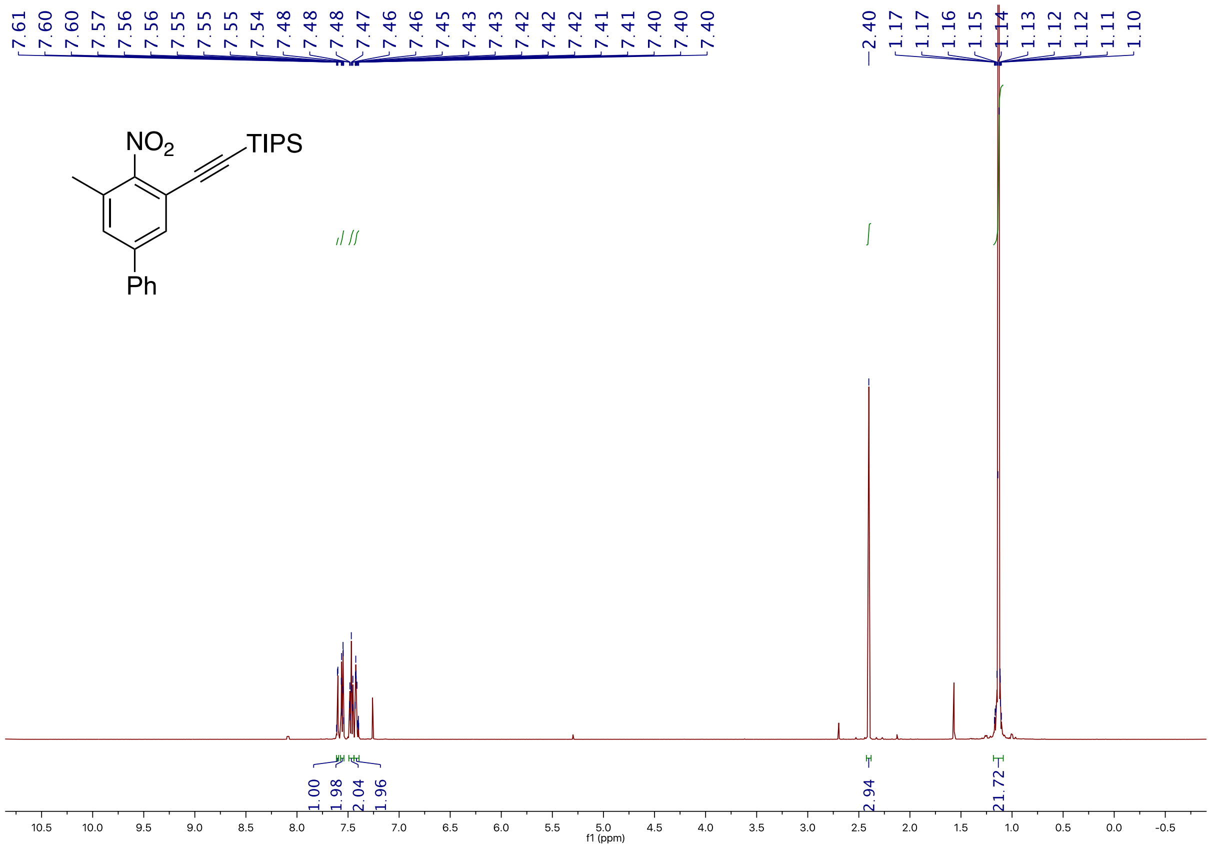

^{13}C NMR: 126 MHz, $CDCl_3$, compound (**3aa**)

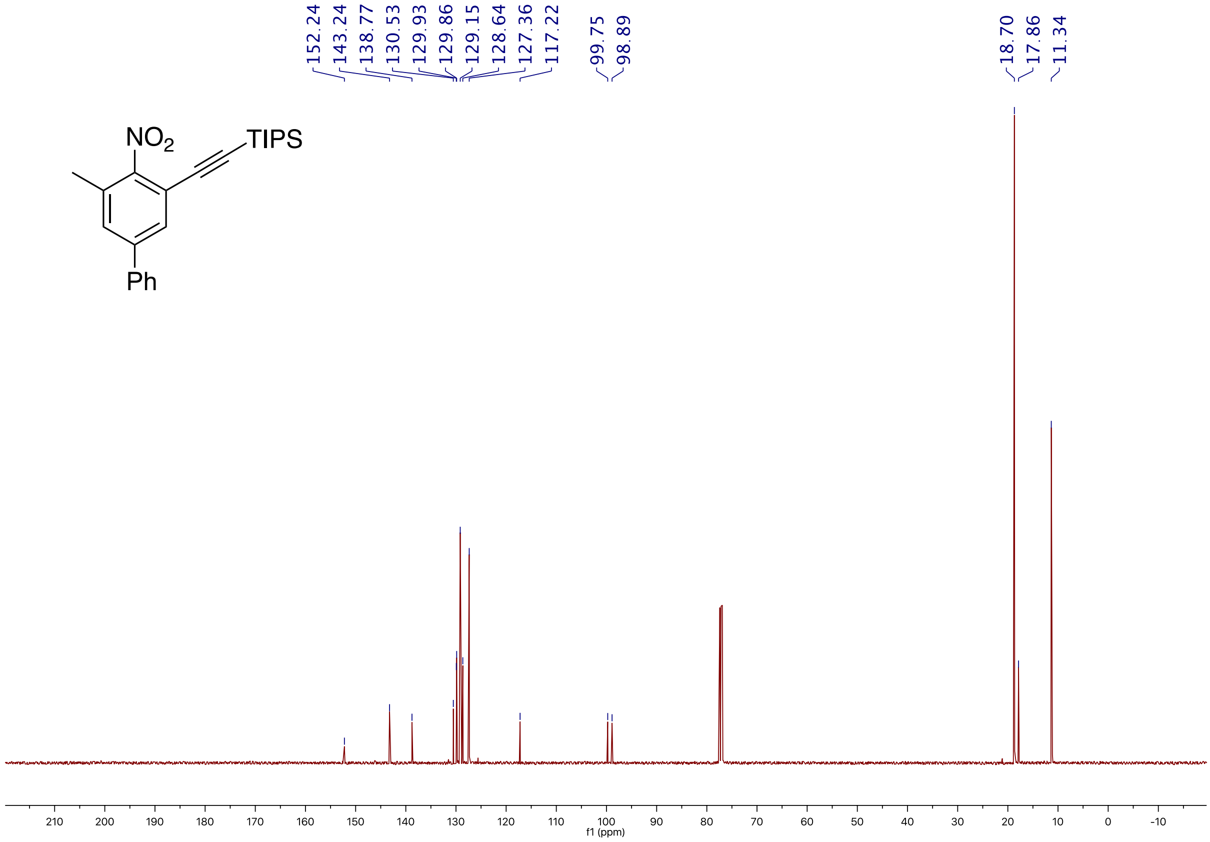

^{1}H NMR: 500 MHz, $CDCl_3$, compound **(3ab)**

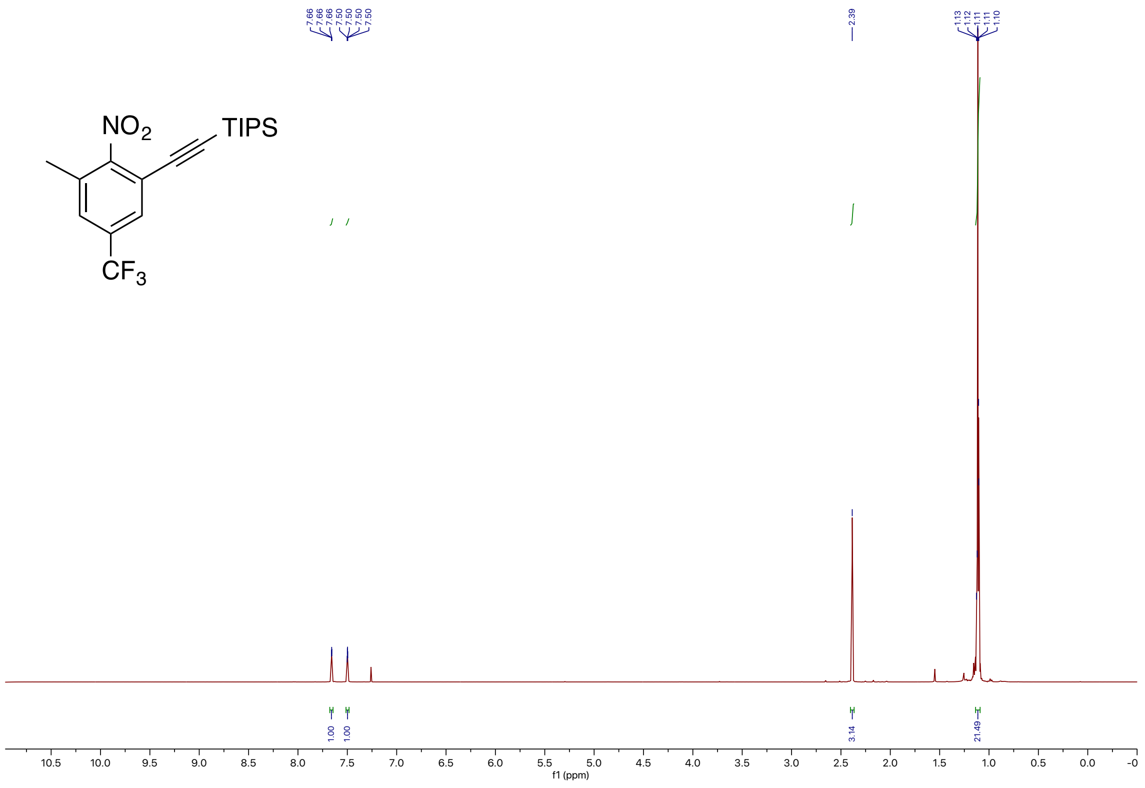

^{13}C NMR: 126 MHz, $CDCl_3$, compound **(3ab)**

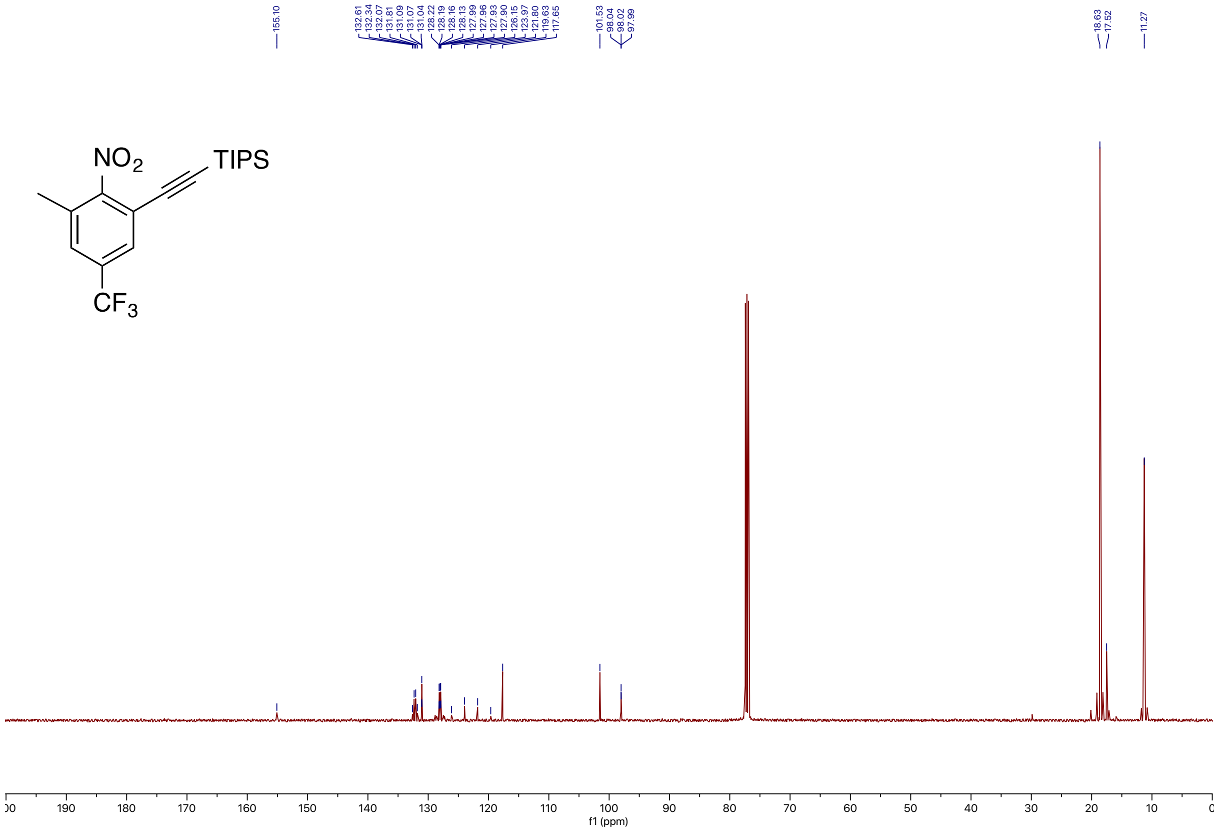

$^{19}F\{^1H\}$ NMR: 376 MHz, $CDCl_3$, compound **(3ab)**

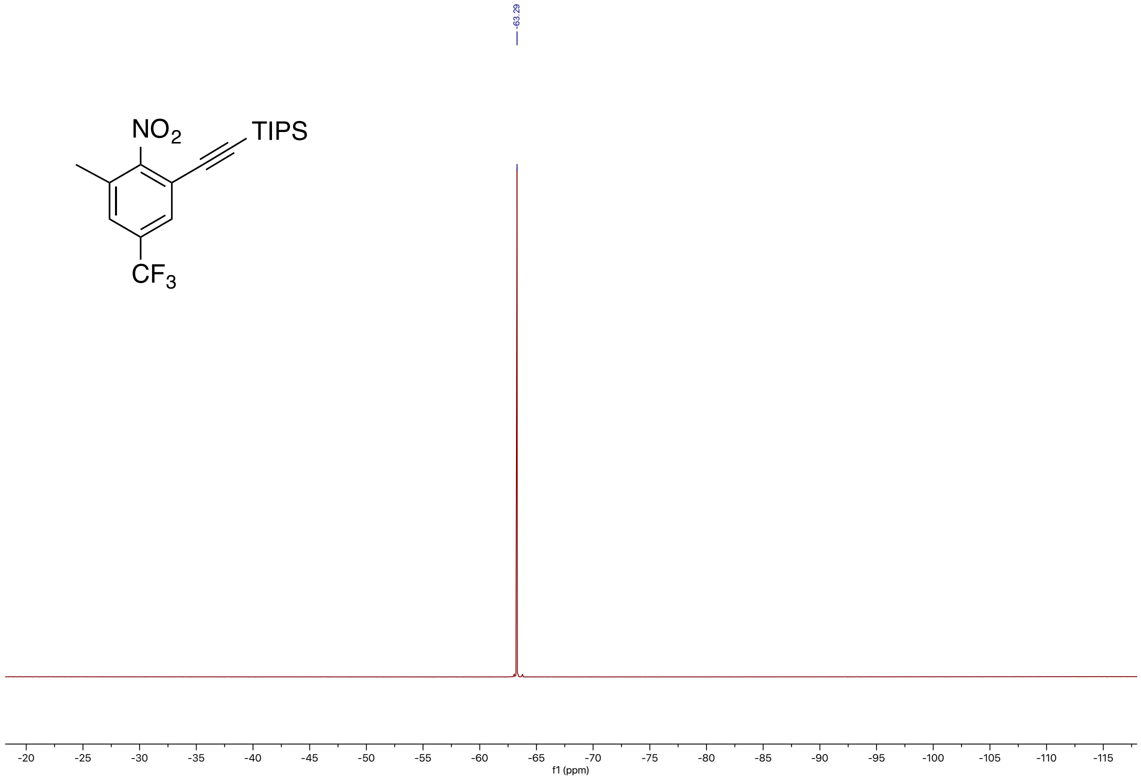

^{1}H NMR: 400 MHz, $CDCl_3$, compound **(3ac)**

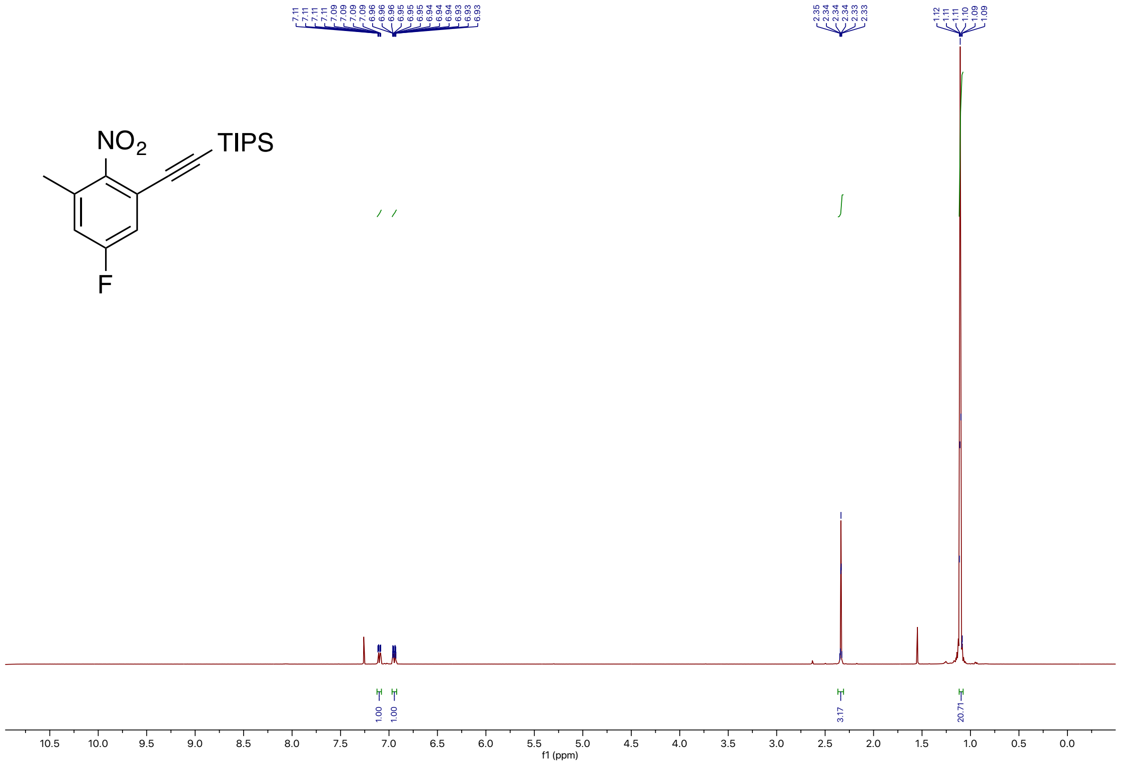

^{13}C NMR: 101 MHz, $CDCl_3$, compound **(3ac)**

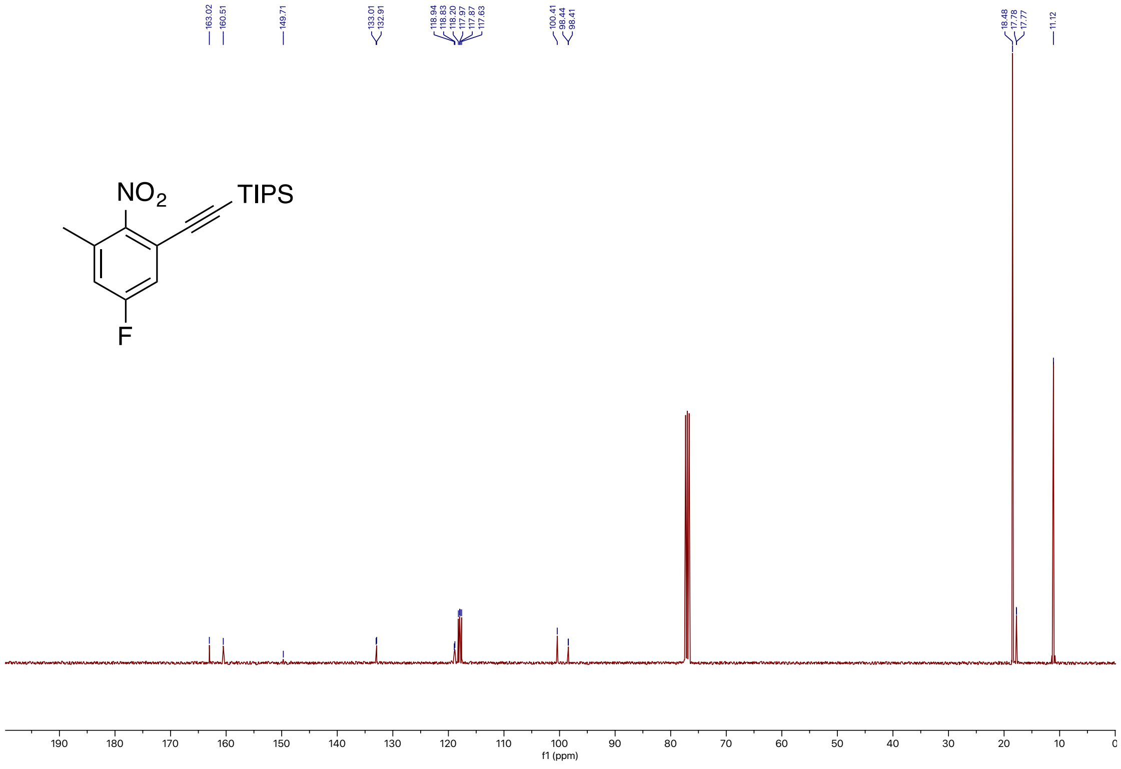

$^{19}F\{^{1}H\}$ NMR: 376 MHz, $CDCl_3$, compound **(3ac)**

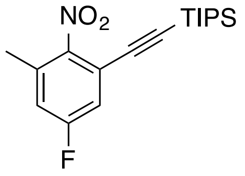

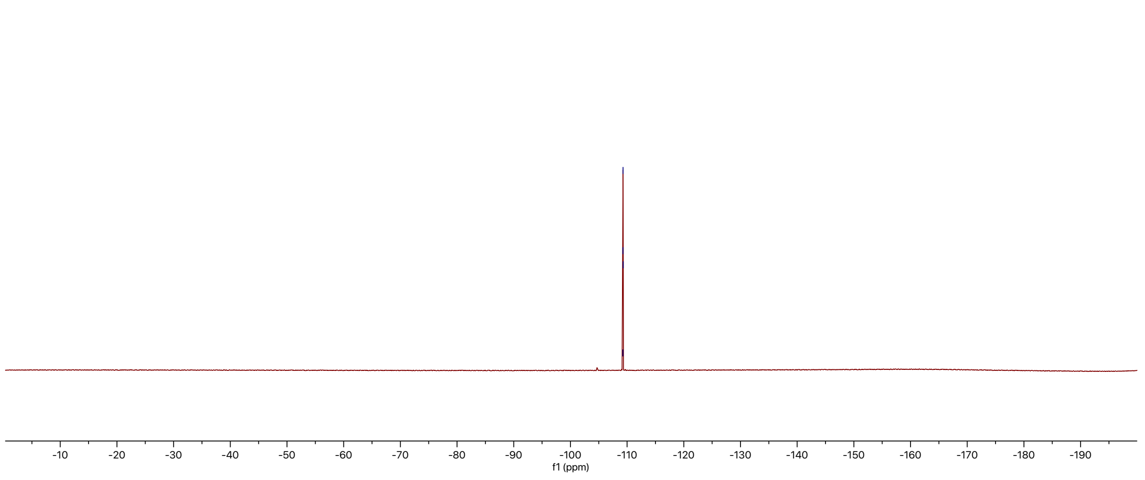

^{1}H NMR: 400 MHz, $CDCl_3$, compound **(3ad)**

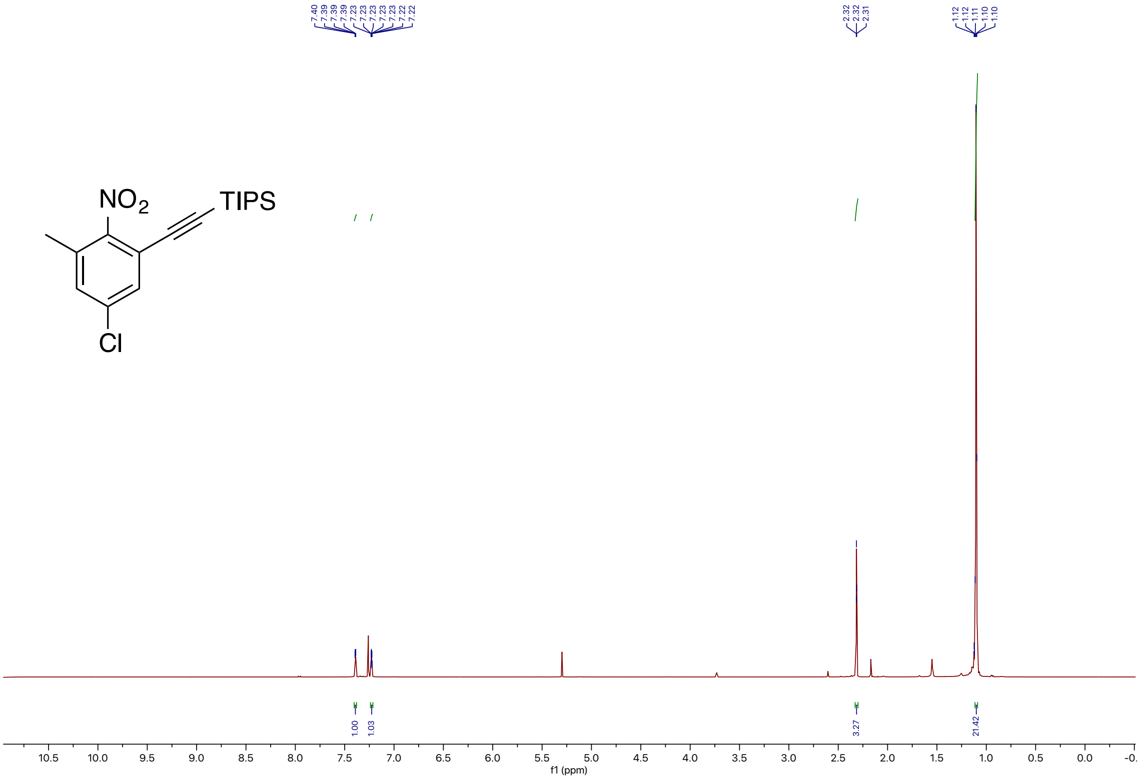

^{13}C NMR: 126 MHz, $CDCl_3$, compound **(3ad)**

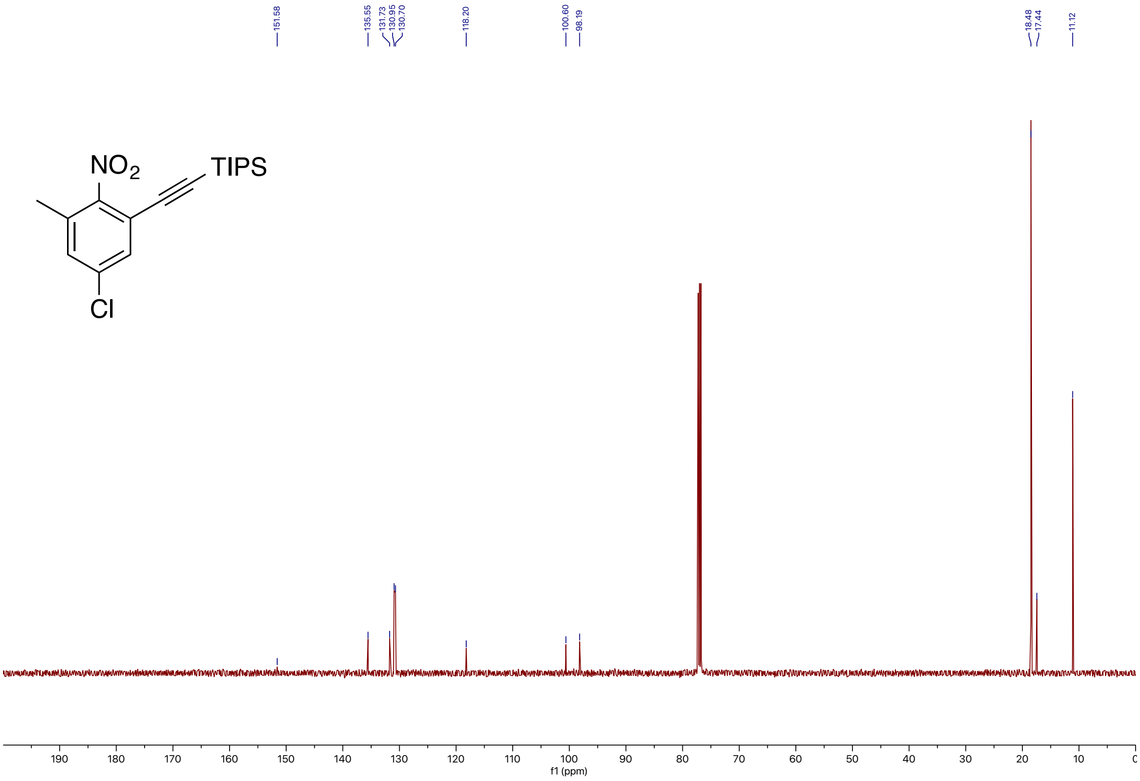

^{1}H NMR: 400 MHz, $CDCl_3$, compound **(3ae)**

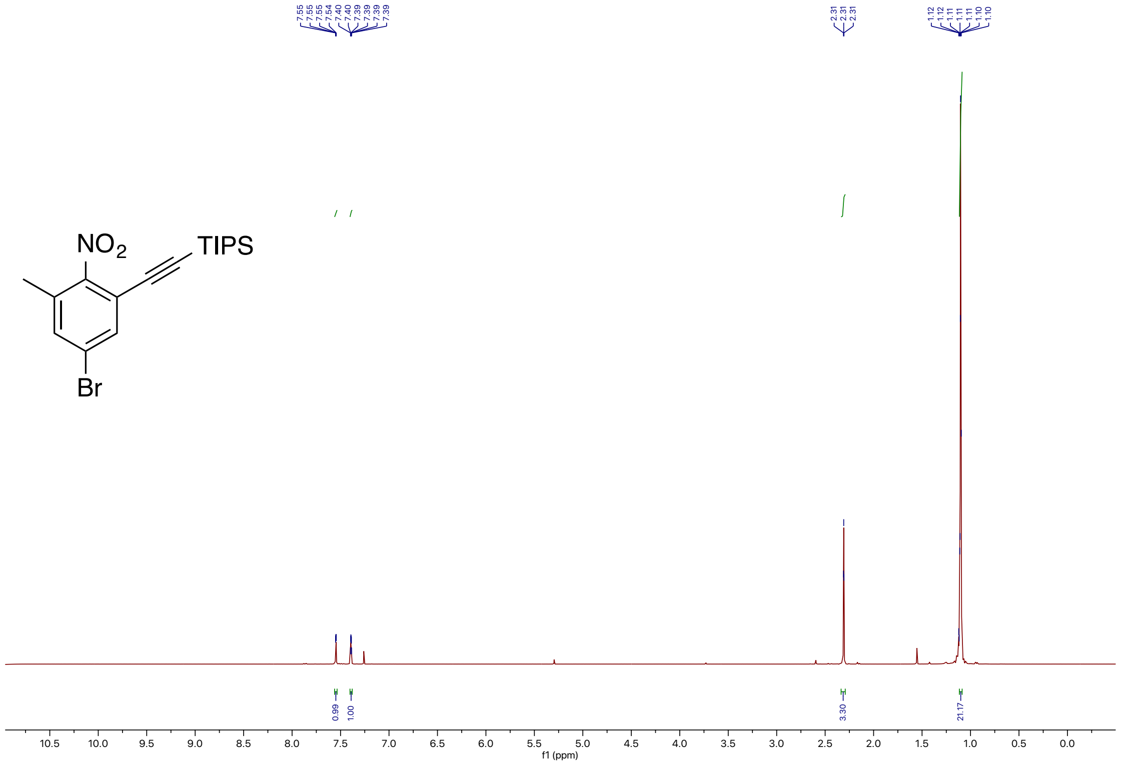

^{13}C NMR: 101 MHz, $CDCl_3$, compound **(3ae)**

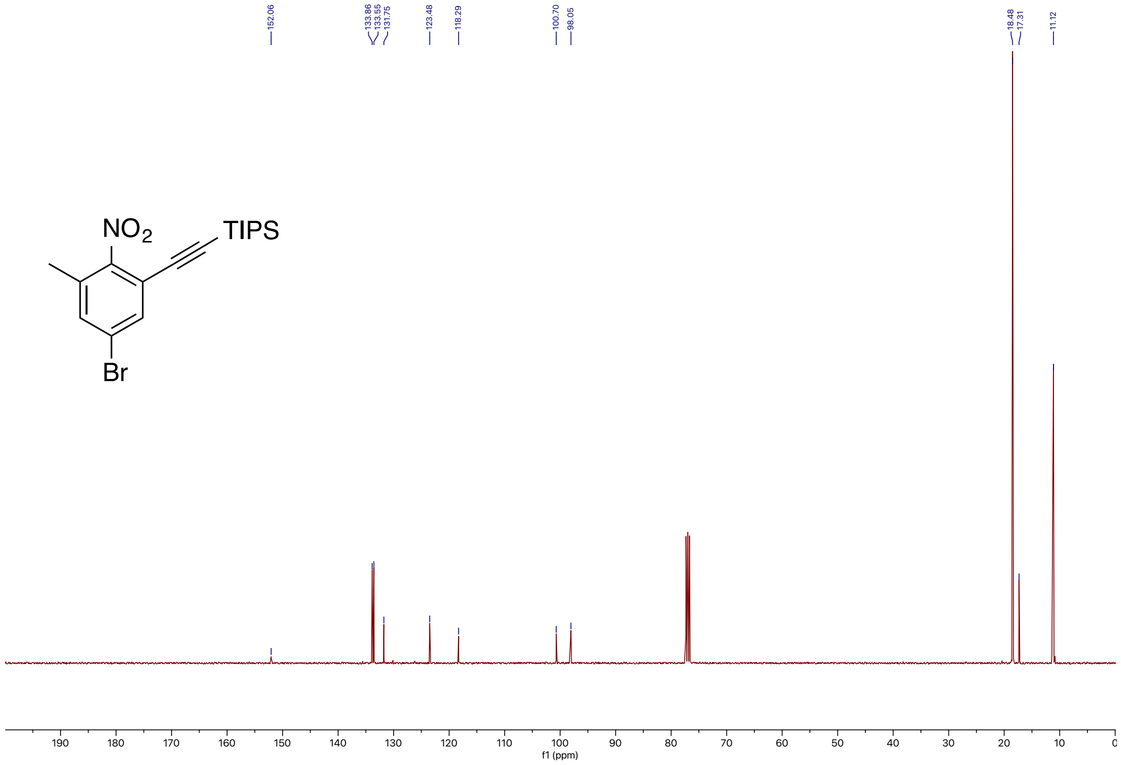

^{1}H NMR: 500 MHz, $CDCl_3$, compound **(3af)**

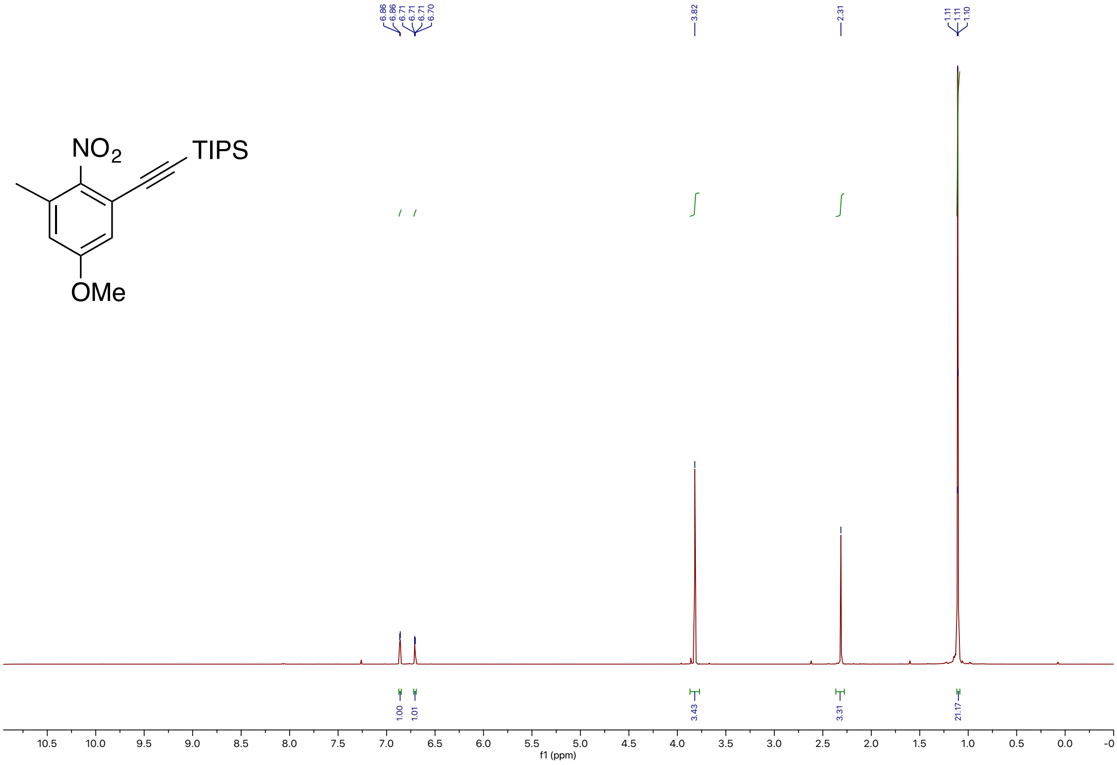

^{13}C NMR: 126 MHz, $CDCl_3$, compound **(3af)**

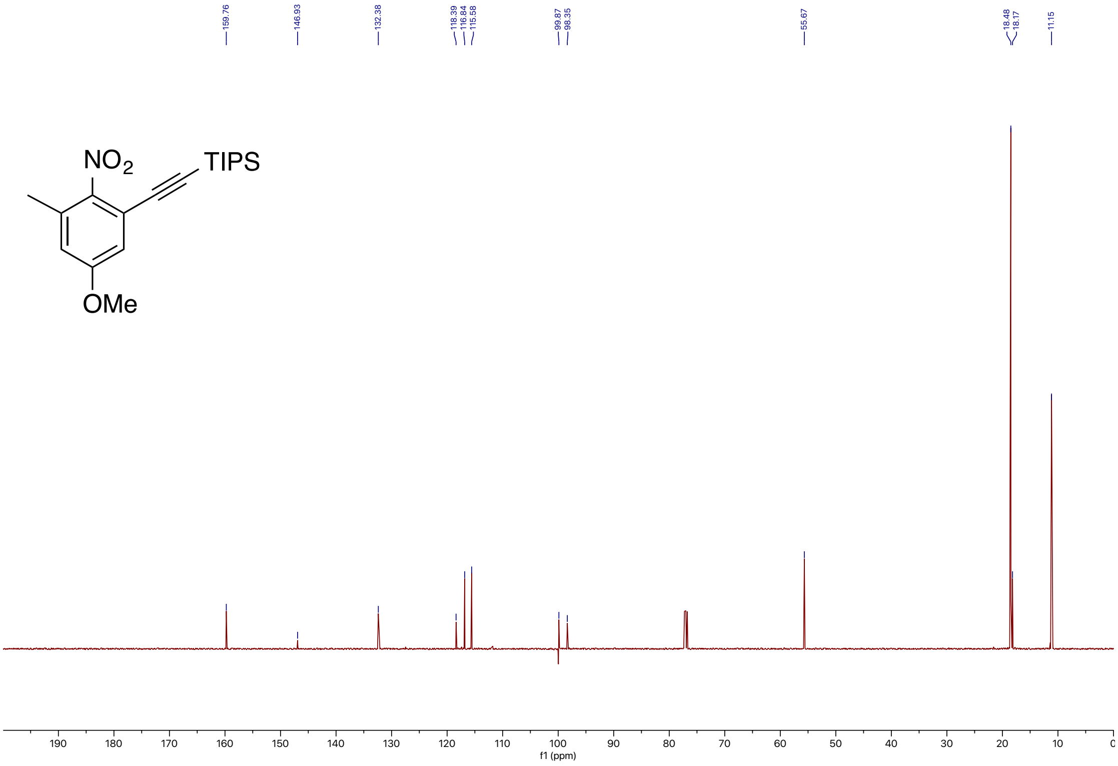

^{1}H NMR: 300 MHz, $CDCl_3$, compound **(3ag)**

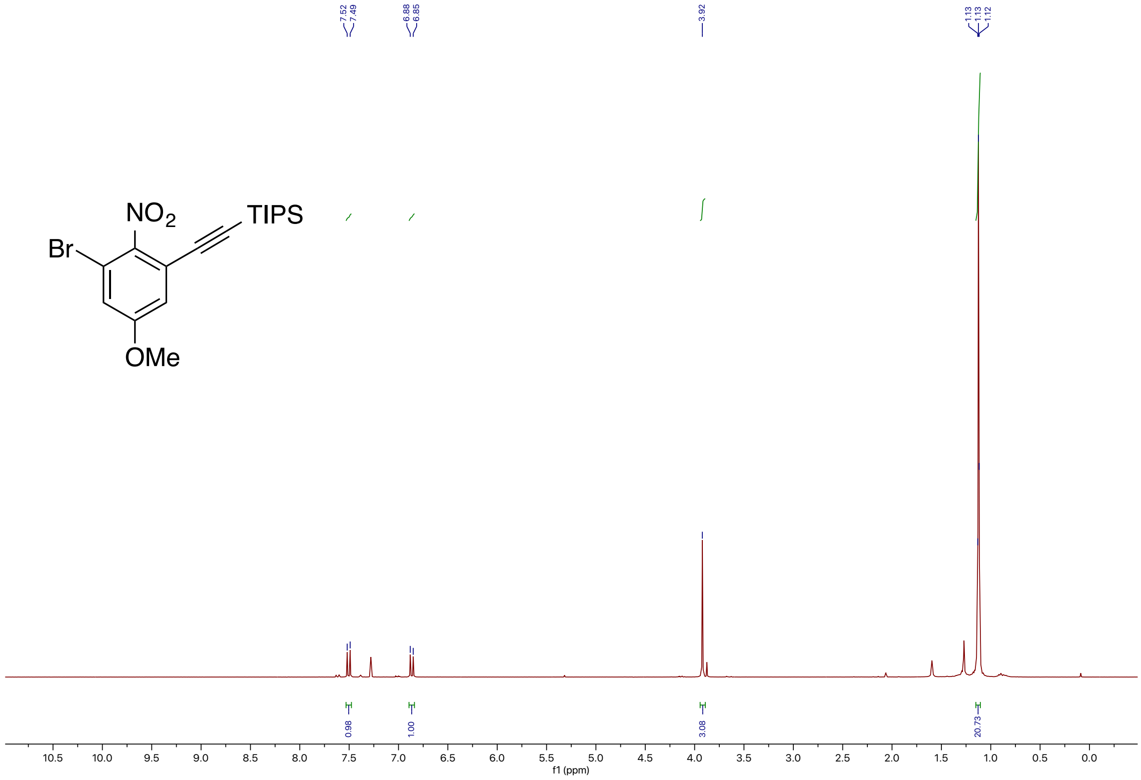

^{13}C NMR: 75 MHz, $CDCl_3$, compound **(3ag)**

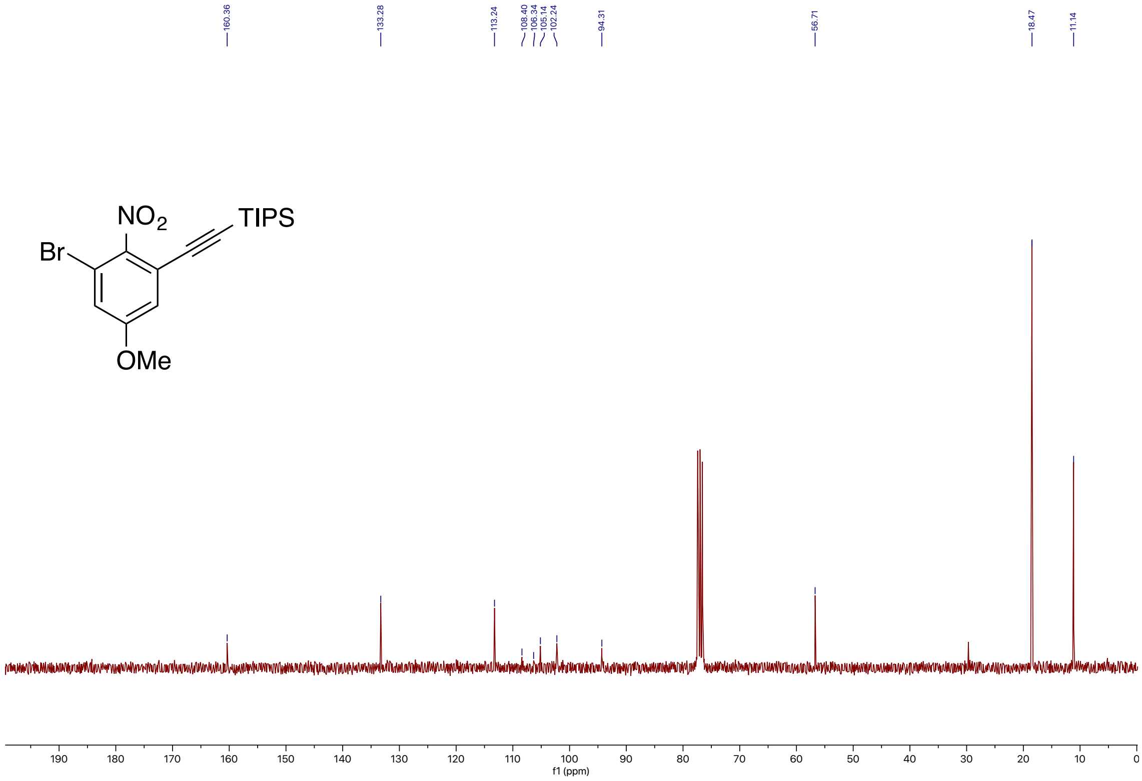

^{1}H NMR: 300 MHz, $CDCl_3$, compound **(3ah)**

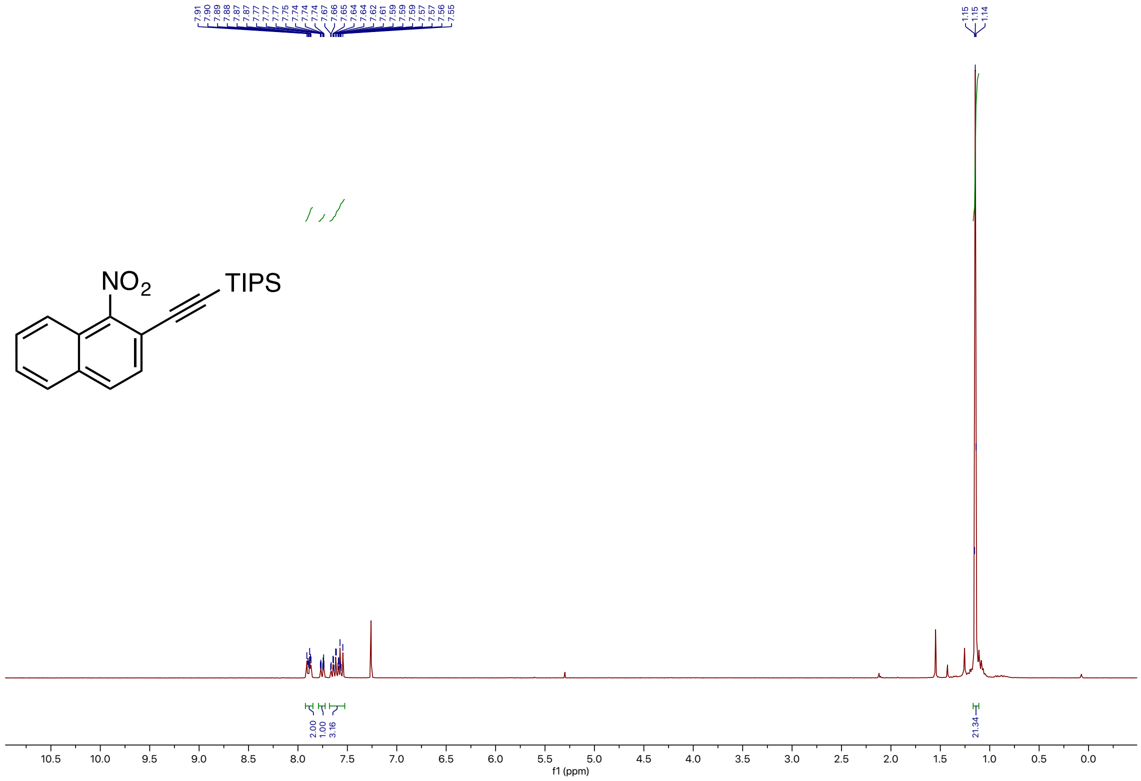

^{13}C NMR: 75 MHz, $CDCl_3$, compound **(3ah)**

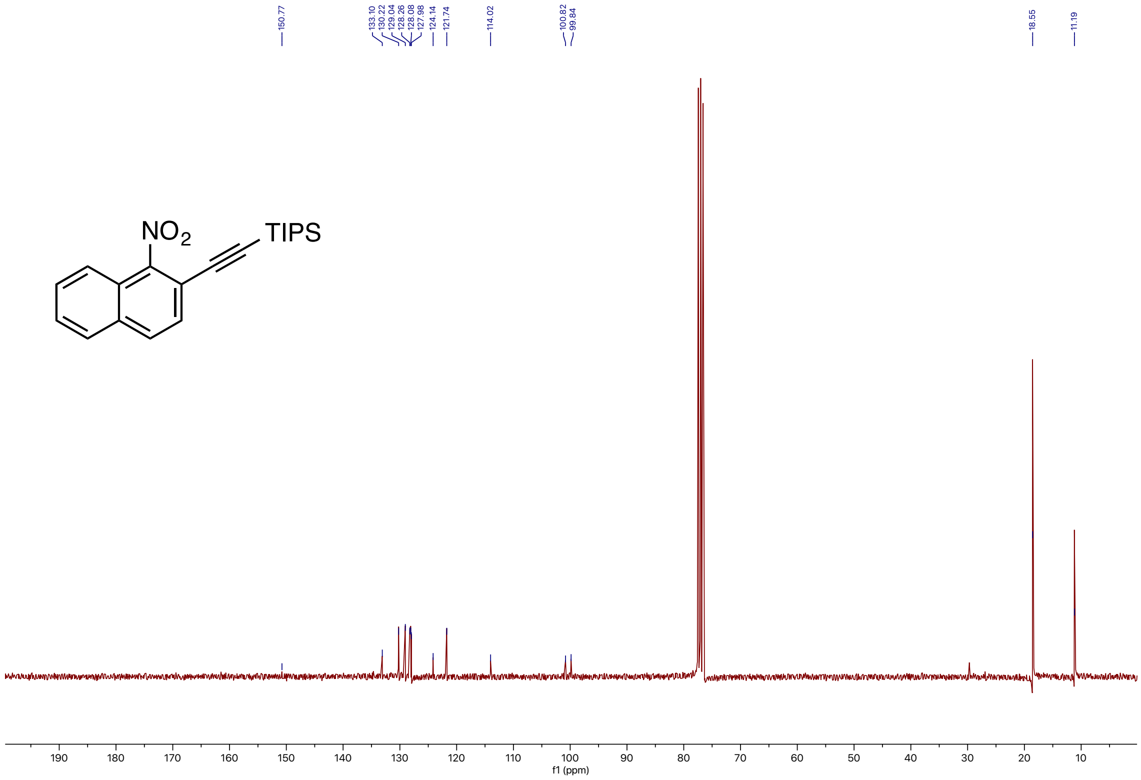

^{1}H NMR: 300 MHz, $CDCl_3$, compound **(3ai)**

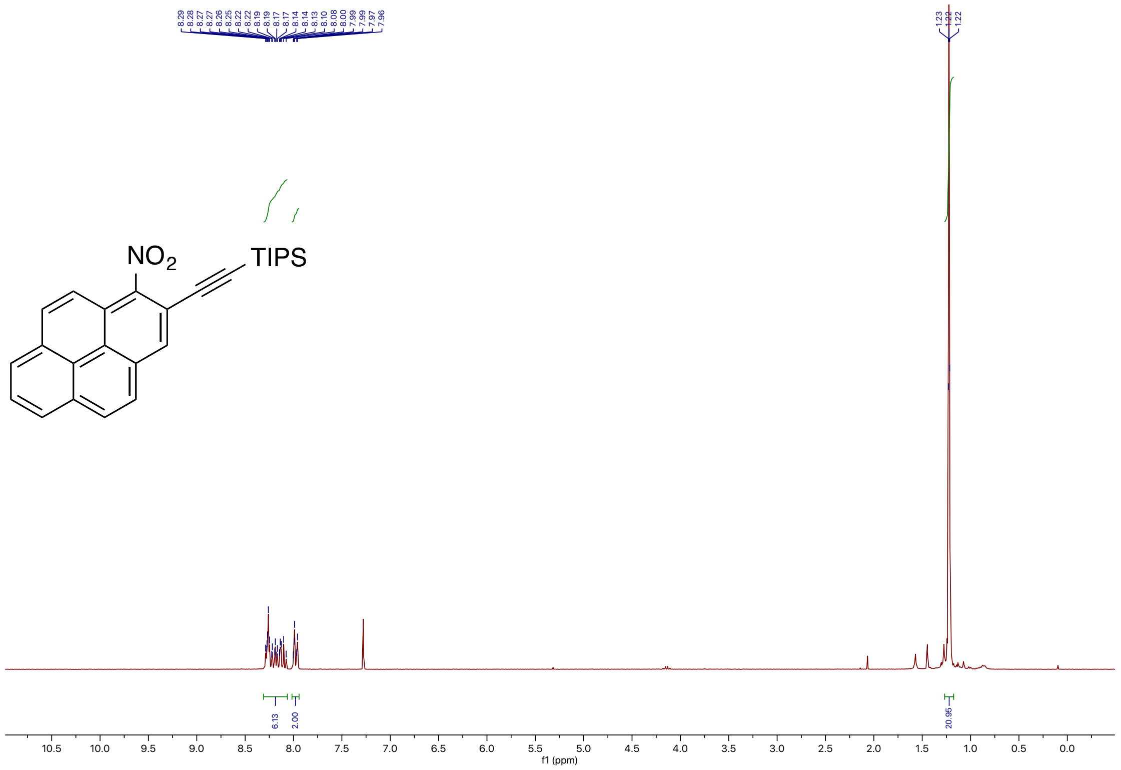

^{13}C NMR: 75 MHz, $CDCl_3$, compound **(3ai)**

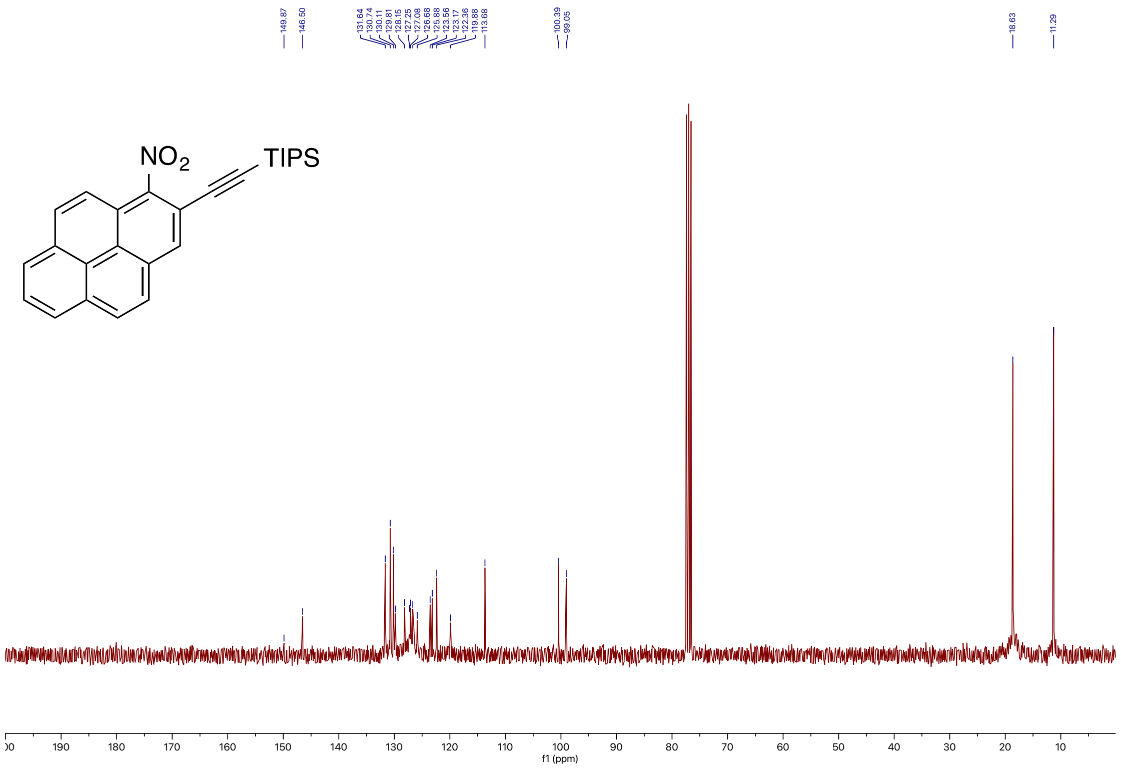

^{1}H NMR: 500 MHz, $CDCl_3$, compound (**3aj**)

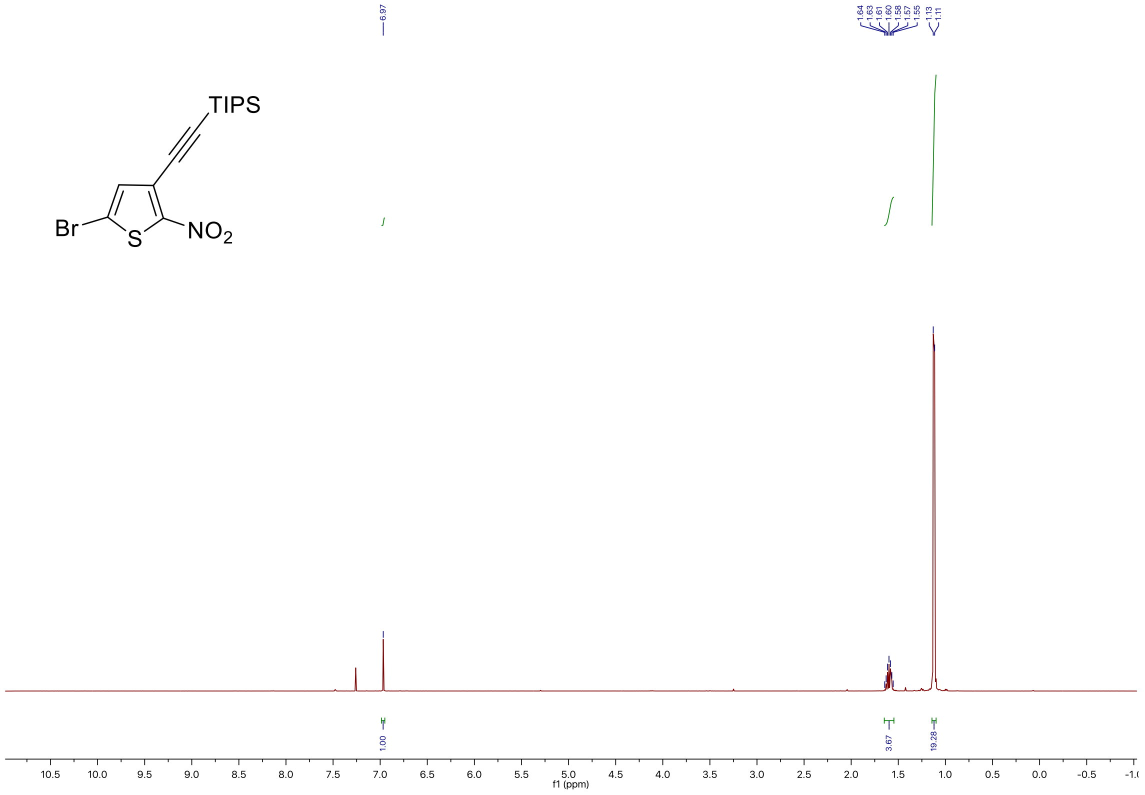

^{13}C NMR: 126 MHz, $CDCl_3$, compound (**3aj**)

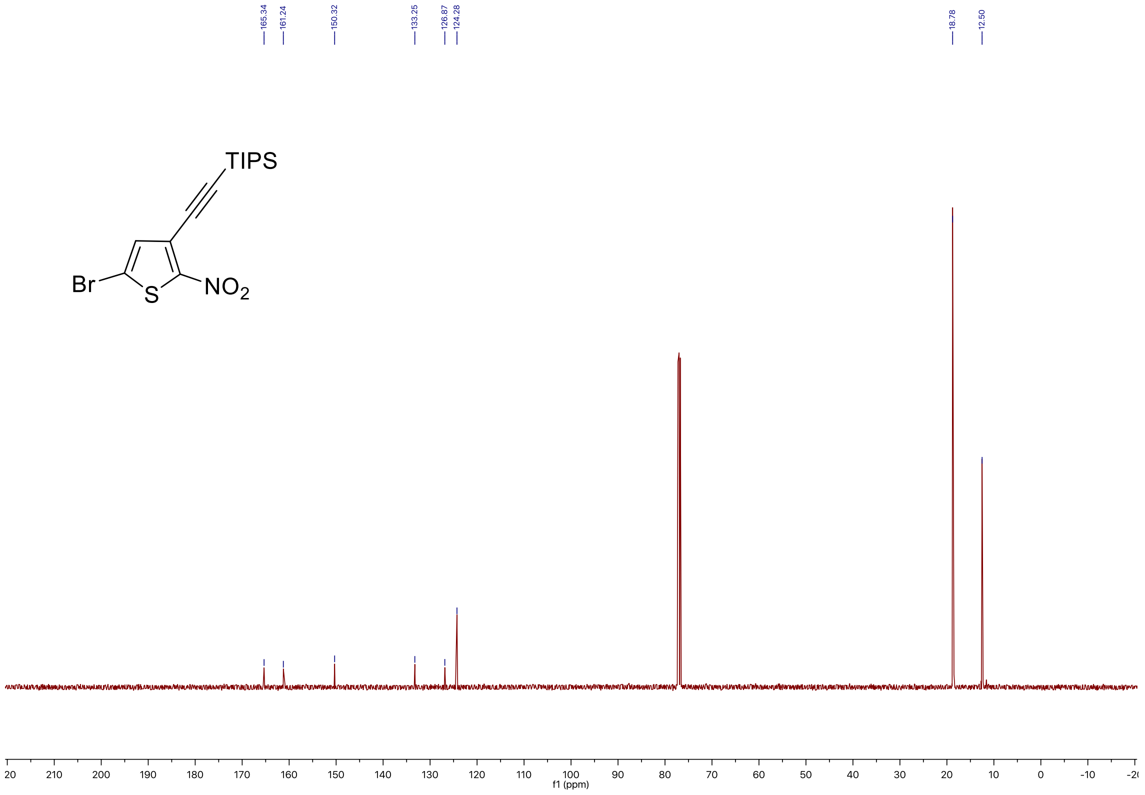

^{1}H NMR: 500 MHz, $CDCl_3$, compound (**3ak**)

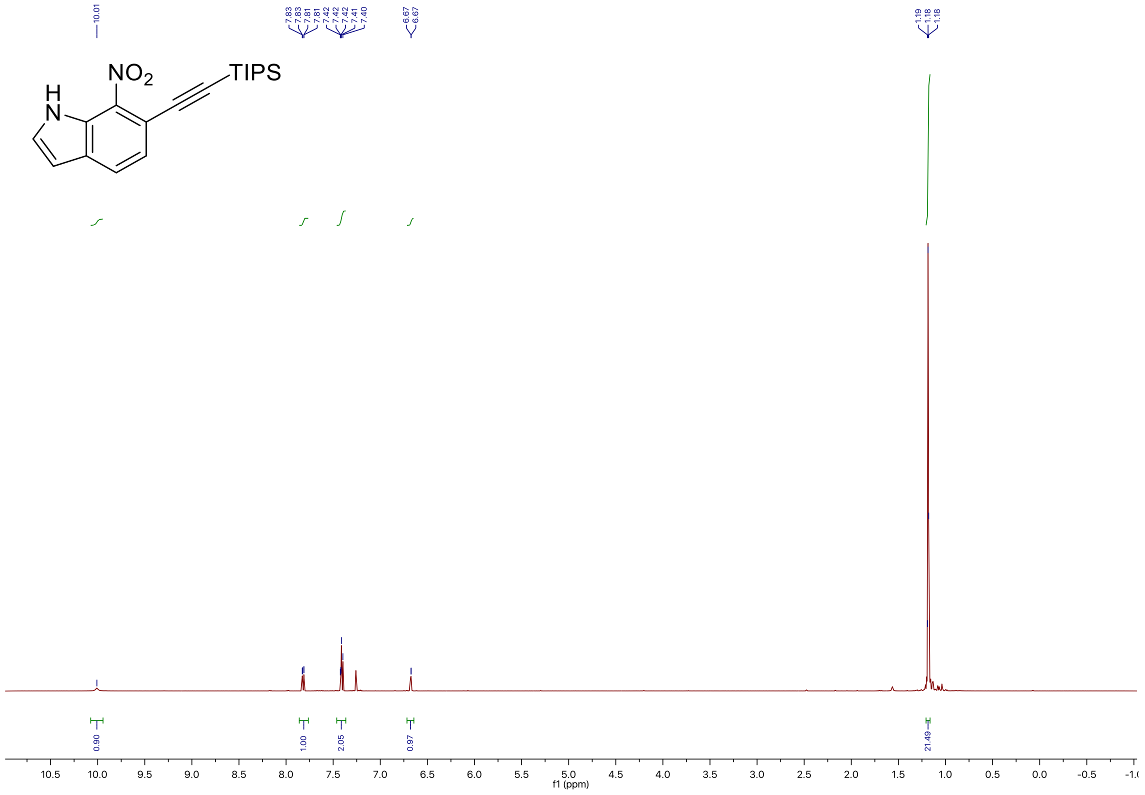

^{13}C NMR: 126 MHz, $CDCl_3$, compound (**3a**k

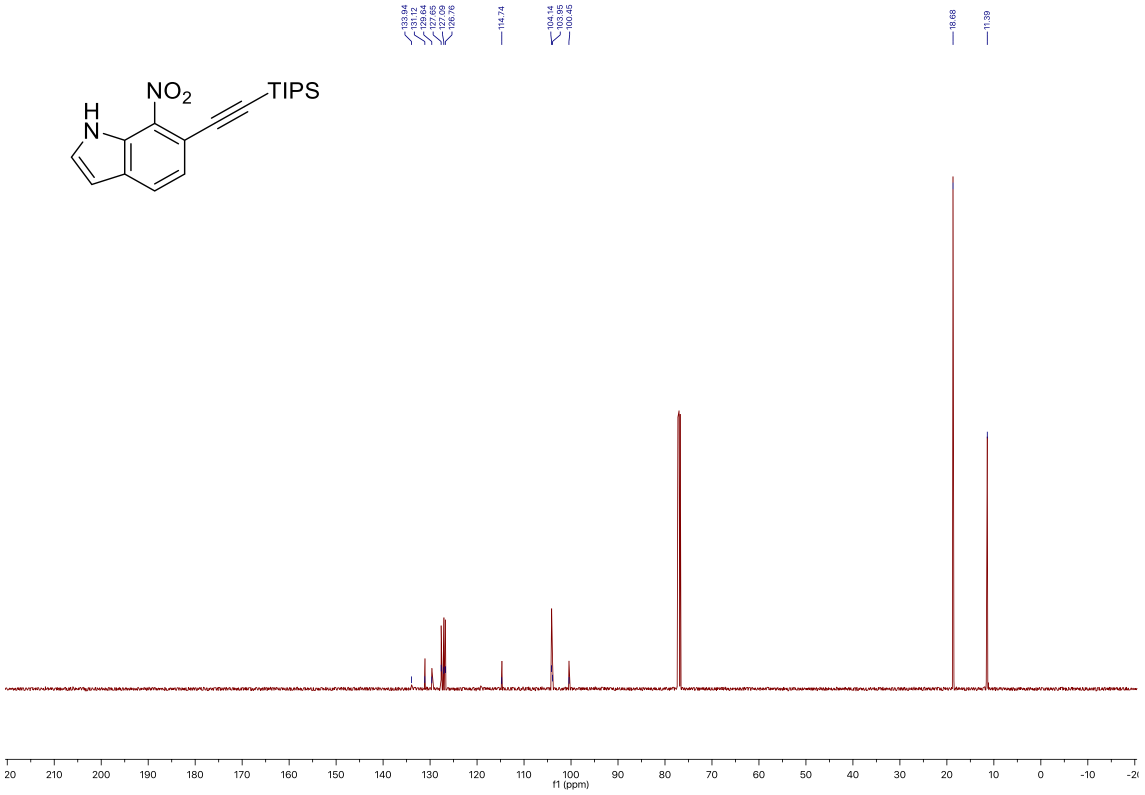

^{1}H NMR: 300 MHz, $CDCl_3$, compound (**3al**)

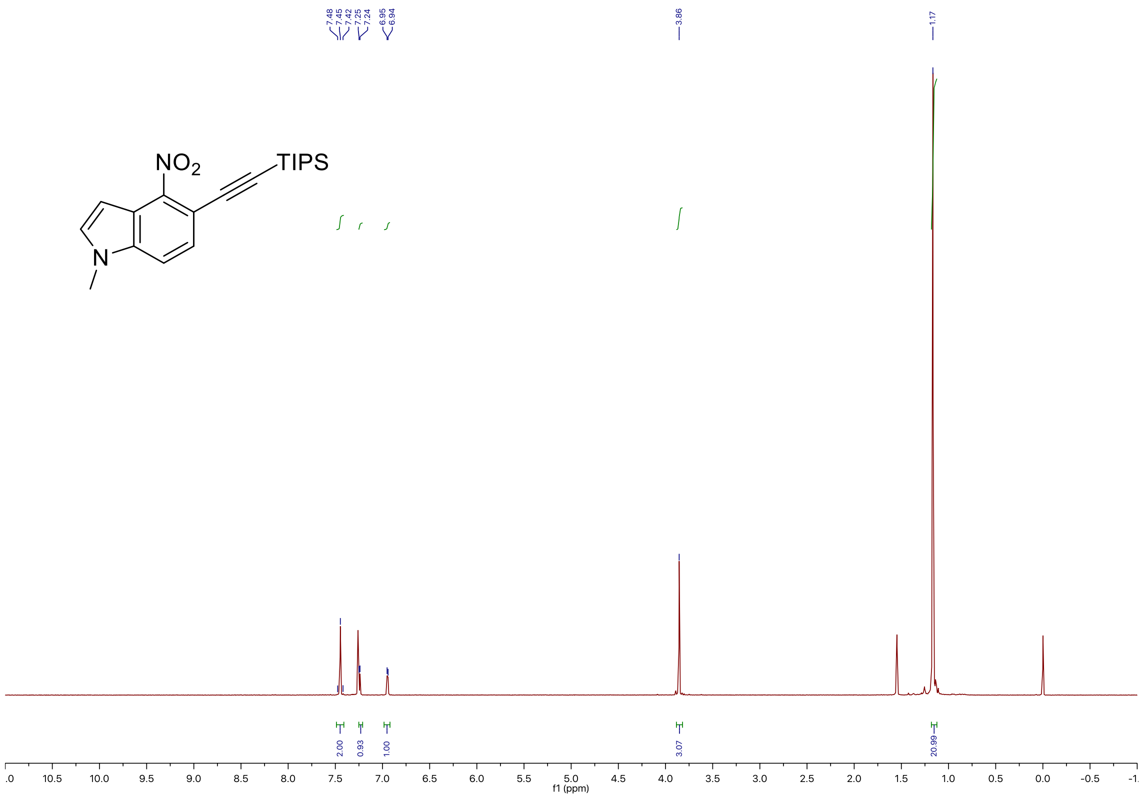

^{13}C NMR: 126 MHz, $CDCl_3$, compound (**3al**)

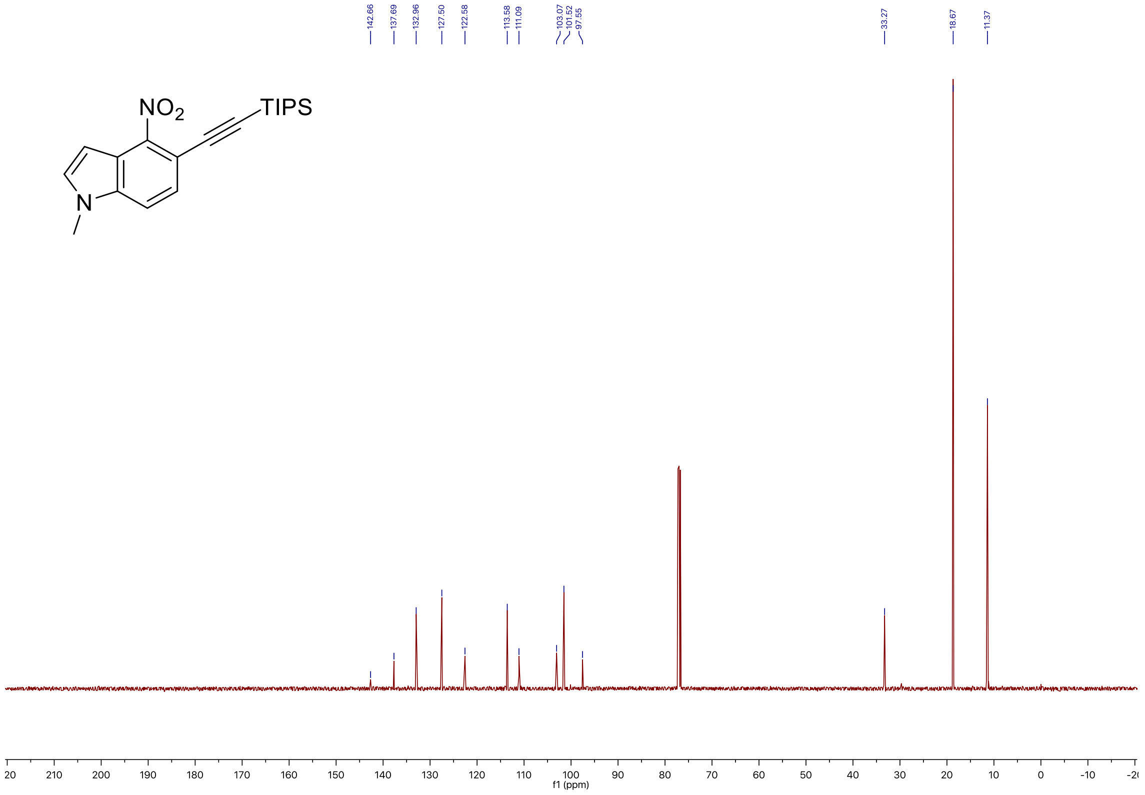

^{1}H NMR: 500 MHz, $CDCl_3$, compound (**3am**)

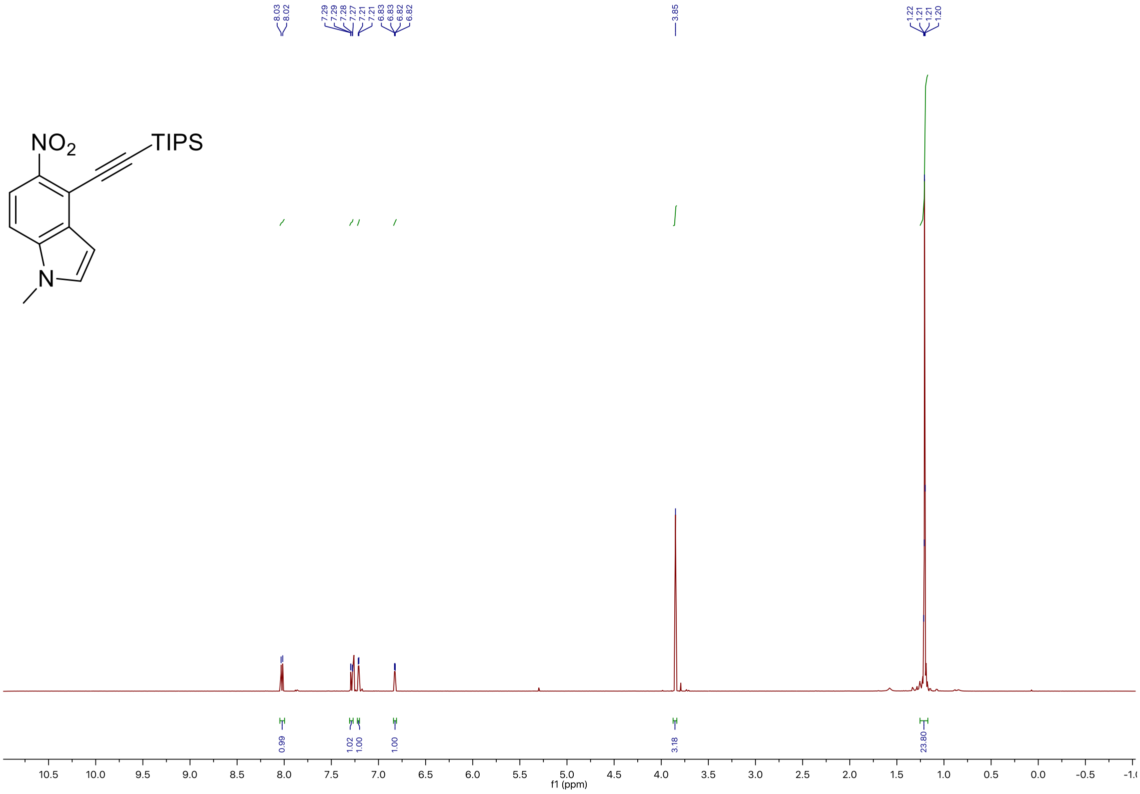

^{13}C NMR: 126 MHz, $CDCl_3$, compound (**3am**)

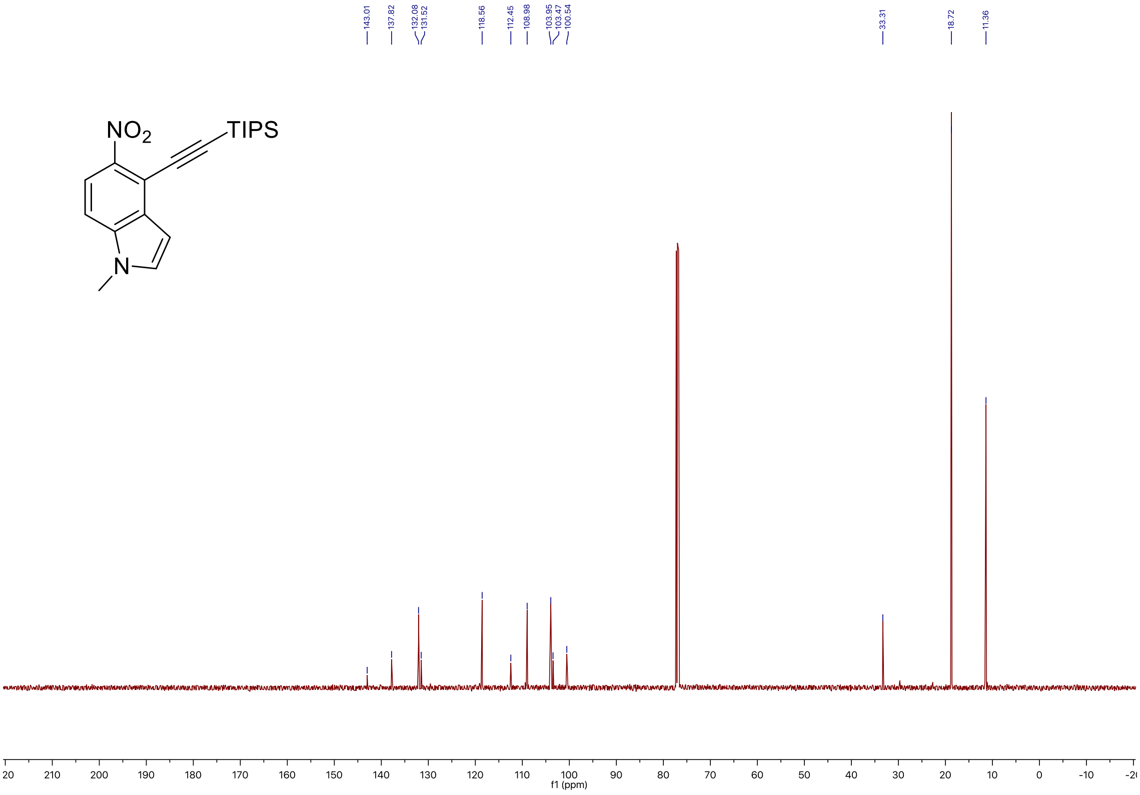

^{1}H NMR: 500 MHz, $CDCl_3$, compound (**3am'**)

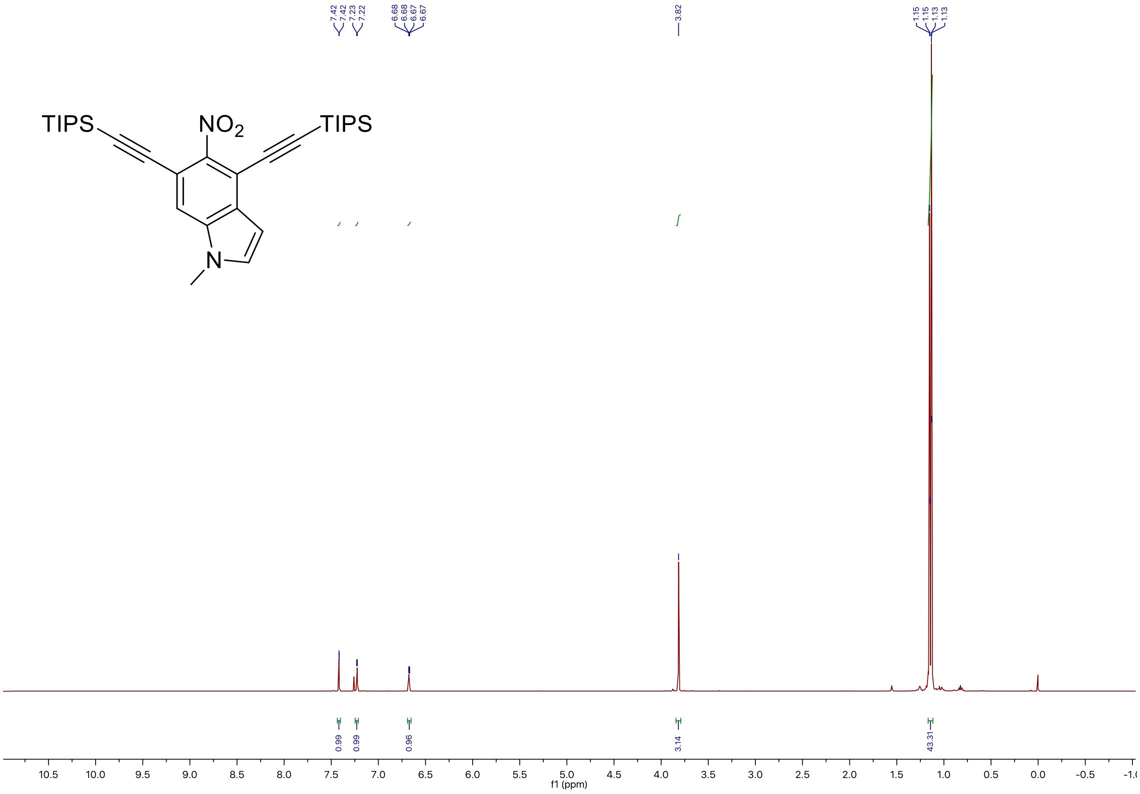

^{13}C NMR: 126 MHz, $CDCl_3$, compound (**3am'**)

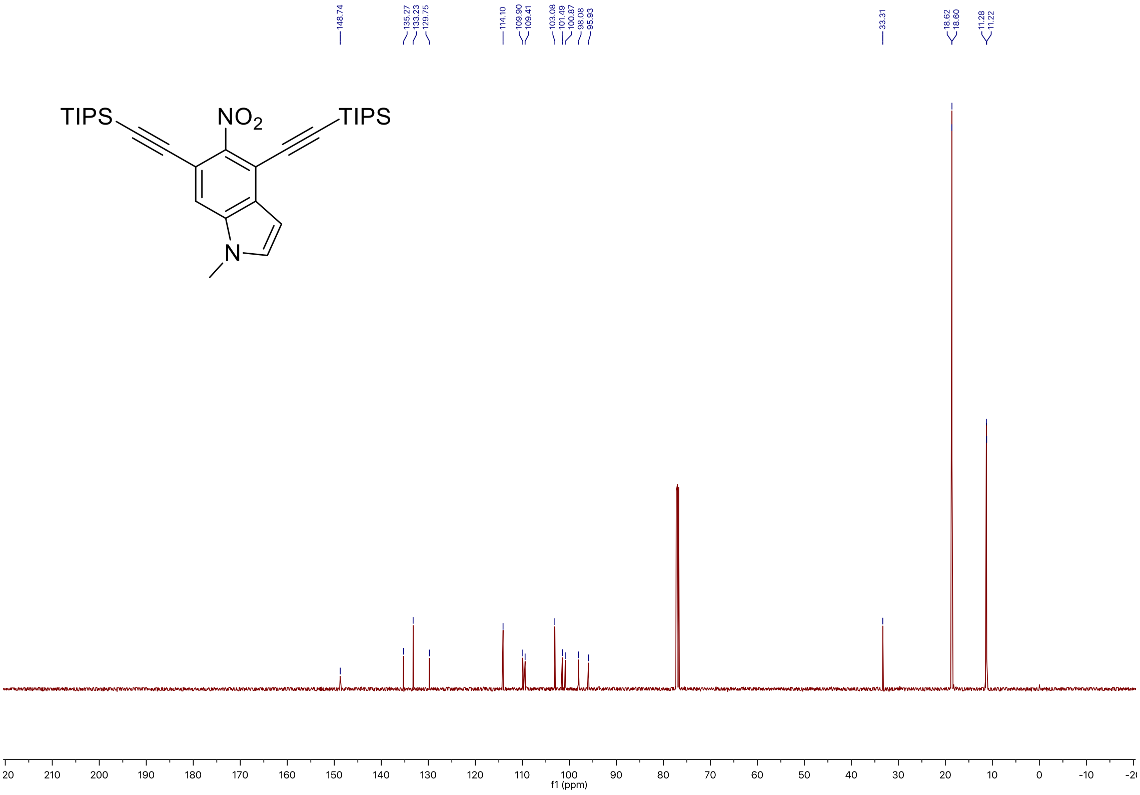

^{1}H NMR: 400 MHz, $CDCl_3$, compound (**3an**)

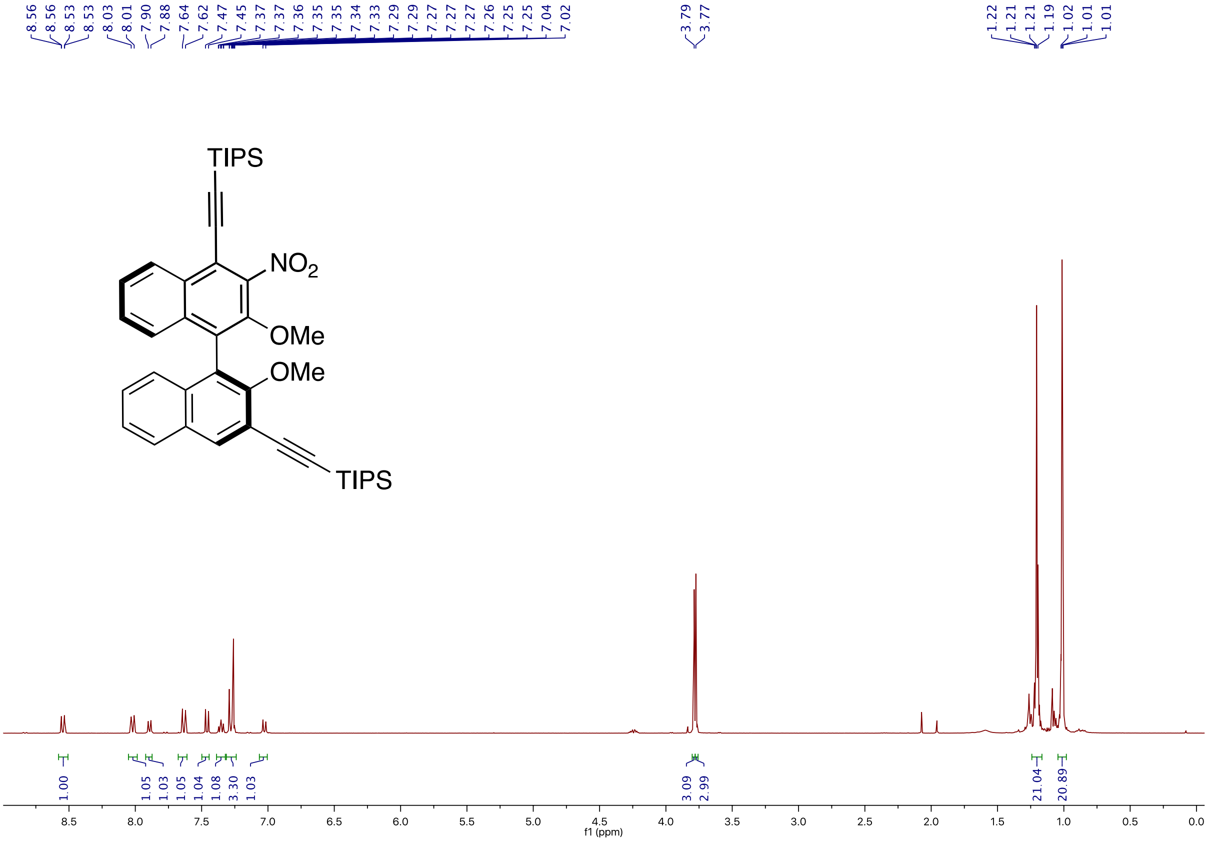

^{13}C NMR: 101 MHz, $CDCl_3$, compound (**3an**)

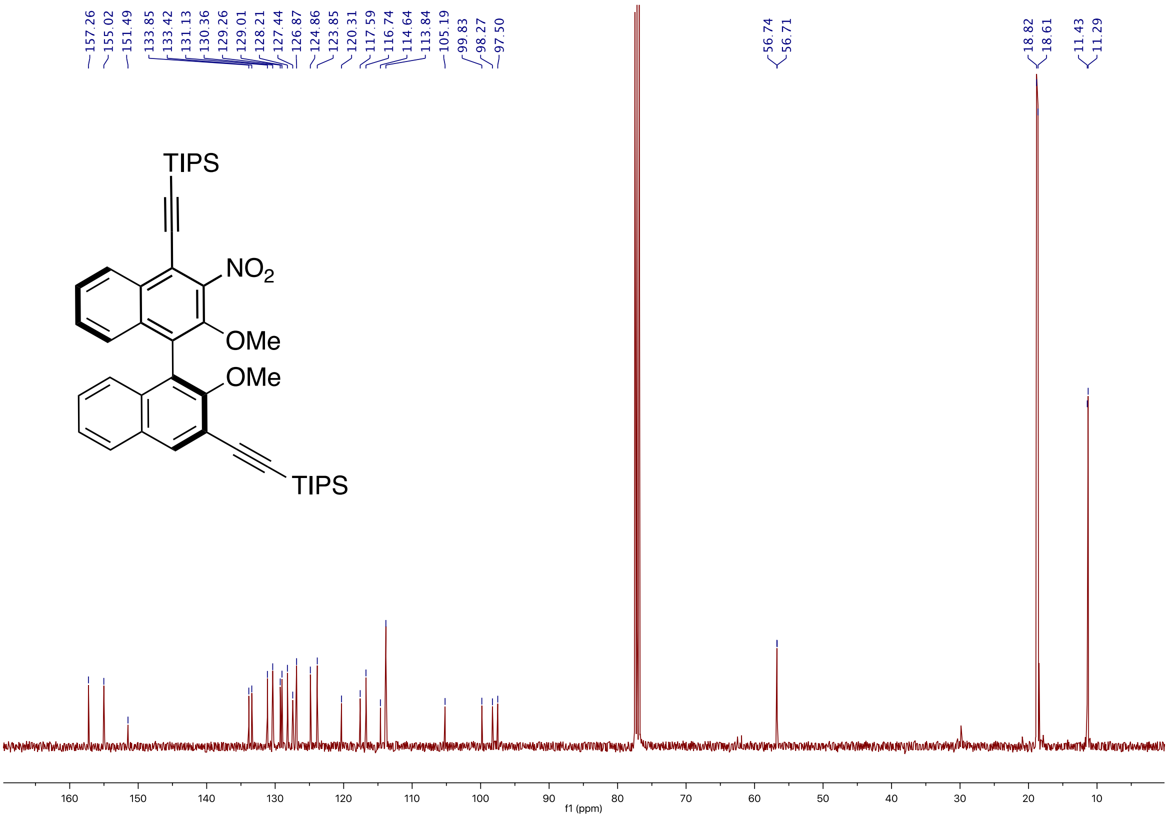

^{1}H NMR: 500 MHz, $CDCl_3$, compound **(3ap)**

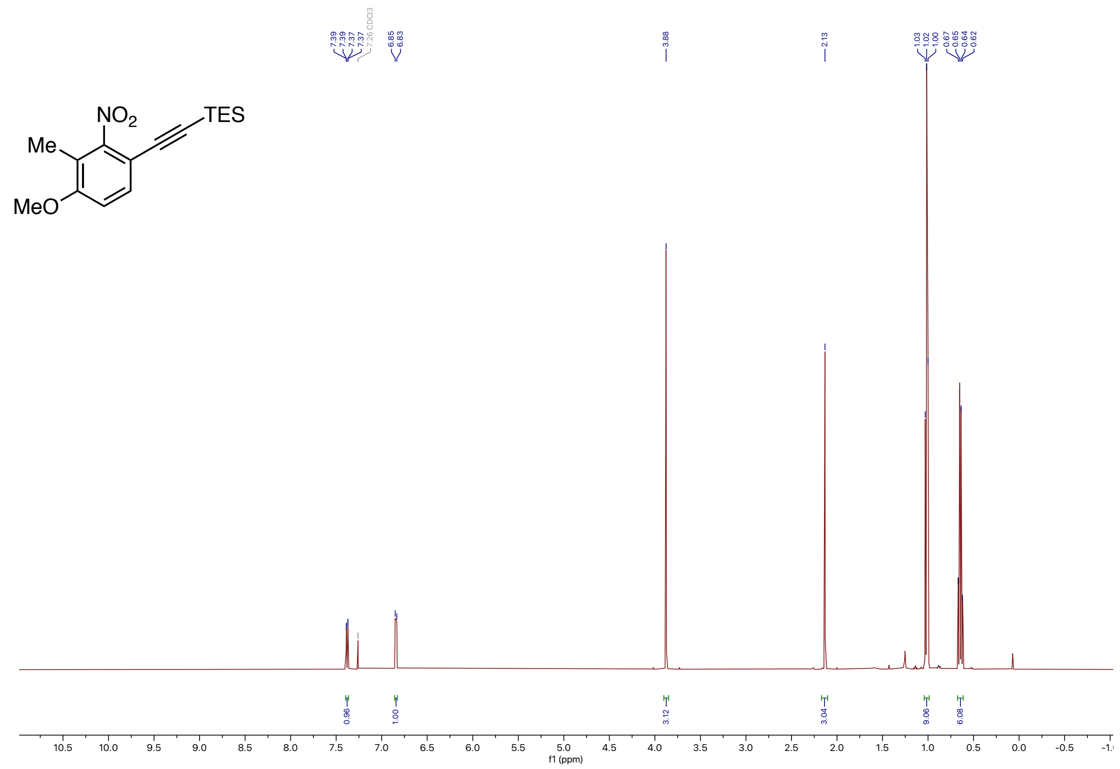

^{13}C NMR: 126 MHz, $CDCl_3$, compound **(3ap)**

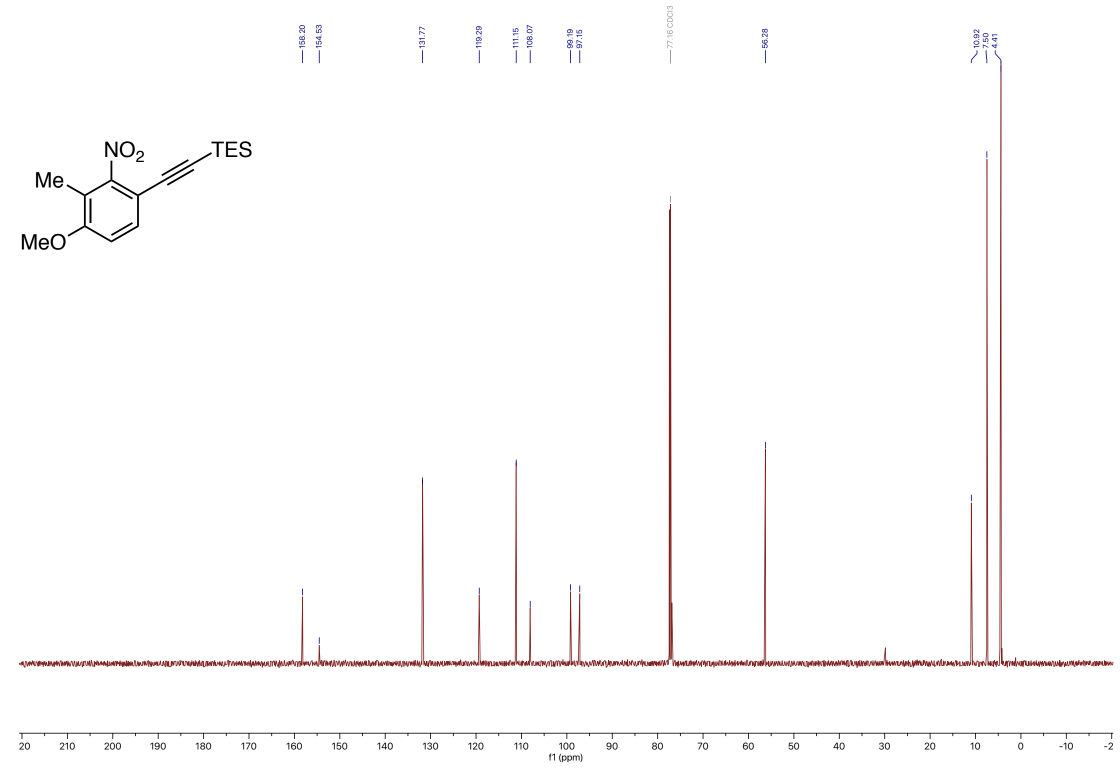

^{1}H NMR: 500 MHz, $CDCl_3$, compound **(3aq)**

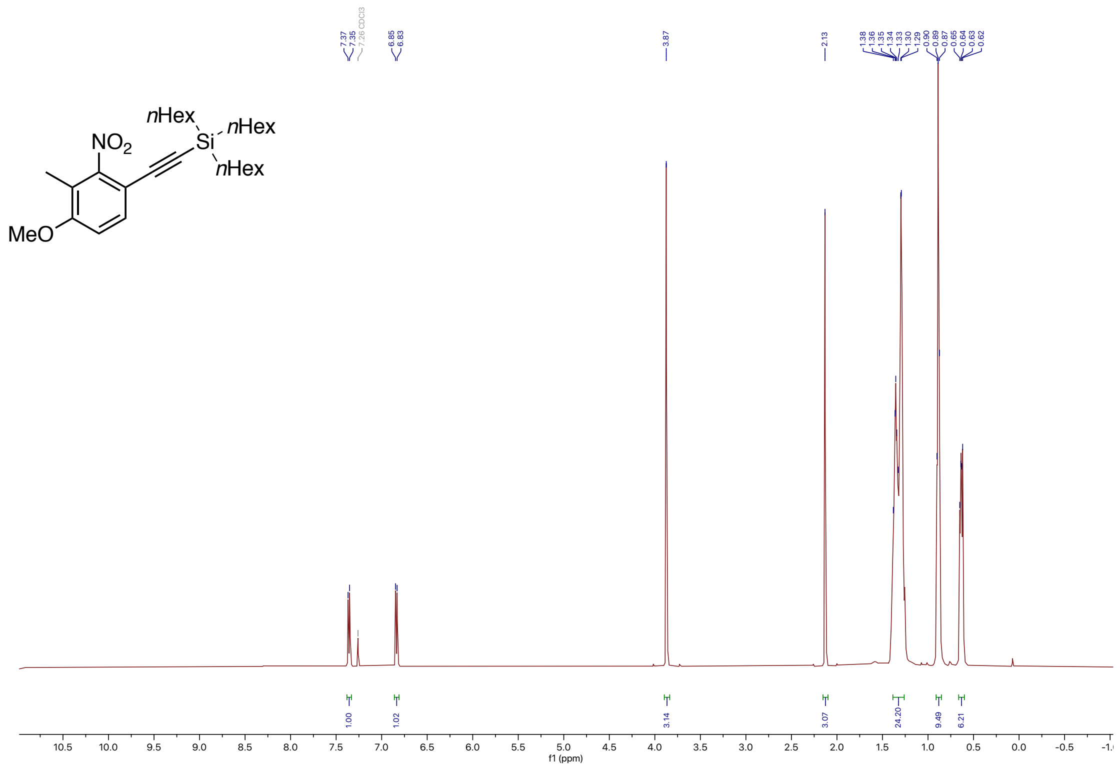

^{13}C NMR: 126 MHz, $CDCl_3$, compound **(3aq)**

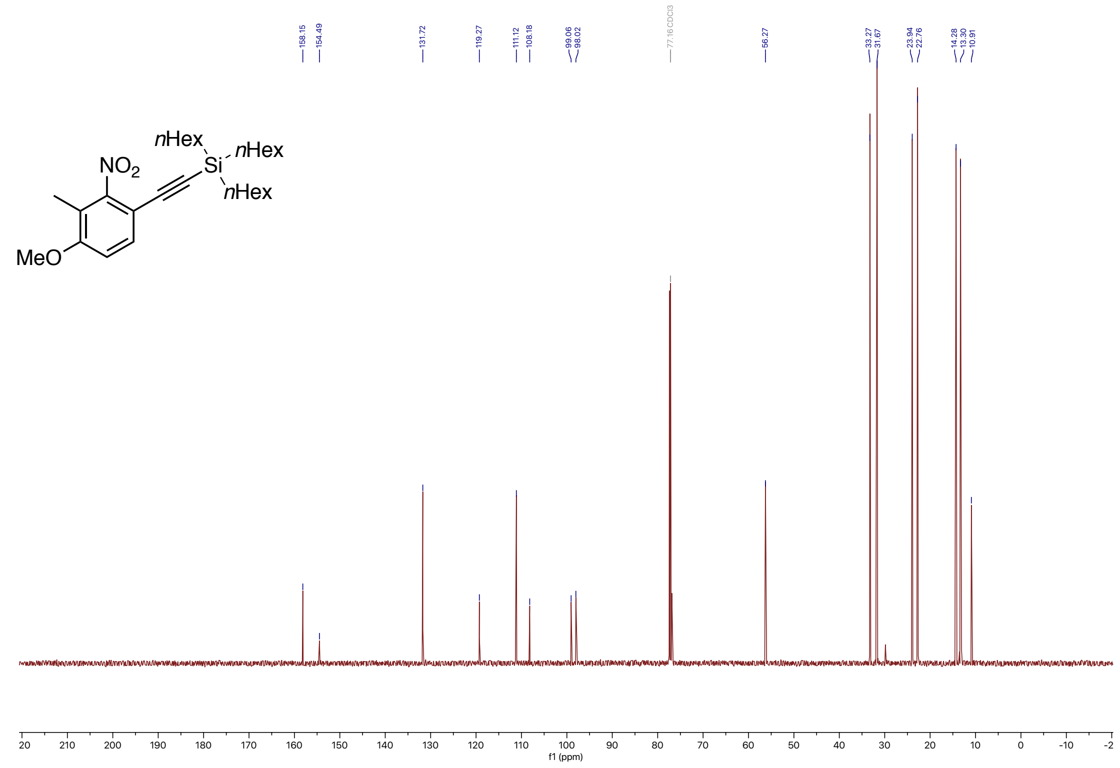

^{1}H NMR: 500 MHz, $CDCl_3$, compound **(3ar)**

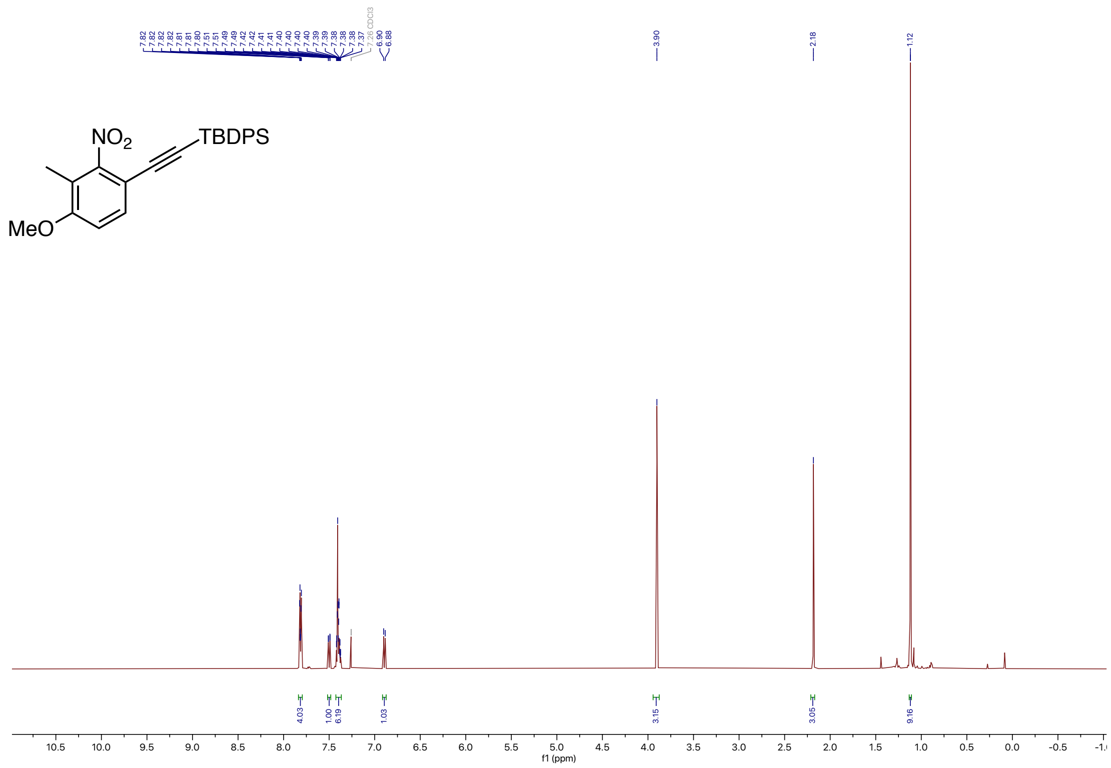

^{13}C NMR: 126 MHz, $CDCl_3$, compound **(3ar)**

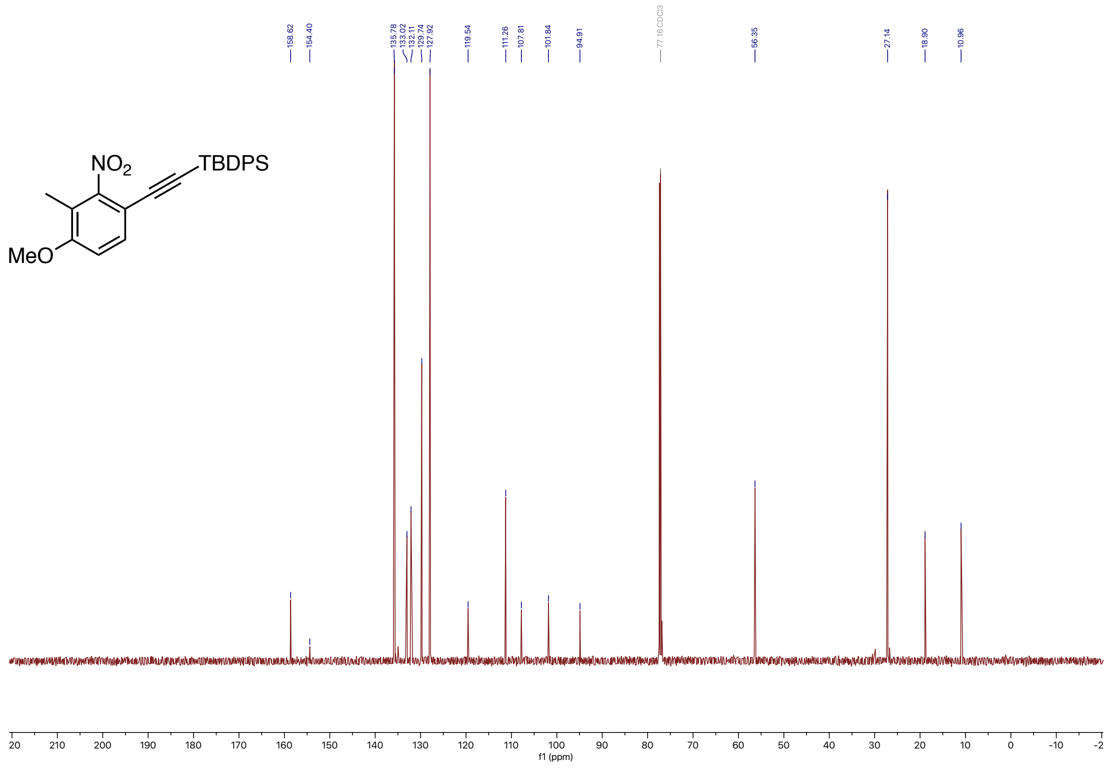

^{1}H NMR: 500 MHz, $CDCl_3$, compound **(3as)**

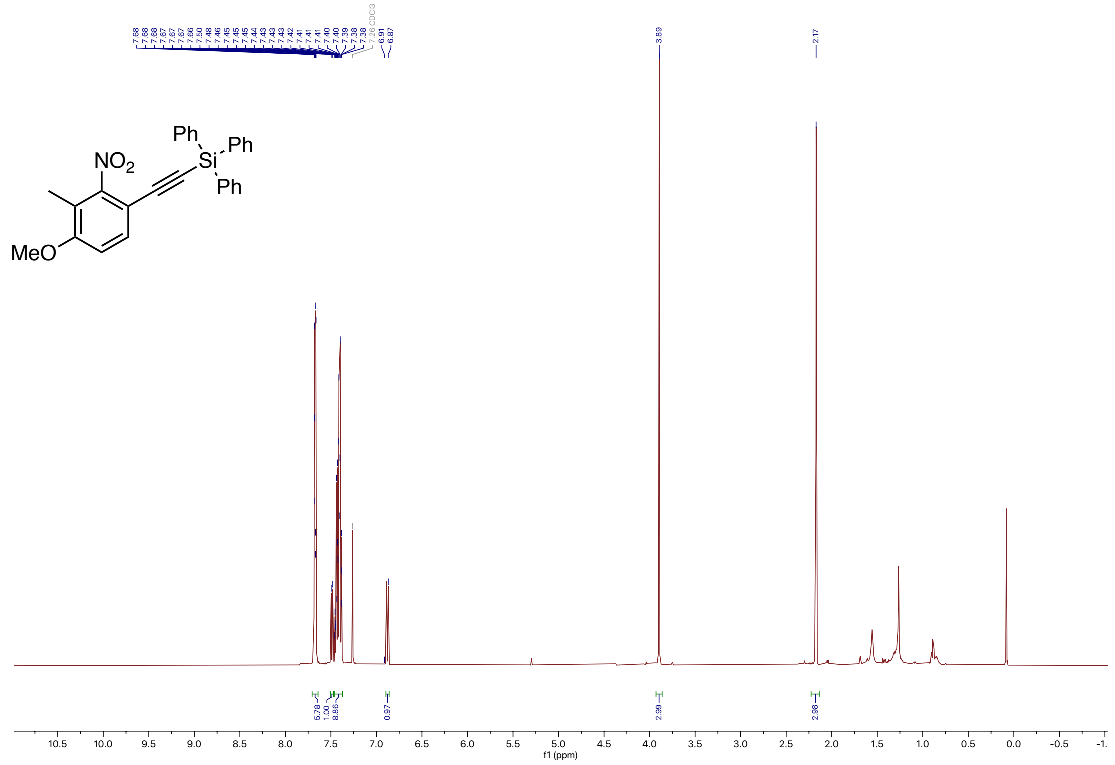

^{13}C NMR: 126 MHz, $CDCl_3$, compound **(3as)**

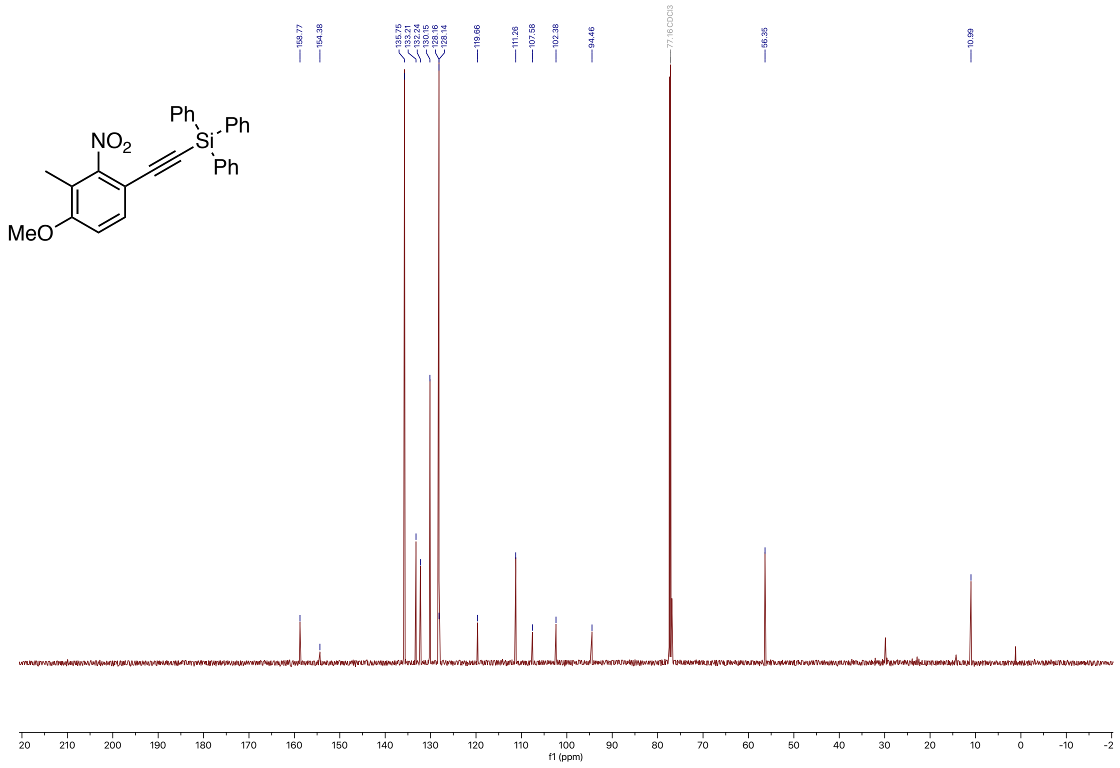

^{1}H NMR: 400 MHz, $CDCl_3$, compound **(6)**

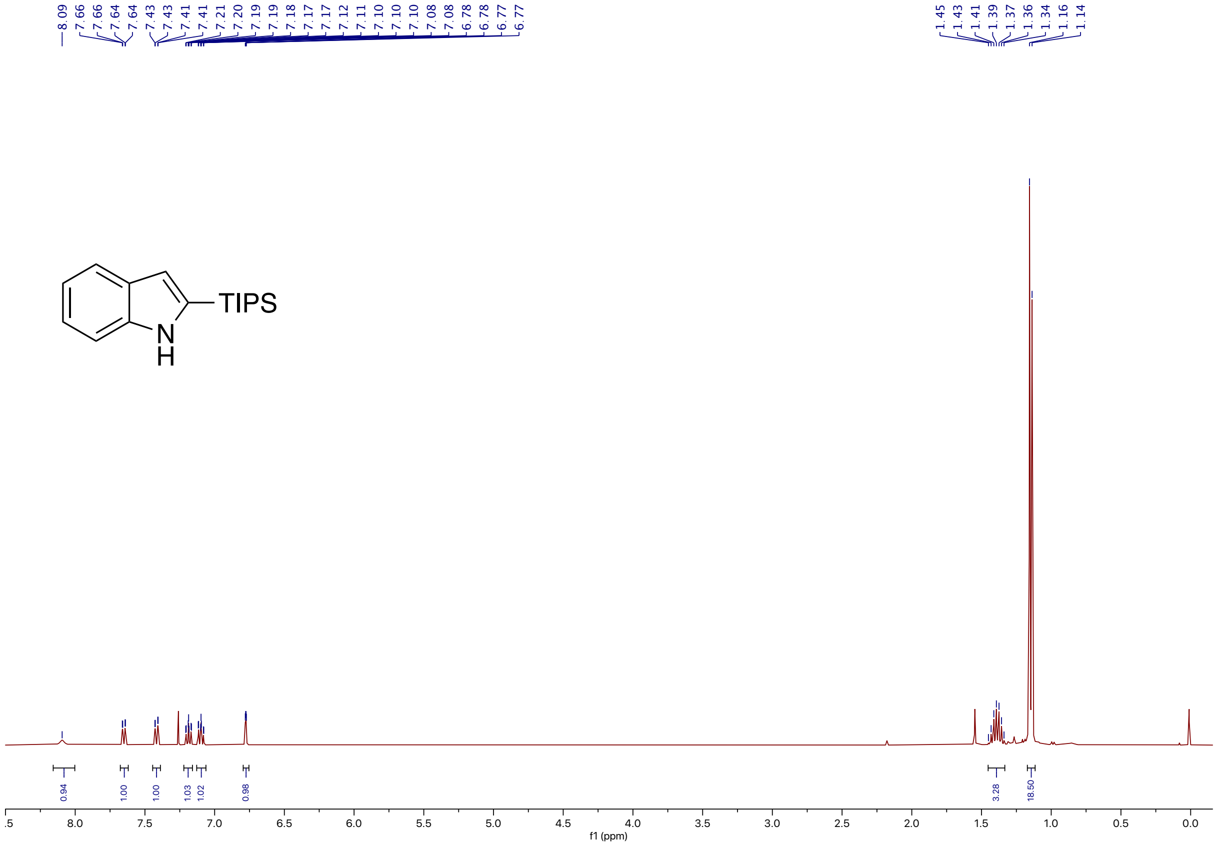

^{13}C NMR: 101 MHz, $CDCl_3$, compound **(6)**

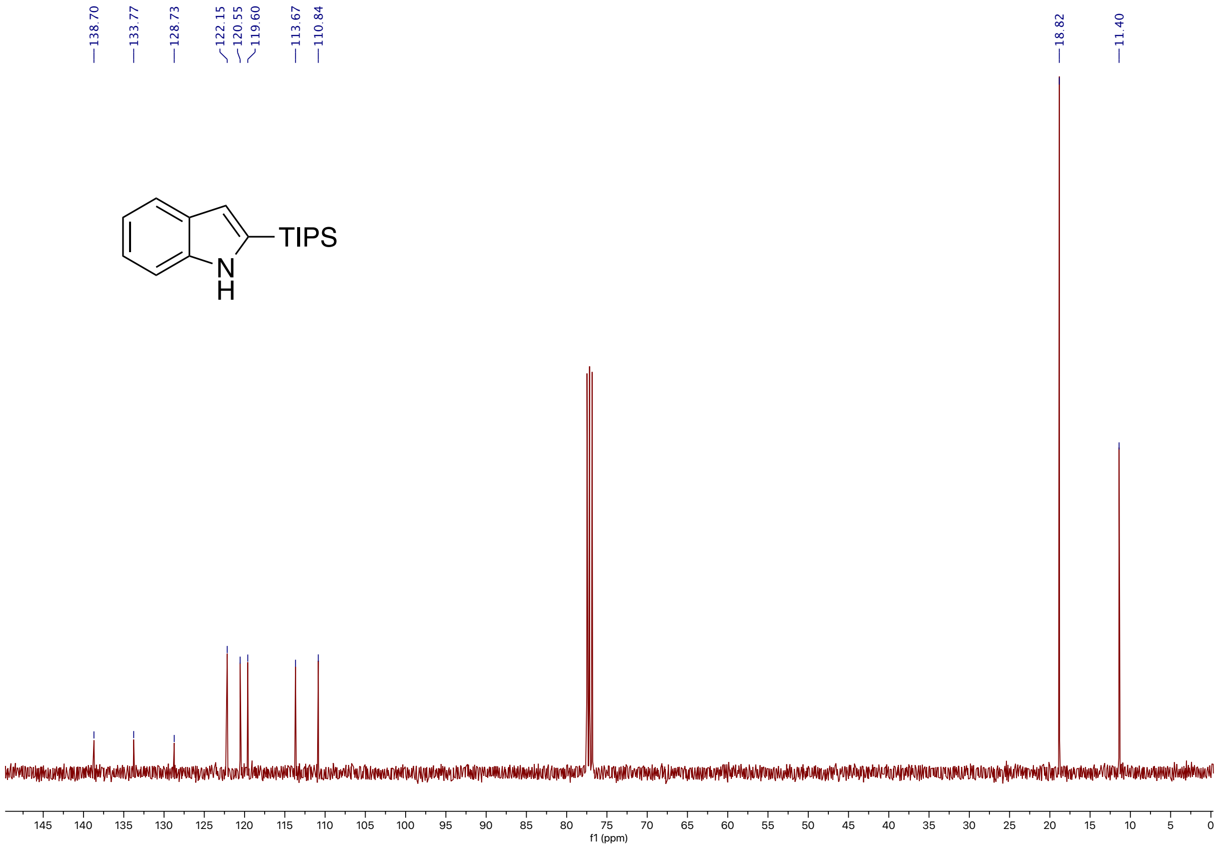

^1H NMR: 500 MHz, $CDCl_3$, compound (**7**)

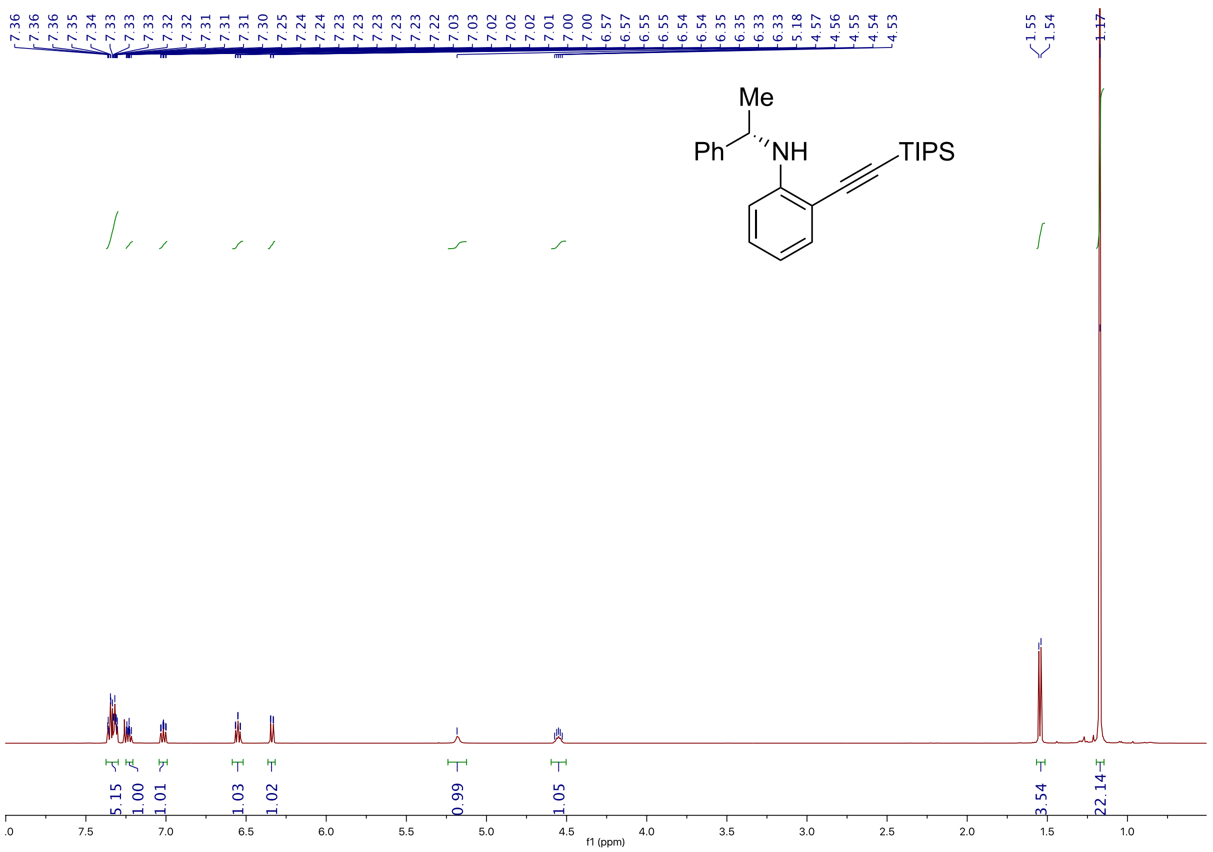

^{13}C NMR: 126 MHz, $CDCl_3$, compound (**7**)

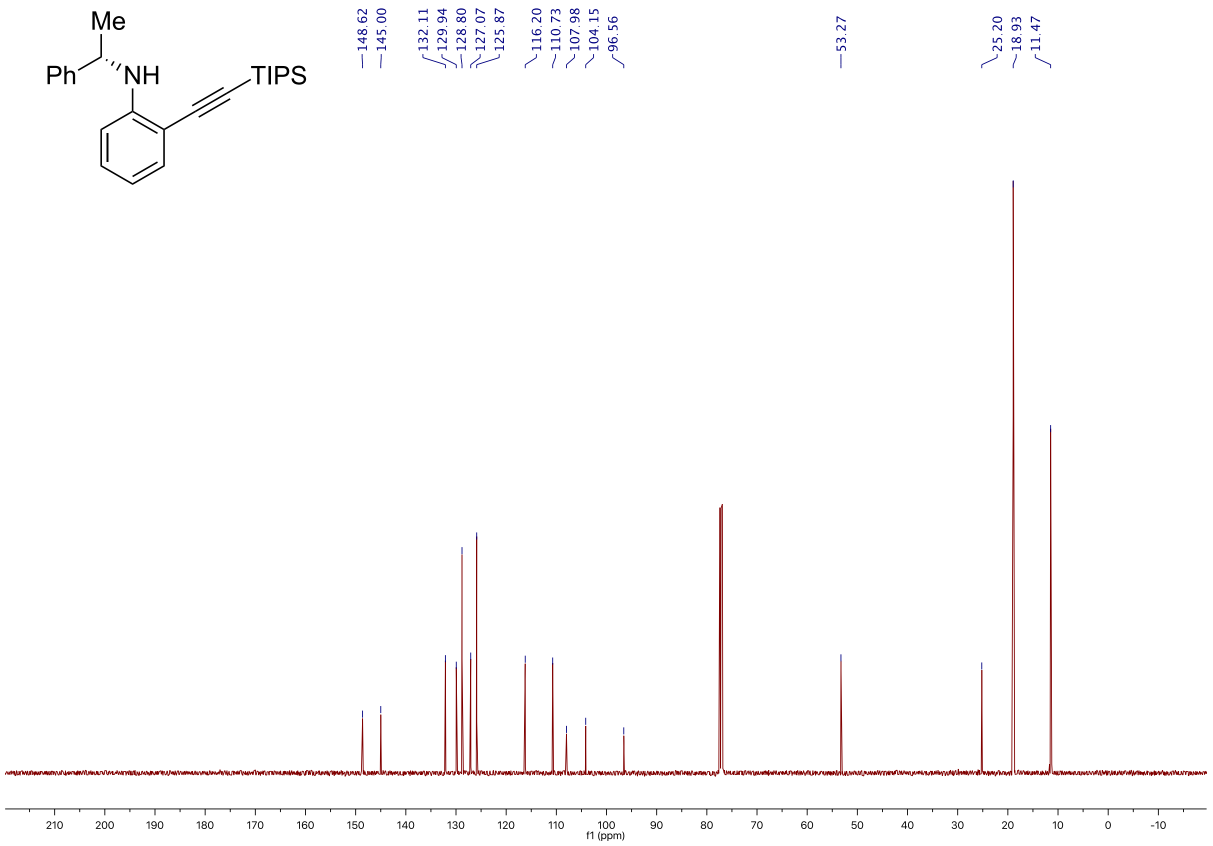

^{1}H NMR: 400 MHz, $CDCl_3$, compound (**8**)

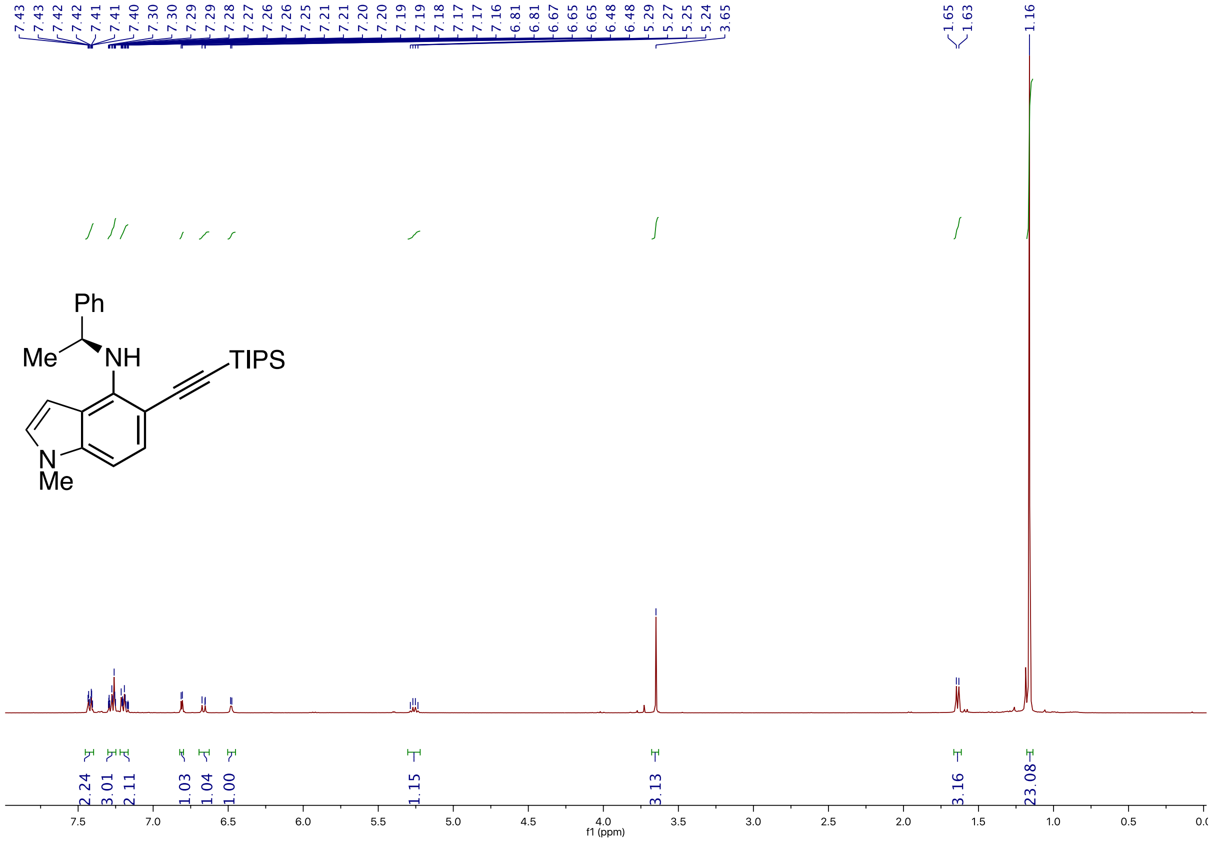

^{13}C NMR: 126 MHz, $CDCl_3$, compound (**8**)

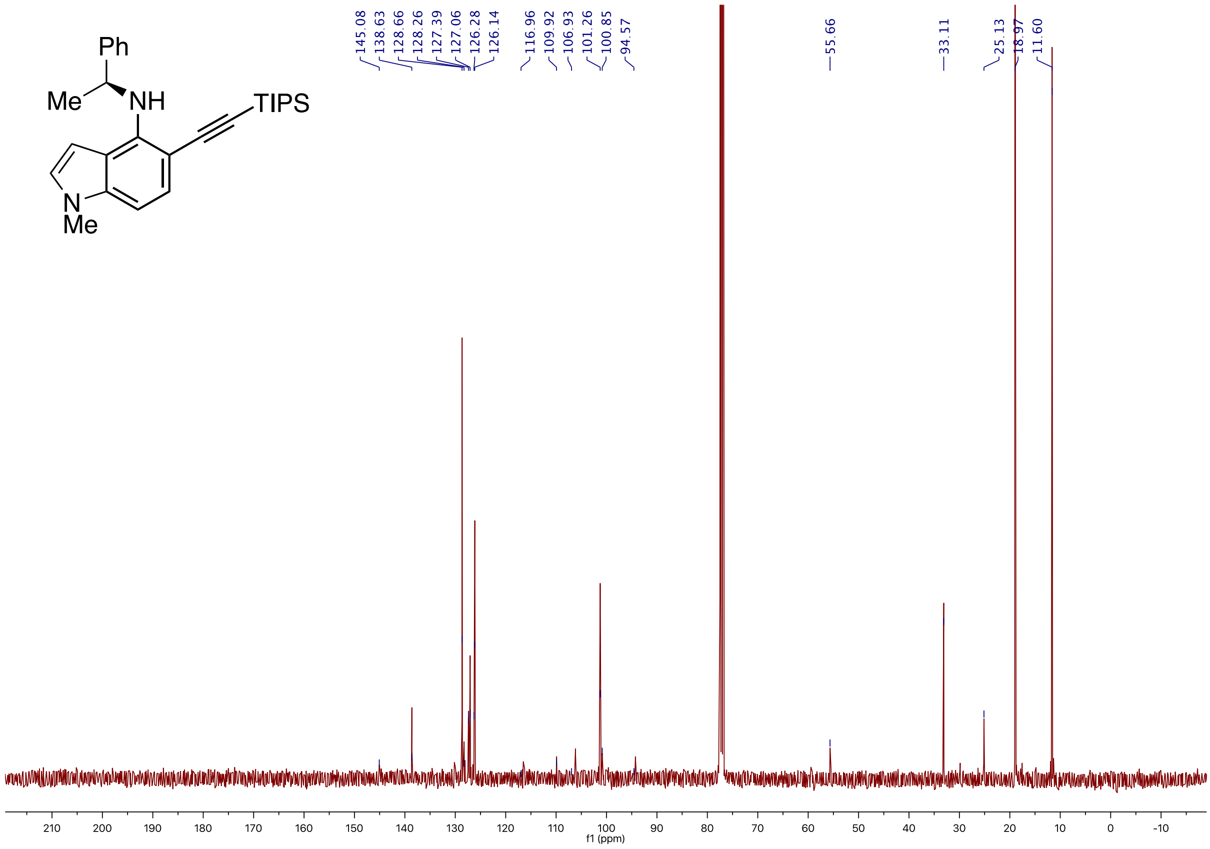

^{1}H NMR: 500 MHz, $CDCl_3$, compound (**9**)

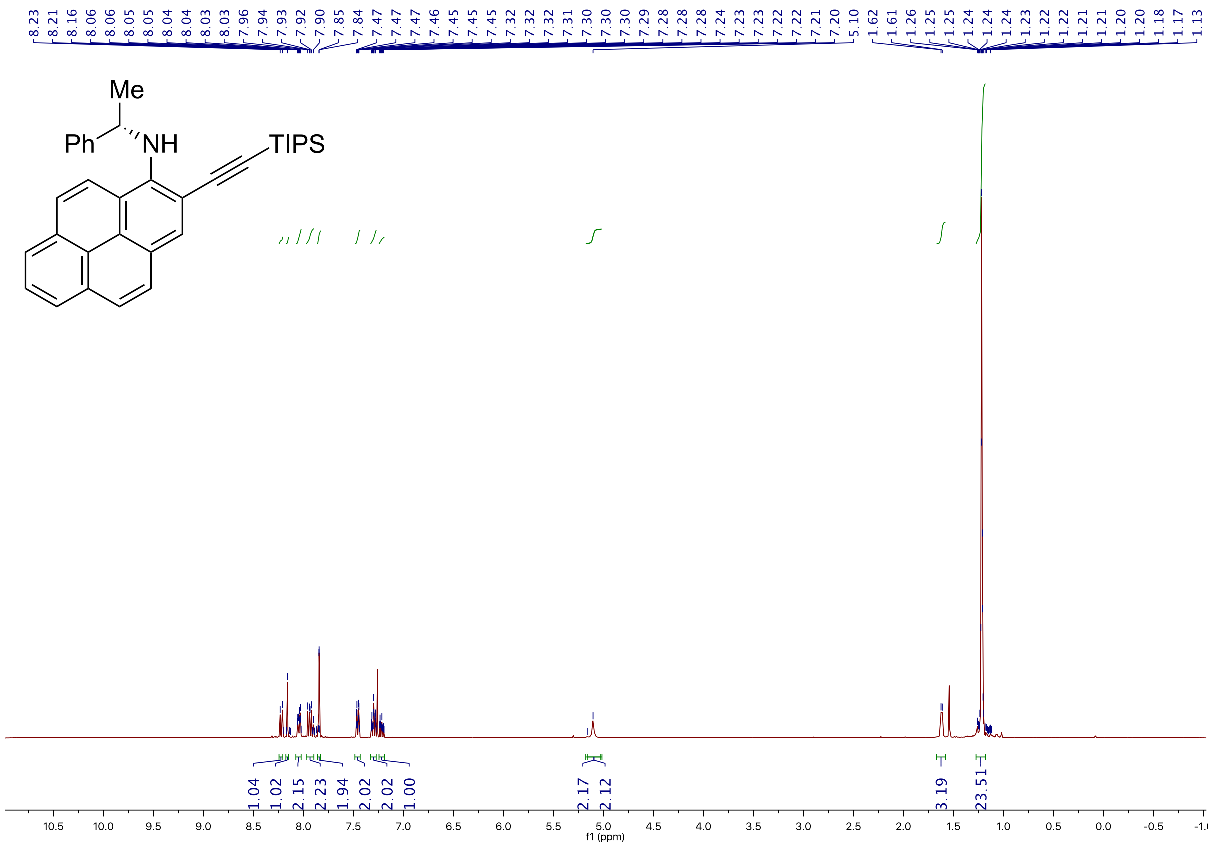

^{13}C NMR: 126 MHz, $CDCl_3$, compound (**9**)

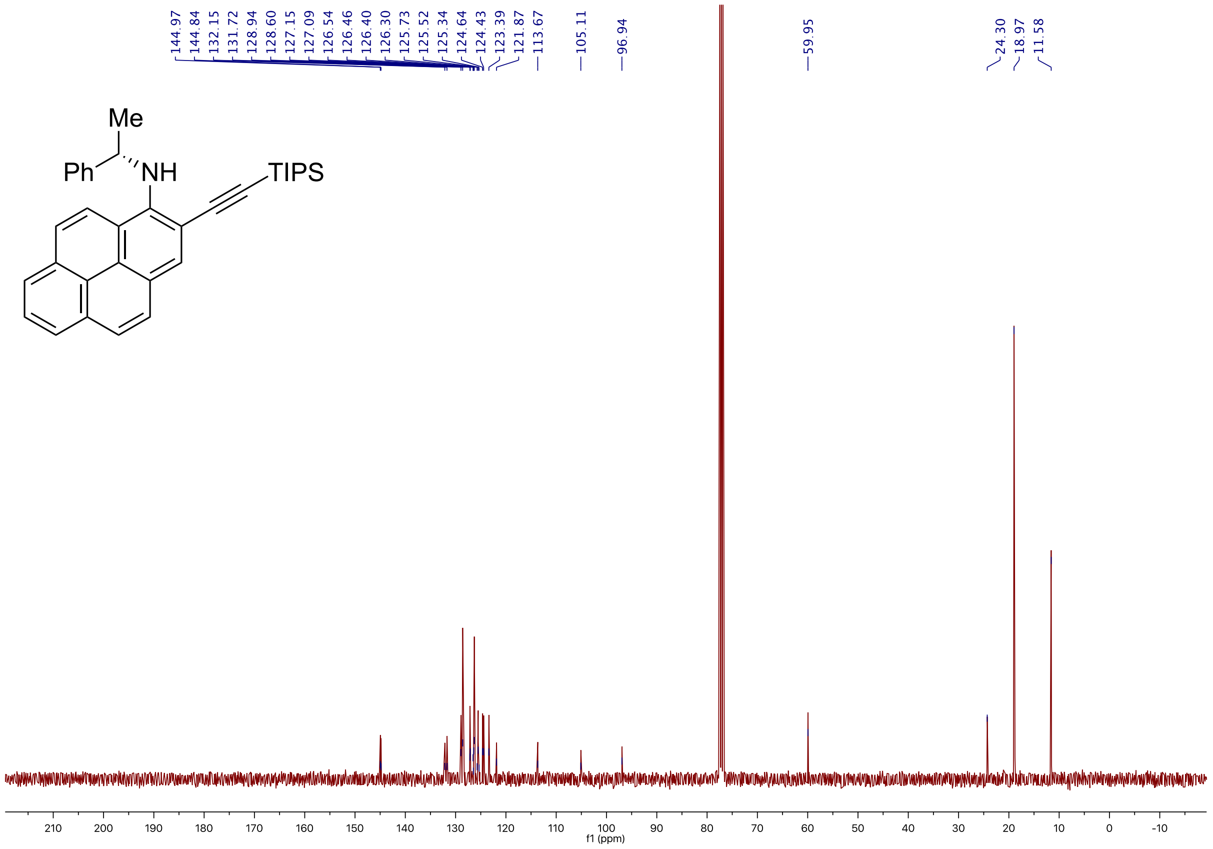

^{1}H NMR: 500 MHz, $CDCl_3$, compound (**10**)

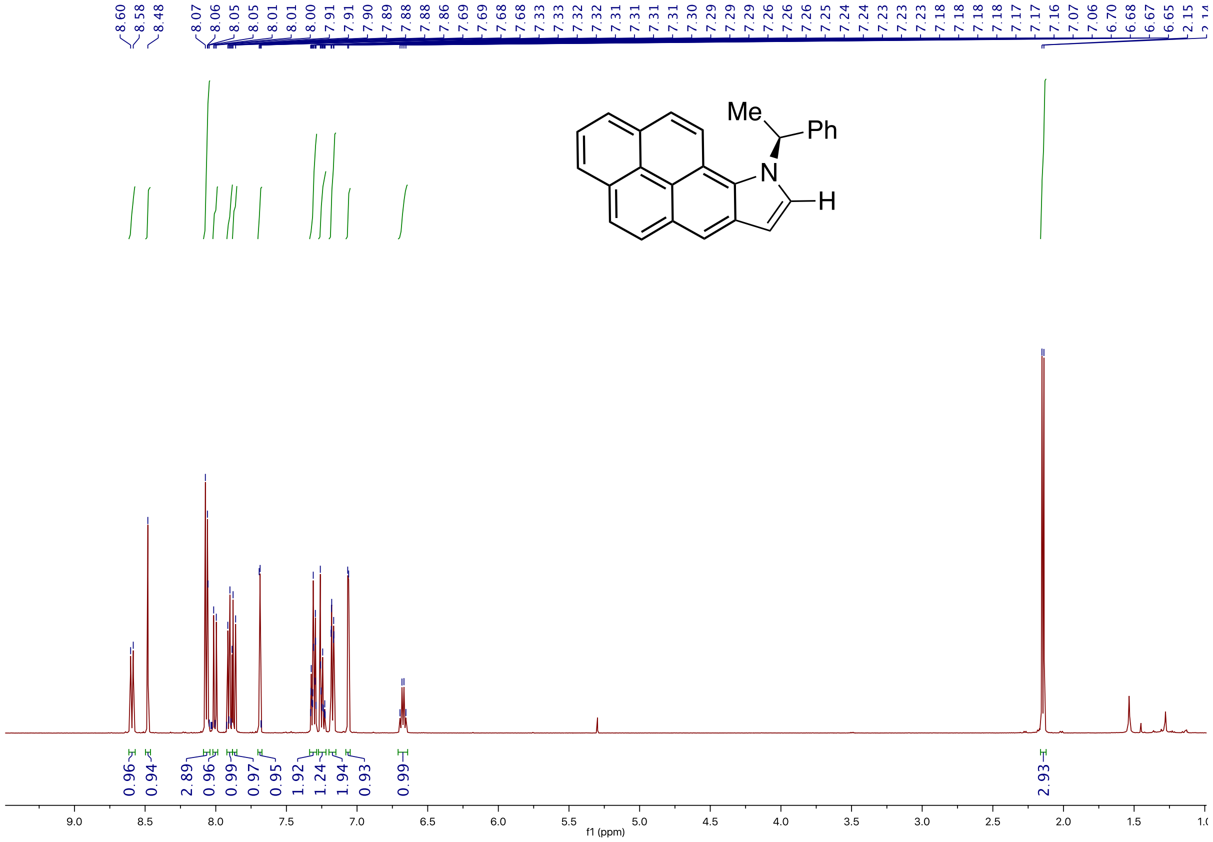

^{13}C NMR: 126 MHz, $CDCl_3$, compound (**10**)

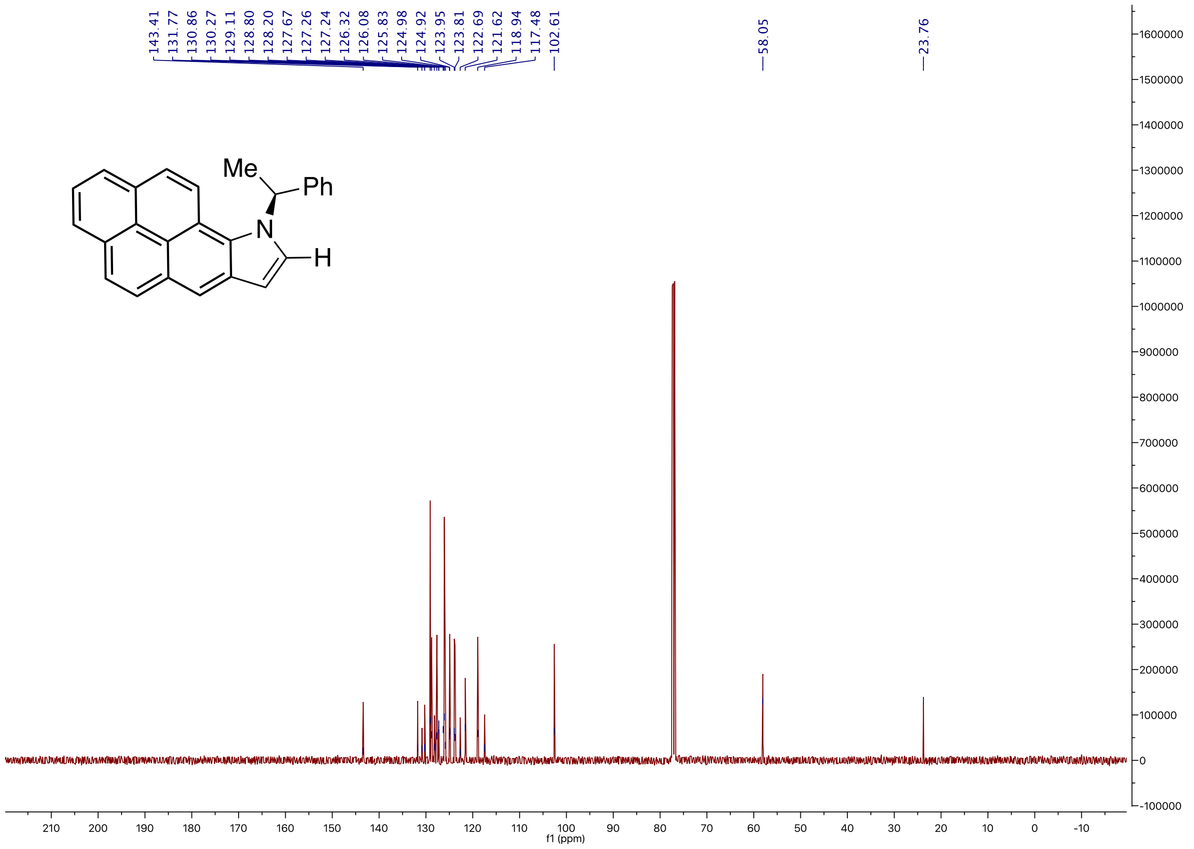

^{1}H NMR: 500 MHz, $CDCl_3$, compound (**11**)

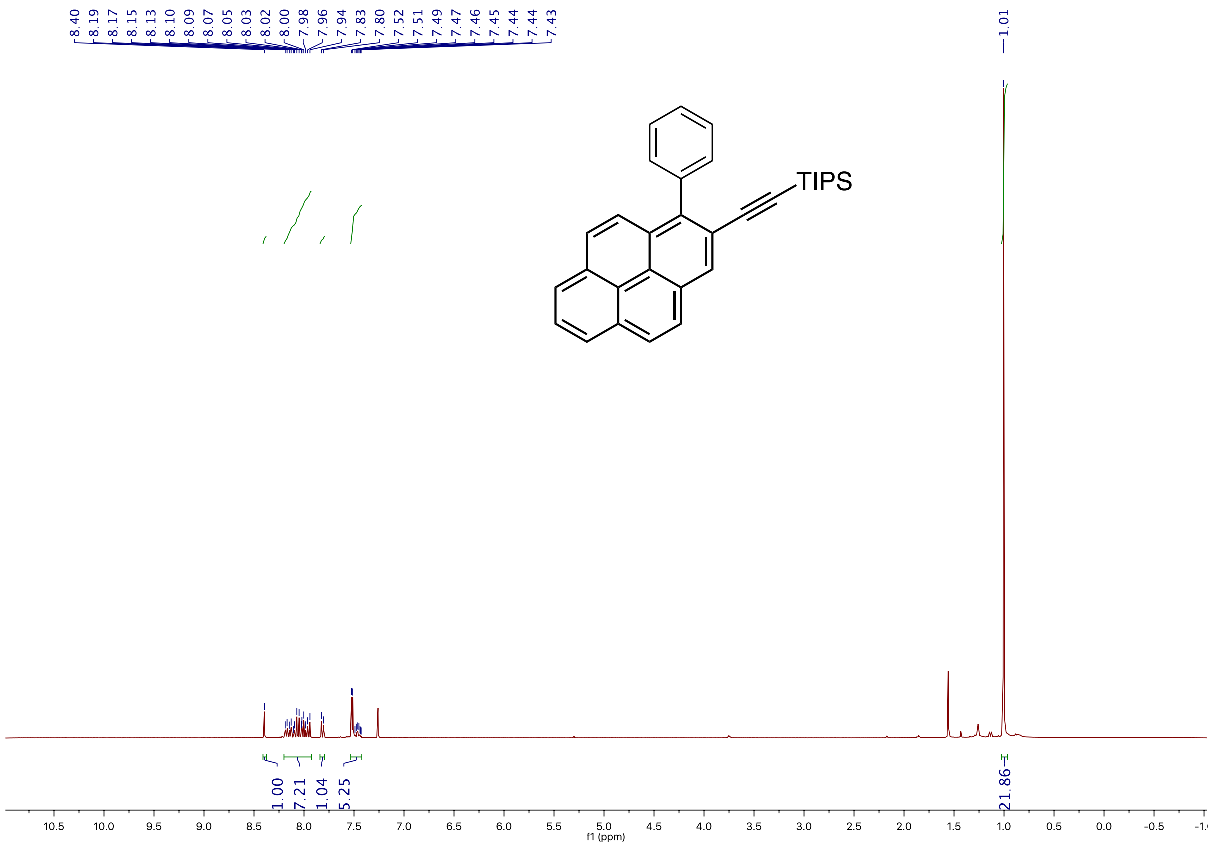

^{13}C NMR: 126 MHz, $CDCl_3$, compound (**11**)

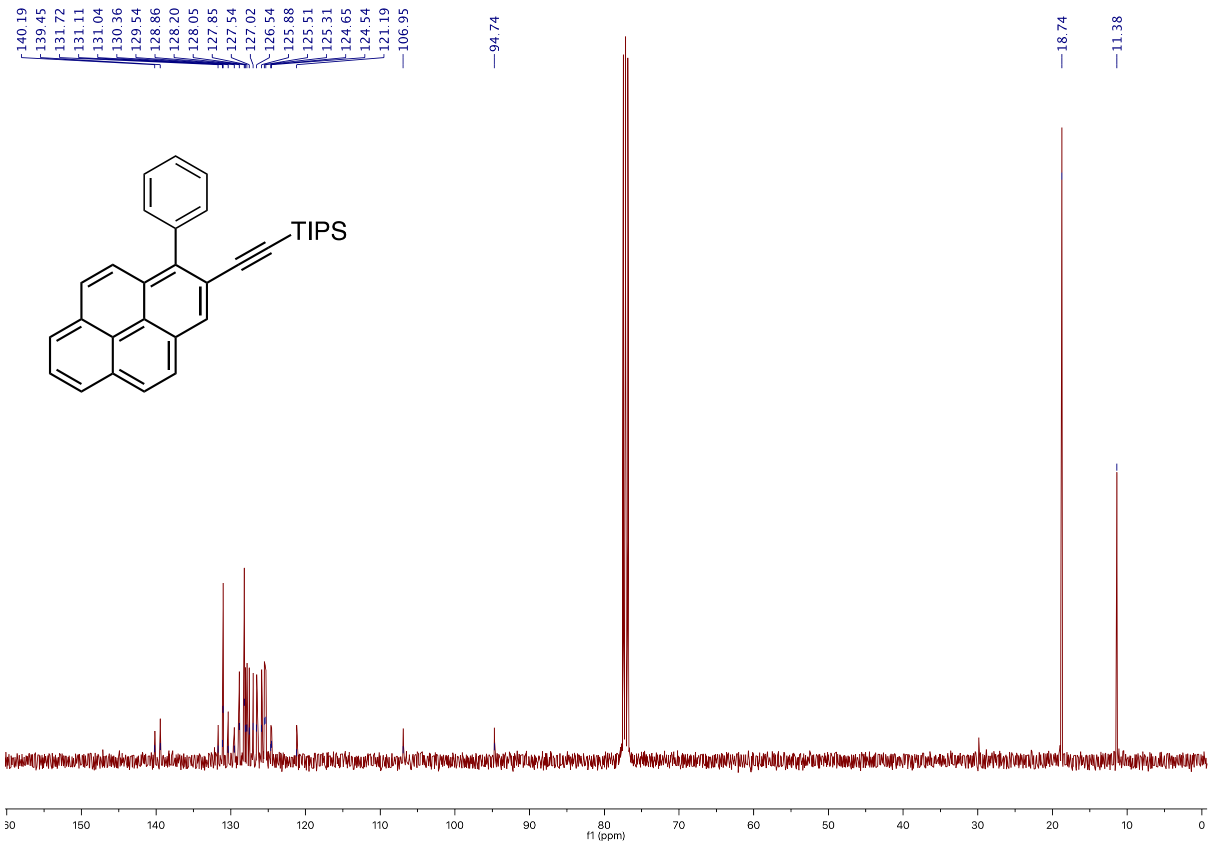

^{1}H NMR: 500 MHz, $CDCl_3$, compound (**13**)

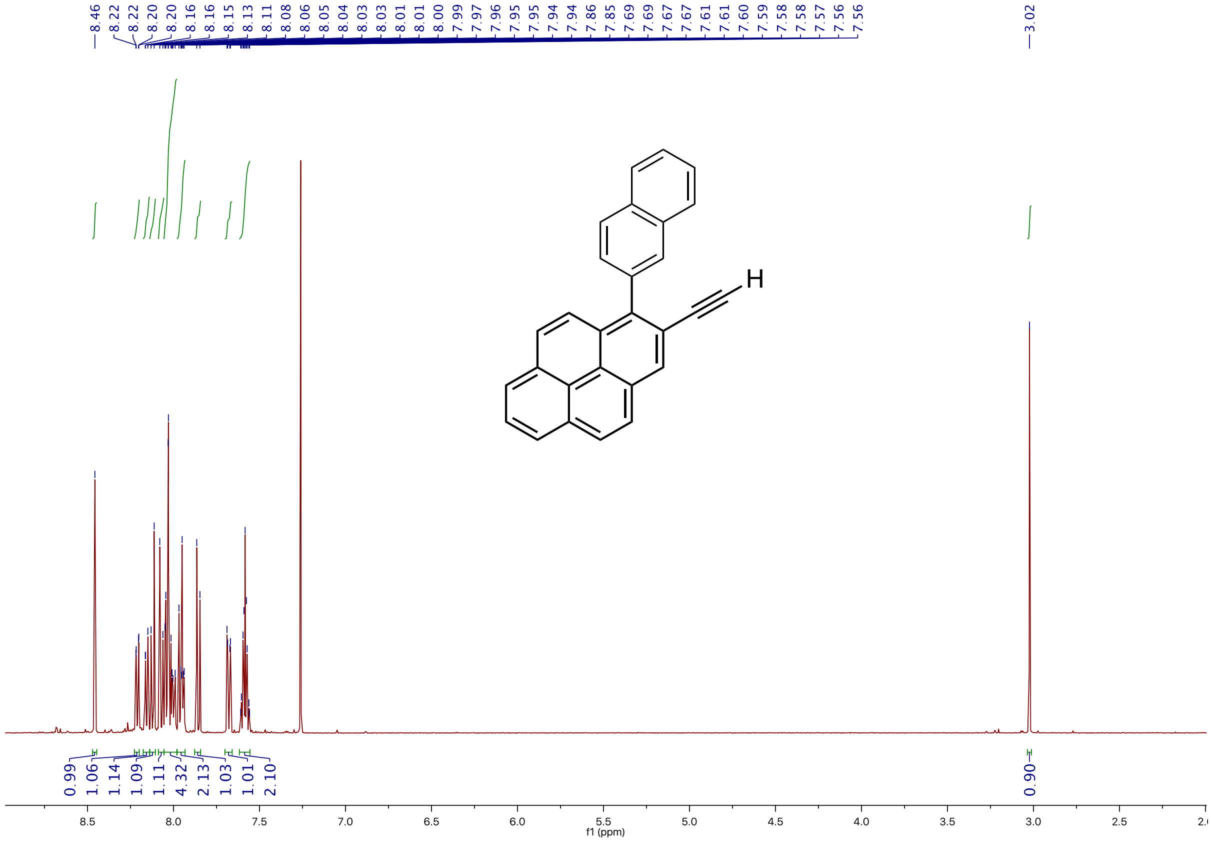

^{13}C NMR: 126 MHz, $CDCl_3$, compound (**13**)

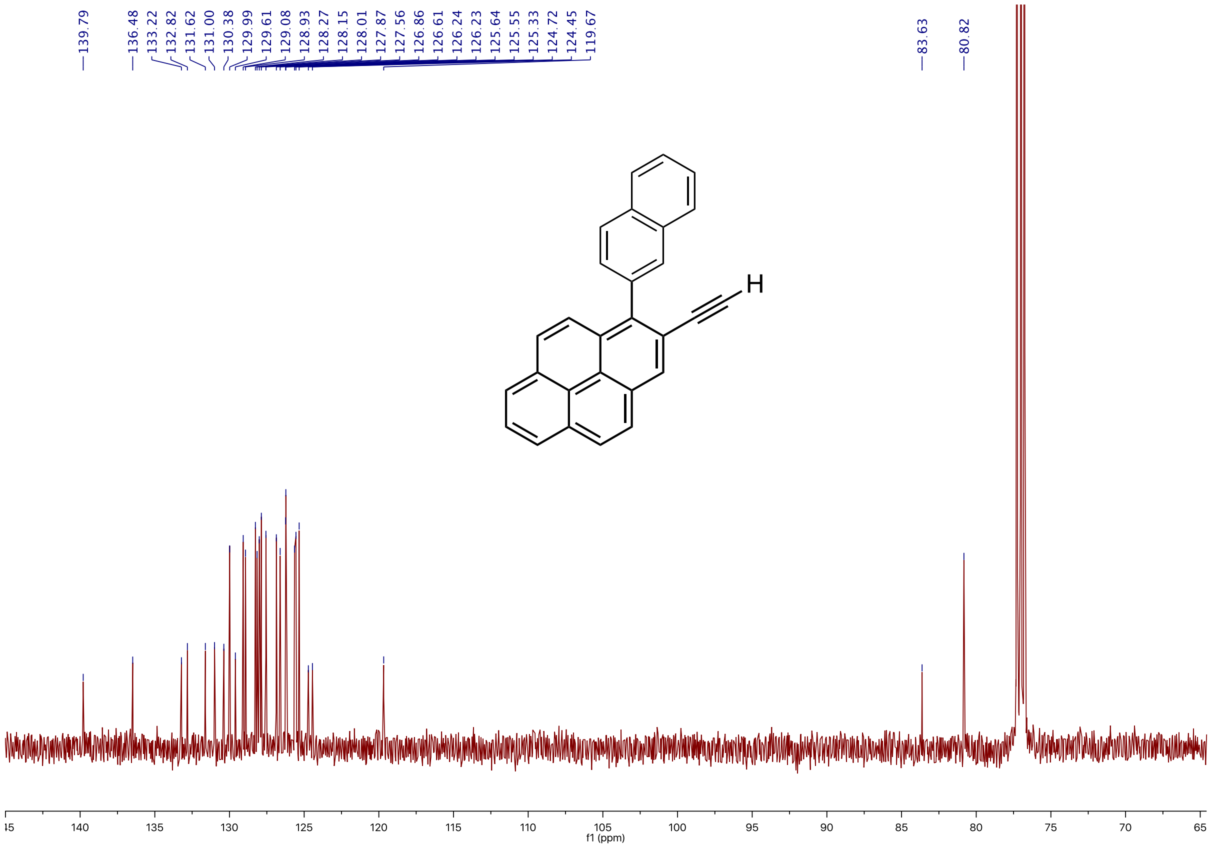

^{1}H NMR: 300 MHz, $CDCl_3$, compound **(14)**

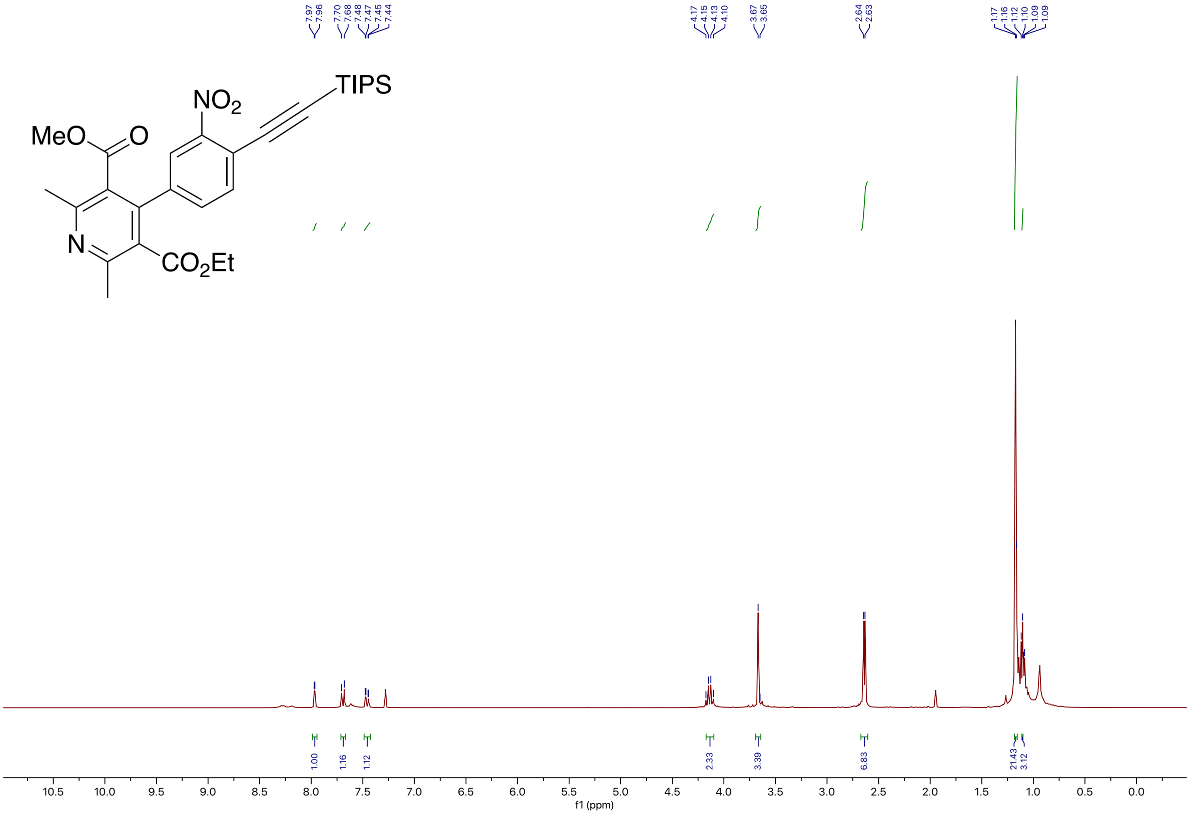

^{13}C NMR: 75 MHz, $CDCl_3$, compound **(14)**

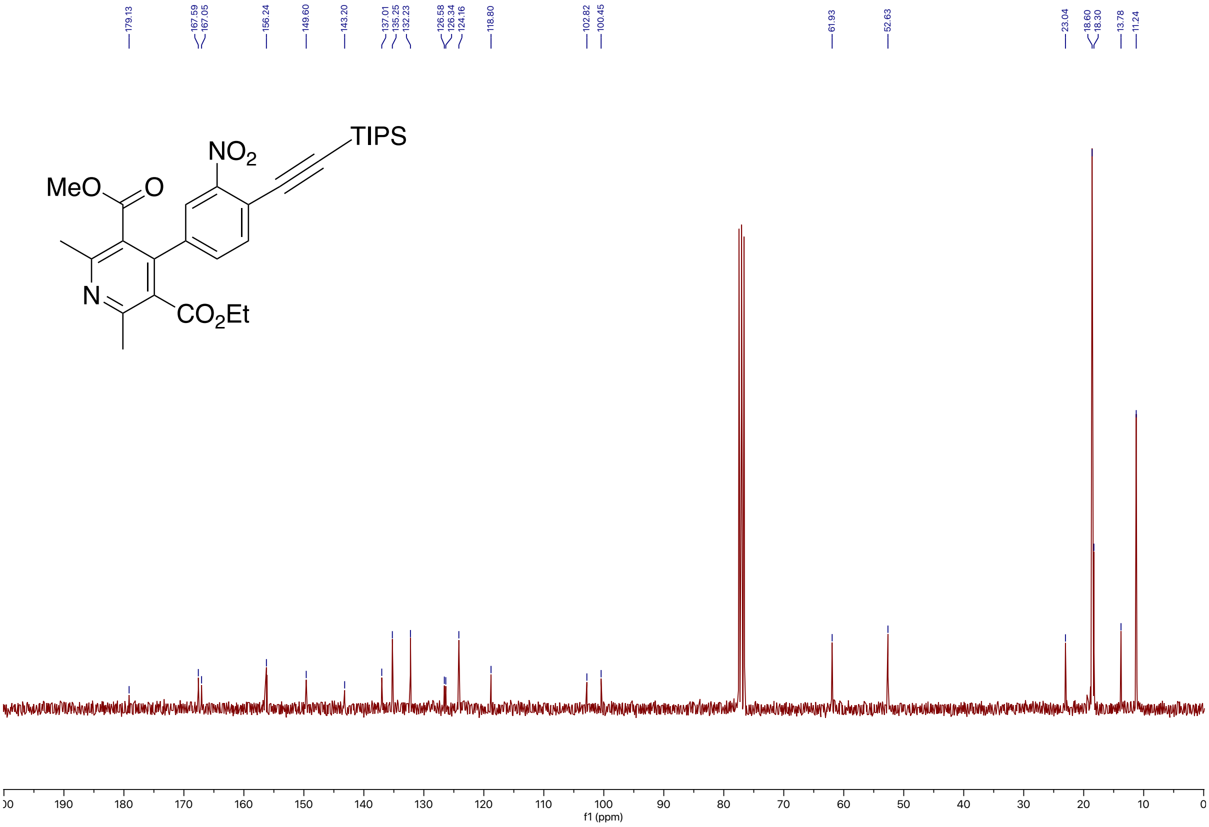

^{1}H NMR: 300 MHz, $CDCl_3$, compound **(16)**

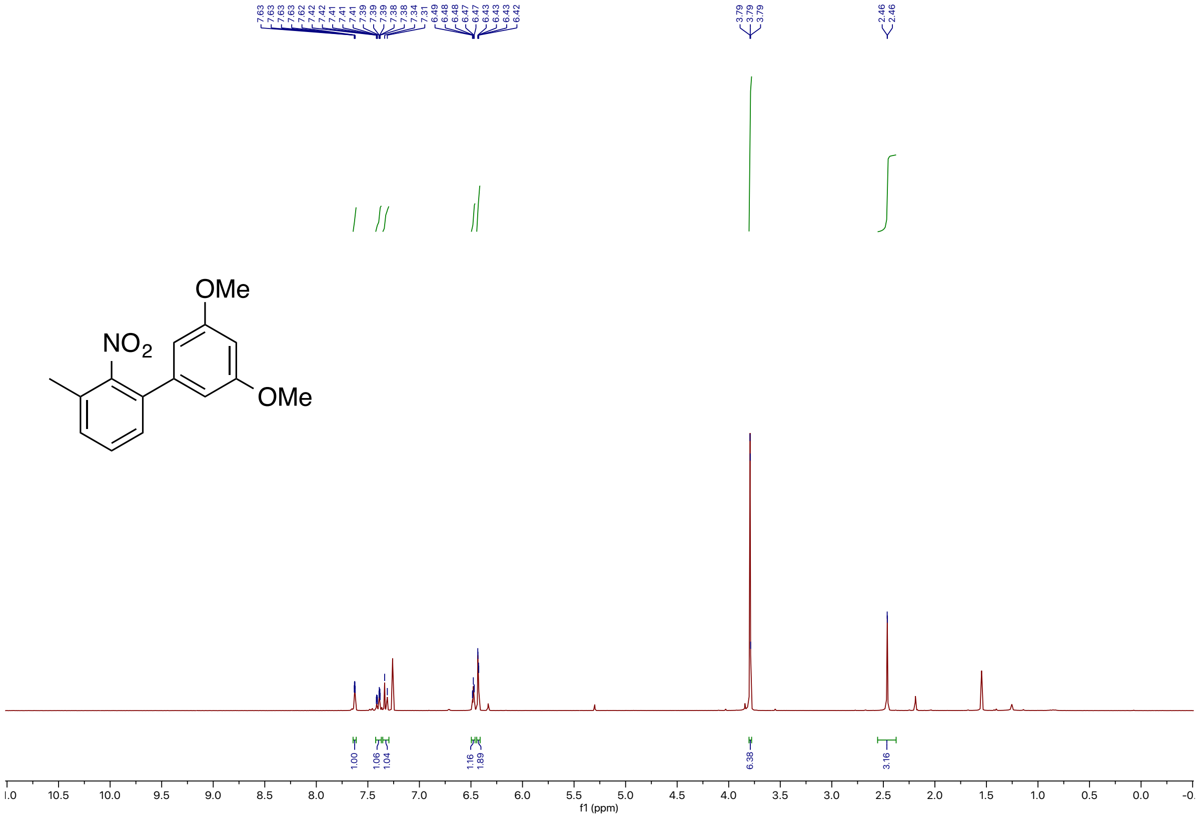

^{13}C NMR: 75 MHz, $CDCl_3$, compound **(16)**

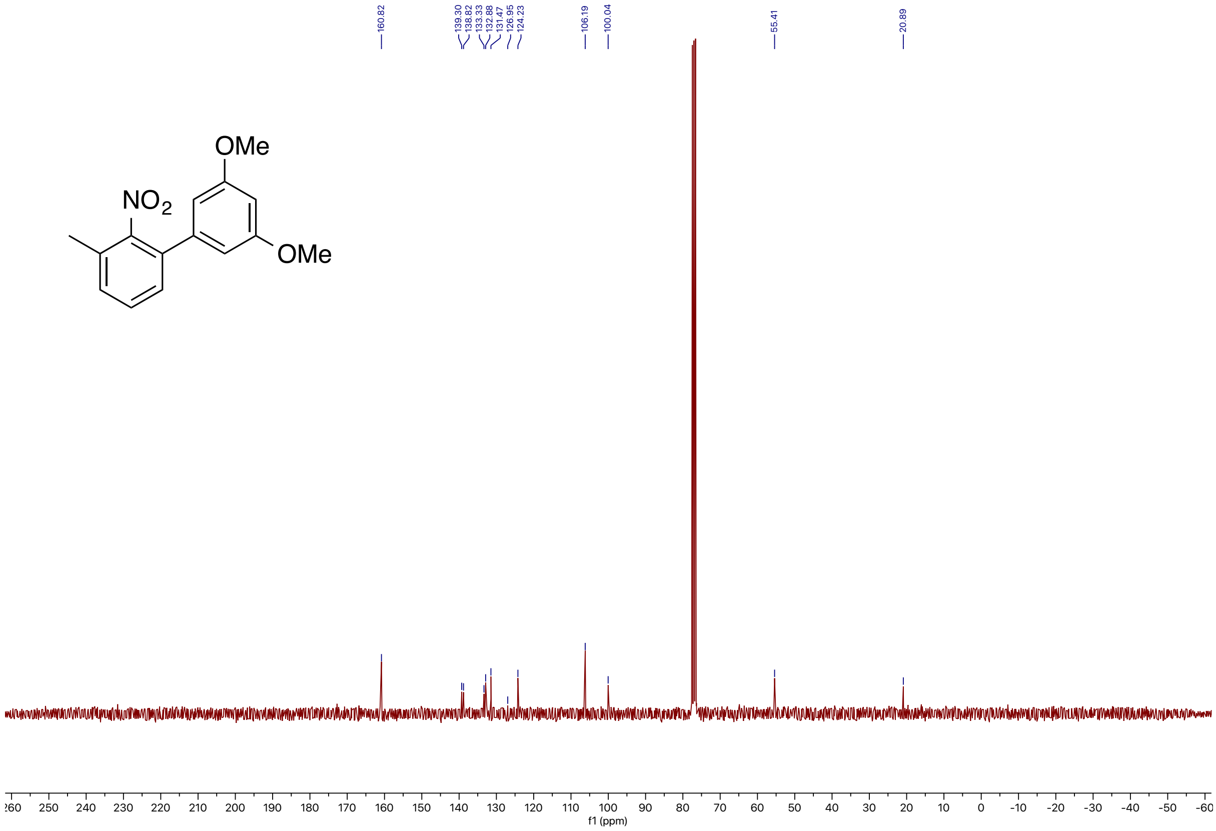

9. X-Ray crystallographic data

The structure of arylalkyne **3y** was confirmed by X-ray diffraction (CCDC/FIZ Karlsruhe deposition number 2099304)

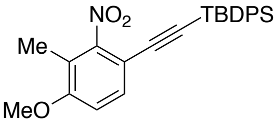

[1] (a) C. D. Campbell, R. L. Greenaway, O. T. Holton, P. R. Walker, H. A. Chapman, C. A. Russell, G. Carr, A. E. Thomson, E. A. Anderson, *Chem. Eur. J.* **2015**, *21*, 12627–12639. (b) T. Wada, M. Iwasaki, A. Kondoh, H. Yorimitsu, K. Oshima, *Chem. Eur. J.* **2010**, *16*, 10671 – 10674. (c) J. P. Brand, J. Waser, *Angew. Chem. Int. Ed.* **2010**, *49*, 7304 - 7307. (d) X. Nie, G. Wang, *J. Org. Chem.* **2006**, *71*, 4734-4741. (e) J. Skotnitzki, V. Morozova, P. Knochel, *Org. Lett.* **2018**, 20, 2365-2368. (f) P. A. Plé, T. P. Green, L. F. Hennequin, J. Curwen, M. Fennell, J. Allen, C. Lambert-van der Brempt, G. Costello *J. Med. Chem.* **2004**, *47*, 871-887. (g) V. Gasparotto, I. Castagliuolo, M. G. Ferlin, *J. Med. Chem.* **2007**, *50*, 5509-5513.

[2] M. S. Maier, K. Hüll, M. Reynders, B. S. Matsuura, P. Leippe, T. Ko, L. Schäffer, D. Trauner *J. Am. Chem. Soc.* **2019,** *141*, 17295–17304.

[3] C. M. Le, X. Hou, T. Sperger, F. Schoenebeck, M. Lautens *Angew. Chem. Int. Ed.* **2015**, *54*, 15897–15900.

[4] I. Sapountzis, H. Dube, R. Lewis, N. Gommermann, P. Knochel, *J. Org. Chem.* **2005**, *70*, 2445-2454.

[5] J.-D. Chai, M. Head-Gordon, *Phys. Chem. Chem. Phys.* **2008**, *10*, 6615–6620.

[6] Gaussian 09. Revision D.01.
M. J. Frisch, G. W. Trucks, H. B. Schlegel, G. E. Scuseria. M. A. Robb, J. R. Cheeseman, G. Scalman, V. Barone, B. Mennucci, G. A. Petersson, H. Nakatsuji, M. Caricato, X. Li, H. P. Hratchian, A. F. Izmaylov, J. Bloino, G. Zheng, J. L. Sonnenberg, M. Hada, M. Ehara, K. Toyota, R. Fukuda, J. Hasegawa, M. Ishida, T. Nakajima, Y. Honda, O. Kitao, H. Nakai, T. Vreven, Jr. J. Montgomery, J. E. Peralta, F. Ogliaro, M. Bearpark, J. J. Heyd, E. Brothers, K. N. Kudin, V. N. Staroverov, T. Keith, R. Kobayashi, J. Normand, K. Raghavachari, A. Rendell, J. C. Burant, S. S. Iyengar, J. Tomasi, M. Cossi, N. Rega, J. M. Millam, M. Klene, J. E. Knox, J. B. Cross, V. Bakken, C. Adamo, J. Jaramillo, R. Gomperts, R. E. Stratmann, O. Yazyev, A. J. Austin, R. Cammi, C. Pomelli, J. W. Ochterski, R. L. Martin, K. Morokuma, V. G. Zakrzewski, G. A. Voth, P. Salvador, J. J. Dannenberg, S. Dapprich, A. D. Daniels, O. Farkas, J. B. Foresman, J. V. Ortiz, J. Cioslowski, D. J. Fox, Gaussian. Inc. Wallingford CT. **2013**.

[7] a) P. Hay, W. Jeffrey, R. Willard, *J. Chem. Phys.* **1985**, *82*, 299–310. b) W. Jeffrey, R. Willard, P. Hay, *J. Chem. Phys.* **1985**. *82*. 284–298.

[8] a) R. Ditchfield, R. Hehre, W. J. Pople, *J. Chem. Phys.* **1971**, *54*, 724–728. b) M. M. Franci, W. J. Pietro, W. J. Hehre, *J. Chem. Phys.* **1982**, *77*, 3654–3665. c) M. S. Gordon, J. S. Binkley, J. A. Pople, W. J. Pietro, W. J. Hehre, *J. Am. Chem. Soc.* **1982**, *104*, 2797–2803. d) P. C. Hariharan, J. A. Pople, *Theor. Chim. Acta*. **1973**, *28*, 213–222. e) W. J. Hehre, R. Ditchfield, J. A. Pople, *J. Chem. Phys.* **1972**, *56*, 2257–2261.

[9] a) P. J. Hay, W. R. Wadt, *J. Chem. Phys.* **1985**, *82*, 299–310. b) L. E. Roy, P. J. Hay, R. L. Martin *J. Chem. Theory Comput.* **2008**, *4*, 1029–1031.

[10] a) T. Clark, J. Chandrasenkhar, G. W. Spitznagel, P. V. R. Schleyer, *J. Comput. Chem.* **1983**, *4*, 294–301. b) R. Ditchfield, W. J. Hehre, J. A. Pople, *J. Chem. Phys.* **1971**, *54*, 724–728. c) G. W. Spitznagel, T. Clark, P. V. R. Schleyer, W. J. Henre, *J. Comput. Chem.* **1987**, *8*, 1109–1116.

[11] A. V. Marenich, C. J. Cramer, D. G. Truhlar, *J. Phys. Chem. B.* **2009**, *113*, 6378–6396.

[12] C. Y. Legault, CYLview; Universite de Sherbrooke: Sherbrooke, Canada, **2009**; http://www.cylview.org.

[13] E. D. Glendening, J. K. Badenhoop, A. E. Reed, J. E. Carpenter, J. A. Bohmann, C. M. Morales, C. R. Landis, F. Weinhold, Theoretical Chemistry Institute, University of Wisconsin, Madison, **2013**.

[14] A. E. Reed, R. B. Weinstock, F. Weinhold, *J. Chem. Phys.* **1985**, *83*, 735–746.

[15] A. E. Reed, F. Weinhold, *J. Chem. Phys.* **1985**, *83*, 1736–1740.

[16] Chemcraft – https://www.chemcraftprog.com.

[17] D. L. Davies, S. M. A. Donald, S. A. Macgregor, *J. Am. Chem. Soc.* **2005**, *127*, 13754–13755.

[18] The corresponding intermediate $\mathbf{I_{Cp^*}}$ was found to be the most stable adduct for all the substituted nitrobenzenes used in the Hammett plot studies.

[19] H. Ryu, J. Park, H. K. Kim, J. Y. Park, S. -T. Kim, M. -H. Baik, *Organometallics* **2018**, *19*, 3228–3239.

[20] The corresponding intermediate $\mathbf{I_{Cp^*}}$ was found to be the most stable adduct for all the substituted nitrobenzenes used in the Hammett plot studies.
